# The SUMOylation Pathway Restricts Gene Transduction by Adeno-Associated Viruses

**DOI:** 10.1371/journal.ppat.1005281

**Published:** 2015-12-01

**Authors:** Christina Hölscher, Florian Sonntag, Katharina Henrich, Qingxin Chen, Jürgen Beneke, Petr Matula, Karl Rohr, Lars Kaderali, Nina Beil, Holger Erfle, Jürgen A. Kleinschmidt, Martin Müller

**Affiliations:** 1 German Cancer Research Center, Heidelberg, Germany; 2 VIROQUANT-CellNetworks RNAi Screening Facility, BIOQUANT Center University Heidelberg, Heidelberg, Germany; 3 Biomedical Computer Vision Group, Dept. Bioinformatics and Functional Genomics, University of Heidelberg, BIOQUANT, IPMB, and German Cancer Research Center, Heidelberg, Germany; 4 University Medicine Greifswald, Institute for Bioinformatics, Greifswald, Germany; University of North Carolina at Chapel Hill, UNITED STATES

## Abstract

Adeno-associated viruses are members of the genus dependoviruses of the parvoviridae family. AAV vectors are considered promising vectors for gene therapy and genetic vaccination as they can be easily produced, are highly stable and non-pathogenic. Nevertheless, transduction of cells *in vitro* and *in vivo* by AAV in the absence of a helper virus is comparatively inefficient requiring high multiplicity of infection. Several bottlenecks for AAV transduction have previously been described, including release from endosomes, nuclear transport and conversion of the single stranded DNA into a double stranded molecule. We hypothesized that the bottlenecks in AAV transduction are, in part, due to the presence of host cell restriction factors acting directly or indirectly on the AAV-mediated gene transduction. In order to identify such factors we performed a whole genome siRNA screen which identified a number of putative genes interfering with AAV gene transduction. A number of factors, yielding the highest scores, were identified as members of the SUMOylation pathway. We identified Ubc9, the E2 conjugating enzyme as well as Sae1 and Sae2, enzymes responsible for activating E1, as factors involved in restricting AAV. The restriction effect, mediated by these factors, was validated and reproduced independently. Our data indicate that SUMOylation targets entry of AAV capsids and not downstream processes of uncoating, including DNA single strand conversion or DNA damage signaling. We suggest that transiently targeting SUMOylation will enhance application of AAV *in vitro* and *in vivo*.

## Author Summary

SUMOylation is a post-translational modification in which a small protein (SUMO) is covalently attached to target proteins. Three key enzymes are controlling this modification: The E1 activating complex composed of the heterodimer Sae1/Sae2, the E2 conjugation enzyme Ubc9 and one of many E3 enzymes which specifically recognize the target protein. SUMOylation regulates many processes such as protein stability, intracellular localization and protein-protein interactions. In our study we identified SUMOylation to be regulating transduction of cells by the human parvovirus adeno-associated virus (AAV). Targeting the E1 or E2 complex by RNA interference led to increased AAV transduction. We also identified putative E3 enzymes involved in this mechanism. Our data indicates that this regulation is mediated by the AAV capsid and it affects different AAV serotypes. Targeting SUMOylation might be a strategy to enhance AAV gene transduction.

## Introduction

Adeno-associated viruses (AAV) are members of the family parvoviridae which encompasses the subfamily of dependoparvovirus so named because they require helper viruses such as adenovirus, herpes simplex virus or human papillomavirus for replication in cell culture [1–5]. They comprise a single stranded genome of about 4.7 kb which is packaged into an icosahedral capsid of a T = 1 symmetry formed by the three capsid proteins VP1, VP2 and VP3. A number of different AAV serotypes have been isolated from human and non-human primate samples but also from other species [6–13]. Infection of humans with AAV is considered to be apathogenic [14, 15] which has been a major determinant in the development of AAV vectors for *in vivo* applications. Recombinant AAV vectors (rAAV) are stripped of all viral genes, the only cis-acting genetic element required are the two inverted terminal repeats (ITRs). Thus the AAV coding region can be replaced by heterologous expression cassettes and all factors required for vector production can be provided in trans, including the helper virus functions [16]. rAAV can be produced efficiently to large scale and they have been used for therapy of genetic disorders such as hemophilia B and blindness [17–23]. Transduction with AAV *in vivo* can lead to long term gene transfer in non-proliferating tissues but existing anti-AAV humoral immune responses as well as cytolytic T-cell responses induced against the transgene or against the virus capsid remain a major challenge (for review see [24, 25]. In 2012 a rAAV1 vector (Alipogene tiparvovec) for the treatment of lipoprotein lipase deficiency has been licensed by the European Medicines Agency under the trade name Glybera [26, 27].

A major limitation of AAV vectors is the rather inefficient transduction efficiency by AAV observed *in vivo* and in vitro in the absence of a helper virus. This requires use of high doses of AAV vectors for transduction which in consequence requires not only large efforts in vector production but also bears the risk of inducing vector-directed immune responses or adverse events. The low efficiency of transduction can be attributed to certain rate limiting steps in the early virus life cycle, namely cell uptake, escape from the endosomal compartment, nuclear entry, uncoating and conversion of the single strand DNA into a double strand (for review see: [28–30]. While the last step can be avoided by the use of self-complementary vectors (scAAV, with coding capacity reduced to about 50%; [31]), overcoming the remaining bottlenecks in virus entry remains a challenge. In the past, a number of cellular factors interfering with AAV transduction have been described, among them APOBEC3A and PML, [32–34]. Further, AAV transduction is controlled by ubiquitination and phosphorylation. Suppression of either function leads to increased transduction and it has been shown that AAV is a direct target of these post-translational modifications [35–38]. Along this line it has been shown that AAV can use alternative pathways for entry with different transduction efficiencies [39, 40].

Entry pathways of viruses into cells have been analyzed meticulously using chemical inhibitors, dominant negative cellular mutants and by complementation analysis using expression libraries (for review see [41]. In the recent years, a number of siRNA library screens have been performed to identify host dependency and host restriction factors for virus entry [42–47]. Previously, the results of a siRNA screen for AAV2 transduction of human airway epithelial cells have been reported [48]. Here, the authors documented that knockdown of their top candidates of the screen deregulated interferon response pathways.

To identify host cell dependency (HDF) and restriction factors (HRF) for AAV2-mediated gene transfer we performed a screen with two different siRNA libraries targeting a total of 21,264 cellular genes. The screen revealed a total of 921 hits, consisting of 740 putative host cell restriction factors. Intriguingly, a number of putative restriction factors clustered in the SUMOylation pathway. The first three top ranked factors negatively regulating AAV transduction were Sae1, Sae2 and Ubc9, the central players in the SUMOylation pathway (for review see [49]). We validated the findings of the siRNA screen using different reporters and different AAV serotypes. Our findings indicate that SUMOylation negatively affects vectors with single strand as well as self-complementary genomes. Furthermore, different AAV serotypes and modified AAV capsids are affected by SUMOylation. We assume that the effect of SUMOylation on AAV transduction is capsid dependent since expression of reporter genes from transfected AAV vector DNA is not affected by SUMOylation. Likewise, transduction of cells by human papillomavirus vectors and the AAV-related autonomous parvovirus H1 is not restricted by the SUMOylation pathway. Taken together we conclude that SUMOylation restricts AAV-mediated gene transfer. This finding encourages and outlines strategies for increasing transduction efficiency by recombinant AAV.

## Material and Methods

### Cell lines and Viruses

HeLa (ATCC-CCL-2) and HEK293T (ATCC ACS-4500) were maintained at 37°C and 5% CO_2_ in Dulbecco’s modified Eagle’s medium supplemented with 10% FCS, 100 U penicillin, 100 mg streptomycin and 2 mM L-glutamine. ATM^-/-^ (GM05849 from Coriell Institute, Camden, USA) and wt-human fibroblast cells (GM00637 Coriell Institute) [50, 51] were maintained at 37°C and 5% CO_2_ in modified Eagle’s medium supplemented with 10% FCS, 100 U penicillin, 100 mg streptomycin and 2 mM L-glutamine. HeLa cells stably transfected with a CMV-GFP reporter construct have been described previously [52]. H1 parvovirus and HPV16 and HPV18 pseudovirions were produced as previously described [53, 54]. Authentication and absence of contamination of cell lines was confirmed by multiplex cell contamination test and multiplex cell authentication by Multiplexion (Heidelberg, Germany).

For production of recombinant AAV vectors 5 x 10^5^ HEK 293T cells were seeded in a 15 cm tissue culture dish 24 h prior to transfection (37°C, 5% CO2, 95% humidity). Transfection mix was prepared by diluting the desired plasmids in non-supplemented DMEM. After the addition of TurboFect (LifeTechnology, Darmstadt, Germany), the mix was briefly mixed and incubated for 20 min at room temperature. After dropwise addition, cells were incubated at least 48 h in the incubator at 37°C, 5% CO2, 95% humidity. Cells were harvested by detachment with a cell lifter, washed with PBS and resuspended in AAV-lysis buffer (150 mM NaCl, 50 mM Tris, pH 8.5). Subsequently, cells were crushed by five freeze-thaw cycles (liquid nitrogen/ 37°C water bath). 50 U/ml benzonase (30 min at 37°C) were used to degrade unpacked genomes and plasmid-DNA. After a centrifugation step at 5000 x g at 4°C, supernatant was prepared for ultracentrifugation. To separate the AAV from viral and cellular proteins, the supernatant was loaded onto an iodixanol step gradient (7 ml of 15% iodixanol in PBS-MKN [1 mM MgCl_2_, 2.5 mM KCl, 0.75 M NaCl] and 5 ml of 25% iodixanol, 4 ml of 40% iodixanol, and 4 ml of 60% iodixanol in PBS-MK [1 mM MgCl_2_, 2.5 mM KCl]) in Beckman Quickseal tubes (25 by 89 mm). After the gradient was ultracentrifuged for 2 h at 50000 rpm, 10°C (Beckman, 70.1 Ti-Rotor), viruses have migrated through the phases of different viscosity and could finally be extracted from the virus containing layer (40% iodixanol).

### Purification and transfection of AAV genomes

Iodixanol-purified ssAAV2-firefly luciferase (2x10^11^ genome equivalents) were used to isolate viral genomes with the Qiagen kit QIAamp MinEluteVirusSpin according to the manufacturer’s handbook. DNA was eluted with 40 μl sterile water. Forty-four h after siRNA transfection of HeLa cells on 24-well plates, medium was replaced with fresh DMEM supplemented with 10% FCS. Two hours later, DNA-transfections were carried out by using 2.8 μl Turbofect (Life Technologies, Darmstadt, Germany) transfection reagent together with 8 μl of eluated AAV vector DNA and 1.6 μg pBKS plasmid DNA as carrier per well according to the manufacturer’s protocol. Twenty-four hours after transfections, cells were harvested and luciferase assays performed.

### siRNA transfections and reporter gene assays

Transfections of siRNAs were conducted according to the fast-forward transfection protocols using HiPerFect transfection reagent from Qiagen. For FACS-analysis 1.5 x 10^5^ HeLa-cells or HeLa-GFP-cells per well of a 6-well plate were plated, followed by transfection with 100 ng siRNA in 100 μl unsupplemented DMEM plus 12 μl HiPerFect transfection reagent. Alternatively, 8 x 10^4^ SV40-transformed fibroblast cells (wt and ATM-/- from Coriell Institute, USA) per well of a 6-well plate were plated followed by transfection with 300 ng siRNA per well in 100 μl unsuplemented DMEM plus 18 μl HiPerFect reagent. Forty-eight hours after siRNA transfections, cells were infected with scAAV2-CMV-eGFP at an MOI_GC_ (multiplicity of infection determined by vector genome copies) of 10^3^. Twenty-four hours after infections, cells were harvested by trypsinization, washed twice with PBS and either used entirely for FACS analysis or one half was used to prepare whole cell lysates for western blot analysis. FACS analysis was performed on a FACS Calibur instrument from Becton Dickinson (Heidelberg, Germany). For luciferase assays 2.5 x10^4^ HeLa-cells were plated per well of a 24-well plate. Cells were transfected with 37.5 ng or 75ng siRNA per well in 100 μl unsupplemented DMEM containing 4.5 μl HiPerFect. AAV-infections were carried out with ssAAV2-firefly luciferase, scAAV2-, scAAV5- and scAAV9-renilla luciferase 46 h after siRNA transfections at an MOI_GC_ of 10^3^ or 10^4^. Twenty-four hours after infections, the cells were incubated for 15 minutes at RT in 50 μl 1 x passive lysis buffer as supplied by the manufacturer (PJK GmbH; Kleinbittersdorf, Germany) on a shaker. Subsequently, 10 μl of the lysate were transferred into a white 96-well plate in duplicates. For luciferase detection, either 100 μl renilla glow juice with coelenterazine (PJK GmbH; 1:50) or 100 μl beetle juice (PJK GmbH) for firefly luciferase detection were added to each well and incubated for 5 minutes at RT. The luminescence was analyzed using the Perkin Elmer Wallac Work station (Perkin Elmer, Rodgau, Germany) and data were processed in Microsoft Excel and GraphPad Prism 6.0.

### Western blot

Cell extracts were separated on a 12.5% SDS-PAGE gel and blotted onto nitrocellulose filters. For detection of Sae2 a rabbit anti-Sae2 monoclonal antibody (D15C11; Cell Signaling, Boston, USA) was used according to manufacturer’s instructions. Detection of Ubc9 was performed by separating extracts on NuPAGE Bis-Tris 4–12% Mini Gels in MOPS SDS buffer (50 mM MOPS, 50 mM Tris base, 0.1% SDS, 1 mM EDTA, pH 7.7) according to instructions of the manufacturer (Life Technologies, Darmstadt, Germany). After blotting using a semidry chamber and EMBL-blot buffer (40 mM Tris, 39 mM glycine, 1.3 mM SDS, 20% methanol, in H_2_O, pH 8.2) the nitrocellulose was incubated in PBS, 0,1% Tween containing 5% dry milk powder and a 1:1000 dilution of a polyclonal rabbit antibody kindly provided by the group of Prof. Frauke Melchior, ZMBH, University of Heidelberg.

### Cell fractionation and DNA extraction

To determine AAV binding and entry, Hela cells were cooled to 4°C 48h post transfection of siRNAs and incubated with AAV vectors at a MOI of 10^4^ for 1 h at 4°C. Cells were then washed with medium and shifted to 37°C for various time. Cells were harvested by trypsin and proteinase K digestion and DNA was extracted using the DNeasy blood & tissue kit (Qiagen, Hamburg, Germany). AAV vector genomes were quantified by qPCR [55]. To determine the subcellular distribution of AAV vectors, Hela cells were transfected and transduced as above. However, cells were harvested 12 hours post infection and fractionated using the Qproteome cell compartment kit (Qiagen, Hamburg, Germany) according to the manufacturer’s protocol except that no proteinase inhibitor nor benzonase were used. DNA was extracted from the cell fractions as above and AAV DNA quantified by qPCR.

### High-throughput siRNA screen

In order to identify cellular proteins having an effect on rAAV-2 transduction, a high-throughput screen of siRNA libraries was performed in two replicates using the solid-phase reverse transfection method described by Erfle and colleagues [56]. Genome-wide analysis was accomplished by arraying siRNAs of two complementary libraries in 384-well plates: The *Extended Druggable Silencer siRNA Library* targeting 9,102 human genes (3 siRNAs per gene; 3.6 pmol of lyophilized siRNA per well) and the *Genome Extension Silencer Select siRNA Library* targeting 12,162 human genes (3 siRNAs per gene; 0.36 pmol of lyophilized siRNA per well) both obtained from Ambion/Applied Biosystems (Kaufungen, Germany). Each well of the plates contained only one individual siRNA.

HeLa Kyoto cells [57] were seeded in “ready to transfect” 384-well plates in supplemented Dulbecco`s modified Eagle`s medium (DMEM with 10% heat-inactivated fetal calf serum, 100 U/ml penicillin, 100 μg/ml streptomycin and 2 mM L-glutamine) at 600 cells and 30 μl per well. For siRNA-mediated knockdown of gene expression, cells were cultivated for 48 h at 37°C and 5% CO_2_ before they were transduced with 1,000 viral genomes per cell of an iodixanol step gradient purified rAAV-2 stock comprising a self-complementary vector genome coding for eGFP under the control of a CMV promoter (scAAV2-CMVeGFP) [58]. Twenty-four hours post transduction cells were fixed in 1% paraformaldehyde (PFA) for at least 30 min, washed three times in PBS and then transferred into 1% PFA supplemented with 70 ng/ml Hoechst 33342.

Image acquisition of *EGFP* expression and Hoechst stained cell nuclei was performed with an automated high-throughput microscopy platform equipped with wide-field screening microscope Olympus Biosystems IX81 and Scan^R data acquisition software version 2.3.0.5 (Olympus, Münster, Germany). The acquired screening data was automatically analyzed and quantified using an extension and adaption of the image analysis method in [59]. Statistical analysis based on the image analysis results was performed as described previously [60].

### Accession Numbers

#### siRNAs

AllStars Negative Control siRNA and siRNAs targeting the SUMOylation pathway from Qiagen (Hamburg, Germany) were used:

Hs_UBE2I_8  ACCACCATTATTTCACCCGAA  2117060

Hs_UBE2I_9  AAGGGTCCGAGCACAAGCCAA  2117061

Hs_UBE2I_1  AAGGGATTGGTTTGGCAAGAA  2117062

Hs_UBE2I_6  CAAGAAGTTTGCGCCCTCATA  2117063

Hs_SAE2_3  CACCGGTTTCTCCCACATCGA  2128851

Hs_UBA2_5  GTGCGGCTGAATGTCCATAAA  2128852

Hs_SAE2_1  TTGGACTGGGCTGAAGTACAA  2128853

Hs_SAE2_2  TCCGACAGTTTATACTGGTTA  2128854

Hs_SAE1_8  CACGAACAGGTAACTCCAGAA  2128855

Hs_SAE1_9  ACCTGATACCTTATAGAGAAA  2128856

Hs_SAE1_2  TCCAGGGATGTCATAGTTAAA  2128857

Hs_SAE1_6  CAGGGCTATGTTGGTCCTTTG  2128858

## Results

### Screening of siRNA libraries identifies putative AAV restriction factors

**Fig 1:**
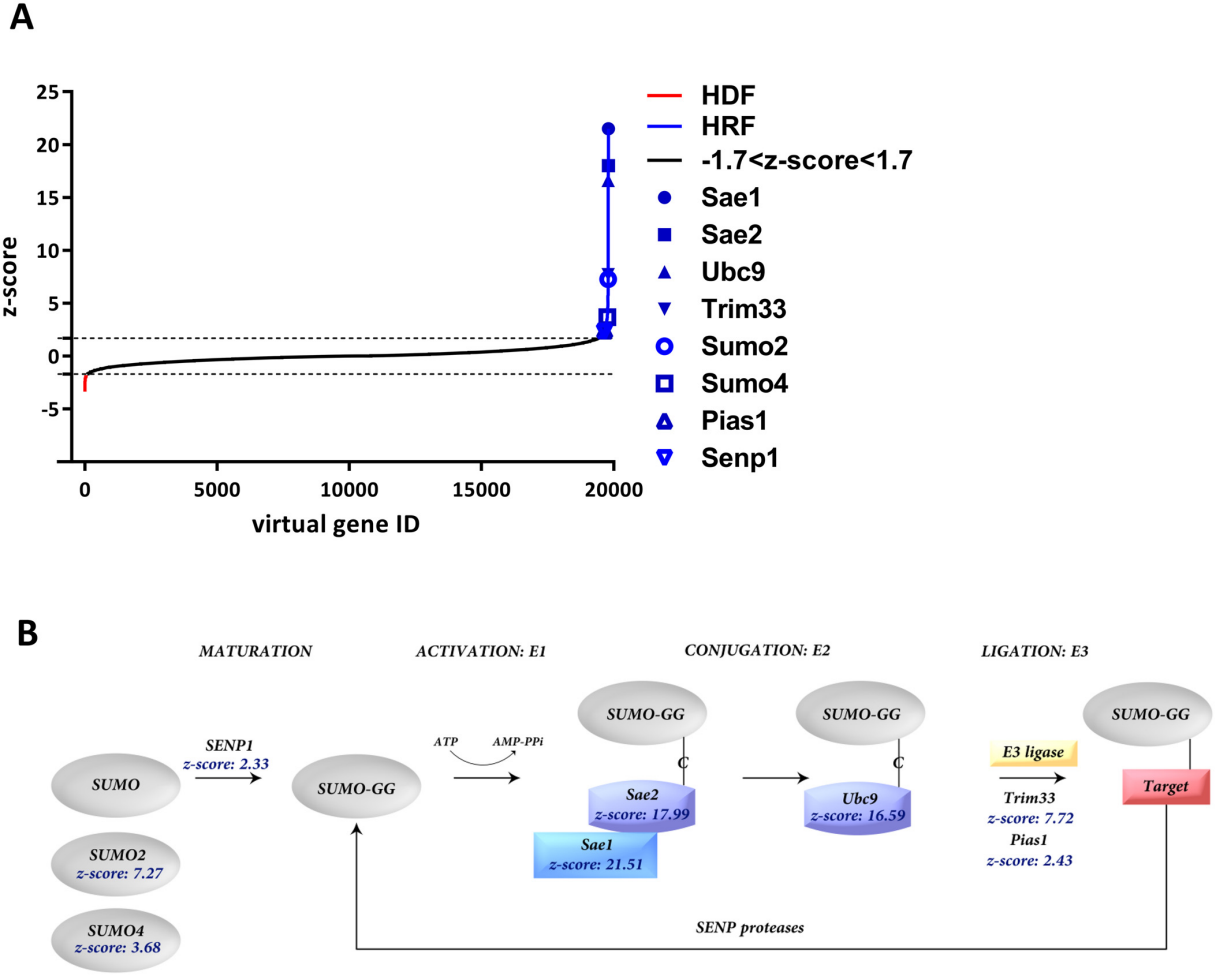
Screen of two genome-wide siRNA libraries reveal that the SUMOylation pathway controls AAV transduction. (a) Distribution of z-scores. A total of 20,290 genes are shown. The dashed lines indicate a z-score threshold of +/- 1.7 resulting in a total of 921 hits, consisting of 740 putative host cell restriction factors (HRF; z-score > 1.7) and 181 putative host cell dependency factors (HDF; z-score < -1.7). Hits concerning gene products of the SUMOylation pathway are indicated. (b) SUMOylation pathway factors and their z-scores identified in the screen are indicated (adapted from [76])

To identify host cell factors involved in AAV gene delivery, two complementary siRNA libraries targeting 9,102 and 12,162 genes, respectively, were used to inhibit single protein expression during vector-mediated gene transduction. The libraries were coated on 384 well plates followed by seeding of HeLa cells for 48 h upon which cells were transduced with rAAV2 (1000 genome copies per cell: MOI_GC_), harboring a self-complementary vector genome encoding eGFP under control of a CMV_i.e._ promoter (scAAV-GFP). Each library contained three different siRNAs per gene, thus, a total of 63,792 siRNAs were analyzed. Twenty-four hours post infection, cells were stained with Hoechst and automated image acquisition was performed to quantify the GFP fluorescent signals. Subsequently, hits were identified after log-transformation of raw data and normalization between different plates. siRNAs that showed cytotoxic effects, i.e. wells with the lowest 5% of cell counts, were identified and excluded from further analysis. Based on the image analysis, a z-score [60] was assigned to each gene. Using a threshold of +/-1.7 (corresponding to p<0.05) a total of 921 hits, consisting of 740 putative host cell restriction factors (z-score >1.7) and 181 putative host cell dependency factors (z-score<-1.7) were identified (Fig 1a). Among the high scoring host cell dependency candidates, factors of different cellular compartments were identified. Intriguingly, the three top scorers of the putative host cell restriction factors were identified to be key enzymes of the SUMOylation pathway: Sae1, Sae2 and Ubc9 (Fig 1a and 1b and table 1). Six out of six siRNAs of the libraries targeting SAE1 (z-score 21.51) and UBA2 (encoding Sae2; z-score 17.99) and five out of six siRNAs targeting UBE2I (encoding Ubc9; z-score 16.59) induced a strong increase in transduction by the scAAV2-GFP vectors. Further, three other constitutive components of the SUMOylation pathway, SUMO2 (z-score 7.27), SUMO4 (z-score 3.38), and SENP1 (z-score 2.33) were among the putative host cell restriction factors. In addition, one confirmed and one putative SUMOylation E3 ligase, PIAS1 (z-score 2.43) and TRIM33 (z-score 7.72) were also among the hits. These data suggest an important role of SUMOylation in the control of AAV transduction.

**Table 1:** Components of the SUMOylation pathway identified as putative AAV host cell restriction factors. The screening of the genome-wide siRNA library revealed several factors of the SUMOylation pathway influencing AAV transduction. Protein and gene names are indicated. The screens were carried out in duplicates and each gene was targeted by three different siRNAs. The screen identified 740 putative host cell restriction factors, the table shows the ranking of the factors according to their z-score. Note: in the manuscript the protein identifiers are also used in reference to the corresponding gene.

Protein/gene	No. siRNAs	rank	z-score
Sae1/SAE1	6	1	21.51
Sae2/UBA2	6	2	17.99
Ubc9/UBE2I	5	3	16.59
Trim33/TRIM33	6	7	7.72
Sumo2/SUMO2	6	10	7.27
Sumo4/SUMO4	4	76	3.68
Pias1/PIAS1	3	294	2.43
Senp1/SENP1	6	330	2.33

All other hits are listed in S1 table. These have not been systematically validated yet. They fall into groups of proteins associated with cell surface, intracellular trafficking and the cell nucleus as outlined in the discussion.

### Knockdown of SUMOylation results in increased AAV transduction

**Fig 2:**
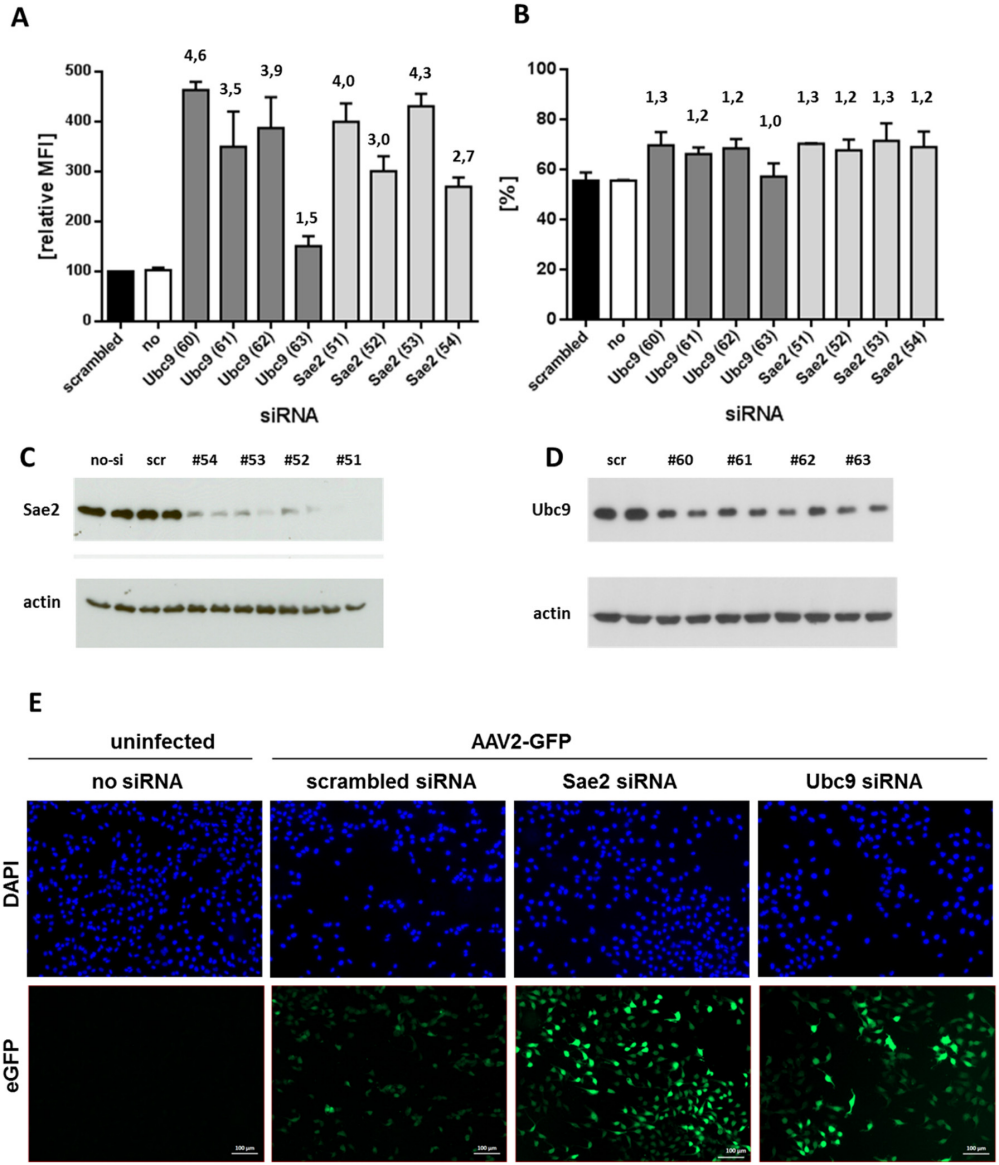
Knockdown of either the E1 or E2 enzymes of the SUMOylation pathway results in an increased infection efficiency of scAAV2-eGFP. HeLa cells were transfected with four different siRNAs targeting expression of Ubc9 and Sae2, respectively. Forty-eighth later, the cells were infected with scAAV2-eGFP at an MOI_GC_ of 10^3^. Twenty-four h after infection, half of the cells were proceeded for FACS-analysis, the other half analyzed by western blotting for protein levels after siRNA transfections. (a) Relative MFIs of three independent experiments are shown. The MFI was normalized by setting siRNA transfections with ‘AllStars negative control siRNA’ (Qiagen, indicated as ‘scrambled; scr.’) to 100. (b) The percentage of GFP-positive cells representing infected cells of three independent experiments are shown. (c,d) Western blot analysis showing a reduced steady-state level of Sae2 and Ubc9, respectively, after transfection of the four different siRNAs in comparison to cellular actin. The blot shows whole-cell extracts of two independent transfections for each siRNA. (e) Fluorescence of HeLa cells transduced with scAAV2-GFP vectors. Knockdown of Sae2 or Ubc9 was performed 48 h before transduction, respectively.

**Fig 3:**
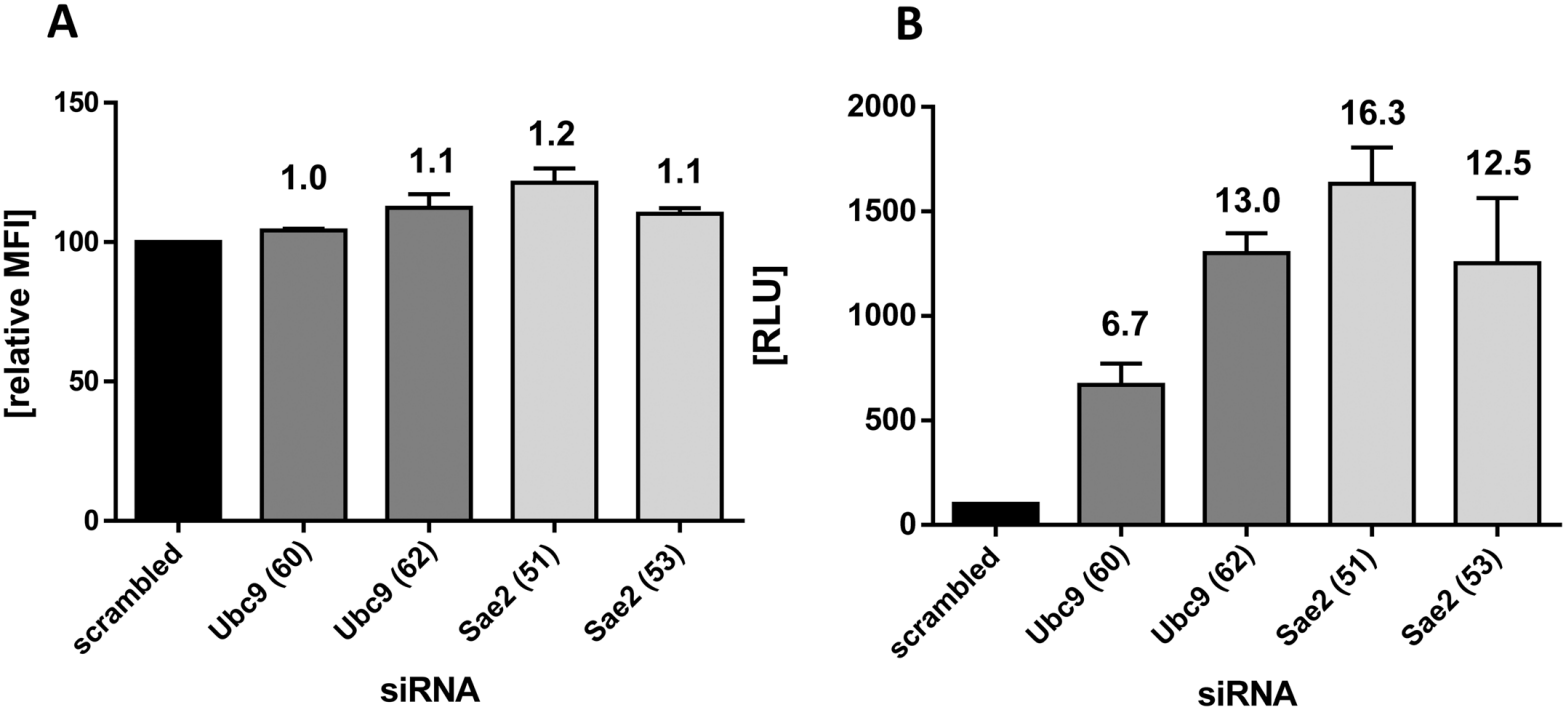
Knockdown of enzymes of the SUMOylation pathway does not alter expression of a stably integrated CMV-eGFP gene. A HeLa cell line stably expressing eGFP was transfected with four different siRNAs targeting Ubc9 and Sae2, respectively. Forty-eight h later, the cells were infected with scAAV2-renilla luciferase at an MOI_GC_ of 10^3^. Twenty-four h after infection, cells were harvested by trypsinization. One half of a well of a 6-well plate was proceeded for FACS-analysis (a), the other half was used to determine luciferase activity (b). Mean values and standard deviation of two independent experiments of mean fluorescent intensity (MFI) and relative light units (RLU), respectively, are shown. The values obtained after transfection with *AllStars negative control siRNA* (Qiagen; ‘scrambled’) were set to 100 in both cases.

The screening of the genome wide siRNA libraries identified members of the SUMOylation pathway as potential restriction factors for AAV genome delivery. Key players of the SUMOylation pathway are the two polypeptides Sae1 and Sae2 of the E1 enzyme and the E2 enzyme Ubc9 (Fig 1b). To validate the involvement of SUMOylation in the transduction of scAAV-GFP, knockdown of Ubc9 and Sae2 was performed with a set of four independent siRNAs from different suppliers. As shown in Fig 2a, three out of four siRNAs targeting Ubc9 and four out of four siRNAs targeting Sae2 led to an increased transduction by the scAAV2-GFP vector as determined by flow cytometry. All siRNAs reduced protein Ubc9 or Sae2 levels, respectively (Fig 2c and 2d). Notably, while the GFP signal was increased 3–4 fold, the number of GFP-positive cells remained basically unchanged (Fig 2b). This finding suggests that inhibition of SUMOylation augments the efficiency of transduction of those cells that were anyway transduced rather than it enables cell entry of AAV into cells which were not transduced in absence of SUMOylation knock down. The higher GFP-derived fluorescence intensity after knockdown of Ubc9 or Sae2 was also apparent when utilizing fluorescence microscopy (Fig 2e). To rule out an influence of SUMOylation pathway knockdown on factors such as GFP expression by altering the CMV promoter activity or the GFP steady state level, we analyzed HeLa cells stably transfected with a CMV-GFP expression construct for transduction with scAAV2 vector encoding renilla luciferase (Fig 3). Again, knockdown of Ubc9 or Sae2 increased AAV2 transduction determined by luciferase activity (Fig 3b) while expression of the endogenous GFP (Fig 3a) remained unaltered. In the following experiments we used either knockdown of Sae2 (key component representing the SUMOylation activation step) or Ubc9 (essential for SUMOylation conjugation step) to characterize the role of SUMOylation in AAV gene transduction, measured as transgene expression.

### SUMOylation affects single stranded and self-complementary AAV vector transduction but not AAV DNA transfection

**Fig 4:**
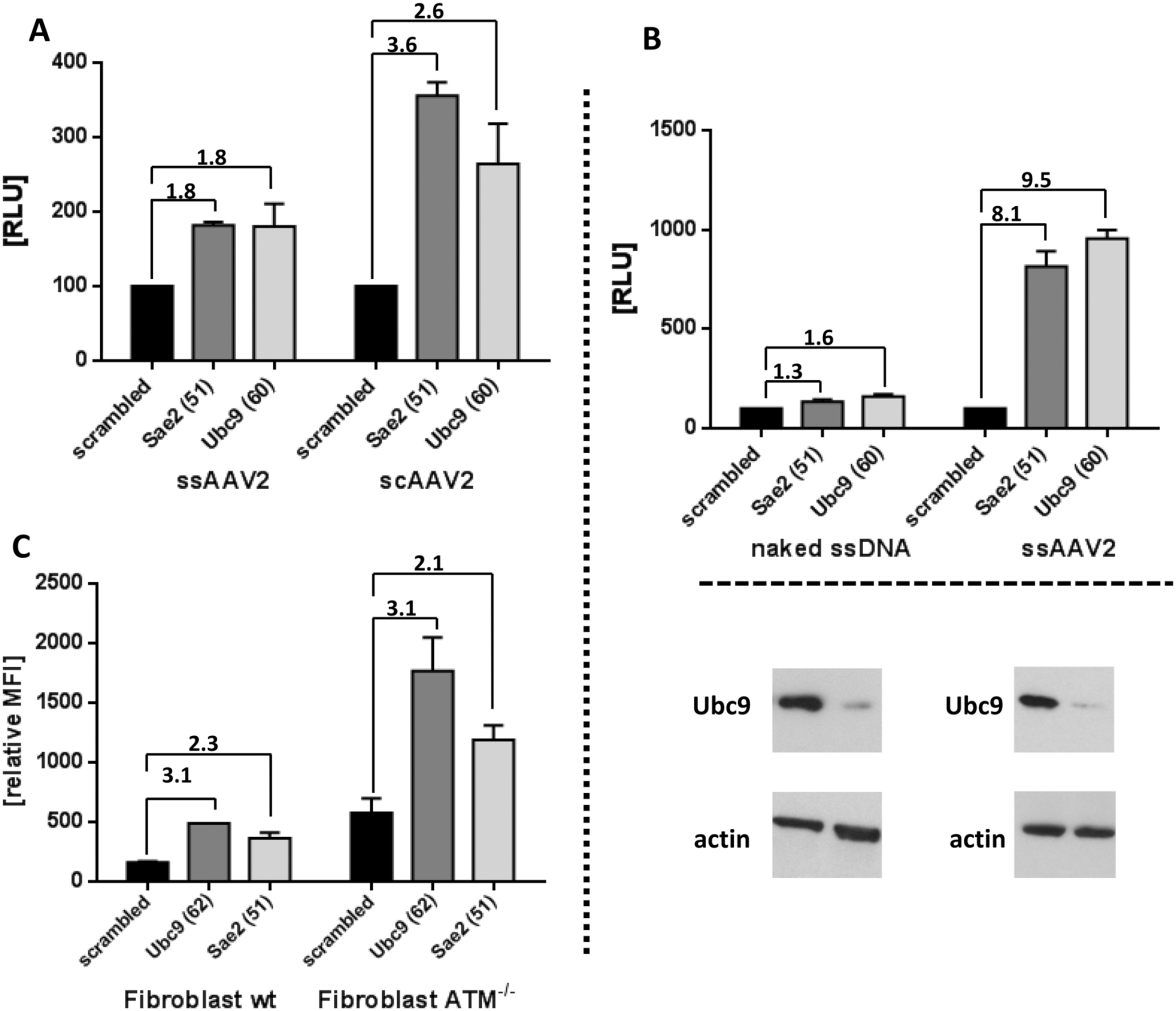
Knock down of Sae2 or Ubc9 enhances transduction by single strand and self-complementary vectors but not transfection of AAV single strand vector DNA. a: SUMOylation affects ssAAV and scAAV vectors. Cells were treated with siRNA targeting Sae2 or Ubc9 for 48 h followed by transduction with ssAAV2 or scAAV2. Shown are the mean values and standard deviations of the RLU of three independent experiments and normalized for treatment with scrambled siRNA. b: Genomes (naked ssDNA) were isolated from virus preparations of ssAAV2-firefly luciferase and transfected into cells treated either with siRNA targeting Sae2 or Ubc9 for 48 h. Twenty-four h later, luciferase activity was determined. The corresponding virus-preparation used for isolation of AAV-genomes was used in a parallel setting to infect siRNA-transfected cells (ssAAV2; MOI_GC_ of 10^3^) for 24 h. Knockdown of Ubc9 was confirmed by western blot analysis. Comparable cell equivalents were loaded, as demonstrated by the western blot probed with anti-actin antibody. Shown are the mean values and standard deviations of the RLU of three independent experiments normalized for treatment with scramble siRNA. c: The increase in transduction efficiency after knockdown of the SUMOylation pathway is independent of the ATM-kinase pathway. SV40-transformed human fibroblasts of a healthy donor (wt) and a patient with a homozygous mutation in the ATM-kinase (ATM- /-) were transfected with siRNA targeting Sae2 or Ubc9. Forty-eight h later, the cells were infected with scAAV2-eGFP at an MOI_GC_ of 10^3^. Twenty-four h later, cells were trypsinized and proceeded for FACS-analysis. Shown are the MFI of three independent experiments, in the case of wt cells transfected with Ubc9-siRNA, the mean of two experiments is shown. MFI values were normalized to values of wt fibroblasts treated with scrambled siRNA.

The initial genome wide siRNA screen and the validation experiments described above were performed with self-complementary (sc) AAV2 vectors carrying different reporter genes resulting in a 3–4 fold increase in reporter gene activity. To determine whether SUMOylation also affects ssAAV vectors we used ssAAV as well as scAAV encoding gaussia luciferase (ssAAV2-GL and scAAV2-GL) side by side in a transduction experiment (Fig 4a). Similar results were obtained for knockdown of Ubc9 and Sae2. Thus, the data show that SUMOylation affects ss and scAAV vectors. In subsequent experiments using ssAAV vectors with different reporter genes we observed up to 10 fold enhancement of transduction upon knockdown of SUMOylation therefore we do not think that SUMOylation has a stronger inhibitory effect on scAAV vector transduction (see Fig 4b and S1 Fig). Next, we asked whether SUMOylation also affects reporter gene expression when the vector genome is introduced by transfection rather than transduction. To answer this question we transfected HeLa cells with vector DNA isolated from gradient purified ssAAV2-firefly luciferase vectors. While again knockdown of Sae2 or Ubc9 lead to enhanced transduction with the corresponding AAV vectors (Fig 4b), it had no significant effect on expression of the reporter upon transfection of the purified vector DNA indicating that in fact SUMOylation targets a step during the entry of AAV before the DNA is released from the virions.

### Control of AAV transduction by SUMOylation is not caused by ATM-mediated DNA damage response

The DNA damage response (DDR) and SUMOylation are tightly linked processes (for review see [49]). Some authors claim that recombinant AAV induce a minor DDR via the Mre11/Rad51/Nbs1 (MRN) complex thereby potentially curbing the transduction efficiency [61, 62]. A central player in regulating the DDR after MRN induction is the cellular ATM kinase. In fact, human fibroblasts deficient for ATM are more aptly transduced by AAV vectors compared to wt fibroblasts. This finding is consistent with what has been previously described [31] (see Fig 4c). However, transduction of ATM ^-/-^ fibroblast was still further enhanced by knockdown of either Ubc9 or Sae2 (Fig 4c), indicating that ATM-related DDR, induced by the vector genome, is not mechanistically linked to the control of AAV transduction by SUMOylation.

### Inhibition of AAV transduction by SUMOylation is independent of MOI but depends on time point of knockdown

**Fig 5:**
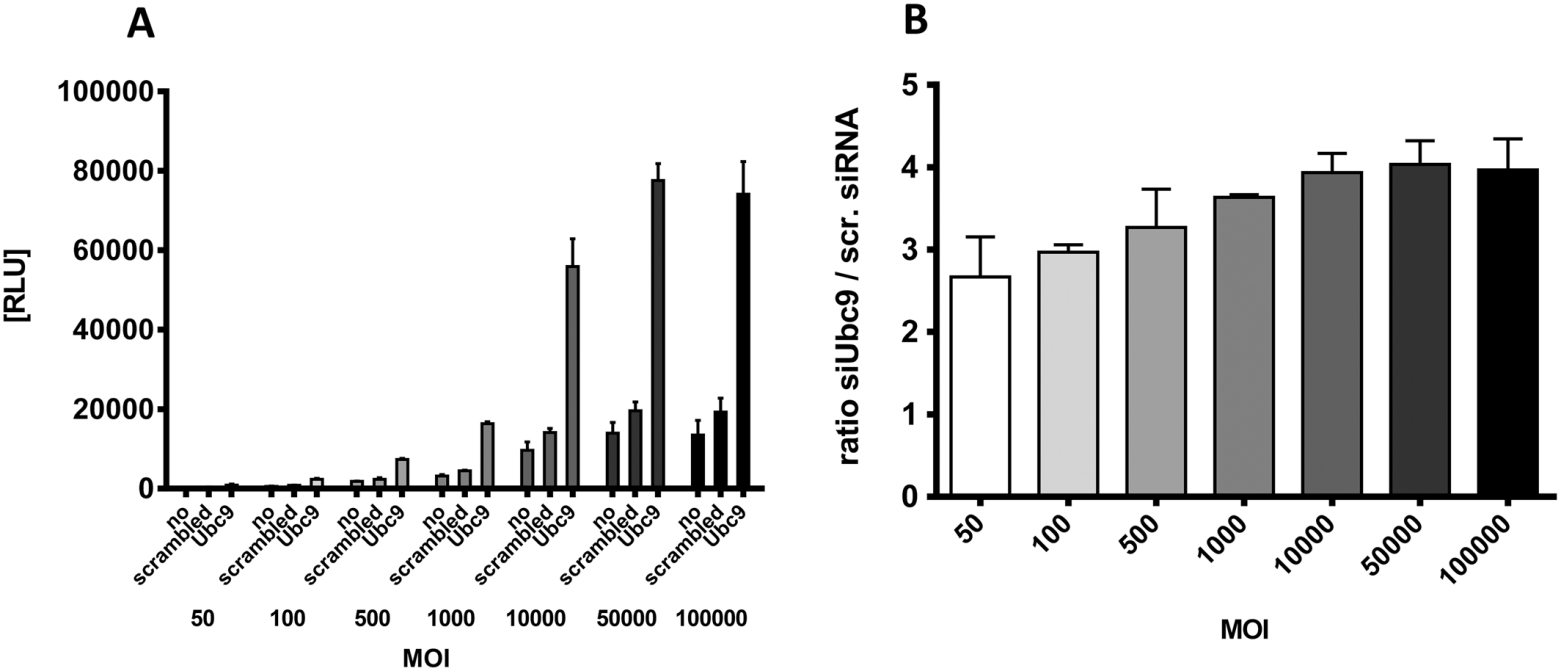
Effect of SUMOylation pathway on AAV transduction is independent of MOI. HeLa cells were transfected with different siRNAs and 48 h later transduced with ssAAV-firefly luciferase vectors at different MOIs as indicated. A: relative light units after 24 h incubation determined in three independent experiments, B: ratio of luciferase activity of cells treated with siRNA targeting Ubc9 and cells treated with ‘AllStars negative control siRNA’ (scrambled).

SUMOylation could directly or indirectly be required for functionality of a putative restriction factor interacting with AAV capsids in a stoichiometric fashion during gene transduction. Therefore, we asked whether increasing MOI would eventually titrate out such a factor resulting in a loss of the enhancement after SUMOylation knockdown. To answer this question, knockdown of Ubc9 was carried out followed by AAV transduction using MOIs ranging from 50–100,000 genome copies per cell. An enhancing effect of Ubc9 knockdown was observed for all MOIs, this effect even increased slightly with increasing MOIs from 2.8 fold enhancement (MOI 50) to about 3.8 fold (MOI 50,000), (Fig 5). The results indicate that the enhancement of AAV transduction upon knockdown of SUMOylation cannot be exhausted by high doses of vectors, at least for MOIs of up to 100,000.

**Fig 6:**
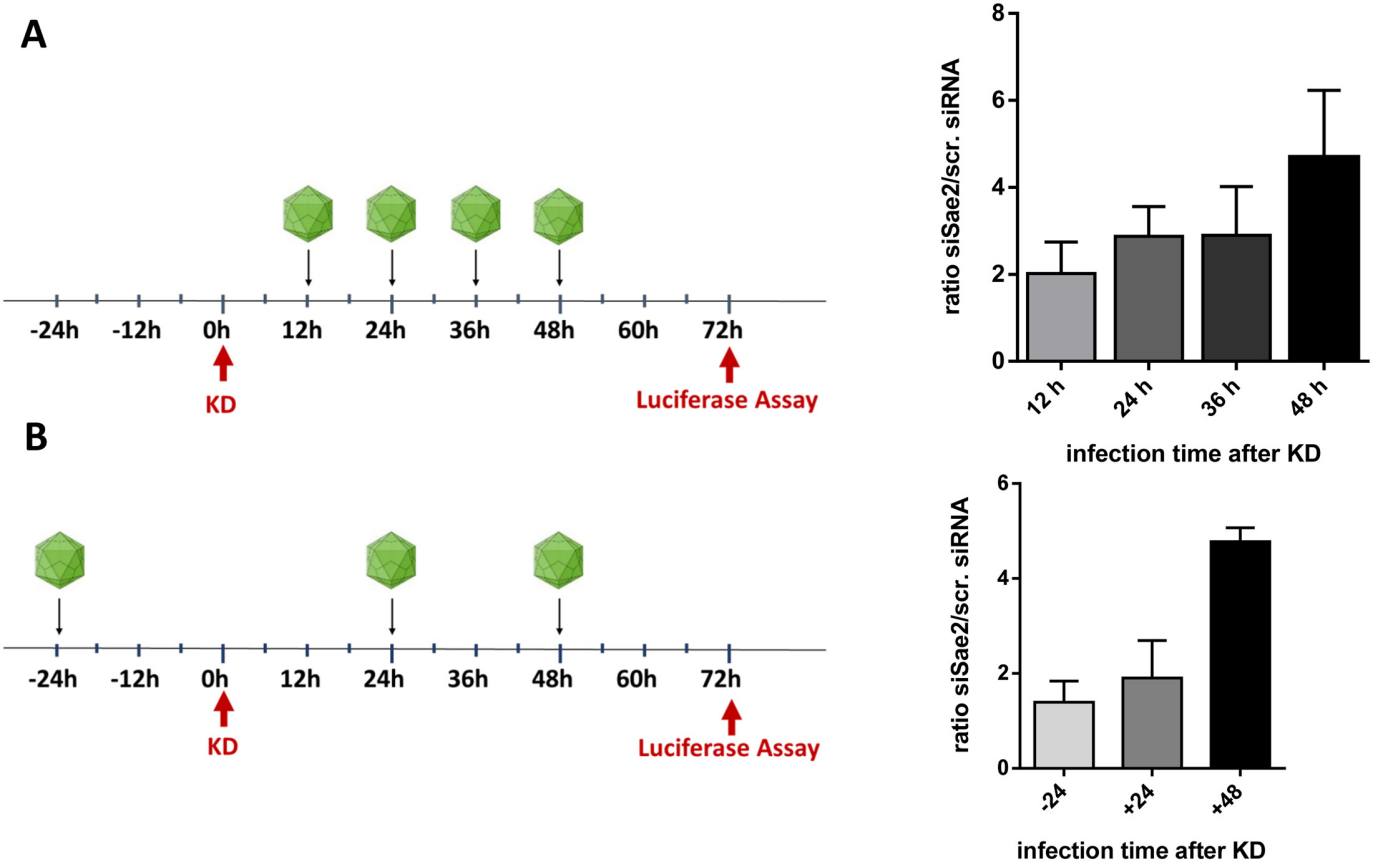
Influence of time point of Sae2 knockdown on AAV transduction. HeLa cells were infected with ssAAV2-firefly luciferase vectors at MOI_GC_ 1000 at different time points after (a) or before (b) knockdown (KD) of Sae2. The ratio of luciferase activity of cells transfected with siRNA targeting Sae2 and cells treated with ‘AllStars negative control siRNA’ (scrambled) are shown. Standard deviation of the mean of three independent experiments is indicated.

Next, we determined whether the time point of SUMOylation knockdown is critical for boosting AAV transduction. In the standard protocol we treated cells for 48 h with siRNA followed by AAV infection for subsequent 24 hours. Therefore, at the time point of infection, significant reduction in Ubc9 or Sae2 was already achieved. Reducing the time between knockdown and infection to 36, 24, or 12 h still resulted in enhanced transduction rates albeit to a lower extent compared to 48 h between knockdown and transduction (Fig 6a). We also investigated whether knockdown after infection would still have an effect on transduction efficiency (Fig 6b). For this, cells were infected with ssAAV-firefly luciferase vectors for 24 hours followed by 72 hours of knockdown. Here, no increase in transduction as a result of the knockdown was observed. The kinetic of Sae2 knock down is shown in S4 Fig.

### SUMOylation affects transduction irrespective of AAV serotype or capsid variation

**Fig 7:**
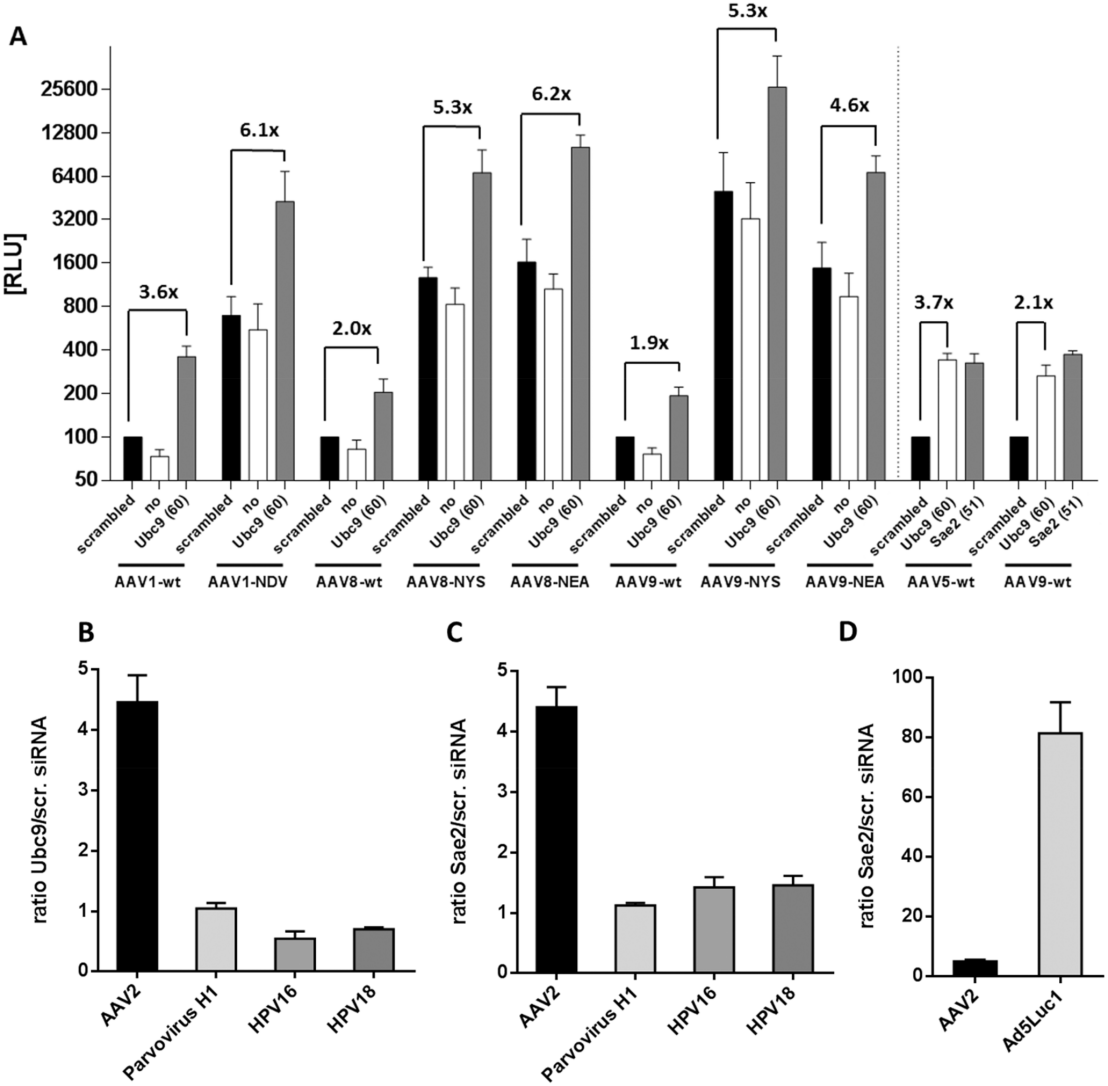
Knockdown of SUMOylation key enzymes increased transduction with different serotypes and capsid variants but not that of autonomous parvovirus H1 or human papillomavirus. A: HeLa cells were transfected with siRNAs targeting Ubc9 or Sae2. 46 h later, the cells were infected with ss-firefly luciferase vectors of AAV 1, 8, 9 and capsid variants thereof (left part) and scAAV5- or scAAV9-renilla luciferase (right part) at an MOI of 10^4^. The variants of AAV 1, 8, and 9 harbored heptamer insertions at the threefold spikes in position corresponding to amino acid 588 of AAV2. NYS: Peptide NYSRGVD; NEA: peptide NEAVRE. 25 h after infection, cells were lysed and analyzed for luciferase activity. The RLU values in the case of transfection of the control siRNA ‘AllStars negative control siRNA’ were set to 100. The mean values and standard deviation of three independent experiments are shown. B; C; D: HeLa cells were transfected with siRNA targeting Ubc9 (B) or Sae2 (C and D) 48 h before they were transduced with different recombinant vectors encoding luciferase reporters. Luciferase activity was determined 24 h post infection. The graphs show the ratio of luciferase activity of cells treated with siRNAs targeting Ubc9 or Sae2, respectively and cells treated with *AllStars negative control siRNA* (scrambled). Shown are the mean of three independent experiments with standard deviations.

To determine whether SUMOylation affects transduction by other AAV serotypes, we transduced HeLa cells after knockdown of Ubc9 with AAV1, AAV8 and AAV9 sc vectors carrying firefly or renilla luciferase, respectively. For all three serotypes and also for AAV5 (Fig 7a) we observed a 2–4 fold increased transduction upon Ubc9 or Sae2 knockdown indicating that the SUMOylation machinery impairs AAV-mediated gene transduction serotype-independent. In a concurrent project we identified that capsid modification of AAV by insertion of certain heptapeptide motifs leads to improved transduction abilities on various cells types, including HeLa cells (Sacher et al. in preparation). We do not know what mechanism lies behind the increased transduction efficiency of peptide-modified vectors but we hypothesize that these vectors may prefer entry pathways in which they are not facing certain host cell restriction factors. Thus, we were interested to determine whether the modified vectors still benefit from knockdown of SUMOylation. Surprisingly, we observed that the knockdown of Ubc9 was even more effective on enhancing transduction by capsid-modified versus wt vectors for all three AAV serotypes analyzed (Fig 7a). In combination, capsid modification plus knockdown of Ubc9 resulted in more than 40 fold enhanced transduction for AAV9 when compared to transduction of AAV9 wt on untreated cells.

### SUMOylation negatively affects gene transduction by AAV and adenovirus but not parvovirus H1 or human papillomavirus (HPV)

To determine whether enhanced transduction after knockdown of SUMOylation is AAV specific, we analyzed transduction of HeLa cells by HPV 16 and HPV 18 pseudovirions and the autonomous parvovirus H1 vector after knockdown of Ubc9 (Fig 7b) or Sae2. In parallel, cells were transduced with ssAAV2 vectors. All vectors encoded the gaussia princeps luciferase. While both, Ubc9 and Sae2 knockdown enhanced transduction by ssAAV-gaussia vector about four fold, there was no significant effect on HPV or H1 transduction. To rule out that the NS1 protein encoded by H1 is able to interfere with SUMOylation directly, we co-transduced HeLa cells with both H1 encoding ss-gaussia and ssAAV-firefly luciferase. Noteworthy, knockdown of Sae2 had a very strong effect on transduction by recombinant, replication competent adenovirus type 5, transduction was enhanced more than 70 fold compared to cells treated with control siRNA (Fig 7d).

## Discussion

In the past a lot of effort has been invested into identifying parameters which enable or limit AAV-mediated gene transfer. The development of siRNA technology to selectively reduce the level of single protein species introduced the possibility to systematically study the influence of individual proteins on the AAV-mediated gene transduction process.

Here, we describe the results of a screen of two siRNA libraries covering most parts of the human gene repertoire for identification of cellular factors influencing AAV-mediated gene transfer into HeLa cells. A similar approach has been performed by Wallen and colleagues [48]. However, their library encompassed only 5,520 genes and they analyzed the transfer of AAV2 vectors into human aortic endothelial cells. They conclude, however, that their top scoring putative AAV restriction factors are a result of off-target effects leading to a perturbation of the interferon response pathway. In particular interferon-induced protein 44-like, interferon-induced myxovirus resistance 1 and interferon-induced protein with tetratricopeptide repeats were found to be downregulated due to the off-target effects and this downregulation increases AAV2 transduction rates. In our screen, none of these three factors were identified as putative host cell restriction factor (HRF) which may be due to the use of different libraries or different target cells. Our results validate the gene targets of the top scoring HRF identified as actors in the AAV gene transduction process and point to a large number of additional putative host cell dependency factors (HDFs) and HRFs. Still, our screening approach has also a number of inherent limitations. Our strategy will fail if knockdown of the target genes is accompanied by cytotoxicity, if there is any redundancy in host cell factor function or if the target presents a factor with high protein stability. Further, the conclusions drawn from the screening data are limited to AAV2 transduction in a particular cell type (HeLa) and the use of scAAV vectors in the screen will exclude factors involved in single strand conversion. Lastly, it is not possible to control the efficiency of protein knock-down for each siRNA.

The threshold for z-scores defining HRFs and HDFs has been defined as 1.7 on statistical and not physiological grounds. With respect to the top 20 factors with a negative z-score (HDF), these can further be divided into nuclear factors involved in regulation of gene expression (e.g. HNRPK, LASS2, DDX54, MED11, SKIIP), DNA repair (UBE2V2), cell surface proteins (GABRA6), proteins involved in intracellular transport (RAB40B) and proteins involved in cytokine signaling (EBAF). Not all of the above mentioned factors were reproduced in the repeated screen and deserve a more critical evaluation by knockdown experiments using independent siRNAs. Prior to the siRNA screen we performed a siRNA knockdown analysis of AAV2 transduction targeting previously identified HDF such as dynamin2, small GTP binding protein Rac1, catepsin B, fibroblast growth factor receptor 1 and hepatocyte growth factor receptor as these factors have previously been described to play a role in AAV entry [63–67]. However, knockdown of these factors had no effect on AAV transduction in our assay nor were these factors identified as HDFs in the library screens. This coincides with the limitation that we could not confirm a functional knockdown in all cases e.g. due to the unavailability of suitable antibodies.

Similar to HDF, the host cell restriction factors with the highest scores are found at various locations in the cell. A large group of possible restriction factors consists of nuclear proteins involved in gene transcription (e.g. ATF7IP, CASP8AP2, NPAT), RNA processing (HNRPC, PABPC1, NUDT17) and chromatin assembly (CHAF1A). Also, lowering the level of two integral membrane proteins (SLCO1A2, NTRK1) enhanced AAV gene transduction. The latter is involved in receptor protein tyrosine signaling. Two proteins in the extracellular space (LEFTB, F8) might also negatively influence AAV gene transduction. Finally, a protein localized to the ER membrane (ERGIC3) had a z-score among the top 20 hits of restriction factors. Neither PML nor APOBEC3A were identified as a HRF in the screen, both of which were previously shown to act as HRFs for AAV transduction [32, 33]. In case of PML this result was expected as we used a scAAV2 vector in our screen and PML was reported to restrict ssAAV vectors only. However, two components of the PML oncogenic domain, death domain-associated protein (DAXX) and alpha-thalassemia retardation syndrome x-linked (ATRX) were listed with a high z-score, 5.39 and 3.38, respectively. Both proteins are able to form a complex in which ATRX has ATPase activity and DAXX recruits histone deacetylases [68]. The DAXX/ATRX complex represents an intrinsic immune mechanism acting as a viral defense against a large number of different viruses (for review see [69]). In return, other viruses have developed strategies to neutralize repression by DAXX/ATRX in order to overcome this barrier [70, 71]. We were able to validate DAXX/ATRX as putative AAV restriction factors independently of the siRNA screen (data now shown).

The most notable result of our siRNA screen was, however, that a number of factors of the SUMOylation pathway were found among the top-scoring HRFs. All three key enzymes of SUMOylation, the E1/E2 activation enzymes and the Ubc9 conjugating enzymes were top hits #1, 2, and 3, respectively. SUMOylation represents a post-translational modification similar to ubiquitination or neddylation (for review see [49]) involving four different steps (Fig 1b). As a result, protein stability, subcellular localization, protein-protein interactions can be altered. SUMOylation is a reversible effect, one out of several sentrin-specific proteases (SENP) can remove SUMO from target proteins. Thus, SUMOylation might be only a transient modification and it typically affects only a fraction of the total target proteins. SUMOylation is also tightly linked to protein ubiquitination which already has been shown to markedly influence AAV gene delivery [35].

SUMO2 and SUMO4 as well as the SUMO protease Senp1 were found among the hits, albeit with lower z-scores. Further, Trim33 and Pias1, two E3 ligases of the SUMOylation pathway, showed high positive z-scores in the screen. We could rule out that SUMOylation affects the promoters used in the vector constructs or the activity of the encoded reporter genes. The effect is independent of the AAV serotype but at the same time the parvovirus H1 vector as well as HPV pseudovirions are not affected, indicating that SUMOylation does not present a general anti-viral defense mechanism.

Which steps of AAV-mediated gene transduction could be affected by SUMOylation? In general, it can be assumed that restriction factors are involved in those viral infection steps that are rate limiting e.g. endosomal escape, nuclear translocation and ssDNA conversion. Cell binding and uptake are generally not considered to be rate-limiting for AAV. Since we observed no increase in the number of transduced cells upon SUMOylation knockdown we assume that SUMOylation does not restrict early events of cell entry of AAV. This has been confirmed by measuring binding and uptake of AAV vectors (S2 Fig). Surprisingly, we also observed no change of the intracellular pools of vector DNA in the cytoplasmic membrane or nuclear fraction upon SUMOylation knockdown (S3 Fig). Hence there is no detectable effect on endosomal escape or nuclear translocation using a cell fractionation technique. However, it has been shown that multiple alternative entry pathways for AAV within the same cell exist [39, 40] which might not become evident in cell fractionations. These entry pathways seem to support different transduction efficiencies. Following this train of thought, SUMOylation could induce preference of AAV for less efficient entry pathways resulting in poor gene transduction. The influence of SUMOylation on the intracellular localization of AAV vectors, however, deserves a more detailed microscopic analysis.

Further, SUMOylation likely does not target events related to AAV vector DNA as it affects single strand as well as self-complementary vectors and did not influence reporter gene expression after transfection of AAV DNA. SUMOylation and the DNA-damage response (DDR) are tightly linked. The AAV genomic DNA, in particular the ITRs are recognized by the MRN complex [72] leading to negatively regulated transduction by AAV [73]. This prompted the hypothesis that enhanced AAV transduction after knockdown of SUMOylation is mediated via the DDR. However, in cells lacking a major component of the DDR, the ATM kinase, knockdown of SUMOylation still enhanced AAV transduction. This is in line with the observation that autonomous parvovirus H1 gene transduction is not influenced by SUMOylation, as for H1 induction of a DDR has been reported previously [74]. Lastly, knockdown of SUMOylation had little or no effect on AAV when performed after transduction, again suggesting that SUMOylation affects events before release of the vector genome into the nucleus.

**Fig 8:**
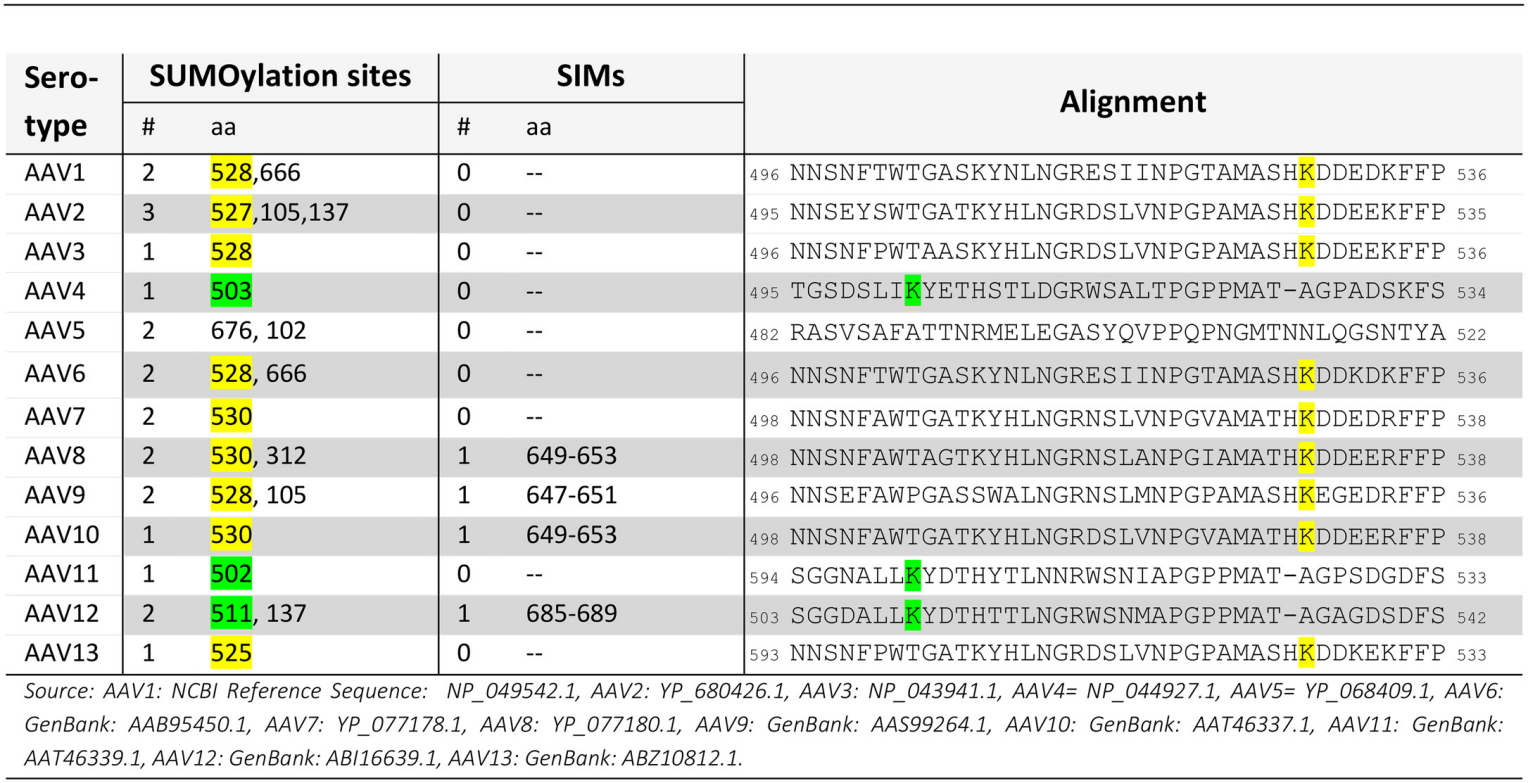
Prediction of putative SUMOylation sites in the AAV VP1 proteins. Potential SUMOylation sites and SUMO-interacting motifs (SIM) were predicted using GPS-SUMO [77]. Number and specific position of potential SUMOylation sites and SIMs are shown on the left hand side. AAV1, 2, 3, 6, 7, 8, 9, 10 and 13 harbor a potential lysine (K) which can serve as SUMOylation target (yellow). Also AAV4, 11 and 12 expose a potential SUMOylation target K at a position nearby (green).

Our experimental data argue for an involvement of the AAV capsid in restriction by SUMOylation although we cannot exclude additional contribution by other processes. SUMOylation acts on all AAV serotypes tested but showed stronger effect on AAV capsids harboring a heptapeptide insertion again supporting the assumption of an involvement of the capsid in SUMO-mediated restriction. One possibility is that the AAV capsid itself is a target of SUMOylation leading to altered intracellular processing during gene transfer. SUMOylation motifs follow often, but not always, the structure Ψ-K-X-D/E, in which Ψ is a hydrophobic amino acid. Capsids of AAV serotypes 1–3, 6–10, and 13 carry a putative SUMOylation motif corresponding to position aa 528 in AAV2 VP1 (Fig 8). In AAV4, 11, and 12, a motif can be found as well (corresponding to K502 of AAV4 VP1), however prediction of functional SUMOylation motifs is oftentimes error prone.

Alteration of SUMOylation motifs in the AAV capsid or identifying compounds specifically interfering with SUMOylation during the AAV entry could be envisioned as strategies to transiently boost AAV-mediated gene transduction *in vivo*. Inhibition of cellular genes *in vivo* has been demonstrated to be feasible using exosome mediated delivery of siRNAs [75] and could also be performed *ex vivo* on isolated blood cells, for example. Alternatively, we are preparing a small molecule screen, similar to the siRNA screen described here, with the aim of identifying compounds that lead to transient inhibition of host cell restriction factors.

## Supporting Information

S1 Table Z-scores of siRNA screen. (PDF)

S1 Fig Effect of inhibition of SUMOylation on ssAAV vectors. Cells were treated with siRNA targeting Sae2 or Ubc9 for 48 h followed by transduction with ssAAV2 encoding renilla luciferase. Shown are the mean values and standard deviations of the RLU of three independent experiments and normalized for treatment with scrambled siRNA. Data was normalized to scrambled siRNA, which was set to 100.
(TIF)

S2 Fig Enhanced AAV transduction by inhibition of SUMOylation is not due to increased binding or uptake. To measure the influence of inhibition of SUMOylation on binding or uptake of AAV cells were transfected with siRNAs targeting Sae2 for 48h. Cells were then incubated with AAV vectors at an MOI of 10^4^ for 1h at 4°C. After washing, cells were harvested directly (1h) or incubated for 5 h at 37°C. Cells were harvested by scraping or treatment with trypsin and proteinase K. AAV vector genomes were quantified by qPCR. The graph shows the mean of two independent experiments with triplicates each. The AAV vector DNA determined by qPCR was normalized to the corresponding siRNA control (scrambled). The Sae2 knockdown effect on AAV transduction in a parallel experiment 72 h after siRNA transfection was about 7 fold over scrambled siRNA.
(TIF)

S3 Fig Inhibition of SUMOylation does not lead to gross changes in subcellular localization of AAV vectors. Hela cells transfected with siRNAs for 48 h and then incubated with AAV2 vectors at a MOI of 10^4^ for 1h at 4°C. After washing, cells were incubated for 12 h and then fractionated followed by extraction of DNA as described in [78]. AAV2 genomes were quantified by qPCR. The graphs the fraction of AAV DNA found in the three subcellular fractions: membrane, cytosol, and nuclear.
(TIF)

S4 Fig Kinetics of Sae2 knockdown. Hela cells were transfected with siRNAs targeting Sae2 or with scrambled siRNA and harvested at time points indicated. Sae2 expression was determined by western blot and quantified by ImageJ. The graph shows relativee2 expression levels normalized for each time point to actin and to Sae2 signals of cells transfected with scr siRNA (set to 1).
(TIF)
