# Supplementary material for: The SUMOylation Pathway Restricts Gene Transduction by Adeno-Associated Viruses
Source: PLoS Pathog. 2015 Dec 1;11(12):e1005281. doi: 10.1371/journal.ppat.1005281 (PMC4666624; doi:10.1371/journal.ppat.1005281)
Supplement: S1 Table — (PDF) [file ppat.1005281.s001.pdf]

Gene Name	Score	p-value
RAB40B	-3,174900097	0,56069987
HNRPK	-3,082451356	0,013246102
SMS	-3,075939108	0,099149459
RP5-1022P6,2	-2,956273719	0,009870307
DDX54	-2,893604686	0,083456925
CCNB2	-2,857032166	0,185857312
EBAF	-2,729669648	0,383296284
COX8A	-2,673278325	0,032978223
MED11	-2,603116907	0,130149344
GABRA6	-2,594768853	0,037785508
C1QL2	-2,58346464	0,05647788
SKIIP	-2,543897924	0,144267705
RSHL1	-2,524168247	0,022289064
UBE2V2	-2,482310142	0,014066891
BAT2	-2,468057738	0,004236076
TESSP2	-2,46403316	0,004444089
LASS2	-2,461232259	0,028650918
TXNDC6	-2,459882966	0,02151054
MAP1LC3B	-2,456670514	0,0984986
TSPAN33	-2,446100141	0,829617729
SERF1A	-2,427522401	0,05992815
PPM1L	-2,401373746	0,058641027
LSM4	-2,40133039	0,023729813
UBE2G1	-2,398280189	0,024090417
PRPF4B	-2,391765707	0,003424347
COL4A2	-2,36165349	0,009189211
NOTO	-2,360208535	0,015422937
HLX1	-2,356708914	0,146473621
TXLNB	-2,344636946	0,671185705
LOC645197	-2,341136186	0,000624802
PEAR1	-2,262617962	0,065589066
CYP24A1	-2,226394291	0,245750841
DYNC2LI1	-2,224530419	0,040566436
LDHAL6B	-2,224518166	0,186867078
TRAPPC5	-2,219112027	0,007002905
MX1	-2,211007522	0,037750641
SPINT2	-2,198745979	0,19747741
OGFRL1	-2,177391535	0,061420295
FBN2	-2,167379893	0,030496401

LOC649935	-2,145607389	0,018234569
LOC645452	-2,143583752	0,851850313
NMRAL1	-2,131608164	0,164381058
LOC652800	-2,123893594	0,146946611
MCTP2	-2,11391324	0,05516641
PAFAH1B1	-2,10748299	0,129400554
ZNF585A	-2,107083855	0,003731682
BXDC2	-2,099938043	0,005201859
PRKDC	-2,097099088	0,077175177
LSM6	-2,095951552	0,012229749
PIK3C2A	-2,095604679	0,0723267
LOC646096	-2,08625818	0,005688004
hCG_2040210	-2,079078329	0,097850977
WTAP	-2,073846005	0,945705388
HGS	-2,05703355	0,124306767
CAST	-2,054588477	0,155460759
EFNA1	-2,051649096	0,000115228
C1orf158	-2,047120272	0,059078274
SAMM50	-2,040012446	0,228123583
FLJ40142	-2,038831121	0,115629195
MADD	-2,033468447	0,000399079
LYPLAL1	-2,029099121	0,208888848
HNRNPF	-2,022452043	0,216192143
ATP6V1G2	-2,02102252	0,083206656
SDAD1	-2,0187968	0,049000148
PLRG1	-2,010109146	0,106686942
ORMDL3	-2,005949611	0,086535706
HNRPH1	-1,991916354	0,752398243
PSMB8	-1,989636446	0,00300666
TXNDC8	-1,988724952	0,032094916
CTSL	-1,98582442	0,023303315
NDUFB7	-1,983203535	0,015968449
B3GALT1	-1,97629884	0,887347098
GABRR3	-1,975746074	0,061906944
ZFP95	-1,975522081	0,045580061
OR2L8	-1,971668899	0,002788414
RNF214	-1,969976898	0,000420559
C17orf90	-1,967151349	0,034245736
NUP50	-1,962528831	7,81E-05
LOC732028	-1,961092169	0,001564586
NPR3	-1,957042629	0,000715511
DIP13B	-1,956425462	0,868149748
ELOVL7	-1,955187751	0,002070708
GPR39	-1,948370455	0,53617002
LOC493869	-1,947700469	0,665813991

HORMAD2	-1,946079337	0,014754446
C20orf103	-1,944705344	0,100813499
TMEM34	-1,943757833	0,114051493
MRLC2	-1,941827352	0,201659201
NUDT5	-1,94082481	0,104717089
PHEX	-1,937617628	0,101915731
COL8A2	-1,937306966	0,30724981
EPHA8	-1,93583477	0,316507833
ADAMTS14	-1,935331343	0,809928004
ZFP91	-1,934142556	9,30E-05
SCAPER	-1,932816853	0,019617009
MED28	-1,932744015	0,018619617
PRIM2A	-1,9303939	0,022209312
LSM8	-1,927112485	0,023560456
APBB1	-1,900826629	0,002848294
UBE1L	-1,898110237	0,210597427
FOXB1	-1,887239804	0,130740552
SLC35A4	-1,886986384	0,12063623
LOC645188	-1,881229894	0,02935252
NKAP	-1,87571592	0,000111922
RHOB	-1,867402163	0,04710218
CIAO1	-1,865500802	0,30421041
DHCR24	-1,864587899	0,269006145
PHB	-1,86381374	0,045358573
C16orf74	-1,858918604	0,050671662
LOC652048	-1,856237233	0,240228311
PIK4CB	-1,85572391	0,006173063
COX7B2	-1,851456189	0,009321516
SNRPB	-1,849250975	0,071394924
APG4B	-1,846480646	0,650151595
KCNK16	-1,838304025	0,045257121
C20orf185	-1,832848453	0,011476719
SLC27A4	-1,829394736	0,055581417
PDZD11	-1,827655266	0,078370651
TMEM128	-1,824378393	0,050398625
DUXA	-1,82139577	0,004954884
ST7L	-1,820052774	0,014733503
SCFD1	-1,817986067	0,000382099
C6orf154	-1,810873991	0,002184532
MAGEA6	-1,810727482	0,00148064
FLJ31579	-1,810367743	0,215393458
ZNF600	-1,806128819	0,294122654
FAT3	-1,805327869	0,064540674
CUL1	-1,802558641	0,064437333
PYGM	-1,799833667	0,925479225

LOC730497	-1,79718833	0,00069452
PRR18	-1,796806699	4,49E-06
DV-E	-1,790849271	7,55E-85
PLXNB2	-1,789214963	0,087118087
LOC731805	-1,789158613	0,22350158
SOLH	-1,788698327	0,121608672
ZNF179	-1,788561142	0,042292076
C1QTNF5	-1,788258281	0,031142356
NAGLU	-1,78261459	0,016094795
FKSG2	-1,777811331	0,026102561
OR7C1	-1,773194522	0,00091236
SOCS2	-1,768988607	0,196126619
RNF167	-1,766785473	0,676555965
ZNF430	-1,766452226	0,038577565
PSORS1C2	-1,760016176	0,059712854
TRIM14	-1,758892102	0,115414235
LOC729705	-1,757816537	0,001115295
C15orf43	-1,756777911	0,001389805
BCHE	-1,753210024	0,008657219
FAM19A5	-1,752470749	0,001547161
LOC392529	-1,750381287	0,108271544
STK11	-1,749551128	0,049360927
DHX32	-1,749451649	0,067495866
DLGAP4	-1,747693017	0,015940879
PRPF40A	-1,746499554	0,042264417
APLP2	-1,74497117	0,035921776
NDUFV3	-1,742597171	0,00495232
OR10A5	-1,741105104	0,00317757
SERPINB2	-1,741017545	0,000198099
LRRC39	-1,740533295	0,060975191
OR2Z1	-1,738892995	0,628013342
SKD3	-1,736910816	0,019209149
ASNS	-1,73519905	0,924822011
Dlc2	-1,733943032	0,060125752
LOC285588	-1,732586537	0,279449258
YY2	-1,732281709	0,069545902
FTSJ3	-1,730505583	0,102222357
RHEBL1	-1,728912955	0,44279482
MRCL3	-1,725680688	0,025095767
PPEF1	-1,725544589	0,000238124
IPLA2(GAMMA)	-1,722661508	0,008569267
VPS52	-1,721698823	0,003020578
LOC645504	-1,716006704	0,089010823
RSAD2	-1,714607438	0,023132653
GPR55	-1,714358895	0,008489786

OIP5	-1,709682221	0,185805569
C12orf24	-1,709292902	0,11132894
MT4	-1,708588336	0,008057158
PYC1	-1,704055332	0,022283455
ABCC5	-1,700156383	0,042095024
PKM2	-1,700109286	0,133107628
ROPN1B	-1,698902848	0,110280802
SRRM2	-1,698553876	0,011378485
SIAT1	-1,696148215	0,22561641
CUGBP2	-1,694389147	0,024781688
KRTAP5-4	-1,691035068	0,698908557
SBNO2	-1,690695607	0,00874033
PBXIP1	-1,689819627	0,04126831
CC2D2B	-1,689321531	0,001045477
SLC2A13	-1,689250694	0,02939452
DMRTA1	-1,687125566	0,30365336
HEY1	-1,686892118	0,076930678
UFSP2	-1,686504181	0,047258317
KIAA0415	-1,685469192	0,717665777
SUDS3	-1,679494751	0,000134317
RHCE	-1,6791322	0,297735044
FAM111B	-1,678332257	0,00831247
LOC643085	-1,674801943	0,463500523
hCG_26523	-1,674686853	0,175962066
MPG	-1,674334126	0,600480336
ZNF385C	-1,671571015	0,011945176
DEPDC6	-1,671114674	0,066042214
MPST	-1,666893238	0,117603751
ARID4B	-1,666507714	0,051820755
SLC26A2	-1,665689572	3,89E-05
TNFRSF17	-1,665206822	0,022100535
LOC285141	-1,660663943	0,043020078
USP30	-1,659246594	0,381824664
LOC731680	-1,656778615	0,147252252
C1orf109	-1,65616014	0,263971711
WDR86	-1,652194696	0,107792429
UBQLN4	-1,651372452	0,192528832
GSTA4	-1,649806394	0,047503995
ATPAF2	-1,649056129	0,36384
LOC728009	-1,647844545	0,37813085
EHMT1	-1,647082543	0,084480093
PIK3AP1	-1,646020268	0,003837879
LOC728382	-1,645808946	0,043943551
LOC728029	-1,64196891	0,021130008
PICALM	-1,641934256	0,05073066

C11orf48	-1,640875094	0,018000617
DYX1C1	-1,639058931	0,003767017
LOC644990	-1,638714558	0,066109976
ROM1	-1,637770478	0,022688768
LOC729990	-1,634038931	0,288945339
TIMM23	-1,630889531	0,001334962
ETF1	-1,628696124	0,058883698
SERPINB11	-1,627580275	0,00083205
CCDC96	-1,623922277	0,040442066
TBDN100	-1,623534548	0,015443374
TM9SF2	-1,622004022	0,4994198
CLCA4	-1,621922155	0,057924968
INTS8	-1,621404252	0,004720932
TMEM131	-1,619853554	0,009202607
LOC653935	-1,61865129	0,006681166
LOC645355	-1,617262367	0,014256955
C6orf130	-1,61419096	0,099450656
RGS7	-1,612775932	0,386774128
MED23	-1,612593115	0,020754714
SLC39A10	-1,611614713	0,027627975
CRYM	-1,611304455	0,036149279
PHF5A	-1,610605886	2,12E-69
KIAA0073	-1,604619946	0,02954728
ADAM12	-1,603297523	0,027174045
FGF2	-1,602927246	1,19E-107
DSG1	-1,602611206	2,58E-05
DDX53	-1,601026015	0,067780801
LOC388948	-1,59653705	0,00049615
KIAA1173	-1,596209586	0,029517868
NAV2	-1,594099539	0,001358536
CHRM1	-1,59405555	0,321744112
SNRPC	-1,59380358	0,077867873
C3orf62	-1,591911406	0,341333288
DCHS2	-1,591615238	0,259284577
hCG_2045830	-1,591349303	0,045658671
C1orf106	-1,590143175	0,018605559
SRPX	-1,588634192	0,032677801
PDZD3	-1,586711235	0,002773419
LOC731851	-1,586183739	0,098143603
PI15	-1,584755289	0,100801388
BBS9	-1,582799007	0,268495494
PAGE2	-1,580670129	0,071088023
LOC387761	-1,57972098	0,03611207
OAZ3	-1,579546305	0,400808027
MGC15937	-1,577191306	0,627745899

LOC729427	-1,575033744	0,001031263
FIBIN	-1,57419075	0,000118536
LOC728684	-1,573933573	0,004400819
DNAJC10	-1,573618734	0,036088503
ZC3H13	-1,573243914	0,153053311
TGOLN2	-1,570752562	0,289045732
COVA1	-1,569921131	0,938908064
TMEM110	-1,569066472	0,041201278
SEMA3E	-1,565492063	0,893338354
HDX	-1,564683967	0,088272953
RBBP6	-1,563020248	0,028642176
LOC645464	-1,561144555	0,389517284
FLJ40292	-1,560738841	0,269570737
RGNEF	-1,560443419	3,41E-05
LOC283585	-1,559747535	0,004649921
PVRL2	-1,558795213	0,05639559
HRC	-1,55827418	0,027537287
ZNF571	-1,554935851	0,027271283
LOC653513	-1,550361992	0,280880045
SPRR1A	-1,54943181	0,001038794
PLAA	-1,549152722	0,047980707
C20orf36	-1,548544995	0,079855017
ZNF569	-1,548310544	0,120932161
PRB3	-1,54722883	0,008961103
LOC646976	-1,547029787	0,029152756
ATP5S	-1,543307306	0,073888485
EIF4A1	-1,542519899	0,056752598
POLG	-1,540260031	0,137855583
AMT	-1,537620355	0,041593761
SETBP1	-1,537159671	0,082698597
PCSK4	-1,536371438	0,241854086
LOC727915	-1,534858093	0,022959269
PLCZ1	-1,534110702	0,034769061
NUP62CL	-1,532731439	0,25766119
WDR61	-1,531734887	0,736444025
LOC390876	-1,53149056	0,047553964
RPL27	-1,527340432	0,049108884
LOC400120	-1,525706611	0,088454317
LSM3	-1,523360816	0,163844872
ZNF132	-1,520023757	0,055695081
CYP2U1	-1,519337858	0,056285116
WFIKKN2	-1,51800178	0,086767306
GFRA3	-1,517786292	0,042788513
LOC648152	-1,516075624	0,038487923
TCTE1	-1,512556535	0,258710345

C20orf132	-1,512180087	0,043995145
DUSP15	-1,511942356	0,002705694
IPP	-1,51056995	0,042214123
ROGDI	-1,509882613	0,07804156
GPM6A	-1,509582609	0,031443198
IL1F6	-1,509353152	0,002462782
LOC401957	-1,508170695	0,179042269
POLR3F	-1,50643975	0,166124311
ANKRD26	-1,506053789	0,001762616
EIF2C1	-1,505881877	0,170601373
CPEB3	-1,505077331	0,001544973
DKC1	-1,50132973	0,238516922
PTGER1	-1,500686038	0,090266386
FAM179B	-1,500406031	0,129876822
CYP3A5	-1,500374537	0,058691316
FBXO9	-1,500373697	0,064943381
C8orf55	-1,499520277	0,674088428
SLC7A3	-1,499231318	0,054477219
LOC732041	-1,498531368	0,234640637
LOC729544	-1,494728399	0,789276836
RMI1	-1,494375836	0,001316961
MSR1	-1,493585887	0,011255807
DCLRE1C	-1,492584902	0,047692323
LIFR	-1,492438461	0,112744171
LOC727843	-1,491687164	0,03335661
HPS3	-1,490006026	0,031213992
CX36	-1,489800883	0,228826937
SLC35D2	-1,48918629	0,103050715
SLC25A26	-1,487321317	0,08333868
LOC729882	-1,484613136	0,059298302
KRTAP5-9	-1,484348127	0,799354329
RNF168	-1,482855167	0,020075859
FAM46A	-1,482427516	0,022303365
CXCL10	-1,482179695	0,051073948
WDR51B	-1,481387784	0,045574429
LOC643464	-1,48077925	0,066482227
PIK3CG	-1,48044732	0,098304834
KIAA1115	-1,478781444	0,392081423
C20orf135	-1,478518653	0,418711684
HDHD1A	-1,476113627	0,015830549
C6orf11	-1,474842389	0,007944595
NCAPH2	-1,474639099	0,866511252
PXT1	-1,474125054	0,004428185
FLJ41200	-1,4738806	0,045620639
ARMET	-1,468757441	0,00677947

GUCY1B3	-1,46730171	0,00028579
THRB	-1,466855422	0,03429742
LOC119358	-1,466792873	0,2089611
ENAH	-1,466110643	0,089581188
C10orf68	-1,466006041	0,17577339
CRTC3	-1,460843788	0,230420198
CCNJL	-1,460603016	0,020458831
G3BP	-1,460211235	0,010093513
NEURL4	-1,459951018	0,091959927
GRM7	-1,458018533	0,02110913
WDR5	-1,45775278	0,022394823
MED9	-1,457261112	0,278936578
C9orf23	-1,455872303	0,077925709
NUDT22	-1,455410223	0,07470935
VPS54	-1,455346941	0,060195528
SNRPA	-1,454935479	0,058012987
PRSS12	-1,45489056	0,299810242
ADARB1	-1,45395783	0,016768712
PLA2G4A	-1,453711535	0,052169641
RORC	-1,452470334	0,013951012
LOC728970	-1,448477179	0,009007862
LOC646388	-1,44749187	0,001017905
LOC645565	-1,446757506	0,011641502
ROBO1	-1,446099866	0,15364453
THRAP6	-1,445659463	0,191709432
TRIM3	-1,444526786	0,036481892
MRPS10	-1,444336759	0,002299884
KCNA1	-1,443423141	0,024150913
SMPDL3B	-1,443011547	0,018677655
PP	-1,441768098	0,068914836
RENBP	-1,438284804	0,605881779
KIAA1161	-1,43715553	0,519789719
LOC729918	-1,436635991	0,024364338
TOMM7	-1,435342901	0,165044197
FAP	-1,434413898	0,061484999
LOC730122	-1,433522926	0,046103384
STX8	-1,433465364	0,020897963
LOC728671	-1,432355589	0,011894621
LOC652707	-1,431832092	4,96E-05
CDY2	-1,431796567	0,008295229
FMR1	-1,430900496	0,057730727
LOC650770	-1,428797736	0,050621946
ATP5O	-1,427909071	0,242253056
FAM19A2	-1,427536679	0,965274449
LOC731929	-1,426388168	0,177637898

ANKRD56	-1,424785022	0,095042018
LOC731115	-1,424533328	0,497305848
ITLN1	-1,424035461	0,749072179
TRAM1	-1,423005413	0,007347583
KIAA1432	-1,422843203	0,005304997
PNLIP	-1,422301468	0,019697817
MCCC1	-1,422180537	0,212639026
CTNS	-1,421523704	0,011275441
SLC36A4	-1,421033439	0,441019426
CNP	-1,420802052	0,033220313
C10orf52	-1,419847825	0,108643618
LRWD1	-1,413582126	0,001409426
LINCR	-1,412799768	0,064551055
LOC644638	-1,411766076	0,093351542
COG6	-1,410891321	0,008580721
CYorf15B	-1,408872654	0,102726144
ERF	-1,408350252	0,07270585
LYRM1	-1,407516499	0,540783324
TMEM161A	-1,406947621	0,000836041
ENPP1	-1,406573599	0,485470086
GALNT4	-1,405465261	0,384340633
FLJ30375	-1,4042869	0,339026694
KIAA0528	-1,403973907	0,056704825
EVL	-1,403857915	0,004019232
PRPF18	-1,403674195	0,038125449
GZMB	-1,402951778	0,068859603
IL17D	-1,40211014	0,13480414
ARMCX3	-1,401495221	0,080786728
CDH18	-1,399616484	0,039504023
KRTAP6-2	-1,398866835	0,063459993
KLHL18	-1,398430673	0,027162927
FAM125A	-1,396628658	0,085156332
PCBP1	-1,394655275	0,081950269
FXYD7	-1,394104696	0,022825199
KLHL23	-1,393811719	0,000806816
TEC	-1,393074153	5,21E-130
CRKL	-1,390084489	0,025033692
ESPL1	-1,389310103	0,051739579
KIAA1383	-1,388838487	0,176443282
VAT1	-1,388760975	0,042285422
MED19	-1,388314244	1,63E-05
CATSPER4	-1,38619058	0,259655874
TEX14	-1,382343477	0,010505966
UQCRC1	-1,382343075	0,039478011
CRISPLD1	-1,381481634	0,051702635

C16orf62	-1,381127358	0,011018706
CSRP1	-1,38047032	0,140011913
CD36	-1,380094209	0,927244437
IMAGE:4907098	-1,379804683	0,247334373
GPR17	-1,379646904	0,043448703
RIMKLB	-1,377792724	0,037541388
OR4A47	-1,376775229	0,073932575
NDUFA6	-1,375896545	0,036441137
PLP1	-1,375695835	0,335475282
ALG14	-1,375114206	0,150430205
NBLA00301	-1,374927608	0,001051553
DHX9	-1,373828009	0,97840054
THY1	-1,373264215	0,144952421
LOC730044	-1,373145635	0,674954553
LOC728460	-1,372028218	0,034421151
MCM3AP	-1,371495255	0,058816599
CNTN5	-1,370064988	0,190913438
ARC92	-1,369868245	0,168604969
CAPS	-1,369230007	0,041159962
LOC731992	-1,369182366	0,457697256
PDCD2	-1,36768995	0,022164099
SMARCE1	-1,367676159	0,007139175
DMWD	-1,365248518	0,211643068
TRIM47	-1,365071879	0,025761115
LDHC	-1,365061072	0,51430195
PSMB6	-1,362864771	0,027515512
FLJ37644	-1,360830413	0,014066042
OR10Z1	-1,359578433	0,103677945
SLC20A1	-1,358301296	0,232098443
SNRPD3	-1,358113992	0,23486521
BTN3A2	-1,357116947	0,242517258
TRIM6	-1,355570956	0,204930084
SPA17	-1,355526543	0,039041224
PIGO	-1,355389604	0,045825122
SLC25A21	-1,355102278	0,118000952
ARL16	-1,354704353	0,143840706
TITF1	-1,354282964	0,220792795
IFT74	-1,352737251	0,1403354
DPP4	-1,35217072	0,768680641
USP49	-1,35137282	0,060748492
GNG3	-1,351274494	0,04034229
HTR3C	-1,350710788	0,554214206
LOC728809	-1,350192853	0,003026195
DHX8	-1,349018803	0,001252633
LOC731771	-1,348979426	0,238617596

KRTHB5	-1,347129634	8,39E-05
GPR89	-1,347011508	0,062479885
NDRG4	-1,346059054	0,046719092
AGPAT1	-1,345617403	0,378045365
GLB1L3	-1,34545207	0,069447149
FLJ26056	-1,345280933	0,005998374
IFITM1	-1,344339243	0,976462859
LOC731938	-1,343751271	0,024834936
LOC440335	-1,34284717	0,086450866
RBMS1	-1,340062518	0,013544288
USP15	-1,338210989	0,080643086
NCK2	-1,336282172	0,006684579
TRPV4	-1,336109503	0,008732628
MUC20	-1,336040965	0,001495506
SCPEP1	-1,33502282	0,086841084
ZIC2	-1,334514298	0,034480138
CLMN	-1,33376076	0,14599294
POU3F1	-1,333126759	0,022316598
KCNRG	-1,332804935	0,022708765
RYBP	-1,332742498	0,029666245
DCT	-1,331856472	0,046788197
BEX1	-1,330160658	0,01254459
LOC645781	-1,330152615	0,000296009
OR10A7	-1,330109053	0,025414263
KIAA0913	-1,328927747	0,804217542
ANKRD50	-1,326161644	0,185538257
SUSD5	-1,325616296	0,937066981
LOC732307	-1,324511818	0,482980585
HD	-1,323758252	0,101589508
TTC37	-1,322763577	0,052749366
LOC730628	-1,321411045	0,039826865
SEMA6B	-1,320755377	0,413719979
LOC728273	-1,320222527	4,01E-05
PPA2	-1,319974365	0,136901519
APOBEC2	-1,319718196	0,04527614
OR51A7	-1,318797545	0,038541418
LOC152663	-1,318643651	0,289670739
RBKS	-1,318287334	0,123043529
CSDE1	-1,317650956	0,024359533
ZNF706	-1,316647089	0,228958071
OR1D2	-1,315844231	0,207271845
PFKFB3	-1,315665108	0,08786028
XPO6	-1,315290211	0,004357982
LOC200008	-1,313749873	0,053169196
SUSD4	-1,313329732	0,004263609

RPA1	-1,313089412	0,338539378
SPHAR	-1,312419633	0,034243673
ZNF148	-1,310759536	0,003422065
TM7SF2	-1,3106197	0,042690232
C1orf186	-1,310614266	0,189402608
LSM7	-1,310059906	0,193128359
C8orf37	-1,310029894	0,001909007
C10orf53	-1,309626231	0,002514563
FAM122C	-1,309524895	0,095896227
WDR7	-1,309464116	0,892880025
KLHL15	-1,307912218	0,329292213
NAV1	-1,307682367	0,13650021
ATG2A	-1,307635266	0,163480849
GSN	-1,307250281	0,036615414
RUNDC1	-1,305868956	0,104644755
PXMP3	-1,303911555	0,468817389
LOC727965	-1,303635288	0,005913333
GTF3C1	-1,30258997	0,005686485
FAM20B	-1,302345864	0,276889454
INTS5	-1,302006605	0,009351663
HSD17B12	-1,30129623	0,033433003
SLC25A5	-1,299603231	0,01868183
WDR62	-1,298926197	0,377776019
OR5AN1	-1,297985901	0,707584772
RDS	-1,29597301	0,491092
PPP1R15B	-1,295917664	0,322666965
KRT74	-1,295838153	0,807051811
CPAMD8	-1,295743639	0,040159987
C19orf67	-1,295492922	0,504510398
PMPCB	-1,293905935	0,020293186
KCNA7	-1,292415016	0,044434179
ZNF479	-1,29235088	0,251409461
RDM1	-1,292172831	0,06754071
THOP1	-1,291319398	0,006190521
ETS1	-1,290270588	0,024568295
ROBO3	-1,289854303	0,019691312
ANXA5	-1,289569832	0,645747625
DPEP1	-1,289167349	0,033837827
AMMECR1L	-1,288933167	0,785648196
EGFLAM	-1,28813862	0,200031813
CHST13	-1,287062555	0,213317428
CDC40	-1,286401388	0,015291769
CDH15	-1,285975908	0,993408199
SLC35F3	-1,285971467	0,037388834
GPD2	-1,285510046	0,252396296

LOC729509	-1,285391861	0,285199988
DKFZp727A071	-1,285260092	0,081439003
KIF25	-1,283533069	0,007769764
SHOX2	-1,283171523	0,035004632
PPAT	-1,282300191	0,299865142
C14orf130	-1,282163287	0,004409095
FLJ30277	-1,281721559	3,49E-05
SLC34A2	-1,281557994	0,013672526
LRRC18	-1,281223405	0,001744424
EXT2	-1,280472024	0,218973421
FEM1A	-1,280271748	0,071778764
SPIB	-1,27998656	0,647607139
LOC729757	-1,279686444	0,128331566
ACTR8	-1,279557942	0,001009695
CCR4	-1,279076709	0,021268282
KIAA1715	-1,277752196	0,039275026
LOC730966	-1,277557799	0,031773534
RABEPK	-1,277482639	0,016750322
STRAP	-1,276336448	0,026239867
PRH2	-1,275611978	0,687561901
CYP3A4	-1,275180829	0,074751107
CAV2	-1,274945394	0,033543525
SIPA1	-1,274798056	0,129613259
LOC728018	-1,273673917	0,884310501
JMJD4	-1,273206879	0,008084546
LOC729804	-1,272809176	0,002236408
HMCN2	-1,27174295	0,044021859
RIPK4	-1,271633998	0,657855903
LOC728218	-1,270396981	0,111574061
FLJ41484	-1,269412426	0,370163331
LOC652516	-1,268469879	0,003107746
C6orf62	-1,268371652	0,767980396
GUCA2A	-1,268265406	0,003588614
IL28A	-1,268128465	0,006264036
C9orf103	-1,267756365	0,002467584
OTP	-1,267545364	0,12325489
NAGS	-1,267507043	0,006931397
USP21	-1,266481989	0,180409696
CRTAC1	-1,266049023	0,016736787
LOC653801	-1,265155533	0,025907931
LOC133874	-1,265136668	0,000495582
DBR1	-1,264588448	0,043140858
BRD1	-1,264557669	0,009511776
MAST4	-1,263968477	0,000105876
CIDEB	-1,263956469	0,096783966

CPT1C	-1,263567398	0,949613687
TPH2	-1,263211626	0,231192779
CLDN6	-1,262764542	0,1625428
MAD1L1	-1,262006927	0,836726821
LOC402176	-1,259497215	0,026042751
NPHP3	-1,257028734	0,07825656
CYLD	-1,256796437	0,06975854
LOC255480	-1,256770653	0,005330965
MED22	-1,256522639	0,941480985
hCG_1815504	-1,256411	0,098530418
LOC732243	-1,25582547	0,011874576
Zep-2	-1,254295457	0,035396582
MDP-1	-1,252945968	0,132855789
PDGFRB	-1,251625781	0,094247738
GNPNAT1	-1,251619793	0,138387725
H3F3B	-1,251480051	0,285323986
BMP6	-1,25130603	0,138927305
SKIL	-1,251293449	0,221217115
C1orf167	-1,251055298	0,069092435
LOC646982	-1,250741275	0,001101342
GATA3	-1,250194843	0,054617412
POLI	-1,249743187	0,070175645
CACNB4	-1,249715013	0,038082145
LOC649137	-1,249683318	0,01746042
AHSA1	-1,249426952	0,134993627
LOC442160	-1,248612229	0,010728602
CNR2	-1,248170492	0,041049169
CDYL2	-1,246486909	0,070942704
API5	-1,245160891	0,007822548
PPP1R13B	-1,244593763	0,082714401
APOL2	-1,242224775	0,008921255
CNPY1	-1,240956495	0,127536828
PLSCR5	-1,2406806	0,029915535
NOX4	-1,240637671	0,050355715
CEP192	-1,240330245	0,147162177
TMEM9	-1,238821565	0,058526214
PHKG2	-1,238232441	0,05244988
PHLDB1	-1,23799085	0,037002303
R3HDM1	-1,23750312	0,017646572
LOC646019	-1,236803417	0,030922403
MARCH10	-1,234129637	NA
PPP1R9B	-1,234117457	0,007891983
WFDC8	-1,233819985	0,834151195
TOB1	-1,233394326	0,000115638
C19orf29	-1,232927986	0,005215175

HOXC12	-1,23243937	0,103749529
SUSD1	-1,232154294	0,919616686
AK1	-1,231829993	0,026801724
GSH1	-1,231659495	0,5346184
EPGN	-1,231276348	0,003035172
PRP19	-1,230219978	0,027359554
H1FOO	-1,228537433	0,193057142
LASS4	-1,228145087	0,120051178
NFKBIE	-1,226634228	0,117447162
CPEB2	-1,226450421	0,128701516
CPXCR1	-1,225262965	0,301384065
NDFIP2	-1,225037592	0,001084535
HIC2	-1,223087993	0,011998856
RAD52	-1,221506345	0,015936825
TMEM127	-1,22080309	0,032185264
VPS45	-1,220690489	0,003948402
SLC25A27	-1,220096586	0,127874285
CCDC50	-1,218152707	0,6396978
LOC728095	-1,217104721	0,044941404
SOX17	-1,215051963	0,103161481
ASB10	-1,214318456	0,428672215
AZU1	-1,214212821	0,020311973
CCDC122	-1,212700317	0,0013388
RAB39B	-1,212479001	0,001290775
RETN	-1,211886414	0,19248415
CMTM2	-1,211715706	0,304626342
EAPP	-1,211151425	0,07369794
GSC	-1,211114305	0,001519418
HCG22	-1,209941573	1,03E-05
LOC642678	-1,2097521	0,271685175
THRAP5	-1,208475816	0,036266365
MRGPRG	-1,20810363	0,059191776
PDE3A	-1,207931719	0,290827323
LOC730410	-1,206368335	0,00290421
LOC283953	-1,205928904	0,032125798
RAB3IL1	-1,205764229	0,058589574
LOC730110	-1,205510565	0,218445788
REM1	-1,205292244	0,016160584
CYP27A1	-1,205119691	0,700361186
FAM18A	-1,204372952	0,00139633
PANX2	-1,204240411	0,078727946
SMEK2	-1,204125307	0,015438249
LOC647065	-1,20348847	0,001391378
PCDH15	-1,203133671	0,033766377
PTPN6	-1,201945285	0,907984515

SPON2	-1,20189158	0,027083492
XKR9	-1,201758683	0,639608417
LOH12CR1	-1,201123376	0,211382454
COL11A2	-1,20088795	0,074367821
GBP5	-1,200708672	0,082559397
LOC729681	-1,2006123	0,021341716
PDXK	-1,199847157	0,723790386
DCUN1D1	-1,198654288	0,499731333
C2orf71	-1,198075809	0,06025076
MCFD2	-1,197605305	0,671972156
LOC728222	-1,195160222	0,221389997
AADAT	-1,194947991	0,049087816
SIX3	-1,193924298	0,080085675
SPTLC1	-1,19371719	0,159744574
LRRC48	-1,192936883	0,088104862
TPRX1	-1,192898832	0,005717335
BCL9	-1,192607531	0,891458484
LOC729412	-1,192348195	0,045602491
LOC730323	-1,19227034	0,085003212
CA11	-1,192238527	0,002009941
MAPRE3	-1,191230075	0,007293818
BNIP2	-1,190397314	0,1999815
LOC647761	-1,190058429	0,261807755
LOC729849	-1,189924188	0,001387405
C14orf94	-1,189019409	0,475630503
VCAM1	-1,188862815	0,037792202
INSIG2	-1,188650179	0,008884212
CCDC104	-1,188038376	0,078131142
ZNF77	-1,184885367	0,054110388
CCNA2	-1,184787477	0,13641666
GSCL	-1,184315193	0,025179869
NUDT6	-1,184269692	0,142757395
WWP2	-1,183779188	0,047366503
KRT10	-1,183764602	NA
LOC730108	-1,183729237	0,149565492
CCDC9	-1,183394651	0,004504419
OR5AU1	-1,183368638	0,17162871
FOXO1A	-1,183265169	0,001078762
C6orf89	-1,182857598	0,09292483
ATP8B2	-1,182821173	0,165286403
GPR78	-1,182513346	0,023739169
NEK6	-1,182356507	0,686395875
VPS53	-1,181923641	0,004035114
SLC6A1	-1,18189657	0,091602036
DNAH10	-1,18108858	0,035170291

SHFM1	-1,179668848	0,029011481
IQCC	-1,178372866	0,240648194
OR5M8	-1,178096245	0,648817885
GNAQ	-1,17741626	0,176579673
RILPL1	-1,177366458	0,435194463
PDZK1IP1	-1,176629058	0,027971007
SGPP1	-1,176442737	0,309407296
GNMT	-1,175631923	0,103192892
ITGA4	-1,17428177	0,038092323
LOC390245	-1,1737926	0,754371098
SNRNP27	-1,17375674	0,001192174
CRBN	-1,173496347	0,000612073
MYBBP1A	-1,172902258	0,087490597
DSEL	-1,171353312	0,052990018
CTDSPL	-1,171278041	0,032347533
MARCKSL1	-1,171238251	0,02473221
FLJ20257	-1,169979564	0,014151051
OR13A1	-1,16996204	0,199142955
PRKCZ	-1,169947783	0,230139437
LOC653103	-1,16970245	0,030951703
MTNR1B	-1,168826684	0,022078804
ACSL6	-1,168465488	0,130388988
KLRG1	-1,167741087	0,728322663
SRP14	-1,167141868	0,039222041
APH-1A	-1,167096061	0,351620461
IQSEC3	-1,166962934	0,026296162
SLC14A2	-1,166737321	0,307960123
ZNF358	-1,166552109	0,4832619
MUC16	-1,165688309	0,741035258
APOB	-1,165622822	0,084607037
CDH6	-1,164964103	0,014492633
ANKRD33	-1,164876783	0,117085507
LOC206227	-1,164011216	0,002775962
OR10J3	-1,163046309	0,000821389
LOC113251	-1,162979007	0,043016231
C19orf21	-1,162418728	0,281892543
RPS14	-1,162055186	0,045340324
RBM12	-1,161436618	0,072401785
GRIN2A	-1,161433413	0,084904481
SCNN1D	-1,161244634	0,010502302
WISP3	-1,160964222	0,040433519
PPIL6	-1,16090229	0,052127205
HOXC4	-1,160853626	0,019937106
LOC729460	-1,160528783	0,085208066
PFDN5	-1,159088178	0,051407607

MYO7A	-1,158812874	0,154330068
ZSWIM1	-1,158566055	0,017299632
NME1	-1,158183225	0,421904187
LOC392288	-1,15686644	0,186270641
SEC23B	-1,155953637	0,042564071
ZNF333	-1,155676356	0,347101453
LOC392542	-1,155505768	0,015911816
KBTBD3	-1,155312719	0,075403124
EIF2B5	-1,15519288	0,192834366
C11orf2	-1,154478435	0,197378116
CCNDBP1	-1,154393963	0,024173731
C18orf45	-1,153693655	0,265866834
TSEN54	-1,153370312	0,186392736
LOC729582	-1,153325304	0,025669844
PLCB4	-1,153277815	0,093854527
JRKL	-1,15246603	0,047018309
STK6	-1,152393913	0,025344663
HLCS	-1,152378321	0,001862534
ZNF337	-1,151853317	0,31133135
PPIE	-1,151722334	0,039247255
ILF2	-1,151349211	0,019531905
LOC652330	-1,150973264	0,960525305
MAP2K3	-1,150583961	0,147489425
AQP3	-1,150567994	0,496525734
IL1F10	-1,149093079	0,8055526
C3orf52	-1,146881864	0,194192653
VPS13D	-1,146536732	0,066312528
ZNF484	-1,14648195	0,201729549
FAHD1	-1,145677277	0,034175624
RPL26	-1,145633291	0,01821372
LOC645470	-1,145261689	0,014250265
MGC88374	-1,144107999	0,048800217
BXDC5	-1,14406357	0,073263292
OSBPL5	-1,143168107	0,677622813
THRA	-1,143083809	0,13103826
LOC644528	-1,14218864	0,140411242
LOC730413	-1,140019027	0,297067794
AMACO	-1,139516403	0,405144805
MYEF2	-1,1391188	0,035091235
LOC644366	-1,138737651	0,052486188
TSPYL1	-1,138541739	0,066507218
TMEM86A	-1,137985742	0,301751383
hCG_1645016	-1,137967672	0,004438992
BHLHB9	-1,137633498	0,013701508
C9orf86	-1,137562147	0,933510378

FLJ23356	-1,137071049	0,028654006
BPHL	-1,136959957	0,010875243
KLHDC7A	-1,135420951	0,230450067
DUSP21	-1,133755917	0,044451522
LOC644697	-1,133025151	0,210733015
RAB33B	-1,132529335	0,358716906
KRTAP4-11	-1,130618666	0,011017737
KLRF1	-1,128932133	0,035487978
C17orf67	-1,128616235	0,047490757
KRTAP21-2	-1,128449492	0,269813087
ODF3	-1,128356162	0,348223056
FAM13A	-1,128189077	0,000172089
GLUD2	-1,128015961	0,002192633
ACTR5	-1,127665191	0,06347166
CYC1	-1,127584925	0,610014871
WDR5B	-1,127454127	0,002007808
EFS	-1,126976528	0,056239966
SLC26A4	-1,12676822	0,09857291
AMN1	-1,126700161	0,057080704
PHGDH	-1,126637331	0,171726739
PPAPDC1A	-1,125259606	0,169287327
HIST1H4K	-1,125122847	0,251655247
RAB7	-1,124591668	0,774741871
C12orf40	-1,123912396	0,040242217
ERCC6	-1,123887893	0,020791843
NUPL2	-1,123158046	0,087012618
CAB39	-1,122779672	0,252158743
KIAA0841	-1,121506191	NA
PHF3	-1,120890139	0,101767242
LOC442261	-1,119850916	0,012300523
TMEM61	-1,118018524	0,016221625
LOC645446	-1,117995935	0,022840604
LOC649264	-1,117916539	0,38473173
LOC728433	-1,117535391	0,059202299
A3GALT2	-1,116919707	0,024319892
LOC730461	-1,116788768	0,043467779
RP1	-1,11565672	0,713518321
CCDC52	-1,115627671	0,020436296
OR51M1	-1,114768445	0,024461926
LOC391722	-1,113613029	0,237759733
PTAR1	-1,113142148	0,11376931
FLJ11151	-1,111787784	0,105126756
DPH2	-1,111299232	0,249404749
EVI2B	-1,109886189	0,259327453
LOC730059	-1,109615075	0,080172563

SASH3	-1,109270204	0,172800969
LOC387895	-1,109233662	0,092410263
ISL1	-1,108445324	0,221757748
WBP11	-1,108164209	0,00236482
YWHAB	-1,107661017	0,118533517
AMICA1	-1,106902276	0,00987268
GBA2	-1,106844666	0,063829974
COPS4	-1,106060455	0,076284675
KIAA1012	-1,105604188	0,072716697
CDK5	-1,10533964	NA
CKS1B	-1,104746309	0,314387643
CTNNBL1	-1,104614964	0,151385094
C8orf41	-1,104520658	0,000737976
LOC440995	-1,104447671	0,001474752
UBE1DC1	-1,104208599	0,040214605
ETV1	-1,104039478	0,03931696
C14orf43	-1,103651752	0,08273971
CYP2A13	-1,103480229	0,077544517
C6orf120	-1,103284016	0,036290764
SNRPD1	-1,103268586	0,005110541
PLA2G4D	-1,102877998	0,122736801
YWHAE	-1,102709554	0,013654394
SCML4	-1,102607709	0,078164929
NCCRP1	-1,102365793	0,007308129
ALS2CR2	-1,102134305	0,00164278
LOC652673	-1,100705106	0,992501313
RAB3A	-1,099440961	0,018333813
APXL2	-1,099175366	0,005958153
CAGLP	-1,098996123	0,032770802
KIAA0947	-1,098780749	0,818179343
RNF20	-1,098613838	0,063472698
CEP97	-1,096749419	0,002288734
TAS2R3	-1,096012803	0,036282992
PDCD6	-1,094771028	0,029523662
ZNF84	-1,094604259	0,013882902
PKD1L2	-1,094438591	0,019028438
PDCD2L	-1,094080426	0,079470424
SFRS18	-1,093688026	0,18224709
DHODH	-1,09347513	0,003672734
GJB5	-1,092223929	0,238639161
TNMD	-1,091113943	0,016021399
LOC728114	-1,090513665	0,306064944
RHBDD2	-1,08968784	0,004763618
PPP1R16A	-1,088343109	0,088663083
ZNF487	-1,087814902	0,063651061

LOC728837	-1,087692478	0,03331721
SITPEC	-1,087598191	0,01180251
SLC16A8	-1,086673237	0,201258504
LOC345643	-1,085002525	0,030393678
LOC729608	-1,084931582	0,041300189
CYP4F8	-1,083908284	0,444322274
TMEM179B	-1,083894367	0,217709197
CNGA3	-1,083878531	0,013885525
HDHD3	-1,083764989	0,022384596
C10orf107	-1,083004855	0,012243551
MATN4	-1,082846374	0,074177573
FLJ23834	-1,082470842	0,528688058
FAM58A	-1,080243598	0,048476795
LOC649279	-1,08010277	0,072528028
TEP1	-1,080072182	0,059725891
LOC648603	-1,080012495	0,00217763
F2RL3	-1,079938447	0,014507263
ZBTB17	-1,079883968	0,013964789
LOC641930	-1,079751212	0,130618686
hCG_2041321	-1,079099527	0,09032288
RP3-510O8,5	-1,078876921	0,088426335
OR1J2	-1,078735969	0,077370741
DNAJC13	-1,078449755	0,013292676
FRG1	-1,078272258	0,139173035
C8orf79	-1,077961961	0,171317841
WNT8A	-1,077665355	0,073715319
ADAM33	-1,077625356	0,112298733
GYPB	-1,076454221	0,734744041
ZNF184	-1,076271719	0,168920525
ETAA1	-1,075899526	0,017972847
FMO4	-1,075855796	0,064187946
SMO	-1,075419585	0,004934596
LOC653155	-1,075414286	0,002011394
MAP3K5	-1,074761118	0,087850483
LOC729180	-1,074678283	0,007709129
SRCAP	-1,074419413	0,096818504
LMF2	-1,074218984	0,355006923
ARRDC3	-1,073421596	0,004241608
STAG3L3	-1,073000912	0,04438146
LOC645513	-1,072663048	0,012492759
EIF2AK3	-1,072091184	0,015439116
XKR8	-1,072018871	0,81872777
ACVRL1	-1,071666453	0,354799499
INTS9	-1,071647215	0,008620731
BTG3	-1,070509972	0,002048104

AP1G2	-1,069868189	0,03587155
APXL	-1,069701635	0,007340208
LOC728628	-1,069534748	0,23516702
RBM30	-1,068881534	0,027368378
C1orf210	-1,068591828	0,032354033
PSME1	-1,068537606	0,1396617
EIF2C4	-1,06770704	0,009434434
MOSPD1	-1,067598255	0,112919529
VPS36	-1,06759313	0,965010228
HERC1	-1,067283613	0,05394407
SARDH	-1,067084727	0,044922248
RAB2	-1,066575168	0,040986848
BLID	-1,066429558	0,335604094
ADORA1	-1,065705138	0,131850537
GPLD1	-1,065636131	0,101847727
ART3	-1,064732792	0,520193906
LOC253820	-1,064500453	0,015482072
LOC728529	-1,064458163	0,019024141
AGTRL1	-1,064268952	0,189544124
ZNF630	-1,064090248	0,026369197
LOC729377	-1,06408766	0,007126674
CPSF3	-1,063977957	0,081464859
FLJ40235	-1,062945895	0,179217696
OR4P4	-1,062775108	0,000969098
MGC17986	-1,061698154	0,253969203
SH2D3A	-1,061522884	0,000475492
LIPG	-1,061147274	0,088718122
NHSL1	-1,060872562	0,014737273
OSBPL2	-1,060748491	0,539445429
TMEM218	-1,0605985	0,005314678
GPR37L1	-1,060577492	0,405081331
DEDD2	-1,060456777	0,028449812
IL13	-1,060209107	0,183261317
LOC729441	-1,059126528	0,051152659
FZD7	-1,059017926	0,013474091
LOC730215	-1,058921337	0,017887097
C14orf101	-1,058201224	0,008222887
SIAT8A	-1,058117701	0,015995176
LRRC41	-1,057841516	0,764013733
SNCA	-1,057676783	0,157202946
LOC645705	-1,056696808	0,022179871
NFATC3	-1,056398086	0,175650653
MCM4	-1,056034253	0,563676866
TTRAP	-1,055618404	0,123265738
H2AFJ	-1,055552386	0,045633703

CDKN2C	-1,054925507	0,009291772
CDK11	-1,054548297	0,057220416
LOC729748	-1,053512142	0,01695287
ASTL	-1,053365918	0,10953095
KIAA1468	-1,053016319	0,086408015
LOC90379	-1,052927239	0,188427644
IL1F5	-1,052786645	0,015612882
CYLC1	-1,052506721	0,034404343
LOC728110	-1,051402208	0,034478777
LOC727997	-1,051347816	0,469439032
KIAA1919	-1,051340248	0,145314385
AGXT2L1	-1,051052407	0,163662074
SMYD1	-1,050415272	0,107228001
SLC11A1	-1,049610322	0,357308178
LOC643962	-1,049310656	0,144903967
PRIMA1	-1,04856321	0,030592591
ARID3B	-1,048353913	0,025908423
PSMD5	-1,048319682	0,173034522
C15orf52	-1,047720335	0,059127232
SNAP25	-1,046998379	0,0410093
ZNF307	-1,046952379	0,050167809
SCGB2A1	-1,045523942	0,360463409
C2orf16	-1,044776904	0,002688494
ASRGL1	-1,04461688	0,016450014
LOC652346	-1,044399878	0,036825055
NIT2	-1,044221046	0,031162477
LOC387890	-1,042941709	0,043294004
LOC732377	-1,042929395	0,030497566
SART1	-1,041558388	0,139216517
C17orf49	-1,041499183	0,005445736
NCAPG2	-1,041460353	0,960207026
C11orf10	-1,041162351	0,046095418
MGC17301	-1,040560385	0,106763332
LOC731323	-1,040159436	0,076743552
C15orf26	-1,039730634	0,024411572
GJB4	-1,039407187	0,487483166
HESX1	-1,039325761	0,027582572
FLJ20531	-1,039082905	0,01006612
MME	-1,038810588	0,128762303
NR2C2	-1,038323678	0,022727244
APOC4	-1,037997014	0,103107716
KDELR3	-1,037749582	0,386064137
RANBP9	-1,037400513	0,179995267
RHBDL2	-1,036843592	0,141303469
NRXN1	-1,035879658	0,014895644

IMP4	-1,03587641	0,195157869
GAS41	-1,035435592	0,079966611
C6orf64	-1,034259379	0,064490468
LOC728687	-1,034134151	0,420308123
OR56A1	-1,034024354	0,059648642
RDH5	-1,033984314	0,08354422
LOC729020	-1,033171218	0,000441878
OR8D2	-1,033140157	0,002710516
C2orf64	-1,033136882	0,011220738
OR4K1	-1,032310146	0,000579918
GALNT1	-1,032299176	0,010848659
CFHL4	-1,031708919	0,450097104
LOC646581	-1,031460279	0,124090417
DDX31	-1,031443449	0,111155712
OR10C1	-1,031248696	0,301286117
TFPT	-1,030864619	0,072081318
CRAT	-1,030705846	0,468158248
LOC732310	-1,03035238	0,029926006
MOCS2	-1,02997926	0,902233976
CBWD1	-1,029725904	0,373160092
LARS	-1,029457198	0,137731037
LDHAL6A	-1,029446689	0,032096237
KRT8	-1,029229604	0,132423952
LOC728073	-1,029090487	0,002989319
TEX11	-1,028534723	0,001293742
C6orf25	-1,028381194	0,798993378
NCOA2	-1,028343001	0,042142611
PSMC5	-1,028004003	0,087065727
MIB2	-1,027299412	0,031516137
PRDX5	-1,027220182	0,19173508
STARD3NL	-1,027085237	0,29366859
CDKN1B	-1,026471626	0,49517662
STX5A	-1,025889049	0,084102345
PKHD1	-1,024107283	0,949794737
COL12A1	-1,023759743	0,029285773
CaMKIINalpha	-1,023729117	0,386013854
SLC27A2	-1,023469172	0,021352114
ITGB1BP3	-1,023406401	0,275390036
LOC643926	-1,023320756	0,063459905
LOC285074	-1,022708133	0,008617555
SLC17A3	-1,022348148	0,126859171
PWP1	-1,021621084	0,035298628
FLJ36070	-1,021478611	0,166945484
PLAT	-1,021425986	0,089480379
DMRT1	-1,020863491	0,098115149

UBC	-1,020650626	0,352925686
RUNX2	-1,020466047	0,424237608
OXR1	-1,020208973	0,01348195
MS4A12	-1,019749397	0,008200786
TPO	-1,019701921	0,058482261
RIBC1	-1,019641085	0,035055085
LOC283412	-1,01940928	0,039940342
LOC727808	-1,019389294	0,095372791
LOC440981	-1,018795383	0,299149326
CCDC135	-1,01861435	0,075967789
PHYHIPL	-1,018450053	0,554689818
MACROD2	-1,018071356	0,086555315
C7orf44	-1,017571406	0,000868227
ELAVL4	-1,017361557	0,091953781
MAGEA1	-1,016775162	0,068779817
LOC730769	-1,016723009	0,032800412
LOC391322	-1,016343565	0,059059253
PLXNA3	-1,015334133	0,010832768
LOC728516	-1,014908055	0,792924582
CTRL	-1,014870388	0,574311341
OR6B3	-1,014337357	0,13193059
C1orf9	-1,014069014	0,0365908
LOC647632	-1,013558829	0,002867055
IL4	-1,013151911	0,055795535
LOC730025	-1,012852579	0,040896959
STATH	-1,012796739	0,02204841
NOD3	-1,012791259	0,049730432
TRIM22	-1,012687458	0,033162006
USP2	-1,012670895	0,080993665
BTN3A1	-1,012581909	0,018424663
ODC1	-1,012499891	0,110771786
LOC731664	-1,012407883	0,990839559
GPR143	-1,011928948	0,233779576
C1orf226	-1,011802034	0,875935233
DOM3Z	-1,011662306	0,036418873
PLAG1	-1,011591332	0,329225187
RPC155	-1,011522551	0,021958628
KBTBD11	-1,011268085	0,039503621
LOC91464	-1,01125329	0,006966531
SH3PXD2A	-1,010576515	0,022774446
LOC652618	-1,010367261	0,090079996
AHRR	-1,010026857	0,394930214
KIAA1196	-1,009805354	0,104375581
KIAA1244	-1,009638367	0,080576774
ZNF187	-1,009515188	0,045095768

ZBTB4	-1,009337602	0,024271203
MGP	-1,009257718	0,019454457
LOC344787	-1,009146635	0,058084995
PPT2	-1,008949218	0,01467444
LOC256374	-1,008852383	0,081558005
C6orf218	-1,008754265	0,555141061
LOC729505	-1,008663478	0,038977575
MGC27466	-1,008487268	0,626792419
TCP10L	-1,00799349	0,023363494
FIGN	-1,006859382	0,81101957
STAC2	-1,006215261	0,31493098
DAAM2	-1,00599125	0,725890017
ALDH3A2	-1,005968398	0,019879501
TRIM42	-1,005297073	0,024188611
ZBTB8OS	-1,005152199	0,008057884
LALBA	-1,004240852	0,045501065
USP41	-1,003998785	0,005239827
GCOM1	-1,003630842	0,038073369
PTK9	-1,00334321	0,104578427
RPL39	-1,003115273	0,013663197
LOC731718	-1,002678365	0,04606372
EDEM1	-1,002467734	0,639095598
HNRPH3	-1,002266225	0,119520733
KIRREL3	-1,001902298	0,474327086
LAMA5	-1,001757102	0,167237645
TMEM183A	-1,001399923	0,046369648
MTP	-1,001093467	0,976627697
TTR	-1,000993434	0,019180179
SMG7	-1,000914124	0,08753335
GSTT2	-1,000497557	0,098429541
ILK	-0,999259353	0,323895297
GNG7	-0,999090182	0,248601737
C19orf6	-0,999028553	0,00133614
LOC145814	-0,998854566	0,976048621
LOC651964	-0,998539268	0,427873136
UGT2B7	-0,998282259	0,013767494
GPR107	-0,99726898	0,037390593
SMARCD1	-0,997256313	0,565413335
IL19	-0,997235853	0,040473005
LOC728518	-0,996606107	0,027467511
STK32A	-0,996101461	0,054855822
SMARCD2	-0,996030505	0,180805739
C20orf77	-0,995062945	0,072120279
CGGBP1	-0,994425465	0,07595694
EDG6	-0,993920115	0,19566688

RP11-410N8,4	-0,993756275	0,013511244
GRINL1A	-0,993589827	0,334192928
PAGE4	-0,993581126	0,271216311
VAMP4	-0,992941083	0,128716105
LOC729568	-0,992676806	0,013677635
FLJ11506	-0,991329793	0,012114085
ZBBX	-0,991021695	0,940999461
LOC375133	-0,991003154	0,108091745
KIFAP3	-0,990234134	0,026035252
Scrambled	-0,989940199	4,17E-192
CGI-14	-0,989325108	0,5
OR11A1	-0,98780615	0,311667216
GDI1	-0,987497951	0,000795467
LOC728450	-0,986301847	0,006995383
HSPA4	-0,986280382	0,009114379
LOC134145	-0,986254169	0,004111537
MMP7	-0,986135777	0,023760923
C1orf27	-0,985986959	0,006530965
HNRPD	-0,985885444	0,032737422
NRPS998	-0,98492233	0,081888916
ZNF735	-0,984834391	0,056173042
NAG	-0,984820876	0,018530821
SORCS2	-0,984575065	0,435619368
IGFALS	-0,984496495	0,959629177
OR4N5	-0,984425888	0,006120031
METTL10	-0,984187239	0,052315577
ANKDD1A	-0,983998526	0,004934373
SERINC2	-0,983898296	0,098119113
LOC388532	-0,983822993	0,355384841
SERPINE1	-0,983298351	0,025797792
UBE2L6	-0,983194909	0,170681044
UBXN4	-0,982625937	0,080943483
PSPC1	-0,982596076	0,059274467
ANO7	-0,982341984	0,634606422
AKT1	-0,982269527	0,167370375
CREB1	-0,982241067	0,019754391
OR4F4	-0,982017259	0,01001068
FLJ12476	-0,982016756	0,391098065
SLC26A3	-0,981900253	0,164806235
LOC732433	-0,981676232	0,458551628
RASSF4	-0,981294935	0,035983355
LOC147645	-0,981135241	0,001321637
SPATA4	-0,980788809	0,017525629
HTN3	-0,980559961	0,014776562
TIMP2	-0,980476135	0,076249721

PCP2	-0,980397426	0,15648052
FZD5	-0,97985998	0,037684338
LOC653748	-0,979566711	0,011380369
LOC730001	-0,979305876	0,020160994
FAM148C	-0,978759749	0,225372551
MCM7	-0,978668422	0,880130367
NBEAL2	-0,978384602	0,000209012
HCFC2	-0,978353093	0,381418195
GSTA3	-0,978138757	0,028059218
RELB	-0,977435094	0,881609097
TNFRSF7	-0,977200036	0,041367281
TRPC1	-0,97704992	0,002344157
NARF	-0,976990121	0,473480722
GGTL4	-0,976952351	0,103341012
LRRC25	-0,976875042	0,054375973
MPDU1	-0,976431727	0,002008661
KRTAP4-7	-0,976262889	3,62E-05
FMO2	-0,976123073	0,229656117
ZNF177	-0,975917518	0,119784694
LOC728241	-0,975531601	0,16609667
SMAD6	-0,974602008	0,05200335
TNFRSF10A	-0,973732467	0,07311717
EFNB1	-0,97328439	0,036191127
HHIPL2	-0,973033612	0,050849277
LOC730264	-0,972934415	0,144083269
FIP1L1	-0,972620282	0,0090853
AQP7	-0,972470724	0,008872444
AHR	-0,972038579	0,053673994
FAM12B	-0,971725744	0,032638305
HMGN2L	-0,971633995	0,167816248
XKR6	-0,971075504	0,268007638
CAMP	-0,970663264	0,044775694
COG3	-0,970271283	0,001272537
UGT2B15	-0,969630049	0,165733714
LOC728079	-0,969546767	0,057754479
PLGL	-0,969385746	0,012543579
TMEM53	-0,968756211	0,309759805
ABCB7	-0,968553019	0,028282
PPP1R3F	-0,968154494	0,034377224
RAPGEF3	-0,96812357	0,178176847
DPP6	-0,967497785	0,041381045
USH2A	-0,967330998	0,884571046
CCDC6	-0,967158889	0,254142475
USP10	-0,966555282	0,159980897
NUDT3	-0,966019956	0,439129854

MYCN	-0,966018122	0,034696187
ACADM	-0,965837195	0,012976758
C9orf12	-0,965254785	0,225312751
MUM1	-0,964800377	0,075024054
KRTAP11-1	-0,964537954	0,212491099
ABCC4	-0,963856308	0,078672812
SDC2	-0,963217566	0,987818353
CRLF1	-0,963147617	0,018269594
OR6C74	-0,962822578	0,022893756
TM7SF3	-0,962608788	0,17478215
LOC646879	-0,962411761	0,010715663
LOC440839	-0,962286804	0,019302499
DDTL	-0,961975027	0,26921872
RFTN1	-0,961969782	0,323514134
GYS1	-0,961578189	0,04225433
AP4B1	-0,961300434	0,220870779
HIGD1A	-0,96113075	0,312601303
ARL13B	-0,961083355	0,044642887
LOC732402	-0,961026	0,145111728
AIF1L	-0,960928623	0,333779415
ZCCHC11	-0,960408393	0,002422373
BCKDHB	-0,959997321	0,101203826
RPRD1A	-0,95932198	0,097190055
C1orf133	-0,958616054	0,026828929
IL1R1	-0,958524149	0,023222521
VENTX2	-0,958067844	0,902350316
TMEM5	-0,958053501	0,182950225
C12orf62	-0,9578608	0,551990277
OR2L2	-0,957824036	0,92320531
ZNF471	-0,957262427	0,042634678
OR8G2	-0,956974503	0,042262359
FLJ14082	-0,956772361	0,004841147
ASF1A	-0,956318732	0,245438274
PTPRJ	-0,956177663	0,163279479
THA1P	-0,956032288	0,407935563
LOC642343	-0,955572019	0,871073407
ENPP6	-0,955505702	0,35242083
SIRT7	-0,95538017	0,014434827
SOBP	-0,955305719	0,275821447
NTSR2	-0,955200262	0,020083153
HTR1E	-0,955026684	0,076382367
FLJ90086	-0,954816796	0,042201465
ADAMTSL1	-0,954786189	0,038951669
MET	-0,95425221	0,288690738
PVRIG	-0,953765745	0,526459938

USHBP1	-0,953270276	0,014548146
GSG1L	-0,953140875	0,178226842
MAGEB3	-0,953037518	0,002281655
EIF1	-0,952680765	0,569486711
LOC729254	-0,952564843	0,218500409
BCAN	-0,952456352	0,12787663
UCHL5	-0,951842184	0,096878107
PLEKHM3	-0,951328673	0,201429756
TNFRSF1A	-0,951166612	0,243592346
OR4D2	-0,950843643	0,727271685
NAP1L3	-0,950658855	0,073542347
CDK5RAP1	-0,95042741	0,001747172
LOC645581	-0,950248229	0,357749431
LOC151234	-0,949808796	0,882927766
ATP2B2	-0,949477556	0,105476898
CNBD1	-0,949138988	0,062383049
LOC283688	-0,948444043	0,00301495
OR4C45	-0,947461595	0,256093344
CCNL2	-0,947175204	0,123596457
DDX41	-0,946562734	0,003934978
DAAM1	-0,946510463	0,197309389
C16orf57	-0,946415178	0,043128354
FH	-0,946282811	0,964062926
GPR120	-0,946270834	0,04251392
AVEN	-0,946119965	0,048506908
LOC727794	-0,945710182	0,013736925
DOLK	-0,945587167	0,780770005
CCDC33	-0,945524142	0,594408098
RRP15	-0,945407846	0,003528769
ERVWE1	-0,944918427	0,510928985
EGFL3	-0,944705631	0,216827798
LOC728401	-0,944587874	0,099929125
CLN5	-0,94442452	0,342025781
C11orf16	-0,944235467	0,051736886
LOC440706	-0,944189641	0,676315566
OLFM2	-0,944078597	0,098409653
SCN7A	-0,943695918	0,138064254
ICAM5	-0,943275809	0,034785638
AGBL1	-0,943238601	0,058061595
LOC652710	-0,942569641	0,045959735
ATXN3L	-0,942367626	0,018443224
HILS1	-0,942159959	0,078702922
ILDR2	-0,940908009	0,009367704
LOC644280	-0,94086418	0,18627572
LAGE3	-0,940861775	0,046273173

ASAH3	-0,940370677	0,026336953
CNN2	-0,93947559	0,002424024
SLC28A2	-0,939255596	0,350498352
LOC729631	-0,939246764	0,005046654
AVPR1B	-0,939048332	0,010349846
LOC731082	-0,938997102	0,378927489
ACSL4	-0,938261482	0,082683146
RSPH10B	-0,938108671	0,790837016
SIAT8E	-0,937910174	0,046250437
LOC441177	-0,937852886	0,048039812
hCG_1642034	-0,937790463	0,107902417
ALDH18A1	-0,937762874	0,697286693
LOC728318	-0,937636268	0,878013014
LOC392222	-0,93707116	0,002071107
RBM22	-0,937026666	0,728276304
MSL3L1	-0,936949154	0,035915395
LOC654350	-0,936732325	0,57322485
ITGB4BP	-0,936512087	0,334500773
GALNT5	-0,936192311	0,219733058
LOC729636	-0,935996865	0,117751896
ASAM	-0,935646486	0,088804743
RGS18	-0,935606604	0,062135834
UBE2G2	-0,935347565	0,017644119
MRPL55	-0,934922295	0,442073271
SUMO1	-0,934727144	0,397578964
hCG_1983896	-0,934540388	0,006062889
HNT	-0,93402031	0,003566697
COG2	-0,933870952	0,093157567
LOC652147	-0,933827523	0,454855309
LOC728270	-0,933714906	0,161219891
GPR144	-0,933445789	0,077885813
DNAJC22	-0,933357369	0,368447249
MAS1L	-0,932840889	0,004795219
HSA9947	-0,932832547	0,723669164
TRAK2	-0,932682854	0,022653777
OR4C3	-0,93260305	0,209871861
POMT2	-0,932439195	0,271409957
APOA4	-0,93219512	0,351163656
SMC4L1	-0,931370492	0,401959008
MRPL36	-0,931311619	0,102258223
OR51D1	-0,931270105	0,005442061
POLR2L	-0,930785549	0,234134403
LOC728426	-0,930242676	0,183745293
PNOC	-0,930166579	0,017992359
RTP4	-0,929960128	0,086222175

LOC652182	-0,929306497	0,005056236
MLLT7	-0,928596583	0,058143586
DAPK2	-0,928305439	0,009492079
SLC39A14	-0,927882878	0,039548866
BIVM	-0,927704398	0,019610284
INO80E	-0,927571203	0,180221222
LOC643501	-0,927405098	0,020430087
TERT	-0,927236273	0,140252876
CD160	-0,927029021	0,172895407
LOC338809	-0,926859272	0,428790663
EXTL1	-0,926830048	0,009953948
ADH4	-0,926756844	0,447592517
GCSH	-0,926422738	0,378699742
LOC728076	-0,926292162	0,086825291
LOC645641	-0,926156225	0,039019139
ADH1A	-0,926006244	0,17438823
CNTN4	-0,925982314	0,000791493
GPRC5D	-0,92525265	0,081404656
SEPX1	-0,925242628	0,009731387
DDAH1	-0,925121485	0,035281413
C7orf34	-0,925074885	0,167502439
ZNF565	-0,924738979	0,398295064
LMTK3	-0,924523606	0,280015245
NR4A1	-0,924505532	0,175064066
OR52E4	-0,924504539	0,086926597
LOC648708	-0,924319224	0,017416782
HSPC133	-0,924196038	0,082498892
CLEC2L	-0,923250829	0,498987178
LOC730867	-0,923083659	0,092554612
PRKG2	-0,922969296	0,128946556
PWP2	-0,922622304	0,089587808
NTS	-0,922401447	0,724292866
VSTM2B	-0,922380894	0,014844643
TMEM67	-0,922349119	0,898686668
GPR6	-0,921829903	0,24268989
SEC13	-0,920413607	0,007747834
C21orf127	-0,920267573	0,240460285
MMP24	-0,920072156	0,220750415
ASMTL	-0,91971569	0,355049499
LOC730082	-0,919651498	0,778360611
EYA2	-0,919359285	0,049383657
C9orf126	-0,919117604	0,168661174
SERPINA1	-0,919019867	0,408741573
LOC730883	-0,918971743	0,753645445
GPR48	-0,918721771	0,035958102

RSC1A1	-0,918619321	0,07301421
PRSSL1	-0,918400003	0,287231357
WSCD1	-0,918200806	0,79965379
PPM1E	-0,918068101	0,102564151
LOC439950	-0,917035304	0,241468136
RPS7	-0,91673879	0,398205638
FAM69A	-0,915767878	0,13066773
TLE1	-0,91575488	0,117473194
BHLHB2	-0,915535774	0,232279134
FSIP2	-0,915122705	0,800691533
LOC729151	-0,915073184	0,002168267
EVI5L	-0,914532713	0,0312125
PSTPIP2	-0,914365307	0,207817543
NUP153	-0,913910633	0,095028981
ELP4	-0,913436305	0,016833241
APP	-0,913417446	0,039239396
SGNE1	-0,913359854	0,072461081
LOC729685	-0,913257643	0,118807087
CIDEC	-0,913252354	0,057826524
LOC653658	-0,912839432	0,11008208
LOC441016	-0,912675168	0,236996665
ACE	-0,912536279	0,062484385
C16orf11	-0,912283727	0,473972483
LOC652828	-0,911913512	0,928258024
TAZ	-0,911709975	0,219514804
LOC644235	-0,911151144	0,198430374
CLU	-0,911062993	0,161303945
LOC730219	-0,910735374	0,605225122
ZNF35	-0,910610747	0,174002378
ZNF212	-0,910286215	0,175219866
C11orf70	-0,910177171	0,03651234
OLFML1	-0,909804143	0,008117749
PLCL1	-0,909794195	0,943759917
APOL6	-0,909622395	0,887715565
ZNF436	-0,909370803	0,952093787
FNIP1	-0,909285148	0,18743327
LOC644727	-0,909115278	0,141482629
INTU	-0,908826554	0,570339378
LCAP	-0,908792726	0,021495523
SLFN12	-0,908427935	0,060782907
CITED4	-0,907953333	0,12187349
WAC	-0,907317577	0,0429768
PDZRN3	-0,907130118	0,003204559
PINX1	-0,906962414	0,005465797
TP53INP2	-0,9069374	0,173168816

MED18	-0,90625826	0,003299459
SMCR7L	-0,905743768	0,327516606
RTBDN	-0,905575078	0,800832915
PGK2	-0,905552231	0,169781738
RASAL3	-0,90545637	0,961496562
CREBL2	-0,905130996	0,374146187
TOMM70A	-0,905022614	0,015885764
ATP5A1	-0,904788444	0,904039154
LOC731146	-0,904335683	0,171880315
LOC651921	-0,904226705	0,047330399
EFNA4	-0,90414465	0,005397266
FLJ40243	-0,904011829	0,064845276
ORC4L	-0,903982201	0,007745003
HES6	-0,903534264	0,026618009
FLJ22955	-0,903384222	0,456187055
SFXN1	-0,903304094	0,02349765
OR13H1	-0,903215352	0,154456571
NICAL	-0,903140695	0,754138249
CDY1	-0,902682035	0,097874636
IQCE	-0,902476157	0,02204617
OR10S1	-0,902423052	0,82403089
KLF12	-0,90202656	0,085932157
UFD1L	-0,901881236	0,010393295
LOC440093	-0,901800496	0,102272559
C14orf20	-0,901414716	0,97404664
LOC730011	-0,900540818	0,538892902
LOC728961	-0,900496725	0,01915679
KRT1	-0,900335178	0,024641233
C22orf13	-0,900046551	0,000701473
LOC728297	-0,900011077	0,982292712
ARIH2	-0,89988711	0,041247131
FAM129C	-0,899850537	0,499842136
LOC646999	-0,899689185	0,007587299
CPNE9	-0,899377583	0,184534374
LOC649907	-0,899244621	0,00213188
C4orf41	-0,899100441	0,163871351
TBC1D1	-0,898929893	0,117285935
LOC731738	-0,898687564	0,173211964
MPHOSPH9	-0,898578143	0,625726639
MON1B	-0,898549622	0,260208288
KIAA1467	-0,898061446	0,590682172
SCAP	-0,897539952	0,026695856
HIST1H2AB	-0,896707517	0,057010089
C6	-0,896404148	0,043926758
ATP1A3	-0,896335608	0,483323826

TMEM134	-0,896332493	0,122233704
PLG	-0,896085574	0,038020461
RNF32	-0,895828711	0,019258502
HFE	-0,895713162	0,401959304
LOC730183	-0,895549178	0,063376388
LDB3	-0,895445104	0,020555662
UPF3B	-0,895403283	0,046545582
CNDP1	-0,895234639	0,272933127
COX17	-0,895110776	0,837970631
BCL2	-0,895010011	0,03870021
PGF	-0,894718526	0,050324755
RAG1AP1	-0,894467295	0,004440963
KCNK9	-0,894125938	0,054650742
FLJ14153	-0,894082386	0,069812466
IFITM2	-0,894042074	0,004596177
DAGLA	-0,894022756	0,168976355
THOC6	-0,893909975	0,259135757
GALNT12	-0,89375999	0,652889058
GPRC5C	-0,893432746	0,061726173
IL27	-0,893374982	0,005473181
MPP2	-0,893341708	0,819090698
NQO3A2	-0,893314351	0,138102433
LOC652793	-0,893266753	0,259484392
UQCRH	-0,893230355	0,093461492
FLJ43093	-0,892500839	0,055372051
PLA2G2E	-0,891728152	0,158768507
GNGT1	-0,891502812	0,718441944
CLEC16A	-0,891236403	0,267110131
DEFB127	-0,891092697	0,023250336
TOPK	-0,890967932	0,055887847
FTL	-0,890963462	0,046688206
LELP1	-0,890682043	0,502898332
SH3BGRL	-0,89022547	0,882501137
OR52N2	-0,889901566	0,023170722
PTN	-0,88974032	0,103528151
TMEM211	-0,889651954	0,162821588
SLC24A2	-0,889118803	0,012234163
LOC729316	-0,8889601	0,056199072
PRSS33	-0,888873962	0,042989959
10, Sep	-0,888812591	0,033945184
RFX4	-0,888580741	0,203655951
KIF18B	-0,888291666	0,059466447
LYK5	-0,888111758	0,306583875
MGC10561	-0,888074331	0,280005298
ZNF440L	-0,88802426	0,126448128

UMOD	-0,887957936	0,410393482
USP32	-0,887598092	0,034884259
LOC653355	-0,886928342	0,0033173
LOC644092	-0,886468707	0,018793743
MGC8685	-0,886459749	0,112242838
LOC644753	-0,885808348	0,184499382
PARD6B	-0,885733209	0,124273751
HUMAGCGB	-0,88554667	0,049367002
ZNF785	-0,885405549	0,089179357
TTC29	-0,885196335	0,260464762
LOC650024	-0,884969427	0,958591169
LOC732305	-0,8848122	0,400114821
MGC3032	-0,884263843	0,459911348
TCHHL1	-0,884236879	0,066552247
TM4SF5	-0,883969968	0,016974902
THEM5	-0,883902492	0,044168782
GCLM	-0,883902174	0,019703701
HTR6	-0,88383424	0,600663016
LOC729320	-0,883770791	0,240555224
DUSP2	-0,883398228	0,031052576
MCRS1	-0,883379593	0,018724447
DKFZP586P0123	-0,881658647	0,281632881
ZNF37A	-0,881633482	0,030412775
DCUN1D5	-0,881583691	0,00274988
ASAHL	-0,881404435	0,029747717
HSD17B7	-0,881338989	0,134433623
THOC5	-0,881273464	0,019567623
ZNF647	-0,881067355	0,537055936
MERTK	-0,880015426	0,075958391
WDR70	-0,879794454	0,220448509
MUC17	-0,879667333	0,021494437
C6orf117	-0,879657457	0,060672663
TAPBPL	-0,879270936	0,109784387
STK4	-0,878802783	0,523662569
C18orf34	-0,878758082	0,88608445
U5-116KD	-0,878589169	0,148126147
CRISP2	-0,878348202	0,106729587
HTR3B	-0,878021949	0,55616766
KRBA2	-0,877448323	0,005108193
LOC441969	-0,87696358	0,006056382
SNRPD2	-0,876900754	0,079984878
C1orf19	-0,876600662	0,006719335
PNMA1	-0,876539036	0,014626316
FLJ43582	-0,876480366	0,545654305
LOC729037	-0,876210928	0,046557975

PDIP	-0,875440629	0,059043869
OR2T6	-0,875135957	0,007408969
GPR89A	-0,874907282	0,439225039
TUBB	-0,874851443	0,213049481
PCDH21	-0,874778912	0,018044205
TMEM30A	-0,874613707	0,078495296
DDX18	-0,874496866	0,192872748
CTDSPL2	-0,874457671	0,011331903
FRAT1	-0,87394248	0,013676093
CCDC99	-0,873690162	0,192477695
LOC729551	-0,873077332	0,035560303
FXYD6	-0,873055398	0,077324521
PPAN	-0,873002606	0,094110151
OR10H2	-0,872681765	0,006486581
ATP6V1E1	-0,872657778	0,075483855
TREH	-0,872346879	0,317012118
LOC149157	-0,872074636	0,019860791
NS3TP1	-0,871120158	0,086677436
OR6S1	-0,87043102	0,052042157
RGS13	-0,870286474	0,184214389
FYB	-0,870228016	0,002396734
ZNF550	-0,869914599	0,111018609
WWC2	-0,869511808	0,002507335
FAM49B	-0,86943536	0,588542838
C1orf89	-0,869050603	0,19276009
TMEM35	-0,868790334	0,030461101
CLGN	-0,868600492	0,862523424
LSDP5	-0,868435599	0,509320017
DEFB106A	-0,868224126	0,354140708
OPN3	-0,86769254	0,183755935
LOC642277	-0,867655353	0,857746967
MKKS	-0,867083842	0,023161351
MED6	-0,866783132	0,01177922
AKAP2	-0,86649625	0,270339343
ADCK4	-0,866398134	0,0966867
COL10A1	-0,865977625	0,110507468
LOC200030	-0,86593165	0,29420471
C11orf82	-0,865523644	0,083177069
C7orf61	-0,865324906	0,133974757
ARGLU1	-0,864972962	0,129927747
FLJ45139	-0,864919191	0,003789966
GTPBP5	-0,864795579	NA
RANBP3L	-0,864762704	0,566628522
GLCE	-0,864590983	0,050869439
MDFIC	-0,864492542	0,016435333

MGC50559	-0,86440975	0,218992161
MGC34774	-0,864408284	0,232259044
KRT8L2	-0,864360926	0,11900894
LMOD3	-0,864051146	0,003493856
PATL1	-0,86334595	0,010100285
LOC729069	-0,863178218	0,285196582
LBX2	-0,862825743	0,04228524
FAM169B	-0,86276281	0,020532034
PEX16	-0,86226692	0,239745618
MRPS11	-0,862155103	0,020866839
RBM14	-0,861789064	0,132641891
ODF4	-0,861654844	0,345686472
OR8K5	-0,861547367	0,559527113
CCDC70	-0,861185388	0,952699493
C5orf46	-0,860714456	0,002532833
MFF	-0,860112751	0,150963417
IK	-0,860010749	NA
March7	-0,859850267	0,108237549
SLC44A3	-0,85983287	0,11115289
GPR56	-0,859638704	0,106425883
SLC12A1	-0,859602463	0,958747682
LOC647546	-0,859386923	0,010119279
ALAS2	-0,859330253	0,051151334
RP11-45B20,2	-0,859265673	0,618644265
LOC732118	-0,859044231	0,010740138
LOC653789	-0,858998172	0,103068
CLEC14A	-0,858830245	0,369703418
RTP1	-0,858436159	0,080191888
C1orf190	-0,858384915	0,279965812
LOC123688	-0,858326708	0,040922471
PPP1R1A	-0,856966273	0,09491326
KCNQ1	-0,856960006	0,061196445
ATP1A4	-0,856947125	0,199735038
LOC728566	-0,856755125	0,008682523
NGFRAP1	-0,856009927	0,070999326
OTUB1	-0,855393122	0,028156447
SOX6	-0,855351216	0,55721844
DTX2	-0,855192754	0,190329306
LOC388559	-0,855064819	0,017312344
C19orf38	-0,855022364	0,052206487
LOC644659	-0,85499997	0,165792278
RAD51L1	-0,854746704	0,757445051
CCNJ	-0,854701287	0,911061446
AIG1	-0,854695995	0,181370116
LOC153328	-0,854661684	0,179015452

DISP1	-0,854544926	0,039758602
LOC642399	-0,854059675	0,069604542
NLGN4X	-0,853636939	0,497974752
SFRS12	-0,85362643	0,003270563
LOC729848	-0,853095989	0,000135961
ARMETL1	-0,853036135	0,050211664
C5orf5	-0,852493139	0,213054787
KRTAP3-1	-0,852316924	0,033199497
LOC731029	-0,852295235	0,010535437
BAGE	-0,852123733	0,123340585
LRRC20	-0,852018665	0,168178295
ALAS1	-0,851516905	0,216467268
LRP1B	-0,85124635	0,537968644
PKD2L2	-0,851099168	0,136978455
LOC645485	-0,851020188	0,061009804
LOC646395	-0,850964767	0,162527268
OR2F2	-0,850828761	0,087091075
CRK7	-0,850393305	0,062114325
KRTAP13-4	-0,849931924	0,008910039
GDAP1L1	-0,849408987	0,042388861
ANKRD46	-0,849082977	0,674137708
HTR1D	-0,84906233	0,204096525
PPP4R1	-0,848988963	0,053048712
BLK	-0,847604523	0,499750704
C17orf85	-0,847513982	0,048573725
SAMHD1	-0,847384747	0,131501064
LOC642384	-0,847356755	0,019485263
MS4A1	-0,847354645	0,073259664
RPL30	-0,846270878	0,032527563
KIAA2018	-0,846112615	0,108423979
TMED8	-0,846094569	0,177628914
LOC728897	-0,845723401	0,002291404
TMEM126A	-0,845614407	0,060402491
APOA5	-0,845545919	0,239787801
ABI1	-0,845539407	0,26865012
LRP5L	-0,845223319	0,517283915
LOC644623	-0,844764817	0,322684404
RSPO3	-0,844326302	0,016691487
SF4	-0,844202595	0,340538248
C1orf70	-0,844121	0,44368595
ACCN2	-0,843763978	0,237666146
ATOH8	-0,843558009	0,045296034
PKN2	-0,843464645	0,12077515
IGDCC4	-0,843296584	0,183724449
SNRPA1	-0,84286703	0,231832978

ASS	-0,842668834	0,013243296
RNF39	-0,842520938	0,166549725
UCK2	-0,842514593	0,86146413
ASB14	-0,842155085	0,398397958
TROAP	-0,841601093	0,015123554
LOC653198	-0,841539341	0,013400578
LOC730162	-0,841309275	0,009918155
MPP3	-0,841042395	0,060266263
GATA5	-0,840952621	0,040219034
DOK5	-0,84050696	0,298165897
GJA10	-0,840374403	0,114359246
ALDOA	-0,840148166	0,043435877
LOC392713	-0,840113686	0,172861606
LENG1	-0,839789576	0,234490656
RNF110	-0,839718742	0,543263765
BRMS1	-0,839631061	0,011061389
LOC728161	-0,839532341	0,014300727
C14orf129	-0,838858939	0,001239799
IDI1	-0,838532114	0,726102721
PLDN	-0,83851527	0,09622316
ASCC3	-0,838443905	0,021973319
TIAL1	-0,838236252	0,19696825
IL18	-0,837967096	0,026337074
LANCL3	-0,837618291	0,228726624
CRHR1	-0,837240351	0,085008114
LOC646862	-0,837003107	0,106092859
HIST1H2AG	-0,836468677	0,026817887
ACTN4	-0,836119813	0,032508057
DKFZp686J0811	-0,835871581	0,091299626
LOC389607	-0,834729305	0,176546046
LOC646736	-0,834037544	0,278178475
CRK	-0,83375389	0,36293288
ZFPL1	-0,833688813	0,114548736
ZNF22	-0,832853273	0,107071688
SNRPF	-0,832305519	0,146841001
ALDH6A1	-0,832029053	0,041909737
GRM5	-0,831699984	0,086950727
SCGF	-0,831446502	0,191904942
DUOX2	-0,831131078	0,265640225
NPIP	-0,831028634	0,142563163
COX6B2	-0,830935069	0,49922921
ZFD25	-0,830740414	0,11344306
TBC1D29	-0,829825373	0,011930915
LLGL2	-0,82959552	0,019029605
FDX1L	-0,829523099	0,020523374

LOC389124	-0,829371783	0,494294087
MAEA	-0,829343271	0,087269776
RAB41	-0,829239164	0,379049043
CACNA1G	-0,829177186	0,035750297
OR5I1	-0,828675557	0,015161674
INA	-0,828246438	0,029669921
CYB561	-0,828185216	0,054945785
SNAPC2	-0,828062	0,066211147
CACNA1D	-0,827088756	0,194610687
MUC7	-0,826634647	0,029343214
LOC728320	-0,826215696	0,722005665
KANK3	-0,825950441	0,163808217
ZNF33A	-0,825892043	0,452022813
SPOPL	-0,825750707	0,091873315
LOC731067	-0,825331802	0,543805056
GPR133	-0,824971709	0,456868482
UGDH	-0,824543399	0,046876268
LOC730271	-0,824099469	0,148889658
PANX1	-0,823960391	0,019840216
RREB1	-0,823289159	0,037765811
LOC728103	-0,823052049	0,059068204
CD79A	-0,822598014	0,333662712
NMU	-0,822526085	0,148515592
RPL10L	-0,822326903	0,03720521
TGIF	-0,822271024	0,168961436
PRKCI	-0,822248586	0,247936148
MGC29891	-0,821164146	0,03923222
SIAT10	-0,821153592	0,714149299
POLR2J4	-0,821127546	0,040857286
SLC17A1	-0,820515085	0,001380844
HNRPM	-0,820273665	0,04406195
HS6ST3	-0,819664448	0,672141813
PTGER3	-0,819380748	0,280759535
SMARCB1	-0,819234573	0,034949605
FLJ38705	-0,819181376	0,003032514
GALR2	-0,819151237	0,061424274
SLC38A9	-0,81911055	0,087658513
RPL28	-0,81906212	0,035149768
PTBP2	-0,818650374	0,441914402
DYM	-0,818530645	0,17953066
CLK2	-0,818477614	0,848156346
LOC650686	-0,817854807	0,085467805
A2M	-0,817621738	0,563546458
PDE6G	-0,817574309	0,446797316
CFL2	-0,817352787	0,010626269

HIRA	-0,817342231	0,512309065
LOC338963	-0,817061229	0,383804355
CDK2	-0,817009267	0,003801514
TKT	-0,817005464	0,65760854
FGFRL1	-0,816670884	0,452900147
IER2	-0,815993221	0,071303766
ANXA3	-0,815456019	0,072774714
ZC3H18	-0,815420663	0,411126218
SCGB3A2	-0,815357827	0,098565363
DIO2	-0,815279698	0,086805624
MEPE	-0,815257794	0,872629078
SLC30A5	-0,814957272	0,094234699
PCDHB7	-0,814256405	0,062529832
U2AF1L2	-0,81419225	0,094678369
CALM1	-0,813903297	0,074283338
CCL19	-0,813604682	0,032407816
TMEM93	-0,813428889	0,610691577
ENTPD2	-0,813422246	0,574704483
PCF11	-0,813382519	0,04506847
LOC731020	-0,813360869	0,056278147
PTPLA	-0,813344949	0,453268161
LOC732002	-0,813158612	0,012347701
C8orf58	-0,813018927	0,005870941
CELSR3	-0,81300817	0,446784537
C20orf187	-0,812984978	0,453009458
CENTD1	-0,812880746	0,041768399
TUT1	-0,812860217	0,06074512
LOC641728	-0,812855393	0,046215155
CBX1	-0,812632835	0,009822739
IL6R	-0,812553569	0,114496857
SLC15A3	-0,812399043	0,857414107
PITRM1	-0,812147681	0,178446527
MGC34032	-0,812027166	0,032751589
UNC119B	-0,811289373	0,210417453
LIN7C	-0,810941496	0,901099521
LOC643449	-0,810915223	0,894879826
CPB1	-0,810750814	0,346116327
GIF	-0,810679195	0,255748749
HSA275986	-0,810035469	0,769865905
LOC729971	-0,809432761	0,168610681
MAP3K7IP1	-0,809315922	0,24476116
SLC2A4RG	-0,808877908	0,031735563
DSG4	-0,808385025	0,450793243
MYH1	-0,807656695	0,575207541
MARK2	-0,807206918	0,383666691

CCDC28A	-0,806886257	0,388928431
KIFC2	-0,806867911	0,069625134
TXNDC2	-0,806628754	0,063786825
C1orf115	-0,806535657	0,045143676
PLEKHM1	-0,806508618	0,119956433
CENPE	-0,806451395	0,473206033
FUS	-0,805734887	0,16058492
ZNF567	-0,805575647	0,087398669
C18orf26	-0,805497732	0,576629325
C5orf51	-0,805103231	0,033011263
MORN3	-0,804989032	0,085576588
ZNFN1A3	-0,804987019	0,051929651
LOC731429	-0,804954136	0,004791001
CS	-0,804935909	0,050773266
AKAP5	-0,804303985	0,155318013
TMEFF1	-0,804044998	0,031482892
PLA2G6	-0,803793558	0,049952033
ZNF45	-0,803651969	0,076550544
NUDCD1	-0,803636939	0,009807027
SBZF3	-0,803550796	0,033816929
COL4A6	-0,803480174	0,011386796
LOC642486	-0,80344557	0,030134354
KCMF1	-0,803010262	0,061637869
LOC729210	-0,80298345	0,068627189
ZRANB1	-0,802934495	0,268406282
FAM36A	-0,802920624	0,029508653
TRPM4	-0,80252465	0,051469029
LOC131909	-0,802462967	0,330430468
FLJ20220	-0,802337951	0,057206811
LOC651541	-0,801723713	0,485464932
RHEB	-0,801143111	0,442866183
ZIM3	-0,801006287	0,013970556
AXIN1	-0,80060707	0,165727122
FLJ14186	-0,800572638	0,144785154
CEP72	-0,800286309	0,319790215
LOC728761	-0,800159411	0,44408751
OR4C13	-0,800101535	0,010282352
ATP6V0A4	-0,799297084	0,123676202
LOC732435	-0,798826024	0,166100383
PPM2C	-0,798729076	0,054050633
FRMD4A	-0,798280446	0,047169543
LOC727945	-0,797897817	0,783219199
CRIPAK	-0,797818398	0,070307886
C6orf1	-0,797257929	0,00903798
GEMIN8	-0,797180557	0,016385509

LOC730812	-0,797029474	0,051783836
PCNXL3	-0,796897742	0,121368383
AAMP	-0,796737625	0,061283788
TOR2A	-0,796735515	0,006371291
OR10R2	-0,796714094	0,035017594
PROM2	-0,796536078	0,886308756
LOC400027	-0,796524985	0,476588835
CRSP2	-0,796392438	0,122567881
CCDC149	-0,796204572	0,511626281
CA14	-0,795984975	0,322930884
ASPHD1	-0,795537246	0,58919158
ZNF554	-0,795455828	0,020347346
UBP1	-0,795037491	0,109665049
FUT5	-0,794917964	0,398261476
C12orf59	-0,794749324	0,140616669
DKFZp781N1041	-0,794519748	0,126853305
AGTR1	-0,793854905	0,422767704
NDUFB2	-0,793760259	0,294671106
LOC728152	-0,793587695	0,064291723
LOC729819	-0,793042404	0,01311989
GINS3	-0,792822615	0,242501768
FLJ40453	-0,79238803	0,219185485
VSIG4	-0,792104512	0,074349629
CDKN3	-0,791758614	0,209094005
LOC150297	-0,791751764	0,670648517
TBPL2	-0,791737628	0,010207857
LOC643684	-0,791599455	0,41097894
SLC47A1	-0,791366998	0,365162777
ZNF694	-0,79123709	0,081332583
SLC30A1	-0,791138636	0,671776291
REEP1	-0,79109281	0,151234162
CASC3	-0,790808701	0,037963294
CRSP6	-0,790570965	0,077761903
TRIO	-0,790545358	0,067373205
RAD21	-0,790294871	0,231510061
NSBP1	-0,790126972	0,051205659
LOC642578	-0,790026112	0,042725685
H1F0	-0,789813819	0,077593639
MGC15668	-0,789643242	0,071935459
EIF3S6	-0,789193728	0,069375475
OCA2	-0,789045995	0,164145534
DGKH	-0,788958019	0,00376192
BARHL1	-0,788867918	0,069387533
CAT	-0,788807308	0,15635168
LOC644578	-0,788608763	0,038636522

LCE5A	-0,788487662	0,765456374
PLCG1	-0,788411023	0,05787676
LOC731184	-0,788343252	0,11447336
CRYGN	-0,787817384	0,504665547
SLC35B4	-0,787538794	0,22589995
TSPAN13	-0,787278122	0,083987804
FUNDC2	-0,787136092	0,008719162
CNTNAP1	-0,786358805	0,774358588
LOC732242	-0,786036058	0,073434575
TRIM9	-0,785723906	0,088703529
KIAA1543	-0,785650128	0,33865059
SFRS5	-0,785077121	0,009123998
COL6A3	-0,784729805	0,023256557
ESRRG	-0,784689292	0,085706873
GPR156	-0,784500819	0,020872179
NPR2	-0,784171656	0,042519687
LOC728339	-0,783979124	0,026224358
P11	-0,783098471	0,747307222
C17orf65	-0,783088616	0,043258646
DPP8	-0,783051034	1,78E-05
B3GNT7	-0,782800336	0,698282651
COG1	-0,782703801	0,069189227
BZRPL1	-0,782536282	0,506037581
LOC728863	-0,78216273	0,425683964
SLC19A3	-0,781794977	0,442027175
INPPL1	-0,781696926	0,406418699
SLC29A2	-0,781502772	0,833449695
SDC3	-0,781313379	0,026701923
SNAPC5	-0,781027562	0,011780176
LOC377711	-0,780842798	0,058735483
C10orf49	-0,780019381	0,051241958
LOC730084	-0,779864564	0,111861595
SEZ6L	-0,779466924	0,556657153
LOC729996	-0,779018874	0,036756836
TMEM51	-0,778592246	0,06418905
PRG3	-0,778039321	0,111732869
GPR87	-0,777975559	0,100356917
C17orf77	-0,777928217	0,038201254
LOC389641	-0,777802808	0,022145164
CRB3	-0,777503594	0,975096588
ERP29	-0,777156439	0,222685137
MUTYH	-0,776902845	0,140704728
RAPGEF6	-0,776717529	0,153664261
MIDORI	-0,776426164	0,006806293
NRARP	-0,776185356	0,072417665

C14orf179	-0,776170341	0,803772279
C13orf1	-0,776080563	0,273417055
ZNF17	-0,775995119	0,243211357
MAFG	-0,775447812	0,053144903
PRCD	-0,77541482	0,056376193
PREI3	-0,775159897	0,910744954
MED10	-0,774897061	0,022994967
LOC134121	-0,774750024	0,46158628
RHOU	-0,774708722	0,003344673
C10orf76	-0,774555127	0,819063179
MRPL13	-0,774393343	0,021312156
AASS	-0,773975368	0,779458727
GPD1	-0,773948668	0,28245125
CTSK	-0,773841388	0,456595241
PLEKHG1	-0,773832905	0,476898174
SEC24C	-0,773808174	0,274486948
CD58	-0,773670086	0,066087609
ARHGDIB	-0,7733576	0,795885162
RNASE8	-0,773249097	0,020136383
HPS1	-0,773128437	0,048387565
SDHC	-0,772875649	0,269622836
LOC652041	-0,77269448	0,478862929
TACSTD1	-0,772675513	0,592166902
LOC648665	-0,772554043	0,032301242
LOC651055	-0,772436287	0,087409817
RFP2	-0,771709159	0,583978991
LOC123722	-0,77147044	0,296037725
CGI-30	-0,77136238	0,097071442
AHNAK	-0,771321693	0,177810116
IRF5	-0,770562295	0,126902647
OR5B3	-0,770374729	0,001032943
ERG	-0,770283708	0,054728746
MAMDC2	-0,769500792	0,139474452
GAS1	-0,769477074	0,497435529
C12orf48	-0,768837217	0,156575038
C2orf79	-0,768776348	0,071940779
CAPN5	-0,768752872	0,118897515
LOC643665	-0,768671861	0,851561242
PLEKHA3	-0,768584107	0,815071857
ULBP2	-0,768457416	0,094557442
ANXA2	-0,768183627	0,439092077
AXUD1	-0,768022022	0,000850431
CATSPERB	-0,767598104	0,080625518
PI4KII	-0,767488044	0,631975962
ZIC5	-0,767366516	0,929466493

TPTE	-0,766856888	0,051390535
LOC649975	-0,766650194	0,931039918
C3orf23	-0,766482017	0,002389881
NXF5	-0,766443915	0,242916679
SLC6A16	-0,766437565	0,268714048
MOS	-0,766224019	0,026822193
LOC730191	-0,766184756	0,003698952
SLC16A3	-0,766153263	0,431358179
ZYG11B	-0,765810634	0,082016423
LOC654056	-0,765736855	0,029357789
LOC728304	-0,765202808	0,002587584
C1orf43	-0,765135592	0,565398465
HSPE1	-0,765065042	0,120061279
TOP1	-0,764835364	0,054811916
CYP2A6	-0,764740662	0,018587167
LOC728473	-0,764655551	0,044921734
STX1A	-0,764333243	0,362362989
KIAA0284	-0,764320626	0,611113838
LOC149643	-0,763802741	0,014272408
MGC8407	-0,763433809	0,016196149
RPE65	-0,763172799	0,001511227
ANGPTL6	-0,763160411	0,010981848
GNE	-0,762999002	0,109185163
SYT9	-0,762993711	0,012621618
LOC646405	-0,762878497	0,057175424
LOC652879	-0,762876449	0,147760547
MUT	-0,762691303	0,023057891
ASXL3	-0,762518204	0,146475852
CA5BL	-0,762117325	0,00758297
LOC730275	-0,762022711	0,092965049
GUCY1A3	-0,761950794	0,067740071
CECR8	-0,761493038	0,18666288
ADHFE1	-0,761312713	0,129305451
SPCS2	-0,761033027	0,075375891
ALKBH4	-0,760983205	0,013809019
POLQ	-0,760939499	0,018613627
JPH1	-0,760907702	0,136600176
ARMC9	-0,760779755	0,032413185
SYN1	-0,760162465	0,531007046
PDE8A	-0,76007946	0,275669799
LOC731682	-0,759773977	0,103380055
GUCY2F	-0,759723818	0,138266131
TMEM14A	-0,759523853	0,059808778
SUHW2	-0,758754434	0,000705034
HCST	-0,758406036	0,730835246

KIAA1429	-0,758192428	0,041283191
MCOLN2	-0,758037533	0,068280475
LOC440925	-0,757631502	0,956913604
hCG_1732469	-0,757629004	0,052396895
LOC440491	-0,757386823	0,969634491
CCNC	-0,757304232	0,039361427
DUSP5	-0,757088615	0,012120846
CCDC141	-0,756892591	0,136839529
SIAE	-0,756759448	0,18996765
FAM44B	-0,756636322	0,002083596
CSEN	-0,756397414	0,98822371
C17orf101	-0,756191489	0,457607709
KIAA2022	-0,756118972	0,153078995
IGFBP1	-0,755965854	0,221316299
RNF133	-0,755457149	0,782397079
TRAF2	-0,755183146	0,02047982
SBK1	-0,755104925	0,095971681
ANKRD37	-0,755084195	0,13726242
CD1D	-0,755017291	0,196777379
DSP	-0,754715565	0,007043476
C1QTNF4	-0,754555547	0,007806242
DNAJC5	-0,75355864	0,007514776
DSC3	-0,753201013	0,06637419
H2BFM	-0,752868434	0,155197198
PHLDA2	-0,752853145	0,98434382
KLHDC5	-0,75250782	0,077938882
SCYE1	-0,752478454	0,574898737
DCST1	-0,752330331	0,620817354
C6orf225	-0,751870354	0,011397
TMEM14D	-0,75173271	0,279202384
GTF2I	-0,751566749	0,05197297
GKN1	-0,751454977	0,396204319
USP54	-0,75128852	0,066973093
LOC731646	-0,750762638	0,585370692
RPA4	-0,750550487	0,137127756
DSCR9	-0,750484699	0,077267226
SNAP29	-0,750445661	0,047887378
LOC729970	-0,750433438	0,047584564
LOC727988	-0,750237505	0,018943737
KCNE1L	-0,750077643	0,059767137
ABCB4	-0,749814384	0,022173655
OR9G4	-0,749726156	0,02786059
CRB1	-0,749547834	0,067932347
LOC731679	-0,749547256	0,025917366
EGR2	-0,749031214	0,048457032

FAM174A	-0,748753732	0,258024935
DNCI2	-0,74853946	0,151045436
SMARCA1	-0,747813199	0,394898967
C17orf63	-0,74775321	0,036916309
hCG_1729902	-0,747649549	0,093120923
FLNA	-0,747399378	0,327961804
SMEK1	-0,747125548	0,001940161
LOC728931	-0,747061345	0,12929206
SNARK	-0,746415574	0,050703835
SSBP4	-0,746336265	0,117810206
BOLL	-0,746269484	0,067013597
SAFB2	-0,74591288	0,41783707
GORASP1	-0,745807786	0,056051204
LOC344405	-0,745647052	0,089465584
C17orf61	-0,74557758	0,055452708
CCR5	-0,745500946	0,341665554
LOC401677	-0,74532012	0,017964541
LOC728715	-0,745126835	0,043258157
GALC	-0,744903052	0,024418731
B9D2	-0,744787618	0,039911862
LOC389827	-0,744231734	0,015638115
LOC642702	-0,744154073	0,20046498
IDUA	-0,743921193	0,664553926
HBQ1	-0,743093313	0,2818552
TEC	-0,743074149	3,33E-45
NAT2	-0,743014614	0,091892834
ADAM11	-0,742927623	0,051995606
SYT6	-0,742877072	0,101416974
HOXD4	-0,742858612	0,252050413
SCAM-1	-0,742296881	0,386385199
CLEC12A	-0,741789997	0,07077193
IFI30	-0,741754678	0,064060504
LOC729242	-0,741738134	0,083160211
IGFBP4	-0,741633835	0,214203988
GTPBP6	-0,741573701	0,510751507
LOC728847	-0,741352916	0,962629052
ABCG8	-0,741190049	0,29117682
COL29A1	-0,741009859	0,015648591
CNTNAP4	-0,740803901	0,011996256
C9orf119	-0,740652629	0,018935363
LOC729591	-0,740501961	0,474182203
CHC1	-0,740163089	0,925233504
C8orf34	-0,739538523	0,887263501
OR52M1	-0,739469721	0,063858829
C19orf18	-0,739070982	0,001336499

C19orf53	-0,738947352	0,674069384
RPL41	-0,738478697	0,829979368
LOC650556	-0,738285816	0,092601529
C15orf2	-0,738204394	0,02294899
NXPH4	-0,738143541	0,697182788
SNTA1	-0,738035398	0,869379887
GORASP2	-0,737912113	0,160695398
TANC1	-0,737836598	0,290246476
CSPG3	-0,737763535	0,797124635
KCNJ13	-0,737472724	0,068441308
LHX1	-0,736932868	0,082493918
GMPPB	-0,736423717	0,121302882
CYP51A1	-0,73630849	0,27618029
VIL2	-0,736084268	0,035505922
ZNF410	-0,735663872	0,065212889
C19orf39	-0,73560053	0,116451928
LGALS4	-0,735576156	0,075013077
NOS1	-0,73511788	0,152326322
LOC731297	-0,735106028	0,887939002
SAC	-0,735094413	0,058295854
FLJ34109	-0,734969693	0,330597158
PAGE1	-0,734727543	0,0039427
LOC729827	-0,734678688	0,806123925
C4orf29	-0,734274831	0,09676485
MYBPC3	-0,734112295	0,018056628
LOC647070	-0,734086827	0,680688517
FLJ14442	-0,733907851	0,05050014
MGC39606	-0,733841565	0,416930005
ABCA13	-0,733637993	0,505602979
RUNX3	-0,73353744	0,103235131
CYP26C1	-0,733526854	0,057874306
LYSMD4	-0,733501365	0,12496596
LOC729457	-0,733469672	0,048534212
GABBR1	-0,732790416	0,368395865
FAM109A	-0,732174583	0,081085644
C5orf42	-0,73200672	0,814846555
TGS	-0,731755871	0,121689429
PCDHGB1	-0,73163256	0,457852483
DDX4	-0,731345044	0,311206196
PDAP1	-0,73114755	0,112114073
RP11-191L9,1	-0,730937832	0,173175382
SLC25A11	-0,730660761	0,184631791
HBZ	-0,730433206	0,563095316
DBC1	-0,730229857	0,007729295
LOC648405	-0,730181567	0,557956305

ABCA9	-0,730039328	0,587314038
LOC731826	-0,729985262	0,036006696
FOXE1	-0,729848804	0,061286349
LOC730612	-0,729835466	0,334086136
FAM84A	-0,729738764	0,08060307
LOC651101	-0,729172255	0,226293371
LOC131873	-0,728873084	0,026704287
PALM	-0,728670687	0,10237077
LOC729917	-0,728490988	0,028180407
DEFB1	-0,728457024	0,613044066
AOF2	-0,728308694	0,062670016
LOC645904	-0,728250195	0,206754322
KANK1	-0,728159153	0,022477438
RPS5	-0,728064685	0,647063182
TMOD3	-0,727825254	0,619592513
NEK1	-0,727820811	0,021019931
SLC35A5	-0,727408669	0,003531503
INTS2	-0,726374388	0,160036846
MBD1	-0,725921327	0,111035939
CACNB1	-0,725552725	0,355390624
TIA1	-0,725016253	0,154559605
DKKL1	-0,724887564	0,086938626
LOC644066	-0,724871144	0,67296419
LARP	-0,72481725	0,00540551
GBL	-0,724368496	0,000113089
PHF14	-0,723837993	0,049429324
MGAT5	-0,723716481	0,119949145
FLJ14249	-0,723681019	0,797884037
MT3	-0,723541105	0,019275592
GPR125	-0,72341175	0,073868111
IFRG15	-0,723340186	0,411536094
DEF6	-0,723221236	0,00596764
MEF2B	-0,723119029	0,32909292
DR1	-0,722749013	0,035980382
HSFX2	-0,722609896	0,012317118
LOC652610	-0,722588713	0,18751875
LOC645947	-0,722361843	0,005330274
STK22B	-0,721958757	0,217570159
LOC729994	-0,721946913	0,204002889
B3GNT3	-0,721938101	0,200425039
NR2F6	-0,72159394	0,001970132
LOC727960	-0,721556035	0,611992764
MEI1	-0,721328316	0,549833009
PCDH18	-0,721257166	0,169643884
POU2F1	-0,721124906	0,099464722

ZNF406	-0,721079341	0,618449846
CCNB1IP1	-0,72105259	0,556630841
KIT	-0,7209349	0,081590681
LOC731634	-0,720912519	0,879476
C21orf34	-0,720847684	0,048594305
LAMA4	-0,720592748	0,370284639
PLEKHB1	-0,720580594	0,247083647
HMGB2	-0,720211042	0,093539841
MGC16385	-0,720063926	0,426425803
KIAA0907	-0,719909225	0,900097391
ARRDC4	-0,719876015	0,11594025
LOC646301	-0,719743726	0,116730814
DSIPI	-0,719490728	0,026697426
ATR	-0,719396467	0,032711871
EFR3A	-0,719012223	0,101568284
CSRP3	-0,718714089	0,20632061
RCN1	-0,718484207	0,166612604
CCDC61	-0,718436046	0,006888274
LOC730036	-0,718209867	0,646185527
INTS7	-0,718175584	0,196804864
TTF2	-0,718132896	0,206674046
CCNYL3	-0,717954625	0,402876692
KIAA1164	-0,717907436	0,07869016
TP53BP2	-0,717835021	0,483865323
LOC728578	-0,71761178	0,121860824
KPRP	-0,717519963	0,292747345
NDUFV1	-0,717342458	0,259928212
TRMT61B	-0,717176293	0,056659584
C20orf197	-0,717165653	0,02657473
FLJ22833	-0,7171457	0,526492412
SLC29A3	-0,716954921	0,203632
SMNDC1	-0,716462338	0,344968359
TOM1	-0,716255971	0,202399795
TMEM92	-0,716100614	0,356708518
C6orf142	-0,716027298	0,119084315
NOL5A	-0,715799541	0,067561974
MEF2D	-0,715652778	0,066318082
C14orf28	-0,715440026	0,538938785
GVIN1	-0,715360142	0,030910862
DKFZp761P0423	-0,715349314	0,021647756
SFXN3	-0,714939729	0,447355586
FLJ46675	-0,714608428	0,004193191
TMEM97	-0,714532878	0,040898644
ADORA2A	-0,714386997	0,048785126
P2RX1	-0,714030281	0,323392383

CDC14B	-0,712384844	0,471381235
GABRA1	-0,7121949	0,11392384
RINL	-0,71218341	0,075466953
SERPINB9	-0,712022112	0,055447403
GJA12	-0,711831774	0,368654235
SLC7A8	-0,711724169	0,457231258
ELOVL1	-0,711722551	0,588395254
IER3IP1	-0,711280349	0,026995472
LEMD3	-0,710956313	0,924537906
APBA1	-0,710929171	0,183249088
C9orf89	-0,710702719	0,00463893
NF-E4	-0,710288381	0,963311138
SLC4A5	-0,710210678	0,0575112
DUSP4	-0,710053295	0,190042378
HUNK	-0,710030779	0,255940918
CHST3	-0,709997774	0,3131969
C6orf185	-0,709366289	0,170556809
TRIM28	-0,709047248	0,176721269
RFC2	-0,708751392	0,085129002
C22orf39	-0,708633765	0,05217527
PFDN4	-0,708530176	0,048417813
CACNG6	-0,708277183	0,039953584
C8orf54	-0,708254997	0,02215116
KDELR1	-0,708122736	0,113883618
GAK	-0,707901391	0,281592868
CKMT2	-0,707801514	0,682813204
LOC731881	-0,707629856	0,083476168
LOC339778	-0,707605427	0,855553609
C5orf21	-0,707518689	0,213316215
GNB4	-0,707517382	0,582207136
ARP11	-0,707357419	0,438971329
OR52L1	-0,70733627	0,124003497
ANKRD22	-0,706890105	0,04204398
LOC729939	-0,706757259	0,053050726
PADI3	-0,706695848	0,158778472
ABCC1	-0,706563652	0,090611424
LOC644010	-0,706224861	0,220387669
KCNH8	-0,705362382	0,421238539
PDLIM4	-0,705203979	0,012771514
DDX46	-0,704991053	0,258298887
LOC648605	-0,704943937	0,760675266
C9orf105	-0,704688634	0,165718789
WDR74	-0,704516239	0,074748097
EFEMP1	-0,70434581	0,063780226
FLJ14681	-0,703902329	0,014793978

UNQ9391	-0,703873	0,297482815
DLL3	-0,70386972	0,207078159
NT5C1B	-0,703824305	0,628361788
OR5F1	-0,703439968	0,222339766
LOC644993	-0,703338427	0,942262003
DNAH11	-0,703269591	0,015026146
LOC731075	-0,703266464	0,297545551
GOLSYN	-0,70322304	0,908441453
GABRB1	-0,703181813	0,011431277
PPP2R2B	-0,703098964	0,062291121
SLC25A2	-0,702815561	0,047342923
MXRA8	-0,702714337	0,493573756
LOC729451	-0,702628792	0,152036238
LOC730200	-0,702286205	0,965527995
LOC732437	-0,702193566	0,05527144
S100P	-0,702049032	0,630314017
IGFL3	-0,701460565	0,040414468
ARTN	-0,70095897	0,038340568
MB	-0,700661519	0,196320577
ATP6V1D	-0,700130517	0,057067489
S100A2	-0,699839202	0,409909501
AGER	-0,699666265	0,624811725
ACOX1	-0,699244115	0,551060867
FBXO15	-0,698945007	0,020634134
SFRS11	-0,69877603	0,770511211
LOC644338	-0,698752531	0,385030422
LCN10	-0,698670339	0,241537379
FAM92A1	-0,698511427	0,07155881
FLJ44048	-0,697990892	0,195943951
INF2	-0,697771576	0,369438788
LOC728250	-0,696989108	0,026683973
LOC730175	-0,696984769	0,017120863
MIDN	-0,696965861	0,40296076
CDH1	-0,696827606	0,040907175
PFKFB1	-0,696684771	0,283185071
LOC728191	-0,696504617	0,042435428
FLJ39639	-0,696378494	0,032336314
FLJ21628	-0,695837228	0,216394447
PHF2	-0,695687409	0,097166004
ASF1B	-0,695508266	0,13591646
PCDHAC1	-0,695201395	0,012325741
MED7	-0,695143565	0,080232186
C1orf21	-0,695120632	0,033266319
C1QB	-0,69509342	0,137923577
COMMD9	-0,694942382	0,33574079

LOC641857	-0,694732059	0,454732135
TOMM34	-0,694667211	0,040372763
C15orf48	-0,694663213	0,122542405
C6orf223	-0,694627616	0,400777232
FAM45A	-0,694454	0,073846627
GIOT-1	-0,694212907	0,851377107
CRYBB3	-0,693686977	0,044892728
RTDR1	-0,69368325	0,587374685
SENP6	-0,693454237	0,631261647
ATP1A2	-0,693215005	0,531456994
AATF	-0,693065549	0,002371761
TGFBR3	-0,692976032	0,434987023
ZNF564	-0,692885029	0,057083575
PPP1R14B	-0,692789913	0,283961648
RG9MTD3	-0,692744422	0,378601106
TSGA10IP	-0,692611795	0,075380924
HOXB8	-0,692498141	0,648984992
ZNF305	-0,691997478	0,068143095
GMRP-1	-0,691884725	0,225128728
ATP6V1B2	-0,691773016	0,574436097
HSPCAL3	-0,690965903	0,172794973
LZTFL1	-0,690792151	0,012056749
COL9A2	-0,690504971	0,253682568
OVTN	-0,690354074	0,029722257
ANGPT4	-0,690255193	0,645396597
SYCE1	-0,690138744	0,744535922
NPC1L1	-0,68993416	0,021275818
SLC24A6	-0,689814373	0,256139687
WDR45L	-0,689633393	0,029561227
PITPNB	-0,689616114	0,816289258
DDX50	-0,689572449	0,027684753
C3orf35	-0,689522922	0,450662227
C13orf16	-0,689455893	0,006627497
GCC1	-0,689223025	0,168335541
PANK4	-0,689065199	0,08845963
EXOC3L	-0,689038387	0,071596418
SOX2	-0,68897262	0,206218097
PUM1	-0,688828997	0,404248557
LOC647624	-0,688700631	0,392944765
SLCO1B3	-0,688621384	0,048636856
LOC645126	-0,68810153	0,07673299
ITGA2B	-0,688020371	0,019493932
hCG_18385	-0,687700059	0,177639119
SLC22A8	-0,68749239	0,067536047
FAM114A2	-0,687327237	0,001354597

IL1RAP	-0,687091099	0,117296849
GBGT1	-0,68701236	0,347820642
LOC731852	-0,686889174	0,370834036
scrambled	-0,686665244	4,71E-303
FAM82A1	-0,686480451	0,092132566
EIF1AY	-0,686438626	0,843691523
LRRC2	-0,686324944	0,04331605
HMG20A	-0,68611283	0,109515928
SMR3B	-0,685792939	0,132015915
NKX2-5	-0,68544266	0,046483556
ZNF230	-0,685416482	0,087177384
AQP5	-0,68520289	0,207987943
LOC646462	-0,685102497	0,110499097
LOC644779	-0,684404496	0,006480461
CYCS	-0,684344459	0,732590751
FAM160B2	-0,684251515	0,29495137
IVD	-0,684217408	0,300122291
RNF26	-0,684016249	0,079198678
CACNG8	-0,683791729	0,479564418
CSF1	-0,683549769	0,097025759
SLC22A14	-0,683539369	0,448031768
HES5	-0,683497765	0,011321107
LOC729605	-0,683059723	0,054909838
RNF111	-0,682765552	0,046860898
SH3GLB2	-0,682258466	0,011117004
LOC402117	-0,682168	0,961316848
SFI1	-0,682155774	0,08233467
ALG2	-0,682120922	0,832917878
TNFRSF9	-0,682096005	0,046235088
MGAT4B	-0,681555857	0,377512992
LOC389073	-0,680988974	0,673066669
RTL1	-0,680971186	0,926268156
ZNF208	-0,680969008	0,015148264
OTEX	-0,680963075	0,549549618
C12orf42	-0,680590682	0,754203725
HGF	-0,680551263	0,299547359
LOC729769	-0,680434124	0,743732635
FLJ35379	-0,680258169	0,051909707
RAP1GAP	-0,68018717	0,833298504
DKFZP586N0721	-0,680151162	0,09341721
JOSD2	-0,679942841	0,605990185
CATSPER1	-0,679918388	0,354330085
TBCD	-0,679877657	0,085270076
LOC728610	-0,679796271	0,105621049
MRPL4	-0,679774914	0,536337168

PEX7	-0,679330961	0,488059317
TGFB1I1	-0,679173552	0,179097957
SPG11	-0,678768386	0,013849531
C9orf123	-0,678394468	0,048705869
SPANXN4	-0,678293403	0,772646867
FLJ20344	-0,678111096	0,659195592
GPR160	-0,678101781	0,04123783
C9orf72	-0,678090363	0,019073044
XAGE3	-0,677877098	0,35896878
CSAG2	-0,677838095	0,679065386
hCG_1641229	-0,677821375	0,759068029
LDHB	-0,677782182	0,118716047
OR2D2	-0,677756573	0,153344656
FLJ13611	-0,677567014	0,228973953
SARS	-0,677383082	0,127313864
LARGE	-0,677071311	0,001050018
LOC342426	-0,677050137	0,163999809
LOC652675	-0,6769166	0,060810021
GOLGA6B	-0,67675634	0,597260628
TUBGCP3	-0,67616206	0,339949709
LOC648576	-0,676108721	0,015344356
DOPEY1	-0,676010197	0,045120895
CXorf15	-0,6759852	0,059658065
PLXNB1	-0,675780347	0,005646047
ACACB	-0,675678286	0,16624871
LOC641845	-0,675467484	0,566028619
KLHL4	-0,675354286	0,04815079
SCGB1A1	-0,675350455	0,964261596
PPRC1	-0,674935519	0,088598571
LOC643753	-0,67487453	0,006977696
CDK3	-0,674794318	0,000646659
RARB	-0,674602127	0,251064617
ZF	-0,674530147	0,461455537
CCDC79	-0,674490759	0,426436775
LOC390738	-0,674490759	0,285275517
LOC440104	-0,674490759	0,085817765
LOC646865	-0,674490759	0,096099822
LOC647413	-0,674490759	0,037931319
RPL36	-0,674490759	NA
PCDHGB2	-0,674490759	0,037698001
TMEM23	-0,674490759	0,010977486
ZNF704	-0,674490759	0,012474928
PIPOX	-0,674490759	0,256550227
SIAT8B	-0,674490759	0,117731881
RAD51L3	-0,674490759	0,132307228

RTN4	-0,674490759	0,395335556
DYNC1LI1	-0,674490759	0,085866283
GPR97	-0,674490759	0,632932509
LOC646654	-0,674157644	0,03492333
WBSCR18	-0,674147243	0,065537231
SLC25A4	-0,674141703	0,896168259
CNIH3	-0,673949842	0,234086672
NAT6	-0,673877296	0,008993055
C19orf48	-0,673786304	0,038084823
NUCB2	-0,673781731	0,160565632
LOC255649	-0,673564406	0,111638718
SLC18A1	-0,673495947	0,101435914
CYP4Z1	-0,673317182	0,421518971
RNGTT	-0,673253751	0,226208824
ARFRP1	-0,673207623	0,108786403
FBL	-0,673118974	0,340109526
SLC1A6	-0,673106002	0,014305076
RFXDC1	-0,672825003	0,222942237
ACTR1B	-0,672819459	0,165065641
SFRS14	-0,67268051	0,243980915
UNC119	-0,67229791	0,134800866
SDHA	-0,672213286	0,01073114
OBFC1	-0,672145542	0,425341585
OR4K14	-0,671912947	0,337861195
MMRN2	-0,671560168	0,047262364
NACA2	-0,671404906	0,016054
MLH1	-0,671366757	0,04717623
ZNF251	-0,671332886	0,148253739
LOC729607	-0,671256459	0,408286368
LOC442147	-0,671124531	0,458013013
ECSM2	-0,671051187	0,824739848
TMEM19	-0,670847545	0,005616511
SLC38A1	-0,670695284	0,102561132
TACC1	-0,670616802	0,055830747
ZNF100	-0,670587629	0,128686129
LOC642852	-0,670552988	0,230818068
RBP4	-0,670520292	0,108033768
hCG_1995786	-0,670398013	0,028585536
AMPH	-0,670210686	0,82159687
IL8	-0,669877318	0,129892588
HOXC10	-0,669690382	0,538170009
LOC646939	-0,669547879	0,984963128
LOC729187	-0,669488594	0,013982913
IQGAP2	-0,669435536	0,272449342
CDC42SE1	-0,669194365	0,948454476

MIS12	-0,668792654	0,189714566
LOC284422	-0,668479833	0,117703049
DLG3	-0,668186322	0,872130104
MGC34821	-0,668044842	0,033297797
DUOX1	-0,667878578	0,169946847
ICA1L	-0,667815787	0,109129559
LOC649259	-0,667765872	0,61389662
SESTD1	-0,667744497	0,629846686
ZNF215	-0,667716373	0,062258649
LMO7	-0,667668752	0,869784681
NUPL1	-0,667658968	0,57296442
IMPA1	-0,667422836	0,687602043
LOC730054	-0,667415918	0,006584739
SLC6A12	-0,667250371	0,475581479
hCG_1806964	-0,666838994	0,395241345
KIAA0494	-0,666790937	0,308563583
C14orf177	-0,666737391	0,079094811
GUCA1A	-0,666726133	0,659222296
LOC388339	-0,666654211	0,332732758
FAM115A	-0,666379772	0,969807051
SETD1B	-0,666372472	0,029679887
LOC728065	-0,666188979	0,00320816
C15orf24	-0,666069817	0,02589241
ANKRD35	-0,665894197	0,063957364
CCDC138	-0,665671441	0,074032819
TRESK-2	-0,665612782	0,040878903
BAZ2B	-0,665518595	0,484124639
ATG9B	-0,665511746	0,068273195
LOC728081	-0,665484434	0,096205119
WAS	-0,665422898	0,046140848
BIRC4	-0,665275354	0,143689838
MLC1	-0,665247537	0,247213237
LOC731643	-0,665105024	0,1943949
CISD2	-0,665018921	0,719867944
OR10W1	-0,664971259	0,072132634
C14orf83	-0,664871804	0,040564395
PPAP2C	-0,664742839	0,047680167
RAB1A	-0,664508177	0,040326863
LOC730055	-0,66439184	0,594910309
DNTTIP1	-0,663708232	0,731266611
HYLS1	-0,663538143	0,036704792
LOC729834	-0,663374983	0,093269589
KTELC1	-0,662985038	0,003108641
NEU3	-0,662973583	0,624387213
C10orf109	-0,662952078	0,087164007

RFXANK	-0,662178589	0,426430529
KBTBD8	-0,661118537	0,114289783
TIMM9	-0,660864001	0,538441325
LOC650010	-0,660754627	0,016340545
LOC643201	-0,660561959	0,01903592
CCDC42	-0,660503837	0,06131465
NDUFA5	-0,66040746	0,732955509
SUV39H2	-0,660138849	0,214673629
NINJ1	-0,659637105	0,150637028
LOC728240	-0,659472428	0,279012282
TLX1	-0,658985335	0,296553785
MSH2	-0,658887568	0,089071944
PLCE1	-0,658730816	0,119070301
CHST4	-0,658692847	0,058899955
ANKRD6	-0,658641385	0,599876816
VGCNL1	-0,65848656	0,176478232
CD8A	-0,658047526	0,11368302
FLJ16517	-0,657906115	0,089898996
F2RL2	-0,657703221	0,022440812
OPN1LW	-0,657472085	0,046393408
LOC652564	-0,657392476	0,779024586
GSTP1	-0,65714061	0,11802418
CLEC4D	-0,656956847	0,582760977
KIF27	-0,656841062	0,05613013
UBB	-0,656717891	0,465105584
ABP1	-0,656512116	0,039711959
SAP130	-0,656130598	0,121467922
CD177	-0,656066127	0,223604113
ATP5J	-0,656010598	0,164436765
GDF11	-0,655913188	0,535236605
CX3CR1	-0,655635439	0,097913191
HLA-DMB	-0,655193692	0,481118058
HAT1	-0,654960315	0,134390066
FAM116A	-0,654936196	0,064494517
RPS23	-0,654845257	0,030245198
LOC732369	-0,654734825	0,005050092
MLF1IP	-0,654668042	0,07958782
FLJ14624	-0,654661592	0,227530294
IL2RA	-0,654559498	0,21358825
GRIN1	-0,654315621	0,147985485
PHC3	-0,654267722	0,177629537
AEBP2	-0,653844887	0,185000122
BRUNOL4	-0,653404472	0,539396552
NOX5	-0,653125884	0,117603873
HTR1F	-0,653104322	0,121598241

CLDN19	-0,65241829	0,361850354
NUP43	-0,652178982	0,002930628
SCGB3A1	-0,65194551	0,387161107
KRTAP19-4	-0,651861047	0,596471196
ZFP92	-0,651385526	0,062862506
CNGA2	-0,651324972	0,088686416
KIAA0759	-0,651218761	0,065054893
FLNC	-0,651134796	0,958092883
ELOF1	-0,651126385	0,363713963
LOC729938	-0,65107765	0,020931548
MTERFD1	-0,651039553	0,041004878
LOC729942	-0,650839903	0,903748802
CD5L	-0,650774935	0,042511652
SALL1	-0,650492903	0,00676349
LOC641895	-0,650449962	NA
ARFGAP3	-0,650308071	0,105211072
LRP3	-0,649912998	0,013801791
GTF2E1	-0,649718399	0,157372162
ABHD6	-0,649353495	0,082280084
ARGBP2	-0,649134543	0,049558228
OXCT1	-0,649043214	0,17083684
VWC2	-0,649030639	0,005691923
KLKB1	-0,648795918	0,005841369
LOC130773	-0,648611781	0,05267851
LOC729397	-0,648604256	0,220307365
LOC729744	-0,648568088	0,023817455
IRGQ	-0,648506705	0,263158764
C4orf15	-0,648504622	0,343847089
GBA	-0,648269712	0,298305441
LOC728689	-0,648158868	NA
KEL	-0,648009132	0,452687455
MAPK10	-0,647744058	0,015937668
TMEM42	-0,647627513	0,150907799
ECHS1	-0,647624891	0,230201476
WIG1	-0,647604045	0,799106922
RAB6A	-0,647484021	0,039199869
PROM1	-0,647333809	0,04164301
PCNX	-0,647278767	0,233590311
CFLAR	-0,647046551	0,016385579
CASP2	-0,647027025	0,126182438
IGSF8	-0,64683058	0,698314164
FAM91A2	-0,646794734	0,066143679
LOC389217	-0,646681844	0,892420178
MGC39558	-0,646634145	0,005870005
LOC647283	-0,64631382	0,024676305

LOC729730	-0,645901559	0,58596694
ASCC3L1	-0,645042074	0,096014068
WDR90	-0,644657775	0,013642065
OBRGRP	-0,644642378	0,19186944
LOC728925	-0,644538891	0,592969124
JAK2	-0,644531935	0,81601689
ZNF800	-0,644278954	0,087565921
CA6	-0,64400244	0,006184253
SCML2	-0,643923451	0,078888655
PKD1	-0,643639923	0,040790338
LYPD5	-0,64362913	0,903142608
RD3	-0,643553581	0,798499764
SCRG1	-0,643399932	0,001215121
RPN1	-0,643389885	0,249903645
FCGR3B	-0,643377403	0,104576536
PHTF1	-0,643087245	0,520409667
NIF3L1	-0,64307114	0,194206753
C7orf53	-0,6427888	0,597866007
CSRP2BP	-0,642770519	0,887335579
C19orf36	-0,642542334	0,934003831
SFXN4	-0,642468675	0,05239315
HIST2H2BF	-0,642289136	0,805193631
C9orf96	-0,642190185	0,457258418
DNAJB11	-0,642187586	0,339881617
TMEM90A	-0,642119447	0,003977001
LOC730182	-0,641857835	0,106401911
TXNDC9	-0,641751372	0,224492785
ZP4	-0,641712132	0,14213965
VEPH1	-0,641570282	0,101042775
ZNF160	-0,64152244	0,11578107
RBM18	-0,641254737	0,410688079
ZNF826	-0,641133163	0,071719837
PRSS8	-0,640766841	0,073688807
FLJ16734	-0,640334875	0,002953151
UBE2J2	-0,639938948	0,461919728
RABGAP1	-0,639738993	0,365239596
ERCC8	-0,639441915	0,439335412
TBC1D17	-0,639037719	0,161873134
JUB	-0,639008471	0,062896221
LOC646463	-0,639001974	0,071108476
ARHGEF6	-0,638263443	0,971828641
MAP7D1	-0,637886	0,545297325
GPR24	-0,637836274	0,798138601
ALDH1L2	-0,637260224	0,049325766
PRKR	-0,637034484	0,255009356

AOC3	-0,636855531	0,946578282
E2F6	-0,636821268	0,068315253
SEC63	-0,636669923	0,065583433
FLJ44450	-0,636618363	0,182212519
SUCNR1	-0,63659141	0,238521919
DNHL1	-0,636554475	0,208802524
MOXD1	-0,636397915	0,176459661
PCLO	-0,63635933	0,260401864
LOC730032	-0,636143833	0,024754425
TTC17	-0,636089471	0,060805917
ANAPC10	-0,636075792	0,298927394
DKK1	-0,636063888	0,972905842
C6orf203	-0,635864645	0,550689815
CHI3L1	-0,635854855	0,048663684
LOC646590	-0,635758013	0,637299763
GLI4	-0,635298824	0,075067331
LOC728343	-0,635128646	0,433463053
LOC728718	-0,634788214	0,85465009
LOC652629	-0,634539924	0,050955044
FLJ11184	-0,63433573	0,319182444
OR52N1	-0,63418467	0,319605387
FN1	-0,634181557	0,020155662
SHOX	-0,634036186	0,153764045
CCNA1	-0,633834096	0,063613575
GPATCH1	-0,633670621	0,54290075
DNMT3A	-0,633624965	0,916840944
KIF11	-0,633436447	0,108489789
PRSS2	-0,633433776	0,067974778
FLJ20433	-0,633419465	0,008567452
KIAA0748	-0,633323743	0,146847888
AGAP1	-0,633158464	0,653187189
EGR1	-0,632863175	0,518498202
NTNG2	-0,632527059	0,509831995
PAX6	-0,632465579	0,008141562
TMEM219	-0,632207382	0,045290449
RIN2	-0,631767555	0,087671368
WDR59	-0,631700107	0,301096325
FAM131A	-0,631541108	0,057386022
MIPOL1	-0,631454297	0,89321738
CHR415SYT	-0,631226702	0,199952252
DKFZp313G1735	-0,631145584	0,205479333
SFTA2	-0,631076792	0,097580861
RNF183	-0,630819653	0,90212336
LOC728997	-0,630774543	0,105419411
UHRF1BP1	-0,630699552	0,036635439

RPS3	-0,630690791	0,362700436
IQWD1	-0,630674082	0,179183971
STARD4	-0,630577319	0,151315546
OSBPL8	-0,630110338	0,053382443
KCNJ16	-0,629899741	0,740282376
CMC1	-0,629863182	0,953766595
C14orf49	-0,629801034	0,253095955
PCYOX1	-0,629374382	0,009977622
OR2A1	-0,629157855	0,893695882
SPINT1	-0,62868381	0,188718891
RFX2	-0,628561334	0,197307627
NCR2	-0,628446039	0,008490838
LOC730603	-0,628319253	0,022670989
NEDD4	-0,628259507	0,754670415
LOC196346	-0,628247474	0,263360017
GOLM1	-0,628189614	0,432486101
PDZD7	-0,628132524	0,86036929
LOC131055	-0,627473966	0,044316611
SFRS10	-0,627243704	0,183181913
LOC392979	-0,62718479	0,609279995
TRIOBP	-0,627143938	0,438786082
TBX1	-0,627080861	0,730050677
C1orf62	-0,627025374	0,087602281
RYR1	-0,62699047	0,044745243
MEIS2	-0,626773644	0,891372815
FAM110C	-0,62657916	0,019463393
ESX1L	-0,626345691	0,119147588
PRG2	-0,626159427	0,985218136
LOC729594	-0,626004681	0,305876559
ACOT1	-0,625912337	0,313930641
LOC642249	-0,625362605	0,056843073
IRF2BP2	-0,625299305	0,29625635
C16orf86	-0,625036074	0,192326682
GTSF1L	-0,624226345	0,025992511
ENY2	-0,624164834	0,12780866
XG	-0,624118013	0,35035456
LOC340094	-0,624117698	0,063014327
CCL23	-0,62405075	0,084059367
DCI	-0,623855541	0,066464129
TRIM55	-0,623766666	0,494496126
COPB1	-0,623721914	1,01E-07
GALK2	-0,623331658	0,339789721
NFATC1	-0,623297707	0,0675332
FLJ36777	-0,622666194	0,086091895
PLA2G5	-0,622662741	0,078234245

CCDC65	-0,622534353	0,005005901
PYGB	-0,622513606	0,374538223
SCN4A	-0,622387816	0,384307792
ANKRD27	-0,622054129	0,941757563
DENND3	-0,621883101	0,196060444
C5orf34	-0,621579327	0,49593912
SLC2A2	-0,621497424	0,133414647
CLDN7	-0,621431851	0,129057667
CCDC83	-0,621404449	0,585752553
C19orf59	-0,621270249	0,362495756
C18orf1	-0,62120971	0,018701468
RNFT1	-0,621109661	0,083609538
SORCS3	-0,621096314	0,009494238
ZRANB3	-0,620961846	0,150912558
IFITM3	-0,620693595	0,003683745
SYCP3	-0,620524046	0,013316723
LOC650293	-0,620519177	0,1624209
ACAT2	-0,62048141	0,176334637
IGFL1	-0,620320424	0,130568109
SLA2	-0,620081713	0,042715381
KARS	-0,620041746	0,030224878
SFT2D1	-0,620006716	0,504703639
ZNF258	-0,619976451	0,038935011
BCAT1	-0,619881904	0,199425241
GPR12	-0,619837068	0,490700717
C7orf41	-0,619750869	0,953364101
LOC96597	-0,619574052	0,939592293
LOC643855	-0,61915949	0,06971741
RASGRF1	-0,619030797	0,768253909
CCT6B	-0,618942662	0,345351272
ATP8B1	-0,618752174	0,169486062
RARRES2	-0,618676905	0,80252893
C15orf56	-0,618654862	0,045174731
TMEM186	-0,618369495	0,033007472
SRRD	-0,617755246	0,771542502
MDN1	-0,617746666	0,165333817
LOC652634	-0,617425113	0,764942643
SLC22A2	-0,617290075	0,091138463
MOGAT3	-0,617228343	0,53880849
LOC644961	-0,617072165	0,354115461
TRAM2	-0,617010603	0,54789965
GTF2A1	-0,616998564	0,410061427
CAMKK2	-0,616940794	0,156147853
RNF148	-0,616925387	0,871209342
ALKBH	-0,616881186	0,64675898

OR8A1	-0,616343599	0,992748434
CA12	-0,616335226	0,063935774
CBR3	-0,616331667	0,101253489
LOC728354	-0,615994883	0,020148034
LOC390231	-0,61593271	0,11876949
ZNF43	-0,615807875	0,228691041
GATA6	-0,615715091	0,077604295
APBB1IP	-0,61571085	0,861426057
RGS9	-0,615687068	0,037242777
AKT2	-0,615667765	0,177232389
ACAD9	-0,615634928	0,48941029
TMEM33	-0,615377699	0,152061595
NR0B2	-0,615264605	0,107753418
LOC646360	-0,615157511	0,039209601
NUDT8	-0,61515275	0,053904435
CSTF2	-0,615077809	0,057606749
RAB40A	-0,615012935	0,030766711
PABPN1	-0,614973346	0,320772796
ARHGAP22	-0,614919922	0,728830732
OR6C65	-0,614876015	0,102077672
USP31	-0,614804533	0,045795725
CDH26	-0,614523117	0,033690069
CLCA2	-0,614488569	0,588265119
IL24	-0,614416073	0,762007356
PTPN13	-0,614197215	0,628195125
TRIP12	-0,614189883	0,891676243
PNMAL2	-0,61413423	0,351272891
TRAPPC6B	-0,613931449	0,367530798
LOC401498	-0,613911244	0,353131682
PMF1	-0,613884446	0,070420594
OATL1	-0,613685741	0,960340644
OR2AG2	-0,613585385	0,483997123
SIAH2	-0,613556067	0,118474031
ABCC13	-0,613375243	0,206005031
C3orf57	-0,613362044	0,985021665
LOC650218	-0,613113175	0,139128466
MINA	-0,611930082	0,033887158
TMCO2	-0,611795736	0,821696352
FHL5	-0,611757379	0,014286369
S100Z	-0,611347347	0,029867271
CCDC59	-0,610848371	0,211148536
LOC200025	-0,610816121	0,02754881
MSH6	-0,61077239	0,710484508
LOC644556	-0,610718336	0,01137123
NAPG	-0,61068002	0,23954906

PHOX2A	-0,610616548	0,254298568
LOC401317	-0,610330443	0,159603839
ARL5	-0,610302793	0,060624303
ABCD2	-0,609616635	0,016893863
MRPL53	-0,609529608	0,358568215
SSB	-0,609378449	0,049037707
UTF1	-0,609281306	0,726046944
MAP1A	-0,609065042	0,164624904
PRR19	-0,609000721	0,054392434
LTA	-0,608713693	0,82459191
FGL2	-0,608713345	0,019313224
ECEL1	-0,608540457	0,829287372
THEG	-0,60841555	0,039987659
MLLT6	-0,608368998	0,825419215
MCM5	-0,608079746	0,002616806
ZDHHC3	-0,607643758	0,136578028
LOC642636	-0,607609814	0,009278858
FLJ14712	-0,607526924	0,097987951
PTPMT1	-0,607503477	0,140144292
SOHLH2	-0,607386837	0,069665651
ZNF336	-0,607058152	0,343934692
C14orf159	-0,607027752	0,04933528
LOC653458	-0,60701776	0,031192881
PI3	-0,606937857	0,105291591
REG1A	-0,606840359	0,029844048
LOC645434	-0,606824765	0,694212486
KIAA1434	-0,606706349	0,293928457
LOC647131	-0,606541312	0,565620126
ABCD4	-0,606514555	0,555169094
OR2W5	-0,606396656	0,910887937
LOC116412	-0,606223682	0,062824747
NDUFA3	-0,605817266	0,847719775
BUD13	-0,605793633	NA
KCNV2	-0,605739873	0,120356454
MYCBPAP	-0,605733414	0,752221461
SFRP1	-0,605420472	0,00831881
GRIP2	-0,605355751	0,094000316
YY1	-0,605300357	0,126936459
NR1I2	-0,604879058	0,008582149
EPB42	-0,604831401	0,961159205
BIRC8	-0,604804734	0,97737188
GSTA5	-0,604716311	0,621192831
GIGYF1	-0,604348809	0,013951176
LOC645932	-0,604296143	0,010368935
NR5A1	-0,604257324	0,948927446

SLC2A5	-0,60410416	0,084513768
LOC729413	-0,603924296	0,462002657
PRLR	-0,603893016	0,06684432
ELL3	-0,603205504	0,326286235
CLNS1A	-0,603176874	0,979366508
EIF1AD	-0,603016002	0,100155517
LOC729680	-0,60297568	0,114795268
LMBRD1	-0,602870665	0,308106375
LOC646992	-0,602745605	0,197690182
LOC728136	-0,602440815	0,586924763
ABCB8	-0,602362823	0,086228358
LOC730117	-0,60223987	0,817253811
OBP2A	-0,601689433	0,194091098
F11	-0,601533333	0,263130663
LOC731515	-0,601326396	0,908826506
LOC389101	-0,601142087	0,046037023
ELOVL3	-0,600869205	0,280454629
LOC387856	-0,600833704	0,15050132
IKBKG	-0,600818167	0,861651316
CXorf21	-0,600599124	0,966773051
SMC2L1	-0,600585929	0,08324883
TRPV3	-0,600152856	0,095773066
FOLR4	-0,599664338	0,119396396
IGFBPL1	-0,599346821	0,248669845
ATAD1	-0,599193746	0,306400828
LOC644558	-0,599134271	0,26490116
GEM	-0,599054747	0,091821027
PIP5K2C	-0,598964328	0,867223457
C10orf141	-0,59881232	0,617092205
HSPCB	-0,59813321	0,96335197
LOC652809	-0,597884434	0,213416874
EBF1	-0,597550225	0,052648272
ADAM20	-0,59666369	0,068505823
LOC728285	-0,596495448	0,042043616
LOC642536	-0,59615718	0,015388474
SFRS12IP1	-0,595930745	0,837688595
KLHL29	-0,59591975	0,048527942
KDR	-0,595909562	0,19061345
ALDOC	-0,59565724	0,040419946
COL6A2	-0,595592728	0,48162895
FLJ23749	-0,595459658	0,964977153
LOC644589	-0,595445504	0,072412479
WBP1	-0,595238379	0,709550798
AQP9	-0,595237834	0,0142291
CCDC62	-0,595130132	0,017765679

DNHD2	-0,594744438	0,110860467
FYN	-0,59472781	0,198729677
GPR42	-0,594587244	0,033331026
D4S234E	-0,594417678	0,409203931
CDH24	-0,594357927	0,142748271
MSLNL	-0,593941434	0,149808215
FAM9A	-0,593613561	0,485718549
OR6C2	-0,593600496	0,055506378
KIAA1109	-0,593351925	0,142493211
NLRP12	-0,592902951	0,015455388
LOC728874	-0,59279115	0,224710335
ASZ1	-0,592625233	0,167122482
PTP4A1	-0,592525594	0,201718088
ARV1	-0,592490267	0,353428723
C2orf54	-0,592467344	0,381995296
ITPK1	-0,592357099	0,810966613
ADAM29	-0,59210478	0,223712976
RPL3L	-0,591939168	0,040355782
KRT6E	-0,591289124	0,02237205
LOC729192	-0,590771748	0,227585151
KRTAP7-1	-0,590715573	0,040944077
ADORA2B	-0,590704131	0,029847377
PMS2L2	-0,59046947	0,140019016
LOC646513	-0,59038196	0,581004818
HIST1H3I	-0,589915468	0,553332916
LOC441005	-0,589663492	0,005980853
CACNA2D2	-0,589636408	0,127325895
ONECUT2	-0,589489404	0,132811551
LOC731795	-0,589384942	0,012525898
EMP1	-0,589267631	0,67903201
C6orf33	-0,589254953	0,058843329
RPL19	-0,589001418	0,553684659
MPI	-0,588789214	0,030501705
CTCF	-0,588606277	0,103491813
ANK3	-0,588570012	0,024860738
ALS2CL	-0,588484938	0,054886353
LOC731500	-0,588438561	0,017581032
LOC728619	-0,588380237	0,27006822
LRRC37A	-0,588305232	0,019047451
NME6	-0,588226463	0,070788021
PLA2G4C	-0,588224115	0,5137623
LPA	-0,587982612	0,336491436
BOLA2	-0,587828957	0,39948227
LOC728571	-0,58780076	0,132575898
UBXN8	-0,587469154	0,431825925

FLJ11756	-0,586816978	0,267095226
EFO1	-0,586620585	0,128053379
PSAT1	-0,58639413	0,680875897
VIPR2	-0,58622205	0,828255638
SATB1	-0,586145205	0,56792377
CCDC144NL	-0,585880815	0,488128402
LOC728785	-0,585733136	0,476141564
FLJ10891	-0,585587374	0,02626682
LOC728857	-0,585287297	0,144229975
OR6K2	-0,585252793	0,907776309
LAMB4	-0,584261727	0,46997377
CCDC117	-0,58405445	0,696704337
PAM	-0,583253727	0,332228453
OR5D14	-0,583233804	0,039679518
SNN	-0,583111082	0,221465468
TTC12	-0,58290166	0,000355516
LOC728645	-0,582672821	0,538445631
NETO2	-0,582610555	0,126737395
COG5	-0,582474663	0,001110795
PSMC2	-0,582362813	0,417244877
SYK	-0,582345991	0,04287093
CSPP1	-0,582328216	0,513045886
CORO2B	-0,582146539	0,058671705
PPARGC1A	-0,582128231	0,067459464
LOC727853	-0,582100484	0,12739771
CHRM4	-0,581736469	0,02811365
MATK	-0,581600005	0,844077095
UTS2R	-0,581426173	0,115032093
ALAD	-0,581227838	0,008990809
SLCO2A1	-0,581126964	0,10816363
CLEC7A	-0,581025669	0,674676084
EPHA5	-0,580975272	0,056931991
FANCG	-0,580645721	0,03691551
AKAP8L	-0,58064017	0,154885802
HSD3B1	-0,580552698	0,104926602
TIZ	-0,579948495	0,18590722
FAM3C	-0,5797308	0,185186797
PFKM	-0,579675425	0,744555867
LOC643143	-0,57938012	0,035642353
CYP3A43	-0,579189019	0,174644632
MYOHD1	-0,579155991	0,081893569
DLX3	-0,578988798	0,078017299
MRPL14	-0,578822627	0,072136914
HIST1H2BB	-0,578591997	0,370151142
OR2G2	-0,578490318	0,006722799

LOC728264	-0,578003164	0,271171458
GRB14	-0,577929983	0,698969313
STARD8	-0,577692829	0,125253305
C1orf159	-0,577654609	0,611089043
FAM175A	-0,57758141	0,033544129
LOC646873	-0,577309503	0,863652303
ISYNA1	-0,577245081	0,560276485
LOC729986	-0,577096076	0,643611584
C15orf29	-0,577035695	0,355013491
BTC	-0,576971313	0,076416487
OR5T1	-0,576896979	0,220621747
DEAF1	-0,5768394	0,041113093
MAP3K13	-0,576706744	0,020923691
UBL3	-0,576589028	0,921406616
TNRC6C	-0,57633903	0,017893082
ERLIN1	-0,576328203	0,001158516
KLHL8	-0,576219775	0,115811914
HARS	-0,575942921	0,207711283
TRIP4	-0,575436612	0,077376044
CARD8	-0,575404081	0,737528962
LOC731208	-0,575104774	0,261464041
JUNB	-0,574930665	0,433963909
BOP1	-0,574899057	0,688308853
P2RY13	-0,57476101	0,052337108
FAM92A3	-0,574734078	0,357773143
SHCBP1	-0,574732992	0,037324938
APG7L	-0,574697837	0,335783639
LST-3	-0,574588165	0,117311258
PLD3	-0,574581616	0,655075853
LOC643596	-0,57457839	0,098568822
RAB40C	-0,574461412	0,993099594
RASGEF1B	-0,574385833	0,13842451
OR2A7	-0,573998885	0,183401421
TTLL1	-0,573860953	0,784420209
PHB2	-0,573773766	0,064580193
OR52A1	-0,573723429	0,183490777
RBM5	-0,573489241	0,095248684
MRPS26	-0,573269882	0,276865468
MTCBP-1	-0,572710874	0,317894234
LOC642891	-0,57264955	0,054345252
LOC642426	-0,572577319	0,01553273
TM9SF1	-0,572264941	0,18499716
BCAR3	-0,57221677	0,256171329
FGD3	-0,572064629	0,210108134
IL2RB	-0,571800403	0,058607289

RABGGTB	-0,571532779	0,055575389
KLHL11	-0,571362486	0,441672684
ANUBL1	-0,571265447	0,073092056
SRL	-0,57078272	0,100607237
EXOSC8	-0,570720893	0,229474676
FOLR2	-0,570594451	0,940529725
SRMS	-0,570486054	0,24775432
LOC644681	-0,570359824	0,986535204
LOC728032	-0,570120688	0,09607513
PPIC	-0,570055163	0,212387322
FOSL2	-0,569952427	0,069390263
RPS11	-0,569877315	0,05483584
LOC645145	-0,569831817	0,145111882
KIAA1430	-0,569644427	0,075911768
POU5F2	-0,569547194	0,214929803
LOC643623	-0,56913839	0,253825937
OR5A2	-0,569007469	0,108991637
DDX6	-0,568869346	0,840144136
CHRNA3	-0,568864995	0,607145279
HSPB1	-0,568837931	0,069443653
VAMP1	-0,568511197	0,062182646
CNOT8	-0,568369614	0,049010332
LOC729961	-0,568294134	0,079796401
IL5	-0,568046685	0,986703159
ESSPL	-0,567827659	0,12523251
MMP25	-0,567740815	0,285595299
ITCH	-0,567708304	0,118509051
CKB	-0,567624532	0,021019882
C6orf100	-0,567073591	0,078271084
GPR61	-0,566915067	0,138487116
ANKRD17	-0,566691521	0,870587352
MYO5C	-0,566030671	0,069080235
H3F3A	-0,565902747	0,837580132
PLEKHH1	-0,565862479	0,026358952
SLC30A2	-0,565805537	0,424249212
MOBKL2A	-0,565795813	0,078197874
TSNAX	-0,565758709	0,915820493
DPPA3	-0,565583248	0,224125771
LOC644222	-0,565509277	0,474384769
MAP6D1	-0,565507886	0,151769339
ZNF32	-0,5654083	0,164515895
LOC646486	-0,565306099	0,063406288
ITIH5	-0,565076074	0,288508581
STBD1	-0,565063018	0,101276673
ZNF161	-0,564812508	0,117524707

GRID2	-0,564748259	0,323271415
C1orf218	-0,564604693	0,024923455
LOC391205	-0,56418724	0,564639626
ARMS2	-0,564141531	0,065474333
SEH1L	-0,563935881	0,402734444
FOXE3	-0,563845449	0,698925302
THBS3	-0,563785849	0,032466191
PPAPDC2	-0,563520355	0,761424874
EVI1	-0,56345463	0,221854376
HDAC6	-0,563357405	0,767710657
SLC37A2	-0,563325587	0,053636038
CALML3	-0,563196409	0,211655731
ZNF517	-0,563151361	0,752099516
SYDE1	-0,563133217	0,057450057
LOC728370	-0,562963363	0,560112122
HSPB11	-0,56286001	0,051038307
RFXAP	-0,562818819	0,160186865
LOC442461	-0,562774904	0,585188191
LOC727878	-0,562444995	0,111262596
NCOA1	-0,562417472	0,655906445
MTM1	-0,562358994	0,073141083
FMR1NB	-0,562281769	0,235481861
RICTOR	-0,562171746	0,00843926
TSN	-0,561967446	0,779237351
LOC728197	-0,561732601	0,448896077
PLCB1	-0,561651717	0,533913182
ZNF724P	-0,561587241	0,789209606
RHBDF2	-0,561473513	0,398732135
FUT8	-0,56118106	0,202681992
KIAA0323	-0,561162025	0,026015216
MtFMT	-0,561132573	0,050193074
LOC643884	-0,561016286	0,185091425
CHPT1	-0,560915504	0,046070196
CTA-221G9,4	-0,560885988	0,969337463
FERMT3	-0,560822873	0,05520846
TMEM129	-0,560709337	0,097969994
YPEL3	-0,560661168	0,040330687
MEN1	-0,560630431	0,041449853
ZNF566	-0,560388978	0,391101509
ZNF483	-0,560351698	0,105907087
ANKS3	-0,560240319	0,026979367
ZNF15L1	-0,560178835	0,067745098
ZNHIT6	-0,560008128	0,056776867
LOC641936	-0,5599376	0,1960808
RPL29	-0,559888346	0,322000433

NEIL1	-0,55974554	0,57966746
RAB30	-0,559684259	0,157170747
TMCO1	-0,559566875	0,363685746
LOC152742	-0,559544669	0,081954218
RRP1	-0,559403076	0,078306415
TNFRSF11A	-0,559292571	0,674220074
ADA	-0,558979482	0,624156423
IQGAP3	-0,558885898	0,083466866
SLFN13	-0,558702021	0,024723171
TARP	-0,558670564	0,16067267
RENT1	-0,558666521	0,018291311
MSMB	-0,558638063	0,156218987
ACTR1A	-0,558611029	0,102002168
DYRK3	-0,558495315	0,248209274
LOC728326	-0,558330692	0,223016381
NUP37	-0,557927765	0,037939854
S100A4	-0,557719609	0,060495666
CYP17A1	-0,557601199	0,1834412
C3orf67	-0,557240162	0,087578931
ZNF828	-0,557186039	0,090261733
RBM16	-0,557116875	0,073885772
LOC730546	-0,557056016	0,593230847
TMEM214	-0,556951466	0,030175585
COPS7B	-0,556832252	0,433074107
GJA10	-0,556805082	0,064846824
LOC646348	-0,556531022	0,021112429
LOC283710	-0,556414349	NA
CXorf45	-0,556395103	0,044918199
GPR108	-0,556174855	0,2373014
MRPS18B	-0,55612753	0,006736423
DFNB31	-0,55609655	0,143822748
LOC729697	-0,555882952	0,027269041
PKIG	-0,555853853	0,703448273
SPAG4L	-0,555822344	0,224819265
OR7C2	-0,555643253	0,67618634
KIAA0449	-0,555189544	0,122641669
UGCG	-0,555097125	0,16489162
PNMA3	-0,554866651	0,680091879
TM6SF1	-0,554852206	0,264485362
DIDO1	-0,554802172	0,092272185
OR5M10	-0,55478774	0,079936721
MGA	-0,554734674	0,13172983
LOC388474	-0,554640371	0,268522784
TMEM201	-0,554465879	0,899616976
PRKY	-0,554445321	0,855110596

FGFBP2	-0,554423164	0,099388307
C5orf48	-0,554394019	0,179977483
POMT1	-0,554297911	0,141039361
hCG_2044975	-0,554265637	0,776014937
ADAM2	-0,554126386	0,005189646
HIST1H2AC	-0,55400658	0,050774519
VAMP3	-0,55391925	0,117075913
CHST9	-0,55383156	0,524918081
MTMR8	-0,5537673	0,04623741
NMNAT2	-0,553673819	0,527064378
LOC729044	-0,553636021	0,180591089
PDZK1	-0,553592934	0,039954618
LOC649620	-0,553516664	0,055610627
ZNF525	-0,553384174	0,564448454
CASP14	-0,55334083	0,434810103
TRIM25	-0,553013823	0,185350425
LIAS	-0,552914746	0,104981891
F13A1	-0,552899128	0,904090337
LOC730471	-0,55275907	0,324147516
LOC641922	-0,552702892	0,007924639
PPM1B	-0,552414466	0,097359009
LOC729070	-0,552260929	0,813801237
TUB	-0,55225354	0,92868091
TFB2M	-0,552149592	0,749176933
LOC442132	-0,552148164	0,325505658
PIAS3	-0,55211069	0,811485755
LOC731109	-0,551972632	0,1300727
PADI1	-0,551966757	0,182207317
SIAT7D	-0,551940207	0,280719002
RGL1	-0,551563264	0,608638025
CTSE	-0,551537418	0,054080329
LOC728347	-0,551427246	0,7891826
LOC649238	-0,551420164	0,370561491
BOLA1	-0,551344467	0,113461053
DKFZP586H2123	-0,551191658	0,03985635
C9orf4	-0,551165301	0,581688061
ADRA2C	-0,550995788	0,409667388
ZBTB11	-0,55098213	0,511622889
LOC644759	-0,550761125	0,04887818
LOC643224	-0,550545071	0,204329064
SCAMP4	-0,550285972	0,639948962
SPIN3	-0,549987441	0,006976142
CCL27	-0,549747223	0,049668445
EPM2AIP1	-0,549705518	0,348754115
ADAMTS15	-0,549682804	0,029327333

P53AIP1	-0,549564642	0,019244134
LOC730165	-0,549198092	0,033501276
CTTNBP2NL	-0,54911435	0,48701732
ZNF165	-0,549114243	0,779316101
LOC653788	-0,549102979	0,062794601
ALOX12B	-0,549011049	0,185595076
BCS1L	-0,548857632	0,907431691
TNFSF12	-0,548329419	0,291195907
EFCBP2	-0,548323322	0,872594068
LOC730229	-0,548162195	0,361234001
LOC729230	-0,54811423	0,185359342
LOC729736	-0,547968297	0,545629389
KCNK4	-0,547781561	0,295732817
CKMT1	-0,547747457	0,018165553
CLEC6A	-0,547465107	0,363662798
BCL2L10	-0,547171906	0,012074032
BECN1L1	-0,54707469	0,053172924
PDE4A	-0,546948551	0,005132713
EIF2C3	-0,546855283	0,327266781
KIAA0319L	-0,546765156	0,082803547
Sep 15	-0,546760437	0,78711052
CDH5	-0,54668577	0,121674883
C11orf84	-0,546627407	0,916270093
EVPL	-0,546594633	0,152365433
C6orf153	-0,546554699	0,790080086
PTCD3	-0,546551284	0,822509604
ATG10	-0,54628078	0,317725202
KIAA0574	-0,546274257	0,016372971
DCTPP1	-0,546194467	0,362410938
RPL12P8	-0,546184801	0,12199958
B3GALT5	-0,54617038	0,825395767
FCGR1A	-0,546143919	0,017167578
LOC647153	-0,546023511	0,121305294
SLC5A12	-0,545849565	0,157307398
DAND5	-0,545842641	0,05831888
RARRES3	-0,5457807	0,26817126
CHMP4B	-0,545766092	0,06188333
CCPG1	-0,545598235	0,646138864
MAPK4	-0,545517823	0,149221454
B4GALT5	-0,54532624	0,870196647
MTMR15	-0,5453191	0,360359704
GTF2F1	-0,545007246	0,189499005
ADAMTS5	-0,54496429	0,320722055
LOC401431	-0,544434419	0,881503579
KRTAP9-5	-0,544415982	0,445623013

DNM2	-0,544375691	0,224459343
TULP4	-0,544347818	0,058160909
CENPB	-0,544282203	0,209498663
ATF4C	-0,544040836	0,012847162
OGT	-0,543769378	0,295225245
C15orf15	-0,54347881	0,127412391
ZNF214	-0,543449782	0,821546711
EXTL2	-0,543295027	0,033342313
SERPINB13	-0,543097661	0,293012932
IER5L	-0,542767203	0,538901479
MPN	-0,542760747	0,25860495
C22orf15	-0,542730289	0,473941532
RBM7	-0,542692439	0,074121623
GABARAPL2	-0,542503866	0,282805037
FOXJ3	-0,542434087	0,205416894
CROT	-0,542272991	0,542416341
JAK1	-0,542254854	0,607428252
C1QL1	-0,54187586	0,058411332
GALNTL4	-0,5418582	0,039347735
BTBD12	-0,541765889	0,487261242
SUMF1	-0,541577881	0,342253863
FUBP1	-0,541311727	0,345380135
KCNE3	-0,541308702	0,192811668
APG-1	-0,541202941	0,462043787
CARD6	-0,541070813	0,371526689
OR7G1	-0,540912112	0,214726655
ATP7A	-0,540624637	0,003611896
LOC731116	-0,540520429	0,149990439
IFNAR2	-0,540282237	0,524779971
POLR3B	-0,540185369	0,115249586
IGFN1	-0,539811188	0,028389124
IGSF6	-0,539797891	0,75580912
FNBP4	-0,539282113	0,007822463
SLC31A1	-0,539235231	0,86557796
KIAA0605	-0,539175837	0,022505834
CLDN20	-0,53864975	0,079775179
OR4E2	-0,538633239	0,708899232
LOC728922	-0,538329079	0,378612033
LOC731473	-0,537795465	0,110201237
FLJ16686	-0,537789922	0,755766536
LOC652788	-0,537719278	0,138675913
FLJ13195	-0,537702882	0,159170627
MGC34680	-0,53716061	0,775366479
IHPK1	-0,537125168	0,985924834
HUMCYT2A	-0,5370015	0,544018028

CYP2F1	-0,536913056	0,181134275
CSF3R	-0,536774345	0,553897144
UMP-CMPK	-0,536747743	0,050013742
ALG12	-0,536405685	0,903392408
DNAJB1	-0,536173319	0,501402184
LOC344382	-0,535892531	0,271855139
TM7SF1	-0,535325293	0,008758889
XIRP1	-0,535243404	0,274865858
PLEKHA7	-0,534930963	0,09307933
IL18R1	-0,534864516	0,111547719
IARS	-0,534781315	0,307382323
B3GNT5	-0,53468908	0,30777539
POF1B	-0,53465853	0,098137709
SSTR3	-0,53441677	0,46388484
STX6	-0,534267025	0,495710643
NAPEPLD	-0,534226559	0,473395783
LOC732022	-0,534208427	0,49231981
RP6-166C19,11	-0,534178062	0,019571222
06, Sep	-0,533951963	0,918193778
OR5T2	-0,533944184	0,092962164
C9orf64	-0,533611756	0,081050923
SET8	-0,533440145	0,50179918
OR4F6	-0,533418864	0,005848953
AIF1	-0,533263797	0,485690681
SSTR1	-0,533031805	0,076918015
C11orf80	-0,532666257	0,047515321
SFRS2IP	-0,532656552	0,02023209
ANGPTL5	-0,532390035	0,037682196
LOC257407	-0,532242783	0,843800846
HIST1H4B	-0,532086541	0,044059185
LOC645848	-0,531988899	0,012372833
CXCL9	-0,531943413	0,187331791
LOC388177	-0,531925095	0,28966871
RPL31	-0,531785398	0,17098165
TMEM202	-0,531536104	0,637342822
RGS19IP1	-0,531435052	0,083979637
PLXNC1	-0,531350102	0,31956506
LOC399881	-0,531338285	0,165521481
KLHL28	-0,53120449	0,102761331
FOXF1	-0,531203774	0,038420816
C9orf50	-0,530763253	0,857907978
DYRK1A	-0,530749041	0,202105532
GLIPR1L2	-0,530741741	0,063175907
MAP4	-0,530348761	0,601081791
MSGN1	-0,530310281	0,007072728

LOC732477	-0,530177713	0,190947399
NUDT13	-0,530154291	0,283174572
LOC728275	-0,529947391	0,151721615
LOC731101	-0,529787335	0,066821182
LOC730098	-0,529664521	0,086893697
LOC654264	-0,52959134	0,091841722
TRAPPC3	-0,529569044	0,70829134
RPL15	-0,529430382	0,453313169
FAM186B	-0,529370822	0,360276887
TPX2	-0,529317517	0,209173521
HERC3	-0,529187589	0,92108546
AMTN	-0,529030073	0,064203287
LOC730835	-0,529004436	0,361006435
TBC1D12	-0,528831988	0,261646723
IFNA2	-0,528778264	0,026330464
WDR67	-0,528766283	0,137897075
NXT1	-0,528643656	0,718210328
TBCA	-0,528602569	0,621745996
CPA2	-0,528554475	0,165246125
RGS7BP	-0,528362374	0,03538903
CXorf23	-0,528301388	0,020576196
HMGCLL1	-0,528205521	0,544989633
LOC390800	-0,527930129	0,031358616
HOXD13	-0,52776979	0,086831182
CCNL1	-0,527755415	0,397280264
LOC729170	-0,527486836	0,064226959
NMUR1	-0,527366394	0,059891983
LOC732275	-0,52718937	0,69313628
COPS6	-0,526913381	0,200605191
UBE2E1	-0,526641744	0,108023619
TMX2	-0,526156788	0,601053195
NLRP13	-0,52614357	0,022134157
PCDHB12	-0,526001394	0,363975199
LOC730100	-0,52597045	0,364693397
LOC729195	-0,525817806	0,131402724
TRIM29	-0,525753282	0,525312015
KCNJ1	-0,525675817	0,025392263
C1orf34	-0,525662111	0,097976643
GPR177	-0,525612144	0,125786877
CLDN14	-0,525587001	0,030027213
AK2	-0,52551546	0,197413585
DNPEP	-0,525461222	0,108469359
GPR128	-0,525451227	0,057467827
LOC148206	-0,525383701	0,091625719
DAG1	-0,525360246	0,039464431

LOC728597	-0,525347333	0,120049877
LOC728166	-0,525214602	0,745315026
PDK4	-0,525197899	0,867193605
FAM74A3	-0,525153403	0,161043437
SENP2	-0,52480761	0,378737235
OR9Q2	-0,524806744	0,324638107
HSPA9B	-0,52471087	0,347287806
SUCLG2	-0,524561972	0,299232873
EIF3S9	-0,524555042	0,107821203
CHCHD5	-0,524494374	0,008691183
XDH	-0,524369317	0,103670685
C20orf141	-0,524133838	0,209649066
VPS4B	-0,524056551	0,287828095
FGA	-0,523482327	0,023531069
YAF2	-0,523411031	0,077336319
LOC644686	-0,522983049	0,063103207
PDGFRL	-0,522950497	0,196651356
ZNF627	-0,522754624	0,343975039
MBNL2	-0,522739206	0,859449965
ITPKC	-0,522675232	0,72359221
HEXB	-0,522613144	0,437522858
PTCHD3	-0,522574834	0,775313184
CXorf41	-0,522569858	0,834414709
AMFR	-0,522514321	0,317537709
LPAL2	-0,522471675	0,641595838
LOC642515	-0,522424634	0,766552281
LOC643875	-0,522384607	0,218409758
HRAS	-0,522305218	0,021268403
GRASP	-0,522180771	0,929299547
LOC728089	-0,522172062	0,843927878
CD207	-0,522104187	0,540211656
APOL3	-0,522086429	0,192039006
FBXO25	-0,52185194	0,073630285
LOC644216	-0,52179941	0,062159515
STAT1	-0,521714067	0,044745596
CENPQ	-0,52155214	0,071377346
SPG20	-0,52125005	0,006757778
FSHB	-0,521194005	0,13536378
EEF1B2	-0,521152764	0,359874731
LOC284215	-0,52096272	0,956878425
LOC727818	-0,520898867	0,04580264
LOC732381	-0,52089817	0,024228056
FBXL2	-0,520735529	0,173885533
LOC729747	-0,520705047	0,221749487
TMEM55A	-0,520634008	0,658226113

ICAM4	-0,52062707	0,113346067
C7orf11	-0,520443383	0,068438889
MDGA2	-0,520293467	0,019236736
ADRA1A	-0,519508216	0,62467385
DPPA5	-0,519418559	0,163825845
FAM179A	-0,519303778	0,558299878
IL23A	-0,519300843	0,158710064
ANKRD32	-0,519209047	0,042943736
ZNF444	-0,519063983	0,178978705
LILRA3	-0,518876726	0,626951323
LHFPL5	-0,518792707	0,456266487
LOC732208	-0,518701418	0,040103387
PA2G4	-0,518392887	0,171415267
C7orf33	-0,518233157	0,040229012
MFN2	-0,517936969	0,121669709
POLE	-0,517909726	0,703122374
KRTAP4-5	-0,517903967	0,720767168
CENPK	-0,517867473	0,299078532
C14orf119	-0,517770764	0,130508431
FAM118B	-0,517703834	0,025596076
PAPOLG	-0,517582202	0,177532617
KCTD1	-0,51740711	0,070190814
LOC644624	-0,517283808	0,115917551
CKAP2L	-0,517203314	0,269773656
LOC729307	-0,517098613	0,120707456
THOC7	-0,517008275	0,096754928
MYF5	-0,516802319	0,315553612
HORMAD1	-0,516090512	0,284559899
CHCHD1	-0,516085261	0,274591953
CTSC	-0,515877687	0,863307763
CNNM1	-0,515606673	0,094508144
CRY1	-0,515036553	0,118510307
LOC646588	-0,514954301	0,509055144
KIFC1	-0,514928318	0,126974213
HIST1H2BJ	-0,514822993	0,003358125
EXPH5	-0,514366071	0,751324553
ADH1C	-0,514335039	0,341930372
TNFRSF19L	-0,513985581	0,553039752
DCC	-0,513953106	0,12291495
CCDC64	-0,513517444	0,202812213
FAM134C	-0,513497701	0,013236981
LMAN1	-0,513280661	0,076571154
C9orf25	-0,513202601	0,078231177
SOD1	-0,513156907	0,11322932
MRPL40	-0,513151118	0,326581443

FAIM	-0,513036013	0,617168098
DGAT2L7	-0,512901189	0,048353589
DUSP6	-0,51285409	0,590844896
LOXL3	-0,51280422	0,028765071
HRASLS	-0,512663194	0,039922303
ANKRD13D	-0,512402884	0,498889902
LOC729333	-0,512222121	0,024721173
LOC153364	-0,512031195	0,067998395
EIF4G2	-0,511866876	0,860640861
PDK2	-0,511598505	0,739203758
KLK10	-0,511439912	0,226811043
LOC646556	-0,510821198	0,319736618
PPIL5	-0,51081343	0,030762639
LOC648795	-0,510714243	0,058274791
PODXL2	-0,510424242	0,011976557
C16orf48	-0,510288421	0,137482029
CASP6	-0,509953714	0,035475727
BCL2L2	-0,509654696	0,218669523
TM7SF4	-0,509646516	0,635643167
SHQ1	-0,509373474	0,094535664
NXF3	-0,509371653	0,015081885
LIG4	-0,509318984	0,074510016
LOC727982	-0,509235356	0,39087287
MTCP1	-0,509009071	0,71903915
ZPBP	-0,508940632	0,003853417
BDKRB2	-0,508838953	0,352226378
LOC644477	-0,508642165	0,077484761
GPNMB	-0,508067878	0,650152069
NOL1	-0,508035725	0,821761655
C1orf95	-0,507922042	0,885191885
RNF222	-0,507853716	0,027917952
hCG_1985469	-0,507701659	0,826064973
LOC729867	-0,507540361	0,004466769
TUBGCP2	-0,507388222	0,965410389
OR8G1	-0,507281654	0,1032926
KLRG2	-0,507234314	0,27969394
LOC727894	-0,507185317	0,036173365
MAN2B2	-0,50717832	0,075042346
TP73	-0,507034244	0,970884959
ANKRD16	-0,50700014	0,038286613
RNF41	-0,50699741	0,2943174
CPSF5	-0,50695798	0,257966329
GCNT3	-0,506922605	0,189137222
C2orf28	-0,506904531	0,670189401
LOC653375	-0,506633708	0,20942377

RAC3	-0,506531948	0,105560074
TMEM150	-0,506481727	0,819365694
KCNK1	-0,50625337	0,051946136
MIXL1	-0,506150787	0,816925698
PHIP	-0,505981165	0,048050749
LOC646658	-0,505980194	0,899499692
TTBK2	-0,505896231	0,269456441
FBXL11	-0,505534236	0,909111572
MTERF	-0,505497953	0,066895773
LOC344332	-0,505176617	0,753840611
LOC388820	-0,505108549	0,022499718
CD79B	-0,504806796	0,207119287
LOC642837	-0,504789316	0,071525794
C20orf123	-0,50456031	0,215716366
LOC727944	-0,504361454	0,173232925
TPSG1	-0,504024613	0,420510656
FLJ36870	-0,503657575	0,053321509
HIF1AN	-0,503478967	0,891347126
LOC731000	-0,503302236	0,655730867
TNNI1	-0,503249841	0,887413113
TSR2	-0,503150246	0,032812506
C13orf31	-0,503119621	0,01148718
FAM110A	-0,502941809	0,381842043
PCK1	-0,502903056	0,026388042
GALNT2	-0,50288825	0,197258641
GIT1	-0,502805412	0,771093907
FLJ38576	-0,502716952	0,041984564
IMPA2	-0,502626066	0,153021859
LOC729862	-0,50248064	0,023449259
ZNF350	-0,502341157	0,360979434
AMOTL1	-0,502324755	0,499439703
hCG_1817208	-0,502267167	0,668285031
DGKQ	-0,502237484	0,445827071
SUV420H2	-0,502113851	0,085730399
ZBTB8	-0,501813025	0,162219899
LOC442710	-0,501792082	0,22636823
ZFP36L2	-0,501743742	0,122509456
ARRDC5	-0,500995809	0,512502658
TPPP3	-0,500965149	0,041386502
IQGAP1	-0,50093347	0,097990636
TM6SF2	-0,500806556	0,687966774
ATP1B4	-0,500594078	0,221604182
RPS26P10	-0,500465902	NA
C10orf111	-0,500440671	0,717362633
LBA1	-0,500426225	0,185758616

SLC7A14	-0,500398252	0,367510842
MLC1SA	-0,500382069	0,052980976
OR10T2	-0,500344009	0,023938924
COX6C	-0,500339659	0,056641765
FLJ10581	-0,50005498	0,785708165
LOC644075	-0,499930632	0,053338234
MKRN1	-0,499862349	0,642313642
SCN10A	-0,499768637	0,475374823
TRIML2	-0,499644409	0,227079996
PRCC	-0,499209706	0,05171211
LOC727841	-0,499194531	0,05358942
DKFZp313A2432	-0,498948974	0,815475456
JCLN	-0,498917131	0,292319026
LOC374920	-0,498786147	0,142792352
ASCL4	-0,498688999	0,423952992
FCER1A	-0,498359113	0,467569723
SMPX	-0,498162447	0,183797463
HMGN1	-0,498161665	0,404077439
FLJ46189	-0,497913034	0,238448998
LOC728150	-0,497880359	0,050395712
LOC729964	-0,497825841	0,607264622
PTPRO	-0,497641955	0,929686704
LOC729569	-0,497589027	0,048211529
LOC439949	-0,497508019	0,059870992
PSMC3	-0,497442952	0,585533336
RINT1	-0,497301539	0,925047272
GNG4	-0,496953295	0,079736156
ATP5D	-0,496814912	0,271074871
DDIT4	-0,496794021	0,686563155
MAML1	-0,496792938	0,446097792
MEIG1	-0,496694371	0,281047581
PPP2R5C	-0,496373582	0,056828165
TMIE	-0,496341563	0,418409354
MART2	-0,495949203	0,346227052
APEX2	-0,495928466	0,543929413
BRAP	-0,495880177	0,202982419
KIF16B	-0,495827958	0,409845096
WDR55	-0,495576903	0,155389999
RAD1	-0,495555386	0,76468145
C6orf189	-0,49552266	0,077374685
TSPAN19	-0,495114275	0,030538281
LOC644592	-0,495045675	0,777090423
MGC14560	-0,494951268	0,509989875
LOC339192	-0,494943539	0,060647937
CHN2	-0,494847455	0,018993357

MDH2	-0,494836758	0,930830193
LOC643916	-0,494617472	0,969965643
ZFP1	-0,494432287	0,375094656
LOC645966	-0,494252428	0,190981267
LOC728242	-0,494250523	0,222105297
LOC729891	-0,494241331	0,012074737
KIAA0390	-0,494111957	0,151838557
KRTAP5-3	-0,494094112	0,405364109
LOC731009	-0,494065757	0,019192941
C1orf220	-0,494011229	0,080584631
MRPS6	-0,493878916	0,278857645
RXRA	-0,493820911	0,275223918
HDAC4	-0,49354071	0,804542671
CENPI	-0,493500275	0,017054636
DDAH2	-0,493265994	0,741419676
FAM14B	-0,493036626	0,283721198
UQCC	-0,492800593	0,545463059
ICOS	-0,492691016	0,72704995
C1orf161	-0,492567469	0,531895745
PXMP4	-0,492446559	0,095423159
NUDT12	-0,49243306	0,814529872
C21orf2	-0,492233998	0,353226853
ATP5J2	-0,492087033	0,145519885
OAT	-0,492062603	0,950271525
SYDE2	-0,491976861	0,372125095
TMIGD2	-0,491713967	0,587995997
C1orf112	-0,491631273	0,070437943
HTR1B	-0,491441315	0,514082776
RNF169	-0,491410906	0,357783205
GJC1	-0,491233083	0,34985935
MYOM3	-0,491223798	0,026372805
OR2AE1	-0,491109979	0,131909571
FMO1	-0,49090889	0,117767031
SERPINA13	-0,490626127	0,668364788
PM20D2	-0,490452104	0,065270827
IHPK3	-0,490411278	0,260075469
GSTO1	-0,490375427	0,271326686
LOC731891	-0,490346626	0,437956861
LOC730118	-0,490309155	0,045894336
FOXK1	-0,490129929	0,120749924
CMA1	-0,490057628	0,117917101
NMI	-0,4900183	0,184996926
C7orf38	-0,490017447	0,049686064
C17orf47	-0,489926682	0,043299594
NKD2	-0,489785181	0,535680106

SCGBL	-0,489727532	0,181193738
LOC644841	-0,489409846	0,674218598
TDO2	-0,488690822	0,100440077
PLIN	-0,488686042	0,763667036
SCD4	-0,488357122	0,071161261
MAN1A1	-0,488182678	0,114879602
LOC729382	-0,487776016	0,20544859
KIAA0562	-0,487742258	0,146700238
ADAMTS9	-0,487703288	0,376484738
LOC646574	-0,487599931	0,184818097
MND1	-0,487589417	0,772382366
LRP8	-0,487321628	0,196947947
POLR3H	-0,487237227	0,039275679
DMGDH	-0,486934676	0,129619097
FLJ37183	-0,486776419	0,210381493
DAOA	-0,48671163	0,038335303
DSCR1L1	-0,486560856	0,082724425
METT5D1	-0,486440142	0,715893264
PTPRQ	-0,486360028	0,238820497
LOC729683	-0,486346315	0,376501862
BZRAP1	-0,48629809	0,035692367
IMMT	-0,486049599	0,340994961
FLJ37228	-0,485349957	0,236565055
OR2T11	-0,4852635	0,550338004
OLFM3	-0,485138495	0,412912821
LOC728867	-0,485095962	0,530018597
ATP13A5	-0,485013359	0,112422845
LOC644816	-0,484791567	0,444511656
DNAJC8	-0,484749595	0,55623095
PPIB	-0,484656186	0,143963201
PIGX	-0,484608605	0,774772206
C1orf107	-0,484288998	0,045644383
LOC647041	-0,484168295	0,172289474
FIS1	-0,484150961	0,033614366
BMP2	-0,483986467	0,152384948
CCDC121	-0,483975069	0,853148036
FLJ32810	-0,483847952	0,265789927
GNB3	-0,48345704	0,921480905
LOC642927	-0,483391677	0,493384505
TMCC2	-0,483185221	0,189593034
CHST2	-0,483120754	0,465133675
VTI1A	-0,48308149	0,182403073
BECN1	-0,483079967	0,022413738
TDRD9	-0,482977431	0,73456139
C1orf198	-0,482837163	0,096228058

ZNF24	-0,482815627	0,38356572
C13orf22	-0,482795163	0,058769867
ERAS	-0,482691997	0,153413332
AXL	-0,482422838	0,008960396
TMEM132C	-0,482403535	0,656746618
PRKAG1	-0,48224231	0,089997647
DMPK	-0,482163787	0,202985812
SLC34A3	-0,481741982	0,024419641
MHC2TA	-0,48167743	0,129939013
WDR19	-0,481631686	0,260117483
ZNF189	-0,481551106	0,383968478
CEP68	-0,481312191	0,15562664
C1orf52	-0,481217373	0,071615199
CHST12	-0,481071999	0,718847662
LOC652045	-0,480809369	0,078233209
TSPAN5	-0,480759539	0,276320425
MYL2	-0,480469716	0,837997999
GGTL3	-0,480459003	0,860929284
ABCA4	-0,480398432	0,996820644
ATBF1	-0,480265558	0,636896588
HLA-DQA2	-0,480251625	0,414402738
FLJ12760	-0,480171906	0,163716867
GALNT9	-0,480088895	0,208471354
IL31	-0,480033964	0,323669033
IGSF21	-0,479946025	0,581298139
LOC646055	-0,479887325	0,27154404
C2orf53	-0,479680321	0,580258335
F2R	-0,479598273	0,340193241
EPHA4	-0,47958954	0,107306804
PML	-0,479463522	0,27150711
SPINK8	-0,479388944	0,465217484
ZNF91	-0,479309688	0,084626672
CWC15	-0,479302452	0,256513127
MPP6	-0,479057547	0,941043571
C1orf55	-0,478910313	0,591216953
ADK	-0,478434253	0,050486103
PTPN21	-0,4782035	0,145285643
ZNF66	-0,478200841	0,162928977
BTBD3	-0,478047852	0,994308209
WDR85	-0,478017448	0,797465383
LOC728290	-0,47791905	0,618937389
TRIM26	-0,477910365	0,088088802
LOC732097	-0,477816185	0,181977264
C9orf163	-0,477771254	0,928511117
DEFB109	-0,477689341	0,999875313

EVI2A	-0,477673924	0,168739431
PIM3	-0,477558205	0,219661095
SOX21	-0,477482374	0,447601769
GALE	-0,477435116	0,926614198
TRUB1	-0,477378314	0,417903718
LOC653053	-0,477087395	0,070825388
RLBP1L2	-0,476797191	0,102958115
C1orf189	-0,476400526	0,777462885
LOC646051	-0,476387845	0,664940988
ISY1	-0,476348654	NA
RNF137	-0,476298519	0,128265453
VHL	-0,476232431	0,231921525
GIGYF2	-0,475989047	0,068659693
CART1	-0,47558003	0,459653571
ATOX1	-0,475482361	0,394168207
PER3	-0,475304652	0,092686082
IL6	-0,475247969	0,062146826
LOC646104	-0,475229609	0,636595327
LOC652436	-0,475112204	0,079663721
LOC730144	-0,475077217	0,991227838
NDRG3	-0,475004121	0,125949415
SLC45A1	-0,474741901	0,105095942
BHMT	-0,474684928	0,828162427
GNA13	-0,474395994	0,967786959
DPYD	-0,474303836	0,321675503
OR10A3	-0,474196591	0,193620368
MKS1	-0,474091789	0,359871078
SEPW1	-0,474011737	0,037273402
DSG2	-0,47397628	0,006682744
LOC642637	-0,473949747	0,17887342
LOC644979	-0,473502648	0,116706683
GNAO1	-0,473340838	0,093807006
NFIA	-0,473233031	0,023359625
SEMA3A	-0,473192467	0,200795321
NOTCH1	-0,473096556	0,984941958
OK/SW-cl,56	-0,472669614	0,044114744
NRP2	-0,472467922	0,21357779
POU2F3	-0,472455422	0,202958668
LPAAT-e	-0,47239177	0,208537147
C21orf66	-0,47177155	0,237816758
FAM102B	-0,471768981	0,011085305
ST5	-0,47134373	0,126987018
LOC652586	-0,471339227	0,064316878
LOC727731	-0,471123186	0,279814173
C10orf32	-0,47059984	0,097258042

BIRC1	-0,470349085	0,116566398
PCDHGA7	-0,469999028	0,285892867
TPSB2	-0,469930224	0,319184424
SNTB2	-0,469551632	0,106093737
CCDC54	-0,46933305	0,084774501
ARHGEF10L	-0,469243315	0,318022732
FHAD1	-0,468952772	0,924928507
KCNN2	-0,468924409	0,645497046
RAB11A	-0,46888402	0,32005437
TRIM16	-0,468855994	0,138536676
ZZZ3	-0,468482327	0,07958886
MLANA	-0,468285966	0,986915137
TRAR3	-0,468248985	0,512401766
NSMAF	-0,467881246	0,334078935
ARF6	-0,46772468	0,545003679
OR2M4	-0,467598731	0,152441246
hCG_27695	-0,467434354	NA
ATRN	-0,467300651	0,025550696
GPR15	-0,467267979	0,440667269
SIPA1L2	-0,467173089	0,406685937
DENND2A	-0,466752818	0,091731038
LOC341346	-0,466581865	0,07055726
LOC730891	-0,466460812	0,388951735
ACTR3	-0,466342868	0,135549491
TIMM8A	-0,466081155	0,867351508
LOC653226	-0,465845847	0,177856138
HPGD	-0,465571411	0,763821006
CAPN8	-0,465563406	0,022872728
FCER2	-0,465157567	0,25230633
NDFIP1	-0,464834922	0,189184332
NFX1	-0,464646778	0,318339743
C14orf118	-0,464347845	0,215581538
KIF20A	-0,464341086	0,264213784
CLN3	-0,46417271	0,025265513
SV2B	-0,463882066	0,294249628
LAPTM4A	-0,463879909	0,115871183
INHA	-0,463520067	0,187815083
C20orf17	-0,463476389	0,682590436
KPNB1	-0,463443635	0,213993383
NSFL1C	-0,463034079	0,659207232
FAM133B	-0,462989841	0,720874865
C6orf138	-0,462909053	0,100681904
NLN	-0,462808613	0,213691068
CRIP1	-0,462783833	0,024140634
C19orf63	-0,462753579	0,13316512

MT1B	-0,462717784	0,429845937
MDH1B	-0,462615293	0,094464033
TMEM144	-0,462480507	0,452783423
C7orf45	-0,462443997	0,239861483
LRRC8D	-0,462438537	0,557482783
GPC1	-0,462351539	0,006770446
CTR9	-0,462331876	0,674045118
LOC348751	-0,46229566	0,303388504
SLC44A2	-0,462171972	0,016838654
SATL1	-0,462066061	0,137608823
CYBB	-0,461996148	0,289710981
KLHDC9	-0,46175409	0,085892295
GRIK2	-0,461747204	0,102437811
ZNF76	-0,461717928	0,221563905
LOC729664	-0,461677404	0,115265685
ATXN2L	-0,461359939	0,096712697
PPFIA1	-0,461290677	0,112140304
ITPR1	-0,461278009	0,658496583
LOC644196	-0,461166962	0,522957777
C10orf92	-0,460986716	0,682529471
LOC729928	-0,460728252	0,753556492
LOC644732	-0,460654359	0,084150449
MEGF10	-0,460481837	0,164837316
LOC730496	-0,460470225	0,622103562
ACTL6B	-0,460449789	0,804008693
MANEAL	-0,460054755	0,447306855
NBR1	-0,460049344	0,458703575
TRAF3IP2	-0,459991375	0,061650456
C12orf65	-0,459911815	0,000474038
KBTBD5	-0,459794201	0,640868995
TAX1BP3	-0,459626094	0,052217559
POLR2F	-0,459476155	0,442523887
RAB8B	-0,459457103	0,067834568
FLJ38723	-0,459389799	0,489811177
PPP1R8	-0,459305705	0,034297058
KCND1	-0,459256826	0,850456015
ELF4	-0,45895499	0,177006203
FXYD2	-0,458608266	0,170719484
CDON	-0,458603823	0,093130692
NEK9	-0,458520953	0,221402695
SLC4A1AP	-0,458460833	0,041150126
HCN2	-0,458141809	0,135756384
CFTR	-0,458097751	0,008610155
GOLPH3L	-0,457756969	0,16603818
LASS6	-0,457698567	0,264972101

PPCDC	-0,457696054	0,26019915
XKRX	-0,457518011	0,555882747
GADD45A	-0,457513851	0,446534314
ETHE1	-0,457214898	0,037821713
FAM83H	-0,45716137	0,328025135
SPTBN2	-0,457095633	0,586333092
LOC440456	-0,456987922	0,422246655
LOC440348	-0,456909286	0,697538616
UBQLNL	-0,456766577	0,048215675
TMEM56	-0,456653727	0,629035728
FDXR	-0,4565917	0,247579533
PIWIL4	-0,456571591	0,148233069
LOC732087	-0,456226425	0,121206172
C10orf58	-0,456220809	0,079994115
OPCML	-0,456075587	0,493511959
FGL1	-0,455711524	0,928752875
LOC730130	-0,455702547	0,097060685
LOC729507	-0,455701016	0,225573207
LOC642563	-0,455644886	0,946005626
LOC113655	-0,455246389	0,153844916
C11orf65	-0,455225707	0,459327479
NFAM1	-0,455006784	0,104265941
IFNA1	-0,454920201	0,202720998
HEXIM1	-0,454919258	0,072922903
LOC729720	-0,454748963	0,088634583
LOC731294	-0,454721649	0,231995962
SOHLH1	-0,454335315	0,257513194
LOC646604	-0,454320786	0,388098085
PYGL	-0,454318462	0,470788743
TNFRSF25	-0,453967999	0,867154935
PAK2	-0,45395902	0,766829389
GGN	-0,453915741	0,64108781
APOA1	-0,453846629	0,689771136
LOC729729	-0,453794491	0,172799238
UROC1	-0,453722667	0,212267859
HECTD2	-0,453689184	0,831208988
GSR	-0,453089951	0,546107763
ATAD3A	-0,452969582	0,52721689
WFDC9	-0,452916481	0,079926777
PAP2D	-0,452893377	0,510140405
CNNM3	-0,452791393	0,082954678
C6orf162	-0,45261908	0,087128399
CMAS	-0,452495755	0,904140886
H2AFB2	-0,452447391	0,85944143
POLG2	-0,452325172	0,653556345

TRA1	-0,452224575	0,611446243
GUSBP1	-0,451903067	0,536018702
APRT	-0,451876119	0,038771039
CYB5D1	-0,451483018	0,073253806
DAP13	-0,451466539	0,092063726
APOA2	-0,451460354	0,138787461
PAPPA	-0,451279494	0,358179232
LOC730333	-0,451249641	0,840011856
OR4F15	-0,451213975	0,28576591
OR2J2	-0,451188302	0,037066548
FLJ23598	-0,450825829	0,265536768
TMEM48	-0,45080489	0,01863888
SERPINB5	-0,450523875	0,045792752
CTSB	-0,450411053	0,236042436
PLA2G2F	-0,450378679	0,174739199
KRTAP1-1	-0,450279657	0,049900204
LOC731477	-0,450257542	0,054422906
TFAP2A	-0,450124732	0,204841997
MUC5B	-0,450123356	0,233992664
LOC642381	-0,449967441	0,103042253
PSCD4	-0,449927345	0,544932351
KRT6B	-0,449846679	0,217101564
HAT	-0,449570041	0,501639693
LOC729120	-0,449456143	0,756454824
KRTAP23-1	-0,449326941	0,108125459
LOC642740	-0,449007269	0,011741058
C19orf46	-0,448857989	0,006893695
SYN3	-0,448825031	0,115026321
ACOXL	-0,448794694	0,702652052
LARP2	-0,448702336	0,151946793
GPHN	-0,448002069	0,677832142
LOC728514	-0,447924457	0,308878841
LOC729811	-0,447909756	0,445435498
CTNNBIP1	-0,447641211	0,049990333
OTOS	-0,447571387	0,296256713
LOC51058	-0,447514748	0,366693343
HSPB7	-0,447222567	0,865097979
LOC92912	-0,447036451	0,122330635
LOC649305	-0,447027115	0,13814246
LOC729825	-0,447026824	0,181388934
TMED7	-0,447016019	0,017928201
PLA2G2C	-0,446759322	0,751217589
C6orf124	-0,446681907	0,105041878
CAMK1G	-0,446676456	0,273453084
ARRB1	-0,446554977	0,569257192

PRPS2	-0,446473149	0,130902017
RAD51AP2	-0,446335858	0,008546294
THSD7A	-0,446238811	0,067662097
ELOVL4	-0,446192917	0,531582283
LOC728856	-0,446032842	0,026663607
HSPC132	-0,445996725	0,364735332
KRTAP5-8	-0,445868568	0,215210176
CUTA	-0,445852476	0,480668993
GSDM1	-0,445808157	0,733302968
SNRNP48	-0,4457006	0,052050269
PRDM12	-0,445567789	0,032604682
MGAT4A	-0,445497386	0,447088931
KIAA1604	-0,44541545	NA
C19orf22	-0,44522817	0,03881007
ITGA3	-0,445216064	0,845864285
C8orf22	-0,444885522	0,58400818
LOC126860	-0,444663883	0,675865225
GUSB	-0,444457025	0,838248846
FTCD	-0,444437064	0,731813667
EFCAB7	-0,444406257	0,526207569
GABRA3	-0,444247919	0,550596677
CYP26A1	-0,444239696	0,190865835
LAT	-0,443974703	0,443245405
SYAP1	-0,443680286	0,716539654
G0S2	-0,443629646	0,024262825
SIN3A	-0,443597494	0,189035719
LOC729602	-0,443587942	0,01016444
ZNF155	-0,443452639	0,25410822
PTPN2	-0,443443615	0,742398124
EPC1	-0,443279517	0,636820181
EFNA5	-0,443188715	0,456844091
C14orf80	-0,442971481	0,028813521
PRPF38B	-0,442947253	0,146277303
LOC115294	-0,442647862	0,755573561
PPP1R9A	-0,44260575	0,014135676
DDX5	-0,442558707	0,420425868
DPY30	-0,442441243	0,400126058
ADAM21	-0,442408106	0,256782978
DUSP1	-0,442293571	0,483330517
LOC730176	-0,442267894	0,10539347
ZNF302	-0,441846968	0,074006315
LOC150739	-0,441827465	0,013985503
C15orf54	-0,441744531	0,091403765
DHRS8	-0,441686303	0,116412786
LOC220433	-0,441665093	0,679625402

LOC647859	-0,441538165	0,576757809
DIP2B	-0,441414679	0,112714202
RAP2B	-0,441285118	0,099680295
VPS37D	-0,441183144	0,585500357
OR6C75	-0,441182401	0,100592495
CLEC3A	-0,440514544	0,725801036
hCG_1986447	-0,440386171	0,159637232
FHL1	-0,440327508	0,631803466
MFN1	-0,440314245	0,331283034
ALKBH2	-0,440275447	0,055398659
HAX1	-0,439565041	0,095878582
CBY1	-0,439560711	0,633621065
ZNF581	-0,439404574	0,4592708
NBEA	-0,439313699	0,365234375
CCK	-0,439171349	0,392990635
CLPX	-0,439168037	0,082851828
EIF2S3	-0,439118953	0,3605724
QRFP	-0,438931503	0,448477989
LOC729615	-0,438823542	0,052719577
SNRPG	-0,438634365	0,089506471
MTR	-0,438567395	0,206481357
PPP1R12B	-0,438305408	0,317177582
LOC729669	-0,438272126	0,290892414
LOC731416	-0,438160704	0,036698053
TMC4	-0,438133151	0,176918214
CHKB	-0,438094817	0,906061701
OR4C16	-0,438082075	0,768673879
UNC13B	-0,438043836	0,325231539
LOC728440	-0,438035156	0,140421075
ITGBL1	-0,437934055	0,392792541
SMP3	-0,437580436	0,94031068
ABCG1	-0,437192587	0,748385924
LOC729911	-0,437079196	0,836961706
LOC646643	-0,437073339	0,089211167
POU4F3	-0,436966305	0,203320337
KCNS1	-0,436829684	0,154365599
BCL2A1	-0,43678851	0,830495238
ATP7B	-0,436336426	0,253053797
PCDHGA8	-0,435855379	0,301585976
TCEAL6	-0,43550868	0,047146842
GOSR1	-0,435483416	0,123727626
PLCXD1	-0,435049779	0,245697244
TSPYL5	-0,435008858	0,604333159
SRXN1	-0,434862975	0,467475788
GOPC	-0,434639833	0,048510044

KIAA1441	-0,434514181	0,436847947
NANS	-0,434479218	0,019465063
SPRN	-0,434375855	0,262929378
LOC731787	-0,434132189	0,082387101
SLC15A4	-0,434038227	0,227279578
CLCN1	-0,433451054	0,086040667
FBXL17	-0,432731589	0,071590742
HCN3	-0,432579881	0,233095377
MRPS5	-0,432343262	0,322003146
PRTFDC1	-0,432030587	0,145007576
ARL6IP6	-0,431849157	0,651857481
SPHKAP	-0,431614195	0,098080355
GPR141	-0,431473403	0,282629704
PLVAP	-0,431448433	0,148652822
FLJ25778	-0,431311416	0,085948472
GATM	-0,431305456	0,735304136
HOXC13	-0,43130255	0,29124526
E4F1	-0,431202178	0,792395145
SEPSECS	-0,431111314	0,183638534
MANEA	-0,431097299	0,838106155
TMEM105	-0,430956124	0,3625844
BBS1	-0,430804454	0,304061483
ABCB1	-0,430744656	0,124943366
PSCD2	-0,430723145	0,351931904
CD5	-0,430692039	0,295470404
C17orf95	-0,430559679	0,217904025
OPLAH	-0,430527415	0,777777488
LOC90826	-0,430394961	0,569715156
METTL9	-0,430057037	0,200974106
ZFP67	-0,429644382	0,35638973
PLN	-0,429528008	0,61424343
PPP4R4	-0,429419483	0,87210736
LOC646014	-0,429414258	0,059083871
KRTAP24-1	-0,429239648	0,165954361
SUV420H1	-0,429221237	0,043741486
HECW2	-0,429034816	0,479305882
CLDND2	-0,428937373	0,111366496
NOL10	-0,428881158	0,659329863
GTF2H2	-0,42882699	0,455128052
PIH1D2	-0,428511764	0,616069505
SMARCAD1	-0,428410817	0,06371137
PAEP	-0,42838322	0,549785356
WDYHV1	-0,428309132	0,043470787
ZNF264	-0,428183666	0,041823574
LOC728637	-0,428051526	0,213926752

ACTL7A	-0,427693279	0,37560154
LOC200810	-0,427549853	0,086718389
IL34	-0,42747976	0,280034405
PAX5	-0,427373453	0,417057247
TMEM49	-0,427370757	0,457477323
ANG	-0,427363494	0,384729028
LOC646629	-0,427097795	0,107150108
KCNG2	-0,427037024	0,696646132
C4orf34	-0,427028527	0,923001989
UGT1A4	-0,426864318	0,154080767
ALDH1A3	-0,426863499	0,219389761
NAV3	-0,426838396	0,065101482
TMEM87A	-0,426822335	0,088631109
KIAA1310	-0,426629657	0,456961827
LOC646754	-0,426358227	0,306954052
LOC641515	-0,425943214	0,880278738
NUP155	-0,425840345	0,198207641
SLC17A4	-0,425776023	0,118573563
LOC731212	-0,425668775	0,338300301
CLUAP1	-0,425648264	0,159121979
HAK	-0,425609463	0,102839411
KIAA0478	-0,425574139	0,797237232
LOC729791	-0,425514302	0,304719805
SOST	-0,425482602	0,128475314
C16orf75	-0,425075268	0,055425904
LOC732390	-0,425043054	0,08078286
BOLA3	-0,424769892	0,193820122
SFT2D2	-0,42476867	0,661472913
PRSS7	-0,424519771	0,364821456
DMXL2	-0,424460092	0,083625353
CCDC123	-0,424170382	0,089052847
VNN2	-0,424114366	0,450103726
CENTB1	-0,424022049	0,22232003
CEP110	-0,423905397	0,359992714
SLC4A3	-0,423609387	0,09720095
C9orf78	-0,423588565	0,504279246
SF3B3	-0,42341491	0,347132225
DMBX1	-0,423313379	0,080030711
APOBEC3C	-0,423153325	0,083210873
XKRY	-0,422624438	0,299852788
HCRT	-0,422542416	0,846528908
ATP5I	-0,422314947	0,726048492
LOC57228	-0,422251944	0,946943997
LYPLA1	-0,422230667	0,7767335
CPT2	-0,422158592	0,044277369

KIAA1394	-0,421948759	0,868795528
CNTLN	-0,421948529	0,542902471
APOC2	-0,421821531	0,943367875
HCG27	-0,421714012	0,022162688
LOC729129	-0,421506118	0,726923336
PRR17	-0,421428966	0,424010645
ACLY	-0,421189544	0,891617922
ATP2B1	-0,421125425	0,455307306
RRAGA	-0,421095391	0,075151839
LOC729107	-0,421055785	0,058329395
OR8B2	-0,421021209	0,101352986
MIZF	-0,420766711	0,527729112
ZNF500	-0,420631162	0,513275431
ZBTB33	-0,420174127	0,190073731
LOC730987	-0,420112986	0,195688771
OCLM	-0,419975594	0,094598784
EPB41L4A	-0,419683362	0,495834745
SHF	-0,419506396	0,306620762
GAPVD1	-0,419402232	0,546967843
ADAM9	-0,419108713	0,162818804
DET1	-0,419092241	0,814006624
LOC642026	-0,419089572	0,45527075
C6orf224	-0,419003853	0,866776859
LOC729598	-0,418649032	0,953206753
MTX3	-0,418420263	0,049424446
KLHL33	-0,418366345	0,90242507
TM4SF18	-0,41834329	0,836338868
KIAA0020	-0,418321916	0,584928792
IFNGR2	-0,417963029	0,124219076
STEAP2	-0,417730022	0,146809586
C14orf112	-0,417690117	0,581550326
NSEP1	-0,417597987	0,288622276
EGLN1	-0,417437609	0,344782648
FAM5C	-0,417408182	0,077128363
PYCR2	-0,417084549	0,296260067
OR2T12	-0,416998045	0,202090636
Prostein	-0,416776739	0,736409207
S100A12	-0,416766714	0,018677776
APITD1	-0,416614411	0,282216951
MS4A10	-0,416546172	0,095618846
SALPR	-0,416378657	0,176983544
LOC730190	-0,416201701	0,010520542
HHLA2	-0,416009457	0,017347638
TPM2	-0,415877333	0,059279209
ZNF80	-0,41575762	0,173422512

ZADH1	-0,415726822	0,578281426
TTLL13	-0,415709277	0,154414628
EDG2	-0,415569583	0,822192428
SHANK3	-0,415536774	0,087098227
KIAA0355	-0,415449142	0,921247944
LOC729704	-0,415342538	0,140681475
CHD1	-0,415246818	0,079592481
TM2D2	-0,415224504	0,136870271
KIAA0513	-0,414924819	0,357462283
TDRD7	-0,41484317	0,104390894
LOC155054	-0,414841577	0,183689646
C14orf48	-0,414770275	0,844068822
AEBP1	-0,414420917	0,142815157
C2orf66	-0,414240101	0,101378383
RPS8	-0,414087839	0,556325349
ESAM	-0,414036303	0,343184073
OR51E2	-0,413632248	0,872836944
PARN	-0,413612297	0,855609957
CCDC128	-0,413351746	0,063601239
LOC729026	-0,413320632	0,058912247
OR10J1	-0,413062666	0,191939349
SERPINB6	-0,412984309	0,464381228
ADSS	-0,412946216	0,176870802
LOC402509	-0,412756443	0,111141682
LOC652698	-0,412648427	0,780063237
TLCD1	-0,412356351	0,251850516
SMYD3	-0,412265415	0,277818446
GRIN2D	-0,412247468	0,45699256
TSNAXIP1	-0,412095115	0,380373883
PDGFA	-0,412056579	0,602413602
CCDC113	-0,41193137	0,028067661
PDE3B	-0,411847165	0,360135353
C1orf69	-0,411739159	0,257099345
LOC652203	-0,411726965	0,113643348
PCDHA3	-0,411661392	0,209085899
AMID	-0,411632141	0,183333493
CSN2	-0,411595925	0,082469213
IER3	-0,411595334	0,869878027
ZAK	-0,411536096	0,418304148
CDC27	-0,41144002	0,038997833
LIN7A	-0,411316264	0,184807804
OXCT2	-0,411259365	0,681104507
PLTP	-0,411176854	0,694729873
IL6ST	-0,410636381	0,44166571
SIAT4A	-0,410589855	0,056648557

PDE6B	-0,410492914	0,438717646
LZTS2	-0,410458915	0,018040677
IL2RG	-0,410353185	0,101350845
RICS	-0,410186351	0,783171796
DTNB	-0,410064475	0,718479936
LOC401923	-0,40945507	0,728699989
LOC645822	-0,409453574	0,083917579
RUVBL2	-0,409387813	0,102403142
LOC399744	-0,409285956	0,553609699
MAGEC3	-0,409170622	0,988043115
LOC729793	-0,408839999	0,156102591
PCDH20	-0,408731629	0,905913918
GRPEL1	-0,408577068	0,031121876
RNF144	-0,408556124	0,864907595
CRYBA4	-0,408437578	0,884967475
CXorf57	-0,40833975	0,034976582
TRIM17	-0,408338842	0,125271444
LOC643783	-0,408134611	0,27235214
DNTT	-0,407912273	0,143484147
GTF3C4	-0,407838674	0,421182037
SLC30A3	-0,40783774	0,028169777
HP1BP3	-0,407774739	0,32850339
DKFZp564N2472	-0,407687943	0,079814621
GLT25D1	-0,407564114	0,149307377
GPR3	-0,40754317	0,12577535
RNASEK	-0,40738228	0,156796174
MARVELD2	-0,406819574	0,139649903
WWC3	-0,406670834	0,38506137
PGA5	-0,406562904	0,267560742
LOC650943	-0,406517363	0,046245509
FAM50B	-0,406438673	0,164243101
FRYL	-0,406254801	0,064523288
PPP5C	-0,406249823	0,441457163
CAPN7	-0,406247739	0,075005489
LOC732272	-0,406208786	0,182734688
SDHB	-0,406079142	0,350005412
OR3A1	-0,405918784	0,947700091
CES4	-0,405531252	0,623739179
NPC1	-0,405492601	0,353139459
BAI1	-0,405487441	0,083430611
PDCL	-0,405426408	0,169278246
C22orf23	-0,405238273	0,397320929
MTMR2	-0,404949184	0,097598165
SLC22A3	-0,404899451	0,08856284
MIR	-0,404651144	0,403871811

C5orf15	-0,404395477	0,609304771
CPT1A	-0,404168869	0,233828032
CISD1	-0,404077369	0,073411642
TETRAN	-0,404056765	0,596651318
PAQR4	-0,403958966	0,998049291
CLDN22	-0,403941547	0,081034172
EIF3M	-0,403890014	0,109957031
EGFR	-0,403889155	0,630062893
ELAC1	-0,403576952	0,326809019
LOC121838	-0,40352001	0,206041315
ZNF544	-0,403504824	0,311874986
MAGEB5	-0,40326472	0,757099018
EEF2	-0,403249515	0,648263729
KIF14	-0,40300648	0,583266658
TNRC6A	-0,402993068	0,800127812
FXYD1	-0,402847332	0,446902189
LOC148766	-0,402733281	0,374985128
USP34	-0,402548393	0,763434933
HOXA11	-0,402412401	0,086101762
LOC730129	-0,402395385	0,093172907
EIF3S2	-0,402330086	0,15064477
CYP4F12	-0,402193918	0,069190911
GABRG3	-0,402031051	0,097107293
LOC402457	-0,401979228	0,105234285
GK2	-0,401940913	0,160063866
BAMBI	-0,401732982	0,632538693
LOC647212	-0,401722706	0,603662761
HERC2	-0,401705787	0,317807035
LOC441061	-0,401681996	0,099968689
SN	-0,401651168	0,914697562
RPL22	-0,401592915	0,090464748
DDA1	-0,401509977	0,899331262
15.09.2010	-0,401479944	0,028375159
SLFN14	-0,401445274	0,047875542
ZA20D2	-0,401246326	0,824765396
LOC730187	-0,401023794	0,682346605
WFDC6	-0,401010768	0,081743659
HOXB7	-0,400825393	0,106610768
SNX25	-0,400817232	0,086646158
AVPR2	-0,400768367	0,090957637
LOC123103	-0,400564885	0,07011026
LOC440956	-0,400482342	0,057112214
CNN1	-0,400470844	0,548033984
DUSP22	-0,40041094	0,649900149
METAP2	-0,40031973	0,331605057

LOC729792	-0,400318033	0,045424864
GRRP1	-0,40026766	0,229694047
CPNE2	-0,400103906	0,317139975
LOC728677	-0,399982288	0,579267037
FBXO6	-0,399813153	0,19309993
C14orf123	-0,399784849	0,232756356
GPR88	-0,399782674	0,953735825
FAM55D	-0,399733571	0,558038172
LIPI	-0,39964425	0,0973511
CCT5	-0,399575305	0,03606059
BRPF3	-0,399440209	0,622874608
WRNIP1	-0,399401063	0,309403381
TIAM1	-0,399337788	0,594186014
SYNGR4	-0,399313651	0,046638603
LOC441864	-0,399296838	0,045976305
LOC340571	-0,39926108	0,542678142
KLRC4	-0,399168227	0,064303708
PMS2L9	-0,399143625	0,200313734
SELO	-0,3989307	0,652659187
PTGS1	-0,398887611	0,242897092
USP7	-0,398702072	0,09090978
CDK5R2	-0,3986858	0,182587996
SURB7	-0,398610148	0,053415438
ARGFX	-0,398408959	0,341670863
LOC651441	-0,398406162	0,133968806
EEF1G	-0,398369149	0,266026372
UHRF2	-0,39820411	0,134619456
PTGES2	-0,398200121	0,832861839
FSCN3	-0,39818911	0,612468978
PIN1	-0,397467452	0,461008539
SLC17A8	-0,397379263	0,109595894
PPP2R2C	-0,397359683	0,779392699
TUBG1	-0,397222184	0,623013639
PCDHB10	-0,396954693	0,438541674
ABCC6	-0,396761985	0,809466719
PKP2	-0,396301348	0,19286797
FRG2B	-0,395893451	0,194736442
PCSK1	-0,395890039	0,561261749
CCND2	-0,395771173	0,564236508
LOC653381	-0,395655207	0,224944642
LOC728316	-0,395618424	0,777472737
GALGT2	-0,395547621	0,205646918
KLHDC8A	-0,395385894	0,031288057
TMEM81	-0,395347503	0,150434477
NOSTRIN	-0,39526116	0,084536109

SLC9A3	-0,395109823	0,127541628
DKFZp761G058	-0,394955757	0,624464567
PRSS15	-0,394861677	0,732255882
SNX1	-0,394749395	0,712329716
PGLYRP2	-0,394727903	0,545987853
LMOD2	-0,394557404	0,080075589
RAB4A	-0,394488175	0,363890446
RPA3	-0,394381762	0,066452866
RAB11B	-0,394032541	0,093447733
CLEC4E	-0,393561702	0,112154355
PTER	-0,393517705	0,382054488
DRP2	-0,393318674	0,313337689
C19orf54	-0,393302158	0,545225737
FLJ35946	-0,392950622	0,961584715
LOC729265	-0,392752703	0,867426402
LSS	-0,392195849	0,496446707
SIRT2	-0,392178435	0,254054338
LOC154907	-0,392124791	0,110481826
TTN	-0,392116728	0,138992308
GPR64	-0,392058405	0,013244873
C9orf9	-0,39205444	0,113659781
TOPBP1	-0,391848469	0,965008144
C15orf38	-0,391673427	0,821959587
MRO	-0,391672982	0,125102208
FGF21	-0,391610074	0,532999512
RBM33	-0,391558708	0,109372352
MRPL34	-0,391528659	0,619367912
ZNF322A	-0,39125356	0,946285253
LOC728056	-0,391208415	0,013468404
IPO13	-0,391126283	0,147303014
FLJ38482	-0,391022644	0,914179846
TAF1L	-0,390963616	0,750484539
ZNF283	-0,390950291	0,861255797
C9	-0,390861358	0,020985145
CCNE2	-0,390756197	0,842654418
YPEL4	-0,390612243	0,066041185
CORO7	-0,390602001	0,051302271
FAM86C	-0,390583979	0,431692473
LOC728965	-0,39048538	0,476329114
USP44	-0,39029612	0,25533811
SLMO1	-0,390206577	0,43700897
ELF5	-0,390174344	0,740946903
EPSTI1	-0,390129378	0,505476319
ENTPD6	-0,390051603	0,304862255
CYP4V2	-0,390043019	0,123983159

LOC390940	-0,389808459	0,394957532
CCL4	-0,389805293	0,504800917
FAM131C	-0,389397131	0,123107294
LOC652554	-0,389322949	0,194410776
LOC390748	-0,389251388	0,079573664
IL1RL1	-0,389060883	0,140961747
DENND5A	-0,389040192	0,593956094
SFN	-0,388926476	0,970523497
GUCY1A2	-0,388905784	0,173936273
LOC732444	-0,38880025	0,136406729
RGS19	-0,388723194	0,628705406
ZCCHC7	-0,388702231	0,527639657
FAM24B	-0,388422979	0,125377753
APOA1BP	-0,388243936	0,289501275
RCN2	-0,388186969	0,504905983
LOC163223	-0,388153114	0,906489734
SLC25A3	-0,388146394	0,400638701
OR2F1	-0,388144362	0,026249304
PLLP	-0,387982006	0,249319197
MEP1B	-0,387955015	0,067858563
BTD	-0,387954921	0,818562633
RNPS1	-0,387820595	0,250837041
JARID1D	-0,387781552	0,394117407
LOC729784	-0,387712091	0,214855967
THRAP4	-0,387550791	0,094156601
DDX58	-0,387275513	0,20326716
FAM90A20	-0,387173689	0,679488684
PANK3	-0,386702404	0,135552489
DMD	-0,386363762	0,128841478
LOC126075	-0,386339925	0,107554376
LOC727758	-0,386330696	0,765983759
MTNR1A	-0,386223118	0,664892619
BRI3	-0,386183408	0,133696379
GIMAP5	-0,386107027	0,494048639
PGAM1	-0,385994939	0,399116165
KCNJ12	-0,385978597	0,984458887
ACE2	-0,385853462	0,082888052
GFPT2	-0,385715074	0,957861326
ANXA7	-0,385651838	0,913586049
CD47	-0,385625013	0,052315597
ICHTHYIN	-0,385442313	0,202088513
APPL	-0,385420931	0,007847836
SCAMP3	-0,385076057	0,088567167
MSL-1	-0,385048072	0,270035213
C20orf4	-0,384843258	0,079404323

STIP1	-0,38481774	0,939387168
C17orf45	-0,384744711	0,572305845
RNF19B	-0,384571204	0,112415974
LOC652531	-0,384549194	0,092082834
VMD2L3	-0,384475972	0,529004182
PCSK9	-0,384450218	0,723558856
LOC729612	-0,384398615	0,570438127
POU6F2	-0,384282664	0,087032119
SLC7A7	-0,384166	0,310520412
UNG2	-0,384151069	0,190280321
LOC731848	-0,384113993	0,244929694
LOC729006	-0,384071194	0,1615228
ABHD3	-0,384035615	0,364784623
LOC729956	-0,384003139	0,318791038
OR2B6	-0,38380928	0,323143957
LOC440737	-0,383790678	0,55109268
GPRC6A	-0,383733924	0,19116296
MRGPRF	-0,383722269	0,975686108
PLA2G10	-0,38366523	0,733341968
ZNF576	-0,383653632	0,78914767
NAP1L4	-0,383498708	0,555620139
RIMS4	-0,383467389	0,343595848
SCYL1BP1	-0,383334694	0,710733569
ABCG4	-0,383190384	0,969807277
NOL4	-0,383123387	0,238701683
KIAA0551	-0,383115317	0,280601718
VAMP5	-0,382974929	0,593237677
SUHW4	-0,382938647	0,999846921
BTBD16	-0,382842437	0,623870769
ZRF1	-0,38277148	0,091257793
WDR66	-0,382679454	0,523823739
LOC347475	-0,38257549	0,204677732
GAD2	-0,382522577	0,158506664
GLRX	-0,382490758	0,299071847
MPP4	-0,382483896	0,144378142
LOC728744	-0,382357272	0,535231523
LCAT	-0,382304812	0,316610338
CCNB1	-0,382221373	0,360162783
SMARCC2	-0,381971664	0,297556008
CPE	-0,381678669	0,697902776
DPP10	-0,381594583	0,355473771
CNR1	-0,381590968	0,089870116
UGT1A1	-0,381549198	0,525534603
ARP3BETA	-0,381454805	0,218308804
SNX16	-0,381431469	0,074825606

HSN2	-0,381239433	0,688221138
GBP2	-0,381174929	0,102854684
TMED5	-0,381166248	0,426407444
PLCB3	-0,381101578	0,218545706
SSX3	-0,381081709	0,860954607
FLJ42289	-0,381062645	0,423293435
SERPINB4	-0,381000231	0,966231559
MICALL2	-0,380529083	0,668382627
LOC729900	-0,380421431	0,213838127
ABI3	-0,380160477	0,445268588
LOC732449	-0,380137049	0,248355828
S100A3	-0,380127924	0,08399701
LOC652049	-0,37994947	0,999395498
CACNG5	-0,379573831	0,208216742
GRAMD2	-0,379564177	0,432098098
C1orf156	-0,379521236	0,143298552
C18orf8	-0,379505337	0,079271628
FAM12A	-0,379456418	0,222494886
LOC730744	-0,379399881	0,589299416
LOC651951	-0,379252742	0,134623879
LOC643179	-0,378982208	0,059081613
LOC199800	-0,378961917	0,152928628
LOC642727	-0,378723575	0,805778267
DEFB115	-0,378694213	0,005555404
RIC3	-0,378631312	0,015170218
LOC646442	-0,378553981	0,163315171
WDSOF1	-0,378535819	0,077239521
CHRNB3	-0,378409623	0,633091154
RBJ	-0,378333989	0,388307658
LOC728661	-0,378320063	0,08382613
ACN9	-0,37830047	0,177263118
KLK14	-0,378162645	0,039770513
ARL4A	-0,378160906	0,622883211
FBLN2	-0,378136071	0,245709717
PPP2R5A	-0,378124492	0,074310713
SUGT1	-0,378120643	0,211936532
GLTSCR2	-0,378039448	0,225647849
SPRR3	-0,377975801	0,315175352
SLC1A2	-0,37796611	0,548826375
MAPK13	-0,377837065	0,790440521
PRR15	-0,377825904	0,961455266
BAT3	-0,377630953	0,541068841
LCE1C	-0,377526386	0,842941056
ENTHD1	-0,377214786	0,628926089
LOC728800	-0,377198204	0,0074209

PRPF40B	-0,377074138	0,12527622
LOC652502	-0,377034908	0,08370948
LOC651738	-0,376938289	0,158621669
SMTNL2	-0,37689903	0,028961524
LOC255411	-0,376752733	0,596222451
CST9	-0,376735076	0,068367184
ARRDC2	-0,37673172	0,263891414
PAPSS2	-0,376691998	0,160743926
BASP1	-0,376596633	0,717957477
WDR87	-0,376485631	0,610179142
FLT3	-0,376368755	0,362215858
TMEM145	-0,376210286	0,958512763
LOC647087	-0,376078036	0,742583278
NOP5/NOP58	-0,375840643	0,337787411
PNPLA5	-0,375829455	0,826231487
LOC644893	-0,375789563	0,096622964
YIPF4	-0,375645438	0,113988747
ECG2	-0,375405711	0,521034512
FIBL-6	-0,3753369	0,038950641
LOC728497	-0,375290543	0,045030852
LOC729257	-0,375240748	0,721728502
LOC440829	-0,375236278	0,992462405
C21orf33	-0,374974741	0,00218259
ZNF202	-0,374786977	0,201304801
MGC34132	-0,374681868	0,755567911
SELE	-0,374670491	0,103816217
P2RX7	-0,374639283	0,189998223
RP9	-0,374582709	0,830507574
IRAK1BP1	-0,374443483	0,100854052
FAM139A	-0,374361945	0,882051902
FLYWCH2	-0,37424665	0,507397002
GABRB2	-0,374207937	0,300975926
C6orf133	-0,374130595	0,076923843
BSDC1	-0,374063815	0,097427413
PHTF2	-0,373971495	0,11288204
KIAA1447	-0,373921092	0,880863947
ZNF382	-0,373843562	0,247602243
ZNF174	-0,373827871	0,217085564
LOC642031	-0,373587882	0,228550184
TRPM8	-0,373553088	0,007680745
CDC42EP2	-0,37347735	0,129026292
KIAA0406	-0,373461883	0,738392519
NCAPH	-0,37342812	0,825281083
VSIG2	-0,373368115	0,141560343
LOC641767	-0,373151856	0,374157234

C18orf55	-0,372997118	0,458034995
PURA	-0,372977565	0,784968301
LOC401089	-0,372926835	0,191944229
FAM54B	-0,372763806	0,396214221
LOC731914	-0,372738877	0,085004346
TDRD1	-0,372725315	0,057917745
CA3	-0,372718833	0,091543808
SLC6A11	-0,372537605	0,181799573
FLJ21075	-0,37250021	0,063839853
DKK3	-0,372489475	0,310111296
FAM108C1	-0,372408512	0,912748742
DCDC1	-0,372403936	0,017028731
SLC5A10	-0,372388811	0,237077929
TRPC3	-0,372380853	0,068972634
STUB1	-0,372348122	0,996756078
ALS2CR4	-0,372236299	0,121777196
AGPAT4	-0,37219913	0,57641179
DCP2	-0,372108597	0,047563236
EPHA1	-0,372025488	0,138479016
TERF2IP	-0,371982246	0,031782146
GOLGA8G	-0,371823467	0,36643851
CCDC23	-0,371681263	0,700819275
KLK4	-0,371441341	0,719256301
LOC642219	-0,371435307	0,271481433
ORM1	-0,371341412	0,358370124
IL7R	-0,371269023	0,31300579
LOC730060	-0,371174215	0,994680477
ANTXR1	-0,371080939	0,500619866
LOC441019	-0,371048681	0,712636782
OTUD6A	-0,371034176	0,746968812
LOC647190	-0,370998602	0,227423818
OR51G2	-0,370807929	0,979133402
IGLL3	-0,370598457	0,68978159
VPS11	-0,370367888	0,108918712
LNX2	-0,370274739	0,282594344
SNRPB2	-0,370155569	0,437601584
DSG3	-0,369878375	0,166670743
GPR20	-0,369847915	0,783688931
FLJ43276	-0,369804649	0,063736046
TOPORS	-0,369614699	0,491367571
SMPD3	-0,369543004	0,217286128
SMOX	-0,369334823	0,863102332
LOC646049	-0,369285876	0,451192992
GOLGA7B	-0,369033538	0,058281194
LAIR2	-0,368900206	0,118812637

ZNF14	-0,368868997	0,221599518
LOC644403	-0,368792132	0,134894935
C10orf35	-0,368734382	0,126516739
COMMD7	-0,368514464	0,097626702
SETD6	-0,368453566	0,853883915
RNF139	-0,368345874	0,671442773
OR5K4	-0,368218205	0,098980832
ZNF18	-0,36818665	0,044762764
MS4A4E	-0,367999017	0,133494697
OR2S2	-0,367913106	0,510084392
TK1	-0,367874286	0,143217928
FAM40B	-0,367832222	0,23034604
DEPDC1B	-0,367688058	0,317854884
SCP2	-0,367582242	0,085869047
CA2	-0,367530645	0,099963986
ZMYM1	-0,367462648	0,003348441
ANXA13	-0,367431018	0,50968743
GPR103	-0,367221637	0,094140298
CHCHD10	-0,367058752	0,480539388
DEPDC5	-0,367049876	0,051384909
HSPC111	-0,366944277	0,132776358
C10orf12	-0,366515735	0,219470586
GABRD	-0,366421071	0,194334384
DLX4	-0,366357178	0,294048087
SCARF1	-0,36627393	0,042840456
SLURP1	-0,365979953	0,324772592
HNRPUL1	-0,365897042	0,960337248
PET112L	-0,365771109	0,033328712
DBNL	-0,365755105	0,980741804
NRCAM	-0,365695177	0,104898951
YES1	-0,365663306	0,9195856
HIST1H3C	-0,36563282	0,759620561
SPDEF	-0,365602518	0,711394157
RNASE3L	-0,365597013	0,748043785
TESK1	-0,365579717	0,345635332
HIST1H3J	-0,365381526	0,068916522
LMO4	-0,365315838	0,151064381
ATP1B2	-0,365273876	0,13647107
ENO1	-0,365064534	0,286470638
DHX15	-0,364842987	0,260668403
LOC647836	-0,364651156	0,034930811
REV1L	-0,364053252	0,286286686
LEMD2	-0,363955694	0,989347115
SAP30	-0,363859595	0,438303631
TERF1	-0,363809933	0,145281225

MCPH1	-0,363651776	0,203852562
ITIH1	-0,363564707	0,16678778
LOC728545	-0,363559902	0,541152306
LOC644090	-0,363550684	0,937789739
FAM96A	-0,363448235	0,321684902
CHES1	-0,363421269	0,653106795
FOXG1B	-0,363364874	0,324985184
LOC649684	-0,363266572	0,944870152
KRTAP1-5	-0,363035007	0,728060491
ACAA2	-0,362916262	0,560630241
DTR	-0,362911896	0,954155302
MYO9A	-0,362681674	0,254955498
FLJ46210	-0,362570201	0,114967667
C15orf40	-0,362443933	0,122294563
hCG_1988300	-0,362429931	0,406902763
RP1L1	-0,362401205	0,06851141
SPG21	-0,362176575	0,676460945
GALNT8	-0,362143431	0,355196382
C12orf28	-0,362017662	0,566101267
ARF4	-0,361724302	0,124979263
MFAP4	-0,361486797	0,11966075
LOC728660	-0,361385705	0,109346634
LOC388910	-0,361319337	0,390420289
KCTD10	-0,361199137	0,226047677
COQ10B	-0,36118209	0,12138983
LOC728817	-0,360965196	0,008692757
BMP2KL	-0,360886293	0,104306226
LOC440040	-0,360801201	0,90036797
SSBP2	-0,360689791	0,141649484
OR52A4	-0,360592372	0,590597981
MUSTN1	-0,360578474	0,853424853
RCE1	-0,360531106	0,018542641
KIAA1688	-0,360418346	0,049518163
CROP	-0,360253675	0,435554838
PGGT1B	-0,360111021	0,371905748
NCOA7	-0,360105518	0,632150567
IQCJ	-0,360081908	0,243359367
SLC46A3	-0,359954804	0,290581737
RAPGEF1	-0,359789958	0,132478487
LOC652641	-0,359533017	0,157158999
BAZ2A	-0,359410068	0,290292192
HOXA10	-0,359281939	0,802756289
TLX3	-0,359148892	0,062424801
LRRC36	-0,359139763	0,148122137
GALT	-0,359014117	0,597560512

ZCCHC2	-0,358901003	0,672104522
COG7	-0,358896107	0,087351467
GLE1	-0,358791205	0,881610361
RNUT1	-0,358712762	0,089621564
LOC729845	-0,358637086	0,817465356
GPBP1L1	-0,35861262	0,722211561
TMEM71	-0,358498711	0,204266904
SEC23IP	-0,358309285	0,260137344
UPF3A	-0,358201951	0,31096737
LOC728879	-0,358198779	0,202627923
PRPSAP1	-0,358151347	0,540231384
LRRC31	-0,358103179	0,241051519
CECR5	-0,357777208	0,937918353
LOC730733	-0,357718256	0,463791021
B3GALT4	-0,357558038	0,716500841
DNAJB4	-0,357469588	0,911915187
GLRX5	-0,357449482	0,920488281
LOC727792	-0,357398911	0,509111821
MMP1	-0,35637352	0,196243694
SLC38A6	-0,356332794	0,302098257
VDR	-0,356157568	0,671339002
C2orf60	-0,356049333	0,970972349
ARHGAP17	-0,355890582	0,118470113
UNQ6190	-0,355768928	0,151389282
LOC645955	-0,355602254	0,055369783
CCDC148	-0,355577917	0,721105828
LOC340089	-0,355363558	0,08392471
C10orf59	-0,355339345	0,559258045
FAM100B	-0,355279176	0,431220004
SCN8A	-0,35520073	0,086158345
TSSC4	-0,355173819	0,132384897
CHCHD6	-0,355159611	0,645417355
LOC731276	-0,355151731	0,20347487
FST	-0,355068246	0,063091814
FAM9C	-0,355048454	0,678452993
TM4SF19	-0,35503816	0,723799101
HSPA1A	-0,354763618	0,50663598
LOC401152	-0,354652474	0,014577583
SRP54	-0,354492647	0,016788735
FANCM	-0,354312832	0,089040306
GRAMD1B	-0,354307304	0,822360717
MTHFS	-0,354141347	0,65905241
C10orf33	-0,354117363	0,881154745
FLJ10997	-0,354044964	0,661468474
LOC401220	-0,35389132	0,397659421

FASN	-0,353869904	0,077147114
MEST	-0,353863041	0,185089576
USMG5	-0,353852051	0,071804599
STMN3	-0,353759177	0,582653211
ASB16	-0,35366006	0,07568
LOC731151	-0,353334146	0,519337168
ADAMTS1	-0,353021726	0,07271095
CCDC13	-0,352961827	0,186228385
LOC646031	-0,352938215	0,405128299
LOC129870	-0,352869919	0,594943819
SH2D1B	-0,352745391	0,831994885
EXTL3	-0,352553346	0,449152715
LOC729434	-0,352318415	0,455488869
LOC731304	-0,352060502	0,056858507
NFS1	-0,35183258	0,340431155
IL17RE	-0,351716365	0,70156477
LRAT	-0,351657846	0,229808239
GRIA3	-0,351396124	0,107062453
C18orf32	-0,351243154	0,663109731
CLEC2B	-0,351012815	0,220151329
OSBPL10	-0,350961392	0,237296719
FLJ37543	-0,350822805	0,02067649
PPP1R16B	-0,350395562	0,116931026
PERP	-0,350218046	0,428255354
ACY3	-0,349992325	0,563317496
TRIP3	-0,349967999	0,472385632
RPAP3	-0,349932488	0,899647567
TXNL5	-0,349830443	0,763496807
LAMP2	-0,349783228	0,447271866
PAF1	-0,349743205	0,08773379
C9orf71	-0,349606006	0,622283476
UCK1	-0,349579362	0,147490991
MAGEA4	-0,349549064	0,676531515
LYPD3	-0,349381247	0,910320472
GMNN	-0,349289618	0,219355239
C4orf42	-0,349279041	0,010515931
CRYAB	-0,349219193	0,03631988
GLYAT	-0,349145682	0,661092745
KRTAP13-1	-0,349056194	0,899390982
INTS12	-0,348937827	0,204528319
NSF	-0,34893095	0,056446023
NFIL3	-0,348901375	0,100671452
PRKCSH	-0,348899641	0,132435947
NAT1	-0,348867222	0,112193507
LOC441052	-0,348862107	0,799210575

CHCHD8	-0,348690536	0,357083462
STXBP5L	-0,348584118	0,670953124
DIRC2	-0,348134393	0,141935221
CUL7	-0,348056452	0,859250165
ZBTB9	-0,347763573	0,316923245
SERF2	-0,347722108	0,256425633
TRMT2B	-0,347555778	0,198815873
WFDC5	-0,347380926	0,156687826
TELO2	-0,347252417	0,207041039
PC-3	-0,347238604	0,072856462
THAP10	-0,347170505	0,075331614
LOC644099	-0,347040837	0,934597759
HDHD2	-0,347040018	0,176336385
RFTN2	-0,347027867	0,618096591
ARHGDIA	-0,346988224	0,999396085
08, Sep	-0,346949397	0,371419769
NME3	-0,34678731	0,279719996
LOC202459	-0,346613648	0,3340453
LOC644171	-0,346505246	0,108303964
MRPL39	-0,346446924	0,604536375
INO80B	-0,345975343	0,810732698
DEFB128	-0,345954523	0,960548859
ARMC5	-0,345950276	0,099468139
C6orf206	-0,345736039	0,629217906
MTL5	-0,345719317	0,735300762
VPS33B	-0,345647391	0,100008702
RSPO2	-0,345563852	0,100166863
LIMR	-0,345527833	0,414204352
DKFZp761I1011	-0,345465447	0,419553489
FGD4	-0,345397332	0,115148022
C9orf106	-0,345271837	0,546962512
LOC729001	-0,345167714	0,558108156
LOC730103	-0,345135259	0,371563739
LOC729898	-0,345097036	0,352506667
SH2D3C	-0,344853753	0,13008838
MGC57359	-0,344801632	0,569501557
C20orf200	-0,344650464	0,551808371
FLJ20444	-0,34462606	0,330090989
TBX21	-0,344582209	0,027401298
LOC730643	-0,344538292	0,440224138
RNASE11	-0,344413927	0,200912956
MYCBP2	-0,344298704	0,534137307
PPP3R1	-0,344181286	0,2267634
RSBN1	-0,343992143	0,162634034
TMEM68	-0,343921284	0,737214858

FLJ41278	-0,343775601	0,695807833
OR51S1	-0,343734126	0,639851848
C18orf30	-0,343566425	0,52736484
PPIL3	-0,343530346	0,280586554
LOC644055	-0,34351721	0,813830356
PIAS4	-0,34347876	0,755032657
C6orf136	-0,343394276	0,048887083
GDNF	-0,343391672	0,277798748
FOXI1	-0,343319551	0,299827529
LOC651400	-0,343259352	0,567713742
ZNF511	-0,343237292	0,236596816
BRDT	-0,343222881	0,151526819
KCNH3	-0,343190987	0,299063693
REP15	-0,343160306	0,48910638
CHAT	-0,343024073	0,478954145
NIPSNAP3A	-0,342955186	0,550430746
SIPA1L1	-0,342735757	0,469032127
PPP4C	-0,342620151	0,244151478
CHORDC1	-0,342585897	0,239825042
KCNMB1	-0,34252604	0,980567267
LRRCC1	-0,342521285	0,305241279
KIF9	-0,34236	0,157579983
C6orf195	-0,342202651	0,387287618
SLC35D3	-0,342081388	0,857361476
CCDC55	-0,341908338	0,762785543
FAM123A	-0,341623969	0,617539814
DPH3	-0,341519402	0,058077117
CRI2	-0,341512719	0,910835512
C3orf21	-0,341508794	0,389920088
ARHGEF4	-0,341147092	0,219947014
LOC728155	-0,341045839	0,056829652
PLOD3	-0,340899304	0,312407492
APOM	-0,34088276	0,13016498
HPD	-0,340705092	0,05781421
PLSCR2	-0,340683221	0,119186017
TREM1	-0,340595475	0,20601499
LOC649891	-0,340568818	0,560512365
COX19	-0,340361158	0,569912976
LOC644504	-0,340148669	0,23691346
LOC728771	-0,340145132	0,129871095
AOC2	-0,340130427	0,79441762
EEF1A1	-0,340125727	0,574872186
C1orf157	-0,340099161	0,5
RXRG	-0,340008162	0,543450026
LOC731407	-0,339992375	0,257891503

KPTN	-0,33998785	0,147486095
HELB	-0,339374823	0,533648909
MCART6	-0,338940077	0,795751395
CDC25B	-0,33890289	0,307508461
RGN	-0,338732418	0,923883809
PAGE3	-0,338617388	0,244958146
ZNF16	-0,338606029	0,455123546
PRSS25	-0,338591781	0,475189211
RNPC2	-0,338546221	0,841039892
UTRN	-0,337900612	0,172536176
GOLGA5	-0,337859963	0,174434054
FXC1	-0,337856353	0,077290396
MTO1	-0,33780877	0,109901959
POLR2A	-0,337737543	0,366375691
ADH7	-0,337714521	0,997926196
PRODH	-0,337701295	0,095133079
PHACTR2	-0,337630585	0,25429029
TRAPPC4	-0,337622914	0,958758064
LOC727995	-0,337523155	0,74102968
DKFZP566E144	-0,337371621	0,97040527
RNF182	-0,337360763	0,421937193
TRNT1	-0,337023938	0,526952875
LOC729155	-0,336999449	0,06410766
LOC729034	-0,336926741	0,107859397
MAGEC1	-0,336866796	0,356269634
GMPR	-0,336789464	0,210213923
PIK3CD	-0,336704495	0,322278208
COL28A1	-0,336702195	0,736403222
TAS1R1	-0,336695125	0,102885676
LOC727866	-0,336399604	0,059299356
MYOG	-0,336365018	0,261164719
LST1	-0,336311437	0,171905696
C1orf103	-0,336268689	0,421789252
PCNT	-0,336248969	0,604687152
SPEM1	-0,336174636	0,483779779
SGPL1	-0,336131217	0,333952434
C2orf57	-0,336121136	0,034697159
SPON1	-0,336046897	0,42912637
TMEM132B	-0,336041718	0,469207604
SLC43A1	-0,335868906	0,282575209
IL4R	-0,33570398	0,192748898
YIPF6	-0,335581765	0,832302991
SPOP	-0,335574585	0,776687088
TXNIP	-0,335547726	0,108330911
ZMIZ1	-0,335425817	0,500387886

LOC344065	-0,335359219	0,561437252
NPTX2	-0,335358133	0,026539427
WHDC1L2	-0,335280241	0,155952621
PRR11	-0,335210693	0,504155794
PPP4R2	-0,335200575	0,034673385
UBA52	-0,335112563	0,477254186
SLC7A11	-0,335023058	0,102147502
LOC400682	-0,334843427	0,437624495
LOC643227	-0,3348406	0,535083323
LOC728717	-0,33483434	0,501223929
BAX	-0,334700672	0,365086129
CYP1A1	-0,334501755	0,318653092
FOXN1	-0,334424111	0,859704815
NBEAL1	-0,334346184	0,040141301
KCNN4	-0,334252221	0,583768713
DCTN6	-0,334141172	0,721599641
TCEAL3	-0,334086952	0,052213366
NOS3	-0,33367972	0,346722497
LOC646338	-0,333442136	0,921895472
SOX30	-0,333186061	0,270707043
GRIN2C	-0,333175779	0,076787811
NDRG1	-0,332727864	0,214614593
FGF18	-0,332336721	0,123896909
DNCL2A	-0,332322829	0,210107954
LOC731709	-0,33232111	0,130126728
TMEM179	-0,332227579	0,238424108
ALDH5A1	-0,332169463	0,143917433
BCL3	-0,331186735	0,134398294
TINF2	-0,331163709	0,308277176
C7orf43	-0,33101507	0,87487411
IGSF3	-0,330986407	0,033302361
CCDC150	-0,33097665	0,18810505
EGFL4	-0,330926521	0,50584745
FLJ43752	-0,330915218	0,024020122
C17orf42	-0,330832351	0,069492371
OR7D2	-0,330753863	0,83776831
SLC16A5	-0,330662337	0,603071172
LOC729293	-0,330644245	0,08735601
TMPRSS7	-0,330476162	0,151125729
TTYH3	-0,330357861	0,711329102
OR4X2	-0,330150895	0,129141665
TSR1	-0,329946796	0,056715226
REG4	-0,329930427	0,926895488
HKR1	-0,329868103	0,584012382
PRPF38A	-0,329833496	0,075907575

GGTLA4	-0,32961389	0,915705294
OR51E1	-0,329596532	0,954183833
ASL	-0,329350526	0,672729712
C14orf128	-0,329204676	0,562835983
FRS3	-0,329196324	0,149759427
HIGD1B	-0,329196235	0,423024268
PRPF4	-0,329168075	0,070893419
ELA2	-0,329161683	0,501415925
MSI1	-0,32911479	0,061226024
LOC728633	-0,329082114	0,322286323
C16orf42	-0,32906858	0,29199084
DKFZp686I15217	-0,328994257	0,164039441
MCART1	-0,328935458	0,536769859
LOC401397	-0,328898508	0,196379934
MUC12	-0,328840804	0,161877075
MYOZ3	-0,32863314	0,071315644
LOC650433	-0,328569054	0,059194042
CYP19A1	-0,328552457	0,212218128
LOC729156	-0,328470496	0,176777791
SIGLEC7	-0,328361139	0,238734368
SHD	-0,32823252	0,873438017
GRID1	-0,328191261	0,58107339
SMARCAL1	-0,32815942	0,211926237
ATP6V1G1	-0,32812864	0,169626108
MELK	-0,328075212	0,285343235
SLCO3A1	-0,327853524	0,986764567
C4orf21	-0,327821397	0,417063894
OR5H1	-0,32773823	0,087617553
TOMM20	-0,327554224	0,381492061
NDN	-0,327530707	0,18571795
PQLC3	-0,32746616	0,575060958
FLJ16353	-0,327386898	0,316191251
HIST1H4A	-0,327372492	0,932742073
LOC645900	-0,327299353	0,063684917
ZBTB20	-0,326972354	0,511563163
PPIAP8	-0,326945711	0,248931914
GPR43	-0,32688385	0,691967573
TTC14	-0,326732308	0,59495701
OACT2	-0,32666688	0,641582732
TBCE	-0,326655779	0,480679667
BLVRA	-0,326568511	0,709096628
LOC644852	-0,326507702	0,09573103
LOC642108	-0,326495433	0,964878513
BHLHE22	-0,326367854	0,195536636
SERPINA7	-0,325727567	0,803774094

SERP1	-0,325722116	0,858091992
TBX20	-0,325686167	0,264526056
tcag7,956	-0,325653284	0,16400227
OR2A5	-0,325617014	0,067741643
CDH16	-0,325588527	0,562795083
FRBZ1	-0,325475568	0,800959963
LOC729478	-0,325464377	0,602418415
LOC646079	-0,32540219	0,710708195
KHDRBS1	-0,325297517	0,071670679
USP6NL	-0,325123922	0,067737254
DEFB118	-0,325095203	0,151451674
SHMT1	-0,325034697	0,809946492
ZNRF3	-0,324996186	0,715503846
APTX	-0,324797275	0,180193999
TAS2R8	-0,324765871	0,120313097
KPNA5	-0,324759669	0,404000025
PREP	-0,324739541	0,777907889
USP11	-0,324711877	0,120525818
TPPP2	-0,324705685	0,42236912
RBP5	-0,324605879	0,82710723
CCDC53	-0,324506556	0,146425607
RRP7A	-0,324378805	0,997268256
ABCB11	-0,324375246	0,192408081
ABHD4	-0,32436083	0,207594201
FOLR3	-0,324298418	0,200777155
LOC338667	-0,324290299	0,159552502
KIAA1529	-0,324197178	0,915738283
C14orf23	-0,323879072	0,101230847
ZNF192	-0,323834854	0,268936637
LOC83468	-0,323726379	0,679290572
LOC652795	-0,323629561	0,646531049
TSPAN10	-0,323153282	0,231367684
LOC338739	-0,323123061	0,268148252
IGSF22	-0,323071672	0,653830883
ANKRD58	-0,323068952	0,265690709
NEUROD6	-0,323050627	0,129297605
hCG_1979841	-0,32298256	0,526095938
CCNYL2	-0,32289044	0,232291001
RFX1	-0,322686498	0,175895503
FAM63A	-0,322641942	0,965733292
KIAA0460	-0,322491924	0,064360762
BAT5	-0,322465983	0,92701903
MYST1	-0,32239234	0,789282825
ACO1	-0,322351031	0,129422219
TMPRSS9	-0,322336399	0,825466161

CIB1	-0,32223749	0,602724368
ADPRH	-0,322227436	0,211796772
FBXO46	-0,322185746	0,29293356
FLT4	-0,322096469	0,630188153
LDB1	-0,322090656	0,083061999
TTC25	-0,32197814	0,513985714
LOC649798	-0,321975263	0,061699938
TMEM44	-0,321847071	0,802982189
TRE17	-0,321796253	0,295656101
CRYGD	-0,321769274	0,260743366
OR2AT4	-0,321767148	0,78330082
LOC730321	-0,321765317	0,383375705
GBP4	-0,321638227	0,249004756
ATPIF1	-0,321602701	0,476112866
SLU7	-0,321580931	0,279456072
LSM10	-0,321551224	0,292082911
CKAP1	-0,321549975	0,376437958
JAM2	-0,321503876	0,817018445
POLRMT	-0,32145506	0,92039696
OR2W3	-0,321145989	0,88009031
LOC730198	-0,320924007	0,053961827
SLC9A3R1	-0,320843953	0,099473472
C19orf40	-0,320836607	0,459453692
BOC	-0,320745409	0,495637605
GHRHR	-0,320700807	0,395846627
C14orf82	-0,320629116	0,084402945
ZNF408	-0,32051473	0,154100106
G10	-0,320494773	0,062240256
MYO1C	-0,320122761	0,126617977
PHACTR4	-0,320092724	0,675595634
SEC31B	-0,320001479	0,102856882
RPL12	-0,319911274	0,564614991
INTS1	-0,319900972	0,778956161
MYO10	-0,319883185	0,384705741
LONP	-0,319767402	0,027756325
OSBPL1A	-0,319721557	0,587604404
HIST1H4F	-0,319672029	0,942824205
ZNF593	-0,319664505	0,199753358
CH25H	-0,319584766	0,657299264
ACBD6	-0,319557731	0,832989256
UQCR	-0,319510933	0,105405705
C4orf31	-0,319482277	0,156847273
AIM1	-0,319291985	0,567141687
C3orf15	-0,319136881	0,049746569
RASGRP1	-0,319119312	0,113029927

INTS4	-0,319050085	0,825138977
TAP1	-0,31903223	0,850585943
LOC727824	-0,318982211	0,274471883
LOC731408	-0,318935794	0,083859224
CMTM6	-0,318887286	0,865632411
ZNF389	-0,318573754	0,019612129
DZIP1	-0,318564725	0,171163152
ANGPTL4	-0,318509459	0,648505505
KIAA1147	-0,31846369	0,287181462
LOC728987	-0,318369042	0,67387007
ZNF346	-0,318231472	0,261741878
EVI5	-0,318179279	0,922569504
OR56B1	-0,318157089	0,021070619
RABGEF1	-0,318156433	0,940338689
POLN	-0,318128647	0,556437857
DDX26	-0,318043669	0,371547265
B4GALT7	-0,317921695	0,907033903
C6orf167	-0,317753082	0,666752187
PCDHGA10	-0,317707115	0,23726448
RNASEL	-0,317581792	0,592414874
CBLL1	-0,31757074	0,873053621
SIX2	-0,317551163	0,130320916
LOC728607	-0,317300233	0,832127059
RNPEP	-0,317242881	0,312750588
EEF1A2	-0,317155691	0,456547638
OR1F1	-0,316898707	0,329385817
FAM155B	-0,31687591	0,663731885
PAK1	-0,316790787	0,938468267
VILL	-0,316778453	0,554234795
HTR2B	-0,316744117	0,900815209
FLJ41766	-0,316562365	0,841113391
ASAH2B	-0,316492272	0,755646562
CCBP2	-0,316389416	0,117480396
C7orf16	-0,316238419	0,630914304
LOC643264	-0,316168253	0,638013487
LOC728168	-0,316161362	0,135900723
hCG_1781062	-0,316106428	0,311924926
LOC645195	-0,316092204	0,307622835
KIAA1598	-0,316083319	0,284029544
PRKCM	-0,316052009	0,321649052
SLC16A10	-0,316045379	0,726508725
BMP8B	-0,315835235	0,166289989
DEFB125	-0,315797837	0,147673751
ARHGAP20	-0,315677526	0,397467756
PPM1A	-0,315651933	0,29635671

ZBTB2	-0,315598057	0,546015466
LOC729454	-0,315557786	0,125113687
OR1L4	-0,315543737	0,096689835
LOC644885	-0,315408401	0,362855375
LOC730462	-0,315316499	0,27658571
RNASE13	-0,315250865	0,541825629
TMEM163	-0,315214474	0,895171107
DNAJB5	-0,315090926	0,616604317
F10	-0,315044622	0,793661954
TRAPPC1	-0,314986367	0,27693254
LOC728653	-0,314913204	0,688596248
LOC730115	-0,314848879	0,783396903
LOC728463	-0,314825741	0,889847161
PCDHGA4	-0,31453523	0,966050436
TGFB2	-0,314426478	0,205267245
PTGFRN	-0,314067986	0,164412344
SLC7A10	-0,313946016	0,01758872
GUCA1C	-0,31389	0,546477422
AKR1C2	-0,313814287	0,984530141
ILDR1	-0,313748639	0,781290769
TXNDC3	-0,313496933	0,519916793
LOC284417	-0,313474142	0,500501038
SPANXN5	-0,313286261	0,061919412
C1orf61	-0,313272187	0,758414988
FAM62C	-0,313216304	0,337058349
OBSL1	-0,313036833	0,400163445
INPP4B	-0,312869412	0,826934345
DUX2	-0,312606066	0,175550885
LOC390688	-0,312443216	0,304706379
LRFN5	-0,312339186	0,633047276
PCDHA1	-0,312295035	0,117512909
LOC148003	-0,312251034	0,953391486
LOC731551	-0,31220715	0,089439162
FKBP1B	-0,31211015	0,205804836
SMURF1	-0,312073359	0,49402084
PDZK3	-0,31207225	0,067855587
STK23	-0,311940044	0,206300722
PF4	-0,311872799	0,055083092
PDE10A	-0,311849578	0,097427672
TBL1Y	-0,311791228	0,796461673
C17orf74	-0,31176188	0,705185773
SPATA5	-0,311746758	0,43640241
C9orf167	-0,311549872	0,115829702
SPATS2	-0,311547092	0,023655957
ADO	-0,311477164	0,547204463

E2F3	-0,311306705	0,691414839
NR3C1	-0,31121545	0,167896113
GEN1	-0,311110228	0,300277058
POLL	-0,311082845	0,865761313
LOC644718	-0,31085289	0,084126445
C4orf30	-0,310700928	0,826885747
KNDC1	-0,310693227	0,11906574
SPATA22	-0,310414005	0,281452561
LRRC66	-0,310128055	0,177823262
LEAP2	-0,310078278	0,94744678
NALP11	-0,309984808	0,910352675
UCHL5IP	-0,30991544	0,196843923
UFC1	-0,30968911	0,115049221
GDF3	-0,30966571	0,342861518
RECK	-0,309512317	0,743068125
IBRDC2	-0,309506372	0,328761645
OPRS1	-0,309396343	0,111673617
MOV10	-0,309359078	0,177561617
SIGLEC15	-0,30930593	0,424538259
CCDC28B	-0,3092315	0,114190874
LOC730031	-0,309176113	0,876614588
C1orf100	-0,309043333	0,160138376
RAB10	-0,308974059	0,104812064
LOC641905	-0,308877868	0,512893829
OR5AP2	-0,308787744	0,776779558
FLJ13842	-0,308734554	0,162845582
TMEM168	-0,308677919	0,568775657
RIMS1	-0,308514157	0,615286352
JRK	-0,308156801	0,286633759
RNF13	-0,308106048	0,301834918
GEFT	-0,308098554	0,874268818
TFIP11	-0,308046246	0,22486131
LOC728221	-0,308038993	0,804729988
ASB1	-0,307865145	0,15451429
KRTAP27-1	-0,307605691	0,238919015
IL2	-0,307594366	0,245919836
DLX5	-0,307205885	0,137197296
IL1RN	-0,307070228	0,153580308
PTGDS	-0,307028893	0,511780704
TACR3	-0,306984514	0,055591221
LOC650203	-0,306977483	0,227641776
MRPS27	-0,306921404	0,074652974
LOC727900	-0,306921388	0,155370957
LOC645967	-0,306904615	0,795338495
SYT5	-0,306819009	0,84001302

VSTM1	-0,306803566	0,142625541
CBLN1	-0,30676944	0,328074014
CYP1B1	-0,306699558	0,591286309
KLK3	-0,306574431	0,167044267
YJEFN3	-0,306536157	0,138378479
DSCR1	-0,306167001	0,421582513
GPR147	-0,306086365	0,657051077
LOC732312	-0,305754029	0,513255515
LOC648149	-0,305753916	0,453205116
LOC727838	-0,305713075	0,440450449
FLJ38281	-0,305712724	0,093883601
ANKRD30A	-0,305706233	0,797235006
TSPAN3	-0,305586195	0,914113321
NAPSA	-0,305555069	0,495907283
PSKH2	-0,305486124	0,09742452
SERPINA11	-0,305428622	0,282813832
UHRF1	-0,305411615	0,547591865
HNRNPG-T	-0,305384371	0,444437697
SLC5A9	-0,305373512	0,714900005
LOC727955	-0,305307974	0,210906342
NFU1	-0,305192521	0,618107481
LOC730225	-0,305058793	0,094534854
GJA4	-0,30479863	0,17398404
ITPKB	-0,304693057	0,895752198
BCR	-0,304658544	0,883560741
RAB26	-0,304658124	0,208090097
SNX5	-0,304649927	0,323764935
GALNT13	-0,304621762	0,908091415
NP	-0,304609548	0,305585663
C9orf57	-0,3045815	0,455502784
TXNDC	-0,304550507	0,037965963
ESPN	-0,304305516	0,571781095
HMOX2	-0,304266295	0,346989919
SERPINH1	-0,304086596	0,85540992
CCL1	-0,304067527	0,988640402
TMEM60	-0,303782381	0,854421743
VPS16	-0,303574231	0,102549693
USP29	-0,303475675	0,124610837
OR4K13	-0,303372158	0,038870119
G6PD	-0,303222213	0,632588512
LOC728784	-0,302857493	0,990885073
CCDC144B	-0,302842543	0,523039134
RRP1B	-0,302662533	0,135875613
CDS2	-0,302534335	0,987335426
PCDHB16	-0,302486691	0,429102062

DYT1	-0,3023931	0,092179963
CDCA2	-0,302251683	0,159970182
ANXA11	-0,30217299	0,656125219
FLJ22447	-0,301985419	0,122316067
LSM14A	-0,30190127	0,299986529
GJB6	-0,301862224	0,675348473
MRPL45	-0,301734259	0,61115553
ZXDB	-0,301707893	0,268187541
LOC196913	-0,301590148	0,114951938
GGA1	-0,301444752	0,049424727
EFR3B	-0,301335144	0,039892852
PCBP3	-0,301288087	0,130034845
C2orf7	-0,300998302	0,197819352
CFL1	-0,300856555	0,254479881
LHFPL3	-0,300768741	0,033933129
ARNTL2	-0,300743387	NA
PCCA	-0,300661443	0,514081687
MESDC2	-0,300596808	0,910442949
SLC35A2	-0,300470008	0,61718898
KLF16	-0,300291394	0,305401676
OR6N2	-0,300279957	0,871265154
LOC729432	-0,300240671	0,705524621
HSPA6	-0,300041197	0,845564514
ZNF75	-0,299988109	0,78321829
OR9Q1	-0,299957558	0,017147444
KCNJ4	-0,299846455	0,593883832
MYBPHL	-0,299760156	0,17339951
TBR1	-0,299751297	0,259299387
SLC39A7	-0,29971941	0,643177635
TMEM37	-0,299715993	0,061850407
LOC643905	-0,299693139	0,824790649
DGCR2	-0,299676233	0,631132973
IL1F8	-0,299652496	0,090379395
TMEM160	-0,299500702	0,849995474
SCO2	-0,299484129	0,59265374
ARPC2	-0,29948328	0,052195217
C20orf149	-0,299469122	0,347956734
CLIP3	-0,299146964	0,158688195
WBSCR21	-0,299109767	0,840531799
LOC728960	-0,299107476	0,666674313
KUB3	-0,29906932	0,096740067
LOC729640	-0,299017962	0,653596953
TAPT1	-0,298982142	0,606696908
SYT16	-0,298667126	0,273971245
RANGRF	-0,298546353	0,227918947

LOC650832	-0,298369885	0,118567892
TIMM23B	-0,298224191	0,177637617
PCID2	-0,298201728	0,127316472
UBFD1	-0,298068341	0,102786155
FAM90A3	-0,298054068	0,061497945
FBXO10	-0,298052034	0,344726805
SH2D2A	-0,297925268	0,043882565
DDX47	-0,29783325	0,725234479
TIMM50	-0,297804841	0,488269187
RERGL	-0,29758514	0,218930564
CHRNE	-0,297581721	0,028471275
LOC653162	-0,297574064	0,791902099
SIPA1L3	-0,297490241	0,874240511
FLJ45872	-0,297480077	0,180840724
RNF219	-0,297402901	0,127927902
TTC38	-0,297388712	0,09647402
BUB1	-0,297372982	0,957088048
CA7	-0,297370614	0,854216882
LRP5	-0,297197915	0,86717106
C1orf192	-0,297052373	0,854266261
SP7	-0,296940628	0,109516576
ANKRD43	-0,296809137	0,09142149
TBC1D10C	-0,296561814	0,728300555
LPO	-0,296507014	0,847249039
hCG_2040201	-0,296236415	0,100608894
LOC653882	-0,29614509	0,105941724
RNPEPL1	-0,296082308	0,360773492
BCMO1	-0,29606513	0,079778859
BCL11B	-0,29598364	0,278892629
DFFA	-0,295915867	0,289956361
NRG4	-0,295795083	0,077496954
ETFA	-0,29566319	0,014431595
SLCO5A1	-0,295533938	0,186534516
TAP2	-0,295427069	0,034462938
NEK8	-0,295406629	0,350700006
LOC647281	-0,295387445	0,066501298
LOC730085	-0,295305013	0,136757103
BTBD2	-0,295205261	0,458732808
PDCL3	-0,295164886	0,534349379
EPB41L5	-0,295155625	0,953153151
TMEM99	-0,295065344	0,689726166
ELL2	-0,295033098	0,320169158
FLJ11838	-0,294961288	0,193072248
ZC3H6	-0,2947649	0,912529265
LRRN5	-0,294512437	0,624672736

FLJ14490	-0,294502657	0,2703005
B4GALT4	-0,294369599	0,312727122
NGFR	-0,294270915	0,784933474
GJD4	-0,294255301	0,1249734
SOX18	-0,294211385	0,106273562
FN3K	-0,294037494	0,097693593
SLC12A4	-0,293821852	0,301325035
VRK2	-0,293743746	0,589958049
FZD6	-0,293588416	0,133481786
LOC729040	-0,293574818	0,065752901
TAS1R3	-0,293412361	0,699510172
GYG2	-0,29336311	0,955121274
HBE1	-0,293123066	0,947012281
TREML2	-0,29310813	0,996916711
PDP2	-0,292847728	0,123272289
MAGI-3	-0,292758486	0,500304641
LOC647509	-0,292678842	0,790752544
CCL17	-0,292348217	0,988927092
TARBP1	-0,292335221	0,250223296
AADACL2	-0,292301337	0,7443642
NELL2	-0,29201356	0,89677198
DDX60	-0,291959883	0,056103491
PRR13	-0,291957291	0,255780473
APOBEC1	-0,291848005	0,160731112
MRPS12	-0,291822506	0,48878266
TMED9	-0,291722099	0,098705226
AKR1CL1	-0,291694891	0,860816095
ANKK1	-0,291644956	0,591946006
SOCS1	-0,291561932	0,045194557
LOC727804	-0,29149341	0,776764304
MOSPD3	-0,291473969	0,259002175
LOC643703	-0,291161089	0,041476264
LOC730070	-0,291047475	0,095964326
TTID	-0,290993692	0,045490181
SUCLG1	-0,29090889	0,451003511
CCDC69	-0,290475821	0,905766217
PDE4DIP	-0,290461462	0,374182694
TZFP	-0,29037663	0,498251389
MGLL	-0,29035246	0,15201727
RC3H1	-0,290308972	0,110358679
LOC730251	-0,290006465	0,712175284
TMEM125	-0,289880742	0,18361239
BZRP	-0,28980143	0,475052164
SKAP2	-0,28977466	0,556304819
RNF29	-0,289649825	0,800041165

CLIC4	-0,289605313	0,319429893
CAMKK1	-0,289157643	0,898358701
NTHL1	-0,289124067	0,721148144
IFT20	-0,289006085	0,035245977
FAM158A	-0,2889619	0,836236808
LOC652292	-0,288865158	0,945450285
TUBD1	-0,2887776	0,144693893
C13orf39	-0,288714439	0,725147401
PTPNS1L2	-0,288652179	0,361887753
RUVBL1	-0,2885862	0,95512129
ZNF441	-0,288533813	0,888765414
XRCC2	-0,28852338	0,738575519
RNF170	-0,288381991	0,742299873
PELI2	-0,288127381	0,896597033
TBL1XR1	-0,288095414	0,235886623
GLT25D2	-0,288021525	0,750235123
DKFZP781G0119	-0,288016969	0,150645293
BBOX1	-0,287926863	0,137444714
ZNF426	-0,287901208	0,937348238
ADRA2A	-0,287734987	0,936958525
SIAT4C	-0,287591078	0,778774844
LOC729821	-0,28751968	0,079153351
LOC728748	-0,287483222	0,544542577
KITLG	-0,287395723	0,496824603
LOC120364	-0,287296918	0,244872691
CALU	-0,287084345	0,121582603
FAM71C	-0,286864369	0,258551693
DBF4B	-0,286839514	0,183188167
C2orf47	-0,286822061	0,401363484
LOC729929	-0,286685673	0,557113959
BAD	-0,286577561	0,304780625
THBS2	-0,286555691	0,1292312
BCOR	-0,286479559	0,879295331
SLC10A4	-0,286352048	0,667449031
CD6	-0,286191251	0,07971894
S100A11	-0,286055941	0,978405558
GAL3ST1	-0,285922516	0,144305546
CCL22	-0,285915683	0,327249065
LOC648640	-0,285880498	0,901676655
FAM38A	-0,285768712	0,256568474
ATPBD4	-0,285675029	0,035077963
FAM32A	-0,285613894	0,887794149
CRHR2	-0,285525643	0,311756049
KCNG1	-0,285135981	0,136540651
CNOT2	-0,28511893	0,398158887

CSN1S1	-0,285091359	0,102512802
PDCD10	-0,285047484	0,07683608
SYT3	-0,284929106	0,550204996
MYBPH	-0,284758478	0,942158632
LRRC50	-0,284632504	0,734766191
HTR4	-0,284490206	0,898890733
EPB49	-0,284439393	0,12624489
VAX1	-0,284146571	0,414568611
C11orf21	-0,284116584	0,344912947
TRPM6	-0,284097069	0,449690287
LOC145845	-0,284082604	0,920164765
AADACL4	-0,284042536	0,212672934
NCKIPSD	-0,284024403	0,223243402
TUBGCP5	-0,283945734	0,410859605
TIP120A	-0,283816359	0,823623063
ALX4	-0,283718581	0,388931553
ZNF510	-0,283679494	0,953705646
PTMS	-0,283620684	0,992157256
OR7G3	-0,283545332	0,77539926
SLC12A7	-0,283472563	0,078410173
C12orf67	-0,283433652	0,023793646
ZNF259	-0,283032847	0,209538231
ATF1	-0,283030604	0,193953284
LOC731311	-0,283026573	0,991794446
CCDC37	-0,282936889	0,38462197
C14orf135	-0,282929871	0,821954872
FANK1	-0,282801908	0,062680736
ZNF495	-0,282737727	0,766022146
ZNF454	-0,282583553	0,039464867
SH3BGRL3	-0,282566379	0,818191567
ARD1B	-0,282526326	0,163641965
LOC650638	-0,282429577	0,120082975
SYT1	-0,282316734	0,121432428
CXCR4	-0,282241222	0,244940137
LOC390226	-0,282187059	0,536797286
LOC338829	-0,281981231	0,072390751
ASH1L	-0,281874836	0,65008891
LOC728723	-0,281852194	0,198621198
C1orf53	-0,281828705	0,615293166
RIT1	-0,281796416	0,789923823
RFP	-0,281787868	0,529838947
HMX2	-0,281639382	0,528853344
TOP2A	-0,281561219	0,670612886
ANP32A	-0,28153337	0,336487406
PAQR9	-0,281303753	0,078935654

ERVK6	-0,281234268	0,110533544
SLC26A7	-0,281220276	0,742882553
TFEB	-0,281157376	0,862177132
NAP1L2	-0,281123052	0,121265836
LOC647279	-0,281071728	0,23405286
INCA1	-0,281062283	0,138255408
BVES	-0,281027879	0,263490151
DGKE	-0,280925081	0,611882664
ARMCX1	-0,280720436	0,157326591
LOC645317	-0,280695274	0,178065142
HAO2	-0,280465037	0,672117012
T	-0,280431128	0,112007289
FLJ10858	-0,280424891	0,80776978
PGAM2	-0,280418973	0,971795797
KIAA1984	-0,280304624	0,619536079
PLOD2	-0,280231231	0,976594346
NXT2	-0,28023005	0,457623467
SCG2	-0,280151879	0,829451304
MCFP	-0,280144818	0,590744684
NOTCH3	-0,280083063	0,076952778
LOC731726	-0,280055613	0,193526392
GAB3	-0,279989383	0,125764685
MFNG	-0,279873015	0,232862523
REEP2	-0,279778114	0,556863705
KIAA0240	-0,279770276	0,964861169
LOC641696	-0,279751181	0,070175973
RMND5A	-0,279745553	0,103071704
RAD51	-0,279707635	0,417484114
PUM2	-0,279675776	0,303154522
CR1	-0,2795323	0,425380691
FSD1	-0,279507014	0,696832225
NTF4	-0,279481138	0,818210204
ACDC	-0,279465059	0,657728121
CML2	-0,279438194	0,532168801
C16orf55	-0,279388664	0,38619322
FAM149A	-0,279348724	0,285738332
COASY	-0,279301058	0,216535191
RNFT2	-0,27925967	0,149269576
RPS19	-0,279094673	0,613801215
LOC441239	-0,278939833	0,137129423
GRAMD3	-0,278917687	0,44426702
CYTL1	-0,278913435	0,823919887
PRM2	-0,278780095	0,978418632
FAM105A	-0,278764032	0,871290207
THUMPD2	-0,278519125	0,252883275

LOC728559	-0,278387023	0,273239427
KNG1	-0,278344977	0,596761719
LOC646471	-0,27827893	0,092849863
SLC10A1	-0,278254097	0,383330069
FAM55B	-0,278156385	0,5889567
WDR91	-0,277565213	0,583284208
ANKRD23	-0,277412472	0,615529922
DUSP12	-0,277397334	0,961731792
N4BP3	-0,277230642	0,167244505
KIAA0895	-0,27715129	0,745400105
ERI3	-0,277096665	0,207599608
DGKZ	-0,276994642	0,339474342
LOC730134	-0,276858616	0,286409518
EHD3	-0,276847452	0,178895222
CDAN1	-0,276746104	0,485333569
NANP	-0,276714243	0,56031012
LOC729308	-0,276699467	0,101128301
CHP	-0,276692341	0,022777439
CARD9	-0,276662558	0,374528355
LOC284120	-0,276510871	0,415707514
RPS6	-0,27648281	0,764827764
RNF40	-0,276393707	0,805509427
TBC1D21	-0,276356187	0,640937618
PLA2G3	-0,276234353	0,814118502
CSN3	-0,276233647	0,452433854
COL6A1	-0,276186587	0,385043885
SLC47A2	-0,276120505	0,957303036
C14orf37	-0,276105594	0,415127572
LOC644962	-0,276073509	0,754872264
CAMSAP1	-0,276050589	0,120290317
NXPH1	-0,275846167	0,121699542
ING1L	-0,275788932	0,189636789
FLJ39303	-0,275563244	0,84426299
ERCC2	-0,275479037	0,422086958
TNRC11	-0,275377084	0,768599104
SLC6A2	-0,275333896	0,511457607
AUTS2	-0,275210581	0,244860807
KLRK1	-0,275093079	0,161238377
MAGI1	-0,275021139	0,121804278
TPSD1	-0,274977038	0,569613257
HDAC11	-0,274713128	0,911220565
C1QTNF3	-0,274623498	0,378050743
PDDC1	-0,27457335	0,104354175
ZC3H12A	-0,274524545	0,820061455
LOC732119	-0,274513957	0,581813404

PME-1	-0,274411881	0,875853796
TXN2	-0,274337227	0,310624994
LOC387826	-0,274288652	0,242039852
ZNF494	-0,274257413	0,52990417
IFI44	-0,274208077	0,73166793
LOC646567	-0,27400329	0,404230885
UBLCP1	-0,273867511	0,674693397
LGALS9	-0,273835657	0,785943738
LRCH4	-0,273659758	0,53935186
PRUNE	-0,273450902	0,051106066
FAM65A	-0,273417686	0,940153962
ARP10	-0,273313512	0,80844405
CENPN	-0,273312084	0,841878312
KCNC1	-0,273309334	0,213029591
MRPL28	-0,273215952	0,666829307
DOLPP1	-0,273204453	0,34997896
2p-PDE	-0,27306185	0,920309333
KLHDC4	-0,273009878	0,083499923
RPA2	-0,272938175	0,872081328
XKR3	-0,272774308	0,132445981
FLT3LG	-0,272748044	0,066787102
RS1	-0,272702232	0,87631763
PPP1R3E	-0,272669312	0,129830034
LOC729490	-0,272658481	0,826012937
GLTPP1	-0,272597366	0,271401754
LOC731289	-0,272427146	0,612537009
MAOA	-0,272231712	0,560372726
LOC387763	-0,272058754	0,574426722
FLJ43980	-0,271977046	0,208890887
FBXW10	-0,27195353	0,559155229
SMC5L1	-0,271781969	0,591108979
MYO5A	-0,27164671	0,184328788
F8A1	-0,271531093	0,855139825
SEMA3D	-0,271520086	0,204053264
RAB13	-0,271371859	0,031944388
LOC402145	-0,271083218	0,832556717
TMEM216	-0,270876949	0,120279311
KLHDC8B	-0,270873506	0,601275078
LTBP1	-0,270806791	0,562516625
LOC646498	-0,270588074	0,089236756
OGDHL	-0,270576982	0,606402308
RBM35A	-0,270573237	0,214757436
ISCU	-0,270402815	0,34426018
BCAS4	-0,270389111	0,135226908
MCTP1	-0,27031808	0,818780828

PABPC3	-0,270287555	0,564833025
RC3H2	-0,270202205	0,735015631
ACOX2	-0,270175107	0,678680233
MTMR9	-0,270138504	0,176459251
ALPI	-0,270115337	0,434703528
LOC729363	-0,270057721	0,236005573
LOC283846	-0,2700541	0,209778391
C9orf152	-0,269959085	0,141303298
OR5P2	-0,269837495	0,231615845
CNTN2	-0,269727222	0,122358142
MMP15	-0,269708677	0,576580693
LOC441046	-0,269653027	0,532558762
LOC729563	-0,269577409	0,072212399
DDX39	-0,269494025	0,647166141
EIF4ENIF1	-0,26937414	0,884788659
LOC732273	-0,269319923	0,614045847
LOC133185	-0,269271034	0,11743945
FOXA1	-0,269270819	0,860646725
OTC	-0,269168854	0,413947467
PLD4	-0,269160113	0,205230055
KIAA1267	-0,269032015	0,436774237
ST6GalII	-0,268959088	0,277158307
HDDC2	-0,26878491	0,484285415
ADAM23	-0,268590696	0,89669004
CHMP1B	-0,268387344	0,588530026
IRX5	-0,268370799	0,438577551
UBASH3B	-0,268319653	0,55723076
C22orf33	-0,268255579	0,675729595
C14orf142	-0,268250485	0,121873038
APOOL	-0,268146349	0,857854763
LOC283755	-0,268108497	0,457907439
ZNF211	-0,26809655	0,141592719
SYT7	-0,268044455	0,721895108
SAMD13	-0,267894494	0,170498441
hCG_2019139	-0,267870673	0,951424957
RNF141	-0,267830435	0,220040495
FUZ	-0,267633085	0,583104121
MX2	-0,267590614	0,132163441
FKBP1A	-0,267561158	0,779238394
FUT6	-0,267512315	0,398677311
SNX27	-0,267050453	0,796288016
LOC727996	-0,26703263	0,091372835
CLCN4	-0,267016817	0,882958466
LOC650004	-0,266846197	0,095119956
NUP214	-0,266758126	0,124307592

FNDC5	-0,266646504	0,514655866
CCDC92	-0,266645968	0,673163464
NHEDC2	-0,266642522	0,244377403
MATN1	-0,266485649	0,734790414
INSM2	-0,266464139	0,110239626
CDV3	-0,266274528	0,382777482
FLJ16287	-0,266197425	0,377493303
LOC650538	-0,266090487	0,390543428
NUFIP2	-0,26587821	0,952470438
SLC2A8	-0,265743924	0,392895646
HOXA1	-0,26550923	0,393161318
MYO1A	-0,265406646	0,773522525
TFAP2B	-0,265304377	0,742771346
MNS1	-0,265235896	0,290633503
ZNF169	-0,265056904	0,750560485
MPV17	-0,265039861	0,604421637
MGC4767	-0,264815628	0,689219772
NOL6	-0,264740472	0,170726398
IZUMO1	-0,264584069	0,184473153
TMEM180	-0,264517525	0,455692012
ARHGAP4	-0,264396397	0,379297923
WDR3	-0,264038964	0,091630746
LOC729132	-0,263954345	0,831645059
OPRL1	-0,263653301	0,967352619
DMTF1	-0,263514444	0,312603568
NOL3	-0,263493745	0,669922922
TAF13	-0,263364747	0,763250927
C14orf143	-0,263346763	0,33771537
ZNF429	-0,263269281	0,851779219
hCG_1731871	-0,2631223	0,215265022
CCDC94	-0,263074876	0,452385065
GADD45G	-0,262523538	0,791718706
C9orf16	-0,262479158	0,952827098
LOC730281	-0,262470245	0,375633479
SIK2	-0,262423565	0,378887517
TLL2	-0,262255509	0,216450065
UBE2D2	-0,262229379	0,849194571
KLHDC7B	-0,262191629	0,618483747
WBSCR17	-0,262190631	0,468364536
LOC727826	-0,262039556	0,115146069
MYOZ1	-0,261924089	0,879285478
ZNF334	-0,261858801	0,095846968
TRY1	-0,261743043	0,449913827
LOC730367	-0,261699314	0,248954192
SLC17A6	-0,261629233	0,280264693

DAP3	-0,261626643	0,32354889
MED29	-0,261584576	0,164096907
PRDM13	-0,261558614	0,804380807
FAM181B	-0,261484827	0,500199956
SAC3D1	-0,261349983	0,087844034
SNX31	-0,261343509	0,06154487
LOC732363	-0,261246796	0,800867034
FAF2	-0,261160098	0,465051228
MVP	-0,261159218	0,439188239
KCTD13	-0,261115771	0,613402848
FAM59A	-0,261066737	0,909753711
MMP13	-0,260862616	0,12126553
C19orf50	-0,260769624	0,274380034
HSD11B1	-0,260457199	0,605281878
LOC388906	-0,260453688	0,74503983
FLJ37453	-0,260223958	0,10116361
GALGT	-0,26014323	0,598340069
HNRPAB	-0,259993465	0,178243617
OR5T3	-0,259857308	0,686230466
SENP8	-0,259811967	0,35342679
DNAJA2	-0,259789444	0,748605523
ONECUT1	-0,259782111	0,090696711
LOC648894	-0,25971261	0,124335396
LOC729088	-0,259665534	0,243260944
C14orf4	-0,259511175	0,078254195
GTF3A	-0,259300904	0,529228664
C21orf56	-0,259269886	0,397173066
LOC652164	-0,259012012	0,508726054
TNFSF8	-0,258780631	0,476388913
ADAM10	-0,258691979	0,132290891
LOC731399	-0,258638199	0,262513319
FABP4	-0,258498874	0,583518258
KLRD1	-0,258481656	0,150907313
SF3A1	-0,25835451	0,268836436
MED15	-0,258296335	0,195306882
CD7	-0,258248237	0,568324276
LOC731656	-0,257980444	0,617685742
SFT2D3	-0,257943444	0,456318522
SLC2A4	-0,257564098	0,249392285
FAM45B	-0,257558983	0,863235312
LOC731464	-0,257540918	0,008326955
NXN	-0,257526274	0,530539452
LOC653768	-0,257525135	0,608828747
KIAA1033	-0,2573927	0,135668059
FTH1	-0,257163104	0,9462748

PKIB	-0,257143612	0,893271012
TMEM63B	-0,257097602	0,193807316
MAP3K9	-0,25699841	0,730717512
C18orf24	-0,256969975	NA
R3HDML	-0,256946794	0,109392065
KIAA1239	-0,256878031	0,37294801
LOC728555	-0,256669135	0,793384123
LOC641763	-0,256606461	0,148340012
LCN2	-0,25650254	0,330388534
THRAP3	-0,256339727	0,288829805
CA5B	-0,256262411	0,124948124
CXCR6	-0,256250652	0,204162225
TBC1D24	-0,256237586	0,486238888
FLJ11011	-0,25620889	0,754825058
APBA2	-0,256158147	0,121871337
PPP2CB	-0,255979253	0,931019249
LOC728902	-0,25596891	0,519090612
SMA4	-0,255938818	0,861728821
LOC729600	-0,255909615	0,130268976
CXCR3	-0,255897011	0,114459534
TACSTD2	-0,255773134	0,289438226
CYP2D6	-0,255584193	0,152617137
FURIN	-0,255581241	0,152264259
RGL3	-0,255424172	0,355515963
PCDH17	-0,255374725	0,621851592
ACAS2L	-0,255286144	0,209257555
RHBDL4	-0,25502154	0,147894352
SLC5A6	-0,255019454	0,898122118
PQBP1	-0,254677127	0,318727743
A26B3	-0,254656713	0,204761396
PPP3CA	-0,25450902	0,280015036
PNUTL1	-0,254445424	0,70800916
FLJ23074	-0,25430917	0,435812585
NFE2L1	-0,253799735	0,723170915
RUFY3	-0,253732707	0,544540117
PVR	-0,253662636	0,092980902
HS3ST3A1	-0,253632371	0,161542057
ATOH1	-0,253548603	0,800292156
UGT2B4	-0,253340363	0,184109586
DFNA5	-0,253302897	0,740788991
ARHGAP11A	-0,252996779	0,782733969
PLCL2	-0,252800337	0,634803355
ARL10A	-0,252613731	0,835972644
WIZ	-0,252547004	0,083858846
FLJ32065	-0,252497804	0,529021298

MCL1	-0,252476765	0,260494368
SRP19	-0,252464436	0,420028714
HEXIM2	-0,252381587	0,4835189
LMO6	-0,252361172	0,50044693
INDOL1	-0,252313372	0,260203942
LOC441294	-0,252129775	0,231461007
LOC391059	-0,252124885	0,890922139
LOC389857	-0,252071431	0,647040393
PTPRZ1	-0,251780623	0,243059877
PDE8B	-0,251744763	0,065020132
SLC44A4	-0,251610835	0,325571169
COQ3	-0,25148732	0,063644965
LOC728614	-0,251478984	0,636576602
TMEM31	-0,251438573	0,310373192
LOC729332	-0,251074444	0,750478077
FAM161B	-0,25090632	0,731269815
LOC729309	-0,250897922	0,074264647
DLX2	-0,250809696	0,318463558
ZNF311	-0,250717904	0,164123582
GABRA4	-0,250675398	0,212093663
ASB6	-0,250434532	0,084053109
MRPL11	-0,250432968	0,127210316
CABP2	-0,250326463	0,092357598
LOC730517	-0,25032142	0,529276413
TAS2R42	-0,250246045	0,096255086
RPL11	-0,250206229	0,291004157
ATG3	-0,250075823	0,411520725
RPL37	-0,250013212	0,64464344
LOC728806	-0,249997087	0,312751365
LOC646902	-0,249983855	0,027161502
LOC647942	-0,249937896	0,320507438
HELZ	-0,249809034	0,51777462
CPLX4	-0,249769547	0,09032537
GAS8	-0,249762563	0,425536649
ENPP5	-0,249567578	0,483611704
OCIAD1	-0,249526116	0,154212943
CALR	-0,249509642	0,86953685
RASGEF1A	-0,24927401	0,70971833
PRDX3	-0,249158818	0,118197548
COBRA1	-0,249155375	0,575150659
FHOD1	-0,24914171	0,089121449
TMEM217	-0,249096546	0,157541175
ZNF829	-0,249054327	0,377966034
PCDHB6	-0,249033369	0,051522935
LOC730398	-0,248963774	0,777836273

RASSF8	-0,248840328	0,928390764
OC90	-0,248768694	0,311025618
LATS1	-0,248046282	0,560543015
MGC15429	-0,248039859	0,508338761
CHCHD3	-0,247868546	0,002855855
LOC644994	-0,247839625	0,022484882
LOC731246	-0,247731264	0,955966049
LOC641766	-0,247717835	0,490974954
PARG	-0,247661468	0,611474264
NARFL	-0,247551262	0,92531134
CYP4X1	-0,247504958	0,981326586
KCNH4	-0,2474849	0,528871577
ZWILCH	-0,247454732	0,25070337
FLJ25404	-0,246966452	0,506905223
PRDX1	-0,246948202	0,094021314
LTB4R2	-0,246927826	0,387686163
PIK3CB	-0,246881538	0,016784252
LOC152118	-0,24684371	0,299288192
SLAMF6	-0,246818657	0,637256087
RYR3	-0,246803066	0,233961112
FPRL1	-0,246766328	0,958399326
SARA1	-0,246715999	0,187489885
TPMT	-0,246604603	0,339053041
ZNF263	-0,246596225	0,912740836
C4orf33	-0,246556717	0,596937887
FGD2	-0,246529601	0,757801507
ZNF540	-0,246225277	0,510300404
CDCP1	-0,245899301	0,085099708
MRRF	-0,245837516	0,281719849
PTGS2	-0,245742035	0,788999038
PEX12	-0,245738473	0,921652594
SH3GL3	-0,245694058	0,584716417
MK-STYX	-0,245564847	0,223173698
LOC730012	-0,245553969	0,552146972
TMEM220	-0,24537825	0,030123095
PPFIA2	-0,245342271	0,618686599
C10orf54	-0,245043076	0,848515566
ZNF418	-0,245019073	0,950992088
LOC643377	-0,244965686	0,13121914
PTDSR	-0,244843435	0,981799734
LOC642587	-0,244840999	0,221685057
HNRPDL	-0,244819046	0,457218034
PACE-1	-0,244813505	0,330703069
KCNMB3	-0,244572377	0,963747585
HEXA	-0,24450644	0,853765525

MGC34923	-0,244439253	0,617475675
LOC645323	-0,244273627	0,06213088
TNFRSF10D	-0,244250623	0,061626883
ZNF281	-0,24417923	0,425460002
C19orf24	-0,244159385	0,673603734
TIGD1	-0,24401841	0,079871683
TRIM31	-0,244001797	0,652393329
PCDHGA9	-0,24399264	0,131178772
ZCCHC16	-0,243757224	0,063339755
HOXA7	-0,243749862	0,839353563
MGC70870	-0,243685784	0,337278843
CEACAM18	-0,243459938	0,414496412
OR12D2	-0,243435524	0,300638883
PSG1	-0,243382942	0,264732479
LYL1	-0,243285728	0,46803401
RNF125	-0,243262168	0,092572167
LOC731038	-0,242758141	0,057962707
LOC729421	-0,242693524	0,265581707
USP48	-0,242633057	0,069995326
C14orf102	-0,242592127	0,157332312
PASK	-0,242534293	0,249054439
HAVCR2	-0,242507965	0,958801173
MARS	-0,242485948	0,140399253
C7orf28B	-0,242387717	0,296121088
GPR151	-0,242384588	0,633400283
MTFR1	-0,242323324	0,30511591
CALCOCO2	-0,242261759	0,702302185
HSD17B1	-0,242120521	0,490151655
L3MBTL3	-0,242095696	0,151736708
ATP6V1A	-0,242044519	0,503888288
C7orf42	-0,241826706	0,494670982
SCN3A	-0,241613681	0,187703541
SLC2A7	-0,241520956	0,616401532
RBM13	-0,241481635	0,21943923
ORAI3	-0,241447083	0,410899927
FLJ78302	-0,24136162	0,289548681
NUDCD2	-0,241318138	0,124400944
LOC341651	-0,241180977	0,504019265
PFN1	-0,241171844	0,270239091
ADAMTS12	-0,241138873	0,650634132
PSD4	-0,241047748	0,389059148
LOC728543	-0,241023571	0,7035966
C20orf59	-0,241022455	0,48907981
SIAT8D	-0,241012398	0,325003007
ENPP3	-0,240738668	0,054955263

TRIB1	-0,240719386	0,13271735
HABP2	-0,240369125	0,271993501
TBX2	-0,240343829	0,242349039
AQP1	-0,240148565	0,044467273
PIPPIN	-0,240080082	0,176234579
CRYGS	-0,239883159	0,263461261
MIA3	-0,239824966	0,116272501
TNFSF10	-0,239811676	0,167093693
G3BP2	-0,239774786	0,828519366
IFI27	-0,239706489	0,165934388
NES	-0,239649359	0,275201276
FLJ31033	-0,239479063	0,324788876
LOC390669	-0,239393511	0,18003369
COL5A1	-0,239314249	0,822211755
HSF4	-0,239259246	0,308302184
LOC729319	-0,239175375	0,154244261
NUDT2	-0,239149567	0,060980175
TMEM2	-0,238935973	0,249927886
RAB28	-0,238845078	0,454422748
hCG_1790950	-0,238819845	0,074500516
DVL2	-0,238698198	0,774599536
LOC652869	-0,238601733	0,695072247
ANGPT1	-0,238423229	0,705288546
DHRS2	-0,238203577	0,771382476
RHBDD3	-0,23810871	0,330167382
C6orf173	-0,238077128	NA
RSL1D1	-0,238002793	0,059511175
MAPT	-0,237935926	0,164184551
FAM83C	-0,237881942	0,461271179
IGSF9B	-0,237880867	0,103419153
MAPKAPK5	-0,237852406	0,230860223
ALDH2	-0,237779582	0,290297594
FSTL5	-0,237757375	0,261892889
PGBD4	-0,237644165	0,227314017
LOC729199	-0,237495617	0,744874549
March10	-0,237433745	0,600145823
KLHL22	-0,237164906	0,130724944
LSM1	-0,23701993	0,289429694
MUC3B	-0,236966252	0,54231732
DNAJC1	-0,23690726	0,958991342
ARHGAP12	-0,236878982	0,211126607
AURKC	-0,23681938	0,97543089
HOXB4	-0,236795842	0,981013982
PLS1	-0,236723351	0,463766485
MED8	-0,236068256	0,241767079

OR4C6	-0,236025228	0,268399462
PECAM1	-0,235898306	0,665523539
LOC729288	-0,235777529	0,823011022
GNAI1	-0,235643055	0,868885278
ARHGEF17	-0,235486842	0,317321468
TRPS1	-0,235419384	0,711760506
OTUD3	-0,235336064	0,096706025
CYP21A2	-0,235310622	0,539722258
CLEC1B	-0,235268564	0,153053682
NGFB	-0,235186574	0,856686357
C9orf117	-0,235102349	0,13724738
SLC30A4	-0,234947944	0,518162622
FLRT3	-0,234646667	0,901478878
EBI3	-0,234467505	0,175428004
C11orf57	-0,234240675	0,211891533
MAP3K7	-0,234237383	0,569214386
SHBG	-0,234169736	0,175607747
STAG2	-0,234028861	0,231291565
DOCK1	-0,233958746	0,656922215
UTP20	-0,233860844	0,148519695
KRAS2	-0,233714913	0,672761203
KCNK3	-0,233662176	0,952945058
ADIPOR2	-0,233594124	0,577115483
ULBP1	-0,23329902	0,26069763
K-ALPHA-1	-0,233154456	0,732418221
LOC124220	-0,233116935	0,4772148
ZNF232	-0,232833439	0,165661124
MTERFD3	-0,232832369	0,361809941
BID	-0,232824814	0,286251658
SRP72	-0,232817918	0,36556793
LOC643714	-0,232774259	0,204495783
FZD1	-0,232716196	0,328493133
C6orf204	-0,232682009	0,659845093
GABRQ	-0,232675294	0,403162646
PCBP2	-0,232623551	0,26954039
PCDH8	-0,232608655	0,129318827
ANKFN1	-0,232342668	0,186328591
FAM20A	-0,232321279	0,187629719
TBC1D15	-0,232304501	0,386939694
FLJ11196	-0,23229201	0,816613784
PAPD4	-0,232241475	0,498587146
ZNF20	-0,232102899	0,338071
hCG_1730474	-0,232056915	0,889793435
MRPL21	-0,231990195	0,25438167
PLEKHA1	-0,231954084	0,090006489

PPIL2	-0,231937819	0,47400325
CARTPT	-0,231902318	0,824283789
MMP2	-0,231799959	0,015628679
LAMB1	-0,231746655	0,195648742
NUCB1	-0,231708415	0,217015911
MPO	-0,231679842	0,267905888
CCDC46	-0,231679202	0,17211889
SCAF1	-0,231678352	0,281392839
MIR16	-0,231597812	0,961706729
MRPS30	-0,231428165	0,156137375
CYGB	-0,231321734	0,083767976
PRAMEF7	-0,231199828	0,489197242
ZNF141	-0,231026956	0,09668849
TSHR	-0,230862514	0,239974325
CCDC132	-0,230731307	0,291128454
WDR38	-0,230723916	0,553444215
FHDC1	-0,230701306	0,726393021
SERHL	-0,230692375	0,167051677
LOC652767	-0,230604196	0,792766758
OR6C1	-0,230521562	0,057330532
PCDHA11	-0,230475528	0,453294434
OR2W1	-0,23044764	0,469421807
C5orf35	-0,230430827	0,400427261
SSTR5	-0,230366541	0,749505569
MGST1	-0,2303521	0,709832352
SYNGAP1	-0,230230556	0,233949774
FLJ36644	-0,230212964	0,518247815
LOC728738	-0,230176485	0,147292763
PTEN	-0,230160137	0,87097093
BRD7	-0,23002151	0,402323165
NRTN	-0,229826077	0,233539695
LOC730236	-0,229809686	0,163300593
GSK3B	-0,229603174	0,185623365
MRPS34	-0,22959224	0,554332128
TESK2	-0,229462272	0,669744227
MGC11308	-0,229453177	0,037913462
FLJ13352	-0,229412209	0,217987505
FBXW5	-0,229352907	0,486514364
ZFAND3	-0,229298881	0,151293549
LONRF2	-0,229246923	0,257460456
PSMB4	-0,229174458	0,309277994
LOC729169	-0,228994897	0,040463197
ANKRD34C	-0,228958912	0,855229853
FARS1	-0,228894341	0,265120405
ABRA	-0,228818873	0,144596761

MAP2K1	-0,228742026	0,266322494
LOC645212	-0,228598227	0,401817585
RAB25	-0,228539608	0,34217712
EGR4	-0,228367008	0,684561417
FAM134A	-0,228356979	0,691774249
SNAPC1	-0,228237863	0,390185326
BRD2	-0,22808607	0,373145038
TRAPPC6A	-0,227984224	0,095423402
C21orf57	-0,227958345	0,431244399
C6orf105	-0,227813677	0,061292868
SYNGR1	-0,227624286	0,138833668
LOC728416	-0,227412135	0,366548747
LOC729566	-0,227285769	0,818575671
TSSC1	-0,227125436	0,721797812
TAS2R1	-0,227066705	0,094597903
JMY	-0,227060589	0,987057808
LOC389117	-0,226979636	0,644193586
hCG_2026038	-0,226902733	0,46041236
LOC340515	-0,226850851	0,544448414
CHSY2	-0,226793956	0,385432702
C1orf49	-0,226691104	0,28664927
M96	-0,22664718	0,144131122
ABCG5	-0,226271474	0,231429965
ATP4A	-0,226254349	0,091048166
AMBN	-0,226223007	0,127367617
PTGER4	-0,226164057	0,859879985
VAPA	-0,226127824	0,584685053
PROX1	-0,22583654	0,26076229
PDE7B	-0,225713042	0,083174844
RNF6	-0,225667783	0,584086099
PIM1	-0,225665983	0,523850583
POP4	-0,225408595	0,887219576
CASKIN2	-0,225270677	0,639106872
BIK	-0,225210104	0,862602262
CPNE7	-0,225199795	0,170284425
FLJ40448	-0,225102443	0,041310807
ITFG3	-0,224985718	0,067686703
SOS1	-0,224984972	0,724924939
PLCD4	-0,2249735	0,608230826
hCG_2045804	-0,224648101	0,256902819
FAIM2	-0,22450772	0,56510795
LOC730273	-0,224473743	0,66139433
C10orf104	-0,22427203	0,879381043
SERPINF2	-0,224238866	0,490574296
EMILIN1	-0,223971946	0,522236458

SLC38A2	-0,223915119	0,277166509
C20orf196	-0,223760256	0,1340986
LOC728792	-0,223687982	0,522836379
E2F5	-0,223604249	0,55291639
LIG3	-0,223590789	0,416185432
C3orf17	-0,223155708	0,368072988
AGT	-0,223098671	0,97774519
UVRAG	-0,223039222	0,667755483
CLDN1	-0,223028166	0,732482188
MRPL2	-0,222916741	0,115784648
TRADD	-0,222889165	0,535357204
IQUB	-0,222880851	0,108609823
LOC730727	-0,222795872	0,928189545
LRIT2	-0,222784326	0,138394309
OAS1	-0,222398221	0,710426459
TOP2B	-0,222336815	0,947067602
ZNFN1A2	-0,222282878	0,135702113
TMEM199	-0,222268395	0,633284302
C8orf68	-0,222235352	0,492721466
LRRC40	-0,222195046	0,74836248
LOC642580	-0,222127594	0,791517464
CGI-63	-0,221755568	0,239975571
LOC731758	-0,221711489	0,853225894
LOC729854	-0,221585017	0,16603495
KIF12	-0,221517836	0,71810244
LY6E	-0,221394872	0,657823724
ZNF397	-0,221288523	0,500228441
FCGR2B	-0,221202876	0,904866621
ITM2A	-0,221192704	0,622550881
RAB1B	-0,221164073	0,944201622
LOC727971	-0,221069015	0,924988588
IMPG1	-0,220978888	0,719049431
MOBKL2C	-0,220892125	0,189229295
CCDC57	-0,220718526	0,683303469
SCN11A	-0,22071363	0,982079915
MAP1D	-0,220658771	0,855241824
TUSC1	-0,220621004	0,129959777
DYSFIP1	-0,220360471	0,673689311
KBTBD2	-0,220214948	0,918307039
SH3GL1	-0,220174653	0,366168194
KIAA0992	-0,220051934	0,207352112
DNASE1L3	-0,220019702	0,826592986
GHITM	-0,219926564	0,129225393
MDM1	-0,219768313	0,271849235
LOC727775	-0,21976059	0,270044061

hCG_2007354	-0,219666734	0,019286746
WHDC1	-0,219520698	0,941813098
AQP10	-0,219461014	0,377356112
CCDC114	-0,219337394	0,146057228
C20orf111	-0,219176326	0,311430134
ADFP	-0,218990479	0,780654684
PTCRA	-0,218964376	0,974395846
H2AFY2	-0,218952297	0,693994236
LOC731465	-0,218900257	0,113086426
ZNF474	-0,218667402	0,966681921
FGD5	-0,218614384	0,167297745
SLC4A1	-0,218339934	0,703854496
LOC285733	-0,218171494	0,128068447
LGALS12	-0,218149447	0,442895729
GALNT3	-0,218034655	0,323863886
EPS8L2	-0,217830328	0,950676792
APEX1	-0,217817926	0,923496652
TSPYL4	-0,217486643	0,176189387
RNF3	-0,217428422	0,843890829
KIR2DL4	-0,217428127	0,309651813
SEMA6C	-0,217413694	0,220282343
LOC645527	-0,217406372	0,162152713
ERBB3	-0,217375058	0,400555534
SYT17	-0,21734431	0,536944003
KRTHA7	-0,217317705	0,178915813
LOC727873	-0,217167764	0,417639897
LOC647259	-0,217166514	0,15324759
EMR1	-0,217142211	0,458170179
PHKA1	-0,217140125	0,37974507
ZFP29	-0,216982466	0,170487401
SP5	-0,216389361	0,229060396
TSARG6	-0,216242256	0,0789293
ACVR1	-0,215979895	0,032307645
VMAC	-0,215860316	0,266387357
RPSAP	-0,21570507	0,780893073
C19orf42	-0,215577502	0,09132976
RING1	-0,215458073	0,418184315
SLC24A4	-0,215355572	0,209695367
GABRA2	-0,215312663	0,791487709
C7orf46	-0,215145569	0,927831163
IL1RAPL1	-0,215050924	0,816687411
GCNT1	-0,215048749	0,88691437
ZNF563	-0,215017903	0,269371027
CCDC74A	-0,214977132	0,073802842
LOC51233	-0,214928656	0,093150884

FLJ00038	-0,214911159	0,40313515
LOC387927	-0,214899335	0,050430501
CPNE3	-0,21489687	0,161202609
LOC391767	-0,214872789	0,845288721
OR2A12	-0,21481789	0,425985474
PLA2G12A	-0,214753316	0,128652795
LOC642874	-0,214645883	0,548074977
FLJ14753	-0,21457098	0,180797296
TRIM71	-0,214486891	0,876395732
CHIC1	-0,214462125	0,397183725
UGT8	-0,214436362	0,338767874
LOC729953	-0,214415774	0,931358056
BATF	-0,214337253	0,929374305
DNAH12L	-0,214322066	0,705387799
CCDC21	-0,214309929	0,144060746
TEKT5	-0,214297026	0,324712708
USP26	-0,214270191	0,208629625
PIP5K3	-0,214252686	0,247754089
TSPAN12	-0,214192588	0,089358403
CXorf58	-0,214148034	0,140811047
C2orf62	-0,214121383	0,898546771
GNAT1	-0,214118999	0,064843629
HLA-DMA	-0,21410137	0,921993784
IL7	-0,213948089	0,293994339
LOC646895	-0,213722477	0,981413164
DNAJA1	-0,213711677	0,104047779
VPS33A	-0,213643932	0,679459495
ZFR2	-0,213362446	0,186712971
SETMAR	-0,213233858	0,562683505
CPA6	-0,213048233	0,925492768
RPS29	-0,213041942	0,50317465
LOC646184	-0,21295685	0,292800236
SAMD1	-0,21292209	0,934429667
HTR1A	-0,212862767	0,133419865
KIAA0892	-0,212851924	0,356004793
PAWR	-0,212797139	0,505018366
CAPS2	-0,212695546	0,195434468
MGC4083	-0,212482925	0,181389575
KIAA1001	-0,212465026	0,508989352
DNALI1	-0,212401822	0,837040635
PAIP2	-0,212391018	0,727686648
BBS2	-0,212360976	0,178267846
RAB9B	-0,21230638	0,026667976
LOC283331	-0,212258752	0,782029013
CNTD1	-0,212124776	0,157128223

GPR45	-0,212109683	0,354836492
SELT	-0,212039798	0,24388523
C21orf51	-0,211984267	0,348037523
PANK1	-0,211915284	0,462028857
ZNF574	-0,211708747	0,259082326
OR52R1	-0,211695785	0,637930974
TSPYL6	-0,211582417	0,401628284
IMP-3	-0,211350451	0,189746399
LOC731561	-0,211253114	0,213421766
AQP12A	-0,21121186	0,211733991
LOC732413	-0,211167213	0,327605254
FATJ	-0,211127605	0,198287263
LOC283788	-0,210878968	0,364933445
DOK2	-0,210735086	0,094724887
LOC731231	-0,210721741	0,788942307
CSNK1A1	-0,210689289	0,618903885
CDK10	-0,21055882	0,315502563
HMX3	-0,210535323	0,428365773
VIT	-0,210494088	0,146535842
UQCRB	-0,210419091	0,967851669
LOC729823	-0,210378521	0,610162397
LOC729913	-0,210350289	0,419612727
TTPA	-0,210317029	0,562636496
SPPL2A	-0,210311447	0,227566577
NLRP1	-0,209977139	0,10355255
GPR180	-0,20996691	0,446046297
LOC388002	-0,209744647	0,95649154
UNC84B	-0,209699033	0,598072549
hCG_2003956	-0,209693297	0,187285102
LOC644070	-0,209665403	0,54324889
C9orf5	-0,209461206	0,697326009
SHC3	-0,209356898	0,132048473
LY6G5B	-0,209241304	0,411388913
C10orf28	-0,209040823	0,676885872
MRPL41	-0,209037571	0,747700325
TMEM22	-0,208885226	0,136177871
FLCN	-0,208860646	0,435773586
MIF	-0,208825449	0,7811474
ABHD12	-0,208724567	0,388496211
SATB2	-0,208667869	0,964709996
OR4S2	-0,208607152	0,439641186
PAPD1	-0,208503553	0,128013831
LOC643441	-0,208399557	0,760219972
LPXN	-0,208377376	0,487039045
FLJ32756	-0,208343343	0,622462006

ITPRIPL1	-0,208298457	0,921260942
LOC340286	-0,208284441	0,471233954
SPATA8	-0,208162009	0,073019134
CPXM2	-0,207705673	0,89490131
KRTAP19-3	-0,207647182	0,929181175
LOC646252	-0,207442903	0,116555095
ANKRD24	-0,207411073	0,469507815
POGZ	-0,20740562	0,400706947
PAX9	-0,207383888	0,190469734
NRXN3	-0,207328185	0,64971456
LUM	-0,20731302	0,89548421
CAPRIN2	-0,207016391	0,258056848
C1orf14	-0,206971532	0,585757689
C1S	-0,206967384	0,264598626
RAB33A	-0,206727041	0,186539464
LOC729690	-0,20665306	0,143574601
VPS13C	-0,206576116	0,790608661
CTRC	-0,206424887	0,3230981
CSTF2T	-0,206258723	0,303856655
MAPK8IP2	-0,206233359	0,949469589
GRP58	-0,206187301	0,813085968
SDS	-0,206089665	0,623663726
LOC441662	-0,205955791	0,736591234
LOC651732	-0,205596397	0,532969776
AIPL1	-0,205571879	0,789146528
HRMT1L3	-0,205540037	0,18469606
LOC651507	-0,205516178	0,563274176
ARL11	-0,205430813	0,617413187
GDF8	-0,205404264	0,356741395
NOC3L	-0,205285335	0,060040165
KIAA1737	-0,205139535	0,146227478
UBL5	-0,205081224	0,100887959
LOC647194	-0,205066023	0,532502776
LTB4DH	-0,204951075	0,063150737
SLC15A2	-0,204911648	0,917010532
TMEM16F	-0,204904647	0,187076531
CALML5	-0,204865578	0,314823967
LOC650200	-0,204826748	0,963890241
KCNN3	-0,204744872	0,156971017
LOC441150	-0,204701368	0,90956456
C16orf46	-0,204665442	0,199105078
FCGR2A	-0,204633493	0,886006675
LOC730053	-0,204572017	0,629802174
RIOK3	-0,204570281	0,440735579
PGRMC2	-0,204502606	0,761677213

NR1D2	-0,204460872	0,470562817
GOLT1A	-0,204346701	0,507507384
STK19	-0,204346104	0,165935317
LOC642956	-0,2041296	0,349925752
RASAL2	-0,204110717	0,94298761
BRF2	-0,204084501	0,393080954
LOC145853	-0,204081447	0,958340301
LOC283902	-0,204079238	0,153475393
DNAJC25	-0,203839765	0,900947899
LOC730775	-0,203697258	0,598641903
LHFP	-0,203690144	0,149585307
LOC388963	-0,203573313	0,169664465
KCTD4	-0,203476472	0,717833251
NEUROG2	-0,203221193	0,172203437
SPAG4	-0,20308859	0,384228387
UGT2B10	-0,203068793	0,674143531
FLG	-0,202955515	0,380577465
ARHI	-0,202907208	0,22439528
PGLYRP3	-0,202732576	0,948050923
BSPRY	-0,202559017	0,956738806
FANCI	-0,202556844	0,187138836
LOC645851	-0,202473057	0,182401158
LMF1	-0,202278419	0,157901564
LOC731450	-0,202268042	0,06556842
TCF21	-0,202209205	0,306122214
IRS2	-0,202111625	0,923647612
ITGAL	-0,202008116	0,838022227
NCAPG	-0,201798097	0,862295718
C10orf119	-0,201667517	0,153732843
TWIST1	-0,201574961	0,872789647
DNAJC9	-0,201545372	0,42777588
BCL11A	-0,201524551	0,318823886
OR11H1	-0,201371733	0,21661486
RBM20	-0,201371304	0,195505422
SLC38A4	-0,201256243	0,151227311
WDR52	-0,201255405	0,8837859
WIPF1	-0,201144653	0,905765084
SPATA9	-0,201112357	0,540585186
C2orf77	-0,201080436	0,621611391
LOC729083	-0,200707522	0,148967571
CUGBP1	-0,200578125	0,938831144
SMURF2	-0,200575568	0,842221385
DRD2	-0,2005696	0,558570105
DLG5	-0,200443248	0,997397134
GOLGB1	-0,200383429	0,605552872

FLJ90661	-0,200306488	0,877456051
REG3A	-0,200239772	0,180032554
LOC402160	-0,200154981	0,788969749
DEFB111	-0,200134738	0,508816696
EOMES	-0,20005036	0,656961733
CPO	-0,199693715	0,186474937
TBX18	-0,19952248	0,368438606
LOC648742	-0,199437138	0,415146985
GCLC	-0,199375221	0,243061098
BCL2L11	-0,199373228	0,25220049
SP4	-0,199359915	0,305651118
FMO5	-0,199295731	0,410823903
ACTC	-0,199208066	0,818371364
FLJ37451	-0,199151302	0,673646206
SGK	-0,199079845	0,304994497
LOC729699	-0,19894859	0,217114131
DKFZp779B1634	-0,198916684	0,076727596
RNF135	-0,198900452	0,901859062
ESM1	-0,198620618	0,475296498
BTK	-0,198568108	0,633065756
RAB24	-0,198522334	0,103391794
ADRB1	-0,198377176	0,963671839
ALDOB	-0,198254589	0,301573868
PAFAH1B2	-0,198204547	0,203782648
GULP1	-0,198146385	0,410365536
NCKAP1	-0,198045329	0,268410596
MTMR6	-0,197875979	0,297500641
FOXP4	-0,197873264	0,651781517
DMRT3	-0,197852538	0,653203718
LOC728288	-0,197516402	0,426915602
HIPK1	-0,19749183	0,143965552
KIAA1712	-0,197405174	0,260420875
DEGS	-0,197403833	0,613660565
LOC653904	-0,197401333	0,392719898
LOC643382	-0,197327505	0,133178144
LOC729616	-0,19732581	0,188516244
SNIP1	-0,197290416	0,145166555
TMOD1	-0,197254087	0,292650174
GAP43	-0,197237254	0,238238841
ZNF74	-0,196939805	0,78692369
LOC731591	-0,196837607	0,363308534
RSPO4	-0,196815225	0,629620763
ZNF509	-0,196770349	0,985747229
LOC643194	-0,196629732	0,279819207
NEK3	-0,196605029	0,939449056

OTOF	-0,196512752	0,094951462
CAPN6	-0,196495798	0,976850186
TMEM63C	-0,196494817	0,603422399
TMCO5A	-0,196443091	0,131296888
NFAT5	-0,196429352	0,811904366
POLDIP2	-0,196346153	0,307645576
LAK	-0,196337517	0,629715359
DKFZP434N1235	-0,196328353	0,33014019
HES2	-0,196295338	0,313186236
LRRC6	-0,196286858	0,229972624
KCNA4	-0,196196101	0,249195257
TNFAIP8L2	-0,196126007	0,74068126
TMEM41B	-0,196117477	0,370620513
LOC283804	-0,195997965	0,158243954
CYB5-M	-0,195842009	0,194707851
DNMT3L	-0,195837436	0,534310645
IDH2	-0,195830338	0,898347821
SLC12A5	-0,195696295	0,423701641
LOC642018	-0,195485476	0,013005783
FCGRT	-0,195356487	0,226837991
VMA21	-0,195325925	0,244781222
MYH4	-0,195262394	0,937868935
ALKBH7	-0,195151597	0,202359879
ZNF271	-0,195132166	0,126811829
PTF1A	-0,195113541	0,84184396
TLR1	-0,195037434	0,338278074
LRRFIP2	-0,194916033	0,967474716
POPDC3	-0,194871322	0,168115968
STEAP3	-0,194861932	0,209707621
LOC652251	-0,194796958	0,842596868
LOC732146	-0,194756599	0,738170919
LOC647286	-0,194656527	0,622228646
FLJ13052	-0,194534919	0,480724395
ZNF443	-0,194379858	0,872798075
HEPACAM2	-0,194271154	0,759260216
SH3BGRL2	-0,19420497	0,308476555
SPATA2L	-0,194203762	0,809041819
RSN	-0,194095613	0,783883297
MID1	-0,194075035	0,22999096
FKBP14	-0,194053652	0,909575432
ZMAT1	-0,194006527	0,240756341
KIAA0467	-0,193963573	0,245116263
TTC27	-0,193920629	0,931265986
KATNAL1	-0,193852919	0,475250233
OLFML3	-0,193830787	0,370675202

SLC33A1	-0,193815746	0,882701491
BCL2L12	-0,193760975	0,137024001
GPR100	-0,19366531	0,346116564
SSBP3	-0,193606822	0,691457385
LOC253970	-0,193582906	0,226101806
KDELC2	-0,193554645	0,381640535
TRIM44	-0,19354078	0,397759954
SKP1A	-0,193412193	0,542200259
C6orf115	-0,193368783	0,308994599
RHOD	-0,193198278	0,767080384
RAB6B	-0,193196542	0,36207983
WDR48	-0,193171543	0,304627926
LOC642262	-0,193144693	0,190952058
NRSN2	-0,193096151	0,827383667
WDR40A	-0,193082478	0,327795314
LOC728287	-0,192994438	0,083839542
GABARAP	-0,192667216	0,238871736
GLI3	-0,19263071	0,896154504
PGDS	-0,192357229	0,487275174
NSL1	-0,19214295	0,339998152
MGC33407	-0,192133623	0,1162044
NYD-SP25	-0,192082645	0,439399558
ACTBL2	-0,192070991	0,420203767
HS3ST2	-0,192031807	0,253275656
C3orf16	-0,191760503	0,83089354
hCG_25371	-0,191737401	0,317628417
C20orf117	-0,191629505	0,1707754
THUMPD3	-0,191542987	0,85870662
NLRP7	-0,191521858	0,480841911
ABCC6P1	-0,191443392	0,543965948
MYCT1	-0,191186577	0,39856693
SAMD9L	-0,191163714	0,141214014
TBPL1	-0,191074696	0,137973478
AFF2	-0,19085229	0,810343615
ADAMTSL1	-0,190804194	0,229848548
ZNF282	-0,190707145	0,786893948
ZDBF2	-0,190622487	0,145629539
ABHD2	-0,190552445	0,219616194
RAB20	-0,190550692	0,749878836
NUDT14	-0,190349599	0,173524407
RDH-E2	-0,190245196	0,233499929
AFMID	-0,190174058	0,484815268
ZNF816B	-0,190120632	0,403692616
PRRT1	-0,190014696	0,49625914
CCNG1	-0,189933333	0,960265011

CLK4	-0,189808261	0,991859743
LOC729446	-0,189730451	0,302393455
TAF15	-0,189567169	0,285668859
CNOT3	-0,189415311	0,174553282
LOC643052	-0,189389727	0,186168214
LOC646268	-0,189229989	0,771077279
TERF2	-0,189215579	0,396963427
ABHD10	-0,189191073	0,403367703
P101-PI3K	-0,1891416	0,563272051
JAGN1	-0,189099984	0,745778961
LOC728058	-0,188827832	0,269194222
MRPL33	-0,188644928	0,703574915
VAPB	-0,188593204	0,065689216
DNAJC3	-0,18849092	0,135969148
GALNACT-2	-0,188468893	0,578117035
DHX57	-0,188370186	0,654270817
ADAMTS6	-0,18829455	0,519187998
HK1	-0,188272629	0,414654292
OR10G2	-0,188130064	0,397474683
SEMG2	-0,188039263	0,396062267
OR52A5	-0,187978462	0,120278071
CFC1	-0,187960463	0,943121325
SEZ6	-0,187926431	0,585211324
LOC728249	-0,187925769	0,75566919
GTF2F2	-0,187799328	0,893113138
RCCD1	-0,187523216	0,211056434
KIAA1009	-0,187482825	0,354550362
ALG5	-0,187381536	0,202465141
GRP	-0,187374437	0,255009423
PECI	-0,187277442	0,115573447
SELK	-0,187231193	0,66941758
JPH3	-0,187202536	0,838166802
TRHDE	-0,187145365	0,492883326
KRTAP19-6	-0,187096452	0,5449896
LOC51061	-0,187088001	0,914072898
INPP1	-0,187043074	0,265957346
MAPK8IP1	-0,186945977	0,249512432
LOC284964	-0,186928134	0,847963872
LOC645691	-0,186888464	0,910891948
CDR1	-0,186845719	0,943527355
C2orf34	-0,186736565	0,218993382
DNASE2	-0,186712572	0,909499307
OR2AG1	-0,18663461	0,46148993
CLDN4	-0,186622927	0,458849838
LOC643073	-0,186555213	0,980332328

HMP19	-0,186448514	0,908904092
LOC441915	-0,186400724	0,15825312
LOC340947	-0,186391658	0,148482862
C14orf121	-0,186337067	NA
SNTB1	-0,186168642	0,440078786
THSD7B	-0,186086364	0,900906981
GRK7	-0,18605332	0,345990709
KIAA1975	-0,186046329	0,215849712
LOC729651	-0,186039049	0,377382087
MYO1F	-0,185945114	0,214832389
CASQ1	-0,185748865	0,883487255
MGC39900	-0,185639657	0,37565944
LYZL6	-0,185601759	0,102620371
PLEKHG3	-0,185520163	0,531710963
MAGEE1	-0,185502616	0,426789463
NUP188	-0,185410553	0,139908811
JUND	-0,185129976	0,069740902
CCDC115	-0,185021213	0,98931804
DGKI	-0,184985959	0,868851771
LOC729653	-0,184941168	0,415828877
TAL1	-0,18490579	0,11316107
OR2C3	-0,184655137	0,861880074
MLL5	-0,184518935	0,132259118
KIAA1765	-0,18448883	0,446058883
SLC35F4	-0,18448618	0,064674786
LOC732431	-0,184464331	0,053339914
HPCA	-0,184286123	0,97094608
PROS1	-0,184247508	0,301682242
PFKL	-0,184244644	0,496491986
LOC283904	-0,184046062	0,165022069
MDM4	-0,184045279	0,527331968
C17orf79	-0,183980427	0,071103808
ALOX15B	-0,183805635	0,389587832
LOC644691	-0,183714793	0,496356536
WDR42C	-0,183705976	0,216446392
LOC729614	-0,183653349	0,765548178
DCTN3	-0,183605219	0,18064255
AGTPBP1	-0,183596679	0,269627885
PHYHIP	-0,183592017	0,66160673
VDAC1	-0,183567423	0,321323666
LOC390998	-0,183523801	0,449824167
DPM3	-0,183355619	0,285694594
LOC732415	-0,183248598	0,21150527
NDUFV2	-0,183195417	0,666907553
GPR173	-0,183096848	0,169556525

HAPLN4	-0,18308903	0,54400982
C4orf39	-0,183073465	0,280783102
FBXO45	-0,18302653	0,752699078
THRAP1	-0,182972754	0,106130762
LOC730421	-0,182923804	0,350894731
OR4K5	-0,182790865	0,737407198
METTL3	-0,182676545	0,720678054
PDS5B	-0,182672027	0,116764671
GAL7	-0,182666329	0,137704708
LOC439962	-0,182604598	0,866462707
HRNR	-0,182509886	0,301521628
IHPK2	-0,182274795	0,179435036
LOC729093	-0,182141139	0,144141269
ACTR6	-0,18208256	0,578303075
LOC440368	-0,182054158	0,758070652
GJB2	-0,181972721	0,951413124
CETN3	-0,181942848	0,924257357
ZNF143	-0,181935414	0,173889964
ELMOD1	-0,181820509	0,298554117
NR2E1	-0,181755775	0,22320762
LEF1	-0,181643385	0,501159927
RHOQ	-0,18161134	0,693736302
SYCP1	-0,181536697	0,05839596
SELM	-0,181402565	0,162128133
OR12D3	-0,181394384	0,974402746
EMR2	-0,18122236	0,152681718
ZNF226	-0,181105031	0,068959386
CXCL5	-0,181094499	0,138040175
G6PC	-0,181091502	0,584469207
LOC728975	-0,1809547	0,110734781
ZNRF4	-0,180930818	0,417923112
GRB2	-0,180900252	0,293558416
SMAD1	-0,180874189	0,769623486
CBY3	-0,180756391	0,336265984
FAT	-0,18070127	0,587810606
CCM2	-0,180561447	0,888001817
CARD4	-0,180488905	0,495226748
LOC646043	-0,180445909	0,512938391
SLC45A2	-0,180433141	0,688786435
LOC649236	-0,180258982	0,691520618
C12orf50	-0,180172628	0,107994816
TSPAN6	-0,180113232	0,550231184
UBXN11	-0,17991952	0,863808212
SLC39A4	-0,179860353	0,213710977
GUK1	-0,179851382	0,442207753

ECD	-0,17960264	0,672050397
ZNF557	-0,179493994	0,47822655
FLJ16139	-0,179047026	0,486981494
PRDM6	-0,179010597	0,401508717
C18orf22	-0,179004746	0,064493768
C16orf68	-0,178938237	0,202603227
DSE	-0,178839094	0,980114844
KIAA1614	-0,178792584	0,677346116
C6orf140	-0,178780282	0,423686821
ANKH	-0,178773565	0,383640272
LOC647137	-0,178716553	0,616369817
FASTKD2	-0,178588819	0,732527298
FBXO2	-0,178495359	0,835026472
UBASH3A	-0,178475596	0,105447337
DBX1	-0,17842128	0,716528905
SSP411	-0,178367569	0,306009105
GPX5	-0,178338355	0,512437697
RAD17	-0,178275786	0,361742942
SMARCA3	-0,178100515	0,745521458
PCDHGB5	-0,178056728	0,143993532
LOC729112	-0,177977099	0,550692722
LOC651787	-0,177970839	0,256313586
ACTA2	-0,177874668	0,611692897
LOC730845	-0,177870384	0,791142605
SALL2	-0,177840236	0,892767088
LOC728176	-0,177832729	0,313046356
KCNK7	-0,177764443	0,947871375
C20orf54	-0,177650428	0,142478887
ANXA6	-0,177507798	0,364328488
C2orf67	-0,177504618	0,336234965
C1orf97	-0,177366228	0,848864689
CYB561D2	-0,177325752	0,244527674
PGM3	-0,177315459	0,274479475
DHDDS	-0,177144041	0,240525471
MADCAM1	-0,177141219	0,436344916
SYTL3	-0,177124172	0,219790921
PSMA2	-0,177039302	0,960391348
ELF2	-0,176919831	0,622259977
CD59	-0,17686677	0,758761438
LHCGR	-0,176665863	0,64984245
LOC728862	-0,176624785	0,290268003
LOC729969	-0,176602713	0,615727772
LOC728232	-0,176522602	0,727000073
HRCT1	-0,176438504	0,148259006
XRCC4	-0,176413104	0,117399562

ZNF34	-0,176360369	0,109993204
ESD	-0,176244863	0,279887783
SAT	-0,17623119	0,510077742
DKFZp761N1114	-0,176197695	0,264831137
LOC728503	-0,175816267	0,264145241
OR51I1	-0,175794518	0,84612985
PM20D1	-0,175756846	0,69095704
C2CD2L	-0,175662183	0,666699851
C7orf49	-0,175559332	0,212530852
AP2A1	-0,175533033	0,403204855
LOC642236	-0,175422665	0,311800555
OR4K2	-0,175398592	0,087081224
DVL3	-0,175087066	0,759903057
KRTHB5	-0,175064406	0,716842821
LBR	-0,175034956	0,524095048
C11orf47	-0,175028237	0,249984202
DAO	-0,174755034	0,763918443
MYO6	-0,174702995	0,162045907
CCL13	-0,174631847	0,762104115
PARD6G	-0,17456829	0,569757719
C15orf37	-0,17441107	0,53826867
MI-ER1	-0,174409615	0,309091112
AGR3	-0,174003023	0,212627679
SULT1A3	-0,173893378	0,517870783
HOXA13	-0,173859238	0,54833965
PRR5	-0,173746336	0,39757559
BTN3A3	-0,17358571	0,517224758
LRAP	-0,173518217	0,05163066
CDH23	-0,173443534	0,804013414
LOC388931	-0,173403165	0,291435628
LOC731139	-0,173373078	0,113706578
OSRF	-0,173316657	0,826752081
LOC729696	-0,173109331	0,972983802
CAPZA3	-0,173055836	0,836377262
FBLP-1	-0,172990003	0,870084097
LOC125242	-0,172982441	0,643453272
TINAG	-0,172943501	0,528432175
SMARCD3	-0,172860054	0,880857351
PLOD	-0,172724477	0,819006505
KLK2	-0,172702653	0,285042
FLRT1	-0,172628263	0,716838655
RNASE4	-0,172549808	0,203633857
MATR3	-0,17242025	0,935301213
ZMYND12	-0,172251285	0,838261417
PTK6	-0,17224811	0,650370097

HIST1H3B	-0,17222388	0,669313254
ACP2	-0,172176778	0,235548621
LOC646625	-0,17215945	0,164087787
LOC647042	-0,172083928	0,743910718
SULT6B1	-0,171811313	0,422427128
LOC729592	-0,171809732	0,793351054
EID1	-0,171791576	0,357384141
MESP1	-0,171726366	0,166106824
LY75	-0,171723463	0,383368115
PRICKLE1	-0,171661758	0,282297028
KIAA0664	-0,171641177	0,602317645
LOC284009	-0,171558259	0,384101113
IMPG2	-0,171452793	0,200045141
GNPTAG	-0,171390874	0,147726247
ZNF499	-0,171335253	0,525845159
SH3D19	-0,171291804	0,7507233
ADAMTS20	-0,171224646	0,022457457
HSPB2	-0,171184589	0,447432261
KRTHA2	-0,170908745	0,304467338
CORO6	-0,170873529	0,920318674
LOC652499	-0,17067396	0,372690033
LOC644096	-0,170627511	0,481546034
OR4X1	-0,170515122	0,381320107
LOC728421	-0,170416586	0,583543075
LOC730272	-0,170406313	0,658735919
LOC729084	-0,170391463	0,325865934
CCDC107	-0,170360378	0,957590928
WDR20	-0,170320661	0,945771775
AADACL3	-0,170267057	0,74710655
LOC643723	-0,170260556	0,355933364
SNX24	-0,170252528	0,416840961
GLIS1	-0,170179104	0,698422593
ZNF638	-0,170155622	0,517087198
PIGP	-0,169869563	0,857069105
NSUN6	-0,169792263	0,68773161
LOC646778	-0,169776208	0,15282933
HOXC5	-0,169765049	0,404030085
COH1	-0,169676891	0,345273215
DDT	-0,169602315	0,545306664
TRIM16L	-0,169477991	0,905286765
ITSN2	-0,169292416	0,35118063
EBP	-0,169219815	0,14362537
ATP5E	-0,169050409	0,346303973
APBA3	-0,168762391	0,542582473
LOC644714	-0,16869145	0,204025305

LOC643373	-0,16869145	0,279025089
VPS37B	-0,168668516	0,261258804
CXCL1	-0,168661197	0,204845208
LOC728662	-0,168581042	0,732896957
VCL	-0,168528782	0,742704
ITIH2	-0,168491496	0,87189246
BOK	-0,168459264	0,188343844
ACOT6	-0,168452184	0,810751191
GPBAR1	-0,168431986	0,825328694
UBE2E3	-0,168310784	0,826682525
GP6	-0,168239889	0,670810146
RAB23	-0,168212836	0,407243704
UNKL	-0,168207562	0,619049629
AHSA2	-0,168158117	0,09846698
HIST1H2AA	-0,168138428	0,975791384
LOC137107	-0,16787348	0,594078443
DVL1	-0,1678359	0,630626781
KIF1C	-0,167828113	0,668164748
MEIS3	-0,167810644	0,291183841
CHCHD9	-0,167774136	0,86919327
LEREPO4	-0,167705065	0,623028218
CAPG	-0,167627019	0,511925927
MNDA	-0,167414157	0,608917233
LRRC4B	-0,167388102	0,248853299
C22orf36	-0,167354713	0,124475259
RECQL4	-0,167230189	0,74168722
LOC375010	-0,167111532	0,286238222
LOC731298	-0,167081662	0,475521753
CD83	-0,166664327	0,862248327
MYBPC2	-0,166645417	0,759683598
RPGRIP1L	-0,166584263	0,307090588
OR5M9	-0,166506391	0,728335862
LOC728580	-0,166431109	0,336550279
C20orf104	-0,166431005	0,191138017
ZNF38	-0,166381614	0,256175927
LSM2	-0,166371818	0,53388635
LOC392447	-0,166315199	0,51073539
PMEPA1	-0,166200793	0,150430098
LOC729977	-0,166196718	0,743137177
GOLGA1	-0,166059671	0,076327425
HOXA2	-0,166019494	0,612084054
ABTB1	-0,165988736	0,208925033
CDC42	-0,165936251	0,209672625
DNAH1	-0,165866252	0,125363431
MAF1	-0,165774319	0,364606868

BCAS3	-0,165723211	0,107135098
LOC729584	-0,16543616	0,616942183
CPXM	-0,165380579	0,890546569
LOC651621	-0,165375195	0,656737833
MRPL32	-0,165355045	0,532746349
SEMA3C	-0,165299749	0,713528312
EGFL6	-0,165164472	0,630977776
CTHRC1	-0,165136676	0,20052494
FICD	-0,164862825	0,545679551
CASP7	-0,164736617	0,167156633
LOC727780	-0,164689895	0,707352328
DOCK2	-0,164596543	0,453423813
LOC730924	-0,164499236	0,176080085
PSTPIP1	-0,164295913	0,748905854
LOC729106	-0,164216169	0,399678131
HRH4	-0,164162953	0,180198854
LOC285205	-0,163969686	0,896661076
UPK3A	-0,163875694	0,597298348
C10orf83	-0,16377843	0,290701689
OR52E2	-0,163726291	0,524295531
SMBP	-0,163670165	0,539921613
CCL16	-0,163636817	0,741793065
TRPC6	-0,163576707	0,910259466
LOC729595	-0,163506503	0,692347738
LOC650887	-0,163480605	0,338337809
FAM7A3	-0,163361538	0,888264277
LOC729904	-0,163177408	0,987166043
LIPK	-0,163068553	0,205261869
BPIL2	-0,162821004	0,893137719
CHRNA1	-0,162707227	0,33179014
hCG_17750	-0,16254338	0,777539527
TLR5	-0,162462269	0,349707348
RDX	-0,162306094	0,257894407
APM-1	-0,162251524	0,733150526
IRAK2	-0,162234363	0,723820459
LOC728587	-0,162121295	0,863695219
ACAD10	-0,162102341	0,691197663
CLCN7	-0,161976601	0,771999609
HOXC8	-0,161890553	0,629907127
CAMK1	-0,16175722	0,539177936
LOC729391	-0,161596896	0,203496346
KIAA1539	-0,161582569	0,653191912
SOX12	-0,161549816	0,770945681
LOC648629	-0,161431386	0,516255389
LOC732228	-0,161130641	0,808308536

FLJ13710	-0,161126892	0,097071524
DENND1B	-0,161106593	0,206155013
RNF19A	-0,161099261	0,425834996
LOC374654	-0,161072773	0,878787732
KIAA0256	-0,161072755	0,951030631
LOC643669	-0,161064248	0,290286313
LOC651158	-0,161054735	0,976017805
MIB1	-0,161050397	0,126862905
ANKRD53	-0,161018542	0,11971679
ARL1	-0,160924677	0,462881384
MDFI	-0,160915563	0,361662447
WDR12	-0,160858031	0,16637597
ZNF273	-0,160833614	0,101030276
LOC730963	-0,160829695	0,838891773
ETV2	-0,160809756	0,94707548
HN1L	-0,160752707	0,538597924
ARID4A	-0,160737999	0,406921
ZNF490	-0,160635072	0,299885175
S100A14	-0,16056474	0,902842439
BBS7	-0,160555607	0,778668138
C2GNT3	-0,160468481	0,850548743
TMEM198	-0,160347698	0,280613087
HOOK2	-0,160250589	0,44933432
C3orf59	-0,160176281	0,184995323
LUC7L	-0,160155072	0,845857879
TXNL4B	-0,159868266	0,66793171
EXOC8	-0,159788485	0,485290367
TRUB2	-0,159559451	0,342194982
LOC731042	-0,159358164	0,713949372
CLECL1	-0,159233321	0,42769163
PSFL	-0,159175384	0,732041217
AKAP9	-0,159148697	0,505161282
CHCHD2	-0,159115754	0,75640628
NPY	-0,158981941	0,141435727
ZNF542	-0,158901291	0,290875384
HRMT1L2	-0,158891262	0,355741665
GPS2	-0,158836352	0,886507462
CAMLG	-0,158616569	0,668610069
WDR75	-0,158569769	0,043581465
LPPR4	-0,158541368	0,358548446
ANKS4B	-0,158538014	0,921316727
C1orf173	-0,158372144	0,147401886
PLEC1	-0,158337489	0,797273812
LOC727819	-0,158284754	0,323805623
PLXNB3	-0,157997297	0,319625687

C13orf35	-0,157972084	0,273906877
MRGX1	-0,157879267	0,358712529
GRAP	-0,15787808	0,647733916
LOC388621	-0,15787256	0,829514704
C6orf58	-0,157856304	0,899489579
LOC645722	-0,157809792	0,27226006
LOC729778	-0,157797391	0,801549094
ADMR	-0,157757527	0,968679529
PI4K2B	-0,157736009	0,697857189
GP5	-0,157706337	0,297258551
OR2T10	-0,157574943	0,556249223
MYL1	-0,157469685	0,364542924
LOC729581	-0,157396394	0,127968118
C6orf122	-0,157207106	0,582632086
OR2T2	-0,157150095	0,414897717
OR4N4	-0,156994821	0,175225169
JIK	-0,156989985	0,765594511
MGC20741	-0,156824412	0,512521867
LOC653151	-0,156767475	0,466563972
MAPK3	-0,156731774	0,806322866
ARID1A	-0,156706361	0,681962603
RNF146	-0,156439431	0,283124098
MPND	-0,156329643	0,641021633
HSPD1	-0,156218077	0,562726922
ZNF804A	-0,156063923	0,434937723
HSPC152	-0,156012641	0,06757424
CLECSF5	-0,156006576	0,967169859
LOC646021	-0,155977068	0,730578605
TSPAN11	-0,155949158	0,200691222
DPP7	-0,155537966	0,235612324
C21orf62	-0,155489273	0,774500911
PRKCG	-0,155488503	0,358172108
C7orf51	-0,155452206	0,906136698
COMT	-0,155246785	0,110571192
GPAM	-0,15508161	0,722934939
CLCNKB	-0,154894656	0,241064278
IFNA5	-0,15483427	0,639774704
C20orf11	-0,15480432	0,279078087
LOC732424	-0,154712412	0,746141637
FGD1	-0,154685309	0,328281478
POLD4	-0,154651147	0,242872256
HOXB2	-0,154616202	0,563938492
CHD1L	-0,154572315	0,479744111
KIAA1856	-0,154564517	0,5
KIF1A	-0,154442701	0,750913775

LEKR1	-0,154401183	0,673639197
IL22RA2	-0,154280955	0,22915069
DENND4C	-0,154245579	0,656642576
LOC644192	-0,154213727	0,361876704
LOC645620	-0,154206728	0,583197529
HOOK3	-0,154055729	0,209044579
LOC728303	-0,153990409	0,193093349
CCDC88C	-0,153874692	0,165966407
KNTC1	-0,153759695	0,950924189
LOC728329	-0,153755899	0,154841701
GOLIM4	-0,153746541	0,281939878
KLHDC3	-0,153737087	0,877596452
COMMD2	-0,153719718	0,854635198
TMEM132D	-0,15364057	0,456423476
SPI1	-0,153628485	0,865819445
DCDC2	-0,153546333	0,302485223
PNO1	-0,153395417	0,746621523
LOC727754	-0,153345043	0,52593063
SLC22A1	-0,153180917	0,80552944
TSPAN7	-0,153069966	0,493560052
CD74	-0,153067201	0,499118674
SDCBP	-0,153040345	0,869375999
FLJ35767	-0,152957966	0,321622576
PPP2R2A	-0,152848433	0,719430569
C9orf122	-0,152847311	0,586218293
LAMP1	-0,152836572	0,216608916
C21orf91	-0,152768413	0,532346806
LOC644297	-0,152632238	0,319302662
RNASEH2A	-0,152595559	0,190203281
SELP	-0,152585051	0,570840345
APOH	-0,152555502	0,364233941
GTSCR1	-0,152462076	0,140240188
MAP6	-0,152372062	0,991244706
HACE1	-0,152363609	0,244025816
ALOX12	-0,152144405	0,528025126
C2orf18	-0,152060025	0,611089649
NLK	-0,151939381	0,915780692
FTSJ1	-0,151885846	0,147794627
UFSP1	-0,151834044	0,93364756
IMPDH1	-0,151786087	0,114270963
RILPL2	-0,151763078	0,461123626
LOC732434	-0,151713647	0,623890066
LOC728544	-0,151597961	0,271342705
RAB18	-0,151589021	0,783713213
SBSN	-0,151529809	0,167041133

PNKD	-0,151195492	0,286199867
TXNRD1	-0,151110124	0,590703928
GRIP1	-0,151101053	0,25539173
DEDD	-0,15089745	0,613095407
INSR	-0,150894142	0,418078917
Hes4	-0,150736485	0,641446972
ANKRD34A	-0,150663154	0,298666914
LOC644569	-0,15053535	0,773526842
KRTAP4-12	-0,150512074	0,153762202
CDKN2A	-0,150353101	0,23879763
ING5	-0,150294447	0,490604749
RANBP2	-0,150292748	0,936770084
LOC388969	-0,15020689	0,916378051
FMN2	-0,15017171	0,560601801
BTN2A3	-0,150154805	0,489286471
UPB1	-0,150141419	0,952453435
IGF2R	-0,150014757	0,979625981
OR52E6	-0,150007373	0,810276463
PCDHGB7	-0,149937763	0,746935884
THBS1	-0,149896621	0,449628772
COX7C	-0,14989076	0,835723797
ZBTB38	-0,149827922	0,514726616
HIST1H1D	-0,149810141	0,932633385
PRKRA	-0,149658481	0,891035291
ZNF265	-0,14965773	0,652627013
WBSCR1	-0,1496419	0,501413922
ANKRD57	-0,149411029	0,373538988
PIGC	-0,149382745	0,220159118
LOC646077	-0,149378664	0,398867115
GCM2	-0,149371028	0,896380201
LOC652721	-0,149359906	0,615666698
RNF14	-0,149322585	0,760515792
LOC732427	-0,149251114	0,28659694
OCRL	-0,14908864	0,909865636
3pHEXO	-0,149035697	0,670433411
PDLIM1	-0,148975053	0,208561768
LOC649839	-0,148956878	0,60388891
FLJ25006	-0,148928356	0,321039241
SFTPD	-0,14890669	0,106462984
LOC255275	-0,148872301	0,083174692
SPATA13	-0,148853068	0,449437212
PDE9A	-0,14884125	0,226981955
POLR2D	-0,148729002	0,776900809
CETP	-0,148697425	0,940622662
HS3ST3B1	-0,148678634	0,638005919

HAPLN2	-0,148638209	0,905909812
LOC653978	-0,14862884	0,307355713
ZKSCAN1	-0,148544057	0,964191208
LOC727854	-0,148462445	0,362185389
LOC729714	-0,148446476	0,761670161
ACTR10	-0,148438544	0,505263014
FLJ31121	-0,148293579	0,906864719
LOC645273	-0,148228637	0,373695917
TPD52	-0,147897182	0,346305736
FAM48B1	-0,147800402	0,253470566
PRICKLE4	-0,147767175	0,990267231
SNCB	-0,147685584	0,287870149
TMEM200A	-0,147635527	0,789959676
TEGT	-0,147592791	0,483190151
NCOR1	-0,147592192	0,128486879
ADORA3	-0,147571558	0,574293343
UNC50	-0,14748534	0,495091398
IRX3	-0,147449834	0,222780484
CANX	-0,147321201	0,928627854
FAM120B	-0,147213269	0,594019893
NTN1	-0,14718798	0,687779383
HAL	-0,147161826	0,139975505
ZNF235	-0,147067282	0,996145094
GNAT2	-0,146868726	0,200644523
GPR146	-0,146824764	0,29492902
ASCL1	-0,146791412	0,859621502
DUSP7	-0,146782611	0,859766698
ENAM	-0,146715952	0,715485619
C21orf119	-0,146686574	0,397399531
TXNL1	-0,146630128	0,721046879
POLR2H	-0,146609224	0,516331729
STK17A	-0,14654299	0,285875398
STX3A	-0,146367724	0,690735269
SUPT5H	-0,146215629	0,354426561
LOC83693	-0,14608313	0,701531185
PLEKHG2	-0,145859467	0,342224818
KRTAP5-7	-0,145829214	0,649596146
NSUN7	-0,145720891	0,091750122
LOC401497	-0,145388863	0,333936685
VSTM2L	-0,145303971	0,681054944
CD99L2	-0,1452998	0,509896722
MACROD1	-0,145231415	0,113174325
A4GALT	-0,144971506	0,249834268
LOC652526	-0,144928364	0,051552129
HK3	-0,144927874	0,526130445

STK24	-0,144925983	0,343755254
CHRNB4	-0,144897081	0,565316339
SPANXN2	-0,144808439	0,110808829
CCDC155	-0,144768807	0,13865509
BAI2	-0,144759395	0,529320257
STX4A	-0,144749487	0,510553111
LOC650122	-0,144647493	0,488877441
FLJ20793	-0,144629702	0,505797699
KCNMB2	-0,144612472	0,580056463
C1orf102	-0,14461056	0,821884623
PKD1L1	-0,144587009	0,342543477
GZMA	-0,1445721	0,2356178
DBT	-0,144523032	0,221889196
NINJ2	-0,144239338	0,813390508
PIGU	-0,144192249	0,211934954
CACNA2D1	-0,144009236	0,458982111
SLC25A46	-0,143912735	0,107407645
C6orf146	-0,143904463	0,607988191
FATE1	-0,143894158	0,605990137
CD68	-0,143891369	0,766221275
LOC646300	-0,143890082	0,815690047
COBL	-0,143845674	0,374482511
TUFM	-0,143836435	0,325281836
NXPH2	-0,143663685	0,565003453
ZIC1	-0,143565363	0,474366991
C20orf165	-0,143552292	0,22452387
RHOJ	-0,143550495	0,847746268
MAPRE2	-0,143488531	0,203303826
SSNA1	-0,143460276	0,838495176
AQP2	-0,143344626	0,569045599
FAM156A	-0,143340969	0,969383805
C1orf71	-0,14318726	0,549185516
PLAC8	-0,143099396	0,148293541
DNCLI2	-0,142985517	0,581034213
POU2AF1	-0,142837888	0,206083999
PR48	-0,142760294	0,956818521
TP53TG3	-0,142754598	0,919375827
GPR155	-0,142732518	0,890707971
CES3	-0,142570328	0,742551689
ESRRBL1	-0,142478064	0,525461384
TNFSF18	-0,142334569	0,340035147
FLJ46481	-0,142308773	0,110865718
SH3TC2	-0,142272389	0,644129116
SPATA1	-0,142233773	0,443375905
LOC643641	-0,142176604	0,186969335

TJP1	-0,142166914	0,856542557
C10orf6	-0,142153295	0,509685568
ZFP36	-0,142025029	0,527623474
FKBP4	-0,141962456	0,538729163
PLEKHG4B	-0,141918795	0,531554432
LOC201229	-0,141889959	0,82915561
C12orf54	-0,141668257	0,555559524
SUNC1	-0,141618509	0,743991748
ADAT1	-0,14151444	0,773987534
DNLZ	-0,141495357	0,219614762
PHF16	-0,141495084	0,615247489
CCR2	-0,141409309	0,774063074
TICAM1	-0,141404828	0,206884385
C17orf37	-0,14136732	0,367091362
LOC729306	-0,141266825	0,12534056
TAF1B	-0,141155487	0,723859136
OMG	-0,141108901	0,174162178
PDE4C	-0,141030583	0,970650248
KCNE2	-0,140984478	0,801263669
PIN4	-0,140956942	0,538476117
OR51G1	-0,140914932	0,673505763
OR6X1	-0,140895385	0,128555773
MAB21L1	-0,1408726	0,969572279
HDAC1	-0,140822224	0,579870928
PSAP	-0,140803907	0,472793951
HDAC10	-0,140536214	0,691508613
CHD6	-0,140152715	0,314926787
LOC643233	-0,140102568	0,549731405
TGFBI	-0,140028214	0,771447573
LOC649009	-0,140000827	0,434019164
MMACHC	-0,13997815	0,227933493
ZBTB1	-0,139963566	0,647499946
XKR4	-0,1399357	0,476763568
LOC391771	-0,139915244	0,653493344
RNF30	-0,139819055	0,967175697
MGC42493	-0,139815432	0,840232492
AHNAK2	-0,139800387	0,573029545
UNQ6125	-0,139790723	0,437311259
CRH	-0,139787732	0,291741941
SETD4	-0,139752883	0,990731612
NDUFB8	-0,139693492	0,814919282
LOC731410	-0,139675316	0,096307618
FAM109B	-0,139655249	0,145933893
VGLL1	-0,139594151	0,099252693
CD97	-0,139589306	0,45541273

CIB2	-0,139442234	0,109811012
TRAFD1	-0,139417714	0,423139452
FMNL1	-0,139359643	0,933793272
C9orf144	-0,139289112	0,650610137
EID2B	-0,139236983	0,763709145
LOC441876	-0,139194377	0,5
UACA	-0,139024318	0,895026588
LOC643554	-0,139018366	0,826887581
BCL2L14	-0,138984314	0,631487581
WIPI2	-0,138919186	0,776998892
INSIG1	-0,138806643	0,585395748
SYT4	-0,138790443	0,801539575
ALDH7A1	-0,138561515	0,234666974
RPAIN	-0,13855124	0,192843691
LOC339809	-0,13845827	0,497539179
ODF3L1	-0,13840524	0,747985508
LOC731014	-0,138326749	0,393055394
IL18RAP	-0,138266262	0,675300682
CES2	-0,138263522	0,415284473
LOC646853	-0,138208052	0,771602652
LXN	-0,138197874	0,41825774
CTSL2	-0,138194245	0,877525231
HIBADH	-0,138153507	0,951482877
LIM	-0,138131683	0,282338302
NUMA1	-0,137895139	0,96006187
LOC550631	-0,137886873	0,111667448
ALLC	-0,137796519	0,211889141
LOC642998	-0,137774769	0,381798598
FKBP11	-0,137729221	0,929534326
LOC729280	-0,137697182	0,685995507
ATP10A	-0,137615536	0,08205442
ADCY3	-0,137537861	0,571965858
SLFNL1	-0,137415212	0,954070312
PGLYRP4	-0,137412845	0,885565003
CALCOCO1	-0,137380402	0,26419717
PAQR3	-0,137303763	0,283184486
TMEM40	-0,137288574	0,225668205
LOC643133	-0,137266572	0,179088085
LOC100132288	-0,137183988	0,876819665
CEP250	-0,136955577	0,814151294
LOC650909	-0,136906668	0,801737737
GPR123	-0,136853933	0,496513593
CDR2L	-0,136677991	0,397897932
ADSSL1	-0,136659198	0,868137254
LOC389906	-0,136599423	0,229870353

hCG_38941	-0,136581151	0,194529513
KRTAP9-2	-0,136551721	0,126761995
NCAPD2	-0,136467876	0,260279279
HAP1	-0,136389792	0,410871765
HBXIP	-0,13634285	0,316566516
PTDSS2	-0,13620631	0,355478494
RABL3	-0,136187388	0,724841105
FRMPD4	-0,136048882	0,690227215
LOC730437	-0,135959143	0,815695958
LOC652608	-0,135955878	0,695160151
LOC729436	-0,135856818	0,169388109
C3orf70	-0,135852517	0,310409513
GPC4	-0,135787361	0,827282787
F2	-0,135776038	0,83265669
RFNG	-0,135752772	0,362630866
PLEKHH3	-0,135560122	0,233401509
CXCL2	-0,13554964	0,366627221
C12orf32	-0,13544438	0,3043067
C13orf27	-0,135357668	0,382821303
MEF2C	-0,135299696	0,383418729
WISP1	-0,135235397	0,619202004
TET2	-0,13512051	0,314154324
RPL35	-0,1350188	NA
C6orf125	-0,134941004	0,235650728
TRAF3	-0,134864848	0,507111975
NLRP10	-0,13485233	0,177332262
SLC22A7	-0,134851317	0,2511295
CAP2	-0,134778785	0,231026228
ADAMDEC1	-0,134713404	0,903110119
LOC400986	-0,134634469	0,263928621
CENTA1	-0,134561978	0,595157675
OR4A5	-0,134499011	0,584768239
GRM4	-0,134447905	0,274373083
TLX2	-0,134404629	0,814045842
OR1N2	-0,134379826	0,979421583
LOC644056	-0,134351351	0,068264109
HLA-DPA1	-0,134289324	0,784123839
LOC92979	-0,134239331	0,724869379
BNIP1	-0,134234278	0,132013254
SCARB1	-0,134216132	0,947105631
TNFRSF14	-0,134189978	0,207007345
GAL	-0,134168003	0,517539799
LOC647188	-0,134157606	0,373130919
SLC9A4	-0,134138647	0,45591911
HS3ST4	-0,134051161	0,174257125

CDADC1	-0,133972686	0,244557696
OR1M1	-0,133903537	0,346157309
NT5C	-0,133806884	0,546005912
CYSLTR2	-0,133774405	0,918121304
KRT25	-0,133726087	0,997145218
LOC402232	-0,133633461	0,240613617
PRF1	-0,133574328	0,270533628
TAS2R5	-0,133534452	0,282756911
EFCAB2	-0,133521418	0,208732468
CRYL1	-0,133505956	0,281158261
SOX13	-0,133423134	0,467187799
HKR3	-0,133410692	0,943546246
CEACAM5	-0,133391285	0,823362894
SLC2A10	-0,133309947	0,456579398
LOC729426	-0,133215686	0,322484955
TAC1	-0,1332144	0,410965372
SLAMF1	-0,133050443	0,251433832
CSPG6	-0,132920541	0,771172492
HSPC051	-0,132797984	0,872435227
SHMT2	-0,132531568	0,39730246
EFCAB3	-0,132509413	0,830954924
TUBA1	-0,132416703	0,652502413
LOC641859	-0,132398233	0,624305176
FAM64A	-0,132383393	0,415670325
LOC729879	-0,132374386	0,254764209
ZNF750	-0,132304517	0,760922384
PAK6	-0,132290192	0,688811719
FCRH3	-0,132271737	0,93040682
LOC729117	-0,132267043	0,68536963
LOC729405	-0,132252749	0,565724119
FREQ	-0,132238779	0,665493742
WFDC12	-0,132159679	0,129187906
MARK1	-0,132028378	0,757796328
RPL18A	-0,131961099	0,482440591
CA8	-0,131912452	0,500090858
FBXO40	-0,131864507	0,248165514
AZI1	-0,131854446	0,884296404
MGST3	-0,131805619	0,4306604
LOC643072	-0,131776746	0,510568943
GAPDS	-0,131633242	0,387867363
GDF6	-0,131487826	0,665574272
CASP4	-0,131370058	0,317719232
TUBA6	-0,131310444	0,299233584
GPR145	-0,131082217	0,323521653
ENHO	-0,13106883	0,270188988

C2orf30	-0,13104431	0,265432709
PGBD1	-0,130844043	0,870305032
SPRR2A	-0,130834529	0,86808296
LOC652697	-0,13081373	0,418306156
AWP1	-0,130689198	0,955853092
LOC127545	-0,130688537	0,787804072
FLJ20399	-0,130577951	0,355788336
ATP11C	-0,130519814	0,484169376
C1orf130	-0,130382073	0,336208418
HNRPA2B1	-0,130318936	0,372135661
EHHADH	-0,130228072	0,360735107
LOC729562	-0,130081979	0,534319814
LOC647313	-0,129878984	0,08210718
IFT172	-0,129870262	0,675316816
RAPGEFL1	-0,129811192	0,393216116
PHKB	-0,129763063	0,666304392
SLC45A4	-0,129728683	0,921180489
BRD8	-0,129661666	0,355726837
KRTAP8-1	-0,129652931	0,77455742
LOC441344	-0,129642333	0,173875762
CENPT	-0,129590144	0,262553864
TRERF1	-0,129525606	0,528254076
POTEA	-0,129404054	0,649132522
OR11H4	-0,129394625	0,392808737
LOC729414	-0,129386554	0,347699406
CCDC15	-0,129384016	0,313818024
NLRP3	-0,129376129	0,61147768
CLSPN	-0,12937582	0,509407635
ROPN1L	-0,12934185	0,3742867
TMC1	-0,129335199	0,928031759
ANKRD45	-0,129222302	0,826998764
EPHX1	-0,129143242	0,605162696
GPR44	-0,129065414	0,810587035
SLC5A1	-0,129055889	0,60915575
HDC	-0,128938675	0,406143378
LOC643563	-0,128895999	0,974541612
KCNK15	-0,128823963	0,587559266
ABTB2	-0,128815922	0,98871283
LOC728336	-0,128812774	0,25364903
CEP78	-0,12878247	0,003642
GSG2	-0,128754982	0,672958013
LOC731384	-0,128610199	0,906490984
FLG2	-0,128453018	0,007162338
FAM20C	-0,128382002	0,317655736
LRRC46	-0,128354511	0,742570581

LOC145837	-0,128310999	0,21496271
UBAP1	-0,128269435	0,832978093
CXXC5	-0,12821603	0,962321336
EDARADD	-0,128079313	0,974977812
IMPACT	-0,128063128	0,149635233
SSB3	-0,128006074	0,404789038
PCOLCE	-0,127973903	0,12564562
LAMA3	-0,127902674	0,361507743
SEC16A	-0,127892269	0,427731158
SELV	-0,127777146	0,16670683
FLJ45994	-0,12765565	0,537626206
BANP	-0,12765523	0,542868416
CEACAM6	-0,127619418	0,313896171
LOC729652	-0,127619332	0,629203364
MMP19	-0,127615819	0,142086545
RPL14	-0,127576238	0,677230787
LOC391524	-0,12749346	0,933765454
FGFBP3	-0,127472571	0,546533462
CACNG3	-0,127455012	0,281699388
LOC653698	-0,127383358	0,152225114
WDR8	-0,127364278	0,593426821
LOC728142	-0,127261871	0,889062277
LOC727785	-0,127253748	0,392275501
MCOLN3	-0,127220817	0,231882815
ENG	-0,127155678	0,871885255
OFD1	-0,127036778	0,472779847
SSH2	-0,12702897	0,450276136
C1QTNF1	-0,126806102	0,144208362
SNRPEL1	-0,126622418	0,584316935
DSTN	-0,126618264	0,323724654
MSTO1	-0,126615335	0,690458495
LOC728542	-0,126542066	0,575211494
VAV1	-0,126446198	0,453363705
PDE4D	-0,126353462	0,64565041
LOC390110	-0,126351051	0,345850811
LOC728655	-0,126291603	0,757428559
PDZD4	-0,126252877	0,366809584
MRTO4	-0,126235358	0,277424076
CDC42BPA	-0,126191221	0,430409343
LOC144817	-0,12604714	0,741134029
EHD4	-0,126019233	0,637545904
GRIPAP1	-0,125968678	0,186595086
LOC391370	-0,125945465	0,458172269
MAPKAPK3	-0,125885514	0,600295269
SPATC1	-0,125822387	0,219021451

CDC123	-0,125779009	0,125898572
GALNTL5	-0,125778101	0,742741091
KIAA0329	-0,125748148	0,491719152
PRKAA2	-0,125715847	0,772144552
C7orf47	-0,125688684	0,354044639
FXYD3	-0,125620711	0,215134897
NPHS2	-0,125585171	0,873138419
BTLA	-0,125430338	0,357456575
PPAPDC3	-0,125422748	0,773385782
ANXA4	-0,125324167	0,332112285
RPL23AP2	-0,1252413	0,705552286
FGF1	-0,125235039	0,353920783
HS3ST1	-0,125131245	0,218487714
LOC729043	-0,125073011	0,359432087
VPS25	-0,125069206	0,940092359
ADCY2	-0,125056361	0,374975273
MIP	-0,124923997	0,352731173
C6orf94	-0,124881905	0,575161555
TSGA14	-0,124790136	0,083188813
HRG	-0,124644578	0,765870364
BRAF	-0,124611484	0,858836414
UTP18	-0,124606661	0,350599255
LOC728325	-0,124596475	0,503100281
LOC731724	-0,124540697	0,098397791
LOC732472	-0,124496365	0,29459018
ZNF365	-0,124411688	0,539159881
MBL2	-0,124367118	0,710207491
TFDP3	-0,124365391	0,564399171
NLRP14	-0,124200344	0,224128537
XAF1	-0,124131881	0,292105224
PRDM8	-0,124105801	0,052464342
CENPM	-0,12365548	0,85860621
LOC388459	-0,123554484	0,172466898
LOC644838	-0,123541167	0,239964763
HCN1	-0,123522208	0,898259299
PON3	-0,123376028	0,928939519
VWF	-0,123312191	0,425514679
NAF1	-0,123288793	0,85763295
RARG	-0,12325163	0,357272315
LOC729054	-0,123124065	0,41919977
CHRNG	-0,122973295	0,564840972
ATP8A2	-0,122954216	0,805155411
SAMSN1	-0,122847362	0,552724478
C19orf28	-0,122725866	0,429609753
TBK1	-0,122714194	0,663061914

TRPC4	-0,122688206	0,609622387
DMRTC1	-0,122546235	0,996052391
BAIAP1	-0,122484367	0,535459894
LOC642633	-0,122401089	0,979767347
FANCC	-0,122361101	0,421471118
LOC644590	-0,122335823	0,444056294
IRS4	-0,122224407	0,742484864
POLE4	-0,122206054	0,188821581
AKAP4	-0,12220018	0,95855408
FLJ40504	-0,122163386	0,084159572
LOC653852	-0,122103432	0,402482726
LCE2B	-0,122090328	0,514421513
CACNA2D3	-0,122078875	0,192846408
LOC645227	-0,122035264	0,558047216
LOC731501	-0,121895848	0,900907053
RAB7B	-0,121838808	0,614276263
FHOD3	-0,121800102	0,407058141
CCND3	-0,12166284	0,209723038
SLC6A17	-0,121595316	0,772804269
UXT	-0,121541398	0,392922903
LOC729623	-0,121486567	0,835313523
TTL	-0,121460311	0,324540445
CARD11	-0,121432133	0,684929078
PRNP	-0,121421505	0,567382639
SH3MD4	-0,121354477	0,728353168
RCP9	-0,121089672	0,307425696
KIF3C	-0,120981068	0,861584601
TTMA	-0,120682911	0,928797531
LOC729709	-0,120650312	0,909757141
SPTAN1	-0,120559868	0,29651893
TLE6	-0,120547608	0,978504493
MOCOS	-0,120470815	0,193991967
SLC35E4	-0,120279247	0,735840487
C16orf90	-0,120181636	0,843794255
MOBKL1A	-0,12014757	0,771684007
SLC39A9	-0,120012844	0,618361008
LOC343851	-0,119992432	0,792440317
LOC729678	-0,11996216	0,471918318
PZP	-0,11992084	0,516916454
DNAJC14	-0,119898418	0,708154192
GDA	-0,119892631	0,957518526
LILRB1	-0,119757794	0,658116501
HLA-F	-0,119705716	0,760283999
LOC731389	-0,11966472	0,19943952
SLC19A1	-0,119662396	0,83894311

LOC402279	-0,119597929	0,348330168
OR6C6	-0,119581198	0,142010355
TMEM166	-0,119529235	0,564354576
SV2C	-0,119527107	0,749396163
RASIP1	-0,119478189	0,942461065
PQLC2	-0,119443247	0,149898909
ARNTL	-0,119424641	0,7125207
LOC731780	-0,119307602	0,249521678
PBEF1	-0,119250719	0,627226064
LOC729429	-0,119214311	0,636433908
SERP2	-0,119196243	0,245227102
LOC730184	-0,119049404	0,343880377
PGM1	-0,118996	0,720282685
ABCF2	-0,118939299	0,049934048
LOC730221	-0,118859176	0,922363346
LETM1	-0,118857613	0,365964159
LOC728068	-0,118755387	0,417940091
WBP2	-0,118667895	0,289228195
BDH	-0,118658095	0,213016984
TMEM57	-0,118481083	0,694927979
RALY	-0,118312042	0,94667416
LOC285908	-0,118175069	0,550509262
OR6C70	-0,118170185	0,367287226
ABCA12	-0,118078966	0,344370348
SNRPE	-0,118074586	0,901933298
FGF20	-0,11800488	0,386020978
CCDC153	-0,117929527	0,580147782
hCG_18635	-0,11781352	0,286458871
CALCRL	-0,117734493	0,705641822
PSG11	-0,117675289	0,220449017
MRPS31	-0,117464697	0,554786941
CRISPLD2	-0,117421661	0,648504678
KCTD14	-0,11736134	0,513139035
INTS10	-0,117357524	0,953437261
LOC402665	-0,11723783	0,515771088
ASTN2	-0,117173144	0,983146693
SDFR1	-0,117090913	0,870292506
SERPINA10	-0,116996007	0,815798497
GALR3	-0,116865517	0,175391787
IL23R	-0,116857191	0,246870742
SPESP1	-0,116807831	0,946486574
LOC730961	-0,11679108	0,276922831
UCA1	-0,116770636	0,170414258
CCAR1	-0,116751627	0,943732884
LOC402375	-0,116647801	0,767201176

ALG1	-0,116539497	0,488949496
SCRT1	-0,116507713	0,219952514
LOC642307	-0,116494482	0,601814944
C11orf55	-0,116264588	0,983275311
KIF21A	-0,116195606	0,955360096
SLC10A7	-0,116077161	0,215218245
HISPPD2A	-0,115938642	0,691637793
TTLL8	-0,115722379	0,76604247
C3orf75	-0,115593607	0,507386383
EIF5	-0,115453301	0,609612834
NFKBIL2	-0,115384657	0,038251498
LMOD1	-0,115383348	0,958622203
CYP2B6	-0,11532405	0,509164659
C6orf108	-0,115177955	0,160541954
LCE3D	-0,115122711	0,177084378
LOC728231	-0,114981771	0,554803615
CHD2	-0,114942992	0,277018055
HAS1	-0,114879652	0,213132345
DDX24	-0,114857392	0,752095038
STK29	-0,114828034	0,364635891
GLI2	-0,114725162	0,821239078
MBD6	-0,11468484	0,256416186
CDC2L2	-0,114680543	0,338194167
PIGA	-0,114636893	0,833861839
MGC26484	-0,114499046	0,635267371
C9orf114	-0,114483374	0,597727425
OR5B2	-0,114467072	0,269598609
CACNA1B	-0,114087404	0,415262181
OR2A4	-0,114019161	0,977153016
FBXO16	-0,113927718	0,590280041
FER	-0,113783182	0,587324336
LOC728569	-0,113757864	0,985057127
DKFZP434L1717	-0,113622586	0,944772996
INSC	-0,113493531	0,150596393
SLC1A4	-0,113483181	0,728403307
C6orf221	-0,113413196	0,172454789
XAGE1D	-0,113356441	0,279016141
BST2	-0,113320114	0,303798206
RPIA	-0,113290528	0,945660792
TARS	-0,113007126	0,062269067
LOC126661	-0,112903199	0,396243742
LOC649917	-0,112762885	0,765141033
TDRD5	-0,112499402	0,649886073
PROP1	-0,112355759	0,539412566
SIM2	-0,11227118	0,550843552

NODAL	-0,112194832	0,921686524
LAPTM5	-0,112133476	0,421509967
STARD10	-0,112109686	0,417287522
POLR2I	-0,11171742	0,426217421
LOC649676	-0,111685262	0,147529522
MGEA5	-0,111530792	0,672813705
TBXA2R	-0,11144154	0,605157165
CBLB	-0,111408348	0,563198446
IKIP	-0,111361412	0,749984811
BSN	-0,111329632	0,674697435
PRR7	-0,111269472	0,457158982
TMEM26	-0,11122875	0,429271909
LOC732443	-0,111177891	0,591815046
SLC22A6	-0,111153375	0,247453166
AHCYL1	-0,111119706	0,193508058
STON1	-0,111036699	0,237138408
TTC3	-0,110926885	0,938076675
UBE2J1	-0,110893207	0,565581353
GPSN2	-0,110879493	0,982472467
TIMP1	-0,110727422	0,56134701
EGLN2	-0,110531329	0,700554778
KIF13B	-0,110363647	0,85488036
IFFO1	-0,110316756	0,255321011
ZNF21	-0,110144552	0,65584416
WT1	-0,11009993	0,95750997
ZNF275	-0,109931656	0,652459906
SCRN3	-0,109877315	0,597257439
FAM59B	-0,109759068	0,287833176
LOC400796	-0,109756509	0,431972267
LOC728967	-0,109755934	0,855065085
HRH1	-0,10964307	0,487438482
APPBP1	-0,109615184	0,831085592
LOC729756	-0,109584993	0,196802786
LOC727939	-0,109328491	0,961908592
TNFSF9	-0,109322956	0,612311963
NETO1	-0,109307726	0,979868457
LOC729345	-0,109274278	0,180972028
LOC647654	-0,109262256	0,449835876
LOC727961	-0,109168453	0,9845286
LOC730228	-0,109067798	0,991405426
LOC731066	-0,108997394	0,670625554
SET7	-0,10895985	0,234687741
FAM100A	-0,108878761	0,207771001
PTHR1	-0,108862118	0,654407178
LOC652607	-0,108753391	0,527694577

LOC731511	-0,108733353	0,718995601
FXN	-0,10866545	0,350936972
NEK11	-0,108484346	0,252360815
LOC727908	-0,10847835	0,19649866
KIAA0391	-0,108198595	0,850936347
GLULD1	-0,108168099	0,644482098
KCNN1	-0,108160112	0,996586117
C14orf145	-0,10807699	0,658071214
ZNF223	-0,108062507	0,518614864
IRF1	-0,107960869	0,729246904
CTBS	-0,107942982	0,540673291
LOC727948	-0,10785884	0,644514625
LOC283584	-0,107793247	0,131762881
NMD3	-0,107776765	0,824185139
LOC730068	-0,107764542	0,231907012
PPFIA4	-0,107704441	0,455827998
ELOVL5	-0,107640088	0,812188915
HOXB5	-0,107626808	0,874030407
IKBKAP	-0,107587668	0,219966114
ETS2	-0,107521723	0,504868171
LAMC2	-0,107371337	0,276369443
NKG7	-0,107311224	0,792363683
C16orf70	-0,107254599	0,369635729
DSCR3	-0,107202867	0,249541981
KRTHB3	-0,107093706	0,758213544
MAMDC4	-0,107058555	0,650757414
SERPINE2	-0,107023771	0,686251092
PGM5	-0,106816688	0,316149142
custom	-0,106762959	0,829047274
C9orf98	-0,1065321	0,726359367
PPIL4	-0,106520479	0,834493514
MAP3K7IP2	-0,106352882	0,511249939
DOK1	-0,106326166	0,754083672
EPB41L3	-0,106279366	0,307288797
MAP3K4	-0,10614561	0,646246546
LOC401577	-0,106067547	0,935870218
PRG4	-0,106064196	0,352496934
LOC729304	-0,106014578	0,908102333
TRMT1	-0,105829868	0,819854013
BGR	-0,10581484	0,400337765
INADL	-0,10579463	0,659200251
ERBB2IP	-0,105751451	0,184116561
GRINA	-0,105721024	0,142457943
MGC26718	-0,105677074	0,753757214
CSNK1G3	-0,105671302	0,237681137

AMELY	-0,105604314	0,563903666
MSL3L2	-0,105581637	0,109463831
ZNF562	-0,105452601	0,762071827
CSMD3	-0,105331253	0,252183331
ATP1B3	-0,10531747	0,72250219
LOC729745	-0,105233431	0,0952634
BAIAP3	-0,105167626	0,782499084
SGCB	-0,105016692	0,604315166
FAM18B	-0,105010945	0,483318428
CEACAM20	-0,105002907	0,944951467
MFSD11	-0,104975726	0,469918173
CPNE1	-0,104970053	0,829351238
FLJ32154	-0,104961829	0,494525001
SNX7	-0,104715754	0,449354992
ADRA2B	-0,104666326	0,324228427
OSBPL9	-0,104601838	0,768675119
SSFA2	-0,104526579	0,255253499
POU4F1	-0,104489002	0,71217389
PTPRF	-0,104420009	0,731495978
LOC204474	-0,10441574	0,286211468
SERPINA9	-0,104410309	0,358255874
HICE1	-0,104317236	0,549159963
PRKAG2	-0,104299177	0,656855936
FADD	-0,104273725	0,839936698
LOC728848	-0,104238603	0,247597164
OR11H12	-0,104215131	0,866049629
LMLN	-0,104124364	0,312315442
01, Sep	-0,104062336	0,337420531
PXK	-0,103989835	0,760356054
TIMM10	-0,103987103	0,661089927
LOC649755	-0,103954158	0,166126456
FLJ23506	-0,103938631	0,506186101
ZCCHC5	-0,103891118	0,791595009
ATP5L	-0,10388969	0,633644068
KIAA0141	-0,103610882	0,646312636
FNDC3B	-0,103370575	0,764047976
ZIC4	-0,103235586	0,447335743
OR4C46	-0,10317797	0,939002548
LOC642648	-0,103149007	0,409620952
LOC728886	-0,103131505	0,541400135
PRKD2	-0,103091259	0,826316833
C3orf54	-0,103031864	0,341525172
RBM47	-0,103009107	0,474452815
LOC728775	-0,102917979	0,394242636
LOC728455	-0,102870326	0,8277205

LOC651771	-0,10283022	0,577226822
TIMP3	-0,102808406	0,159096143
C16orf28	-0,102793234	0,344618752
ARHGAP15	-0,102784731	0,386718637
ATP11B	-0,102719923	0,242469142
EBNA1BP2	-0,102643594	0,182872164
RBM35B	-0,102625678	0,425132363
STK25	-0,102598732	0,842829785
LANCL1	-0,102544824	0,326129355
LRRC61	-0,102534711	0,277028334
NPS	-0,1024921	0,240568337
GATA2	-0,102417401	0,799952344
LOC645686	-0,102375356	0,441439344
MOSPD2	-0,102282113	0,229034811
ARL13A	-0,102174342	0,402017489
LOC728377	-0,102152118	0,251930585
RHBG	-0,102141238	0,403174265
C19orf16	-0,102074195	0,25067071
LRRC37B	-0,101979575	0,888610334
LOC646915	-0,101961847	0,700308828
NMT1	-0,101885333	0,416347234
CRABP2	-0,101737421	0,240408784
HAPLN3	-0,10167395	0,966336624
DARS	-0,101668846	0,352763929
IFNGR1	-0,101651113	0,819119294
CNTD2	-0,101557655	0,711475192
PLEKHA4	-0,101536689	0,981619377
PDCD8	-0,101453117	0,91248278
LOC730429	-0,101420055	0,889714692
FLJ25059	-0,101362049	0,150476739
IFNK	-0,101311253	0,175896959
DDX1	-0,101301903	0,811120078
DKFZp762F0713	-0,101197051	0,737893388
CXorf38	-0,101145732	0,813832126
LOC731128	-0,101093283	0,376429004
ARMC6	-0,100962042	0,574966918
C20orf82	-0,100928099	0,14119149
ATP1B1	-0,100856177	0,756882546
CYP46A1	-0,100816338	0,981480772
LBX1	-0,100797267	0,440654824
RNF207	-0,100781814	0,856665479
PDK1	-0,100750434	0,532127642
GNA12	-0,100704732	0,236748832
CCDC102B	-0,10050561	0,828674571
MPHOSPH10	-0,100505399	0,867859984

HAAO	-0,100411197	0,37793232
DENND2D	-0,100372686	0,629577577
MUCL1	-0,100352649	0,323673826
LOC220032	-0,10033763	0,445811122
GLYCTK	-0,100297195	0,608281987
MYST3	-0,100260579	0,45683084
POLR1E	-0,10024881	0,568708269
LOC730240	-0,100206553	0,157370557
CRP	-0,100148247	0,699362853
LENG4	-0,100052805	0,698387683
CCDC34	-0,099995935	0,445516516
ZNF383	-0,099977299	0,634096807
RIMS2	-0,099970983	0,411605489
C20orf20	-0,099908389	0,723289508
SPATA19	-0,099840857	0,97506443
C19orf12	-0,099823445	0,241712425
LOC730015	-0,099815266	0,817814463
SREBF1	-0,099802989	0,400102806
GPR63	-0,099772363	0,299772035
ALKBH3	-0,099643673	0,621429131
MBTPS1	-0,099643618	0,589639129
LOC652265	-0,099626572	0,790896355
C4orf32	-0,099526572	0,987705215
CARKL	-0,099413102	0,280816609
C16orf72	-0,099360231	0,230007154
BUB3	-0,099316874	0,106751513
UAP1	-0,099273309	0,151653605
TREML1	-0,099219212	0,678403135
APOD	-0,099205841	0,536520199
ZAP70	-0,099129866	0,860212382
LOC128192	-0,099062953	0,269494564
LOC254531	-0,098778698	0,815384333
BTBD1	-0,098656563	0,514968106
GLP1R	-0,098558907	0,408153457
BCL7C	-0,098545506	0,406849211
LOC729417	-0,098491967	0,788932682
LOC285033	-0,098472858	0,295713258
KRTAP15-1	-0,098439226	0,747451507
DGCR6	-0,098434201	0,406093345
CLEC4A	-0,09829385	0,759440898
RFK	-0,098210393	0,139389383
LOC402057	-0,098203659	0,781538712
LOC90701	-0,098148768	0,614531063
MBNL1	-0,098108644	0,573605487
KRTAP5-10	-0,098075533	0,999989522

HSZFP36	-0,097988998	0,601977105
ENDOGL1	-0,097939554	0,331946339
WBSCR23	-0,097711171	0,380940344
LOC729966	-0,097684531	0,352391523
MGC22014	-0,097683663	0,267951543
YPEL2	-0,097582241	0,363518617
CILP2	-0,097567532	0,229172933
RABEP1	-0,097369385	0,2074361
DEFB114	-0,097359885	0,504532525
TOP1MT	-0,097300027	0,887860153
CD55	-0,097252385	0,166803742
PALM2	-0,097178655	0,467389591
FAM110B	-0,097124655	0,13388957
ARPM1	-0,0971099	0,110581404
MYH13	-0,096989157	0,457087142
PARP6	-0,096968719	0,191670165
LOC643506	-0,096847115	0,670134607
RBPSUHL	-0,096664459	0,98627748
KIAA2013	-0,09658405	0,6380619
DPM1	-0,09652027	0,785540691
REXO1L1	-0,096413133	0,539016177
FOXN4	-0,096378863	0,621889596
LOC730056	-0,096324145	0,514905422
BRS3	-0,096297029	0,667360075
GSTZ1	-0,096293534	0,394213665
P2RX3	-0,096262835	0,810472724
LOC441964	-0,096214587	0,907833197
LOC729295	-0,09620552	0,475103843
ZNF599	-0,096140646	0,918289008
C3orf31	-0,096105446	0,535486513
LOC644224	-0,096020434	0,813957316
SPAG11B	-0,096004362	0,314142354
LOC728796	-0,095832256	0,703626669
SNX21	-0,095787474	0,618173282
LOC728169	-0,09575877	0,35608001
FAAH	-0,095756478	0,593629389
FAM122A	-0,095751182	0,18889921
TLE4	-0,095520476	0,853349959
SOX14	-0,095355377	0,838973289
FAM83E	-0,095300358	0,58476886
HIST1H2AJ	-0,095275064	0,733429332
DHRS3	-0,095254326	0,679305981
LOC256483	-0,095238841	0,561446484
AQP6	-0,095228942	0,435565974
MBNL3	-0,095135605	0,532031078

LOC728350	-0,095030441	0,293390542
DPYS	-0,094923474	0,313501798
HPRT1	-0,094867235	0,680903039
TAF8	-0,094839292	0,780169548
SOX15	-0,094838703	0,444135418
TRIM4	-0,094780221	0,595136479
DEFA5	-0,094741653	0,259945394
C8orf76	-0,094697939	0,897430616
ADCYAP1R1	-0,094621778	0,230683565
SCCPDH	-0,094610763	0,637651873
hCG_1790806	-0,094469949	0,569366682
RHOA	-0,094430351	0,77517024
FAM47B	-0,094394263	0,350685878
UNG	-0,094343942	0,352272132
FCHO2	-0,094292527	0,339050176
GPX1	-0,094201918	0,565447094
LOC729376	-0,0940622	0,794104705
BRCA2	-0,094035702	0,369258147
HGNT-IV-H	-0,09402249	0,174982041
GFRAL	-0,094011611	0,254609805
SNX12	-0,093925404	0,37599278
LOC729253	-0,09385264	0,542357454
ASB4	-0,093811503	0,806340659
TNFAIP2	-0,0937893	0,510600044
LMNA	-0,093712239	0,345167189
MAGEB10	-0,093592914	0,321458328
GP2	-0,093317823	0,730360092
WNT2	-0,093174741	0,162761998
TUBE1	-0,093152223	0,921460673
LOC646916	-0,093112315	0,735878007
SERPINA3	-0,093110633	0,342497836
MAP4K5	-0,093086234	0,607554507
TGM5	-0,09293479	0,696581608
TSPAN16	-0,09293131	0,447750284
LOC642024	-0,092927881	0,883542511
SNX2	-0,092902715	0,26448079
C12orf52	-0,092851611	0,306458592
FLJ43860	-0,092548399	0,614563735
KHSRP	-0,092541663	0,361259672
PRDM16	-0,092500624	0,897373687
HCG3	-0,092497539	0,726795362
NID	-0,092409812	0,828471082
NOLC1	-0,092408446	0,768303405
ERBB4	-0,0923045	0,42420944
FBXL5	-0,092279817	0,288114318

LOC647089	-0,092113393	0,951849468
PRNT	-0,092034417	0,315820056
NAGA	-0,092025819	0,411008121
GPR50	-0,09202419	0,134260029
PADI4	-0,091927786	0,499154258
FLJ22675	-0,091834889	0,323066367
HERV-FRD	-0,091833702	0,363635893
WFS1	-0,091816494	0,438021166
GINS4	-0,091795834	0,651118411
PUSL1	-0,091790839	0,96898516
RIPK5	-0,091748545	0,895001977
C10orf79	-0,091728359	0,384387922
ALOX5AP	-0,091693418	0,207647342
OR1S2	-0,09167757	0,463348617
FAM46D	-0,091612251	0,891101575
ARPP-19	-0,091552952	0,278221445
IFNA10	-0,091341408	0,224898523
GLDN	-0,09129735	0,315002728
HCLS1	-0,091171183	0,164406309
RGAG1	-0,091136349	0,29744879
IFNA21	-0,090997486	0,456577702
OR2B2	-0,090906753	0,188388723
LHFPL2	-0,090892639	0,530862576
SNF8	-0,090825173	0,963021228
LOC646626	-0,090740755	0,13364008
SIP1	-0,090718012	0,159923192
MGC57346	-0,090705195	0,591508117
C1orf113	-0,090668061	0,31296325
PTTG2	-0,090633607	0,554517311
BICD2	-0,090581073	0,539671989
FGF16	-0,090443181	0,391293948
BTF3	-0,090390493	NA
SORT1	-0,090372225	0,342704751
DDX27	-0,090336578	0,86923919
ZNF390	-0,090334054	0,338164953
CASC1	-0,090311878	0,859545516
LOC653135	-0,09027556	0,372817444
NSMCE1	-0,090169113	0,220666482
CCDC72	-0,090164455	0,28587004
LOC727925	-0,090100895	0,722823842
Sharpin	-0,090062326	0,68091811
MS4A8B	-0,089983384	0,185207583
LOC728676	-0,089919101	0,88808924
PIK3C2G	-0,089908285	0,16248252
ATP12A	-0,089581277	0,753349884

LOC645984	-0,08955548	0,236487273
TLN2	-0,089509646	0,236806827
CA13	-0,089461683	0,730986123
FLJ12993	-0,089444459	0,347334847
TRIM48	-0,08942757	0,334783346
USP27X	-0,089320989	0,879790294
OSTF1	-0,089310579	0,33185256
ALOXE3	-0,089302726	0,413264164
UCP3	-0,089262404	0,42931901
LOC344167	-0,089245764	0,275543275
PIGG	-0,089143898	0,543169971
NPAL3	-0,089128025	0,922309394
LOC732398	-0,089017153	0,375654494
CUL3	-0,088903952	0,650998195
RAP1B	-0,088881403	0,326403114
ITM1	-0,088821963	0,353429482
GABRE	-0,088770255	0,707970165
IL17R	-0,088763897	0,850574598
NEU4	-0,088723938	0,950289567
RHO	-0,088713051	0,192917034
LRRC28	-0,088680288	0,887304876
LOC728727	-0,088588523	0,124520284
SLC13A3	-0,088583842	0,738292632
LOC728755	-0,088543435	0,174593571
SEC14L5	-0,088502374	0,723970816
C14orf181	-0,088478682	0,43535461
CYP4F3	-0,088418248	0,381196652
HOXC9	-0,088410348	0,206919696
CCDC19	-0,088338234	0,646861083
LOC644119	-0,088236701	0,174635842
LOC729296	-0,088154024	0,852177962
HIRIP3	-0,088064131	0,834021771
C16orf84	-0,087991475	0,189766197
LOC728342	-0,087820176	0,905335415
C22orf29	-0,087697506	0,599580312
FAF1	-0,087557943	0,230168789
C1QTNF7	-0,087407685	0,388260533
SERPINB3	-0,0873563	0,936335986
EMR4	-0,087339785	0,935400183
ATP6V0A2	-0,087310662	0,958456981
IKBKE	-0,08728074	0,262242462
ZNF594	-0,087258284	0,431527954
CPN1	-0,087032128	0,977001542
MRPL19	-0,086982088	0,466122535
FAM58B	-0,086906098	0,639089905

C17orf48	-0,086892298	0,793571858
RP11-592B15,4	-0,086874703	0,606739159
CDC42SE2	-0,086775356	0,986783643
TOX	-0,086763937	0,459886468
CCDC18	-0,086679866	0,283424203
ATP6V0E	-0,086615147	0,962141659
N6AMT2	-0,086545943	0,157076471
RFPL2	-0,086508436	0,964002831
LARS2	-0,086486323	0,897380639
LOC644100	-0,086480622	0,880628777
KIAA1257	-0,086473696	0,256723524
LOC728801	-0,086408328	0,971140601
RALGPS2	-0,086305294	0,319193009
IL12A	-0,086280252	0,523221277
TCEAL7	-0,086250246	0,540878233
OR9I1	-0,086228835	0,243789146
EXOSC10	-0,086135137	0,985327077
ADRM1	-0,086097423	0,993465491
SNRP70	-0,086075558	0,426491928
C11orf63	-0,085748419	0,247066487
SC4MOL	-0,085667727	0,64744984
LOC729358	-0,08546101	0,700191968
THEDC1	-0,085424271	0,518809419
HAVCR1	-0,085409094	0,66375034
C11orf36	-0,085390355	0,409249761
CRY2	-0,085281732	0,451920597
GC	-0,085243922	0,560778396
LOC340113	-0,085220092	0,265234097
FBRS	-0,085189425	0,140998525
NPR1	-0,085183601	0,44088909
QDPR	-0,0851131	0,272798061
C5orf23	-0,085102163	0,804073241
ZNF628	-0,084947223	0,731072265
SFTPA2	-0,084907356	0,773740588
IRX2	-0,084698745	0,586286166
PHACTR1	-0,084639578	0,795716697
C11orf60	-0,084637741	0,604726589
TAS2R14	-0,084570348	0,519343896
PDZRN4	-0,084439601	0,412426715
SIRT6	-0,084427488	0,432807319
SSRP1	-0,084396432	0,76025991
FLJ34658	-0,084335853	0,930526195
LOC439994	-0,084169234	0,495468463
GJE1	-0,084092356	0,904867762
OR9G1	-0,084049979	0,226618455

MT1M	-0,084030737	0,753896587
PDCD1	-0,084030737	0,142224266
AGGF1	-0,084029905	0,664424639
LOC390937	-0,083951506	0,987501229
COL9A1	-0,083946914	0,15132698
ZFP90	-0,083821829	0,267158557
LOC728349	-0,083768568	0,810940793
MCF2	-0,083767596	0,426739701
NDNL2	-0,083758154	0,503761133
KIAA1797	-0,083721613	0,575193609
GADL1	-0,083715105	0,46022546
PTP4A3	-0,083682191	0,816316332
RPL37A	-0,083642863	0,602413134
PGK1	-0,083635439	0,478528847
MAP7D3	-0,083626084	0,34192082
ZIC3	-0,083597879	0,441175234
MSI2	-0,083594725	0,228323845
KRT15	-0,083547484	0,188875478
COX5A	-0,083518782	0,550274673
SDR16C6	-0,083497161	0,92783791
C2orf24	-0,083237368	0,345527598
LOC90113	-0,083236986	0,106629324
RGS3	-0,083227103	0,574125655
LOC731647	-0,083203685	0,486669067
ARID3C	-0,083174366	0,875088708
ENTPD3	-0,083094248	0,375766944
YPEL1	-0,083025498	0,458833481
CD22	-0,082993736	0,129544998
GDI2	-0,08297223	0,526678279
GDAP2	-0,082938309	0,551509488
DESC1	-0,082911027	0,467674616
NPY2R	-0,082894968	0,688558144
HLA-A	-0,082857869	0,246018516
SLC35C2	-0,082822944	0,623227614
LOC402269	-0,082755592	0,395429344
C11orf49	-0,082667607	0,634228386
LOC731348	-0,08247798	0,330511051
VSIG1	-0,08245171	0,256506459
PPM1D	-0,08243513	0,661463018
TMEFF2	-0,082313789	0,727242072
OR6Q1	-0,082150743	0,818883577
SLC38A11	-0,082102133	0,795210431
HOXA6	-0,082079364	0,417418735
PHF10	-0,082068006	0,790305468
C9orf75	-0,081941994	0,99205006

hCG_2040224	-0,081896076	0,447274296
CLDND1	-0,081773701	0,269579477
CETN2	-0,081771237	0,637050102
NTE	-0,081731207	0,460690087
CA5A	-0,081703335	0,549884237
TINP1	-0,081615662	0,179206859
USP19	-0,081586361	0,252163995
DDR1	-0,081465567	0,440421506
LOC728008	-0,081422529	0,26393131
FADS1	-0,081151317	0,6893184
LOC400456	-0,081101908	0,194932905
NAT15	-0,081031056	0,310287004
LOC643599	-0,080987039	0,943336529
LOC652766	-0,080982804	0,361702613
SLC26A6	-0,080950435	0,132843146
PPBP	-0,080921743	0,657611763
LOC642681	-0,080892744	0,267836055
BCORL1	-0,080678179	0,383493919
FLJ11183	-0,08061097	0,764861293
ARL6IP1	-0,080452333	0,399036998
KCNQ4	-0,080440289	0,354430959
GLMN	-0,080430572	0,405412094
NEDD4L	-0,08028869	0,394608474
CADPS	-0,080283172	0,423550888
TRIM50A	-0,080174115	0,916065932
LOC729541	-0,080134493	0,808990219
TF	-0,080085383	0,32658692
CCL24	-0,080038484	0,309104858
P2RY12	-0,079893289	0,501971592
LOC729085	-0,079866435	0,627513412
OR4D1	-0,079802362	0,820492994
LOC137885	-0,079794086	0,165033886
C14orf45	-0,079575106	0,940810062
LMO3	-0,079544031	0,892379012
SYTL4	-0,079438594	0,368183634
UNQ9438	-0,079307791	0,559727852
SLC25A1	-0,079199936	0,529480037
MFAP3	-0,079183916	0,638521647
GCK	-0,079162901	0,79992025
CKM	-0,079117843	0,143257793
SMAD7	-0,079109394	0,764043978
NEO1	-0,079088555	0,728515594
GJA8	-0,078951271	0,865876408
TRH	-0,078940445	0,157488181
CYP2R1	-0,078921378	0,864858593

CEACAM1	-0,078919596	0,35677498
LOC729967	-0,078890499	0,714083014
FAM120AOS	-0,07884282	0,551378815
LOC645839	-0,078714294	0,818161338
ANKRD42	-0,078675514	0,308889597
HMGN3	-0,078552611	0,671859059
C4orf40	-0,078551535	0,166913483
FAM26E	-0,078382389	0,478992458
NAGK	-0,078368382	0,735672489
SNX30	-0,078175692	0,189444485
C4orf16	-0,078146595	0,244501659
BBS12	-0,078095384	0,670584722
ZXDA	-0,078007354	0,300897852
ARF4L	-0,077995774	0,722868236
TMEM151A	-0,07797366	0,95234986
DNAJC19	-0,077836107	0,9603372
C1QA	-0,07777257	0,50450373
PLD6	-0,077693837	0,492386663
PODXL	-0,077684944	0,707779559
KIAA0556	-0,077674497	0,821044064
WWC1	-0,077658642	0,344972849
TAS2R9	-0,077502361	0,642454137
CYP4F2	-0,077501765	0,872745608
COL16A1	-0,077484736	0,58335162
SP140	-0,07724788	0,762611521
KLF4	-0,077224356	0,358241389
SCN1B	-0,077187398	0,876712246
SPINK5	-0,077145418	0,531793611
PLA2G1B	-0,076975388	0,778646905
WIPI1	-0,076913435	0,653111085
EDG7	-0,076687461	0,237333595
TMEM167A	-0,076564714	0,483857767
GCA	-0,076563128	0,209237774
SLC12A2	-0,076552775	0,800388113
CENPH	-0,076512398	0,290974447
TREML4	-0,076463919	0,954173847
MUC2	-0,076410654	0,946136613
RNF128	-0,076338663	0,60038463
GAS2	-0,076271875	0,21986676
CCDC116	-0,076245222	0,972603919
CDKL5	-0,076236543	0,454528252
ZNF257	-0,076170285	0,953037695
OPN4	-0,076081066	0,564660508
CCDC27	-0,07607886	0,303630532
LOH11CR2A	-0,076045345	0,63800965

KRTAP4-4	-0,076031185	0,558651449
FALZ	-0,075899282	0,790538599
KIAA0101	-0,075857955	0,464964016
SETD5	-0,075855656	0,345180052
UMODL1	-0,075765914	0,611128797
LOC730222	-0,075728415	0,452082634
LOC729239	-0,07568118	0,422778158
MGC70863	-0,075616127	0,353938745
USP25	-0,075524524	0,989862996
C7orf63	-0,075409556	0,295370603
LY6G6F	-0,075340735	0,332352404
hCG_15200	-0,075271016	0,257834622
BFAR	-0,075206959	0,908183636
TNNT2	-0,075154744	0,545082371
RHOF	-0,075130028	0,316559755
TRIT1	-0,0751126	0,945408571
LOC729467	-0,075046473	0,746958049
LTBP4	-0,074573707	0,848629036
FLJ36157	-0,074535761	0,156343985
JARID2	-0,074413245	0,446876779
NEGR1	-0,0743281	0,522225537
RRAGB	-0,074294772	0,337289844
AGPAT2	-0,074245413	0,18028752
LOC649201	-0,074017486	0,5483304
C17orf70	-0,073945772	0,455153441
FLJ22374	-0,073939664	0,259875402
LOC393078	-0,073846187	0,519660886
LOC729630	-0,073822016	0,309470261
RAPSN	-0,073737631	0,170666536
GNG5	-0,07372288	0,50584858
INCENP	-0,0735971	0,002193142
CXorf36	-0,073510706	0,271256258
UTP15	-0,073420309	0,7099172
MGC15885	-0,073296617	0,910473372
BACE1	-0,073247344	0,35290171
CORO1C	-0,073231372	0,88865296
TMEM95	-0,073117674	0,69935658
LOC728530	-0,073004527	0,627380868
LOC442590	-0,072918022	0,634473982
TYRO3	-0,072857744	0,878756833
ABL1	-0,072854412	0,802403778
FLJ43806	-0,072818732	NA
C9orf95	-0,072816284	0,32830618
KIAA1045	-0,072719946	0,437275387
FAM150A	-0,072688125	0,866442123

DBI	-0,072677424	0,543441248
CNOT6L	-0,072674934	0,416195637
LOC440597	-0,072667049	0,218968527
C11orf87	-0,07264992	0,179111565
LOC728870	-0,072563605	0,735148314
C22orf40	-0,072500673	0,473558312
BRIP1	-0,072378368	0,808713066
NCOA6IP	-0,072369256	0,438277994
LOC728427	-0,072357212	0,250343984
RUFY4	-0,072212247	0,690912069
RTKN	-0,072028165	0,866408792
POLA	-0,071997012	0,994503834
CDC2L5	-0,071954024	0,321680336
MYLK4	-0,071886914	0,614662441
KLHL5	-0,071826505	0,553955699
FLJ10514	-0,071688204	0,418112825
RODH-4	-0,071683379	0,538165027
ZFYVE20	-0,071598996	0,299510986
CHM	-0,071572621	0,20633693
FOXF2	-0,07151131	0,826700662
SUPV3L1	-0,071504007	0,331781072
TBC1D19	-0,071460371	0,263480223
STIM1	-0,071428922	0,545609374
ZMYM4	-0,071385737	0,162674146
RAD51AP1	-0,071350811	0,173346874
LOC643717	-0,07130797	0,497471807
FBXO22	-0,07096016	0,980117361
LOC729056	-0,07089295	0,677557236
SEC61A2	-0,070773331	0,430135146
PPIH	-0,070741803	0,800156727
INPP5B	-0,070724438	0,160760693
PFKFB2	-0,070652776	0,529465404
MPP7	-0,070590667	0,980630392
LOC728726	-0,070528142	0,609105047
C9orf82	-0,070517119	0,774244381
ABCA17	-0,07039527	0,474318586
C3orf22	-0,070296081	0,15457673
CASC4	-0,070246847	0,756707799
YWHAG	-0,070204522	0,445716076
NADSYN1	-0,070143433	0,560525563
MYL4	-0,070127139	0,750929029
FLJ45831	-0,070062849	0,674084463
NME2	-0,070019502	0,740677825
SLC17A2	-0,069976687	0,459477542
TMEM141	-0,069849818	0,193513618

LOC652355	-0,069809864	0,541956673
LOC651033	-0,069683871	0,261887871
MKL2	-0,069665013	0,60159592
CD40LG	-0,069599668	0,512483425
ELN	-0,069527292	0,601618325
MYH10	-0,069514591	0,363893661
TRAF4	-0,069474132	0,975898741
LOC728845	-0,069449434	0,334796199
MESDC1	-0,069342812	0,303557965
QRSL1	-0,069202468	0,553163748
CARM1L	-0,069175576	0,709475567
FLJ44817	-0,069141474	0,67715103
CTXN3	-0,068954368	0,589876973
DPY19L2	-0,068828227	0,624407771
WDR16	-0,068770547	0,508555466
LOC645877	-0,068763883	0,291378184
DEPDC1	-0,068742769	0,770196218
MGC43026	-0,068688869	0,205105289
DKFZP686E2158	-0,068632857	0,73926078
TPI1	-0,068538311	0,687886324
FZD2	-0,068417241	0,447975686
TLP	-0,068391406	0,56733723
TCTEX1D1	-0,06837032	0,950494533
C12orf39	-0,068345603	0,379684552
NDUFC2	-0,068263301	0,473348436
DHPS	-0,068257246	0,257810437
GRM3	-0,06818613	0,327055998
FASTKD1	-0,068123898	0,755902829
tcag7,441	-0,068087662	0,684401398
MAP1B	-0,068012931	0,998741975
COL4A1	-0,067892113	0,467091679
GZMK	-0,067771048	0,50087492
QTRT1	-0,067744884	0,323227249
MUC5AC	-0,067722979	0,901782765
ENTPD8	-0,0676577	0,786041705
FAM135A	-0,06754351	0,182973475
SEMA4D	-0,067542362	0,247402037
FLJ20323	-0,06733128	0,579020836
MLL	-0,067294928	0,96374718
SR140	-0,067288224	0,969876264
GJB3	-0,067270347	0,256013331
LOC649133	-0,067253897	0,683929976
NIPSNAP3B	-0,067225254	0,610574236
RP11-529I10,4	-0,066947191	0,796154241
C16orf5	-0,066743379	0,727738047

ENPEP	-0,066692931	0,720400989
ALS2CR16	-0,066550353	0,305649123
ZNF193	-0,066536544	0,278629613
SEPN1	-0,066484842	0,527968069
SUPT16H	-0,066456568	0,396227517
GPR158L1	-0,066377565	0,272655545
TCN1	-0,066356321	0,667072289
BTAF1	-0,066323816	0,424759331
SLC35A3	-0,066303877	0,250353405
EMP3	-0,066301598	0,222194475
SLC1A1	-0,066293121	0,776318454
SLC20A2	-0,066225189	0,893921114
PPAPDC1B	-0,066119163	0,589188457
CDKN2AIPNL	-0,066073456	0,309774577
PHOSPHO1	-0,065913216	0,385498181
PARK2	-0,065909332	0,175358101
POU1F1	-0,065811104	0,375110599
LOC727862	-0,065809665	0,306722489
LOC644153	-0,065764622	0,492458181
OAF	-0,065752519	0,590264591
SC5DL	-0,065708835	0,352119934
C7orf55	-0,06570032	0,296582965
SLMO2	-0,065546581	0,844684626
VDAC2	-0,065546338	0,231316358
C10orf84	-0,065424195	0,167185177
DDX3Y	-0,065385899	0,449274475
CD109	-0,065381776	0,341478543
DLGAP5	-0,065293756	0,930666497
METTL11A	-0,065279791	0,600190249
PLXDC1	-0,065203284	0,396688597
OR13C5	-0,065160743	0,358176265
PPT1	-0,065074097	0,35434364
hCG_1644301	-0,065063338	0,912333576
LOC652637	-0,065060761	0,243499722
ZNF268	-0,064996645	0,510907374
HCN4	-0,064898867	0,939414136
A1CF	-0,064887619	0,560284861
STMN4	-0,064838082	0,338556622
NTN5	-0,064761179	0,30209056
GATS	-0,064662277	0,667554559
CRADD	-0,064576376	0,767917328
GFI1B	-0,064535965	0,68994281
CDSN	-0,064327038	0,254942517
PRDM1	-0,064139889	0,864612578
RRAD	-0,064118319	0,805689533

NLRP9	-0,064091798	0,630587327
TANK	-0,064047475	0,513571856
MAZ	-0,064037177	0,837395312
LOC728763	-0,063898448	0,977783273
C4orf27	-0,063835221	0,664594938
KLHL7	-0,063778583	0,429883024
LOC644357	-0,063681497	0,200567802
FGF23	-0,063670415	0,422064397
TMEM135	-0,063628363	0,282757781
C6orf210	-0,063575964	0,966455316
OR8D4	-0,063435594	0,207365411
TMCC1	-0,063357417	0,2236652
GPR62	-0,063297899	0,437636092
IRGC	-0,063297537	0,141735792
DDX21	-0,063271777	0,821312308
LOC728998	-0,063243027	0,86482484
COMMD8	-0,063238633	0,699520936
BAG3	-0,063216513	0,4976143
hCG_1651889	-0,063190558	0,930945475
NT5DC1	-0,063080034	0,933294536
KIAA0859	-0,063068011	0,683065633
AFAP1	-0,063050445	0,916577861
C1orf105	-0,063039965	0,17788584
LOC441268	-0,062937306	0,320723484
MIER2	-0,062914155	0,792712604
SFRP4	-0,062853076	0,461765489
APC	-0,062821927	0,354242815
hCG_1789710	-0,06278388	0,789302674
DLST	-0,062733214	0,354087694
ADAD2	-0,062621547	0,33874393
DCDC5	-0,062488002	0,270849506
TUBA8	-0,062482084	0,901256196
EPO	-0,06246806	0,189144317
LOC644634	-0,062420713	0,276347022
IVL	-0,062347366	0,491341167
EIF4EBP1	-0,062338105	0,394202484
LOC400891	-0,062328694	0,178796181
CCNI	-0,062210275	0,795533248
BMP7	-0,062057256	0,840733724
C6orf192	-0,061980082	0,923487972
TFAP2BL1	-0,061950958	0,895780512
FAM18B2	-0,061900828	0,774211116
STEAP1	-0,061890495	0,509038986
CHCHD4	-0,061865312	0,617398217
ABCB10	-0,061818547	0,924271095

GNG2	-0,061779905	0,978499916
C2orf37	-0,06172373	0,696858282
DNAH14	-0,061717111	0,821546417
C11orf54	-0,061703765	0,142263986
C5orf13	-0,061591892	0,201909885
PF4V1	-0,06154057	0,473986291
KMO	-0,061533347	0,822183784
NCF2	-0,06139314	0,747183989
FLJ46284	-0,061285331	0,425155031
MAPK9	-0,061204437	0,891833399
LCA5	-0,061200744	0,242281726
hCG_1643176	-0,061126608	0,525612258
NDUFS4	-0,061091247	0,195856376
FEM1B	-0,061082245	0,562287154
ZNF624	-0,06091932	0,828511358
PCM1	-0,060848968	0,575831073
SLC12A8	-0,060778476	0,819323595
LOC729782	-0,060664249	0,31963324
BAT1	-0,060623684	0,414728033
SLC16A11	-0,060345237	0,586671138
LOC285697	-0,060303122	0,383531784
LOC728617	-0,06005213	0,547335705
LOC731632	-0,060033605	0,206957628
POP1	-0,06000294	0,710893692
RBP1	-0,059755527	0,402124301
UCN3	-0,059736272	0,409208385
THBS4	-0,059522038	0,47866788
ANKRD39	-0,059466768	0,182542436
LOC649217	-0,059447394	0,311024511
C6orf97	-0,059274912	0,896367532
TOX2	-0,059109605	0,525371538
hCG_1980447	-0,05903714	0,9266037
LOC401233	-0,059029456	0,415970687
PRAC	-0,05894503	0,695146617
GARS	-0,058843461	0,435621547
CHMP2B	-0,058819711	0,503678782
NCOR2	-0,058795592	0,939444395
RASGRP3	-0,058767633	0,374555211
LOC728977	-0,058747032	0,535436228
PCDHGA12	-0,058731725	0,877911405
LOC729095	-0,058683432	0,455523944
MDM2	-0,058666122	0,416163884
MTPN	-0,058651063	0,462568486
MAP3K8	-0,058571413	0,956314971
SAH	-0,058491472	0,908420015

LOC441742	-0,058293261	0,844172764
LOC730817	-0,058288898	0,939321574
PAX1	-0,058284297	0,911318326
LOC729870	-0,0582013	0,238018882
LOC732223	-0,058111928	0,218223423
MAOB	-0,058028894	0,944064934
SUSD3	-0,057985095	0,525776541
DDHD1	-0,057959935	0,280668357
GNB1L	-0,057903361	0,359374477
FBXW2	-0,057889744	0,143089377
LOC730194	-0,057822318	0,142504949
SAMD8	-0,057773517	0,457527375
TMEM91	-0,057685096	0,23200864
LOC284577	-0,057672184	0,849725336
C1orf131	-0,057511559	0,491061746
CDCA7	-0,057497846	0,382715947
C13orf15	-0,057433884	0,339716459
ARHGAP26	-0,057350554	0,336163521
PRSS23	-0,057333998	0,93698202
FUNDC1	-0,057274577	0,849074384
LOC646253	-0,057217519	0,164933746
LOC441108	-0,057152107	0,485525454
ITIH3	-0,057150558	0,948737286
LOR	-0,05709837	0,336207459
C1orf160	-0,056814663	0,521978273
YTHDF1	-0,056614339	0,683623975
RAD50	-0,056552776	0,91632227
C20orf70	-0,056431526	0,935483865
FGF3	-0,056397842	0,198801837
KRT1B	-0,05637857	0,473966438
TMEM138	-0,056317601	0,449283759
TRIM61	-0,056203881	0,595098372
GNAI2	-0,05615429	0,321415499
REL	-0,056133788	0,440991171
IL17C	-0,056043776	0,418334049
TMEM106B	-0,055592226	0,461568124
SLC4A2	-0,055589787	0,81834708
NMNAT1	-0,055433877	0,413204292
CASP9	-0,055377501	0,973994761
ch-TOG	-0,05531381	0,804665384
LOC647471	-0,055303788	0,713885514
CASQ2	-0,055273767	0,677075585
GPR119	-0,05522126	0,9526877
C8orf4	-0,055173182	0,19625491
RARRES1	-0,05513197	0,293547032

FLJ45079	-0,05508928	0,267699275
SPATA5L1	-0,055081527	0,752057123
KPNA3	-0,055046541	0,904986564
DNCI1	-0,055011307	0,732148436
PTPN14	-0,054874091	0,421229188
RBM15	-0,05480993	0,738366743
TEX264	-0,054720708	0,91982557
LOC645811	-0,054647916	0,245347702
KCNK5	-0,054621254	0,391629097
LGP2	-0,054620698	0,968333831
TXN	-0,054526533	0,287145482
MAGEA11	-0,054472602	0,420622505
KIAA1305	-0,054452341	0,188433931
WDR47	-0,054417634	0,625325334
NRAS	-0,054334643	0,45521293
TMEM47	-0,054252405	0,336289904
C9orf30	-0,054227758	0,444672362
LOC401410	-0,054012081	0,522033171
LOC732391	-0,053884085	0,646258971
MYH7	-0,053568377	0,672792823
SLC39A6	-0,053535962	0,728580848
ABCC10	-0,053497796	0,256530041
LOC387934	-0,053444821	0,205981214
OR52D1	-0,053436794	0,669330432
hCG_1642624	-0,053410332	0,326694575
FLJ10094	-0,053408731	0,398068889
MED13L	-0,053294199	0,310778476
AGR2	-0,053257318	0,632221058
LRRC58	-0,05315478	0,885139249
SNAG1	-0,053134845	0,321590108
OBP2B	-0,053095346	0,847340264
ABHD8	-0,052926882	0,687010989
LOC146542	-0,052870223	0,330591673
SPAG1	-0,052802817	0,270332011
PMPCA	-0,052732184	0,670875782
COPS3	-0,052715926	0,506576165
TMC8	-0,052667308	0,221062772
PSIP1	-0,052666843	0,751935129
MBD3L2	-0,052624178	0,825066461
ATP2B4	-0,052575297	0,270591576
HSD3B2	-0,052509361	0,433227674
H1FX	-0,052501997	0,370902435
LOC729381	-0,052454282	0,52478012
LOC729318	-0,052442279	0,347540455
SP2	-0,052434382	0,799125209

C9orf91	-0,052386286	0,74248926
WSB2	-0,052364048	0,800791558
LOC646864	-0,052360128	0,543550831
SULT1C2	-0,052357265	0,836419418
DO	-0,052341558	0,167861565
LOC643534	-0,052246156	0,839732888
C16orf38	-0,052230387	0,229165372
C6orf12	-0,052172692	0,629210255
LOC728437	-0,05215337	0,437771688
KPNA2	-0,052145642	0,287755358
FAU	-0,0521456	0,987641274
SYVN1	-0,052096943	0,658259386
GPR49	-0,05208754	0,356742847
MMP16	-0,05206047	0,490602788
C16orf13	-0,052049584	0,222819172
KLK15	-0,052006287	0,323641399
PRKWNK4	-0,051997689	0,672598589
ZNF287	-0,051969844	0,572129562
GLP2R	-0,051915215	0,916671246
DAB2	-0,051912857	0,624235294
COL9A3	-0,05182051	0,336608696
RAB3B	-0,051777534	0,477683749
LOC731568	-0,051745532	0,509518412
NLRP6	-0,051742526	0,98414395
OLR1	-0,051706702	0,881104305
GRIA4	-0,051690748	0,486605216
KRT5	-0,051619836	0,675542253
LOC730089	-0,05156519	0,376057561
C6orf159	-0,051471521	0,395184262
SKB1	-0,051443689	0,828857128
GADD45B	-0,051348278	0,860170164
LOC644511	-0,05133655	0,376147651
C14orf72	-0,051233017	0,244792354
C10orf18	-0,051229429	0,739397713
CCDC25	-0,051228605	0,653359052
SERPIND1	-0,051170246	0,921820588
DHRS4L2	-0,051136063	0,267323421
RNF217	-0,051111834	0,125224133
GNGT2	-0,051084443	0,50564049
FAM5B	-0,051066175	0,946554471
TTC39B	-0,051049832	0,503002213
C17orf58	-0,051019317	0,465251761
C20orf85	-0,050954046	0,628048957
CBFA2T3	-0,050901041	0,576701318
LOC387723	-0,050672654	0,90984188

DAZL	-0,050425616	0,974024483
LOC645799	-0,050425278	0,415082852
PRKAR2B	-0,05040053	0,933313171
SLC9A2	-0,050379119	0,332221654
LOC642781	-0,050288904	0,824923767
LOC652684	-0,050164549	0,49682915
SPDY1	-0,050143169	0,776303511
LOC730123	-0,050134641	0,907865595
PDE1C	-0,050125323	0,583987073
STAT3	-0,049977826	0,506792274
FAIM3	-0,049957767	0,441263596
SNX32	-0,049778973	0,821645596
FBXO31	-0,049621237	0,419386273
OR2L13	-0,04960423	0,973234373
GABRR2	-0,049546445	0,463460471
RPS19BP1	-0,049366416	0,402142074
PRELID2	-0,049349696	0,644190558
C10orf140	-0,049294645	0,819872487
C19orf25	-0,049275523	0,514522059
C2orf65	-0,049092505	0,302535817
MAP3K1	-0,049063951	0,989915015
LOC652968	-0,049039304	0,264104928
ZMYM5	-0,048946242	0,446959737
DKFZp434E1119	-0,048931972	0,675619604
FLJ41046	-0,048877315	0,453472
LOC731531	-0,048870425	0,886761653
VTA1	-0,048869624	0,434189277
LOC644277	-0,048836513	0,279436094
UBXN2A	-0,048749086	0,347910862
EPS15L1	-0,048648226	0,797868034
CSTA	-0,048581769	0,39105983
CTH	-0,048536306	0,682233638
KLF8	-0,048528879	0,730264715
CDC10	-0,048431185	0,314736746
ANKFY1	-0,048352665	0,978230109
LOC646584	-0,048203061	0,619450175
ARD1	-0,04814803	0,185553723
hCG_2036596	-0,047904298	0,549542626
ZNF579	-0,047873263	0,528271468
FLJ45983	-0,047854073	0,21325246
LOC728431	-0,047820081	0,338621119
SPAG8	-0,047745998	0,25138547
MRPL54	-0,047668466	0,178112093
OR6C3	-0,047621281	0,23011254
LOC441124	-0,047557596	0,202875791

BGLAP	-0,047536094	0,890764457
OR2T4	-0,047422105	0,628730881
LOC731866	-0,047256611	0,315426776
ARID1B	-0,047209281	0,315186442
UTP23	-0,047173431	0,70324181
FUT4	-0,04701158	0,952360161
RB1	-0,046951378	0,91972004
B3GNT1	-0,046938435	0,889430312
LOC649179	-0,046835088	0,694977437
LILRB4	-0,046829997	0,884946586
RAMP1	-0,046801884	0,792436125
LOC729922	-0,046801001	0,880732838
CCRL1	-0,046795993	0,330540673
KCNK2	-0,046784481	0,815417979
OR2A25	-0,046750889	0,441546074
INSL5	-0,04673984	0,356640131
POLR2K	-0,046646079	0,919611124
TCF19	-0,046604156	0,763143191
CDRT15L2	-0,046529263	0,264725727
GIMAP7	-0,046499736	0,228012657
LOC729136	-0,046434162	0,396142277
LOC642141	-0,046415797	0,253985006
SLC36A3	-0,046402358	0,587672829
RBX1	-0,046352988	0,801013882
LY6H	-0,046351499	0,552394431
CST9L	-0,046291078	0,855414276
ZNF295	-0,04624585	0,687190842
MYBL1	-0,046233028	0,891915688
RASD1	-0,046209402	0,960478421
STXBP2	-0,046133611	0,172226834
TIGD2	-0,046130095	0,951368912
LOC644173	-0,046092336	0,266696229
FBLN7	-0,046046414	0,631003967
C11orf1	-0,045974674	0,169752879
FETUB	-0,045958312	0,108714184
KBTBD10	-0,045950383	0,577821388
DAP	-0,045948252	0,905893329
FBXL14	-0,045943947	0,440138494
LDLRAD1	-0,045936697	0,649612917
LOC728353	-0,045873057	0,81198728
SLC6A14	-0,045836823	0,638568892
PGBD5	-0,04582767	0,742929525
VDRIP	-0,045817028	0,242893588
POLH	-0,045795519	0,68346268
LOC652460	-0,045793476	0,730900597

TOP3B	-0,045772873	0,85782423
LOC728069	-0,045766286	0,955295596
SNAPC3	-0,045721767	0,723651466
TRPM2	-0,045697547	0,936870872
XCL2	-0,045675312	0,701727657
LOC388780	-0,045552569	0,96220752
NLGN3	-0,045546624	0,519236297
OR14I1	-0,045531768	0,833502835
MYO5B	-0,045498983	0,406158749
OAZ1	-0,045465722	0,660778893
RLTPR	-0,045437162	0,844538932
GNA11	-0,045380282	0,235239235
RASSF10	-0,045362353	0,472747684
NXNL2	-0,045305238	0,464590404
COQ6	-0,045287833	0,652844228
NCBP2	-0,045265297	0,434005276
YPEL5	-0,0449971	0,220550779
PRSS11	-0,044983613	0,247171275
hCG_1790262	-0,044910311	0,866602885
WDR76	-0,044831234	0,448465713
LOC728688	-0,044806031	0,453087439
DRG1	-0,04469265	0,86842741
NUDT10	-0,044686693	0,473354626
RPL38	-0,044601373	0,906560895
GABARAPL1	-0,044486493	0,271955998
LOC650428	-0,044437941	0,457715702
PGD	-0,044281809	0,499664407
CERKL	-0,04423141	0,962323073
LOC340204	-0,044219946	0,360195527
KIAA0247	-0,044181176	0,979482271
LOC728209	-0,044125378	0,62684752
MTDH	-0,044093705	0,209776811
WDR78	-0,04407999	0,180979359
GYG	-0,044006657	0,760992812
KAT3	-0,043964144	0,52857824
NPTX1	-0,043962094	0,988960019
ERGIC1	-0,043960742	0,813147748
RSBN1L	-0,043928527	0,272342297
GPR113	-0,043915963	0,468061246
LOC646823	-0,043914447	0,835926803
LOC730621	-0,043895308	0,568839022
GCAT	-0,043798722	0,226921164
HBB	-0,043767752	0,324108393
STAU	-0,043649551	0,260537453
UPK1A	-0,0436271	0,845653158

FAM124A	-0,043621004	0,689388179
CD3EAP	-0,043615102	0,730145068
XYLB	-0,043609503	0,778030317
RAB36	-0,043410494	0,59039687
PROK1	-0,043324999	0,521435093
ARG99	-0,043273016	0,256044668
DHX38	-0,043206143	0,336810195
MGRN1	-0,043076977	0,964571034
MAP2K5	-0,04303531	0,400541389
HUS1B	-0,043032751	0,783187531
FAM132A	-0,042991994	0,701734506
MYH3	-0,042838468	0,984445534
CRX	-0,042812503	0,430466649
SCN4B	-0,042792144	0,786947278
C17orf54	-0,042673571	0,164996311
LOC731922	-0,04256596	0,914414268
LOC653740	-0,042564517	0,966255437
C1orf64	-0,042531034	0,711552881
C2orf83	-0,042514312	0,293062014
KIF2	-0,042483835	0,986302288
CEP27	-0,042417644	0,749397464
CST3	-0,042348624	0,382817497
PRKCA	-0,042342629	0,799502225
C12orf29	-0,042321361	0,336643893
LOC642242	-0,042271733	0,803485078
C21orf88	-0,042244689	0,633904432
ZNF537	-0,042206742	0,943485178
LRRC8E	-0,042204524	0,592002225
ASCL2	-0,042200483	0,132241698
HPX	-0,042148976	0,969999787
GAMT	-0,042036337	0,673857727
SH2D6	-0,042025834	0,172901945
PRSS22	-0,041989263	0,239429393
TRIM36	-0,04197931	0,700875277
LOC729077	-0,041965932	0,824571054
C10orf63	-0,041906333	0,73404655
CNTN1	-0,041811202	0,805634759
NLGN2	-0,041806303	0,899777472
B4GALT1	-0,041701452	0,803897541
ECE2	-0,041658638	0,649251184
OR2V2	-0,041594618	0,588708679
WDFY3	-0,041582002	0,757664649
DLK1	-0,041579502	0,429859372
LOC727725	-0,041574418	0,175823553
SEC61G	-0,04156063	0,724381165

SFXN2	-0,041512981	0,57378916
CLSTN3	-0,041438027	0,578766313
WRB	-0,041412357	0,396143501
MC3R	-0,041280963	0,88737431
IL11RA	-0,041256478	0,738954706
CCM1	-0,041250981	0,97616355
PIGY	-0,04118406	0,335617324
DCK	-0,041134051	0,939869252
NNMT	-0,041040397	0,754427197
LOC730081	-0,04096856	0,331337104
C15orf59	-0,040927831	0,786238333
SEPP1	-0,040814813	0,854561762
LOC645941	-0,040771224	0,798614521
OR4F3	-0,040731276	0,891011631
LACRT	-0,040713346	0,541983362
ROPN1	-0,040672018	0,289011583
LOC646262	-0,040611393	0,94571815
MSCP	-0,040610547	0,159857183
FLJ31813	-0,040586618	0,749132081
NT5E	-0,040571612	0,884567536
KIR3DL1	-0,040526487	0,976838673
REG1B	-0,040481715	0,878313773
FANCD2	-0,04048024	0,955259477
EXOC5	-0,040404648	0,570751361
UGT1A10	-0,040389252	0,41580552
LOC645210	-0,04034697	0,492661294
MGAT1	-0,040232345	0,609180382
HIST1H2AE	-0,040192612	0,658873675
LOC729269	-0,04016526	0,821596739
DSC1	-0,040103227	0,981620339
LOC731813	-0,040066663	0,606014723
SIAT8C	-0,040013699	0,314890153
GRWD1	-0,039957009	0,997960766
LRRC7	-0,039842991	0,352056314
LOC646698	-0,039795269	0,124363698
MRPL50	-0,039695913	0,696504791
SLC6A18	-0,03965666	0,99596554
PIP5K1B	-0,039637815	0,497772509
C17orf81	-0,039422425	0,544853486
RPS27L	-0,039411122	0,876410315
FKHL18	-0,039406695	0,497260304
MXD3	-0,039299769	0,644150209
LOC729657	-0,039142904	0,920236184
GALNTL1	-0,039142323	0,286775791
TNIP3	-0,039129537	0,524820741

LOC729336	-0,03912496	0,669123158
GLOD4	-0,039087016	0,675133906
C11orf46	-0,038868454	0,710349643
LOC646508	-0,038835526	0,532293071
DMN	-0,038779329	0,49981586
CDK4	-0,038762753	0,579121895
SLC5A7	-0,038756446	0,416428552
SLC22A15	-0,038756446	0,689541562
HOXA5	-0,03874992	0,225424399
C13orf3	-0,038628949	0,869240298
RAG1	-0,038454422	0,804977516
LOC730091	-0,038263435	0,616912415
ACVR2	-0,038062291	0,924492921
hCG_1818237	-0,038049487	0,395144341
LOC730995	-0,037929029	0,304829842
CPNE4	-0,037724355	0,322630095
MAD2L2	-0,037715949	0,703581627
SNTG1	-0,037708657	0,579012949
TCL1A	-0,0376331	0,41317652
LOC730416	-0,037595236	0,981009626
CBL	-0,037590185	0,741282556
LOC147710	-0,037577611	0,844397891
SLC4A7	-0,037560829	0,487807596
LOC727950	-0,037542181	0,853221165
TMEM120A	-0,037338987	0,549444208
PSG8	-0,037079921	0,839127005
LOC643825	-0,037036231	0,259340277
C1orf144	-0,037011081	0,969986411
KRT222P	-0,036865385	0,665669402
KANK4	-0,036856379	0,415075494
LOC401021	-0,036759199	0,266930209
MGC48332	-0,036748423	0,794007613
CD2AP	-0,036679943	0,201476428
XCL1	-0,036655103	0,296691279
CIC	-0,036629301	0,988774487
ProSAPiP1	-0,036587797	0,72571141
ELA2B	-0,036510625	0,416330082
TMEM142A	-0,036371455	0,77041783
PARP2	-0,03637144	0,576096916
C12orf69	-0,036215602	0,643034834
LOC643514	-0,036179697	0,820028633
LCK	-0,036142379	0,452966223
CMKLR1	-0,036085679	0,34755634
ACTRT1	-0,03602847	0,446247067
CAV1	-0,035988019	0,57034938

DEFB131	-0,035975651	0,398949681
MBP	-0,035846265	0,805938345
LOC651763	-0,035816661	0,652362689
ADCY6	-0,035666769	0,737458934
WDR53	-0,035586083	0,287612801
NDUFS7	-0,035538219	0,608208517
CLIP4	-0,035503204	0,589105459
EIF4A2	-0,035417062	0,460225554
HGSNAT	-0,035406294	0,899176442
ITGB1BP2	-0,035244788	0,277389777
MGC2396	-0,035210745	0,968762364
GALNS	-0,035185112	0,854891776
TTYH1	-0,035086868	0,610141986
LOC440132	-0,035071854	0,349122259
C3F	-0,035033202	0,606243039
NAT5	-0,035025545	0,614952741
BAZ1B	-0,034999994	0,894663892
LOC728335	-0,034983647	0,652288007
AKR1D1	-0,034865191	0,58541519
EFG1	-0,034812537	0,588439466
NKAPL	-0,034750968	0,981921542
L1CAM	-0,034747893	0,462203359
TMEM213	-0,034716255	0,368370776
GTSE1	-0,034676613	0,306978761
EVX1	-0,034640625	0,507722793
HPX-2	-0,034608153	0,284056177
MAGEC2	-0,034593645	0,366469386
ZCCHC13	-0,034501319	0,701300765
LOC730571	-0,034484322	0,526687719
SLC16A4	-0,034476823	0,504328132
RPL24	-0,034469024	0,334516494
MRFAP1L1	-0,034413071	0,5759427
hCG_2015956	-0,034412357	NA
YEATS2	-0,03440793	0,950942994
LILRB2	-0,034372696	0,556683917
SPINLW1	-0,034339952	0,561427502
LOC653203	-0,034298589	0,616874138
GLCCI1	-0,034293567	0,471022993
C1orf182	-0,034242697	0,875962495
YBX2	-0,034225061	0,35526402
LOC729887	-0,034126841	0,777907571
INPP5E	-0,034094216	0,915911548
LOC644482	-0,034058178	0,426644297
FZD3	-0,034036392	0,834448212
CENTD2	-0,034018621	0,811361023

NR5A2	-0,03401593	0,434902478
FLJ34048	-0,033949893	0,382656074
CFHR1	-0,033936293	0,966329524
LOC642433	-0,033892675	0,65721933
ABCB9	-0,033846107	0,979545924
SURF6	-0,033803984	0,368218203
TCTN3	-0,033773482	0,42868814
MST1R	-0,033738188	0,247289039
CCDC73	-0,033730491	0,331303608
DPPA2	-0,033707542	0,243354842
HTF9C	-0,033653612	0,802941801
TIFAB	-0,033645191	0,660311741
HP	-0,033593096	0,588086834
LOC729305	-0,033545766	0,212924253
KYNU	-0,033414388	0,804485532
ASCC2	-0,033405297	0,898051404
LCE1B	-0,033394404	0,677324285
CDK8	-0,03336898	0,743900225
LOC731097	-0,033298737	0,497845714
NANOG	-0,033292273	0,275087125
hCG_1981531	-0,033211335	0,305685513
FLJ30851	-0,033187598	0,346017879
SLC25A19	-0,033164163	0,507639039
LOC285346	-0,033157589	0,656393498
FLJ46838	-0,03315282	0,904064211
BAG4	-0,033071538	0,569389154
C1orf38	-0,032958063	0,405401554
PCYOX1L	-0,032897784	0,505775801
DUS3L	-0,032787133	0,458928822
FREM3	-0,032711998	0,659788717
C20orf112	-0,032639084	0,367144261
ADAM19	-0,032616939	0,682182168
RPS25	-0,032614539	0,549322077
HEATR2	-0,032595722	0,812013436
ZNF347	-0,032562112	0,602602101
SLC23A2	-0,032443956	0,626567336
LOC730150	-0,03238613	0,746692172
ANGPT2	-0,032362929	0,626522656
FAM22G	-0,032344778	0,383371197
ARHGAP24	-0,032231603	0,67626159
CBLN3	-0,032103668	0,221016023
CALM2	-0,031979478	0,756593179
LOC442609	-0,031950248	0,367307671
C17orf55	-0,031800428	0,778185966
CYP2S1	-0,031797302	0,847050479

C14orf68	-0,031710539	0,854045384
PPFIA3	-0,03141682	0,539739399
ACER2	-0,031398913	0,631623649
COAS2	-0,031306181	0,176528099
KLK7	-0,031303114	0,90412712
OR51Q1	-0,031212058	0,39009576
LHX9	-0,031087405	0,947028008
MASTL	-0,031084344	0,551321452
CYP2J2	-0,031006369	0,342584263
OBSCN	-0,03090588	0,249174308
TBC1D7	-0,030779348	0,708684125
ZNF11B	-0,030719568	0,787569823
LOC338588	-0,03071652	0,820899379
CACNG1	-0,030709001	0,251219523
FLJ20604	-0,030634666	0,585903524
POLE3	-0,030487943	0,983958016
FLJ20574	-0,030460514	0,780846001
TNFRSF12A	-0,030404251	0,310034729
ATP8A1	-0,030331181	0,582078688
LEMD1	-0,030274367	0,299729455
KIAA0157	-0,030270554	0,732527663
DNAJC28	-0,030263706	0,76648769
MYOM1	-0,030241602	0,219519713
TYSND1	-0,030240471	0,599965665
LOC729430	-0,030239847	0,457727794
DNAJC5B	-0,030231962	0,4025085
CTBP1	-0,030203629	0,998078984
IRF2	-0,030111982	0,394417475
DSCR10	-0,030097346	0,421990106
ZNF442	-0,030064582	0,538899056
CEACAM19	-0,029940555	0,945024374
FTHFSDC1	-0,029862227	0,818574807
TASP1	-0,029841202	0,398077727
DEFB134	-0,029774871	0,173898728
RDH11	-0,029754031	0,169465026
MCEE	-0,029717709	0,508626696
COMMD6	-0,029642445	0,66173881
LOC286528	-0,029531581	0,638660643
PRDM9	-0,029422827	0,802071682
MTSS1L	-0,029413253	0,110436664
LYST	-0,029335596	0,540306745
TIMM17A	-0,029291532	0,904308856
CLCNKA	-0,029288258	0,529827902
MRPS22	-0,029234709	0,645743335
ATAD3B	-0,029119652	0,708315189

C6orf145	-0,029088345	0,58807979
LAIR1	-0,028981396	0,816858751
SERINC4	-0,028980666	0,324477413
LOC645478	-0,028915183	0,995006171
LOC441376	-0,028899149	0,437780547
AMACR	-0,028845559	0,332621146
RPL6	-0,028813106	0,681750806
NIT1	-0,028766481	0,58562319
LCMT1	-0,028720319	0,437795853
LRPPRC	-0,028706872	0,299458681
LOC442077	-0,028698457	0,369261808
SOX3	-0,028686222	0,671768005
OR4D10	-0,028570662	0,318554976
RGS6	-0,028566733	0,481979193
KLK5	-0,028547318	0,709882707
CCDC102A	-0,02852734	0,966134264
DENND1C	-0,028481526	0,635684639
ATRNL1	-0,028451925	0,725341647
ZNF272	-0,028432054	0,770465703
PLXNA1	-0,028380401	0,805107167
ZNF514	-0,028341452	0,622944861
ID4	-0,028241979	0,220991679
LOC648999	-0,028230265	0,952214201
ZAP128	-0,028179415	0,312075915
PCDHGC5	-0,028076777	0,650260628
TAS2R16	-0,028050314	0,448579044
SIGLECL1	-0,027988888	0,539432193
LOC729167	-0,027850024	0,520438889
SLC11A2	-0,027820641	0,722008838
TCF3	-0,027776853	0,455281564
C9orf51	-0,027624996	0,477300577
UPK2	-0,027618543	0,724839353
MTX2	-0,027567155	0,672369947
ZFP260	-0,027531883	0,729991462
PHLDB2	-0,027530246	0,401577562
ABCG2	-0,027506196	0,872617476
PHCA	-0,027476228	0,720979361
C16orf88	-0,027438679	0,584453845
DPP9	-0,027422341	0,367287097
AGXT2	-0,027394392	0,985130656
NPHP4	-0,027385782	0,857732314
TMEM65	-0,027364628	0,344375551
PRSS3	-0,027358108	0,489590723
MIST1	-0,027293573	0,845021053
FLJ21865	-0,027284399	0,608254547

FABP2	-0,027260556	0,887662606
TTLL12	-0,02725668	0,794696988
C10orf50	-0,027241138	0,823705008
LOC730188	-0,027191785	0,294042531
WDR27	-0,027178668	0,384715858
GJA1	-0,027065083	0,593345936
NKX6-2	-0,027015212	0,792351971
P2RY5	-0,027000613	0,500085953
ZFP37	-0,026991847	0,27182249
CMYA3	-0,026952162	0,903106442
CPLX3	-0,026915582	0,277366505
SECISBP2	-0,026849259	0,416407437
SYNJ2BP	-0,026815833	0,845013841
LOC652728	-0,026753091	0,419481976
OR51V1	-0,02672202	0,697470097
PHLPP	-0,026603446	0,535088201
ZNF396	-0,026569107	0,462627478
OR4N2	-0,026531882	0,768292563
LOC388381	-0,026480116	0,567466193
DOK3	-0,02644676	0,938360835
MFSD6L	-0,026355296	0,542393941
ACVR1B	-0,026334441	0,465810689
TRALPUSH	-0,026290639	0,61525833
COX4I1	-0,026257563	0,892227174
LOC158830	-0,026223451	0,404658253
GPR68	-0,026077251	0,267427046
TLR10	-0,025980542	0,614277416
LOXL1	-0,025909206	0,373878278
HCP5	-0,025808686	0,966835583
ZMYND15	-0,025803159	0,781470375
AKAP6	-0,025786657	0,779807769
PXN	-0,0257691	0,339147251
MAP7D2	-0,025721881	0,722982523
LOC732394	-0,025584937	0,702199524
STXBP6	-0,025453749	0,696407239
ISGF3G	-0,025383886	0,672732756
PGR	-0,025364515	0,742775867
NDST3	-0,02533313	0,638484872
TMEM85	-0,025212235	0,680243351
LOC728769	-0,025202623	0,293885926
CARS	-0,02518985	0,379704643
LOC730852	-0,02515832	0,23941364
FLJ37786	-0,025107036	0,788076631
SIM1	-0,025054152	0,38918404
NR2C1	-0,025049	0,988096392

LOC730512	-0,025012272	0,981607953
ZSWIM4	-0,025002968	0,455135275
PTRH1	-0,025000807	0,807939952
C13orf23	-0,024890532	0,384456319
LOC440895	-0,024858244	0,436441964
LOC729386	-0,024753496	0,234318766
POU2F2	-0,024652542	0,341805101
DPYSL5	-0,0246352	0,336123682
KISS1	-0,02461143	0,911458616
HPSE	-0,024600121	0,377748705
SMTN	-0,024570156	0,298855098
LOC729183	-0,024508603	0,329877982
FAM71B	-0,02436994	0,3265586
SGOL1	-0,024369627	0,5
MAPK8	-0,024340035	0,33350245
CLN6	-0,024336038	0,242090521
C18orf37	-0,024295443	0,715043399
AKR1C3	-0,02425749	0,843402919
TAF7L	-0,024205868	0,889188838
PSMA6	-0,024124498	0,574584694
ZNF583	-0,024122695	0,602704332
INSRR	-0,024052041	0,53058725
NRBP	-0,023930028	NA
PRKCBP1	-0,023736575	0,826654893
LOC729812	-0,023694126	0,903628989
ABAT	-0,023656651	0,909505711
hCG_20426	-0,023642367	0,720001712
HYAL3	-0,023619106	0,65010518
GPA33	-0,02346835	0,324276782
LOC645817	-0,0234408	0,427382216
C13orf28	-0,023350363	0,305545693
C1orf26	-0,02332754	0,53916526
PRRG4	-0,023327153	0,350816178
C15orf21	-0,023286425	0,914145056
DAPK1	-0,023265206	0,408961021
POLR1D	-0,023227903	0,744113136
B4GALT2	-0,023208131	0,485809443
TMEM38A	-0,023165067	0,23380078
C14orf106	-0,023147002	0,288233602
TRPC4AP	-0,023129058	0,968692876
SYT8	-0,023070326	0,996691166
NPM3	-0,022830302	0,740190383
hCG_1642425	-0,022827664	0,540345665
C1orf85	-0,022822261	0,360271572
CP	-0,022783331	0,497133377

FGFR1OP2	-0,022729461	0,908230881
LOC652832	-0,022595123	0,772309259
NCALD	-0,022496587	0,776516994
CCRL2	-0,022486278	0,444899514
LOC728754	-0,022482479	0,396433578
MMD	-0,022452568	0,290433717
DDX56	-0,022442145	0,197874703
LOC150935	-0,022377711	0,930846879
CD2	-0,022364279	0,988313337
KCTD11	-0,022294742	0,788598676
NBPF15	-0,02228101	0,991798906
LOC653712	-0,022248268	0,705267855
VPS18	-0,022124389	0,50001415
GAS2L3	-0,022118117	0,78044438
C18orf56	-0,022087168	0,632235711
FAM38B	-0,022062836	0,478802076
ARL6IP4	-0,021926087	0,672376023
TNNC2	-0,021920371	0,99378562
KCNK12	-0,021908327	0,215973359
FAM13C1	-0,021846635	0,945534622
C14orf156	-0,021844801	0,585527028
LOC645177	-0,021703753	0,290606953
IL9	-0,021692881	0,516549504
MREG	-0,021640505	0,180359798
USP1	-0,021634488	0,979262164
LOC642672	-0,021631064	0,581841169
DUSP16	-0,021623587	0,582554579
KRI1	-0,021619752	0,802898212
SMG1	-0,021618899	0,522507705
GNS	-0,021579004	0,37561436
C11ORF4	-0,021541053	0,353508713
ZNF547	-0,021427064	0,251414922
LOC644058	-0,021407845	0,650743165
PRKCH	-0,021339028	0,327564545
TET1	-0,021335682	0,853799182
LOC727809	-0,021270949	0,388398947
EYS	-0,021237458	0,319621546
ATP1A1	-0,021047913	0,691108705
EWSR1	-0,020977745	0,966764366
C16orf82	-0,02094977	0,453461327
TSC1	-0,020946467	0,376988871
CD52	-0,020917984	0,526582693
ATP5SL	-0,020862758	0,290223579
GABRA5	-0,020846688	0,320937657
TMEM177	-0,020842332	0,460985508

DTL	-0,020760245	0,748638213
MYH11	-0,020706103	0,316930609
ARHGAP25	-0,020640582	0,862063581
ANKRD13A	-0,020637847	0,534159382
LOC641994	-0,020580285	0,261486956
NOC2L	-0,020571929	0,578090004
MC1R	-0,02055028	0,929647605
FLJ22167	-0,02054095	0,550094918
LOC388814	-0,02048795	0,543903907
DPH1	-0,020486776	0,339109391
OR6C76	-0,020479951	0,96395269
CACNB2	-0,020476133	0,620078046
HMGXB4	-0,020455261	0,699772262
LOC732440	-0,020442346	0,631656047
EXOSC7	-0,020362492	0,423416886
LOC441009	-0,020309741	0,802813353
FLJ32255	-0,020257612	0,823919303
WBSCR27	-0,020236789	0,434485699
LOC728842	-0,020226701	0,95979934
NDUFS2	-0,02019242	0,576592602
PRRT3	-0,02017604	0,644872861
HSPC159	-0,020173377	0,27824416
FCER1G	-0,020072601	0,445411456
LILRA5	-0,019964918	0,690906392
PALMD	-0,019899296	0,476915969
FAM171A1	-0,019872784	0,239501576
PLAC1L	-0,01975623	0,509044342
PPM1H	-0,019736881	0,56205477
AS3MT	-0,01970311	0,697395627
FAM171B	-0,019663352	0,565851777
hCG_1646491	-0,019638639	0,839201622
C16orf73	-0,019634274	0,88932379
C11orf42	-0,019625482	0,487592369
TMEM133	-0,019592747	0,27734204
KLHL2	-0,019471339	0,659266312
DYRK1B	-0,019463994	0,943041055
HADHA	-0,019431951	0,929350267
FAM104A	-0,019409636	0,433876861
CYP4F11	-0,019389317	0,866436548
YLPM1	-0,019341254	0,238683301
MPDZ	-0,019306234	0,524864422
PDE1B	-0,019255439	0,731812315
DDX19	-0,019200955	0,79906584
LOC651789	-0,019158691	0,911573185
LOC729207	-0,019152157	0,803068159

LOC728171	-0,019124756	0,155106099
LOC727779	-0,019114757	0,888772042
SPIN2B	-0,019027046	0,444507701
OSBPL11	-0,018977318	0,660134408
DGKK	-0,018946429	0,844199673
SHE	-0,018897091	0,92620614
LOC729178	-0,018876458	0,78912068
LOC644632	-0,018841562	0,280344944
SLN	-0,01884047	0,9961462
IRX4	-0,018804186	0,403659001
GALNTL2	-0,01879406	0,395895954
ZSCAN1	-0,018737652	0,489845393
NHLH2	-0,018652739	0,740901301
CISH	-0,018638224	0,381398738
WDR25	-0,018573017	0,613374238
IL28B	-0,018513868	0,471942299
CEACAM4	-0,018479128	0,928577825
LOC731277	-0,018471342	0,461854402
ADAM18	-0,018470415	0,87763327
SYNCRIP	-0,018402417	0,316931807
LOC730338	-0,018400861	0,713193114
IL13RA1	-0,018370191	0,887147322
RAB8A	-0,018310508	0,295457148
EGLN3	-0,018257215	0,817109598
PMS2L1	-0,018246701	0,525280342
MCCC2	-0,018241629	0,505931086
EGR3	-0,018236814	0,982511098
ASCL3	-0,018078946	0,559796219
CLC	-0,018035495	0,311086952
HLXB9	-0,018012584	0,915425569
RPS15A	-0,017970849	0,245254111
RAB7L1	-0,017909604	0,511549453
LOC730136	-0,017843813	0,580243062
LOC389137	-0,017811414	0,849700673
NDOR1	-0,017809757	0,8706183
GPAA1	-0,01774809	0,948361618
GFRA2	-0,017728777	0,257661387
HARBI1	-0,017645805	0,840247279
LOC727745	-0,017580762	0,462575856
MAWBP	-0,017547416	0,262020065
LOC730121	-0,01751736	0,401133493
PIK3IP1	-0,017495587	0,369131555
SYTL1	-0,017475439	0,231702036
SASS6	-0,017397896	0,571655708
BAI3	-0,017369236	0,790135421

PRAMEF2	-0,017328298	0,807086906
ACPT	-0,017313218	0,907410404
GPHA2	-0,017290991	0,354237299
FLJ22795	-0,017266382	0,449671424
CCT2	-0,017222571	0,533249277
PABPC5	-0,017168373	0,250825858
ZNF341	-0,017145909	0,285624045
EMILIN3	-0,017068359	0,2209534
FCRH1	-0,017058867	0,36631848
SNX6	-0,017006947	0,281705125
R3HDM2	-0,016900171	0,57557965
FASTKD5	-0,016792489	0,987093655
ADCK1	-0,016737868	0,425542559
PCOLCE2	-0,016651371	0,652221727
STMN1	-0,016600504	0,416708183
AUH	-0,016593599	0,531927784
KCNC4	-0,016584861	0,683556763
LOC729888	-0,016477423	0,276142691
C13orf34	-0,016456324	0,907408602
ZNF596	-0,016442903	0,858971747
FRAS1	-0,016389256	0,767451527
STK22C	-0,016294767	0,861608975
HRB	-0,016276048	0,67844641
SLC24A1	-0,016245107	0,462874123
FAM117A	-0,01620118	0,71971055
SNIP	-0,016133115	0,250175843
DUX4	-0,01612731	0,387211103
PCDHA6	-0,016124416	0,496194539
MAP9	-0,01606537	0,539915213
LOC731440	-0,016001363	0,253730146
LOC644538	-0,015984095	0,446828966
FOXJ1	-0,015924119	0,550417383
FAM154A	-0,015853498	0,228060656
SLC38A5	-0,015770504	0,868240702
PIAS2	-0,015762178	0,991347201
ANGPTL2	-0,015730247	0,843456902
PTPN4	-0,015711227	0,44588738
LOC641858	-0,015574852	0,24252318
CPSF2	-0,015485745	0,850081426
SMARCA4	-0,01548391	0,815329406
ATIC	-0,015483062	0,932111858
LFNG	-0,015459561	0,621325255
ATG2B	-0,015428752	0,36939925
ELAVL1	-0,015351315	0,31913069
ASAH2	-0,015318859	0,380132914

SP6	-0,015265702	0,864649348
SALL4	-0,015219539	0,608396412
TBC1D2	-0,015149302	0,679278186
C11orf8	-0,015066419	0,573000905
PFN4	-0,015059156	0,96715112
HIST1H2AK	-0,01503469	0,902230892
NHEJ1	-0,014914523	0,214849387
UBIAD1	-0,014880151	0,346082928
DRD4	-0,014867908	0,454295515
BCL2L15	-0,014816902	0,445797688
TCEAL1	-0,014803357	0,986257857
MAST4	-0,014751861	0,833872317
TREM2	-0,014734544	0,289258059
C15orf12	-0,014718325	0,230807867
IQCK	-0,014706626	0,929656215
FERMT2	-0,014674817	0,55716385
FKBP3	-0,014517243	0,809313858
FAM170A	-0,014517018	0,723131687
PSG4	-0,014507574	0,804215225
HSPBAP1	-0,014272895	0,411035606
GPRASP2	-0,014203422	0,55503073
GTF3C2	-0,014187765	0,852644899
CCDC49	-0,014160653	0,282446167
ANKRD60	-0,014092433	0,899520754
DHX33	-0,013966904	0,984354694
C9orf66	-0,013928458	0,18347779
PRSS1	-0,013866578	0,627005983
TM4SF4	-0,013818489	0,373510708
SLC5A4	-0,013807575	0,938873268
TIMP4	-0,013774404	0,46238305
AK3L2	-0,013744727	0,365805413
CHML	-0,013732773	0,320917989
CTBP2	-0,013701401	0,986762836
RBM3	-0,013664526	0,532953501
NFYA	-0,013619457	0,420322838
C6orf106	-0,013575932	0,302707009
FBXL15	-0,013485708	0,774419821
LOC646557	-0,013469907	0,666146026
C3orf10	-0,013420806	0,695552943
LOC728808	-0,013418274	0,732439779
C1orf187	-0,013327073	0,735986385
MUL1	-0,013296997	0,217139679
BZW1	-0,013279781	0,72309123
LOC255374	-0,013261101	0,357463437
LTBP3	-0,01324177	0,855941481

SLC23A3	-0,013239437	0,886604137
FAM71A	-0,013177266	0,837855495
TYRP1	-0,013142042	0,45928748
OAS3	-0,013093733	0,437781023
PHLPPL	-0,013079089	0,968976711
CSMD1	-0,012999307	0,447559337
CCDC43	-0,01299702	0,793075404
VPS26	-0,012948197	0,881903108
EXOSC6	-0,012799733	0,458497609
LOC730197	-0,012788085	0,199338301
LOC727776	-0,012768179	0,359545855
F2RL1	-0,012748807	0,722198982
PUS7L	-0,012672922	0,799978218
PEF1	-0,012668296	0,455473203
LOC649294	-0,012650925	0,608170855
TFE3	-0,01253955	0,240522701
YIPF2	-0,01253759	0,715986949
LOC729810	-0,012405711	0,70057518
EPHB1	-0,01236475	0,490431102
ATP9B	-0,012315026	0,398777824
SLC7A5	-0,012264875	0,614514587
MSH3	-0,012230783	0,905133454
IL17REL	-0,012160189	0,666748395
ARHGAP1	-0,012158568	0,427534315
MAP3K11	-0,012045837	0,97625899
C3orf49	-0,012044863	0,559682076
KIAA1618	-0,011998562	0,988495493
PRDM10	-0,011980961	0,366042156
NOL9	-0,011977786	0,588359158
MBC2	-0,011937326	0,551926664
RALA	-0,011922511	0,790711928
SRP46	-0,011918684	0,525655459
LOC729968	-0,011917147	0,410001729
ZAN	-0,011912634	0,778405069
RASL10B	-0,011910982	0,277284878
IFLTD1	-0,011869222	0,582859665
C7orf2	-0,011832949	0,739020684
C8orf30A	-0,01180883	0,399991857
MLEC	-0,011755462	0,822099723
HSPC105	-0,011624825	0,741781208
NDUFA9	-0,011589706	0,481952378
FAM33A	-0,011530727	0,365014609
EXOSC5	-0,011519443	0,308838895
NGEF	-0,011480378	0,425383166
KLHL31	-0,011410929	0,279137129

SDCCAG10	-0,011401573	0,950378254
CCND1	-0,011384587	0,389193667
MR1	-0,011367973	0,738260202
MRP63	-0,011327692	0,756183251
HOXD10	-0,011292711	0,851265172
PCSK5	-0,01127527	0,638937771
GNA15	-0,01127138	0,563399229
DELGEF	-0,011186633	0,913465495
LOC653583	-0,01117369	0,392348938
CNIH	-0,011043639	0,286810501
ANKRD36B	-0,010956636	0,231077303
SFXN5	-0,010906148	0,696112804
C21orf70	-0,010796109	0,719202821
GHSR	-0,010717166	0,648277418
LOC730715	-0,010705984	0,969763775
LMAN1L	-0,010609758	0,680229036
C1orf114	-0,010609117	0,385042834
FLJ16518	-0,010582627	0,475296137
TMPRSS3	-0,010558691	0,54114171
C10orf71	-0,010553538	0,390924756
PLA2R1	-0,010485513	0,551322955
SRD5A1	-0,010477852	0,685376358
NTAN1	-0,010268964	0,655611393
LOC646170	-0,009958028	0,746238902
ALMS1	-0,00989515	0,522230343
PNLIPRP2	-0,0098791	0,602194574
LOC387654	-0,009842137	0,886122278
IPO9	-0,009824086	0,731690836
SEMA5A	-0,009801832	0,43977971
MRC1	-0,009795039	0,279756195
MIER3	-0,009728731	0,988251524
TCP10	-0,009722297	0,347053404
MAN2A2	-0,009658087	0,251332158
LOC731074	-0,009637026	0,439235614
CYP39A1	-0,009611355	0,443789885
TFAM	-0,009588921	0,286530341
C1orf35	-0,009584231	0,314830993
GALR1	-0,009571799	0,706876958
ZNF597	-0,009536473	0,695786175
C4orf35	-0,009473159	0,339071752
UBL7	-0,009464088	0,515192026
OMD	-0,009379745	0,456984282
METTL2	-0,009289564	0,511219986
RAPGEF5	-0,009274385	0,605823013
C4A	-0,009242508	0,312502793

LIX1	-0,00923061	0,442731862
C20orf75	-0,009188455	0,477750799
KRTAP2-4	-0,009100739	0,482544196
GNAS	-0,009031631	0,631070096
HMGCL	-0,008990385	0,47276313
USP46	-0,008873326	0,663175363
GLIPR1L1	-0,008873089	0,580811147
OR4K15	-0,008854881	0,334825211
TMEM207	-0,008852073	0,569320238
LOC554235	-0,008791941	0,420525972
KIAA1881	-0,00876737	0,210489283
LOC401286	-0,008763334	0,778758344
LOC646576	-0,008527089	0,412864081
CTAGE5	-0,0085158	0,783769537
TUBB5	-0,008485578	0,566132515
HNRPA0	-0,008466642	0,860768456
H2AFV	-0,008466419	0,982699774
HSP90Bf	-0,00840266	0,692321319
CAPN13	-0,008370084	0,368925182
TFR2	-0,008187971	0,958272499
FIGLA	-0,008183588	0,37231192
ABCF3	-0,008166845	0,825094163
DIO1	-0,008104335	0,852301671
LOC728852	-0,008102875	0,972124681
PSMF1	-0,007998345	0,832305932
LOC441377	-0,007998078	0,4277979
FLJ35220	-0,007985111	0,358666361
TNKS1BP1	-0,007952352	0,666835108
HBD	-0,007916099	0,758435967
ST7	-0,007908133	0,971394822
LOC731414	-0,007907411	0,429177895
OR52B4	-0,007905832	0,894531836
LOC441238	-0,00789304	0,501440036
LANCL2	-0,00768884	0,289422503
TMEM206	-0,007644029	0,81970645
SIGLEC6	-0,007621345	0,717072965
LOC645332	-0,007566993	0,952992932
LOC728236	-0,007554804	0,314904625
GGCX	-0,007544078	0,67141132
TMBIM1	-0,007471205	0,252470361
C5orf28	-0,007415513	0,220697315
GOLGA8E	-0,007404307	0,221898827
PSEN2	-0,007396236	0,248879137
RBPSUH	-0,007385421	0,544074297
UGP2	-0,007089533	0,371156025

LOC729991	-0,00707469	0,36446107
OR1K1	-0,007049259	0,275143024
DYNLT3	-0,007021917	0,413566676
VSNL1	-0,006972529	0,507891467
CEP170	-0,006950969	0,38297685
DICER1	-0,006896862	0,36400124
LOC643862	-0,006894323	0,647697608
TIMELESS	-0,006874679	0,343948399
ATP11A	-0,006865948	0,735220729
ZNF440	-0,006855172	0,796018484
SCDR9	-0,006845273	0,351950075
TGFBR2	-0,006683	0,572433318
C6orf118	-0,006659348	0,882626469
PDE1A	-0,006645424	0,407139568
TCF8	-0,006504976	0,445843246
OR4B1	-0,006475092	0,377915418
WDR24	-0,006458035	0,599589294
CCDC77	-0,006451788	0,826972313
OR52B6	-0,006448598	0,490355688
TLK1	-0,006387371	0,360865657
LOC284023	-0,006372048	0,234196203
RAN	-0,00633998	0,5
NOM1	-0,006315026	0,554461919
SAMD12	-0,006304045	0,524501502
USP47	-0,006291912	0,982719483
LOC727978	-0,006239001	0,565645808
ESRRA	-0,006219165	0,807264841
BTBD17	-0,006160392	0,365181981
GRAMD4	-0,006051279	0,275701267
FGF4	-0,006050877	0,285032893
MAST3	-0,006009925	0,45934792
LOC728940	-0,005980672	0,409290258
LRRC8B	-0,005979002	0,368076502
C1D	-0,005967628	0,49692514
NOTCH4	-0,005940196	0,419947603
SLC16A7	-0,005903535	0,250785343
ABL2	-0,005821575	0,390974405
ADAMTS10	-0,005819792	0,691153443
C20orf108	-0,005789341	0,589843085
C10orf97	-0,005750685	0,283723422
C10orf4	-0,005733535	0,985094902
EEF1E1	-0,005730306	0,256368331
hCG_1646420	-0,005721396	0,210968169
MMP8	-0,005581545	0,619167244
NRIP2	-0,005551277	0,685955458

C10orf57	-0,005548919	0,278300208
ATP2A3	-0,005517987	0,630355368
SPHK1	-0,005479815	0,421400432
KRBA1	-0,00545838	0,22645831
BMP15	-0,005443014	0,980473298
CNTNAP5	-0,005409212	0,871803718
C4orf26	-0,005402694	0,943670645
ARHGEF7	-0,005289696	0,489212203
DCTN4	-0,005216849	0,49965994
POLR1C	-0,005181204	0,408331422
AKR1CL2	-0,005159651	0,628901353
LOC646932	-0,005123931	0,926922471
GPR73	-0,005087528	0,421408109
HNRPA3	-0,005067954	0,392989854
SCYL1	-0,005050512	0,596428317
FLJ22763	-0,005029695	0,262942752
OPHN1	-0,004994702	0,818409417
NUDT9	-0,004960461	0,566103553
C3orf25	-0,004952034	0,498082074
GLRX2	-0,004903738	0,911782477
TRIM51	-0,004883626	0,946934669
LOC730291	-0,004861898	0,493653679
LOC732253	-0,004840242	0,345764416
KIAA1666	-0,004769277	0,663777744
THOC4	-0,004744727	0,542761036
MAGEF1	-0,004720183	0,255512914
NEFL	-0,004719987	0,585647343
MOBK1B	-0,004692017	0,578329608
DRD1IP	-0,004682375	0,442932126
IPMK	-0,004632577	0,755830575
LOC727732	-0,004624546	0,919861157
C1orf125	-0,004598692	0,848766759
LOC729628	-0,004471747	0,472316635
tcag7,350	-0,004419492	0,515202997
LOC730045	-0,004411394	0,230486687
HSD17B8	-0,004391185	0,752598213
LOC731941	-0,004286828	0,472668252
PRKCB1	-0,004279573	0,772001841
PPP1R7	-0,004261232	0,976170377
NKX1-1	-0,004199829	0,870116025
FLJ32332	-0,004130756	0,67086765
LOC257106	-0,004097153	0,901471371
GDF5	-0,004084508	0,199763346
TMIGD1	-0,004058944	0,700065176
MAP4K2	-0,003951663	0,420050389

FGFR2	-0,003944956	0,666736741
PFTK1	-0,003924634	0,386696637
SGEF	-0,003788327	0,886045636
MMP12	-0,003787581	0,330790884
PTPRS	-0,003779685	0,823601894
OSBP2	-0,003712633	0,478535284
LOC730163	-0,003693121	0,481718284
TTC21A	-0,003687363	0,357820099
LOC729251	-0,003652134	0,523134946
TGFB1	-0,003563449	0,855713693
GOLGA3	-0,003489458	0,468529756
AP3S1	-0,003467121	0,844459662
LOC729335	-0,003460215	0,756850399
EXOC6	-0,003445227	0,689149074
CRYGA	-0,00343245	0,571121676
CINP	-0,003333948	0,437840631
CYB5R2	-0,003284631	0,348711291
PCNP	-0,003157147	0,564355348
C17orf39	-0,003128547	0,48302547
FIGF	-0,003025438	0,990208005
ZHX1	-0,00299308	0,554976398
PRAM1	-0,002862525	0,995677733
LOC642861	-0,002818163	0,620086326
TMEM185A	-0,00276123	0,830933139
MGC16703	-0,002700847	0,352883262
KIF17	-0,002686565	0,95021602
MCOLN1	-0,002656519	0,971281362
KCNIP4	-0,002627239	0,438210348
SLC1A3	-0,002617262	0,824140475
LOC729508	-0,002501056	0,813825473
ITGAM	-0,002471738	0,746804575
RPRML	-0,002438147	0,22033133
C11orf59	-0,002420474	0,457792394
EIF5A	-0,002413684	0,698042754
LOC648262	-0,002326185	0,228372184
LOC642044	-0,002304025	0,193025902
PHACS	-0,002288571	0,744166041
MAGEE2	-0,002263191	0,649383483
LOC729770	-0,002112163	0,221444318
EPN1	-0,002083127	0,321784976
C20orf144	-0,002040541	0,786352886
NNT	-0,002031573	0,640031957
LARP5	-0,002022117	0,456143683
RPL32	-0,001964945	0,892546032
MAPKBP1	-0,001951425	0,250001076

NT5C3L	-0,001864549	0,487093702
GALM	-0,001832018	0,332540529
FA2H	-0,001791042	0,357807073
ILVBL	-0,001736241	0,883366968
MUC6	-0,001604061	0,482223843
TA-PP2C	-0,001599518	0,983158472
LOC644554	-0,001449048	0,431372491
LAG3	-0,001412057	0,816615365
ZNF648	-0,001332943	0,760857695
PRR12	-0,00125431	0,401845227
HYAL2	-0,001158201	0,941030113
BCDO2	-0,001148194	0,30833242
LOC647109	-0,001139853	0,894723597
RFPL1	-0,001120705	0,691514402
ELK3	-0,001038971	0,431900918
C19orf55	-0,001011748	0,938804483
LOC732093	-0,000905982	0,43926135
SPDYC	-0,000896116	0,454578619
TD1	-0,000867207	0,714037316
FLJ20481	-0,000862014	0,479763734
LOC646845	-0,000793319	0,491238121
SPC25	-0,000777941	0,514286385
CYP8B1	-0,000769125	0,816954345
PSME4	-0,000746945	0,592570896
TRAP1	-0,000701647	0,544388355
EBPL	-0,000669513	0,985394846
LOC730800	-0,000660922	0,303354425
IL4I1	-0,000615373	0,864778416
LOC732455	-0,000518549	0,589177653
CAPN3	-0,000479264	0,979825584
PRTG	-0,000470099	0,829040445
NR1D1	-0,000469018	0,995622127
GYS2	-0,000431316	0,38170124
RASA3	-0,000431138	0,505599826
SAA1	-0,000420453	0,549841931
PTGIR	-0,000419077	0,653797188
SPTA1	-0,000399071	0,613809817
SPC24	-0,000378365	0,698404692
C20orf43	-0,000267844	0,54369843
C20orf142	-0,000237688	0,728566701
NVL	-0,000221464	0,728270573
IBSP	-0,000195654	0,21837248
PLK2	-0,000187001	0,358030958
WHSC2	-0,000160519	0,87492922
h461	-0,00013694	0,884891248

ZPBP2	-0,000100008	0,655166983
PTPRC	-9,70E-05	0,866278888
AAK1	-6,15E-05	0,875695578
CYBA	-8,74E-06	0,251780273
ERV3	0	0,491666226
IFRD1	0	0,524760227
CSPG4	0	0,86313816
GYPE	0	0,231035887
BNIP3	0	0,836584687
CRISP1	0	0,254868892
DCX	0	0,425827151
DMP1	0	0,675630204
NEDD8	0	0,513597419
FBXW4	0	0,692411145
MAGEB1	0	0,228941271
NTF3	0	0,676646104
PCP4	0	0,565608372
PSD	0	0,463937331
PIP	0	0,999977712
PDGFB	0	0,503356983
PTCH1	0	0,272531172
TM4SF1	0	0,634769718
PLEK	0	0,991117788
PLP2	0	0,997004607
SEMG1	0	0,89362292
SURF1	0	0,177678591
TSPAN8	0	0,792726464
BCAS1	0	0,996271296
HIST1H2BM	0	0,935875312
TNFSF14	0	0,291101799
TCTA	0	0,225588201
C2orf3	0	0,864628177
TCN2	0	0,728928414
TAF4B	0	0,792127403
KIAA0408	0	0,48713511
JOSD1	0	0,524423531
KIAA0649	0	0,745146112
N4BP2L2	0	0,508643788
C14orf2	0	0,552949563
ATP5H	0	0,208734258
FGFBP1	0	0,540800715
REC8	0	0,284192638
CLEC10A	0	0,269049955
JMJD2A	0	0,791723364
SYCP2	0	0,539239877

LPIN1	0	0,347225948
DNMBP	0	0,41014403
KIN	0	0,970401381
IQSEC2	0	0,990853683
KIAA0090	0	0,861702313
CLCC1	0	0,415562595
SDCCAG3	0	0,762163747
NPC2	0	0,467826952
KIAA0564	0	0,439261955
AVL9	0	0,65883588
SLC35D1	0	0,823875114
SPIN1	0	0,788420679
SORBS1	0	0,793669324
ARMC8	0	0,777508581
BAT2D1	0	0,713065358
LEPROTL1	0	0,481432978
FAM162A	0	0,405955261
SYF2	0	0,424676265
CIZ1	0	0,972903663
TMEM158	0	0,821699034
UBQLN3	0	0,325341517
SMR3A	0	0,205004017
SCG3	0	0,665979642
CEND1	0	0,305406654
LACTB2	0	0,397640589
FAM134B	0	0,325178899
C9orf156	0	0,477766385
NUDT11	0	0,350210111
ALG13	0	0,602113754
USE1	0	0,737668204
IFT122	0	0,680536161
KLHL26	0	0,287919615
C20orf46	0	0,473361419
C5orf22	0	0,191550614
C20orf29	0	0,586205407
NPLOC4	0	0,480208936
UBAP2	0	0,708693551
CENPJ	0	0,910790037
DENND1A	0	0,566626161
SERTAD4	0	0,595361341
C9orf102	0	0,086882702
KIAA0492	0	0,780722082
KIAA1609	0	0,669585463
NDUFA4L2	0	0,360805911
EXOC2	0	0,345982519

KIAA1219	0	0,4829868
GSDMC	0	0,665083515
C16orf58	0	0,423463029
MRPL9	0	0,474956463
GPBP1	0	0,533313098
RAPH1	0	0,891126058
TMEM43	0	0,407227702
GPATCH3	0	0,394649955
MRPL17	0	0,61780381
MRPL24	0	0,390097985
TMEM108	0	0,423152034
IIP45	0	0,335686722
CCDC71	0	0,966758493
C7orf23	0	0,37337213
FAM111A	0	0,296190571
PHF23	0	0,449305296
TRABD	0	0,829484961
PRR3	0	0,541267994
FAM49A	0	0,227267984
THAP9	0	0,402885729
C7orf50	0	0,514460972
TMEM101	0	0,355955191
FLJ13305	0	0,907958117
MFSD7	0	0,713382259
TMEM107	0	0,580488093
IQCG	0	0,428431945
CCDC3	0	0,55229417
DTNBP1	0	0,260812075
HOPX	0	0,19855005
FAM113B	0	0,72226215
CIRH1A	0	0,27659534
LMBRD2	0	0,251706301
CEACAM21	0	0,682806527
TIFA	0	0,215584447
LRP11	0	0,837691899
FAM40A	0	0,367185093
CCDC97	0	0,913310144
FAM181A	0	0,70125428
LOC91664	0	0,620092446
COL27A1	0	0,789238377
ORAI1	0	0,480054525
FAM46B	0	0,313198855
MRFAP1	0	0,473842139
GYLTL1B	0	0,388363565
C17orf64	0	0,401596262

MAPK1IP1L	0	0,413158645
SCAND3	0	0,39608218
ANKRD9	0	0,453623844
FBXO17	0	0,233070483
VPS37A	0	0,526217648
LRRC38	0	0,926104899
OR6N1	0	0,722255409
C2orf50	0	0,610042925
LOC127406	0	0,85560273
C5orf37	0	0,547618924
MESP2	0	0,769898014
SMCR8	0	0,291545476
FLJ30428	0	0,507556236
C10orf46	0	0,991644536
ASB15	0	0,667194338
OR52I2	0	0,378863513
CBLN4	0	0,638929072
DNHD1	0	0,401336543
PRUNE2	0	0,771448193
CSAG1	0	0,513438683
CCDC60	0	0,473042977
RBM46	0	0,365653759
C12orf12	0	0,673264827
DENND5B	0	0,67115524
PEBP4	0	0,658378537
OR4S1	0	0,751721622
DEFB105A	0	0,960446417
OR5M11	0	0,588731797
C19orf26	0	0,920820213
LASS3	0	0,842624403
TMEM130	0	0,655536637
TMEM188	0	0,892822464
FERD3L	0	0,433089408
LYSMD2	0	0,221368126
MDGA1	0	0,535167671
LOC284064	0	0,875984911
DOK7	0	0,742235087
LOC283398	0	0,672963491
KLHL35	0	0,64773203
C3orf46	0	0,573506064
C19orf30	0	0,538463609
CXorf59	0	0,48456383
OR1J1	0	0,383965907
TMEM173	0	0,367362562
LOC342979	0	0,76856851

LOC389174	0	0,288139803
LOC387876	0	0,283061495
LOC389043	0	0,291186935
C20orf107	0	0,844341151
LOC387693	0	0,924144843
OR5W2	0	0,930746148
LOC388813	0	0,591078314
LOC388344	0	0,527366466
LOC392563	0	0,793046951
ELFN1	0	0,357621927
PRAMEF12	0	0,864097393
FLJ26245	0	0,339308108
LOC391037	0	0,431422904
LOC400756	0	0,910198463
FIGNL2	0	0,260255872
C10orf85	0	0,96334045
CTXN1	0	0,224065025
FLJ41733	0	0,698603908
LOC390829	0	0,506204075
FLJ45537	0	0,613233372
C11orf38	0	0,710916926
PRAMEF3	0	0,574838158
LOC391539	0	0,543577485
FLJ41047	0	0,665538821
GOLGA8B	0	0,971444137
FLJ43692	0	0,468538935
LOC641931	0	0,271821588
OR4Q3	0	0,789438441
LOC541469	0	0,602889742
LOC441868	0	0,796821459
LOC642103	0	0,744260768
LOC643260	0	0,228329709
LOC642872	0	0,227563783
hCG_1787519	0	0,679491407
KIAA0754	0	0,985435065
LOC643153	0	0,191034295
LOC644992	0	0,701812543
LOC644215	0	0,930636414
LOC646670	0	0,422649731
LOC645776	0	0,979470431
LOC645553	0	0,374347047
LOC652612	0	0,231453804
LOC650995	0	0,966512605
LOC649136	0	0,480884057
LOC652722	0	0,829313634

LOC651986	0	0,568043316
LOC727817	0	0,519171263
LOC727927	0	0,347972508
LOC654106	0	0,933771081
LOC728449	0	0,830119173
LOC728193	0	0,218938933
LOC728178	0	0,389138045
LOC728055	0	0,944285663
LOC728622	0	0,33175067
LOC728711	0	0,722891754
LOC728962	0	0,446078803
LOC729108	0	0,565481429
SLC15A5	0	0,41152342
LOC728649	0	0,972823303
LOC729165	0	0,981473379
LOC729147	0	0,347869121
LOC729012	0	0,672210289
LOC729315	0	0,792605471
LOC729030	0	0,317676054
LOC728704	0	0,422649731
LOC728826	0	0,978893704
LOC729017	0	0,371613553
LOC729173	0	0,228890721
LOC729847	0	0,56522694
LOC729542	0	0,267579425
LOC729865	0	0,86795095
LOC730020	0	0,364919476
LOC729398	0	0,202551234
LOC730137	0	0,911002064
LOC730834	0	0,510454068
LOC731354	0	0,38572036
LOC730189	0	0,556096918
LOC731136	0	0,245785287
LOC731419	0	0,456606157
LOC730765	0	0,372366052
LOC732445	0	0,673803758
LOC400236	0	0,894606724
LOC285095	0	0,734967386
FAM107B	0	0,580577126
LOC732141	0	0,601834818
HIST1H2BD	0	0,516653103
MAGOH	0	0,474500529
TFF2	0	0,59215269
COBLL1	0	0,558293948
ANAPC13	0	0,637871555

NBPF14	0	0,643631137
C16orf80	0	0,880124468
MRPL15	0	0,36817293
PUS7	0	0,805546469
PEX26	0	0,397393779
CENPL	0	0,220360631
CYTSB	0	0,870914462
C1orf59	0	0,425489401
DCST2	0	0,459687513
CCDC110	0	0,36375115
HIST1H2BA	0	0,402952357
DEFB113	0	0,865493716
C1orf175	0	0,83864537
LOC344709	0	0,718131536
LOC339879	0	0,95528256
LOC389257	0	0,280510339
OR2B3P	0	0,32558487
LOC641827	0	0,540520107
LOC644934	0	0,497402544
hCG_1660379	0	0,326656819
LOC647074	0	0,846122818
LOC729609	0	0,700880366
LOC730807	0	0,44595584
SH3BGR	0	0,925698286
MRPL10	0	0,767321739
LOC653232	0	0,721742718
NEDD1	0	NA
LOC374395	0	NA
LOC440055	0	NA
INCENP	0	0,098523724
CDK9	0	0,773341301
EPHB3	0	0,581219936
DGKG	0	0,866154323
ITPKA	0	0,94604143
FLJ10074	0	0,326752942
CNKSR1	0	0,849975304
HCV_321	0	0,163881593
MKNK1	0	0,347623353
ADRBK2	0	0,940624927
HIPK4	0	0,528182849
CD81	0	0,046853665
IRAK4	0	0,771388623
CLK1	0	0,589829357
CAND2	0	0,741314729
ELAVL3	0	0,44111448

EIF4EL3	0	0,605907993
AIRE	0	0,692400164
ALS2CR19	0	0,733314868
LAMB3	0	0,581199343
MKRN3	0	0,319864859
MICB	0	0,284747433
PAFAH2	0	0,546959884
PARD3	0	0,854741912
LOC342096	0	0,775996747
TIEG	0	0,394748331
PCDHB15	0	0,554510412
KLF5	0	0,557047405
PRKCABP	0	0,495160706
CYSLTR1	0	0,649344461
ROS1	0	0,788425192
SRPK2	0	0,97005052
CCR3	0	0,815673198
PRKCD	0	0,362540137
BDKRB1	0	0,432680472
ADRB3	0	0,302054167
OPN1SW	0	0,607781881
GPR85	0	0,246511571
SDCCAG33	0	0,44258739
ADAMTS17	0	0,404342465
HRH2	0	0,359508383
ADAM22	0	0,72866885
NPY1R	0	0,261018905
PPARG	0	0,910626654
TRAR1	0	0,400782145
GPR25	0	0,429513198
GPR135	0	0,986231255
MRGX2	0	0,671993382
RORA	0	0,27000067
ZNF501	0	0,709312461
STAMBP	0	0,740601148
OIP106	0	0,334373689
ZNF41	0	0,681099626
RNF12	0	0,242494824
ZNF124	0	0,259726238
XPNPEP1	0	0,803412934
XPNPEP2	0	0,593366681
YME1L1	0	0,512784643
ZNF79	0	0,744914778
TMPRSS2	0	0,747086235
USP5	0	0,270934472

DKFZP572C163	0	0,869033712
ELA3B	0	0,301029793
ITPR2	0	0,794245081
MMP10	0	0,914931022
CTSH	0	0,813936051
PSMB1	0	0,558330643
GGH	0	0,939856476
KCNG3	0	0,560891886
SLCO1B1	0	0,26057452
ABCA7	0	0,633714319
SLC2A11	0	0,360855874
SLC24A3	0	0,967897999
GLRA2	0	0,385093119
ALOX15	0	0,786144352
SLC7A2	0	0,246111201
SLC6A7	0	0,358700681
PPP1R12C	0	0,26876383
DHRS1	0	0,476177537
GPR21	0	0,430410319
HSD17B3	0	0,609598321
IL12RB2	0	0,675609478
CHDH	0	0,557187489
IL1R2	0	0,737546413
IL10RB	0	0,343391846
MCTS1	0	0,414737015
C22orf20	0	0,827995797
KIR3DL2	0	0,276420365
CD1E	0	0,973091403
GRHPR	0	0,781998465
GPX2	0	0,473039022
MAP4K3	0	0,816788206
ADH1B	0	0,310838398
RDH13	0	0,610634151
MOGAT2	0	0,641057589
MAT1A	0	0,830537011
HADH2	0	0,902012643
MAP3K14	0	0,988557375
MGST2	0	0,668177828
TGM4	0	0,803565034
RGS5	0	0,451209362
RGS17	0	0,581358099
PNPO	0	0,803381374
UGT2A1	0	0,71224304
GSTA1	0	0,629118103
GPSM2	0	0,437486205

GNG13	0	0,703027851
HBXAP	0	0,453264874
RGS4	0	0,352187734
THEA	0	0,298123407
ICMT	0	0,830387049
FLJ10326	0	0,349194786
WHSC1	0	0,243114628
SELI	0	0,23578313
ARSD	0	0,687696291
ARHGAP5	0	0,857871808
SMUG1	0	0,491647056
PLA2G4B	0	0,985590958
HAGHL	0	0,334395685
SLC25A16	0	0,447609353
ZNF385	0	0,445934334
FABP6	0	0,446021537
RBP2	0	0,47362227
CACH-1	0	0,584645888
EPAS1	0	0,795081118
NONO	0	0,386381471
GLS	0	0,690580559
NKX2-3	0	0,542775715
BLM	0	0,354287206
MCM8	0	0,348706182
ETV6	0	0,925598453
CDX2	0	0,389026165
RABIF	0	0,480670814
XAB1	0	0,313516207
HDAC3	0	0,841274103
SMPD2	0	0,527818322
CHERP	0	0,538106413
HEAB	0	0,614969543
HNRPUL2	0	0,777152324
PPARBP	0	0,26509309
CHD5	0	0,675868988
CHD7	0	0,429493134
LOC200895	0	0,881522552
PHF11	0	0,190247975
RAD54L	0	0,357358862
PPP1R15A	0	0,322744901
HNF4A	0	0,469120702
RBM8A	0	0,552023392
RAB3C	0	0,656037318
NISCH	0	0,567573854
FLJ32978	0	0,210486483

PARC	0	0,296270604
CDX1	0	0,936016569
CTNNB1	0	0,637584828
NIPA2	0	0,372783199
NKX6-1	0	0,747041367
SCLY	0	0,605567591
ICEBERG	0	0,929578848
TLN1	0	0,851341445
TARDBP	0	0,735675369
MYO18A	0	0,945735348
USH1C	0	0,783790797
RORB	0	0,444704317
KRTAP5-1	0	0,889666395
AKAP1	0	0,44569515
DNAI2	0	0,954566328
ZNF219	0	0,781967044
SPIC	0	0,413643014
SOX4	0	0,407452256
MAX	0	0,202131845
FBXO11	0	0,656979889
NEURL	0	0,794479142
TEKT2	0	0,635211345
HOXB13	0	0,912398102
RAC2	0	0,271504301
CD151	0	0,307755771
RAB15	0	0,298176322
RRBP1	0	0,471799233
MGC33211	0	0,900893329
EDA	0	0,209412872
Empty Well	0	0,11996788
JUP	0	0,69402499
LILRB5	0	0,383294447
TRIM52	0	0,660706957
RAET1E	0	0,423930395
CSNK1E	0	0,427166247
CABP4	0	0,538984307
PCDHB5	0	0,333977084
NRF1	0	0,67653897
TAO1	0	0,738673446
OVOL1	0	0,819460974
CAPN10	0	0,180376618
MGC4171	0	0,724596636
ZNF450	0	0,507535465
FLJ45880	0	0,300562674
USP13	0	0,540676945

PHGDHL1	0	0,48433108
CLIC1	0	0,582381744
WWOX	0	0,409315464
SLC30A7	0	0,725470582
SIGIRR	0	0,467949146
PTPN11	0	0,963551989
LTBR	0	0,715638187
MGMT	0	0,194915726
RGS10	0	0,471241804
ABHD7	0	0,485304571
PLA1A	0	0,715109648
CCRN4L	0	0,897936049
GANAB	0	0,195862126
ARHGAP21	0	0,351189663
PSMD2	0	0,435224646
PRKRIR	0	0,346285381
MAPK8IP3	0	0,465299037
PITPNM1	0	0,863701991
PYCARD	0	0,963256973
BIRC6	0	0,70198841
LOC440515	0	0,885158248
ZNF292	0	0,265966181
CDC37	0	0,284361335
F12	0	0,559941124
LOC729514	4,89E-05	0,918563429
MRPS23	0,000117339	0,341712762
DSCC1	0,00013692	0,293263897
RPL39L	0,000184671	0,386348849
PTPRB	0,000294332	0,938614763
LOC644695	0,00032036	0,645434456
FLJ45256	0,000384335	0,496300876
PPP1R14C	0,000429381	0,805654891
HPN	0,000431007	0,446186768
SLC15A1	0,000443917	0,883279122
KLRB1	0,000460679	0,291439645
MKL1	0,000547493	0,962643745
LOC647264	0,000612195	0,700836303
C2orf39	0,00062005	0,539571746
TCEB2	0,000701647	0,313883376
GAN	0,000798728	0,579003125
C1orf200	0,000834351	0,233629917
LOC728558	0,000873824	0,840053701
ZNF157	0,000879287	0,731782863
HADHSC	0,001022661	0,95478943
FMN1	0,001141801	0,213641971

LOC730336	0,001161612	0,840231176
LOC729410	0,001182111	0,864272687
NEUROD1	0,001190362	0,523305868
FLJ20309	0,001292345	0,337854307
PTTG1	0,001304805	0,933243345
CCDC84	0,001313049	0,903095306
HSD11B2	0,001341074	0,478231239
ARHN	0,001445648	0,422277065
LOC138652	0,001520813	0,570232926
LOC136288	0,001538126	0,563782718
ACAN	0,001731394	0,545813429
LOC391766	0,001811965	0,818679606
LOC650157	0,001841281	0,782515953
BARX2	0,001916366	0,863326836
ETV3	0,001920373	0,957965235
IRAK1	0,001972478	0,456743552
WIPF2	0,001995057	0,89873181
PCDHGB6	0,002022117	0,355201846
C17orf91	0,002036822	0,275165503
LOC729479	0,002045463	0,739815452
TMEM181	0,002054466	0,531852552
C16orf59	0,002124782	0,389849057
LOC730597	0,002125515	0,229369453
CBX3	0,002173217	0,673054726
C1RL	0,002176482	0,994959267
CDC5L	0,00219321	0,994776745
FTHL17	0,002205241	0,898310889
GTF3C6	0,002350009	0,620469597
DKFZp586M1819	0,00237343	0,383986898
TNP1	0,002418367	0,274547664
TNFRSF10B	0,00246549	0,546072825
DKFZP434P211	0,002470563	0,544445087
LOC728469	0,002539784	0,579642199
SCN1A	0,002554114	0,585546445
OR1E1	0,002577718	0,497508282
NMUR2	0,002607917	0,265078186
OR6F1	0,002684442	0,960679442
B3GAT3	0,002686022	0,804368295
KBTBD6	0,00276123	0,441636868
MT1IP	0,002784039	0,243410596
LOC730212	0,002924296	0,423071327
RRS1	0,002928168	0,97842604
FIT1	0,002972873	0,438901708
LOC652725	0,002973333	0,898445886
APG4C	0,003016241	0,457891976

LRRC8A	0,003039468	0,917170172
LOC644021	0,003222537	0,28457409
BIN3	0,003244464	0,227938651
NEK4	0,003282105	0,469869289
CMRF-35H	0,003289896	0,405196509
PVRL4	0,00330573	0,6841444
KIAA0232	0,003351966	0,507964065
INO80	0,003413951	0,516631733
DUSP10	0,003415531	0,715065348
PIGL	0,003449607	0,380737213
VTN	0,003472481	0,240079882
ZNF609	0,003480457	0,551080225
CECR1	0,003530135	0,469946629
LOC728228	0,003534748	0,991762869
C20orf39	0,003588847	0,697885681
TTPAL	0,003644803	0,238115234
LOC644186	0,003669111	0,227805972
ANGPTL3	0,003822566	0,380400522
USP42	0,0038408	0,589256571
PLEKHA8	0,003882646	0,401907289
GREM2	0,003893874	0,504884923
PTPRA	0,003901353	0,45170219
ERMAP	0,003930452	0,648060517
ZBTB26	0,003961713	0,864840619
TNFRSF21	0,003964515	0,441210984
OR1L8	0,004017545	0,286632177
SLC16A6	0,004060839	0,921188499
OR8K1	0,004231228	0,489510141
LOC641826	0,004292287	0,304998785
RBP7	0,004293585	0,878780531
NPEPL1	0,004365913	0,277895691
MGC45438	0,004373722	0,436453643
ARFGEF2	0,004406554	0,424402871
EPN2	0,00440897	0,882318774
KEAP1	0,004427055	0,224287331
LOC391258	0,004433745	0,459739286
A1BG	0,004743336	0,783836312
C1orf25	0,004750302	0,40975445
LOC285556	0,004760559	0,887527881
PLEKHG7	0,004789271	0,888062499
FUCA2	0,004804182	0,34744108
FLJ41603	0,004807947	0,418419971
ZNF846	0,004820492	0,934456634
MGC13057	0,00482623	0,427500422
FNDC7	0,004826441	0,195692619

PPL	0,004827117	0,510068193
GPX4	0,004827147	0,544156007
C10orf47	0,00490895	0,527406149
MEA1	0,004926418	0,965772926
RPS15	0,004995602	0,451054981
KIAA2026	0,005003649	0,51977352
IL15RA	0,005016723	0,439125451
THRSP	0,005180863	0,785616103
LOC728799	0,005323619	0,288663953
FOXP2	0,005326082	0,670491296
LATS2	0,005336216	0,388472588
TMPRSS4	0,005400609	0,623284582
KLF2	0,005426616	0,431468349
KCNJ9	0,005430686	0,383194642
EDNRA	0,00543798	0,499455734
RNF157	0,005448882	0,308237701
LOC730005	0,005479643	0,412837725
B3GAT2	0,005563781	0,834491862
TMF1	0,005564893	0,400600667
LOC650656	0,0055747	0,480194938
DPYSL4	0,005621505	0,919284711
SMTNL1	0,005691345	0,368094286
FCHSD1	0,005798292	0,435474658
LOC387941	0,005867359	0,658647656
SIGLEC11	0,005940196	0,361747333
C2orf29	0,00597814	0,21435421
C12orf30	0,005988893	0,469422046
C12orf57	0,0059929	0,504515011
NF2	0,006020442	0,475104263
MASS1	0,006081227	0,962043522
OSGIN2	0,006105124	0,305714804
PRND	0,006171505	0,356166199
HLA-DQA1	0,006181401	0,680414637
LOC729576	0,006230845	0,852776246
VNN1	0,006263094	0,909799233
TEAD4	0,006307645	0,96998589
RGL2	0,006315026	0,581521702
PDPN	0,006332092	0,797347634
SFRP2	0,006430042	0,970308224
MGC119295	0,006446388	0,44202229
NUDT16L1	0,006458035	0,504964988
SNAPC4	0,006460156	0,447469169
WIPF3	0,006487866	0,568927196
NLGN1	0,006543854	0,757070599
C6orf103	0,006566604	0,803445425

PRAF2	0,006595543	0,720537635
TAS2R39	0,006603371	0,324898198
DYRK2	0,006653943	0,541586582
AFAR3	0,006671422	0,977968634
TBL1X	0,006714248	0,540977854
LOC643678	0,006749942	0,248422331
C5orf39	0,00678199	0,736296277
ULK2	0,006789574	0,301787817
LOC285679	0,006871159	0,653830071
XYLT1	0,00688144	0,917002572
ZYX	0,006884581	0,810559048
EN1	0,00689387	0,606444222
CRMP1	0,006896862	0,457912407
UBE3B	0,006945481	0,273314877
EZH1	0,00706557	0,823331882
THYN1	0,007073244	0,486431853
LOC730669	0,00707469	0,836530519
LOC647163	0,0070865	0,966899324
FAM163A	0,007092191	0,346705668
FAM187B	0,007102952	0,979682861
PTK9L	0,007172645	0,78485461
RET	0,0072253	0,867916468
LOC387939	0,007283838	0,433069726
EPOR	0,007291847	0,897958212
KCNH1	0,007353337	0,379969964
C2orf40	0,00735471	0,323840257
SELENBP1	0,007406892	0,867290725
HTR3D	0,007437651	0,582164512
HSBP1	0,007442354	0,843088889
KRT6C	0,007506252	0,37014017
KRTAP2-2	0,007517241	0,345648015
APOF	0,007543011	0,882021074
CYHR1	0,007566723	0,741967121
OAZIN	0,007576828	0,441571681
FLJ23311	0,007593866	0,846319911
C10orf114	0,007595313	0,748717153
AKR7A3	0,007610081	0,475972392
FLJ12592	0,007614532	0,771587644
CDH2	0,007619326	0,562587763
LOC645269	0,007642034	0,789436169
KLF13	0,007642202	0,93422253
SLC6A5	0,007663073	0,747325111
DKFZp761E198	0,00769784	0,405964353
ENO3	0,007737556	0,355521653
LOC650515	0,007750033	0,345463883

PTPN5	0,007780933	0,192411204
CT45-3	0,007826511	0,261845064
CGI-26	0,007836794	0,498882806
ADRBK1	0,007839827	0,672629857
PRSS35	0,007856399	0,204838151
CXorf26	0,007971507	0,536241628
MLXIP	0,008044688	0,882619637
CAST	0,008052999	0,573988176
TJP3	0,008074625	0,837012251
DISP2	0,008098114	0,830506046
FAM92B	0,00815615	0,185430589
HDGFL1	0,008166494	0,333841472
MIA2	0,008169738	0,761125171
WDR63	0,00817039	0,794563392
ANAPC4	0,008180884	0,387648899
LOC401097	0,008239902	0,83062644
ATP5G3	0,008242963	0,480413771
C17orf82	0,00824842	0,239179659
LOC643770	0,008305364	0,470700211
JARID1B	0,008408706	0,500500803
NDUFC1	0,008427008	0,247722565
ARHGAP19	0,008449152	0,691400159
LOC728281	0,008479165	0,360254814
SLIT1	0,008539301	0,363571912
FLJ10759	0,008552238	0,649581378
ABLIM1	0,008557506	0,712278634
LOC389118	0,008564618	0,975507927
HIST4H4	0,008718024	0,228166761
C6orf186	0,008737309	0,459154782
STYX	0,008764417	0,601994118
PDCD5	0,008783658	0,88499212
CYP7B1	0,008824055	0,595405125
RPRM	0,008916034	0,360631772
LOC401898	0,008935861	0,257949008
SRRM1	0,009016957	0,35305944
SMARCC1	0,009035307	0,528554652
COX10	0,009052506	0,361141512
PTPRT	0,009053429	0,410883749
INPP5D	0,009139254	0,98818754
C12orf63	0,009221874	0,487702578
FUT3	0,009225121	0,825236313
ZFYVE16	0,009226736	0,602957085
NOL8	0,009243166	0,817437377
LOC730282	0,009250672	0,323039939
EXOSC9	0,009262012	0,258039387

BTNL8	0,009264294	0,362066156
ORAI2	0,009287032	0,738251252
CNO	0,009373991	0,534791092
ZFP28	0,009389425	0,2946497
GTPBP1	0,009445558	0,778863061
CCDC136	0,00944681	0,606664175
LOC729715	0,009576078	0,881668997
LOC728795	0,009633461	0,469956861
ERRFI1	0,009648806	0,418841522
LOC728647	0,009659831	0,808120978
FBXO5	0,009678168	0,4322782
ZCCHC14	0,009686323	0,286567652
C2orf56	0,009706338	0,443104845
LOC645949	0,009710527	0,448079391
CABC1	0,009732314	0,632087585
HXMA	0,009770148	0,791415439
HMFN0672	0,009817315	0,524983351
KLF14	0,009833975	0,284063835
OR10G8	0,00989263	0,311821898
EPS8	0,009947645	0,443459565
C2orf15	0,010056677	0,943855804
TUBAL3	0,010067659	0,666359234
ADAMTSL3	0,010096478	0,528643472
C1orf74	0,010322568	0,365707827
IFNA7	0,010333933	0,27627599
RBM	0,010457438	0,860712934
PTK2	0,010545602	0,531398461
NPAS1	0,010548625	0,456421149
GDF9	0,010614851	0,738925201
KRTAP3-3	0,010700824	0,60397983
PC-LKC	0,010731582	0,311289306
FGF19	0,010820233	0,42908493
HSF2	0,010853181	0,86655877
THAP7	0,01086818	0,888982089
TATDN1	0,011007669	0,566463556
EBAG9	0,011017513	0,812179866
RNF2	0,011062194	0,868065561
PBX2	0,011101715	0,696810283
ZSWIM5	0,011134975	0,510644557
DGAT2	0,01116069	0,258746809
LOC440338	0,011196483	0,289282652
TNKS	0,011230501	0,688244556
RLBP1L1	0,011389785	0,699346275
RTTN	0,011414352	0,254733505
RGMA	0,011427574	0,194983654

LOC732438	0,011443391	0,196154525
BCKDK	0,011456211	0,5
FAM133A	0,011466042	0,312780745
BTF3L4P	0,011486895	0,255716244
LOC730809	0,011538286	0,317982425
IL32	0,011543503	0,763465488
SEC22C	0,011564967	0,325443115
C17orf69	0,011570328	0,945608491
FOXJ2	0,011582537	0,662928965
RAB5C	0,011606404	0,797426433
VPREB3	0,011679167	0,510940191
DNAH7	0,011707895	0,784391673
CEBPB	0,011716869	0,442301402
ARNT2	0,011801729	0,847732348
SHANK2	0,011804055	0,548053368
RFX5	0,011815579	0,86297081
LOC651536	0,011860572	0,406397394
hCG_2001000	0,011863268	0,241332987
KDELR2	0,011899768	0,72361863
C4orf18	0,011916485	0,830469187
MOCS3	0,011969229	0,723143055
SACS	0,012160912	0,954780914
SLC6A8	0,012264875	0,699385933
LOC729367	0,012281732	0,854304787
ABCA10	0,012290531	0,964518794
LOC729549	0,012291235	0,311427102
GZMM	0,012312632	0,731655049
C3orf27	0,012352913	0,305363861
LOC729076	0,01249628	0,244066179
LOC285941	0,012538215	0,789918565
CTRB1	0,012540172	0,825477902
CHPF	0,012620453	0,934944344
NKD1	0,012646425	0,388029335
TRO	0,012661108	0,589166315
PDE6C	0,01268241	0,552519174
FAM107A	0,01269918	0,999644917
SPTBN1	0,012714982	0,383667462
C1QR1	0,012759173	0,233222393
BET1L	0,012778781	0,281240272
PRPF3	0,012839872	0,38768484
SCN9A	0,012851726	0,497400337
SH3RF2	0,012857249	0,490478383
FKSG44	0,012877261	0,891024122
FAM108B1	0,012947888	0,553232941
LOC730096	0,0130483	0,299669623

PKN1	0,013048824	0,663554163
LOC729974	0,01311039	0,461639045
KCNG4	0,013126216	0,660665703
FGF7	0,013249105	0,815175955
PRKAA1	0,013276181	0,94178191
HSD17B2	0,013384746	0,200919584
UBXD7	0,013412483	0,271483632
OR2AK2	0,013440896	0,768203773
CC2D1B	0,013491108	0,69186509
ALDRL6	0,013547123	0,396950298
TMEM190	0,013598899	0,340586264
PIH1D1	0,01364719	0,635448718
TXNDC7	0,013697196	0,475224896
NDUFS1	0,01374475	0,76903973
PDHA1	0,013776604	0,911945069
LOC645490	0,013818838	0,442849782
RTN4RL2	0,013908877	0,337500445
OPRK1	0,013930179	0,964263453
GMPR2	0,013965482	0,747655435
PGLS	0,013977089	0,443140063
C2orf42	0,014005628	0,634900421
FSIP1	0,014093633	0,383041286
B4GALT3	0,01412436	0,520618347
LZIC	0,014131821	0,307040474
MTP18	0,014160653	0,249599965
MPRP-1	0,014172354	0,492172479
DLEC1	0,014176801	0,616489666
LOC650780	0,014309954	0,588344559
LOC441490	0,014324875	0,258166089
SERF1B	0,014416986	0,614513825
CDH8	0,014421918	0,544176474
7A5	0,014436423	0,72353303
YRDC	0,01446987	0,46076121
TMEM143	0,014479677	0,747313068
COMMD3	0,014495318	0,877109823
C10orf91	0,014503423	0,455547185
PSMG1	0,014602269	0,242149488
CHRNB1	0,014644129	0,467358978
LOC729951	0,014666877	0,484386351
LOC727811	0,014687772	0,384872236
EXOSC2	0,014716878	0,896421867
NDUFB10	0,014734931	0,35107076
RGS16	0,014775268	0,560053147
M-RIP	0,014860897	0,824253516
APOC3	0,014939199	0,305820728

FREB	0,014963589	0,595956778
CSNK1A1L	0,015013418	0,272202784
GPATCH8	0,015033991	0,375034367
LYPLA3	0,01507863	0,989761122
SLC27A6	0,015099554	0,770054871
CCDC93	0,015180713	0,189693309
OR5M3	0,015197403	0,583296474
WDR72	0,015247683	0,540313803
APCDD1	0,015320243	0,685982
TAT	0,015350996	0,857612842
PROL1	0,015354724	0,638158425
RIOK2	0,015378424	0,877222436
DSCR1L2	0,015422115	0,608072998
LOC116236	0,015540493	0,833462342
MRPS2	0,015578778	0,527453932
RPE	0,015626036	0,775756328
FLII	0,015647451	0,961446677
TRPM5	0,015664859	0,418782355
CHIT1	0,015788568	0,814199972
CDH13	0,01581676	0,754246786
LOC653498	0,015821447	0,354530908
NRN1L	0,015838299	0,564171365
MGC4172	0,015863353	0,547372611
TNFRSF18	0,015906061	0,236415057
CENTG3	0,015910294	0,703787684
LOC727845	0,016058571	0,750024946
WDR40C	0,016114443	0,876258378
SHPRH	0,016132451	0,479547546
OPRD1	0,016279568	0,891216762
NKAIN4	0,016282502	0,536608505
LOC644334	0,01639003	0,304051693
LIPN	0,016415744	0,662091575
MYLK	0,016512995	0,646272179
FAM129B	0,016566973	0,237355246
CCL15	0,016567743	0,906353403
MS4A3	0,016635183	0,922243883
CTSD	0,016696663	0,338551332
GAGE1	0,016747314	0,875814846
LOC728812	0,016772883	0,348630352
HYOU1	0,016934955	0,727271514
LOC650137	0,016972506	0,427403091
PPP2R4	0,017046728	0,890271171
OR56A4	0,017061089	0,730829354
DRD3	0,017090102	0,194903704
LOC652189	0,017206178	0,646592193

EHBP1	0,017326594	0,216435399
DHRS4	0,017393383	0,915744865
LOC441018	0,017452487	0,516372456
AMBRA1	0,017533357	0,584259616
NUBP1	0,017575397	0,436035186
TK2	0,017579574	0,730759578
SLC26A9	0,017640343	0,250982032
SLC6A9	0,017650883	0,437938175
LOC643031	0,017651906	0,874168537
HMMR	0,017654739	0,882863494
TXNDC4	0,017749431	0,56745133
TCTEX1D2	0,017756115	0,842541934
FUT11	0,017809095	0,743041934
MC5R	0,017844927	0,448445868
LOC652260	0,017870614	0,526351285
LOC729175	0,017885163	0,913361771
ZNF180	0,017914365	0,40282654
LOC731946	0,01796909	0,568381841
PPYR1	0,017986622	0,614591547
CCNI2	0,018034698	0,981649386
C1orf127	0,018143099	0,288009105
LSP1	0,018147664	0,839472599
NET1	0,018213975	0,345518787
ELMOD2	0,018218221	0,285794276
KLRA1	0,018266353	0,716290518
HLA-E	0,018267031	0,641988343
CTCFL	0,018275284	0,727455672
SLC8A3	0,018322617	0,723892867
FBLN5	0,018341804	0,224720565
LOC729186	0,018355857	0,579071297
ITGAX	0,018387253	0,399058554
LOC643718	0,018474677	0,976070444
NEK5	0,018514751	0,680323831
LOC646173	0,018543418	0,194917713
DDRGK1	0,018605439	0,634012477
LIPA	0,018663581	0,607795955
PMVK	0,018693612	0,91999054
LOC651831	0,018702173	0,230815849
TTC26	0,018705834	0,919919262
LOC143458	0,018773724	0,322276931
ZNF30	0,01877997	0,764768306
TAF1A	0,018799855	0,284754336
C12orf23	0,018813013	0,291731447
WSCD2	0,01885304	0,296056942
MARCO	0,018877988	0,180480701

LOC729291	0,018932452	0,545026932
LOC649965	0,018958534	0,942647372
MAPRE1	0,018979922	0,44178724
FAM131B	0,018990958	0,610771032
LOC730248	0,019015808	0,600304148
CDKN1C	0,019059978	0,515194817
SENP5	0,019133496	0,297740978
CCDC14	0,019168462	0,672307122
SPAG9	0,019199156	0,472988766
PKP4	0,019217578	0,981239536
ALDH1A1	0,019381485	0,961090075
MAPK14	0,019514248	0,638640388
CLPS	0,019525752	0,312889471
hCG_23177	0,019551564	0,493724985
BCAT2	0,01956729	0,691605227
ADARB2	0,019568256	0,786045415
CXorf20	0,019618357	0,233657176
TMEM30C	0,019714742	0,721619864
E2F2	0,019739265	0,362752833
RODH	0,019748223	0,970501506
LOC652688	0,019779206	0,242013142
SRrp35	0,019790264	0,246887295
FBXL12	0,019796741	0,351983836
SSH3	0,019799325	0,668032711
hCG_1820801	0,019810726	0,508142139
MRPL16	0,019831	0,454538208
BAT8	0,019845227	0,369709027
OASL	0,019907615	0,610755727
MAGEA12	0,019941444	0,559912733
WBP4	0,019970375	0,526520885
ABHD5	0,020300343	0,506375722
ABHD13	0,020435262	0,318480059
OR10V1	0,020479951	0,484451602
KLHL36	0,020482729	0,849512944
LOC389342	0,020483103	0,406749565
NFYB	0,020532549	0,44006048
SPAG17	0,020557432	0,293666768
LEPREL2	0,0205685	0,392274266
RTP2	0,020613349	0,521673361
KAI1	0,020621479	0,628457292
ADAMTS19	0,020640547	0,527668899
KIF18A	0,020778743	0,999650094
ICAM3	0,020917984	0,517742429
SEPHS2	0,020979932	0,827726773
PRPS1L1	0,020984714	0,765926596

SSPN	0,021016053	0,962753379
OR5K3	0,021056605	0,405743708
FAM119A	0,021064405	0,268159995
NHP2	0,0210658	0,518382468
COL11A1	0,021253153	0,392804163
RSHL3	0,021263219	0,931618501
MUM1L1	0,021270801	0,822417204
LOC729543	0,021298563	0,866599378
ABO	0,021357061	0,354300916
C10orf81	0,021402821	0,795379437
ZBP1	0,02140449	0,465565327
AMIGO3	0,021466704	0,675378273
IDE	0,021601354	0,513354861
LOC730201	0,021651808	0,503367179
HIST1H1C	0,021660428	0,421754064
PCDHGA11	0,02167614	0,320895146
KIAA1982	0,021703563	0,567064936
DDIT3	0,021758496	0,67640762
ANK1	0,021781218	0,3998693
ELF1	0,021852582	0,843249873
NR1H3	0,021870508	0,375293115
L1TD1	0,021902255	0,512088185
CPEB4	0,022032477	0,698699154
HSPB8	0,022079027	0,935008098
IGBP1	0,022098362	0,702943574
IQCA1	0,02210565	0,80549485
FMNL2	0,022176281	0,313052399
C21orf45	0,02218966	0,210073422
LOC392275	0,022274347	0,796699201
K5B	0,022335605	0,672296793
U2AF1L3	0,022358316	0,578336366
IPF1	0,022460491	0,540850172
P2RY2	0,022497146	0,429030073
LOC392242	0,02258081	0,991163024
EFHC1	0,022607067	0,255967749
KCNB2	0,022631558	0,803045638
CYB5RL	0,02267177	0,891365376
LOC728768	0,022695977	0,7350731
MOSC1	0,022700083	0,712059816
MS4A6E	0,022811825	0,864334774
LOC643338	0,023046168	0,25619739
FBN1	0,023092721	0,703820754
RAB22A	0,02310477	0,730660371
TLR3	0,023111049	0,700773004
ZNF267	0,023120942	0,557261425

SPATA2	0,023154054	0,921921537
TAS2R38	0,023196258	0,393676858
EFNB3	0,023227282	0,604136118
C3orf36	0,023296655	0,859018172
C6orf27	0,02331336	0,863875638
LOC729245	0,023470424	0,988832454
CCDC82	0,023538366	0,416991588
CPNE6	0,023608873	0,850755948
LOC400652	0,023637706	0,836611942
LOC729982	0,023656958	0,875738937
EIF2A	0,023788519	0,538711078
SESN2	0,023938512	0,569435207
IQCD	0,023959681	0,377482852
ADCY1	0,024009002	0,302501111
HMGCR	0,024022659	0,409621423
SRGN	0,024031338	0,193148277
SLC9A5	0,02416911	0,765911979
MYH8	0,02423639	0,312343963
TCHP	0,024239099	0,297297679
LOC729303	0,024316064	0,80045721
LOC728777	0,0243261	0,663266092
MGC20419	0,024343357	0,892107253
HOXC11	0,024674148	0,929782004
ACAD8	0,024675313	0,586900551
MFTC	0,024703278	0,878772819
PVRL1	0,02474738	0,356498296
RAB11FIP1	0,024859978	0,419777408
PBX1	0,024863915	0,974596345
DSCR4	0,024905619	0,813043623
SFMBT2	0,024912615	0,906639314
CUTC	0,024972493	0,329261798
TR2IT1	0,025011962	0,562357326
PCDHGA5	0,02515008	0,617803943
TMEM77	0,02516097	0,905008992
RNF28	0,025165526	0,813830561
CCDC112	0,025373793	0,83116599
FLJ20298	0,025433806	0,586774281
CD3E	0,025455901	0,844259468
LOC652559	0,02551183	0,326166773
KIAA1704	0,025517551	0,18057261
C3orf53	0,025547042	0,539393821
LOC729287	0,025654504	0,398664257
SLC41A2	0,02566956	0,696792093
PAPLN	0,025814418	0,739761403
ACCN1	0,025827349	0,45627888

TAF6	0,025842845	0,777972187
LOC729599	0,025933259	0,231959483
ALDH1B1	0,026012135	0,459445255
GPC5	0,026024738	0,573764274
ARR3	0,026043589	0,31823902
PCDHA5	0,02613486	0,484872162
SLC22A13	0,026177495	0,630183432
PLEKHN1	0,026366483	0,963675897
KRTAP13-3	0,026425819	0,439471553
ZNF431	0,026444285	0,824719861
KIAA1545	0,026447182	0,313597429
AMDHD1	0,026456916	0,48457218
SYNGR3	0,02652508	0,640600387
RHBDF1	0,026525371	0,685955452
CACNA1A	0,026595431	0,626381995
NR0B1	0,026633439	0,930479642
LOC647038	0,026659747	0,743675648
GRIK5	0,026669193	0,558729681
LOC90835	0,026672391	0,437821693
LOC646425	0,026722326	0,283286372
SIAT6	0,026769717	0,918545935
LOC646746	0,026777745	0,792214239
NHLRC2	0,026894281	0,903157894
CEP350	0,026936766	0,972815832
RPL26L1	0,026970678	0,830456734
tcag7,875	0,027022482	0,606305187
LOC641835	0,027125382	0,414181156
FANCF	0,027163532	0,655636749
HSPH1	0,0271879	0,375549256
TRAT1	0,027199585	0,405446597
LOC728376	0,027214022	0,375115462
OR2B11	0,027244928	0,936012499
YTHDF2	0,027296209	0,540318083
KA35	0,027358879	0,964497135
LOC441454	0,027449712	0,419412514
LOC729765	0,027468097	0,181577592
MGC4796	0,02749097	0,850523052
NKX2-8	0,027606998	0,400733327
CHX10	0,027890596	0,698608184
EBF2	0,027945093	0,541430751
KLHDC6	0,027968359	0,413131283
NAB1	0,028056367	0,355563509
LOC650070	0,028097583	0,391975345
THTPA	0,028162764	0,327166785
GFOD1	0,028209423	0,374123406

OR3A4	0,028443716	0,170125892
LOC727739	0,028459309	0,611137945
FARSLA	0,028484401	0,791416281
SOD3	0,028518801	0,247753936
EIF3S8	0,028561956	0,207710315
NLE1	0,028579774	0,980510029
ACADS	0,02862735	0,564803513
UBTD2	0,028679819	0,978353117
KCNMB4	0,028693422	0,563212248
ABLIM2	0,028746993	0,34803148
ZNF7	0,028753657	0,95890937
HIF3A	0,028800781	0,62650545
FBXL4	0,028848982	0,378206978
LOC731397	0,028849159	0,661383185
NBN	0,02888581	0,381847019
LOC649771	0,028904252	0,981135956
QPRT	0,028962021	0,436159771
WDR17	0,029041592	0,446774199
LOC652231	0,029055181	0,854330894
STARD9	0,029055366	0,857671134
C9orf128	0,029064993	0,489048481
C1orf168	0,029107347	0,74708904
C1orf92	0,029194622	0,670685989
PRSS16	0,02928479	0,257416649
ICA1	0,029298351	0,935584686
LOC338579	0,029319449	0,497711215
ANKRD49	0,029398632	0,824689282
ZBED3	0,029472515	0,583788306
PARP3	0,029491377	0,631294605
SLCO1C1	0,029601027	0,97708539
JUN	0,029788793	0,204696737
LOC730713	0,029869725	0,409697548
TMEM100	0,029875442	0,835834944
C19orf2	0,029968058	0,781172518
SUSD2	0,029976116	0,682605121
EIF1AX	0,029979688	0,718835138
LOC732258	0,030092911	0,131118588
RNF187	0,030106094	0,638698038
EIF4G3	0,030150847	0,329292032
RNASE3	0,030166172	0,920970044
ATP6V1G3	0,030178175	0,410189515
NFKB2	0,03027144	0,240728583
SLC9A1	0,03029057	0,41325856
CARHSP1	0,030306127	0,334737817
FLJ11126	0,030372075	0,370019222

CREB5	0,030467958	0,371736785
LOC139249	0,030552052	0,759967149
ZNF561	0,030575016	0,692659928
LOC729842	0,030764901	0,26160075
LOC730205	0,030815989	0,363123966
BAIAP2L1	0,030933517	0,579974064
KCNA10	0,030950147	0,898216639
MAGEB2	0,03095853	0,707237454
FLJ10357	0,031160886	0,414086861
USP9Y	0,031200349	0,36476905
FAM81A	0,031339504	0,979933911
LOC150763	0,031344761	0,856062339
HMBOX1	0,031372193	0,93083154
C16orf85	0,031423432	0,606177242
LOC728789	0,031428817	0,607221977
CTSF	0,031454536	0,616068617
TMCC3	0,031531816	0,292419722
LOC388553	0,031540165	0,567635573
TCF2	0,031588522	0,487427403
EYA4	0,031594546	0,797599591
DTYMK	0,031614224	0,750628287
LOC441722	0,031637435	0,979214089
C20orf74	0,031698876	0,174075782
LOC729932	0,031792283	0,433305501
C11orf53	0,031860904	0,225588784
OR1A1	0,031921182	0,244821248
OPTN	0,031928209	0,374740194
COP	0,031999296	0,697914297
CCNY	0,032026052	0,579763027
S100A13	0,032074502	0,837878578
C13orf38	0,032120771	0,750167318
SLC35B2	0,032140768	0,531937558
PTGES	0,032172743	0,685322178
CDC2	0,032180299	0,837332501
MYH2	0,032215185	0,92159656
PRKAG3	0,032250099	0,235020244
MRPL47	0,032297997	0,755033119
PLK4	0,032451105	0,562174432
TMEM41A	0,032502875	0,224570683
RASA2	0,0325303	0,656431434
ELSPBP1	0,032547517	0,58088852
TNR	0,032603237	0,34954947
C9orf3	0,032631169	0,155323984
SS18	0,03269295	0,619880422
FAM123B	0,032715717	0,826873266

CALR3	0,03277718	0,353357042
TBC1D28	0,032790856	0,550087805
UBE2L3	0,03280787	0,229530255
CNFN	0,032986356	0,3570515
UCP1	0,032996049	0,225655883
HSPA14	0,032996087	0,205530092
LOC730019	0,033042472	0,909682929
C20orf96	0,033261728	0,753494328
CDCA4	0,033269375	0,978942207
LOC731533	0,033277552	0,250960357
PPIAP19	0,033345269	0,819454799
RDH8	0,03339806	0,847180525
KIAA0450	0,033406125	0,619622647
LOC648570	0,033530863	0,382974851
LOC730665	0,033587543	0,510145374
OTOP3	0,033625246	0,742419369
ACSM5	0,033770552	0,512769743
USP14	0,033828643	0,767182782
DPY19L2P2	0,033842524	0,880006955
DDX43	0,033848216	0,850700771
MUC19	0,033903253	0,536703571
ARFGEF1	0,03407606	0,35336
DUPD1	0,034081261	0,348899667
LOC647031	0,034087413	0,793219178
PGC	0,034114788	0,473261825
C1orf84	0,034145074	0,49122594
GSTK1	0,034336745	0,957660734
CC2D1A	0,034366061	0,969073009
DKFZP686M0199	0,034407883	0,596034346
FBXL7	0,03442754	0,471621144
TBC1D10B	0,034756311	0,228981089
C22orf4	0,034761351	0,275286592
CADPS2	0,034811761	0,906083657
NDUFB9	0,034851113	0,692008785
ANKMY2	0,034949287	0,418237196
FAM126B	0,034989706	0,521841366
MICALCL	0,035018807	0,571496801
DUSP18	0,035055224	0,461253141
MAGEB18	0,035069065	0,700338357
MRAP	0,035096795	0,164534182
ISG20	0,035108367	0,946659485
CRYAA	0,035177302	0,42409198
RIPK2	0,035236857	0,199171467
LOC729390	0,035490901	0,564253445
C4orf7	0,035499718	0,266843485

PELO	0,035517443	0,521626186
WDR73	0,035603741	0,785008763
CDH12	0,035613363	0,251852931
CLEC17A	0,035614273	0,482586752
LOC644489	0,03563313	0,284659297
MUC1	0,035634349	0,240147338
hCG_1646157	0,035685064	0,452140395
WDR35	0,035761715	0,374810189
WDR26	0,035794908	0,185498856
PEX10	0,035866078	0,254656676
LCE2A	0,03599263	0,526984688
OGDH	0,036001234	0,231654925
GLIPR2	0,036028746	0,365387948
LOC347364	0,036079766	0,374396814
TTC23	0,036081461	0,688104606
LOC728133	0,036096136	0,405859455
IL13RA2	0,036148604	0,862624942
FBF1	0,036167844	0,335682927
FLJ41649	0,036759199	0,872968777
ASB12	0,03680696	0,972301914
CTNND1	0,03690905	0,345208862
LOC121006	0,037005081	0,353965735
CCNYL1	0,037019855	0,307316495
CSNK2A2	0,037214983	0,324071893
HEY2	0,037235697	0,39836593
DCD	0,037326465	0,198204546
ABI2	0,037402131	0,400242822
RIC8B	0,037453167	0,662859309
ZFYVE9	0,037505425	0,502908181
SGIP1	0,037648109	0,261921251
LOC728173	0,037933666	0,725703214
C17orf59	0,038030027	0,391133593
KIDINS220	0,038075607	0,432426939
PADI2	0,038177982	0,241580514
GPR	0,038287195	0,788264186
CASRL1	0,038293362	0,88614567
LUC7L2	0,038354415	0,847278936
KAL1	0,038433766	0,279677162
LOC729973	0,038465609	0,375597262
LOC391358	0,038579777	0,825084979
CARD10	0,038631729	0,707661927
PRTN3	0,038703389	0,667959287
LOC728189	0,038716145	0,534814896
NKTR	0,038780543	0,223804676
HCCS	0,038794438	0,243284094

ASCC1	0,03883794	0,234304194
LOC344595	0,03885663	0,801639034
GADD45GIP1	0,038870127	0,593975081
KIAA1276	0,038997383	0,261898641
TNPO3	0,03904678	0,340046939
GTPBP4	0,039198536	0,719929761
MRC2	0,039252295	0,857679121
LYSMD1	0,039322901	0,544665248
LOC644419	0,03936574	0,417675454
KIRREL	0,03948658	0,340902274
LOC728306	0,039505503	0,159768296
LOC729407	0,039512453	0,861502546
CES1	0,039586374	0,979729175
RYK	0,039589875	0,435662438
LOC647275	0,039607837	0,985963131
ZNF31	0,039654567	0,929210008
CEP76	0,039711951	0,362376591
UBE2CBP	0,039779921	0,859602942
GAB4	0,039782224	0,539061011
CXXC1	0,039832089	0,452998466
FGR	0,039900537	0,286112483
FGB	0,039907716	0,502569966
PCDHGA3	0,039997306	0,880023003
LOC401387	0,040028601	0,826438707
EDG3	0,040138456	0,690320517
ELL	0,040177795	0,95146618
ARMC2	0,040192902	0,763133196
U1SNRNPBP	0,040237679	0,417832519
DUX1	0,040344211	0,401493383
LOC645146	0,04034697	0,673097494
CDKL1	0,040372699	0,779870001
DAPK3	0,040449108	0,73172402
SH2D1A	0,040460872	0,343414809
LOC646756	0,040469439	0,478808626
MBD2	0,040507438	0,366222103
SLC8A2	0,040548819	0,32813182
ST18	0,040579254	0,942816693
OR56B4	0,04061218	0,87904571
C9orf24	0,040635873	0,479899129
LRRC29	0,040731276	0,262954738
TBC1D20	0,04079204	0,973714331
RPN2	0,040814364	0,750469309
ELMO1	0,040955329	0,310081008
MGC3196	0,040990515	0,663954908
SEMA4A	0,041055579	0,461138215

SPEF2	0,041125036	0,434586457
MST1	0,041163418	0,314662588
ICOSLG	0,041177136	0,930531241
LOC399706	0,041280961	0,532520201
ZNF146	0,041328311	0,348325966
tAKR	0,041347153	0,256934478
TUBA3	0,041409397	0,877711269
CD226	0,041457651	0,921554466
NOTCH2	0,041549703	0,260643464
EIF3K	0,04155232	0,783516359
LOC652614	0,041571285	0,363509638
LOC644870	0,041574422	0,586277439
LOC340900	0,04160956	0,267979361
LRRK1	0,041652665	0,189528513
ANKRD33B	0,041700087	0,236012883
C7orf30	0,041761824	0,933509173
NGL-1	0,041875862	0,169391008
ITGA1	0,042155012	0,560966119
KLHL32	0,042303146	0,640501538
BPIL3	0,042340286	0,34172963
PLEKHG6	0,042448157	0,53266421
KCNMA1	0,042460848	0,264854266
DEFA4	0,042558445	0,869371606
IL17RD	0,042808666	0,367401241
ACTR2	0,042915975	0,177344251
BRP44L	0,042930887	0,860259788
LOC730074	0,042952519	0,129351566
RNF38	0,043143055	0,553775712
GAPD	0,043202468	0,802866019
SLC35F1	0,043248481	0,363863672
LOC399936	0,043346326	0,478041672
LY96	0,043406215	0,953290207
hCG_31916	0,043528544	0,591217807
LOC338611	0,043646323	0,551364423
EHD2	0,043693132	0,557497194
CYTSA	0,043722714	0,562930398
CKLF	0,043806708	0,531364607
LOC641894	0,043845464	0,177354609
ORMDL2	0,043862806	0,154456299
MBD3	0,043885786	0,298180759
OR5D18	0,043928527	0,627842286
MRPL18	0,044018795	0,807844241
LOC652825	0,044080731	0,327421495
RBM12B	0,04423141	0,481049531
ROBO4	0,044315045	0,76676487

KIF3A	0,044449359	0,454045671
PIK3R6	0,044477292	0,262693208
C20orf191	0,04450925	0,33988827
CCDC36	0,044524603	0,651960755
C1orf88	0,044560081	0,772762527
UNC45B	0,044635443	0,180124869
LOC149351	0,044823009	0,511771736
CACNG7	0,044883994	0,345020709
GJA7	0,04490476	0,52111731
GMCL1	0,044922287	0,404517502
AP2A2	0,044953643	0,397380668
FLJ12985	0,044974842	0,624628728
SCRN2	0,045054667	0,71572207
ESF1	0,045090849	0,575565207
GJA5	0,045206289	0,915045269
MOBP	0,045265593	0,993065773
GPR115	0,04526676	0,96940054
C7orf52	0,045337618	0,304573976
GOLGA4	0,045426526	0,935594008
SNF1LK	0,045587576	0,30141245
RMND1	0,045608595	0,894627794
PAK1IP1	0,045647237	0,413265234
CXorf39	0,045657436	0,456798062
HNRPA1	0,045660604	0,358789039
ABHD1	0,045717053	0,950290905
LOC401442	0,045717716	0,405858672
ZNF354A	0,045719309	0,754263652
hCG_1776980	0,045734629	0,227794047
ARNT	0,045754376	0,517933081
LOC729506	0,045791555	0,285812663
HNRPA1P4	0,045829527	0,219290256
LOC91807	0,0458357	0,459931287
TRPA1	0,045873833	0,774626329
TATDN3	0,045878855	0,754713463
CLECSF13	0,045945462	0,450205834
STAG1	0,045950383	0,495742764
C1orf213	0,046033368	0,946906455
TBC1D4	0,04616302	0,300544668
LOC729224	0,046234963	0,512808372
MYR8	0,046243792	0,347305752
ACTB	0,046248869	0,362035232
CLCN5	0,046253933	0,48500715
LOC147650	0,046298997	0,884711403
MRPL37	0,046395894	0,251418423
LOC649055	0,046444104	0,888679724

NPL	0,046503968	0,85649543
APOL1	0,046557639	0,996013692
PNCK	0,046692269	0,531479805
DRGX	0,046750215	0,363825141
SLCO2B1	0,046784481	0,755248414
PYROXD1	0,046791162	0,941146865
MICAL2	0,046891261	0,741544583
HIST2H2AC	0,04689465	0,243673081
PLAC4	0,046987326	0,284821784
RPS17	0,047024273	0,876274608
KLHL9	0,047058294	0,88553562
PRSS21	0,047150704	0,371132768
CACNA2D4	0,047172507	0,631433941
ALG10	0,047204178	0,49037737
IL28RA	0,047311435	0,823841107
MGC46336	0,047334132	0,968031877
C1orf184	0,047343467	0,906696487
KLK6	0,047397677	0,294287054
ERCC5	0,04742146	0,808090564
HUMAUANTIG	0,047476464	0,891901614
LOC651686	0,047485266	0,22893348
PXDN	0,047808197	0,200328308
GPR73L1	0,048137892	0,976937828
LPHN3	0,048283865	0,87074593
TTC36	0,048619069	0,55639329
ANKRD1	0,048648414	0,820524674
PLSCR1	0,048651732	0,733929387
DHDH	0,048704385	0,5208387
GIMAP6	0,048719201	0,292992285
LOC652449	0,048741123	0,97165461
GAPT	0,048841943	0,870906419
KATNA1	0,049001779	0,308756405
CCDC81	0,049046347	0,299621333
VTI1B	0,049067353	0,382937257
FLJ46082	0,04918515	0,246025693
FLJ23614	0,049197096	0,563743395
LOC728716	0,049206256	0,68393698
CD164	0,049218272	0,455658682
RGR	0,049406959	0,763399967
IGFBP3	0,049408929	0,923866398
COX6B1	0,049597309	0,338180242
LOC646793	0,049671561	0,815844966
C6orf156	0,049686704	0,581708773
C1orf96	0,049690795	0,555791853
TRIP	0,049697304	0,689187924

PRDX6	0,049833592	0,472944588
BCDIN3D	0,050170861	0,399769109
DPF2	0,05040053	0,794448653
LOC648691	0,050485077	0,641949301
LOC402120	0,050490273	0,759545514
LRRC1	0,050510631	0,478744436
HMGA1	0,050683539	0,337354626
SLC25A20	0,050712923	0,38000362
RDH14	0,050732556	0,796312352
LOC644276	0,050849741	0,270654715
KSR2	0,050858747	0,337335793
CDH22	0,05086848	0,688133953
OR2T1	0,05089086	0,719570772
OR52K2	0,050925428	0,597817517
RRN3	0,050931566	0,894583956
LOC650717	0,050972782	0,189537208
CHIC2	0,05105289	0,596539069
RIPK1	0,051131045	0,259023451
CHN1	0,051140194	0,824723549
FAM155A	0,05120173	0,35194052
LOC388503	0,051325813	0,316282845
MMS19L	0,051369626	0,93142443
LOC285423	0,051565981	0,667017066
SORCS1	0,051714848	0,231209662
PECR	0,051728681	0,26220566
RELL1	0,051740709	0,638867756
COL4A3	0,051751058	0,983802081
MSL2	0,051775734	0,581067819
POU3F4	0,051809769	0,318915768
F11R	0,051871927	0,525282094
PPIA	0,051952644	0,763707138
TBX3	0,051958362	0,899275197
LOC728196	0,051959152	0,281511125
FCHO1	0,051996633	0,177049351
GUCY2D	0,052000348	0,896041603
DNAJC7	0,052010594	0,828211814
LOC729601	0,052014115	0,367315285
LOC729558	0,052120346	0,554455047
MPL	0,052181269	0,660067595
C17orf53	0,052265466	0,211694183
AGTRAP	0,052316375	0,690703486
PLK3	0,052418246	0,433786941
ACADSB	0,052419002	0,29996828
GARNL1	0,05244263	0,836562694
EIF2B3	0,052471399	0,864851289

EIF2S1	0,052562822	0,428554076
CD300LG	0,052626947	0,741868118
FKSG24	0,052795623	0,461143502
ALKBH5	0,052814797	0,749337711
DGAT2L4	0,052851783	0,535202433
KNCN	0,052854696	0,992776632
CHRNA7	0,053046627	0,185376834
STIM2	0,053084608	0,218484416
LOC731915	0,053089898	0,196578228
CTSW	0,05347492	0,631783911
OCEL1	0,053506222	0,224063583
TMEM106A	0,05356209	0,227617587
PAX3	0,053610227	0,393380292
DBH	0,05363905	0,734133213
SEC11L2	0,05364776	0,267361161
PSEN1	0,053734395	0,537220083
STXBP5	0,053738954	0,852961225
LOC729989	0,053741894	0,998919557
EREG	0,053759647	0,580029609
ARPC4	0,053842685	0,348807042
INMT	0,054073939	0,359928491
ANO10	0,054121718	0,386980915
DRAP1	0,054222499	0,843142742
KCNV1	0,054241759	0,279061082
LOC731467	0,054352329	0,406656246
LOC728927	0,054402124	0,759129505
CPZ	0,054490103	0,958923216
HIST1H1B	0,054513576	0,66950655
NGRN	0,054533258	0,187774296
CC2D2A	0,054556653	0,22186069
GPX7	0,054652525	0,978474405
C3orf18	0,054700319	0,625032578
PTGDR	0,054726355	0,873311714
PGBD2	0,054731384	0,463255718
FLOT2	0,054777528	0,448535422
CPLX2	0,054872038	0,6779179
LOC643652	0,054872171	0,586991243
RRP9	0,05493555	0,995664062
MAP3K6	0,055099759	0,795979676
PRDM4	0,055128949	0,258115393
CCL3	0,055204584	0,365034795
GPR153	0,055212973	0,338326071
LOC727771	0,055417631	0,535812382
CBWD5	0,055497081	0,839557191
LOC646989	0,055518403	0,280222252

CYP2C9	0,055586419	0,983368089
SERPINI1	0,055706417	0,293434165
DHX35	0,055707145	0,566937566
C6orf114	0,055707961	0,742545045
MOBKL2B	0,05571209	0,326835969
hCG_38984	0,05572999	0,570942095
LOC729639	0,055730327	0,850251101
ZNF205	0,05575155	0,71984819
04, Mrz	0,055763607	0,277834968
ACCN4	0,055767217	0,691779878
OIT3	0,055793367	0,989730622
SREBF2	0,055863673	0,496412633
FAM152A	0,055953905	0,997912803
SLC25A22	0,056051093	0,193744436
LOC729903	0,056172535	0,296913889
C1orf65	0,056390995	0,252796209
ROBO2	0,056558384	0,303036085
HTR7	0,056605304	0,77640458
HOZFP	0,056719484	0,295176918
LOC339970	0,056724206	0,412875022
LOC730224	0,056771121	0,553900105
COX6A1	0,056788175	0,770110554
FEZ1	0,056831552	0,513969846
ETV3L	0,056888557	0,771569257
LOC653348	0,057045919	0,320063292
LOC644191	0,057046041	0,310439988
TRIB3	0,057058693	0,336012106
SMPD1	0,057171334	0,259866911
LHX3	0,057185372	0,415213758
PDE11A	0,057205496	0,624655489
TRAR5	0,057246056	0,511454756
PAK3	0,057338501	0,882975561
ZNF239	0,057360678	0,865400882
NPAL1	0,057550889	0,489314226
WDR89	0,05761191	0,564232101
ACADL	0,057702526	0,747517807
LOC730007	0,057760799	0,337415249
ZNF142	0,057761715	0,999571076
COL23A1	0,057849863	0,230717159
ODAM	0,057862879	0,530925208
ANKRD2	0,057889744	0,127318867
LOC400950	0,057915387	0,283883166
NCAPD3	0,057933387	0,789229893
C12orf45	0,057995209	0,915827354
LOC283951	0,058033713	0,200485218

FGFR1	0,058066391	0,7313692
AVPI1	0,058074468	0,415813265
FLJ16046	0,058101435	0,310936827
C3orf38	0,058127098	0,7477508
ME2	0,058200348	0,912076663
KNSL8	0,05825932	0,442435403
BTN1A1	0,058323193	0,916783521
FAM76A	0,058422554	0,374112187
LOC652022	0,058479244	0,975703084
TMEM175	0,05848827	0,938690321
GPR132	0,058514252	0,71477998
ODF3B	0,058515727	0,414695904
LOC642539	0,058685902	0,89949296
SLC7A13	0,05872808	0,531778594
DSN1	0,058763293	0,23472667
LOC731984	0,05883315	0,533076025
COX18	0,058870829	0,578746437
TTC30B	0,059025033	0,132555278
KLHL10	0,059108991	0,635423316
GPRASP1	0,059192077	0,633886038
RPL21	0,059276937	0,32609783
LOC92270	0,059294081	0,979229192
PUS10	0,059323494	0,452909262
KIAA0663	0,059361668	0,359406159
MGC24132	0,059377122	0,152381151
CDX4	0,059466386	0,367732804
ZNF221	0,05947027	0,669063641
GLRB	0,059502641	0,216048669
STRBP	0,059554773	0,989770792
KRT3	0,059645414	0,240649341
FAM62B	0,059653106	0,174288671
SPRED1	0,059695981	0,267459743
BACH2	0,059855569	0,572457233
LOC387647	0,059866317	0,923943982
DOK6	0,059908682	0,439291096
CDK2AP2	0,059910455	0,358286057
FBXO21	0,059963286	0,750034052
MGC34829	0,060026938	0,663521982
CSNK1G1	0,060059347	0,790263678
TNFRSF6	0,060130208	0,741879477
PDE6H	0,060140303	0,594386661
DNM1L	0,060214176	0,583756226
SUPT4H1	0,06026132	0,807812891
USP33	0,060381685	0,563957386
MON1A	0,060465723	0,499311574

PLXND1	0,060496931	0,341924017
TXK	0,060539862	0,865088703
SSSCA1	0,060712169	0,28383171
LOC286238	0,060759722	0,363357134
FLJ42957	0,060782899	0,700329667
HIST1H4C	0,060848446	0,411085404
RNF130	0,061068639	0,571859745
PGBD3	0,061092553	0,787156262
NUP210	0,061165661	0,816819637
TUSC5	0,061185561	0,626224377
PSG2	0,061207616	0,707229626
ASH2L	0,061291402	0,895199267
SRFBP1	0,06140651	0,899873895
LOC654259	0,061628132	0,726990338
ASAH2C	0,061698042	0,831477482
TMEM30B	0,061760812	0,664389118
POU3F2	0,061837649	0,619335505
NDRG2	0,061914463	0,731615775
PPP1R10	0,061989881	0,191098075
IER5	0,062002533	0,485058612
HAPIP	0,062039848	0,480804795
KRTAP5-2	0,062213008	0,964762251
C20orf118	0,062280542	0,978974969
C3AR1	0,062296429	0,434338025
TWIST2	0,06229886	0,562014685
ABCA6	0,062315046	0,759023031
EXT1	0,062548665	0,737475809
SMC1L1	0,062610153	0,363820124
FAM65B	0,062615287	0,925216285
OLFM4	0,062776016	0,996216678
PIP5K2A	0,062813051	0,932269988
MGC15763	0,062818713	0,706470498
AMAC1	0,062834534	0,907077894
LOC729249	0,062892422	0,355278251
AHSG	0,062894501	0,777187085
LOC731489	0,062906865	0,596666003
empty	0,063027835	1,88E-05
ZNF518	0,063032653	0,795415615
LOC727890	0,063052055	0,263725943
TAC3	0,063064766	0,962235982
LOC286434	0,063243027	0,104352293
VCP	0,063339182	0,381290422
C21orf129	0,063354031	0,462987356
VPS39	0,063518822	0,249040132
RAB4B	0,063606738	0,33506758

CABLES2	0,06377118	0,278162587
ANKRD10	0,063856781	0,311656496
LOC646195	0,063892547	NA
APOBEC3D	0,063998326	0,397821416
FHL2	0,064038945	0,339658935
GNAZ	0,064069013	0,913808608
ZNF445	0,064085837	0,328987916
CDC23	0,064110783	0,520167766
HTR2A	0,06411252	0,564926143
SLPI	0,064190208	0,522363952
C6orf65	0,064278136	0,21433291
DPP3	0,064349585	0,47770075
TNFAIP8	0,064358406	0,543724599
CDC37L1	0,06438491	0,486476329
WDR21C	0,064385033	0,448265973
ERGIC2	0,06466637	0,81244032
LOC731442	0,064684517	0,720603108
FOXC2	0,064714015	0,682403789
ESR1	0,064717839	0,54092778
C15orf45	0,065010855	0,433276667
SHC2	0,06518261	0,892028073
hCG_2003663	0,065196447	0,534012521
THG1L	0,065465197	0,404789308
CFHR5	0,065527822	0,351396783
LOC645676	0,065534528	0,574535594
SLC4A10	0,065671472	0,942199553
RP5-964H19,3	0,06579511	0,365572682
CADM2	0,065796425	0,795998374
RAB9A	0,065868024	0,642002737
RPL35A	0,065951484	0,847145341
PSMB10	0,065969186	0,501047842
ZNF75A	0,065992722	0,657038362
LOC441655	0,066152393	0,640474292
KIR3DP1	0,066173199	0,947619273
PLAC1	0,066223376	0,878608902
SOD2	0,066303216	0,21039855
LOC644929	0,06631078	0,733116536
CD69	0,066351261	0,837631405
ERO1L	0,066428248	0,507725645
SERPINA5	0,0664637	0,261225362
LOC729850	0,066656019	0,445588149
CNPY3	0,066728564	0,591971325
CHRM5	0,066772964	0,603511401
LOC646279	0,066807889	0,954012825
CHEK2	0,066831816	0,499492687

SCLT1	0,066875143	0,812165942
LOC729051	0,066899619	0,198459715
OPA1	0,066904019	0,907220899
MT1G	0,067010719	0,602335336
MRPL30	0,067013972	0,493680826
LOC729987	0,067106336	0,551135392
CEACAM16	0,067174644	0,353045536
CHGA	0,067252975	0,595388855
KIAA1361	0,067349931	0,784539667
TBX22	0,067603163	0,372844963
RALYL	0,067609461	0,504066229
hCG_2004593	0,067614472	0,426519706
LOC732052	0,067617361	0,841636474
COPB	0,067773902	0,816138581
CCDC89	0,0679143	0,994020862
C10orf25	0,06792792	0,4843241
HSPA2	0,068013483	0,861630303
LOC730062	0,068089246	0,760170535
LOC730712	0,068149773	0,896007181
RAB2B	0,068406607	0,286611171
TMEM88	0,068436048	0,466510957
NDST4	0,068469923	0,321130849
EIF1B	0,068562316	0,908675667
ZNF807	0,068632265	0,362474933
SARA2	0,06871805	0,696171426
C10orf78	0,068795371	0,812377773
LOC642666	0,068863337	0,893057679
FCN3	0,06892385	0,187901354
BMI1	0,068953745	0,945527624
LOC731447	0,068968644	0,903049852
C10orf112	0,0690015	0,324145554
TMCO4	0,069058423	0,287505928
DRB1	0,069177097	0,810867234
NME5	0,069203191	0,505826246
CD244	0,069217821	0,936023421
MGC44328	0,069267546	0,305142719
CAPNS2	0,06954374	0,296432824
RBM28	0,069702986	0,218277795
KPNA1	0,069768811	0,452474387
RNASE7	0,069779327	0,484119049
ADAMTS3	0,069790947	0,918722601
LOC730957	0,069813085	0,98981555
KLF3	0,06982061	0,296854268
MRPS35	0,070060681	0,812459262
LOC391656	0,070107418	0,841452412

LOC728581	0,070134841	0,959677739
TADA1L	0,070184111	0,284409896
APEG1	0,070255852	0,990908684
YIPF7	0,070391119	0,457703158
KIAA1530	0,070403724	0,211548222
KRT12	0,070404388	0,487963801
TFDP2	0,070539978	0,745908895
NR1H2	0,070676152	0,698021894
SPAG5	0,070676424	0,211909991
SLC44A1	0,070689296	0,332843864
SPO11	0,07069543	0,182318883
C11orf34	0,070702364	0,464397061
03, Sep	0,070747943	0,778725235
BCL10	0,070770974	0,728435566
CLCN3	0,070826681	0,668570972
CD63	0,07084229	0,442046528
MAPK12	0,070881402	0,173420254
FOXL2	0,070950196	0,845963971
ARHGEF12	0,070961618	0,449744195
BARX1	0,070972991	0,918564382
TBP	0,071055575	0,16356583
ALDH3B2	0,071117004	0,970011415
WNT10B	0,071167284	0,220598471
FLJ14800	0,071228411	0,230708742
ING4	0,071287389	0,609387807
TIGD3	0,071368749	0,971586412
LOC651825	0,071428815	0,808980795
SERPINB8	0,071442207	0,93731195
STAR	0,071470684	0,832600993
FLJ46321	0,071560206	0,202411829
RAB17	0,071617625	0,380274835
LOC727910	0,071643865	0,254301066
LOC401296	0,071653489	0,618027826
TRAF7	0,071712008	0,936608815
CYR61	0,071803861	0,66124164
TMSB4Y	0,07187813	0,238497061
UBE1C	0,071921888	0,194760238
C5orf24	0,071930848	0,617675747
ZNF428	0,072090057	0,297202681
STX16	0,072204283	0,357861607
LOC729126	0,07229932	0,562535192
AGTR2	0,072334412	0,97255492
BRD3	0,072618497	0,642181831
CCDC38	0,072648781	0,604865619
SLC30A8	0,072676641	0,654743593

STK11IP	0,072720485	0,292104581
LAMB2	0,072772945	0,15019508
LOC644941	0,07281571	0,366775971
C6orf35	0,072960233	0,802671965
C10orf129	0,072963425	0,253360466
ZER1	0,073070134	0,207532141
RNF103	0,073121388	0,763312283
NCKAP1L	0,073215209	0,925344195
GML	0,073266	0,210550377
LOC284998	0,073282374	0,13854857
SDR39U1	0,073339084	0,734463311
VN1R5	0,073542562	0,215067565
OR1J4	0,073550728	0,203464384
WBSCR28	0,073576761	0,481060729
KRT7	0,073599607	0,239493792
UNQ9368	0,073634724	0,455019043
LOC648153	0,073688151	0,390727019
ANK2	0,073729716	0,407595481
C1orf216	0,073921816	0,969079983
MKLN1	0,074127994	0,564638501
MYLK2	0,074255756	0,837550035
FAM154B	0,074271248	0,448112202
ITGB3BP	0,074369731	0,701268032
SLC35F2	0,07446139	0,870450764
LOC389333	0,074535761	0,767292082
PTD004	0,074535797	0,762445005
FAM150B	0,074573815	0,937628182
LRRC30	0,074602667	0,338757599
SLC22A18	0,0746692	0,434921637
SURF4	0,074781376	0,33596323
N4BP1	0,074807684	0,867204089
BRP44	0,074966586	0,560195894
SACM1L	0,07499178	0,403125781
TCP11L2	0,075015652	0,308577475
CRYBG3	0,075116924	0,406012847
LOC729773	0,075206149	0,485043043
KRTAP13-2	0,075225527	0,170042945
ARSF	0,075269218	0,892239788
OR13F1	0,075300467	0,640070704
UBE2D3	0,075319597	0,587595364
PACSIN1	0,075331896	0,4476398
STARD7	0,075480021	0,838834371
POR	0,075578831	0,582997804
NOL11	0,075597746	0,490825917
DPCR1	0,075614067	0,535642733

ENO2	0,075623493	0,998440978
PLSCR4	0,075635853	0,558162613
CLDN3	0,075686139	0,494585254
NOV	0,075694748	0,283263211
PPIF	0,075751284	0,272350592
ZSWIM7	0,075899982	0,822434564
FLJ40176	0,075944078	0,186779351
CD34	0,075963033	0,193073281
SOX9	0,075993265	0,281445211
ARMC10	0,076000176	0,955585997
CST5	0,076054749	0,424730958
SDR-O	0,076072203	0,263099941
CLK3	0,076101788	0,979377105
LOC389405	0,076271385	0,581468524
DUSP9	0,076377081	0,987586039
PRY	0,076393584	0,480805901
C22orf28	0,076427563	0,374637787
GPR161	0,076499107	0,377795015
LOC728030	0,076599847	0,355574216
CSTL1	0,076744831	0,756283696
LOC652645	0,076744877	0,822622946
TECTB	0,076776784	0,603205574
BBAP	0,076874286	0,168212542
TBC1D10	0,076924164	0,246734984
C9orf135	0,076976762	0,532298875
A2BP1	0,077005848	0,629794251
LOC730126	0,077088105	0,610530262
PSMAL/GCP III	0,077115828	0,417603527
SLK	0,077163562	0,848747885
FAH	0,077243349	0,201219158
ARID2	0,077254358	0,838991662
LOC646359	0,077408366	0,900753159
AMMECR1	0,077468629	0,198402948
LOC729072	0,077512812	0,173811083
DTX1	0,0775471	0,5628276
PRKCDBP	0,077602748	0,291367943
KIAA0195	0,077603757	0,819105771
OR2D3	0,077611615	0,39194725
CKAP4	0,077671391	0,580845352
Bit1	0,077773823	0,861367589
EDG4	0,077855089	0,339667034
RCHY1	0,077904558	0,405731134
LOC729772	0,077929841	0,95310759
FER1L6	0,077937743	0,735012402
RAB27A	0,077950819	0,821725729

UBAC1	0,077951124	0,625952245
LMX1A	0,078182216	0,323212752
FKBP10	0,078248189	0,5689633
ACTN2	0,078316834	0,52756567
ZBTB44	0,078327791	0,749083542
POLR2E	0,078362227	0,617861811
LGICZ	0,078394646	0,440839715
HRASLS5	0,078406335	0,230268222
UGT3A2	0,078413363	0,225086661
LOC284541	0,078465097	0,961624345
RACGAP1	0,078526548	0,255946436
FLJ42177	0,078551535	0,814185815
ROR1	0,078754075	0,603988145
LOC647805	0,078793699	0,207114819
LUZP4	0,07890885	0,55158686
IFNA8	0,078999068	0,546705727
APC2	0,079008226	0,895202426
C12orf4	0,079047346	0,227902398
LOC729578	0,07915471	0,81786647
C1orf172	0,079154806	0,612604714
MAGEA5	0,079183916	0,376748446
THEM4	0,079214725	0,197981408
EPN3	0,079252234	0,912103667
Magmas	0,079352259	0,350701981
GNB2L1	0,079444167	0,626516872
PITX2	0,079468847	0,823879128
NOL7	0,079480615	0,856925201
SAMD5	0,079503854	0,733970488
NXF1	0,079512355	0,490234317
RHPN2	0,079689525	0,156390441
TFB1M	0,079712471	0,577214874
ARPC3	0,079737573	0,238725013
GRXCR1	0,079745223	0,88135339
ADH6	0,07990349	0,405678706
PMM1	0,079912888	0,358395897
VN1R4	0,079918237	0,135105178
LOC729920	0,079918238	0,845774924
LOC649563	0,079932973	0,580710302
CIB3	0,080098142	0,299050466
TCEB3B	0,080121584	0,990435975
KIAA0626	0,080153359	0,71496371
NUDT18	0,080172305	0,541595952
FOLR1	0,080209759	0,289943457
DSPP	0,080211155	0,674504874
LOC728625	0,080224294	0,625946521

FBXO18	0,080252254	0,520671787
DNCH1	0,080267093	0,52994368
HRI	0,080334763	0,159030624
BMF	0,080434028	0,680814469
ODZ1	0,080465324	0,630754297
KCNIP1	0,0807819	0,702671032
C1orf81	0,080798363	0,178862946
MRPS33	0,080858306	0,225409415
ATG9A	0,080861982	0,190217359
FNDC8	0,080865625	0,975843756
LOC646719	0,080924586	0,970879575
EDC3	0,080979343	0,146497813
KRTCAP2	0,081138098	0,502690047
ELKS	0,081228649	0,244199361
LOC647082	0,081521285	0,549179731
MYCBP	0,081604792	0,695226069
ATP8B4	0,08161572	0,371108868
SAT2	0,081618731	0,586985274
ZBTB16	0,081692125	0,990999339
GRPEL2	0,081707676	0,871292808
MIPEP	0,081847951	0,554950384
PSD2	0,081877246	0,508675436
C1orf58	0,081971253	0,195094952
TIMD4	0,081976373	0,207526745
GPR158	0,081989704	0,634588199
LOC729352	0,082010734	0,88549571
TCERG1L	0,082075757	0,926741182
RBL2	0,082145089	0,198398276
ZBTB7	0,082208575	0,756849206
LOC731855	0,08222321	0,907182777
APEH	0,082224759	0,381463639
SCNN1B	0,082237254	0,674890025
RFC5	0,082241875	0,378387046
CAPN12	0,082271053	NA
VN2R17P	0,08239503	0,338892496
GPRIN2	0,082689527	0,693149623
MUC15	0,082792101	0,273185849
COL21A1	0,082913778	0,279080542
MGC39650	0,082961263	0,267809195
CCDC87	0,082985543	0,722272919
TRIP10	0,083077397	0,811401867
DCBLD2	0,083141458	0,320680347
ZNF154	0,083168554	0,874183557
PRRX2	0,083237161	0,178214853
MS4A7	0,083249425	0,858130531

SLC25A30	0,083340431	0,945628166
ACTN1	0,083375359	0,584023024
SQSTM1	0,083427325	0,438164851
ELK1	0,083433553	0,724772771
TTC4	0,083510533	0,398624924
EPPK1	0,083524884	0,639208307
LOC729751	0,083636169	0,244199167
GBA3	0,083662493	0,408404178
PASD1	0,083762237	0,902480083
COX4NB	0,083782383	0,972988573
SSTR4	0,083824283	0,441258894
OR8B12	0,083874212	0,783207879
LOC727903	0,083875903	0,679104556
PHKG1	0,083998601	0,591708804
WDR42A	0,084023665	0,699902676
ANO9	0,084143842	0,506150287
FLJ14011	0,084322657	0,34646164
CMTM7	0,084348455	0,500595858
TAF9L	0,084396432	0,355326144
C5orf45	0,084439601	0,345139133
FLJ35776	0,08453773	0,848056775
TAF5L	0,084664906	0,273585321
LOC730151	0,084698729	0,25031254
HNF4G	0,084725721	0,876938412
CGNL1	0,084823264	0,091781025
PPP1R14A	0,084895696	0,817256943
OS9	0,085023993	0,283066475
LOC729738	0,085212615	0,701406959
KCNB1	0,08524053	0,29027261
NDUFB3	0,085314947	0,468882602
TRIM8	0,08545192	0,582481192
AMOTL2	0,085522219	0,3404414
WDR42B	0,085601581	0,864982325
ZNF507	0,085648312	0,776984241
LOC730254	0,085686863	0,922011969
LRRC23	0,085746823	0,952448218
COQ10A	0,08575486	0,298626704
PKD1L3	0,085815923	0,887968016
RPS27	0,085816989	0,882875384
UGT2B28	0,085949824	0,664565738
FCRL6	0,085975663	0,698161292
LRRC10	0,086021378	0,93232572
SGSM2	0,086112805	0,929007318
SGKL	0,086145312	0,383386675
UBXN6	0,086282145	0,764580562

SRPK1	0,086302996	0,166815443
IDH3G	0,086379561	0,742103068
CXorf40A	0,086437612	0,595035469
KIAA1409	0,086451265	0,197171417
ALS2CR7	0,086464971	0,523168467
LOC388333	0,086708084	0,146332075
VARS2	0,086722894	0,766261623
HIST1H2BF	0,086725599	0,421592137
TSPAN9	0,086799929	0,589285408
ITGB7	0,08680435	0,777264879
ANKMY1	0,086822388	0,299521587
MYL6	0,08685484	0,914559855
RASA4	0,087067146	0,323891457
PCIF1	0,087085898	0,456623441
ZNF195	0,087087627	0,258142926
RBL1	0,087096622	0,700674012
SULT4A1	0,087247464	0,461224824
COL25A1	0,087350009	0,407851913
REV3L	0,08735576	0,278714333
ADAMTS8	0,087381926	0,490960673
PSMD9	0,087388896	0,543450496
ZNF304	0,087391559	0,33777224
PRKRIP1	0,087536894	0,942483609
AKT1S1	0,087828858	0,233545956
LOC652557	0,087942497	0,140232797
DBNDD1	0,08806193	0,513677744
RRP12	0,08812121	0,878199273
SLC39A1	0,088215007	0,566209483
ISG15	0,088370202	0,297529323
PIGF	0,08844429	0,135133085
CCDC140	0,088454957	0,647305045
SPN	0,088673525	0,22262442
OR13C8	0,088693239	0,178385855
FAM66C	0,088712077	0,964072582
SYNPR	0,088803281	0,569135748
LOC643412	0,088919491	0,187010072
SLC28A1	0,08896483	0,471815055
LPIN2	0,088975213	0,569809833
LOC652179	0,088986543	0,207069954
C19orf70	0,089000826	0,323624464
SPERT	0,089036633	0,790537255
UBTF	0,089067391	0,595803779
PTX3	0,089107046	0,16910212
LOC727977	0,089157134	0,29419901
LOC729851	0,089418301	0,709955964

CST8	0,089431848	0,767584029
LOC388458	0,089450274	0,520460708
NGB	0,089497189	0,38414325
ARHGDIG	0,089533859	0,244889281
CNPY4	0,089538638	0,318709917
IPO4	0,08956448	0,496360119
CIT	0,089566067	0,204728672
RHOG	0,089618236	0,950574082
RSPH3	0,089763907	0,256640698
LRRC16B	0,089977245	0,955520765
REPS2	0,090047684	0,331251293
WDR13	0,090080313	0,55257125
STAB1	0,090094239	0,768352473
LOC732206	0,090159869	0,155353326
KCNE4	0,090323524	0,618490353
CYP20A1	0,090463804	0,54861556
H2AFY	0,090612081	0,47358451
ChGn	0,090628981	0,427925212
LOC728154	0,09066063	0,279497051
PRDM14	0,090663583	0,291031508
SPRR2B	0,09076182	0,422285605
DIRC1	0,090796082	0,485722162
NCR1	0,09084026	0,332774649
CA1	0,090917137	0,450010753
DND1	0,090937652	0,735891051
PKP3	0,091028711	0,731234386
LRTOMT	0,091039616	0,287378121
ZNF462	0,091385278	0,267017502
LOC730199	0,091433006	0,400284461
CHD8	0,091449717	0,633985891
ZNF256	0,09151086	0,416663199
CYP11A1	0,091566167	0,90144522
TOLLIP	0,091638571	0,303792224
LOC220929	0,091675761	0,182190429
MYBL2	0,091714111	0,740759972
PAK7	0,091812707	0,527262771
DHX37	0,091820026	0,96926012
PFKFB4	0,091976651	0,722942733
OLFML2B	0,091985803	0,398937286
PNPT1	0,092045948	0,516950096
LOC727934	0,092095281	0,84351267
DLX6	0,092105839	0,240203555
IL25	0,092110198	0,852538131
SLC13A2	0,092156873	0,46553869
DACT2	0,092290965	0,880981892

BAGE4	0,092459079	0,649584383
LOC342541	0,092475854	0,949444821
LOC728296	0,092552413	0,528295347
SNAP91	0,092648562	0,339149471
LOC649458	0,092742614	0,845600849
ORC6L	0,092765049	0,860932807
SLCO4A1	0,092805392	0,475265194
LOC643988	0,092831594	0,596290067
FAM26C	0,092920202	0,855059449
TAS2R43	0,092943649	0,260662949
ZNF117	0,092944991	0,409420165
ARMC3	0,092962365	0,586440977
ACP1	0,093015574	0,208088718
LOC202051	0,09308925	0,716496362
RNF44	0,093213805	0,597072436
LISCH7	0,093237759	0,714749874
LOC649930	0,093239253	0,309477918
BRMS1L	0,093562437	0,873256767
CD86	0,093662088	0,656209718
ATPBD3	0,093761608	0,369356959
ETNK1	0,093995666	0,322742749
DDX3X	0,09413882	0,760009165
C16orf54	0,094291175	0,256413638
DRG2	0,094315624	0,270461609
OR56A3	0,094330485	0,847758688
PIK3R3	0,094391949	0,866459984
NEUROD2	0,094470634	0,580794807
FAM53C	0,09466948	0,537782879
CSNK2A1	0,094722379	0,696665166
LOC388279	0,094753978	0,831903896
CD320	0,094756517	0,845241067
ABLIM3	0,094865944	0,761854055
ZNF19	0,09506625	0,21814043
SP1	0,095135353	0,836928451
RND1	0,095188408	0,536899473
CHUK	0,095259887	0,402961365
LOC648596	0,095292415	0,429127882
COX7B	0,095338162	0,795818726
DEFB104A	0,095455477	0,446513982
KRT27	0,095530895	0,297817775
GPR34	0,095533426	0,212641964
TACR1	0,095539459	0,582148276
ACATE2	0,095581097	0,258090026
DDX20	0,095767625	0,364270515
AHI1	0,095847629	0,221411827

LOC440389	0,095903971	0,567282326
PPP2R5E	0,095985876	0,591166346
LOC643790	0,096063969	0,396788382
PNLDC1	0,096365259	0,586368194
FLJ39963	0,096382277	0,623120212
LYRM4	0,096432987	0,403319941
FCF1	0,096721322	0,9067143
EVA1	0,096873168	0,458560508
NAT8	0,096957597	0,691447749
CCDC103	0,097069929	0,246548397
HSH2D	0,097105045	0,500481974
RAB35	0,097151853	0,91599371
LOC648790	0,097180863	0,463843703
SKI	0,097206284	0,384545825
LOC728091	0,097269479	0,157671132
IMP-2	0,09728178	0,912938383
LOC651758	0,097398479	0,322909929
WFDC3	0,097427875	0,248423533
TUSC3	0,097578918	0,302797423
LZTS1	0,097666864	0,605299306
SERPING1	0,09773089	0,931645799
DHRS9	0,097811006	0,475010279
LOC645276	0,097995475	0,739432787
C9orf97	0,098053083	0,228013136
AK5	0,098066905	0,547502152
QSCN6	0,098107929	0,847060266
YIPF1	0,098204007	0,688079429
BANF1	0,098228337	0,458147159
TFEC	0,098257561	0,169418818
LOC642947	0,098333363	0,298887153
KIF4A	0,09842001	0,665147456
CAMK2B	0,098503435	0,863413068
STEAP4	0,098657267	0,626997113
LOC728051	0,098657506	0,173971227
MAGED4B	0,098676412	0,091721761
C1orf124	0,098788447	0,837827721
SIRPB1	0,098916257	0,889985082
ZMPSTE24	0,098936863	0,258770765
SIX5	0,099000395	0,974613477
FNTA	0,099073474	0,51481679
SLC35E3	0,099155	0,201556829
ATP10D	0,099205841	0,938590314
MGC10204	0,099315905	0,154036863
LOC728310	0,099332728	0,638979552
ALPL	0,099400816	0,889747873

CLDN11	0,099439139	0,251197232
CD209	0,099743974	0,449292654
SRC	0,099787527	0,887267509
LOC729671	0,099813432	0,958101491
LOC729978	0,099874571	0,900257394
NQO2	0,099910171	0,306897343
NUP210L	0,099927611	0,388038101
TYK2	0,099995312	0,514744987
LOC727751	0,100037989	0,513202968
LOC728710	0,100093384	0,519317838
LOC730351	0,100112691	0,58494235
USF1	0,100116269	0,903886129
CXCL14	0,100160802	0,439360151
SMPDL3A	0,100207091	0,595529717
CDKN2D	0,10024554	0,693351262
SFRS2	0,100278668	0,448713113
ZNF580	0,100278902	0,460720856
AES	0,100283126	0,344801885
LOC728417	0,100361953	0,410118806
PMAIP1	0,100431933	0,362967304
MTRF1	0,1004516	0,885280903
MAN2C1	0,100544746	0,291855089
LOC389370	0,100636869	0,423270703
LOC727885	0,100855419	0,31976376
PCK2	0,100859511	0,741716356
C14orf178	0,100867461	0,56433005
LOC342933	0,100887047	0,147315713
DBNDD2	0,100945582	0,460090602
KIAA0140	0,101004609	0,91980498
QPCT	0,101052103	0,387528965
PTTG1IP	0,101113628	0,550879875
NFKB1	0,101176039	0,529149178
KIAA1024	0,101252051	0,219023327
PAPSS1	0,101280104	0,988893224
GPM6B	0,101379429	0,267848038
DGKD	0,101521726	0,544599707
SH2D4B	0,101687048	0,510196633
MGC87895	0,10175361	0,738781866
RCC2	0,101777412	0,467626003
TRPM7	0,101793461	0,452904252
TDRD6	0,101937654	0,244417165
ARFIP1	0,101999984	0,365301803
B4GalNac-T3	0,102037462	0,573797497
FKBP6	0,102059004	0,790408912
LOC646030	0,102090237	0,221771079

FCAR	0,102234469	0,760857678
CT45-5	0,102402201	0,450103004
CASKIN1	0,102533226	0,888823482
ZNF513	0,102556829	0,261260164
LOC652900	0,102582643	0,734903511
LOC731334	0,102658226	0,307991268
MCCD1	0,102759891	0,635717189
RAPGEF4	0,102826842	0,948702296
KIAA1026	0,102964793	0,63964061
LOC727959	0,103021333	0,619502486
RUNDC3B	0,103047519	0,410433267
MANBAL	0,103161205	0,260730433
NR6A1	0,103441417	0,789299152
KCNJ10	0,10354634	0,984212695
MYB	0,103594566	0,347521261
ZNF28	0,103645067	0,835561571
DES	0,10366442	0,757137595
CHRNA5	0,103678193	0,489567487
TOR1AIP1	0,103692216	0,459835338
KCNH7	0,103872708	0,668098896
OR5H2	0,103894481	0,924457802
SAG	0,103932917	0,316716703
FJX1	0,104053761	0,254348041
RAB5B	0,104087903	0,303599935
PGP	0,104157069	0,776445357
PCOLN3	0,104207595	0,630792163
DHCR7	0,104278184	0,591744849
GLTPD2	0,104431596	0,449952641
C1orf128	0,104456546	0,297357236
KIF5C	0,104481005	0,477782708
SYNE2	0,104649819	0,556724321
SFRS9	0,104724865	0,556755799
DNAJB8	0,104775365	0,695773146
DKFZP566K0524	0,104841443	0,823606095
TNFRSF13B	0,104852666	0,959520899
LOC729448	0,104915178	0,603078696
ITGB4	0,104995441	0,491971753
OR8J3	0,105076519	0,292499339
LOC643386	0,105267172	0,500031463
SIKE	0,105347813	0,282601539
HYDIN	0,105351361	0,493023686
ITGA6	0,105353424	0,168905848
LOC648581	0,105562786	0,278584556
PABPC1L2B	0,105609435	0,609846487
DCOHM	0,105704789	0,507934835

MMP14	0,106174411	0,449997203
CMTM5	0,106296876	0,318175762
FAM89B	0,106469925	0,792534063
EVC2	0,106507059	0,178342979
HYAL1	0,106541079	0,878539341
CPM	0,106545567	0,974944471
ZNF804B	0,10682495	0,419503114
CACNA1S	0,106842927	0,490211662
LSAMP	0,106857998	0,25102156
STIL	0,106950199	0,056011887
LMAN2	0,107085727	0,788240718
TLR8	0,107139602	0,439233205
LOC728101	0,107145207	0,180709895
KIF1B	0,107320744	0,508800038
COL22A1	0,107380984	0,809003803
ZNF545	0,10739543	0,975263139
SGSH	0,107410626	0,232636954
BPY2	0,107664396	0,617189094
NAALADL2	0,108001178	0,227243002
XPOT	0,108013786	0,621811196
R3HCC1	0,108071651	0,444170594
bCop	0,108165849	0,021153654
COX11	0,108171506	0,264282931
C8orf40	0,108262913	0,659885383
C1orf39	0,10828147	0,931549063
LOC652737	0,108320116	0,348979444
PPARD	0,108385708	0,653006989
FLJ20626	0,108630173	0,683380075
LRSAM1	0,108831335	0,880933762
SIRT5	0,108903047	0,333295901
PSENEN	0,108904208	0,130207838
LAPTM4B	0,108966141	0,588273646
hCG_1981635	0,109159503	0,86303343
SLC39A11	0,109413398	0,812796124
CRYBB1	0,109616955	0,510416414
RBBP8	0,109639518	0,782172685
MRPL12	0,109766919	0,247422697
ZNF496	0,109829976	0,377728219
SPCS1	0,109892571	0,318247887
LOC729268	0,109934299	0,121668777
SNX11	0,110008243	0,326321272
CEP290	0,110079109	0,557135175
U2AF1	0,110137326	0,509677547
LOC732174	0,110178559	0,197812665
FAM23B	0,110258222	0,95564544

C22orf32	0,110412438	0,529793765
CALB1	0,110486533	0,240155124
INCA	0,110539907	0,4450997
DGKB	0,110649471	0,602289582
LOC728883	0,110651189	0,817289614
DEFB119	0,110805417	0,773102069
LOC642991	0,110916533	0,769930039
TTLL4	0,111592623	0,737467672
PADI6	0,111618004	0,552242716
AP1B1	0,111619315	0,92949567
TMEM115	0,111694259	0,593010743
PDCL2	0,111903635	0,898923134
COL15A1	0,111961417	0,53643782
FLJ20674	0,112000236	0,356431521
C3orf44	0,112067993	0,630508894
LOC727923	0,112182228	0,27422
PENK	0,112386847	0,102208713
VPS37C	0,112387921	0,167087007
CSRP2	0,112414306	0,264607208
SLC12A9	0,11244351	0,397193764
FLJ12994	0,112508141	0,237924195
NUP107	0,112582138	0,637117943
SLC31A2	0,112675385	0,211486166
LOC440589	0,112753109	0,787782155
TRIP13	0,112777387	0,662947826
KIAA0861	0,112841373	0,358784008
TBC1D30	0,1129107	0,218333883
GMFB	0,112928776	0,207571327
SGPP2	0,112955293	0,298327634
TPTEps1	0,11299667	0,764081952
EPB41L2	0,11301312	0,723787841
KCNQ3	0,113024597	0,502577259
PARP1	0,11303821	0,967714822
FAM98A	0,113076881	0,319343876
RABEP2	0,11327307	0,375283125
ABCB6	0,113278353	0,621944708
RPL22L1	0,113491325	0,199953587
BPIL1	0,113554436	0,764527266
C10orf27	0,113596986	0,33496642
LOC728866	0,113630276	0,338349111
ADAR	0,113641272	0,296910855
SMCP	0,11367402	0,649046321
METTL8	0,113689088	0,63684906
hCG_1818231	0,113699542	0,557909934
FBN3	0,113793319	0,844200125

FLJ33590	0,113894923	0,661165475
BMPR1A	0,113896834	0,651160891
PARD6A	0,113979175	0,115220432
ITIH4	0,114042601	0,347194507
PNMA6B	0,11415304	0,206021206
ARHGAP6	0,114252055	0,554450056
LOC645645	0,114273062	0,721576912
LOC641933	0,114357298	0,791406357
PAG1	0,114359914	0,792420861
TMEM155	0,114360004	0,200979127
CACNA1I	0,114411149	0,37982204
ISLR	0,114477197	0,571229917
LOC729553	0,114574078	0,18631784
APCDD1L	0,114617714	0,120449525
ECT2	0,114631981	0,369553987
MCM10	0,114747421	0,726178277
GSTO2	0,114944348	0,215963746
PTPLAD2	0,115009207	0,868384924
LOC401052	0,115036074	0,358886075
CCDC124	0,115078288	0,820043365
RAB34	0,115154247	0,594923215
LOC646439	0,115201211	0,667983135
SNX4	0,115238542	0,238004201
FGF5	0,115257829	0,450846352
LOC728017	0,115334245	0,236368709
LOC254559	0,115362253	0,722416797
HDAC2	0,115412973	0,199816081
TG	0,115470547	0,31008421
LOC731863	0,115516256	0,207632307
SLC25A29	0,115636827	0,405984533
NRG1	0,115709115	0,476028776
BTG2	0,11576548	0,38047262
JMJD2D	0,115869754	0,877048367
FLJ13855	0,115890934	0,162950619
LOC732134	0,11590605	0,266012116
KLF1	0,115918811	0,625116717
C16orf78	0,115957347	0,865930935
GPR22	0,116169166	0,913483369
LOC728192	0,116229276	0,390237346
POPDC2	0,116265834	0,839438727
C18orf17	0,116290655	0,515337326
BST1	0,116306221	0,214134244
LOC728246	0,116316132	0,182613851
COG4	0,116336796	0,967667707
CALML4	0,116431875	0,770319595

ZNF3	0,116573312	0,701394768
KRTHA8	0,116648564	0,996530529
CDK5RAP3	0,116730202	0,150824989
LOC401321	0,116921628	0,318848604
GNG12	0,116976406	0,256883235
SAV1	0,117032867	0,391459748
CENTB5	0,117062683	0,907675776
TTC31	0,117069607	0,177177637
LOC730179	0,117125523	0,972977613
NAALADL1	0,117152635	0,371740139
SEC61B	0,117173144	0,705530537
LOC652899	0,117176658	0,757651413
MDS1	0,117252698	0,180557151
NLRP5	0,117387904	0,781214968
ACAS2	0,117425513	0,774033076
ENOPH1	0,117696846	0,227202306
NT5C1A	0,117847982	0,647203723
XPMC2H	0,117876911	0,324750118
RSPO1	0,117929527	0,167691806
LOC441461	0,117933245	0,167135304
BACH1	0,117934516	0,224365981
hCG_1757335	0,118019895	0,228558215
AP4M1	0,118090335	0,310787732
LOC391359	0,118145102	0,962965581
LOC728227	0,118272044	0,50132697
FAM101A	0,118277059	0,682263337
CCDC63	0,118302995	0,833872345
ADM2	0,118416568	0,372575572
HSMDPKIN	0,118431793	0,837494762
tcag7,1272	0,118660197	0,765961681
STRA6	0,118697815	0,633931316
GFAP	0,118738096	0,862857905
FUT10	0,118761969	0,782178305
LOC728861	0,118891803	0,255311042
CDRT15	0,118917038	0,271637717
CHST5	0,119020553	0,362068198
FLJ46836	0,119059401	0,72786135
LCA5L	0,119107907	0,146151939
HOXD8	0,119143321	0,671574641
HTLF	0,119171682	0,586158161
MGC35361	0,119215755	0,385765637
LOC729807	0,119301957	0,246975699
LOC728174	0,119457285	0,13023182
TMUB2	0,119493278	0,524954646
TMPRSS5	0,119564276	0,473415847

PEX19	0,119735445	0,601870841
PSD3	0,120000472	0,911932569
LOC652859	0,120021947	0,232805783
TBC1D16	0,120046221	0,277942954
PLCD1	0,120051259	0,626546638
LOC730446	0,120114166	0,752907564
LOC647521	0,120183916	0,87210283
QKI	0,12026631	0,232028915
BUCS1	0,120269738	0,433693439
YWHAZ	0,120352374	0,756688389
CLCF1	0,120357626	0,881043558
RBMX2	0,120364327	0,405473658
ADAM17	0,120401628	0,942370645
OR2K2	0,120498852	0,463557171
SCD	0,12062062	0,290805399
C1orf183	0,12070409	0,433099965
DAPL1	0,120865039	0,581395473
KCNAB1	0,1210695	0,861684414
GRCA	0,121106733	0,060201448
LOC133789	0,121260516	0,525010502
LOC728972	0,121348603	0,229005373
ASPA	0,121362032	0,631569163
WDR79	0,121614713	0,303144799
LOC728485	0,121652694	0,881762585
MGC87042	0,121677035	0,867752283
AQP11	0,121692333	0,303076215
LOC728904	0,12177158	0,259176805
MAP2K6	0,121888262	0,266128104
SFRS4	0,121930614	0,905699285
ANKRD12	0,121986219	0,725858266
CUL5	0,122078612	0,17507476
SLC25A6	0,122137571	0,987112671
FRMPD2	0,122268173	0,141650593
NLGN4Y	0,122375639	0,493628279
NR4A2	0,122376291	0,375901501
LOC730073	0,122784443	0,350376164
LOC729152	0,122875247	0,61021056
LOC731062	0,122875872	0,293063993
WDR92	0,122959539	0,724594145
CNNM4	0,123094177	0,884921431
KRT17	0,123146725	0,239727266
CHRFAM7A	0,12321378	0,869524521
EXOC6B	0,123267385	0,715975696
NLRP8	0,123407642	0,358430208
LRRC37A3	0,123446831	0,791920903

MATN2	0,123525718	0,266524475
LOC646437	0,123535375	0,21665694
WBSCR16	0,123582436	0,476086807
TPST2	0,123730089	0,397907029
TBX19	0,123802091	0,816160312
OCIAD2	0,123805869	0,161776197
PCDHAC2	0,123986746	0,645157964
POLR3K	0,123992207	0,73666721
MSLN	0,124227592	0,345236389
CCS	0,124296513	0,81524696
STOML1	0,124304751	0,562004545
LOC646520	0,12432278	0,133783815
ZNF286	0,124353734	0,154685673
TMEM62	0,124714964	0,256048752
NUSAP1	0,124784878	0,274005073
LOC728605	0,124894884	0,572910861
STAM2	0,125184995	0,853537215
GTF2H5	0,125217813	0,383619998
KIF26A	0,125322172	0,669229465
ABR	0,125324167	0,923208632
VWCE	0,125477884	0,26582677
DAPP1	0,125520806	0,267093794
CCDC45	0,125584117	0,196642696
MAN2A1	0,125634603	0,29404188
MAP2K7	0,12570724	0,333482918
PNPLA7	0,125809236	0,165318402
LOC643081	0,12581403	0,878741623
FGGY	0,125815259	0,83216486
LTK	0,125829874	0,335873539
IL20RA	0,125980354	0,762125399
CYP2W1	0,126142282	0,457651948
GRCC9	0,126225848	0,327363578
AK3	0,126487511	0,423358364
LIN54	0,126503648	0,641177343
LOC729487	0,126504848	0,25260268
HUMRTVLH3	0,126599789	0,483688986
CXCL6	0,126753984	0,498687119
DKFZp547K1113	0,1267736	0,793328588
ZNF435	0,126809549	0,744655219
RAB3D	0,126851524	0,429396739
EID3	0,126868529	0,985247746
CNN3	0,126933599	0,129178764
PISD	0,126955628	0,604278571
DDO	0,127069479	0,925171849
VIPR1	0,127151773	0,469696683

TCFL4	0,127174959	0,423697667
LOC728360	0,127261871	0,183776138
LOC338805	0,127395823	0,396503692
PSORS1C1	0,12752357	0,271703791
TH	0,127547126	0,419174377
C10orf26	0,12756305	0,46125071
ZHX3	0,127624353	0,698472067
FASTK	0,127675569	0,454366476
ARS2	0,127793915	0,317631965
GJB7	0,127809403	0,23310624
NHS	0,127875309	0,420413644
C20orf195	0,127954779	0,637436759
RLBP1	0,127970807	0,987583145
MGC11349	0,128060494	0,75321737
MSP	0,128138888	0,804442868
RNF134	0,128225591	0,830586078
LOC730510	0,128233471	0,809537088
TAS2R45	0,128260875	0,524679506
FAM60A	0,128342083	0,467231891
AKAP7	0,128497066	0,36032238
TPP2	0,128631221	0,698754782
LOC343384	0,12867278	0,108284357
NEDL1	0,128684774	0,88772728
LOC402377	0,128756242	0,827683318
SBNO1	0,128979424	0,360099123
ENTH	0,129111136	0,794488661
SLAMF8	0,129125879	0,515433429
LOC729725	0,129155555	0,381241465
LOC645605	0,129194022	0,523487637
CHRNB2	0,129224102	0,290701771
WDR40B	0,129410964	0,910785082
ZCSL3	0,129418511	0,314628147
C12orf61	0,129447726	0,653163677
LOC728220	0,129449541	0,430160016
B3GALT6	0,12945822	0,912878859
LOC732185	0,129473382	0,971946897
LOC653428	0,129483259	0,76825417
LOC731286	0,129616512	0,53845421
LOC730862	0,129731524	0,574583013
C10orf82	0,129762381	0,848158559
MEMO1	0,12978633	0,151608407
PCDHA8	0,129851721	0,819381453
ARHGAP23	0,12997999	0,237516686
LBH	0,130028881	0,62666845
FLJ43663	0,130037251	0,547400645

LOC732058	0,130138461	0,866201472
CD53	0,130450061	0,315194673
GSPT1	0,130462274	0,506554984
PCNXL2	0,130601797	0,572984084
DXYS155E	0,130639001	0,998651248
RUNX1	0,130746897	0,930565622
BIA2	0,130764904	0,29553954
SNX29	0,130860835	0,239632566
LOC375328	0,130896679	0,696810781
KCNJ3	0,130898943	0,974720668
ANKRD13C	0,131046011	0,444427313
HAO1	0,13105731	0,833175721
KIAA1333	0,131089411	0,320308401
ANKRD13B	0,131101841	0,291808402
PRDX4	0,131116612	0,906430642
LOC390956	0,131168132	0,347884615
PDK3	0,131265508	0,731363368
SPACA4	0,131342307	0,611574673
STK36	0,131390726	0,129662805
ALDH3A1	0,131398385	0,210968138
LOC728397	0,131481639	0,34634872
CDC73	0,131573561	0,773557663
FIBP	0,131745209	0,656606203
CSF1R	0,131751288	0,795282747
ZNF2	0,131772017	0,316887582
DENR	0,131947556	0,926856951
PNMA5	0,132034391	0,749903132
LOC652860	0,132092855	0,984973155
OCLN	0,132116667	0,804592311
UBL4A	0,132133568	0,743692684
RNASE6	0,132288529	0,510403598
KIAA0644	0,13236021	0,400446309
RAET1G	0,132600686	0,996689541
TP53INP1	0,132643467	0,160370732
KLK12	0,132760925	0,627956722
SH3BP5L	0,132767314	0,872942059
GALNT10	0,132768682	0,798899117
LOC728714	0,132778443	0,167351226
HSPBP1	0,132835686	0,188956054
CPOX	0,132887338	0,566351201
LOC652388	0,132952604	0,558853022
hCG_1651160	0,132961203	0,608047598
C14orf147	0,13296479	0,222840909
TMEM9B	0,133020875	0,617232392
LOC730430	0,133103317	0,446571663

LOC648415	0,133175099	0,220799153
ATP2C1	0,133221191	0,570411862
TRAF1	0,133241771	0,979102289
ACVR1C	0,133252892	0,688606791
DHX36	0,133396151	0,928385118
CTNNA3	0,133406857	0,748793984
ADAM30	0,133481108	0,795291546
UEV3	0,133523569	0,103689998
POLR2B	0,133579639	0,921258836
PAPPA2	0,133689719	0,243964624
GP9	0,133748265	0,460157512
KIF24	0,133772601	0,80206826
RPS4X	0,133834443	0,619747227
HIST1H2BK	0,133862797	0,62261611
C4orf22	0,133892828	0,09773995
PHOX2B	0,134015415	0,753731666
TGM7	0,134040132	0,980469079
MRS2L	0,134138414	0,819236522
LOC729933	0,134296925	0,617327311
LOC731933	0,134369828	0,894104261
G6PC2	0,134407355	0,095602385
C12orf44	0,134410385	0,886979192
KIAA0669	0,134437636	0,281793422
STOX2	0,134576285	0,443642262
TST	0,13466228	0,52537951
C9orf99	0,134678932	0,589773649
SNX14	0,134892234	0,248631729
LOC644248	0,134955412	0,356767449
LOC730168	0,135030796	0,222667512
LOC283663	0,135072723	0,287630517
METAP1	0,135117581	0,406139757
JAG2	0,135359223	0,831888794
HPSE2	0,135433969	0,25689881
LOC728903	0,135554408	0,418125429
TMEM200B	0,135642719	0,526065459
SCGB1C1	0,135691796	0,144310237
LOC646272	0,135764433	0,233353397
METRNL	0,135818136	0,179859446
FBP2	0,135863469	0,380035459
HNRPU	0,135956074	0,368732123
FUCA1	0,135962243	0,300779753
LOC646569	0,136135665	0,400261669
DHTKD1	0,136167366	0,648167112
FSCN1	0,136276388	0,924653394
ZNF225	0,13639406	0,498977796

KCNJ5	0,136482581	0,607357696
LOC732380	0,136523908	0,798704197
ANKRD40	0,136534186	0,32226117
LOC641971	0,136770137	0,625348036
C14orf131	0,136797874	0,758028661
PSMD14	0,136836263	0,694325266
LOC646762	0,136892913	0,637127545
FBXO27	0,137309903	0,962141949
KATNB1	0,137585087	0,517618541
LOX	0,137585305	0,161771927
H2AFZ	0,137869548	0,538272758
GOLT1B	0,137923084	0,476055125
SLICK	0,137931379	0,236594886
LOC644969	0,138050095	0,825326088
VPREB1	0,138150795	0,764886626
PITPNC1	0,138333544	0,450699722
hCG_1992287	0,138366924	0,87265226
F5	0,138638127	0,121963648
LOC730226	0,138693342	0,363913365
LOC388885	0,138747352	0,54797291
DEC1	0,138767779	0,362688505
hCG_1644239	0,138834406	0,845162046
LOC730658	0,138840568	0,44888235
TNFRSF4	0,139080716	0,674074487
CYP4A11	0,139113267	0,296299696
MYST4	0,139201146	0,640954423
ESR2	0,139241902	0,990188875
LOC132430	0,139348473	0,737058115
C3orf56	0,139391911	0,220363739
GPRIN3	0,139406538	0,210789595
LYZL1	0,139487206	0,552389099
TCOF1	0,139491923	0,575756981
KAZALD1	0,139501986	0,699498554
CBFB	0,13952988	0,178002005
TOR1AIP2	0,139587533	0,470959069
PEX6	0,139602364	0,470491155
LOC284890	0,139603149	0,299355495
WDR51A	0,139651066	0,82952393
GNAT3	0,13965353	0,803978996
KIAA1524	0,139663181	0,118038348
CDKAL1	0,139906798	0,771050251
LAMC3	0,140005452	0,218658023
LOC648170	0,140117284	0,737722409
CCDC80	0,140212749	0,337888178
SLFN12L	0,140293083	0,186386931

SNX26	0,140346217	0,936322641
PIGK	0,140399373	0,426373904
DLAT	0,140409668	0,838220418
FBXO8	0,140422126	0,880841099
PEX11B	0,14042459	0,97731896
FHIT	0,140500702	0,799473094
N2N	0,140539453	0,573365266
MMD2	0,140592734	0,444122592
IFNW1	0,14063593	0,813580103
ZNF362	0,140791111	0,788500455
FABP5	0,140820535	0,39814817
C17orf46	0,140835028	0,856249337
RCSD1	0,141039034	0,347207995
C13orf33	0,141265141	0,885251581
FLJ37464	0,141299882	0,229972014
KIAA1542	0,141532051	0,156470514
SGSM3	0,141780978	0,38749343
LOC729788	0,142333524	0,084365149
FAM19A4	0,142334227	0,133073189
RBM4	0,142360225	0,962050289
LOC730615	0,142412803	0,135512478
C19orf23	0,142485259	0,267095977
CTRB2	0,142606817	0,538243716
CCL21	0,142651682	0,381407084
LOC647024	0,142678239	0,253753698
TANC2	0,142832604	0,501049388
RPL36AL	0,142837888	0,396042438
LOC730738	0,142928955	0,085277909
CHC1L	0,142935191	0,380020259
TMC7	0,142987017	0,515761505
NDEL1	0,143029885	0,193617428
LOC643972	0,14304297	0,085289888
LOC652647	0,143149666	0,100834706
VANGL1	0,143528389	0,259921537
DHRS7	0,143555595	0,432548243
TCF7	0,143707808	0,187770484
ANAPC11	0,143746883	0,220388159
LOC348262	0,14386994	0,630659837
GIMAP2	0,144170766	0,195316024
TMSB10	0,144243079	0,09043621
LOC730926	0,144390457	0,317678284
SLIT3	0,144657871	0,158186708
FBXW9	0,144661857	0,262559944
C9orf142	0,144760728	0,800675166
KIAA1751	0,14495627	0,243593035

XPA	0,14507439	0,848331463
LOC652095	0,145130105	0,517896728
EIF4B	0,145240104	0,584428591
FBXL21	0,145347479	0,686956821
C14orf25	0,145383143	0,166502954
POLB	0,145412506	0,715648505
OR10J5	0,145498511	0,853953229
OR8H1	0,145510207	0,160827045
C22orf26	0,145655301	0,956332463
LIMS1	0,145787188	0,328735766
SLC35B3	0,145844565	0,304650457
IGSF9	0,145907656	0,443406284
MTAP	0,145915071	0,166002367
FAM127C	0,145993221	0,172276146
CENTA2	0,146257487	0,79347606
TSGA10	0,146377462	0,8687726
CHRD	0,146704289	0,628995493
CYP11B1	0,146743499	0,602971301
LOC731832	0,146788335	0,750268419
MRPS25	0,14679258	0,191379132
PBP	0,146817243	0,204199709
POSTN	0,146839122	0,399032582
BCL7A	0,146870418	0,969933788
S100B	0,14687276	0,957185494
SIDT2	0,146944918	0,635662631
FAM101B	0,146945399	0,234076248
UGT1A7	0,146952175	0,548582126
C4orf17	0,146976294	0,137791353
LUZP1	0,147004322	0,415451376
CAPON	0,147075523	0,805726714
FRK	0,147087254	0,898482728
NOL12	0,147170528	0,552162358
DNM3	0,147178254	0,684391186
ERCC3	0,147235253	0,722275261
C6orf10	0,147239354	0,564788374
EMD	0,14730456	0,600798756
PLXNA2	0,147374621	0,522220729
PPP1CC	0,147532112	0,79735475
PMS2	0,147546104	0,581168797
RNF4	0,147554888	0,155207497
RDH10	0,147632746	0,497789639
ZNF527	0,147685381	0,330826173
RAX	0,147914762	0,814444884
KRTAP17-1	0,147997899	0,253118734
PRAME	0,147998243	0,607334026

LOC651091	0,148003808	0,424016084
hCG_1643692	0,148178587	0,690268739
WDFY1	0,148222117	0,658538765
CALCR	0,148293636	0,328803313
BPNT1	0,148304215	0,517374248
KIAA0146	0,14831745	0,948977203
C1orf110	0,148485321	0,324084207
RHCG	0,148605635	0,571212436
VPS41	0,148774392	0,194392718
P2RY10	0,148882449	0,186071513
OR52N4	0,148921652	0,655616676
ARID3A	0,148935318	0,415118151
OR2M2	0,149041256	0,60582263
RWDD3	0,149320417	0,313538122
XRRA1	0,14932358	0,31138953
LOC650724	0,149383365	0,623746985
RBM24	0,14950539	0,256270834
PLCB2	0,149635304	0,676117714
MFSD6	0,149656409	0,176219336
LOC283143	0,149874906	0,723783544
ASTN1	0,149985511	0,462424401
SWAP70	0,149990462	0,548624572
PPP3CB	0,150181525	0,792336528
TMSB4X	0,150186348	0,710657914
LOC730227	0,150270206	0,231556304
RAD23A	0,15028425	0,441258217
CRLF3	0,150314035	0,192971518
KLRC2	0,150343991	0,863963757
HERC5	0,150419599	0,303451128
C13orf26	0,150451917	0,426010802
HGD	0,150679657	0,984137275
CR2	0,150751152	0,417395052
LOC730579	0,150755168	0,245785602
SLC4A8	0,150919917	0,934480768
MAMLD1	0,15106063	0,575130613
GRM2	0,151139395	0,50067134
LOC727976	0,151146329	0,233199826
LOC730922	0,151156692	0,531515328
FAM113A	0,151349999	0,524890953
C20orf177	0,151384833	0,53159223
CAPN1	0,151469535	0,614392218
PREX1	0,151558098	0,559590463
EIF4E	0,151596937	0,531462695
HMGB1	0,151650602	0,303381184
SLC9A8	0,151750355	0,427072733

LRRIQ4	0,151871874	0,194492083
SNX13	0,151872112	0,797444169
FAM70B	0,151890754	0,578821884
EXO1	0,15215015	0,909427466
FLJ12242	0,152174895	0,210360792
C19orf44	0,152490139	0,698520098
LOC729082	0,152637478	0,334515537
PPP1R13L	0,152652665	0,539132827
ACR	0,152699171	0,303170561
MPPED1	0,152785099	0,393801756
C10orf128	0,15281289	0,537242302
PSME2	0,152836572	0,421362771
LOC649267	0,152847337	0,761335915
CEP57	0,152890908	0,976374578
KCNK13	0,15312383	0,843618036
LOC647850	0,15337446	0,688139195
WDR37	0,153472844	0,230921149
PYHIN1	0,153587801	0,196570065
FRAT2	0,153647898	0,161100627
LYRM2	0,15366505	0,786512644
CRYBB2	0,153760155	0,31523878
UBE2E2	0,154035312	0,26233409
NRP1	0,15409463	0,803108915
FAM166A	0,154136337	0,295499114
PIK3C2B	0,154196401	0,763308165
KIAA1407	0,15449858	0,921671983
FLJ11164	0,154589797	0,524684508
LAT2	0,154603499	0,183691663
LOC731137	0,154613299	0,634284015
WDR1	0,154681331	0,549853811
C9orf85	0,154688301	0,621973126
LOC730378	0,154738001	0,36828637
REEP3	0,154800322	0,297627787
EDNRB	0,15482166	0,309024514
MGC12966	0,154869339	0,558028847
UROS	0,155034542	0,235134037
FLJ32191	0,155091386	0,314516166
N4BP2	0,155305607	0,634677466
DOT1L	0,15545832	0,352610313
SV2A	0,155466234	0,479492992
HUMMLC2B	0,155472052	0,876340152
CLIC6	0,155490312	0,32943166
DSCAM	0,155777948	0,854733516
LOC146439	0,155902696	0,99335939
ABHD14A	0,15593908	0,122918627

OR5D16	0,156027089	0,480546385
TULP2	0,156150813	0,555388052
TRIM39	0,156277256	0,738015831
C6orf222	0,156355293	0,284810033
RECQL	0,156471526	0,314031092
STX7	0,156753072	0,230521984
CACNG2	0,15682795	0,07519224
LOC284801	0,156874316	0,751083874
DLEU7	0,157006491	0,806534632
LOC729243	0,15703745	0,769299361
ERP70	0,15712417	0,476752883
OR2C1	0,157156836	0,347856286
ANTXR2	0,157178249	0,654213217
LOC729190	0,157328943	0,207360929
ALCAT1	0,157374428	0,409754886
LOC643561	0,157401786	0,166539776
SLC9A7	0,157513312	0,130469883
TMEM132E	0,157622821	0,350446194
SSX1	0,15766063	0,702222369
DIP2C	0,157724588	0,899138695
LOC729182	0,157733399	0,244006332
C4orf19	0,157793627	0,69593673
ORMDL1	0,157828466	0,812224237
PTGIS	0,157983741	0,414638156
LOC729465	0,157991616	0,237067827
MAP2K1IP1	0,158015491	0,096268075
CTNND2	0,158141817	0,339532219
B3GALT2	0,158229378	0,738121117
WNT4	0,158465712	0,182180077
HPR	0,158650639	0,191968921
SAFB	0,158738141	0,485405423
LOC729980	0,158889463	0,602247291
CXCL13	0,158944954	0,249315291
TRIM56	0,159104224	0,627209796
IL21	0,159340672	0,933692688
STAP2	0,15939116	0,877908052
GRM6	0,15941932	0,981334931
SMC1L2	0,15951798	0,146759505
PIK3CA	0,159703724	0,490494723
CDCA5	0,159712231	NA
LOC440386	0,159715704	0,325831858
NIP7	0,159724058	0,59828376
RGPD2	0,159795228	0,14393865
LOC284100	0,15979595	0,290882146
KLHL24	0,159866267	0,116711758

LOC730192	0,159993822	0,842530874
SHANK1	0,160129423	0,82687252
MTMR1	0,16014117	0,208074407
FYCO1	0,160169179	0,951880126
COX5B	0,160214	0,548688661
NDUFA1	0,160341532	0,851389279
STAU2	0,160342835	0,812455292
LOC728420	0,160561278	0,711437906
LOC642197	0,160757582	0,640491218
MAGEA9	0,160758832	0,649375363
LOC728147	0,160777826	0,503180359
OR52H1	0,160857003	0,912169292
POLR1B	0,160997298	0,736619335
HARS2	0,161048516	0,576384163
PTGFR	0,161170326	0,408384976
HCG9	0,161223193	0,217145649
LRCH2	0,161312095	0,217681292
IL22	0,161447228	0,027179753
MGC10715	0,161591781	0,715514126
MYO3A	0,161617955	0,399290615
CCDC51	0,161712267	0,343024763
CACNG4	0,161748466	0,404266684
ABCA2	0,161823684	0,563704155
PRX	0,161863314	0,737542404
TADA2L	0,162011151	0,326501916
LY6G6C	0,16203788	0,217300567
SYNPO2	0,162057304	0,626883711
SLC14A1	0,162250943	0,477088067
EFCAB5	0,162275358	0,823448542
C21orf29	0,162295324	0,456790039
ROR2	0,162424451	0,929802175
OR1C1	0,162459632	0,13334181
ANXA8	0,162534474	0,878237112
GPRC5B	0,162686615	0,384595592
FLJ45717	0,16271447	0,098578407
ARG2	0,162794756	0,66433485
PLD1	0,162807267	0,20486882
SCHIP1	0,162824817	0,409137114
TAGLN3	0,162828178	0,254185438
U2AF2	0,162853333	0,889850099
GUCY1B2	0,162889735	0,653302783
SH3GLB1	0,162903649	0,13585737
ENTPD1	0,16302162	0,408521071
MYST2	0,163030207	0,202851884
MYCL1	0,163135918	0,238933258

SIGLEC5	0,163281568	0,471998706
KRT73	0,16351229	0,357659754
UGT2B17	0,163767629	0,608101852
CBFA2T2	0,163926957	0,926037809
SS18L1	0,163981355	0,941735774
CCDC126	0,163986547	0,821990126
LOC727822	0,164032529	0,972108771
CDRT1	0,164085554	0,13868454
PCDHGB3	0,16423839	0,972033328
CAPN11	0,164478622	0,79291129
DACT1	0,164542263	0,571563705
NCLN	0,164651514	0,478114909
OR2H1	0,164746475	0,749402785
PARP4	0,164852836	0,370670823
PFN2	0,164905248	0,588002817
LOC730528	0,165092488	0,728375169
PTBP1	0,165485824	0,300112347
LOC646951	0,165571942	0,253142685
RILP	0,1656091	0,629320491
TAC4	0,165876708	0,620463992
LOC729289	0,165883204	0,747343757
LOC727786	0,165957453	0,284709824
GDF7	0,165975662	0,399672229
LASS5	0,166036601	0,261605026
SCNN1A	0,166088889	0,612841911
ADAMTSL4	0,166157863	0,316102425
DAZAP2	0,166363932	0,200809432
LOC730195	0,166391336	0,716927221
MFHAS1	0,166521238	0,560194174
HIP1	0,166732964	0,279620952
LOC645015	0,166812748	0,443397213
PC	0,166814424	0,263199732
GPR148	0,167067781	0,537572202
EIF2AK4	0,167292461	0,215579053
BRCA1	0,16732186	0,284783887
NGDN	0,167422424	0,386172645
MPP5	0,167555628	0,20846417
SLC9A6	0,167610762	0,634852816
TMEM121	0,167647645	0,180949321
TIMM22	0,167648958	0,697054465
NR2C2AP	0,167684976	0,288722341
LMNB1	0,168148897	0,65774701
LOC441511	0,168194542	0,356599442
UBTD1	0,168262206	0,700737294
LOC375295	0,168360959	0,65978453

GPR37	0,168457749	0,389621358
C3orf20	0,168490575	0,576034989
GLO1	0,16898836	0,533709202
WBP2NL	0,168990996	0,949281285
CPS1	0,169089788	0,430634457
AGXT	0,169267675	0,60997117
SLFN5	0,169299872	0,434923522
DKFZp564I1922	0,169466797	0,179322468
APOE	0,16954124	0,865444551
C12orf64	0,169599026	0,243361324
ZNF227	0,169731255	0,707702915
FAM80A	0,169860324	0,193943875
C14orf39	0,169922122	0,369647731
CD1A	0,169993444	0,338143257
XPC	0,170101464	0,266129706
CHAC2	0,170216317	0,410256788
HAND1	0,170333163	0,238017057
PHACTR3	0,170415548	0,261040052
LCN6	0,170627813	0,418008531
NDP	0,170770736	0,237391623
FAM82B	0,170889024	0,802598821
LRCH3	0,170902081	0,468565445
FLVCR	0,170933137	0,900293098
PDYN	0,171046991	0,099610178
ECHDC3	0,171065515	0,495119239
LOC645253	0,171077755	0,323505882
FN3KRP	0,171197715	0,32170556
TPRG1	0,171244641	0,687458924
OR6T1	0,17128118	0,634241858
WASF2	0,17135093	0,361479021
LOC440577	0,171365211	0,112121264
UBQLN2	0,171426726	0,385092959
DJ971N18,2	0,171535064	0,305382978
LOC23117	0,171539327	0,845468937
CLDN5	0,171742356	0,325225091
TEX28	0,172064603	0,66490853
S100A16	0,172125299	0,561602606
FLJ23548	0,172131578	0,992379824
HSPA8	0,172208678	0,556023707
PCDHGC4	0,172285038	0,326843872
TM4SF20	0,172298127	0,186498109
HSD3B7	0,172361042	0,674889185
hCG_1640171	0,172437539	0,885600622
TECTA	0,172530764	0,632595983
USP22	0,172532113	0,307700999

NCF4	0,17283023	0,673202379
CGI-69	0,172852824	0,510119242
LOC644976	0,173067506	0,213899776
USP37	0,173118894	0,547689391
ABCC11	0,173135394	0,426587299
BLR1	0,173205475	0,156983161
GTSF1	0,173256032	0,669635885
EPS15	0,17326309	0,349793724
DULLARD	0,173292359	0,5
KCNA6	0,173423306	0,360191821
IGF1R	0,173474641	0,332862236
DECR2	0,173508872	0,825116403
SPANXN1	0,173632126	0,446016585
FUT1	0,173643385	0,302024869
H-plk	0,173721417	0,368530906
DNAJB7	0,173803348	0,208424879
CCDC109B	0,173812645	0,58686376
FAM44A	0,173941869	0,303077109
DLC1	0,173982564	0,838447051
OR7D4	0,174056366	0,73376711
RPESP	0,174066962	0,314092768
LOC728969	0,174302112	0,429057834
DMKN	0,174435054	0,153869052
USP12	0,174562314	0,188780788
C17orf56	0,17458168	0,197685732
PROC	0,174721653	0,322958096
SLC37A4	0,174826107	0,519767668
LHX4	0,174875198	0,447334771
C9orf40	0,174898472	0,135854329
CELSR2	0,175066312	0,150728049
ARMC1	0,175110942	0,285174496
PEX5	0,175147604	0,968379594
SETDB2	0,175155999	0,355727918
STRN4	0,1753219	0,797154235
GABRG2	0,175341663	0,606224823
NOXO1	0,175401716	0,37303788
P2RX4	0,175427812	0,157757403
LOC730244	0,175439778	0,553917156
FAM29A	0,17555808	0,302033002
HYPB	0,175605303	0,64538452
RBM6	0,175649719	0,29112745
LOC729806	0,175653977	0,339879356
LOC390352	0,175689921	0,565300099
CCDC68	0,175855948	0,559783926
LOC729889	0,175905343	0,078092488

TP53	0,175962507	0,714510603
RPAP1	0,175967694	0,132605458
PLUNC	0,176028745	0,400205436
LOC728083	0,176032456	0,639129976
SYT15	0,176103395	0,370022268
SLC22A9	0,176161617	0,356464539
LOC652759	0,176236831	0,774288102
GPIHBP1	0,176273797	0,471522191
MTF1	0,176386449	0,479212985
LOC652134	0,176421541	0,750485706
LOC283481	0,176477842	0,217731562
TNIP2	0,176511053	0,526142391
LOC389300	0,176569338	0,197809567
IGJ	0,17663782	0,186380449
C1orf54	0,176667215	0,630366758
INS	0,176728219	0,202523714
PDLIM3	0,176840971	0,249164399
JAKMIP2	0,177007962	0,405939394
MPZL3	0,177189948	0,20094592
C10orf65	0,177299822	0,572330894
LOC728869	0,177309537	0,662205769
CEACAM3	0,177330086	0,998138142
LOC643749	0,177368698	0,935089724
TSP50	0,177388347	0,329684485
MURC	0,177558294	0,474803863
KIR2DS4	0,177633195	0,302730565
HS6ST1	0,177686338	0,303144469
LOC730465	0,177735357	0,384200694
PSMC1	0,177743374	0,291913818
LOC652148	0,1779461	0,970467268
FXR1	0,177968285	0,190859413
LYG2	0,17808428	0,389099232
LOC727798	0,178347777	0,460311159
LOC732416	0,178542118	0,503292079
SIRT1	0,17857254	0,769000523
PPP2R3A	0,178573248	0,792911746
RTN2	0,178763371	0,265869821
OR51B6	0,178837269	0,942875287
APOL5	0,178935895	0,544159767
KIAA1199	0,179019721	0,336724114
CHRM2	0,179035696	0,904135766
FBXO47	0,179058543	0,152624506
TNFRSF11B	0,179454613	0,355702303
HIST1H2BC	0,179514389	0,284738875
KIF5A	0,179547947	0,379108715

SLC17A5	0,179601811	0,214992058
CACNA1E	0,179820452	0,90236985
P2RY14	0,179922454	0,61938913
LTC4S	0,180107438	0,054849217
FSHR	0,180116435	0,563429029
PACS2	0,180117872	0,559831508
GSTM5	0,180310558	0,282610008
TJP2	0,180432867	0,229666087
CCL8	0,180457752	0,30992416
SIAT7E	0,180613068	0,16641754
LOC727999	0,18066893	0,243590626
TSPAN2	0,180753016	0,526924814
SOX5	0,180792635	0,282491439
TCF7L1	0,181071432	0,553904012
LOC729144	0,181124075	0,391899696
LOC645225	0,181215294	0,814264878
ATP5F1	0,181271505	0,452691032
MAP3K2	0,181347357	0,174138455
SEMA7A	0,181733564	0,827579967
RNF129	0,181853892	0,993686711
EIF2S2	0,182007823	0,660089005
LOC729388	0,182027536	0,172651504
FAM27L	0,182161173	0,208630182
HTN1	0,18218326	0,290484165
PEA15	0,182224867	0,641955661
DCDC2B	0,182268813	0,215311975
C1orf50	0,182449355	0,802787566
FILIP1L	0,182776545	0,06304125
NFASC	0,182787561	0,864143519
GEMIN4	0,182852139	0,229985455
SLC25A10	0,182858303	0,634265533
LOC388849	0,18290689	0,341413654
DKFZP434L1435	0,183017361	0,555123366
GRIK4	0,183064812	0,206001831
ECHDC1	0,183304214	0,379483655
C7orf64	0,183361794	0,606161242
PTOV1	0,183393452	0,161848984
FUT7	0,183487555	0,645319748
ME1	0,183672556	0,189687408
TMEM183B	0,183680334	0,190179355
TIRAP	0,183734893	0,440836474
ORAOV1	0,183745556	0,245671493
MAT2A	0,184030629	0,889847101
BAG2	0,184051602	0,382049931
DENND4A	0,184125488	0,24208949

ADNP2	0,184143153	0,120613729
C6orf72	0,184143768	0,282654161
LOC729104	0,1841712	0,403726222
LOC729041	0,18420079	0,647328867
SRP9	0,184235179	0,322543765
SAMD7	0,184274829	0,571263194
FBXO39	0,184435137	0,57622976
EHD1	0,184509971	0,719260116
NXF2	0,184514777	0,367750167
FAM177A1	0,184607244	0,214597782
LOC729244	0,184832971	0,190864858
EMP2	0,185123724	0,280948308
LOC729464	0,185499685	0,668517566
LOC339674	0,185555731	0,647886158
LOC347411	0,185653174	0,522606966
HIST1H2AH	0,185871725	0,633845734
LOC728459	0,185902451	0,305510423
GPR154	0,185926471	0,309788585
SEZ6L2	0,186553897	0,606950943
GPR54	0,186571729	0,398553264
ELF3	0,1865987	0,454691517
CAPN9	0,186627208	0,311602114
FZD8	0,186729401	0,918704676
FRG2C	0,186836346	0,254738445
KRTHA3A	0,186872898	0,07125036
ZNF767	0,187020405	0,667084012
IRF3	0,187109074	0,417866655
SLC41A3	0,187227966	0,169489919
CD72	0,187251853	0,200439459
PNMAL1	0,187454805	0,235824987
RNF126	0,1877187	0,585360134
ELP2	0,18798982	0,23441561
MGC11134	0,187991272	0,361306819
C7	0,188001536	0,871452331
LOC389654	0,188242754	0,789974725
LOC730112	0,188269496	0,95650428
FAM96B	0,1883474	0,216816096
OR4A15	0,188356632	0,634452701
LOC63920	0,188369949	0,093474668
ADCY9	0,188446511	0,298821953
FYTTD1	0,188464206	0,543108797
TCEAL4	0,188595115	0,366548522
TTC28	0,188624806	0,65344119
HIPK2	0,188712341	0,228867575
ACYP1	0,188760828	0,301830041

GOT1	0,188857864	0,646597682
BTBD8	0,188922895	0,174277397
ADAMTS13	0,189051856	0,21559864
WNT8B	0,189057248	0,743022654
FLJ33544	0,189356597	0,308520138
NPTXR	0,189384498	0,78676335
ATP13A	0,189421602	0,209169702
TRIM21	0,189455231	0,428051763
LOC729002	0,189589326	0,126703312
SH2B3	0,189629779	0,274983732
P66beta	0,189645674	0,142266024
KIAA0010	0,189663703	0,513209793
PVRL3	0,189682153	0,791194715
LOC646649	0,189931673	0,98585096
IGSF2	0,190023163	0,852339138
RAMP3	0,190072781	0,307026692
RAP2A	0,19020929	0,226366132
NEUROG3	0,190432103	0,396175436
ZNF606	0,190664813	0,17661388
LOC643547	0,190668235	0,264841196
LOC647250	0,190919605	0,674088369
PPP2R2D	0,191014423	0,563514481
TBXAS1	0,191025567	0,572421844
P2RY4	0,191048318	0,278478129
FCN2	0,19114519	0,843426352
SLC35C1	0,191323887	0,164679555
HAND2	0,191532246	0,46782828
LOC644500	0,191575215	0,599993566
LEPR	0,191935436	0,51833939
PPP1R1B	0,192028938	0,528147459
C10orf136	0,192036847	0,240474234
hCG_1815491	0,192085552	0,812476561
FECH	0,192127463	0,42478107
CNRIP1	0,19215795	0,28959137
UCKL1	0,192206939	0,540179132
GLIS3	0,192363772	0,486048287
C15orf23	0,192443674	0,4838803
SQLE	0,192725232	0,212599015
TNRC18	0,192762516	0,948069404
LRRC67	0,192828754	0,059785824
HTR2C	0,193035947	0,362946133
GABPB2	0,193284733	0,122645233
UNC45A	0,193393831	0,183660207
LOC123855	0,193425106	0,210112289
DPT	0,193528473	0,414359691

RASA1	0,193770533	0,677794473
DLGAP3	0,193812042	0,966054181
OSBPL6	0,193897056	0,757548286
LOC646667	0,193900455	0,151175414
PIK3R4	0,194066314	0,264174485
G22P1	0,194127314	0,261686711
AKAP3	0,194364786	0,7723571
FLJ90724	0,194530216	0,350210964
LOXL4	0,194634595	0,217221556
LOC643637	0,194719767	0,805256442
OR4C12	0,194810743	0,160742664
LOC649698	0,19483296	0,401627495
LOC730651	0,194891869	0,62156827
DHFR	0,194901641	0,136128502
RNF208	0,194963846	0,806389159
HPS5	0,194987478	0,242026938
C6orf211	0,194989092	0,630713562
FLJ31052	0,195092451	0,807641752
ZADH2	0,195195712	0,311912583
ARL17	0,195303907	0,116702426
ATF5	0,195419443	0,529677158
NBPF3	0,195486889	0,496756529
ZMYND11	0,195659926	0,461008199
PSMD7	0,195709915	0,373985335
OR14C36	0,19574827	0,225054031
CTSS	0,195973676	0,441657873
ASB18	0,195988352	0,545480209
DIXDC1	0,196073796	0,637854811
NDUFA7	0,19616222	0,226341263
CCKBR	0,196567166	0,560087657
LOC728839	0,196629271	0,753242522
BMS1	0,196650346	0,20131255
LOC201725	0,196782858	0,4549915
CORO1A	0,196841474	0,407616938
C6orf129	0,197068065	0,89447801
ICT1	0,197090081	0,2752402
C12orf49	0,197117018	0,299949658
TUBGCP6	0,197193876	0,489160525
RGS22	0,197210818	0,671641033
RSRC2	0,197254357	0,785282429
SSR2	0,197277132	0,343585458
TTLL3	0,197304005	0,269518347
AP2B1	0,197336596	0,311979667
CBX5	0,197599419	0,166153484
GPC2	0,197650158	0,287676371

TMEM184B	0,197719478	0,931324667
CLIC2	0,197813133	0,373444553
C2orf61	0,197827214	0,218177773
TGFB1I4	0,197874178	0,742453835
TLP19	0,19806148	0,285844634
GNB1	0,198148257	0,625734954
MCM3	0,19821023	0,618474495
NECAB3	0,198257614	0,270438646
MT	0,198261802	0,36950042
LOC643042	0,198376906	0,550476846
NFE2L3	0,198639709	0,920589662
ZNF395	0,198724777	0,297284426
LOC649896	0,19873409	0,233366808
PTGER2	0,198916881	0,350723245
GCGR	0,198963037	0,466754404
SEMA3B	0,199009469	0,150131802
LOC651501	0,199117292	0,703524095
EBF4	0,199160124	0,24308876
XPO5	0,199316867	0,100799376
AMY2B	0,199381213	0,719045777
LOC729763	0,199456716	0,348322513
LOC729197	0,199497642	0,678185949
IRS1	0,199503251	0,160522332
ESPNL	0,199540307	0,113218
FPRL2	0,199588061	0,832220047
Cab45	0,199605978	0,128060281
CD1C	0,199699773	0,053407867
MAGEA8	0,199751747	0,627630575
KIAA0922	0,199941519	0,430575436
KRTHA1	0,200032494	0,586772674
C16orf45	0,200052334	0,538240183
CDH17	0,200118873	0,548732213
GDF10	0,200170252	0,811159661
RNF212	0,200261314	0,103054245
LOC389813	0,200271844	0,761930539
RTN4IP1	0,200334514	0,160980379
LOXL2	0,20055015	0,650136168
FAM120C	0,200601868	0,454708788
LIME1	0,20068928	0,971942676
UNC13D	0,200726692	0,74661747
CUTL2	0,200844301	0,337361391
FOXO3A	0,200963282	0,425885763
ANKRD55	0,201186413	0,590755606
ECE2	0,201235027	0,400005064
SPAM1	0,201256785	0,371071618

GPR150	0,201272276	0,941396685
GABRP	0,201307019	0,414258796
ASGR2	0,201394755	0,76431645
LOC641832	0,201602052	0,529697753
C3orf60	0,201613274	0,568083691
PPGB	0,20164745	0,497146379
C14orf148	0,202097989	0,420013711
C17orf87	0,202103102	0,169507522
RER1	0,202105419	0,478794758
C6orf168	0,202153499	0,142053813
FAM178B	0,202197721	0,45909058
AVIL	0,20229407	0,351496954
KIAA0182	0,202438142	0,173577192
SPRR2G	0,202517484	0,289823041
POLS	0,202696645	0,270793021
CST4	0,202747988	0,683316106
HOXB1	0,202910461	0,298742124
PTHR2	0,202996558	0,581129092
RPS10	0,203013954	0,069548933
MCF2L	0,203057763	0,177880863
ATXN10	0,203060983	0,852354991
GAS6	0,203083285	0,581214136
SULT1A1	0,203095656	0,946835475
CNTROB	0,203176333	0,51353747
LOC647407	0,203334437	0,207557485
ZNF224	0,203345306	0,172128854
GPR30	0,203597708	0,236101117
MAGEB4	0,203611862	0,186841451
FZD10	0,20367716	0,305767424
GPR31	0,203723396	0,829301827
DDI1	0,203724243	0,481140822
MYEOV	0,203823096	0,143172922
TPRT	0,204073796	0,570698548
PEPD	0,204124886	0,246396518
MTHFR	0,204875905	0,653463476
IGF2AS	0,204892164	0,026467631
PRR16	0,204893884	0,045497459
GPR172A	0,204937963	0,517031339
COMMD10	0,205008361	0,313076208
LOC653881	0,205076636	0,154827253
PTE2B	0,205396808	0,212869321
FLRT2	0,205518849	0,628316758
IL10RA	0,205593975	0,430516072
ALPPL2	0,205614965	0,946411693
OTX1	0,205691777	0,275857436

LOC650689	0,205745554	0,075719912
LOC222032	0,205808774	0,727619499
MIST	0,205837622	0,94804241
NARS	0,205941999	0,385329653
TMEM195	0,206060646	0,394910893
MAD	0,206231346	0,302977327
KIAA1279	0,206361203	0,61007785
SLC5A3	0,206363075	0,900954498
LILRA6	0,206393461	0,371925956
EGFL5	0,206730558	0,583605363
CXorf22	0,20705368	0,216969447
PON2	0,207095353	0,953919692
SLC6A4	0,207133866	0,545298552
ATP5C1	0,207245552	0,926985184
EML5	0,207291337	0,284171475
IGFL4	0,207323724	0,347957228
KLHL1	0,2073727	0,340998242
GLTPD1	0,207443769	0,320757912
DZIP3	0,207450925	0,59135075
AP2M1	0,207602263	0,435181461
HIBCH	0,207620373	0,188613935
NEK7	0,207636293	0,290818212
NR1I3	0,207803944	0,386241093
C18orf51	0,208045739	0,950357887
FBXW8	0,208051792	0,90633653
CCDC7	0,208071478	0,624481115
MSX2	0,208168047	0,233788824
CYP2C8	0,20820857	0,897306748
CD163	0,208328656	0,651570074
PRAMEF5	0,208336246	0,340861723
CEBPA	0,208399123	0,900097637
ZNF320	0,208411308	0,070707694
OR13J1	0,208433808	0,443990505
ANKRD47	0,208437455	0,175990102
ARHGAP11B	0,208491253	0,741254764
LAMP3	0,208521776	0,315500109
SMARCA2	0,208539728	0,555792764
ISG20L1	0,208597977	0,211624353
CD4	0,208607991	5,87E-10
EPHB6	0,20863036	0,029020488
C20orf79	0,208762283	0,114141073
LOC643677	0,208835364	0,154430907
MYOD1	0,208868807	0,818233972
LOC400987	0,20893264	0,960775689
RGS1	0,209053746	0,120671891

OR52B2	0,209429107	0,403279171
DKFZp761B128	0,209557473	0,370255573
OXGR1	0,209767628	0,353937121
SEC14L4	0,210044457	0,877500542
ODZ2	0,210064851	0,393117621
CCDC40	0,210082494	0,175027488
PPP1R12A	0,210212407	0,170264108
hCG_1778643	0,210268748	0,08340164
MYL5	0,21038412	0,442512057
TMEM63A	0,210401396	0,406427102
PSAPL1	0,210419031	0,476499297
RBBP5	0,210453641	0,074550804
LOC644925	0,210460861	0,184234182
LOC729011	0,210710824	0,216234144
FLJ38028	0,210926659	0,918522219
IL29	0,210999939	0,230570196
OTUD1	0,211107115	0,528242895
PORCN	0,211113475	0,692505503
LOC728107	0,211196811	0,619435447
SESN1	0,211317171	0,159016128
GSDMB	0,211469815	0,154568249
NID2	0,211474584	0,727842448
FILIP1	0,21154863	0,810144902
HIST1H4G	0,211768409	0,445421755
PRKWNK3	0,211953688	0,353732892
TSPAN1	0,212171702	0,834377936
C9orf116	0,212396826	0,254123577
MSX1	0,212522625	0,602058209
SLC37A1	0,212566068	0,489919647
hCG_1660138	0,212569314	0,39184679
MVK	0,212587064	0,509333008
LOC728175	0,21266079	0,400553912
C14orf105	0,212823943	0,325251532
HNRPF	0,2131741	0,115953198
ASB3	0,213222288	0,70111183
CASP5	0,213255762	0,364423601
LOC728626	0,21327448	0,282575791
FLJ30726	0,213470959	0,829543612
BCL2L1	0,213555754	0,262960642
SCML1	0,213595945	0,027554182
LOC342918	0,213762164	0,159068904
LIPT1	0,213902834	0,122389409
S100A15	0,214022635	0,339941195
PSME3	0,214065057	0,413465454
LOC652046	0,21411362	0,304253421

DNAJB2	0,214142112	0,570499591
LOC730596	0,214182993	0,60008836
SCNM1	0,214461584	0,353296612
C20orf3	0,214678971	0,881109943
USP36	0,214795273	0,180291513
C1QTNF6	0,215270223	0,424570431
RPP14	0,215661963	0,242047655
PIM2	0,215701595	0,170137673
ZNF639	0,215997983	0,950639577
GM2A	0,216065923	0,531181457
C14orf149	0,216275721	0,524866544
VAV2	0,216309584	0,235072104
SLC5A5	0,216348369	0,311073197
LOC729474	0,216382696	0,999429318
FLJ42280	0,216458371	0,702488768
PANK2	0,216614927	0,306143348
ZDHHC11B	0,216712746	0,208724961
MEP1A	0,216840672	0,38349534
AR	0,216956272	0,187287268
SYNGR2	0,217057694	0,329774606
PRPSAP2	0,217107289	0,14449082
SLC25A25	0,217174492	0,382050721
LOC729018	0,21721481	0,185514095
PLEKHA9	0,217219243	0,355173766
LY86	0,217244438	0,63580969
C10orf75	0,217370289	0,695975445
KREMEN2	0,217428127	0,802988805
LOC728432	0,217578581	0,240121093
RWDD2B	0,217610951	0,075267709
CPA1	0,217914218	0,317145959
TAF1C	0,217991831	0,819883533
MDH1	0,218134479	0,137510902
SPC18	0,218300245	0,774532755
LOC441617	0,218337964	0,074075935
PSMA7	0,218349108	0,714814999
RRM2B	0,218546509	0,608229274
STS	0,218552081	0,680620132
KIAA1191	0,21870026	0,041369824
LOC728478	0,21879857	0,247738884
FOXD3	0,218884641	0,296548495
NUP54	0,218889039	0,184666088
TMPRSS6	0,218895736	0,186132685
GLRA3	0,218997972	0,455364127
PPP2R1B	0,219173653	0,416864131
LOC731320	0,21919236	0,285980878

PSMA1	0,219218092	0,878011638
TBL2	0,21936185	0,445677562
DNCL1	0,21936315	0,314201359
RNF31	0,219374407	0,537855838
UBE2C	0,219491362	0,454022382
RP11-353N4,5	0,219511215	0,639498919
LOC654029	0,21980606	0,176714578
DKFZp434J1015	0,219832683	0,108428909
BTNL3	0,220068301	0,203735504
LOC729503	0,220243642	0,936930532
IMP5	0,220254454	0,990260553
ATXN7L2	0,220279045	0,597293824
GUCA1B	0,220345807	0,608629977
ZNF12	0,220355329	0,675826778
NEK2	0,220369501	0,180160854
DIABLO	0,220385573	0,759459919
LOC731250	0,220503068	0,310231985
DLX1	0,220617808	0,07958071
hCG_2023776	0,220757414	0,524607339
LOC647000	0,220886229	0,680797347
SLC12A3	0,221072451	0,444192384
GAS7	0,221092527	0,17485785
LOC649984	0,221188622	0,80735936
UBD	0,221412071	0,682316482
TXNRD2	0,221608934	0,293070941
TGFBR1	0,221691644	0,553951626
LOC650947	0,221717882	0,791095039
ACAT1	0,221880257	0,894396669
C1orf146	0,221948097	0,301976541
KIAA1573	0,222037883	0,377759909
HINT2	0,222447009	0,779444096
CEP120	0,222470938	0,093659544
IBTK	0,222677986	0,33693141
LOC645263	0,222902984	0,916311878
SPINL	0,222995686	0,264767761
SSTR2	0,223016084	0,308252172
NMT2	0,22302261	0,922837126
KIAA1324L	0,223053676	0,59601244
WAPAL	0,223071108	0,142506543
FOXA3	0,223220183	0,795757469
GARNL3	0,223278827	0,016855009
OR10AD1	0,223384097	0,252258939
COX15	0,223421554	0,812302565
THAP4	0,223475351	0,493971657
FBXO24	0,22355204	0,588653245

FAM86B2	0,223640692	0,300878693
TSCOT	0,223673439	0,427013913
K6HF	0,223893145	0,907026818
RP11-408E5,4	0,224079432	0,122930988
WISP2	0,224459863	0,718510377
HEMGN	0,224485165	0,143887272
JARID1C	0,224564457	0,569261591
RNF10	0,224568632	0,286274864
MINPP1	0,224796297	0,059937922
TMEM156	0,224822761	0,540852402
HTATIP2	0,225017789	0,157470164
FAM105B	0,225085295	0,865267211
HMHB1	0,225088429	0,103256024
OR5AS1	0,22511714	0,27077872
STARD5	0,225124156	0,812791054
GPC3	0,225132251	0,333828208
TMEM69	0,225133492	0,579697065
TMEM140	0,22514616	0,872178325
DNASE1	0,225180998	0,205626914
LOC201292	0,22532287	0,812624928
CYP2A7	0,225417926	0,488567157
FBXO43	0,225430637	0,229592035
BAG5	0,225450111	0,84968032
CAP1	0,225676343	0,188133669
RAD51C	0,225700697	0,172536404
LOC729717	0,225812544	0,277930066
FTS	0,225969965	0,831512117
C4orf28	0,226301432	0,312464545
SCXB	0,226494867	0,381341593
SULT1E1	0,226515236	0,666555697
CXCL17	0,226632388	0,73932286
KIAA0776	0,226665281	0,22243785
XAGE5	0,227066917	0,180555527
LOC441907	0,227073303	0,400979277
KLRC3	0,227153302	0,305310373
LOC728284	0,227346128	0,649969349
MLLT4	0,22735979	0,661341612
LDOC1L	0,227405129	0,538142081
SLC3A1	0,227518731	0,354711338
LOC645320	0,227637138	0,323431681
UBE4A	0,227715627	0,430703666
LOC650050	0,227724418	0,389473898
MYBPC1	0,227834806	0,282744759
CYB5D2	0,227859601	0,642641715
FAT2	0,227874189	0,144639663

C10orf115	0,227945812	0,252852058
C11orf40	0,228118014	0,824336458
TMEM136	0,228200435	0,878990947
THG-1	0,228332627	0,133646247
DNM1	0,228495998	0,291931818
CAS1	0,228518587	0,501451166
PYCRL	0,228518874	0,478524989
HLA-C	0,228567192	0,305481129
WTIP	0,228637386	0,102770583
AKIP	0,228671189	0,123826448
NUB1	0,22883035	0,340003029
LOC730002	0,228882232	0,611810036
LOC727844	0,228950981	0,54785866
CFH	0,229029307	0,455526048
S100A6	0,229117113	0,598677328
LRCH1	0,229127474	0,188692852
BPGM	0,229250515	0,606014482
SCNN1G	0,229287284	0,319371963
BARD1	0,22936951	0,260721668
GPC6	0,229576757	0,035682764
SMYD2	0,229585663	0,899499526
RPS20	0,229670524	0,825149599
LIPC	0,229917385	0,189105286
LOC727799	0,229936757	0,787042972
FGFR3	0,23008758	0,208994842
PDE7A	0,230210936	0,97971766
CENPF	0,230395196	0,160169668
LOC731804	0,230577955	0,743744236
PTPRN2	0,230760709	0,516111705
DEFA6	0,230800433	0,141420437
CORO2A	0,230884796	0,959086734
C17orf50	0,230886321	0,182120601
SRPR	0,23124358	0,397840967
hCG_1982709	0,23138678	0,298142582
KIAA0082	0,23154479	0,102809813
LOC129293	0,231579846	0,746900374
TMEM209	0,231604271	0,280214767
KCNJ14	0,231627116	0,247236314
PAXIP1	0,231636916	0,874790862
PYCR1	0,231690247	0,838206287
SLC12A6	0,231963269	0,259763395
LOC648672	0,231989437	0,187118751
HSPA1B	0,23198948	0,24223224
SERPINB10	0,232022038	0,427366976
RPL9	0,232034888	0,236819572

FIG4	0,232523085	0,165544619
LOC729611	0,232533427	0,599903235
ACTL7B	0,232622374	0,280134938
ALG9	0,232638944	0,683369351
LIN7B	0,232678028	0,947919483
DCUN1D2	0,232728583	0,158062438
TSPAN32	0,232873837	0,148937812
FBXO33	0,233003784	0,377690021
LOC652631	0,233060808	0,637468187
SORD	0,233221625	0,374895881
FLJ12606	0,233526627	0,997319496
CD33	0,233573996	0,962105404
CSTB	0,233890963	0,223774114
SPAP1	0,233909737	0,098114008
OR5AK2	0,233963636	0,162983108
LOC646119	0,234276355	0,596088513
CSPG5	0,234283424	0,772434956
BAAT	0,234283784	0,335685281
BMP3	0,234322472	0,110383593
LOC389834	0,234639723	0,084093919
C6orf201	0,234656047	0,728169974
LOC650468	0,235313651	0,957146489
MRPL52	0,2354872	0,726071389
LOC729672	0,235618683	0,361195252
UGT1A9	0,235643055	0,167939712
GPR149	0,235661713	0,225322365
TMEM82	0,235666145	0,294896751
FAM148A	0,235702256	0,787811016
ASB17	0,235724295	0,199407637
NPAS3	0,235798802	0,981762672
C21orf63	0,235800544	0,733755964
OR8S1	0,235815748	0,494322787
CMTM3	0,235826682	0,859178985
CYB561D1	0,235908633	0,911438635
HELLS	0,236069862	0,313214085
NBPF6	0,236133655	0,199371857
DMAP1	0,236191763	0,884124851
CRYGC	0,236397271	0,176626929
APOBEC3G	0,236650152	0,537238047
FLJ20489	0,236690594	0,342544144
ADCYAP1	0,236691412	0,162035202
LOC642597	0,236736451	0,174566913
NR1H4	0,236962304	0,151054815
USP16	0,237138244	0,086036299
MKI67	0,237201884	0,294470825

IGFBP5	0,237378616	0,224170324
LOC648749	0,237381576	0,312901687
MDK	0,237714489	0,143108179
TAPBP	0,23785005	0,164779791
CHODL	0,237893601	0,407253994
PAPOLB	0,237983178	0,834875485
LOC652010	0,237998552	0,580977926
ZNF324	0,23802038	0,522087687
FLJ45422	0,238180339	0,930669169
FBXW12	0,238218653	0,524808975
ITIH5L	0,238373027	0,702744493
USP3	0,238507401	0,664985014
MT2A	0,238556523	0,18198507
RAB31	0,238699791	0,124451697
SIT1	0,238789569	0,23226334
CAPNS1	0,238812622	0,603137327
LOC645843	0,238857189	0,705470227
TOP3A	0,238881864	0,445789218
FGF9	0,239216428	0,523650806
RPL27A	0,239253539	0,6255458
SOCS5	0,239513359	0,487020074
RHOV	0,239777469	0,92888402
LOC389936	0,239877543	0,213799288
LOC730811	0,239905974	0,700079455
LOC729087	0,24035563	0,163444011
COL24A1	0,240489668	0,223105271
C3orf55	0,24054626	0,427124273
FCHSD2	0,240632714	0,324674581
SI	0,240706531	0,672880812
CHEK1	0,240718388	0,760432558
CECR6	0,240955936	0,501745859
WDR31	0,24100668	0,230786426
CPA5	0,241029798	0,142079261
STARD13	0,241222003	0,464530769
C6orf91	0,241261164	0,379107941
XCR1	0,241393044	0,271366102
PON1	0,241413441	0,66367016
KIAA0117	0,241435728	0,410657743
FLJ30092	0,2415725	0,750954362
FLJ20184	0,241730052	0,972026256
LOC729471	0,241754214	0,951769059
LEPREL1	0,241983418	0,354479024
RASGRP2	0,242208512	0,609577462
MGC9564	0,242498371	0,278268468
SLC7A9	0,242514763	0,287636965

ANKIB1	0,242654463	0,120297665
HS3ST5	0,242747497	0,18836691
PAPD5	0,242761089	0,455357142
KIAA0211	0,242842033	0,411729527
COPS5	0,242968605	0,094246118
EPS8L3	0,243138637	0,775100555
THEM2	0,243160542	0,808329631
IFI6	0,24316196	0,232528836
MGC42105	0,243231267	0,625443981
OR6K6	0,243416274	0,856161598
FLJ45721	0,243494833	0,138251495
FCN1	0,2434974	0,90877016
LOC201175	0,243521578	0,073981001
PTPLAD1	0,24356995	0,612474517
S100A1L	0,243584612	0,244576838
KRT19	0,243585626	0,352316027
LOC651987	0,243901095	0,309680405
MRPS15	0,243985719	0,090553547
CDH9	0,243988403	0,393248819
GRIA1	0,244035199	0,245546525
NKAIN2	0,244069302	0,549921174
KRTAP6-3	0,244154418	0,815657103
FAM71F1	0,244198003	0,250739417
FXR2	0,24421209	0,1939136
DEFB124	0,244247403	0,499452067
SULT2B1	0,244343956	0,571786345
NDUFB5	0,244502379	0,983794319
LOC646130	0,244532034	0,853711927
ARHGAP8	0,244748998	0,546027175
CYP26B1	0,245039666	0,789053202
C5orf33	0,245169688	0,209392894
KCNA3	0,245184377	0,213417102
PUS3	0,245204398	0,111141506
FOXP1	0,245296727	0,38855491
TNFRSF8	0,24571678	0,305069356
IRF7	0,24588775	0,490774857
MPRG	0,245920051	0,248984561
KCNJ2	0,246024114	0,349038416
DPEP3	0,246169729	0,306570031
LOC645852	0,246237886	0,019972245
CDK5R1	0,246254707	0,43271966
C1GALT1	0,246583659	0,280708609
TADA3L	0,246614521	0,164506996
CDC34	0,246670647	0,143037255
OR2M5	0,246870644	0,214513177

LOC441956	0,247143269	0,941725707
VCPIP1	0,247159016	0,43450292
DEFB130	0,247226513	0,54897534
CCL2	0,247251805	0,738678742
MBD5	0,247253548	0,848659279
HIST1H4D	0,247288443	0,992063745
C2orf49	0,247382193	0,127708286
FLJ20668	0,247413397	0,741197521
CAMSAP1L1	0,247423362	0,154800741
LOC728844	0,24744553	0,562561154
SELL	0,247462049	0,160098596
LOC200420	0,247661134	0,134396704
LOC727840	0,247755449	0,293319673
DOHH	0,247788917	0,419008708
ZNF613	0,247825434	0,167307967
FBXL22	0,247830423	0,637066177
ZNF330	0,247832337	0,291317482
TEX13A	0,247935843	0,195099694
WBSCR22	0,247948582	0,314485442
MS4A13	0,248074675	0,388823557
CFHR2	0,248135438	0,090360856
LOC727867	0,248145752	0,121278815
SCEL	0,248361942	0,332366489
MASP2	0,248399492	0,555536562
TMEM187	0,248859067	0,267381757
TSFM	0,248866436	0,204076349
LOC730916	0,248903263	0,59080885
TNFRSF5	0,248921377	0,413018124
AFP	0,249106273	0,207466038
HOXA4	0,249160169	0,287281167
USP35	0,249385008	0,451532871
SPOCK2	0,249454586	0,159950502
ANKLE1	0,249455962	0,933745096
HEG1	0,24954189	0,247889362
OR7E24	0,249575676	0,075556489
HMG20B	0,250054303	0,124637367
FBXL20	0,250111334	0,072723517
FLJ32682	0,250213485	0,136273543
PMP22CD	0,25030769	0,265895517
KIAA0368	0,250332639	0,114040643
SNX22	0,250552496	0,583145698
LOC339209	0,250591108	0,176772477
BLVRB	0,250730347	0,156345411
ANGPTL1	0,250736385	0,163011101
ECM1	0,250736438	0,355358051

CDKN2B	0,250795446	0,616990443
UBE4B	0,250822678	0,805542393
GHDC	0,250946009	0,273069665
OR4D6	0,251254146	0,636697877
PCDHGA2	0,251266585	0,181066401
CDC25C	0,251323736	0,279303557
LOC340371	0,251348551	0,872070868
KIAA0196	0,251521971	0,579644962
KCNK17	0,251901199	0,864377128
C20orf94	0,251912724	0,810825726
CCR1	0,251927692	0,712249843
MYO15A	0,252029011	0,197260561
FXYD5	0,252492629	0,210688792
PSG5	0,252896634	0,855777235
GNA14	0,253018827	0,079903473
CNOT7	0,253157616	0,131722774
CP110	0,253187522	0,44435608
LOC285191	0,253286442	0,125046743
BFSP1	0,253692056	0,76027068
FKRP	0,253723632	0,964551008
LOC728332	0,253726541	0,929506946
LGALS2	0,253903621	0,316016485
GRIK1	0,254081629	0,608289187
NHLRC1	0,254133553	0,705238335
LOC646449	0,254169448	0,621021135
SERHL2	0,254221748	0,848019444
DEXI	0,254383573	0,597743428
SLCO4C1	0,254468032	0,318890753
FAM123C	0,254527066	0,717027428
C11orf67	0,25461842	0,183278326
ECGF1	0,254646356	0,670340691
OTOP1	0,254928086	0,385376948
RELL2	0,254974804	0,122058865
C14orf79	0,255088796	0,185439254
OR5D13	0,255468575	0,216182984
LOC541473	0,255551579	0,754225662
SLAMF9	0,255674405	0,488513718
LOC144383	0,255675717	0,345777593
PTPRD	0,255827992	0,133195927
MAP3K3	0,25583715	0,084515052
POLR3G	0,255877628	0,363494583
C16orf87	0,256423338	0,709058721
BTN2A1	0,256456996	0,185437059
CREG2	0,256497343	0,500177141
HIST1H2BH	0,256538893	0,100965617

LIX1L	0,256665284	0,787258036
KLHL3	0,256789415	0,983137779
hCG_2029702	0,257012091	0,085091441
ADRA1D	0,25716984	0,428973373
KCNA5	0,257321664	0,310010714
FLJ38628	0,257597681	0,250361777
PRIM1	0,257623687	0,744892064
EXOC4	0,25792399	0,175083822
SCO1	0,257924316	0,22116723
NCRNA00153	0,257975391	0,261147563
JAK3	0,258018148	0,36613083
NEUROG1	0,258192401	0,999598793
RARSL	0,258295598	0,279733097
CBLC	0,258481084	0,563008823
SCGB2A2	0,258634677	0,093177479
YARS	0,25868736	0,870556908
EBF3	0,258777179	0,137498541
ACTA1	0,258879999	0,429028832
FLJ21767	0,258990011	0,483623071
LOC728621	0,259188126	0,472398888
ATF3	0,25921864	0,625253414
CORIN	0,259250798	0,385997916
HBM	0,259265972	0,6690982
RNF220	0,259292567	0,538652005
NKX2-2	0,259312054	0,103743553
ETNK2	0,259317072	0,603235007
HEPH	0,259340274	0,715114207
CCNT2	0,259522462	0,110086266
AIM1L	0,259719966	0,187721819
AGPS	0,259724414	0,217483677
EPM2A	0,259959659	0,635449303
LDOC1	0,260114838	0,131774505
HYAL4	0,260790568	0,891111922
ADAMTS2	0,260924293	0,175477547
NLRP4	0,261010558	0,182871536
RNASET2	0,261042921	0,870387898
CARKD	0,261156193	0,286523091
LRMP	0,261234174	0,182602347
PAOX	0,261771618	0,19625855
CUL4A	0,261782145	0,204683835
PPP3R2	0,261831671	0,209467584
TLR4	0,261996356	0,720577902
ORC3L	0,262125295	0,97772109
LOC731564	0,262130845	0,021233908
SC65	0,262139154	0,22286287

OR2T29	0,262377071	0,245016062
ZNF134	0,262859309	0,159797621
ZFP36L1	0,262938763	0,197258821
CDT1	0,262944345	0,348414095
KRT20	0,26308172	0,990816844
ASPH	0,263494675	0,714065883
TMEM14B	0,263508733	0,080305413
PLCD3	0,263646881	0,059220396
C12orf51	0,263762518	0,094478977
TMEM27	0,263857438	0,719341837
ZNF345	0,263920555	0,880805667
LOC645435	0,264126625	0,385192871
FLJ36032	0,264143817	0,121260191
TUBA4	0,264346658	0,478426733
LOC728476	0,264417259	0,230693267
KRT4	0,264431529	0,385933165
GLIPR1	0,264606192	0,153403578
CDIPT	0,264958448	0,187959587
VGLL4	0,265170029	0,338912348
ARL8	0,265533771	0,510438245
LOC391347	0,265557179	0,286602318
ATP6V0E2	0,265643536	0,940915837
FBXO44	0,265729191	0,369436631
LOC732005	0,265776948	0,704771521
FLJ40160	0,265800504	0,357900466
POLR3GL	0,265834732	0,125461572
IGFL2	0,265853134	0,721137463
ACRBP	0,26600388	0,101676587
SLC16A14	0,266248029	0,333917015
DNAJC12	0,266348738	0,941864799
LOC652741	0,266573772	0,115333765
NME7	0,266677653	0,248084868
LOC652578	0,266795614	0,450000342
C11orf24	0,266956728	0,11983907
GSH-2	0,267378138	0,480014605
KHK	0,267450665	0,834308359
PRCP	0,267512811	0,379026531
LOC645158	0,267576846	0,577118069
FEM1C	0,267606173	0,230014932
RBM41	0,267639669	0,186407843
KRTCAP3	0,267795082	0,1079252
CAMTA1	0,267855521	0,572345644
OR51T1	0,267956918	0,44555397
CST7	0,26835041	0,554215575
DCTD	0,268385597	0,105551022

C19orf60	0,268444759	0,764024655
DUSP13	0,268462205	0,728444862
MAK	0,268524714	0,816576152
C6orf126	0,268863984	0,475821784
RIF1	0,268921471	0,145281907
C2orf52	0,268981801	0,739193743
BAALC	0,269300271	0,610882239
ANAPC1	0,269345433	0,376521393
C20orf186	0,26935938	0,628991663
TBC1D14	0,269447732	0,492046647
TTC10	0,269458671	0,369486826
NT5C2	0,269727684	0,359117683
MS4A6A	0,269731937	0,32507225
LOC644548	0,269785548	0,239925174
WASH1	0,269872475	0,543830885
OR2Y1	0,270036083	0,190279918
PIK3R2	0,270073729	0,290359771
TGFBRAP1	0,270180337	0,609927409
AMBP	0,270538522	0,46170419
STK35	0,270672737	0,725853993
SLC1A5	0,270676453	0,230681531
SYNJ2	0,270714329	0,114344868
SPPL2B	0,2708044	0,319358104
ODF2	0,270922095	0,338409119
LOC727992	0,271004855	0,544711612
TNL	0,271013749	0,384435584
KIR2DS1	0,271135933	0,737239075
KLHL30	0,271268815	0,126953267
DNAJC6	0,271294046	0,193744548
HNRNPH2	0,271297403	0,323643413
STAT4	0,271311357	0,549787459
FCGBP	0,271330737	0,117250508
CRIM1	0,271338534	0,620867342
LOC644397	0,271402704	0,88161589
UBE2D1	0,271420855	0,534551093
KIAA0222	0,271449646	0,141142204
ITGAE	0,27154641	0,955035089
SH3BP3	0,272092841	0,767539997
IGFBP2	0,272097331	0,325018426
IRX6	0,272134553	0,474730981
WDR22	0,272140131	0,439103625
C6orf111	0,272194374	0,055239177
TPRA40	0,272229741	0,19473197
PCDHA10	0,272393957	0,287567365
LOC652636	0,272395883	0,81145244

LOC344875	0,27241351	0,103781594
GNB2	0,272527678	0,079767766
PGLYRP1	0,272566823	0,208946156
MTIF3	0,272693946	0,238884422
PP1665	0,272707234	0,479307466
LOC643923	0,272710744	0,080844191
REST	0,272722023	0,762463559
DNAJB6	0,272728549	0,5163359
C5orf20	0,272756173	0,994955414
MLNR	0,272925361	0,189426037
SIAT7A	0,272972001	0,506941013
SYTL5	0,273109914	0,548141955
TMSB15A	0,273163159	0,675451159
PRDX2	0,273284478	0,143033185
DUSP11	0,273356867	0,238988198
CDA08	0,273403164	0,915413877
OSCAR	0,273441843	0,240135387
TRPV5	0,273472868	0,999581539
GUCA2B	0,273491551	0,389329213
LOC643305	0,273498642	0,718376623
KLHL14	0,273552205	0,316633357
FTO	0,273618657	0,618606167
DOCK4	0,273723255	0,102108343
DKFZP434I0714	0,273780793	0,180927375
C20orf152	0,273960292	0,019340981
TFAP4	0,274031576	0,377350386
TNC	0,274034357	0,356394744
NLRC5	0,274038192	0,283075619
CCDC78	0,27408307	0,497744043
C9orf37	0,274122102	0,484620565
TWSG1	0,27423144	0,864938844
CHST6	0,274249001	0,345548192
ACSL3	0,274296862	0,60631991
XRN2	0,274440019	0,099584617
DNAJC15	0,274466677	0,145944982
CSF2	0,274531227	0,522966274
VASP	0,274531548	0,253968461
LOC652215	0,274546615	0,133298267
CXCL3	0,275173735	0,731733959
CCNG2	0,275475265	0,498377842
CNGB3	0,27576362	0,011108634
C20orf134	0,275834106	0,281552331
THBD	0,275967763	0,105607278
CTSG	0,276017506	0,855233669
NOX1	0,276141375	0,120874103

ARHGEF15	0,276418191	0,808886456
LOC731860	0,27669176	0,897046647
D4ST1	0,276701699	0,35293299
ERP27	0,276892294	0,377528564
FLJ22595	0,276960702	0,223444908
LOC653073	0,277033553	0,567616391
CDCA7L	0,277043743	0,147133568
C10orf113	0,277082761	0,093356953
NT5DC3	0,277198497	0,293725988
SMAD2	0,277200145	0,610231688
CLEC2D	0,277295731	0,567851382
HIG2	0,277404518	0,416768949
DIRAS2	0,277757037	0,797062219
LOC728394	0,277814121	0,33381545
SNX17	0,277899924	0,14254574
BRWD1	0,277946356	0,216984855
PRAP1	0,278031966	0,306545905
KLHDC10	0,278040905	0,255759121
NAALAD2	0,278063396	0,151187225
PTCHD2	0,278190094	0,589574297
HSPA5	0,27828146	0,197276602
SS18L2	0,27840866	0,711215305
LOC728392	0,278631289	0,55641653
LOC441097	0,278686339	0,535254175
FAM90A1	0,278737191	0,307987595
CYP1A2	0,278744947	0,34863704
LCMT2	0,278898151	0,185320035
LOC647811	0,279247923	0,246149537
C2orf21	0,279952791	0,207154479
FIBCD1	0,280055515	0,159128835
TMEM59	0,280087805	0,158394303
LOC442064	0,280110217	0,025963645
OR14J1	0,280215393	0,254361348
SNAP23	0,280479707	0,230533071
RIOK1	0,280530611	0,275889442
EXDL1	0,280583159	0,070936057
FLJ22659	0,280588064	0,682016189
DONSON	0,280633883	0,654576744
PPP2R5D	0,280955254	0,562529008
NR2E3	0,281169546	0,14619216
LOC158376	0,281188328	0,141588108
LOC731228	0,281238015	0,513665579
C10orf90	0,281243673	0,192971877
POM121	0,281312968	0,729792328
SIAT9	0,281470356	0,711255558

ZNF539	0,281481987	0,437790991
RIBC2	0,281554634	0,08519958
MARCKS	0,281631447	0,612814004
ISG20L2	0,281705838	0,224146733
LOC387885	0,281937506	0,159386102
LOC388789	0,282026745	0,179606419
CCDC4	0,282130196	0,397019074
PPIL1	0,282287296	0,454407354
ACACA	0,282297257	0,317573619
C1orf212	0,282299496	0,310225625
SUFU	0,282583285	0,363786085
ZC3H14	0,28279226	0,721497803
GLRA4	0,283202273	0,16537825
ATP6V0A1	0,283243647	0,384429893
WIBG	0,283482895	0,123953788
LOC402715	0,283756625	0,205117933
WFDC11	0,283880608	0,116070858
MMAA	0,283933044	0,150737012
LOC730252	0,283945915	0,414636501
HRH3	0,284000554	0,296527886
LOC728884	0,284030996	0,278833975
PHF12	0,284200062	0,477518643
OR11L1	0,284266587	0,649626082
C8orf31	0,28430246	0,508351584
UROD	0,284735907	0,078034986
LOC732161	0,28488307	0,194063124
FBXO36	0,284914173	0,145272449
LOC729218	0,285076452	0,68499465
C12orf37	0,285131302	0,634276423
FLJ25076	0,285168544	0,393910953
JARID1A	0,285248827	0,712998891
SERPINA12	0,285316282	0,846647103
MIA	0,28543192	0,708543881
ZYG11A	0,285539027	0,75640286
USP8	0,285694945	0,068565425
LOC646938	0,28586662	0,077171277
ZNF200	0,285870681	0,515595489
SCGN	0,28610856	0,211707841
KRTAP5-11	0,286134812	0,358419968
ZNF229	0,286163822	0,051199767
ITGA7	0,286171562	0,414993486
ATXN7L1	0,286175489	0,377289573
ABCD3	0,286293053	0,122590857
LOC647868	0,286348153	0,619285846
MANBA	0,286389702	0,258777371

LOC642137	0,286402672	0,116103363
ADAM8	0,286537878	0,81097148
HOXD1	0,286648472	0,53255021
BTBD6	0,286922507	0,161829836
OR2G6	0,286974749	0,115384305
TNRC6B	0,287006387	0,732520712
LOC730090	0,287188759	0,088453884
TEX9	0,287208939	0,126749651
SOCS3	0,287887743	0,263584027
OR8B8	0,287982554	0,292738518
TMC2	0,288074139	0,634505769
HS6ST2	0,288193574	0,793985741
ERAF	0,288298739	0,06531324
LOC649597	0,288441894	0,146005985
CEP55	0,288459671	NA
C9orf43	0,288674243	0,048137984
KRTAP19-8	0,288686321	0,097760525
FGF12	0,288820886	0,861483037
C9orf68	0,288874093	0,117813473
LOC730295	0,288931272	0,787246249
LOC339742	0,289296625	0,062245345
ZNF434	0,28933246	0,364842047
ANKRD11	0,289585807	0,155982723
LOC730105	0,289598313	0,189915127
SLAIN1	0,289627506	0,056706119
MPHOSPH6	0,289689329	0,298667772
AADAC	0,289689699	0,304667917
AKAP8	0,289776509	0,567727801
C8orf42	0,289866896	0,270555728
RGPD3	0,289956921	0,609607364
SDCCAG1	0,290081098	0,054748324
UBE2N	0,29014529	0,232617123
UBE2A	0,290358253	0,365030409
TNN	0,290429563	0,995948661
VEGF	0,290508129	0,898135913
FVT1	0,290543913	0,271657021
LOC652491	0,29069504	0,596745971
KIAA0100	0,29076695	0,079356224
LOC348021	0,29084555	0,707019644
OR1Q1	0,290845719	0,306056489
LOC650483	0,291027823	0,987212463
PTPN12	0,291038209	0,186368996
LOC91801	0,291140059	0,993561884
PPP1R14D	0,291200976	0,459228356
NPHS1	0,291289391	0,757830686

CYYR1	0,291368207	0,236308919
PACAP	0,291449017	0,104618545
C1orf162	0,291472133	0,650588466
UBOX5	0,291492826	0,238194505
HMX1	0,291512187	0,343716353
PRKAR2A	0,29152089	0,16747125
NUS1	0,291702983	0,684942181
LOC728213	0,291814979	0,679665377
LOC729184	0,291847202	0,67643836
DTNA	0,291902735	0,312937765
AKR1B1	0,292115016	0,251584038
TUFT1	0,292219804	0,053141275
ZNF44	0,292258144	0,441068779
hCG_2028557	0,292393033	0,876374787
LOC169834	0,29240339	0,429550122
ALOX5	0,292419994	0,545334237
LOC730113	0,292594565	0,129290633
LCP2	0,292648133	0,749948348
CESK1	0,29276483	0,09381209
STK32C	0,292779699	0,84432264
FLJ31568	0,292805288	0,97647598
PINK1	0,292866892	0,100316225
LOC728390	0,292955727	0,826854975
SAS	0,293009021	0,257432162
CLDN8	0,293369289	0,157384915
TXLNA	0,293433472	0,129677123
RNASEH2C	0,293707465	0,944005232
FLJ45513	0,293955888	0,257536505
CXorf48	0,294077918	0,538296536
SLC25A23	0,294168886	0,406144069
TTYH2	0,294257059	0,882115667
KIAA1217	0,294324322	0,163685212
GK	0,294451048	0,633341872
FOXC1	0,294557619	0,08743509
OSTN	0,294562921	0,190788473
LOC647037	0,294712962	0,182738329
C20orf72	0,294779973	0,183617744
DEPDC4	0,294857696	0,159781594
C5orf38	0,294880535	0,22053923
ZCCHC12	0,295022755	0,068059073
FAM19A3	0,295151885	0,678197727
FLJ13213	0,295326344	0,155514867
LOC728731	0,295408618	0,472999088
LOC91461	0,295685553	0,303494026
C10orf10	0,295710681	0,20108219

GRN	0,295762512	0,037113953
PFDN6	0,295927305	0,872677338
LGALS13	0,295942672	0,378193164
P4HB	0,296014797	0,162131703
SPTY2D1	0,296052414	0,298183615
PDCD11	0,296111957	0,96468432
VPS28	0,296134591	0,264433553
LOC731069	0,296298716	0,196251789
LOC731868	0,296349685	0,853268342
NTSR1	0,296416849	0,9984056
HIST1H2BL	0,296608524	0,896612486
UNC93A	0,29666099	0,968182613
FBP1	0,296694589	0,621169658
FMOD	0,296807581	0,378742527
CRISP3	0,297012206	0,112474574
LOC26010	0,297104239	0,133193979
SGSM1	0,297265317	0,369296771
BMPR2	0,297438842	0,586252015
CBS	0,297601756	0,157051559
GPR124	0,297933292	0,880287501
FBXL8	0,298227284	0,200452749
SLC6A19	0,298376795	0,047483804
ZCCHC24	0,298475988	0,09381582
LOC51326	0,298509319	0,652142295
LOC730116	0,298925321	0,260387658
FAM83A	0,299041881	0,023570949
NACA3P	0,299079549	0,481455054
ZNF384	0,299126118	0,926887502
C10orf55	0,299129204	0,693942008
OTOL1	0,299146806	0,123269492
ZNF414	0,299203268	0,241128691
ZNF502	0,299330901	0,739764229
SAMD4A	0,299551971	0,241911987
LOC729439	0,299648256	0,098461533
PCDHB4	0,299717395	0,169014283
AFG3L1	0,299848361	0,427858924
MAD2L1BP	0,299926193	0,377337431
RWDD2A	0,300025774	0,984042036
MYO1D	0,300137185	0,667064529
BIRC2	0,300304634	0,974602057
LOC731559	0,300600587	0,146380392
LOC642451	0,300604956	0,41211121
MTHFD2	0,300639703	0,224027421
APOC1	0,300773607	0,345232967
FAM151B	0,300839006	0,044729805

TNNC1	0,30087087	0,074026211
OR11H6	0,300985352	0,935976409
FAM104B	0,301090348	0,732270957
C6orf57	0,301136996	0,417450511
GCM1	0,301204646	0,254093492
MSH5	0,301267843	0,208379286
SLC6A15	0,30132077	0,390887273
FHL3	0,301458282	0,095371754
BPI	0,30146243	0,312163843
LOC400752	0,30154324	0,289669462
WNT11	0,301637151	0,298379593
ASPRV1	0,301690993	0,268708773
CDK6	0,302031705	0,403268142
ZBTB12	0,302096193	0,075237241
MACF1	0,302171955	0,280508466
LOC348174	0,302250782	0,463835251
DEFB137	0,302321244	0,076924427
RPSA	0,302329925	0,482058364
RPTN	0,302377249	0,92975045
NFIB	0,302517759	0,212792934
NHP2L1	0,302580472	0,32019357
TIE	0,302850307	0,21016484
TMEM75	0,302944408	0,870844011
LOC641765	0,302960823	0,209059673
C9orf11	0,303002015	0,388996841
MDC1	0,303061437	0,132574847
LOC728182	0,303158981	0,996668633
SORL1	0,303356016	0,700170326
FAM43A	0,303428027	0,224362904
LOC650794	0,303695415	0,112538227
UAP1L1	0,30370009	0,04557058
CDRT4	0,303715156	0,292851134
SIRT3	0,303751094	0,50975632
BEX5	0,303792712	0,268439499
LOC644953	0,303802226	0,108980202
ARHGAP28	0,303828814	0,580622205
STXBP3	0,303852809	0,834056616
FAM83B	0,303958307	0,396999718
ETV7	0,30412184	0,313150952
LOC391508	0,304260876	0,346539178
LOC389033	0,304302189	0,15654956
LOC729065	0,304325451	0,591501065
RFPL3	0,304333791	0,549245205
XRN1	0,304602338	0,418998664
PLEK2	0,304664401	0,050730693

LOC646947	0,304679115	0,259565947
PCDHB1	0,304716748	0,556669312
INTS3	0,304783719	0,020912271
LOC647748	0,305165812	0,061712957
NPDC1	0,305204452	0,152732235
CPSF4L	0,305221194	0,622701885
ZCCHC10	0,305265312	0,262700358
LOC728002	0,305311381	0,542466165
LUZP2	0,305327711	0,153692147
NOTUM	0,305328144	0,819695885
LHX6	0,305376386	0,857815292
SOAT	0,305431908	0,525654121
LOC729777	0,30557392	0,447102485
LOC732441	0,305683819	0,155235345
PAH	0,305785275	0,028769482
SPP2	0,305832737	0,655578147
GPR32	0,305928727	0,098241613
GPR41	0,305959236	0,241309498
LOC729691	0,306328116	0,1936096
ZCWCC2	0,306492031	0,335287046
ZNF217	0,306521702	0,079302709
CLDN15	0,306582252	0,15601604
EDF1	0,307201711	0,065011377
MAEL	0,307206471	0,141428307
RNF213	0,307267537	0,195920373
LOC651386	0,307470741	0,695071199
ZNF398	0,307577566	0,273578375
ICAM1	0,307610723	0,46061643
ANKRD36	0,307620317	0,189125579
VPS8	0,307629276	0,308544527
LOC728292	0,307725049	0,130593916
TNXB	0,307764339	0,016170349
ATG16L2	0,30791939	0,178407236
LOC729741	0,308511399	0,022202696
STK39	0,308616928	0,67712869
LOC654253	0,308679423	0,605726647
DUOXA2	0,308733451	0,907566244
DLG2	0,308895968	0,739402543
SCAMP2	0,308990311	0,109019823
GDAP1	0,309011951	0,160643694
C11orf52	0,30930607	0,18316191
ANKRD7	0,309341179	0,620877952
FAM53A	0,30943064	0,32680826
LOC643355	0,309588377	0,448221137
LOC402164	0,309622604	0,183316128

DNAJC18	0,309742375	0,062648689
METTL1	0,309794394	0,844548291
LOC139604	0,309947273	0,731003643
LOC389151	0,309962187	0,014667015
LOC728616	0,309992013	0,365852584
PNLIPRP3	0,310057734	0,837708497
EXOC7	0,310070722	0,339810615
RIT2	0,31018329	0,194256458
PEX3	0,310192105	0,916673889
LOC648245	0,310402282	0,082985074
ACSF3	0,310471323	0,201597457
C15orf44	0,310973954	0,122032657
C13orf30	0,311015586	0,220368578
PLAUR	0,311064611	0,346700725
DDI2	0,311111778	0,22978179
MALT1	0,311133717	0,390710616
ALX3	0,311148914	0,175567693
LOC646603	0,311175949	0,990753132
BSX1	0,311267354	0,965974506
COTL1	0,311271396	0,570910115
ATM	0,311736977	0,072301145
PYGO2	0,311883975	0,489517391
TMC5	0,31189101	0,215223148
BCL2L13	0,311980414	0,182523332
FRMD7	0,312034231	0,767567489
ATP6V1F	0,312119263	0,754408846
FAM70A	0,312173899	0,483293952
GNAI3	0,312309158	0,321228696
ELA2A	0,312320389	0,192566754
SHARP	0,312384038	0,108371523
KIAA0895L	0,312639461	0,731137079
SLC43A3	0,312762873	0,144808183
PITX1	0,312919248	0,433630182
TPM1	0,312954203	0,207934238
SAP18	0,313020837	0,004102672
SEMA4F	0,313026879	0,144253213
RALB	0,313085715	0,30880818
PIGQ	0,313143591	0,213207695
UGT1A5	0,313156625	0,078646447
HMGN4	0,31322084	0,022191407
ZNF621	0,313502411	0,229530285
LOC728724	0,313682476	0,649431517
SLC25A15	0,313798589	0,685543551
CBX7	0,313799815	0,342926079
FARSLB	0,314012682	0,795164576

KLHL17	0,31415095	0,201611127
LOC728639	0,314172075	0,350883211
OR52N5	0,314226577	0,47788101
hCG_1645220	0,314270489	0,214746304
C6orf165	0,314322756	0,136991064
CTDSP2	0,314499434	0,435005764
LRRC43	0,314535575	0,847060134
CNOT10	0,314848043	0,110189705
MPZL1	0,314877368	0,545480965
CCDC101	0,315045808	0,318869927
SYNPO2L	0,315139834	0,906171638
TCP10L2	0,315173867	0,054901855
NPAL2	0,315260935	0,472378679
TCTN2	0,315303603	0,155648844
C3orf43	0,315333744	0,154955563
PHLDB3	0,315354913	0,184600437
NFE2	0,315416873	0,170359144
TGDS	0,315435715	0,895040559
TRIM10	0,315446744	0,174345255
MRPL38	0,315497852	0,513135041
ZFP64	0,315718021	0,109287067
CCDC109A	0,315909094	0,090384191
LOC645030	0,31594581	0,059747265
ZNF512	0,316146218	0,443011151
HSCB	0,316159634	0,161701031
PPP1CB	0,31616929	0,288766544
PEG10	0,316307013	0,479222862
C1orf63	0,316356828	0,633985711
LOC728667	0,316401279	0,180538896
SPARC	0,316423483	0,203939302
HIST2H2AB	0,316530292	0,345075032
TMEM1	0,316567447	0,249172273
LOC729650	0,316590217	0,172247872
DUT	0,316689524	0,111093191
UBXN1	0,316760533	0,7784969
SRPX2	0,316886827	0,835334492
C3orf58	0,317025243	0,344565985
LOC389328	0,317087925	0,182182202
RRH	0,317318881	0,199052095
SALL3	0,317327262	0,084373542
PPEF2	0,317372473	0,745481611
PRC1	0,317404294	0,518863916
WNT16	0,317425686	0,31682327
PLA2G2D	0,317717193	0,490140291
WDR49	0,318023061	0,096011181

ZNF468	0,318040315	0,257187908
LOC729455	0,318289514	0,171708508
LOC641776	0,318291078	0,665944435
ARSE	0,318416446	0,950892286
AMAC1L2	0,318639136	0,912277197
CTDSP1	0,318649465	0,957750038
ELOVL6	0,318667859	0,53526392
LOC731793	0,318674651	0,166192405
SAA4	0,318819063	0,210927057
LOC730441	0,318930242	0,219141079
FAM168B	0,318952012	0,064848799
LOC730132	0,318980199	0,22388271
PRAMEF10	0,319188915	0,148273514
TMEM182	0,319332573	0,638155679
TEKT1	0,319488205	0,348201991
PSMB9	0,3194891	0,18934159
PCDHA9	0,319584771	0,216022836
TMEM38B	0,320251034	0,196361767
STK33	0,320683093	0,832942981
IFI44L	0,320740116	0,358765596
HAMP	0,320777934	0,882577839
KLK9	0,320906728	0,116298629
LOC732450	0,320946345	0,358232265
LOC653560	0,321075591	0,071674841
TMEM16D	0,321136061	0,193575259
C20orf95	0,321272966	0,310831107
LOC729723	0,321354401	0,190402072
LOC65243	0,32138661	0,263329712
PAN3	0,321610232	0,570733004
FAM170B	0,321637051	0,538491378
RBM25	0,32210592	0,109766297
MMPL1	0,322221456	0,357570845
RTF1	0,322223486	0,868935708
C11orf72	0,322235847	0,36796271
LOC440731	0,322419161	0,953931818
LOC645757	0,322435634	0,11179619
C14orf115	0,322572359	0,770190478
AARS	0,322640502	0,435335336
CIDEA	0,322795759	0,225838067
C7orf58	0,323044117	0,069624943
SPACA5B	0,323059337	0,275141169
KBTBD7	0,323093702	0,718101403
PRKAR1A	0,323370418	0,189035064
NEFM	0,323629361	0,257976441
LOC647995	0,323725801	0,237705629

SP110	0,323928299	0,784336123
INPP5A	0,324452228	0,42663851
TREX2	0,324482042	0,217447863
NMBR	0,324523466	0,214280948
LOC647890	0,324535674	0,31206318
SF1	0,324681729	0,297717446
GPR126	0,324686312	0,207831468
TXNL2	0,324734887	0,108212827
C20orf24	0,324783267	0,088788081
CCDC120	0,324954434	0,982151887
FOXQ1	0,324970228	0,178921317
MTMR3	0,325060662	0,193962786
LCN1	0,32509482	0,7046589
TCEAL8	0,325279376	0,158148959
LOC729580	0,325279421	0,290102219
GFRA4	0,32532688	0,110589882
hCG_401283	0,325381005	0,067989145
LOC727813	0,325600111	0,67385325
T3JAM	0,325689934	0,043488247
HSPA1L	0,325895572	0,199011891
CDH11	0,325965794	0,631453438
LOC646522	0,326230263	0,089700513
H1FNT	0,326354944	0,371088387
PIP5K1A	0,326460011	0,526129673
IL22RA1	0,326529941	0,045070436
ZNF407	0,326589539	0,170916269
C14orf93	0,326720943	0,279786344
CPVL	0,32677135	0,121545956
ACBD3	0,326862554	0,127288719
PID1	0,327028883	0,105048364
C18orf62	0,327136068	0,943483975
SP8	0,327305988	0,418529942
LOC729154	0,327346032	0,145584162
FAM126A	0,327535876	0,01503058
PKN3	0,32753688	0,048490189
PMCH	0,32754178	0,690118276
MC2R	0,327930697	0,103099497
LIF	0,32798545	0,18104751
LOC653441	0,328007729	0,616013788
CCDC130	0,328079866	0,218498764
LOC731424	0,328154775	0,112241758
TPRG1L	0,328226016	0,330738566
PNLIPRP1	0,328460127	0,958966753
LOC731282	0,329085775	0,107896983
MYOZ2	0,329095331	0,126469421

MYT1	0,329161246	0,121828074
FAM153B	0,329278489	0,125079203
TCTE3	0,329340825	0,162475064
GCET2	0,32943167	0,22589929
ZNF439	0,329775545	0,148822493
MTMR7	0,329884093	0,283497822
CLRN3	0,330063853	0,248272737
NHLRC3	0,330239341	0,075968521
ARSH	0,330320299	0,209772103
LOC642574	0,330412844	0,630333821
IRG1	0,330616919	0,949448176
FRAP1	0,330694195	0,138848266
GRSF1	0,330780233	0,352783987
TMEM154	0,331014052	0,158615721
KIAA1486	0,331380676	0,075781776
PSMA3	0,331471343	0,277758481
MRPS7	0,331703324	0,653578887
LOC728815	0,331738213	0,292176702
CWF19L2	0,331739563	0,604158886
CDGAP	0,331897115	0,89252312
C6orf60	0,332073965	0,077047592
SCN2A2	0,332085119	0,520867464
LOC732432	0,33211077	0,146535929
GSTM4	0,332133543	0,40597072
LCT	0,332273175	0,187720459
C17orf60	0,332305294	0,137435582
FNDC1	0,332351726	0,124474166
MAGED2	0,33249046	0,146839468
EXOC1	0,332664904	0,283697672
MINK	0,332775312	0,109496383
IL1RL1LG	0,332909738	0,180572944
ITGB1	0,332929862	0,210698717
PLS3	0,33303363	0,389255812
LOC645529	0,333063815	0,12096471
CEPT1	0,333286069	0,089854653
OVOS	0,333359061	0,610692413
TDE2	0,333507801	0,35659582
PRKCE	0,33359496	0,546094411
P2RY6	0,333594991	0,131955796
LOC441251	0,333606531	0,044472759
C1orf56	0,333648877	0,288823861
GCNT2	0,333832315	0,69965808
ABCB5	0,333947075	0,700243832
GTF2IRD1	0,334148868	0,103886043
MITD1	0,334379194	0,370431656

LOC728119	0,334641191	0,480972947
KLPH	0,334754517	0,271475593
RTN3	0,335047376	0,512603422
PAIP2B	0,335204102	0,376483859
PTPNS1	0,335289792	0,27194096
LOC644682	0,335336947	0,214714761
VGLL3	0,335609539	0,744199397
TKTL1	0,335739311	0,708204638
LOC727801	0,335895447	0,218447888
EZI	0,335963575	0,373558755
LOC730065	0,336128679	0,143080811
ALS2CR8	0,336141054	0,547624625
SLIT2	0,336275693	0,053352476
PRL	0,336465189	0,831443322
ZC3HAV1L	0,33662545	0,326386128
DKFZp434C1418	0,336711022	0,701940533
MBD3L1	0,336758733	0,347766628
GBAS	0,336833038	0,167971993
S100A11P	0,336858781	0,253243811
ADCK5	0,336885593	0,292808658
FLJ10842	0,336896262	0,092930501
RIMBP2	0,336911891	0,317037298
LOC646644	0,336962593	0,876497286
TRAF5	0,33715613	0,095668234
DOPEY2	0,33724538	0,228693315
NS	0,33732362	0,270410051
LOC730242	0,337371597	0,16908199
C15orf17	0,337429001	0,423416336
AKR1C1	0,33746491	0,263642699
LOC653886	0,33747222	0,546094861
LOC644135	0,33755916	0,963941367
EML1	0,337595763	0,111187949
TTBK1	0,337803179	0,605998924
HEATR3	0,337820629	0,098203215
KIF2C	0,337864341	0,579493308
ANKRD44	0,338094648	0,136373691
HARSL	0,338286771	0,063067801
ALG6	0,338419921	0,842592983
GNG8	0,338545006	0,122449211
STRA13	0,338690481	0,878081388
AKR1A1	0,338848598	0,498107168
PGAM5	0,33906621	0,865466859
DKFZP586A0522	0,339136473	0,787677337
MRGPRD	0,339443985	0,3144987
DUSP14	0,339471035	0,180242071

LOC439992	0,339601069	0,473917985
STK38	0,339749969	0,175781858
REPIN1	0,339759814	0,137463063
SLC2A14	0,339919741	0,230670012
C1orf141	0,339969886	0,372522413
LOC286411	0,340313806	0,972981161
STX11	0,340461088	0,407593062
EPHA2	0,340517466	0,237140471
OR2T27	0,340569831	0,056589183
MS4A2	0,340715804	0,255806811
LOC220594	0,340766499	0,098111736
C6orf205	0,34082028	0,961364724
LOC641977	0,340851869	0,359475948
TTC19	0,340860292	0,156762439
LOC732410	0,34107448	0,274464789
ITGAD	0,341351306	0,472892451
C4orf23	0,341438018	0,685864664
IDS	0,341484218	0,143411986
PCSK6	0,341839323	0,644836954
CLRN1	0,342116463	0,161696566
PACRG	0,342127936	0,463606729
C6orf190	0,342208555	0,759520432
IGSF10	0,342366358	0,207904975
CYP27B1	0,342438711	0,083851407
UNC5CL	0,342448263	0,598782724
PDCD7	0,342516859	0,242722526
EGFL9	0,342574276	0,660932391
TAS2R13	0,342578102	0,552220491
LOC732458	0,342650491	0,175379642
MFAP5	0,342686167	0,408771672
DNAH9	0,342765715	0,207312962
CHRNA10	0,342818032	0,302738729
THOC2	0,342868911	0,673188691
PSCDBP	0,342892227	0,290282892
KIAA0753	0,342986531	0,239294287
FASTKD3	0,343025398	0,247984512
JHDM1D	0,34305311	0,458061102
LOC729675	0,343131144	0,146523009
CDKL3	0,3432231	0,859833245
LOC728322	0,34336865	0,5500299
E2F7	0,343374529	0,753653628
RAB3IP	0,34378341	0,56399219
BHLHB3	0,343790665	0,483210839
CNPY2	0,34400381	0,402290577
PTPLB	0,34404267	0,03592565

LETM2	0,344126046	0,329289067
MOX2R	0,344161338	0,102605658
VAX2	0,344167738	0,289519097
C8orf15	0,344237755	0,301569305
C14orf100	0,344370858	0,7355776
FAM180B	0,344408437	0,158840683
LOC729247	0,344644127	0,12517492
STAT6	0,344669167	0,066653161
MUTED	0,344972592	0,092395622
ZBED1	0,345027593	0,770172347
PNPLA10P	0,345050614	0,478335384
ASB7	0,345303218	0,166110121
UPP2	0,345317976	0,197228266
FOXL1	0,345579055	0,218733697
FCA/MR	0,345622895	0,090671051
CBX6	0,345623331	0,104683417
LOC732197	0,345662704	0,136515454
OR51I2	0,345707672	0,313214368
PTHLH	0,34587203	0,781094231
C6orf150	0,346222354	0,495420742
SOCS4	0,346613821	0,089736835
LOC730259	0,346622066	0,146246013
KIAA0436	0,346672658	0,193117184
PROZ	0,346685717	0,747815821
SLC41A1	0,346927271	0,790405847
TMEM25	0,347044218	0,132324303
LOC730049	0,347291815	0,103189314
PIK3R1	0,347484982	0,391992761
BTBD9	0,34792268	0,436619386
WIF1	0,348182747	0,965137935
TNFSF4	0,348454479	0,083765603
IL17A	0,348797121	0,93739408
NRK	0,348991037	0,082937831
RAMP2	0,349098549	0,228891129
KLEIP	0,349108778	0,712076415
OR4D9	0,349238455	0,154182891
HOXB9	0,349334402	0,218851074
LOC116166	0,349478448	0,530441159
LOC729005	0,349625683	0,87967383
CEP70	0,349640199	0,194844854
ETFDH	0,349665221	0,185485316
TAF2	0,349893106	0,559525451
LOC728764	0,349896526	0,68891289
PHLDA3	0,350022307	0,919358957
C2orf74	0,350104501	0,150392186

KRT23	0,350109238	0,327815208
DPYSL2	0,350165341	0,18632215
SHISA2	0,350182389	0,066606962
TMEM16E	0,350270708	0,336873381
GUCY2C	0,350399217	0,414676841
ALB	0,350592409	0,771775692
FLJ22054	0,350857454	0,243034002
GPR75	0,350866816	0,717923575
NUDT16	0,350968784	0,195497498
HIST1H3F	0,351019845	0,124846263
TBCCD1	0,351111516	0,598744442
ZNF860	0,35123392	0,052251081
SECTM1	0,351299626	0,076353719
HSA9761	0,351360099	0,641998914
LOC339766	0,351548803	0,174112701
CDK5RAP2	0,351679735	0,67745083
ZNF70	0,351695933	0,525270961
ARHGAP9	0,351802768	0,221211882
SOX1	0,351808253	0,863057914
TUBB4	0,351871313	0,298546366
MS4A4A	0,351894557	0,239383342
PIWIL1	0,352055244	0,230197085
KIAA0882	0,352125111	0,155841004
ADAM15	0,3521916	0,294767684
C15orf58	0,352192859	0,194278542
CCHCR1	0,352643865	0,200077739
EIF4G1	0,35286105	0,116975292
LOC201140	0,352933705	0,214336004
ERMN	0,35310068	0,086823767
SETDB1	0,353139275	0,08860238
CMYA5	0,353220431	0,690399285
LOC642731	0,353707591	0,034548458
LRFN1	0,353764721	0,224987193
FKBP2	0,354044895	0,318704237
CARM1	0,35405646	0,191345711
B3GAT1	0,354106978	0,798585734
LOC284067	0,354135143	0,96509707
PTPN22	0,354193355	0,617334749
ZNF83	0,354200763	0,164451098
XRCC1	0,354232875	0,280849807
LOC644660	0,354437913	0,167120809
RBM10	0,354528754	0,573547341
UGT1A6	0,354552187	0,12706643
GPR18	0,354562339	0,068651833
LOC647795	0,354690501	0,654270782

CALB2	0,354732111	0,996537947
LOC729393	0,354778454	0,496400491
LOC440900	0,354928438	0,147999324
KIAA0831	0,355016124	0,259382655
ERO1LB	0,355485479	0,335951216
LOC728868	0,355488809	0,983938887
LOC728040	0,355608536	0,439042978
LOC732024	0,356165122	0,582126072
ZNF585B	0,356170906	0,167393879
DEFB107A	0,356281598	0,286156414
LOC729010	0,356401372	0,032284353
NPEPPS	0,356504747	0,541066838
RP11-11C5,2	0,356665031	0,388645293
LOC642335	0,356738935	0,666835473
AOX1	0,356834648	0,104166736
KIAA0133	0,356886331	0,255551027
ZC3H12B	0,356909485	0,522240605
TTC22	0,357016016	0,352502431
APOO	0,357085947	0,286443323
FLJ21511	0,357209779	0,175097624
CIAPIN1	0,357296038	0,948527508
FLJ12528	0,357309414	0,574999605
CT120	0,357405236	0,350012467
KIAA1586	0,357505456	0,151365743
KIAA1627	0,35755246	0,028611082
ACO2	0,357706688	0,049854767
RABL4	0,357883534	0,042724005
LOC647011	0,358035046	0,385175285
PEX11G	0,358158033	0,732511562
FLJ31547	0,358277721	0,275884267
ASB9	0,358556733	0,365485304
CEBPZ	0,358571754	0,270818379
NBPF7	0,358576276	0,34933011
SLC39A8	0,358897993	0,274866929
LOC338586	0,358930278	0,195176465
PRES	0,358963318	0,064690381
LOC729049	0,359183803	0,240960757
KRTDAP	0,359216358	0,130956761
C2orf63	0,359221342	0,676268356
LOC727912	0,359241257	0,091777506
FBXL3	0,359362551	0,089382561
SVEP1	0,359374609	0,330575322
DHRSX	0,359399571	0,024980194
LOC644065	0,359513522	0,374791448
NFKBIB	0,359718884	0,021938913

PTCD2	0,359941815	0,355052146
PLAU	0,359988379	0,776893755
LOC645206	0,360239287	0,896807379
STK17B	0,360383194	0,035635113
HDGF	0,360501433	0,14047573
ZCCHC6	0,360629186	0,130085743
RPL18	0,360641108	0,501243877
LOC728050	0,360906434	0,200406089
IKBKB	0,361070323	0,078819092
ZNF770	0,361119888	0,712701424
BEGAIN	0,361169468	0,135431186
CCDC8	0,361223825	0,204086432
AGL	0,361308411	0,34066453
ZP1	0,361324578	0,179945806
C9orf41	0,361474529	0,117181318
LOC441996	0,361526925	0,698008894
KCNA2	0,361649931	0,033276725
MAP4K1	0,361695675	0,75000876
JMJD2C	0,361748408	0,801917042
SLC35F5	0,361831505	0,280047756
FLJ33360	0,361861071	0,250216504
FRMPD3	0,361871879	0,604582041
EEPD1	0,36188754	0,117853733
LOC728480	0,362051924	0,001347034
GNG11	0,362163975	0,183576646
TMEM120B	0,362517883	0,793784284
CBX4	0,362709525	0,37568196
ADAM7	0,362856479	0,42418927
CGN	0,362875611	0,923452909
LBP	0,362982646	0,959003464
HSF2BP	0,363101245	0,08181968
CIB4	0,363141446	0,190628669
IL17B	0,363163308	0,206954738
SLC6A13	0,363205479	0,141845797
C1orf163	0,363266585	0,345385388
UIMC1	0,363269726	0,010059354
C9orf20	0,363511627	0,104683377
KRT80	0,363531108	0,160586356
TMEM132A	0,363858504	0,214450622
LOC729822	0,363914184	0,155961438
RAI3	0,363917811	0,964995532
C1orf87	0,364230039	0,332696328
GGT1	0,364390653	0,390676235
hCG_1983058	0,364679183	0,103240048
ATP2B3	0,364997274	0,584778439

ZNF578	0,36502946	0,990043533
LOC728387	0,365405132	0,120019422
DPRX	0,365405477	0,541051835
LZTR1	0,365541663	0,440005024
LOC729644	0,36559681	0,877630184
JAKMIP1	0,365664545	0,420580812
ZNF534	0,365695831	0,166874149
LY6D	0,365746879	0,094700039
SPRYD4	0,365753607	0,30222521
QRICH2	0,36588907	0,5644067
PHF7	0,365918014	0,638722824
SLC2A3	0,365954192	0,319616856
C12orf11	0,365987895	0,242717159
LOC400099	0,366119279	0,088141081
MCC	0,366218362	0,466442548
SLC22A25	0,366309907	0,102506475
DGUOK	0,366414686	0,468945286
ZNF432	0,366432672	0,85021364
LOC730821	0,366706545	0,371675364
KIF3B	0,366831129	0,105866945
APOBEC3A	0,366914677	0,31021384
NLRX1	0,366929585	0,307846779
BCL7B	0,367084096	0,879322739
LOC347487	0,367372193	0,492665354
MNT	0,367431807	0,68169322
CXorf1	0,367661714	0,121991808
PACS1	0,367680848	0,100937273
LOC729940	0,367799273	0,334426584
ZBED2	0,36785062	0,062169268
C17orf62	0,3678888	0,816101264
ATG12	0,367905915	0,757946488
ZNF584	0,36804249	0,344726963
GNRH1	0,368065	0,143637802
C14orf126	0,368195176	0,136966615
CDC20B	0,36879548	0,73004394
C6orf182	0,368828242	0,928141014
IRF6	0,368829816	0,188865259
VEZT	0,368833677	0,287401901
C4orf6	0,369063188	0,418938064
SPOCK3	0,369147283	0,312320126
SCAND2	0,369294487	0,278160559
C8orf44	0,369361862	0,231720048
TMBIM4	0,369371031	0,19754554
GLUL	0,36937504	0,080526315
LOC200493	0,369433602	0,935046169

PALB2	0,369448824	0,041274693
DNAJC11	0,369476974	0,253960421
GRIN2B	0,369657805	0,707952189
MRPS16	0,36969549	0,371546436
FRMD5	0,369705353	0,1496222
NAB2	0,369816991	0,345531223
NDUFS5	0,369854761	0,147800419
FER1L5	0,3698764	0,073362286
CAMK2N2	0,369996191	0,40223475
PPP1R3C	0,370426747	0,393289816
PHF15	0,370449558	0,956010924
MYO9B	0,370461848	0,164650417
GPR2	0,370629833	0,296008163
PDE6D	0,370743273	0,310714681
C7orf10	0,370745087	0,186006635
NKAIN1	0,370866015	0,379502542
SELS	0,370872527	0,242160452
ZNF497	0,371121232	0,501572646
ZMYM2	0,371148259	0,940184391
SHISA3	0,371312924	0,046135316
MAP1LC3C	0,37140063	0,566196972
LSM12	0,37142679	0,227641684
JPH2	0,371431687	0,320682041
CST2	0,371434283	0,12897806
SCAMP1	0,371445542	0,369934738
DDX10	0,371451269	0,247539005
ZCCHC17	0,3716719	0,907598279
HPCAL1	0,371951732	0,517598903
hCG_2025063	0,372022519	0,029524
SLC24A5	0,372112632	0,335091224
SOAT1	0,372160582	0,480644119
CCDC147	0,372238598	0,087784852
CNDP2	0,37224239	0,071138812
LOC647979	0,372274394	0,831915934
SH2D4A	0,372624884	0,136866044
ANXA1	0,372672319	0,090075652
LOC730647	0,372750726	0,085186548
CCDC86	0,372896688	0,13674743
ZFY	0,373007088	0,888470918
CRYZL1	0,373007669	0,620740067
SFRS6	0,373196143	0,286933826
LOC644749	0,373205081	0,5
LOC731292	0,373531713	0,26965682
FLJ40288	0,373763778	0,145857024
H2BFWT	0,374188443	0,203606964

SLC16A13	0,374399139	0,962639465
UST	0,374476748	0,06719074
UNC13A	0,374543148	0,343087193
CREM	0,374546504	0,427344352
GPR152	0,374614114	0,992614956
TTC23L	0,374697814	0,682598593
WNT10A	0,374860992	0,201426028
P2RXL1	0,374962586	0,140660235
PLEKHB2	0,375168584	0,604701995
VSIG6	0,37519279	0,530195623
LIPF	0,375611707	0,27988379
C11orf51	0,375713371	0,021714641
ITM2B	0,375740275	0,947944935
CKS2	0,375830781	0,08920379
ZCWPW1	0,375929808	0,067130132
LOC731734	0,375974942	0,157936416
CFP	0,376050773	0,651716078
ACSL5	0,376132223	0,504682263
LOC732306	0,376175092	0,158408941
KCNIP2	0,376281037	0,124021115
SFRS16	0,376417578	0,1905773
LOC51321	0,376465079	0,149975117
KCNF1	0,376565205	0,713604734
TCF15	0,376578039	0,115265988
STK10	0,376822117	0,023587539
ZSWIM6	0,376831812	0,561117066
Kua-UEV	0,37684222	0,194485159
MLLT10	0,376870578	0,772721869
KRT126P	0,376890363	0,151753171
NTRK2	0,376979024	0,19166157
LRRC14	0,377099134	0,9418483
LOC731350	0,377420216	0,306526508
PRKACB	0,377503193	0,17797004
MUCDHL	0,377536639	0,404766806
UBXN2B	0,377664474	0,691080833
LOC729626	0,377739975	0,630993348
SHISA4	0,377758179	0,524770502
HIP2	0,377772559	0,18798124
LOC342346	0,377882249	0,160810107
ALDH1A2	0,378763461	0,215303588
DNAJB9	0,378893714	0,995334736
TEAD3	0,378907381	0,096072416
BAIAP2	0,379139092	0,208344865
RALBP1	0,379366187	0,287484554
TJAP1	0,379367544	0,174879312

JMJD5	0,379582505	0,130594774
ACSF2	0,379751442	0,312857453
F13B	0,379999358	0,406062752
MGC71993	0,380253478	0,378030331
LOC644662	0,380315202	0,760470543
LOC732392	0,380504827	0,280853735
RAB32	0,380696583	0,099522282
FLJ22761	0,380921588	0,773724708
C9orf125	0,380973322	0,167173811
KIAA1755	0,380996613	0,684151474
FLJ20021	0,381080195	0,44093773
CYP2E1	0,381119231	0,483159392
LOC729378	0,381178827	0,098827289
GLDC	0,381193639	0,395803253
C19orf52	0,381209203	0,133097598
AMPD1	0,38123201	0,801109281
GNPDA1	0,381250977	0,014229089
NUPR1	0,381299736	0,044117288
LOC731158	0,381455925	0,537700126
LOC643933	0,381457232	0,087853497
ZNF42	0,381470595	0,655349866
SH2D5	0,381474645	0,71490063
RPP38	0,38167166	0,92795203
TPRKB	0,381752139	0,193511866
FAM83D	0,381784272	0,84484341
SLC5A2	0,381939669	0,657213804
AP4S1	0,382171464	0,101165551
LOC440776	0,382220809	0,288569968
JTV1	0,382342075	0,122209176
CSTF1	0,382431471	0,382393537
DEPDC7	0,382619693	0,32867008
RSPH1	0,382967899	0,345323357
ADRB2	0,383368386	0,239324899
MetRS	0,383381291	0,205741038
CCDC56	0,383415542	0,008637532
NOVA1	0,38375222	0,310857061
PCDH19	0,384004845	0,367795786
LOC728999	0,384219691	0,245776002
TRAM1L1	0,384572412	0,170147897
LYPLA2	0,385410263	0,867001648
CLTC	0,385699808	0,293205073
OR4L1	0,385880917	0,511707714
PDHB	0,385991494	0,300146544
RABL2B	0,386066029	0,039639917
TIPRL	0,386089485	0,060605432

LYN	0,386108722	0,705428687
SLC2A1	0,386175125	0,075372459
CCDC24	0,386216033	0,109435697
CTPS2	0,3862319	0,180602928
MANSC1	0,386319708	0,211512354
LOC492311	0,386643584	0,707934714
ABCD1	0,386769511	0,018981896
HOXA3	0,386848898	0,109247431
C8orf82	0,386878263	0,34862614
OGFR	0,387483744	0,096706974
MRPL22	0,387491154	0,258750256
MT1H	0,38752918	0,335198332
GGNBP2	0,387613679	0,117840347
TBX15	0,38773048	0,446028173
DTWD2	0,388092496	0,037503882
CDH4	0,388194661	0,509156428
MMP28	0,388251619	0,236011778
CCDC111	0,388668879	0,158926738
LOC648728	0,388927912	0,056193924
FAM73A	0,38910733	0,497416417
CABP5	0,389160109	0,398341203
KRT13	0,38919061	0,178487385
C15orf42	0,389519099	0,172695666
TGM2	0,389573966	0,221923953
LOC731117	0,389649908	0,180875354
LOC644285	0,389731408	0,734129205
LOC729934	0,389853007	0,240154143
LOC727857	0,389905808	0,142029501
OLIG1	0,390002683	0,175163889
ADIG	0,390070736	0,19616366
ZNF482	0,390254083	0,870679402
FADS2	0,390280556	0,085444894
STAG3	0,390283167	0,205207973
TBX10	0,390447221	0,042994217
CCDC48	0,390515353	0,851985248
NRAP	0,390530939	0,390255551
PLEKHG4	0,390535557	0,657302976
MYD88	0,390579364	0,131587285
POL3S	0,390671873	0,232650176
LIG1	0,390682613	0,838285887
LOC728254	0,390751708	0,137508239
LOC644949	0,390891865	0,139521684
F8A2	0,391001444	0,423892054
SLC38A3	0,39126029	0,053348034
TMEM194	0,391433799	0,039982518

LOC642483	0,391447837	0,110082323
YTHDC2	0,39145262	0,201828763
TUBB1	0,391469376	0,149177443
SPRED2	0,39155247	0,085580677
ZNF228	0,391916531	0,10796864
C2orf82	0,391944758	0,037321577
CENPP	0,392103902	0,123295594
SLC29A4	0,392137687	0,203427693
LOC729294	0,392354121	0,106022948
DUSP23	0,392372137	0,128444051
LOC390282	0,392489006	0,135580271
ZNF238	0,392657839	0,332691312
LOC732034	0,392809814	0,228712895
PWWP2B	0,393220978	0,123807515
C11orf85	0,393252733	0,135528692
LOC729302	0,393290255	0,394558344
WFDC13	0,393308783	0,726842719
PCDHA7	0,393523044	0,115368721
AASDHPPT	0,393594353	0,233479065
CHKA	0,393603912	0,317769846
TYR	0,393654425	0,437991969
LOC645730	0,39365586	0,05073997
LOC728345	0,393692125	0,288108515
LOC644026	0,393757241	0,693100165
HMGCS1	0,393802729	0,187289278
LOC728838	0,393823481	0,086728608
OXTR	0,394100931	0,250221523
LOC645137	0,394227838	0,446331763
FLJ30626	0,394362426	0,239273728
DSC2	0,394548549	0,105389042
CLEC12B	0,394703067	0,754040038
AKR1C4	0,394738488	0,17507327
MAPK7	0,395053392	0,443377769
KIAA1549	0,395461473	0,2037698
IL27RA	0,395539596	0,524372856
RAB6C	0,395919906	0,477326222
PRR14	0,395926416	0,24317942
NIPSNAP1	0,395966165	0,787166648
WNT2B	0,395987974	0,387830847
CNTN3	0,396036284	0,58169488
RCV1	0,396184425	0,252004323
TCF7L2	0,39626951	0,866876631
C14orf133	0,396390669	0,134363623
HSPC150	0,396651879	0,031731783
LOC645979	0,396943345	0,203973299

HIC1	0,397176907	0,148815234
FKBP15	0,397195573	0,231211263
CSNK1D	0,397306513	0,504344455
LOC731347	0,397572777	0,482893918
BCL6	0,397942564	0,589989539
CABP1	0,397993355	0,179033978
FSTL3	0,398001837	0,172866793
GPR83	0,398113824	0,588478175
CXorf30	0,398346361	0,856175793
DDX49	0,398375548	0,468498473
EDN2	0,398506336	0,101842731
LOC653097	0,398516704	0,152960906
TXNDC5	0,398608415	0,123081537
LOC728600	0,39862491	0,14416068
SDC4	0,398685596	0,2342058
MFI2	0,398711376	0,134704929
UBE1	0,398724765	0,655733324
CABP7	0,398770373	0,099834336
DCTN1	0,39880566	0,521630836
OR5L2	0,399260247	0,511161196
KIR3DL3	0,399428078	0,181942672
PCTK2	0,399436042	0,385148114
PLEKHO2	0,399455171	0,081472783
LOC730231	0,399672329	0,8226505
GCHFR	0,400342727	0,379269597
PPP1R3B	0,400452322	0,241479516
C12orf5	0,400485395	0,808865297
VANGL2	0,400755286	0,238638354
ZCWPW2	0,400912466	0,020027958
KIRREL2	0,401040936	0,131090307
HADHB	0,401154417	0,219539138
HRMT1L1	0,401329377	0,189203354
ACD	0,401392041	0,177812339
hCG_1640785	0,401650192	0,111931221
CYP2C18	0,401750788	0,534716287
FLJ45455	0,4017752	0,299752694
C8orf74	0,401874747	0,102212793
P2RY1	0,402082443	0,112758061
FPR1	0,402210025	0,088716124
CEMP1	0,402251194	0,310976072
GRLF1	0,402329418	0,724014016
LIPH	0,402484929	0,712114828
LRRC56	0,402488184	0,375656115
LOC731310	0,402536566	0,057129848
RALGPS1	0,402555948	0,058281111

HMGN2	0,402745862	0,514308478
LOC729808	0,40275471	0,31689016
SCGB1D4	0,402830789	0,140201124
UPK3B	0,403028756	0,772031834
GGPS1	0,403066057	0,552286198
KLF7	0,403085457	0,249063144
ZNF101	0,403132395	0,065112664
TSC	0,403173077	0,073419845
IFT52	0,403450662	0,104000542
ATP5G1	0,403816314	0,813060206
SLC26A8	0,403863968	0,147028231
GLI	0,403872557	0,17493666
C3orf1	0,404190894	0,089024208
KLHL13	0,404239878	0,154529909
LOC729411	0,404246159	0,491796275
LAYN	0,40477372	0,051980027
SLAMF7	0,404777518	0,240014967
TPH1	0,404883359	0,273715822
LOC730279	0,404907666	0,085003749
LOC342994	0,405038448	0,272530805
PTK7	0,405122313	0,241246539
LOC730159	0,405142131	0,637083466
FLJ25770	0,405194782	0,100080778
LOC727974	0,405206088	0,069845157
FLJ22184	0,405245881	0,199588788
LOC285484	0,405331218	0,196664837
ZIM2	0,405332254	0,018583038
URG4	0,405340603	0,005630386
RNF165	0,405378895	0,08255247
SETD3	0,40544339	0,22519767
PRRG1	0,405637918	0,306658126
LOC730167	0,405758292	0,796164894
MTRF1L	0,405862335	0,48308076
LOC650412	0,405919921	0,719287399
ENPP7	0,406121085	0,112513524
BAT4	0,406507443	0,207630546
COMTD1	0,406809895	0,077238287
FAM132B	0,406967382	0,543208316
C12orf53	0,406967909	0,171391507
FAM173A	0,407016442	0,23175846
LOC442448	0,407114423	0,248489802
CDC7	0,407196201	0,156435865
HPS4	0,407326409	0,137535135
ZNF317	0,407382038	0,148214655
KRT28	0,40744346	0,17152231

STOM	0,407732535	0,086602082
CHRNA9	0,407844933	0,07597323
PAMCI	0,408229103	0,133699199
ITGA5	0,408740743	0,029690015
HMOX1	0,40885477	0,26162708
HRMT1L4	0,409051414	0,558142587
SLC25A12	0,409096762	0,303784034
LOC643287	0,409157501	0,087362946
KIAA1274	0,409233799	0,572106789
TMOD4	0,409757265	0,427085961
LOC728328	0,409981498	0,039425437
LOC728145	0,410115125	0,05696765
OVCH1	0,410248335	0,292804707
LOC650751	0,410256708	0,420568162
MKNK2	0,410391883	0,299952024
EDG1	0,410409354	0,477589673
BTEB1	0,410534968	0,810558441
OR5B17	0,410592223	0,103932423
NKIR	0,410781585	0,108174027
LOC729560	0,410826511	0,43444452
SMARCA5	0,410837617	0,064520168
FAM21B	0,410951363	0,563247963
LOC642980	0,411444133	0,835098644
HSD17B4	0,41147915	0,821423878
LOC283547	0,411742142	0,158718146
ATP5B	0,411987129	0,075809865
RQCD1	0,412140848	0,171029683
LOC645650	0,412763441	0,125263472
FTMT	0,412814893	0,965045964
LOC642452	0,413004379	0,061407778
MMP3	0,413050588	0,427899908
LOC283480	0,413113533	0,507089644
IFT81	0,413203414	0,07859739
OLFML2A	0,413222419	0,210608918
DCBLD1	0,413430674	0,168544953
FBXL13	0,413492025	0,820785045
IFIH1	0,413508118	0,37924692
KRTAP12-2	0,41351326	0,277210234
GSTA2	0,413535632	0,523900392
CASP8	0,413629634	0,303926803
RP1-14N1,3	0,414105422	0,207847849
FKSG83	0,414125896	0,137721603
MECP2	0,41432724	0,306953651
OTOR	0,414422202	0,091287902
COL19A1	0,414627246	0,335066777

C20orf114	0,414826559	0,171265203
LOC730795	0,41502368	0,483135637
LOC441120	0,415349849	0,049037446
ARL2BP	0,415781741	0,370148968
C15orf55	0,415834628	0,311359242
C7orf31	0,416254745	0,281071424
CLDN10	0,416319889	0,791964251
C1orf140	0,416350088	0,259933954
TBC1D2B	0,416666993	0,068670892
LMO1	0,417296307	0,461144454
RFC3	0,417714722	0,209278934
PAK4	0,417884318	0,543601207
LOC255130	0,417922843	0,242849583
LRRC16A	0,418271053	0,162425257
ANXA10	0,418284302	0,078544416
C17orf57	0,418473494	0,148858456
PEX1	0,418590362	0,044970892
GPR52	0,418691952	0,875943623
WASF3	0,418692548	0,972661498
VASH1	0,418746957	0,337811752
THSD1	0,418923056	0,170719843
HPRP8BP	0,418940008	0,100024679
SRI	0,419096855	0,415168435
C1orf116	0,419228099	0,06488535
SHISA5	0,419370672	0,444448913
SAMD9	0,419728175	0,017896887
LOC151174	0,419942828	0,861774165
PCYT1B	0,41997007	0,215218786
STK16	0,420025022	0,345023275
HJURP	0,420078065	0,318468057
C19orf45	0,420132042	0,018666233
ZNF703	0,420261924	0,268716601
GEMIN6	0,420316363	0,196517124
GAB1	0,420399983	0,812786611
LOC400451	0,420420774	0,289119063
IL18BP	0,420545301	0,415733145
HIST2H4A	0,42084089	0,176127527
APOL4	0,421021085	0,270517467
LOC729795	0,421060831	0,136925695
GBX2	0,421245125	0,183942879
MUDENG	0,421704253	0,543384128
TNFAIP6	0,421929662	0,116060186
C3orf33	0,422234134	0,109936812
CCDC85B	0,422645817	0,409535403
STRN3	0,422870865	0,306100042

LOC402635	0,423014987	0,302786865
SLC35E2	0,423034665	0,096682391
DBN1	0,423369647	0,67474061
SULT2A1	0,423381172	0,639227493
LOC728774	0,423455493	0,192476478
KRTHB1	0,423496582	0,192777288
LOC728276	0,423594177	0,303755703
NCAM1	0,423690162	0,454402508
PP591	0,42379088	0,063050686
FBXL10	0,423993637	0,62184855
TEKT3	0,424079504	0,148623865
C5orf30	0,424115741	0,092239311
OR6Y1	0,42428607	0,271960092
RPL13	0,424435097	0,199085366
KRTAP1-3	0,424440451	0,057899245
LYPD6B	0,424508072	0,167545064
ACOX3	0,424525297	0,261806527
GATA1	0,424615697	0,117878625
LOC729846	0,424775387	0,009653568
LOC391025	0,424781165	0,241241883
PRDM15	0,425170898	0,252772079
AKAP13	0,425253225	0,561996152
ROCK1	0,425269526	0,691596694
C6orf66	0,425314798	0,6145524
LOC646260	0,425379791	0,78655248
SLC39A13	0,425420709	0,587122961
CDC42EP3	0,425576333	0,139691063
LOC90874	0,425650586	0,21155395
LOC441452	0,425786453	0,611633473
ATP5G2	0,425798755	0,855668781
LOC729749	0,425801953	0,066049271
TMEM14C	0,425939894	0,227292304
LOC728508	0,426067117	0,123306346
PPP1R3A	0,426583896	0,231327215
VIAAT	0,426604885	0,503761433
LOC340654	0,427056118	0,243539094
IVNS1ABP	0,427064495	0,241819107
RIPK3	0,427530544	0,468635671
CLDN2	0,427640966	0,21505903
OAS2	0,427969847	0,231616365
OR10K1	0,428134957	0,411052554
LOC729124	0,429266607	0,121478929
RBMS2	0,429270707	0,10590285
PHC1	0,429558245	0,288867624
IL8RA	0,42983272	0,013069452

MPV17L	0,429853219	0,802332895
ARMCX2	0,430124351	0,284246092
FKBP8	0,430151629	0,419229245
C12orf36	0,430247908	0,677830734
PTAFR	0,430291117	0,42658804
LOC727911	0,430324237	0,162845405
CBR1	0,43046643	0,041922177
TREML3	0,430670557	0,161923297
FLJ14345	0,430704989	0,142078856
LOC341112	0,430742158	0,043140162
GNPDA2	0,430801582	0,737115174
DNAJC4	0,430861129	0,331867197
IL5RA	0,43087045	0,451249281
UNC5D	0,431013277	0,076801982
C1orf101	0,431420586	0,162601908
ZNF469	0,431519175	0,066564019
TACR2	0,431525579	0,108456876
ZNF595	0,431564056	0,609703301
LHFPL1	0,431585176	0,295920483
THSD3	0,431597535	0,013251693
RARA	0,431904581	0,137632342
COQ4	0,431943824	0,1594981
KA36	0,432052997	0,277619659
LOC730119	0,432182612	0,262988054
C17orf51	0,432568244	0,022355413
LOC645136	0,43262328	0,282104899
C17orf75	0,432756462	0,595002284
G6PC3	0,432760137	0,041913577
CDO1	0,432808643	0,215505183
INPP4A	0,432944319	0,414696998
MCM2	0,433150581	0,134189358
LOC647869	0,433161063	0,032545094
LOC732370	0,433167871	0,139951794
SPTLC2	0,433342028	0,296124276
PPARA	0,433404852	0,126836672
DCL-1	0,433437532	0,245871259
SSX5	0,433467973	0,09233904
DNAJA3	0,433607189	0,772721208
LENEP	0,434105258	0,756156447
GPR58	0,434282512	0,262879322
APLP1	0,434362067	0,190296088
M6PR	0,434707745	0,500374395
RWDD1	0,435187902	0,054826477
PRDM7	0,435232045	0,272434853
PRH1	0,435346746	0,22740433

UBXN10	0,43546436	0,427254035
ABCC8	0,435718092	0,407177605
DUOXA1	0,435718248	0,8226945
COL2A1	0,435748357	0,301159816
TLR7	0,43574922	0,187695192
DMXL1	0,435753951	0,370177521
C14orf180	0,435772706	0,245801666
RAB11FIP2	0,435825357	0,241949608
C1orf151	0,43600027	0,108604418
LOC388282	0,436142796	0,152210441
LOC644670	0,436405488	0,972992707
STRA8	0,436623128	0,5
Rgr	0,436715862	0,183414452
GPR51	0,436815371	0,25803375
KIF13A	0,436994001	0,098306172
GLT1D1	0,437243268	0,064000934
C6orf134	0,437616292	0,147361826
C8B	0,437751858	0,749039704
KCNC3	0,437859129	0,257944681
CDKN1A	0,437966479	0,497999382
CLDN12	0,437989414	0,276139221
PCDH16	0,438071305	0,437403567
ZNF318	0,43813467	0,01682076
LOC645685	0,438208981	0,014259916
LOC728968	0,438640294	0,426583486
C22orf30	0,438718004	0,12569211
CTGF	0,438955272	0,868257919
KCNJ11	0,439079468	0,252081094
LGR8	0,439420898	0,225968027
AFM	0,439641915	0,212778112
DCAMKL1	0,439907962	0,087437125
LNP1	0,44000507	0,356503066
PHF13	0,440301494	0,240221955
GRK1	0,440314853	0,50275214
LOC727827	0,440452813	0,378885286
KCNS3	0,440458397	0,444557071
TAF6L	0,440629748	0,554595547
XK	0,440791966	0,636528647
LOC391764	0,440991491	0,350186062
PLSCR3	0,441559892	0,972189344
GPR139	0,441705188	0,10256792
MTHFD2L	0,44179702	0,342911893
TAF11	0,441900355	0,14927879
SCARB2	0,441937272	0,232879492
LOC729529	0,442026581	0,095256227

LILRA1	0,442081229	0,517766188
OR4D5	0,442208223	0,751424765
TMEM174	0,442307602	0,217019723
SLC10A3	0,442674718	0,211659134
MGC45780	0,442829362	0,081781545
OR9A4	0,44293159	0,313110057
GDPD2	0,443131686	0,107841702
ATP6V0C	0,443515761	0,079860085
ACP5	0,443783277	0,015497764
NDUFA4	0,443854699	0,090042651
MMP11	0,444083455	0,513790116
SPATA16	0,444091191	0,449105151
ZNF323	0,444136335	0,351288433
LOC730033	0,444139179	0,087783508
LOC651281	0,444167254	0,575862342
LOC730474	0,444296092	0,918486375
LOC643281	0,444336865	0,120405161
XPR1	0,444420468	0,176231726
ABCC3	0,444441796	0,192339817
NKX3-2	0,444541355	0,177189869
SYCP2L	0,444568751	0,103917022
OR51L1	0,444577985	0,367620861
RPS28	0,444769974	0,155063992
SIN3B	0,444874216	0,245864171
GMDS	0,444909725	0,435582636
CHSY1	0,445035768	0,008880552
LOC93432	0,445400135	0,106775009
ELP3	0,445424603	0,272670423
PKIA	0,445483568	0,246996176
PLEKHO1	0,445562387	0,318489699
RNF151	0,445738028	0,101538611
GNLY	0,445791107	0,070225248
NIP30	0,445933166	0,222017666
LOC728877	0,446059003	0,103519058
LOC92906	0,446175447	0,224955231
DHRS6	0,446296082	0,106048309
COX8C	0,446712769	0,501628767
DPY19L3	0,44686642	0,742845887
EI24	0,446970592	0,241267373
NIPBL	0,446992481	0,185454412
WWP1	0,447021711	0,211912572
LOC729556	0,447041971	0,654517779
SP100	0,447122588	0,379386858
FAM54A	0,447161706	0,648692988
FAM164A	0,447202992	0,088443219

TOP	0,447229213	0,061831841
FDPS	0,447367121	0,494060373
CCBL1	0,447384359	0,27812062
CMTM8	0,447860788	0,25503741
AFG3L2	0,447962797	0,137514819
FLJ37638	0,448325847	0,089966937
CMIP	0,448563995	0,661999523
SULF2	0,448569529	0,506298028
FAM125B	0,44859671	0,105609567
C16orf65	0,44867458	0,074746331
ANKRD20A3	0,448860538	0,172153978
TTC32	0,44893373	0,213833522
LOC652787	0,449228046	0,067845686
FRZB	0,449351867	0,240621582
OR6C68	0,449374552	0,643918403
KCNQ2	0,449405912	0,933570846
LOC440917	0,449573538	0,664394675
FAM91A1	0,449950066	0,266398264
SLC25A17	0,450129522	0,350810304
FGF17	0,450340413	0,06292342
LOC642278	0,450386687	0,184224714
LOC442287	0,450398863	0,433791815
LOC728183	0,45042132	0,220879987
SUPT6H	0,450440595	0,490378917
AMY2A	0,450509786	0,265022295
LOC388795	0,450586411	0,519838389
LOC729206	0,450614813	0,370916179
NRBF2	0,450794592	0,112566173
CDCA3	0,450891764	0,584798712
CASS4	0,450951417	0,014832287
OR4C11	0,450964661	0,973393873
LOC732474	0,451138487	0,488633199
PCSK1N	0,451177225	0,154491746
ZNF85	0,451435143	0,313638039
BRF1	0,451693961	0,041028282
SLC4A4	0,45171513	0,631902543
MAP3K10	0,451724373	0,698368804
CROCC	0,452137169	0,108464592
EYA3	0,452296039	0,233228963
UHRF1BP1L	0,452318319	0,055786
PSMA5	0,452421949	0,364273456
C16orf14	0,452467407	0,957072645
WDR82P1	0,452555833	0,278268365
SLC7A1	0,45296391	0,112122181
TEK	0,453048567	0,262739688

ODC-p	0,453490633	0,088744144
AQP4	0,453679513	0,884698679
PDHX	0,453696842	0,141731824
C8orf13	0,453857592	0,214757218
EFCBP1	0,45418576	0,045384111
LOC390414	0,454199354	0,113299858
RDBP	0,454259973	0,071496672
MRPL35	0,454331254	0,216907755
P76	0,454351038	0,250694707
TGM6	0,454355529	0,112709636
TMEM126B	0,454488637	0,119048584
GLRA1	0,45449175	0,105811792
PPOX	0,454524919	0,151449641
TRAR4	0,454746459	0,876798194
KIAA1462	0,454811568	0,885155702
HERC6	0,45498667	0,292056697
GPR92	0,455261605	0,137876097
C1orf174	0,455278771	0,058520251
PSKH1	0,45571308	0,184892582
SRF	0,456034314	0,184922983
LOC728983	0,456332475	0,24493066
LOC651713	0,456486804	0,100055189
C10orf95	0,456643276	0,248061414
ACPP	0,45697862	0,961826554
FAM35A	0,457328789	0,510901222
C21orf58	0,457419882	0,016565791
GSTM3	0,457550895	0,700766205
GOLPH3	0,457773007	0,042236857
LOC647016	0,457886889	0,103473872
USP52	0,457979577	0,171721198
CDT6	0,458082098	0,199123074
LOC729645	0,458469538	0,032507901
AGA	0,458530796	0,864549852
OR51F1	0,458598677	0,064159647
ARF3	0,458859836	0,849917256
RPL3	0,458984978	0,210194311
LOC647786	0,459083218	0,06141761
C22orf25	0,459106934	0,162825966
CDKN2AIP	0,45927831	0,334977383
ZA20D1	0,459298412	0,141506128
LOC728511	0,459515676	0,21853516
MRPS9	0,459802186	0,098088188
COLQ	0,459879187	0,224554251
IWS1	0,459922615	0,123846076
TULP1	0,460136658	0,07272749

LOC643631	0,460264447	0,651952009
ERLIN2	0,460359087	0,38936063
MRPL43	0,460483528	0,293036557
LOC644093	0,46049071	0,310427962
ZFYVE21	0,460606711	0,066874447
ARL9	0,460627405	0,184257365
C8orf73	0,460663387	0,166427619
LOC727874	0,460762176	0,708827856
LOC729310	0,460928872	0,051844034
OR5C1	0,460954161	0,31428
C14orf166	0,461026398	0,139113293
HINT1	0,461097106	0,410256973
VASH2	0,461455909	0,51005015
ZNF37B	0,461472454	0,139560241
LOC402382	0,461560722	0,502824622
RNF5	0,461673909	0,913804987
SUHW1	0,461682281	0,208015732
LOC643136	0,461749274	0,905179325
PIGB	0,461894603	0,234863589
RAB21	0,462093305	0,249598761
NUBP2	0,462115972	0,357388446
C15orf60	0,462157218	0,109442745
ULK1	0,462160425	0,276470972
LOC440268	0,462174471	0,33432902
ASPG	0,462446476	0,056401805
LOC731312	0,462585095	0,526951902
GABRR1	0,462621281	0,09943428
LOC387787	0,462660095	0,900469502
SIGLEC10	0,462787698	0,233477694
SLC5A8	0,462901982	0,206648207
RBMS3	0,462952871	0,085017625
LOC644860	0,463197313	0,812633148
HNLF	0,463203106	0,558231513
PRKWNK1	0,46334828	0,060261482
IL12RB1	0,463857591	0,009044988
hCG_1984468	0,463915722	0,434561368
LRRC27	0,464015525	0,214706962
LOC728550	0,464027319	0,139365285
LOC339535	0,464240069	NA
DAZAP1	0,464396735	0,159204556
BRUNOL6	0,464712422	0,172310688
LOC731213	0,465040988	0,926242415
EXOC3	0,46504776	0,172451459
ACRV1	0,465273225	0,194320573
LYPD6	0,465809491	0,305594017

BLZF1	0,465892686	0,212774944
LOC732417	0,465907927	0,693504438
TPCN1	0,466059608	0,157735901
TAF1D	0,466502866	0,218630209
L3MBTL	0,466567628	0,081020746
CENPC1	0,466654822	0,420045513
GSTCD	0,466715424	0,093323897
SLC27A1	0,466816891	0,555777774
ABCF1	0,466829433	0,104562151
TEAD2	0,466936592	0,272151506
EMX2	0,467021542	0,124392709
TMEM104	0,467222019	0,485882912
SCFD2	0,467267037	0,175243181
CSN1S2A	0,467404167	0,256775761
KCNH6	0,468117728	0,417170702
FAM71F2	0,468168382	0,508405065
LOC728383	0,468170284	0,150049536
CALM3	0,468176631	0,70723706
MYH7B	0,468252745	0,410278094
STYK1	0,468501508	0,024799062
OR1I1	0,468550085	0,318088187
LOC643950	0,468663192	0,061539499
FLJ37512	0,468717191	0,079693905
LOC648852	0,46876372	0,664168725
IL9R	0,469056928	0,417266311
RGMB	0,469088089	0,146969299
LOC644978	0,469283891	0,160355686
TMC6	0,469284605	0,080570081
OSTbeta	0,469375978	0,258942592
SQRDL	0,469462254	0,065484842
DTX3	0,469664959	0,162049003
MAN2B1	0,469719651	0,98212852
LOC650181	0,469773537	0,211013544
ZNF394	0,469882485	0,297035558
BMPR1B	0,470156227	0,220218511
FZD9	0,470259066	0,582354593
RNF181	0,470328695	0,301649288
PRDM5	0,470381028	0,542179763
FGG	0,470432822	0,205372766
TNS4	0,47050714	0,501219769
PELP1	0,470671776	0,270919343
KLRC1	0,470867063	0,732043436
FABP3	0,470988161	0,670080234
HKR2	0,470989142	0,194166945
KIAA0226	0,47100536	0,300400466

GPR27	0,471023611	0,341284722
KRTAP12-3	0,471066037	0,012741526
FAM89A	0,471286155	0,113127684
KRTAP9-4	0,471371046	0,299721193
MLLT11	0,471376232	0,110996897
CPLX1	0,471451515	0,364736729
LOC654342	0,471676678	0,638506762
HRBL	0,471730325	0,223861927
LOC730067	0,471846847	0,759485844
COL5A3	0,472092236	0,445262452
CBR4	0,472456362	0,376251139
GHR	0,472470234	0,066567824
C14orf153	0,472551356	0,042348989
EPHB4	0,47294989	0,564119724
FKBP9	0,473101181	0,577342291
CPNE8	0,473146129	0,370084794
KCTD20	0,473234253	0,174429791
CEP63	0,47369863	0,069534239
LOC729829	0,473726995	0,191717714
ITGAV	0,473745325	0,136437119
TRIM23	0,47380345	0,332869071
IRAK3	0,474064693	0,181273646
LOC729712	0,474136069	0,390504503
WDSUB1	0,474354792	0,046151777
YIPF5	0,474411746	0,160400267
CAMK2G	0,474478398	0,659985813
TCFL5	0,474515105	0,516118736
SLC22A12	0,474570313	0,167913085
ADPRHL2	0,474672222	0,190698577
LOC645249	0,47505059	0,032893473
BUB1B	0,475138441	0,51681011
A2ML1	0,475376032	0,169291299
C1GALT2	0,475388852	0,153713172
MCMDC1	0,475844141	0,254197763
TUBG2	0,476038697	0,236156946
KIAA0174	0,476080377	0,155986042
PPP2R5B	0,476302097	0,387127036
PDGFD	0,476328866	0,132481534
ITGA11	0,476415257	0,168844122
RAB40AL	0,477116099	0,094802471
RNMT	0,477140642	0,315075817
OR9A2	0,477193104	0,162244003
COMMD4	0,477197886	0,111917874
NHEDC1	0,47736744	0,124181913
C19orf47	0,47749067	0,103433834

WRN	0,477493358	0,31713132
hCG_1645727	0,477545557	0,048664264
HECTD1	0,477803232	0,038686682
PPM1F	0,477809565	0,236124193
SLC39A3	0,478341546	0,350632184
SBF1	0,478533849	0,670840573
TMCO7	0,478621148	0,9324947
LOC645733	0,4787459	0,115305536
CTLA4	0,479017235	0,139185729
LOC391269	0,479172295	NA
ODZ4	0,479221149	0,153953443
DEFB4	0,479642702	0,129440527
C15orf27	0,47968654	0,338427551
LAMA2	0,479709203	0,076652167
C9orf131	0,480021054	0,169388658
FAM168A	0,480258052	0,270338612
CXCL12	0,480543882	0,56365465
LHPP	0,480954039	0,966525129
STRC	0,480977899	0,111503569
GPR40	0,481217914	0,336123171
HIST1H2BG	0,481288699	0,451828159
TBKBP1	0,4813171	0,407120671
LOC652733	0,481320258	0,111270597
AP1G1	0,481527106	0,232469313
LOC651186	0,481600527	0,011279121
hCG_1749005	0,482060716	0,651502008
OR3A2	0,482295294	0,438011755
KIAA1370	0,482316927	0,054352345
ALK	0,482471043	0,162358839
KLK8	0,482682394	0,639587677
GNPAT	0,482745329	0,058823135
SOX10	0,483163679	0,941098045
RYR2	0,483344567	0,207590093
LOC731135	0,483649047	0,126250833
CALB3	0,483695833	0,127338262
PSG6	0,484402605	0,141790976
CDYL	0,484524571	0,314041047
KRTHA5	0,484588821	0,462254411
AKAP10	0,484728359	0,578943597
HIST1H4E	0,484805948	0,134881512
LOC643428	0,484854437	0,109439403
AFTPH	0,484869545	0,020843001
BRI3BP	0,484937729	0,108442274
PDE5A	0,485032008	0,700031265
PCDHB8	0,485054648	0,278892673

GGA3	0,485076988	0,167497523
NCOA3	0,485123887	0,226792323
FLJ10213	0,485140651	0,793870251
NDUFS6	0,485162142	0,147494923
MYO3B	0,485255348	0,297547109
LOC729526	0,485262351	0,259516681
COL5A2	0,485292095	0,130486985
CNIH2	0,485326151	0,58684849
CBLN2	0,485922155	0,051781747
KIAA1303	0,486304649	0,350573622
CYorf15A	0,486338231	0,115568734
PCDHB13	0,486449822	0,273536686
TCEA2	0,486526347	0,51342903
PAGE5	0,486728406	0,299918305
RAF1	0,48691031	0,578068272
OSTM1	0,48720115	0,306171898
FLJ12895	0,487468458	0,125556759
COMMD5	0,487721283	0,812260471
C10orf125	0,487745054	0,053836503
CPB2	0,487965968	0,332719571
LOC728780	0,48839877	0,126617399
RHOBTB3	0,488400491	0,015676237
TNFRSF19	0,488558446	0,907896462
NFKBID	0,488622516	0,686227087
LOC440292	0,488679452	0,759034081
PCDHGA6	0,488911003	0,238222048
ARX	0,489001339	0,263961772
SIAT7F	0,489530209	0,205371366
NDST1	0,48978416	0,054040679
GRIK3	0,490035618	0,206924004
SMCR7	0,490115036	0,121540509
LOC148213	0,490394314	0,077205758
LOC649841	0,490526025	0,362135322
PRRG2	0,490890695	0,194575405
NME4	0,490934168	0,379932044
FAM55C	0,491439322	0,109415452
WSB1	0,49169949	0,453804666
HLA-A29,1	0,491780253	0,396411311
CNGA4	0,491785857	0,177975747
LOC731039	0,492148413	0,127321944
LOC196415	0,492412967	0,232922095
C1orf108	0,492554697	0,331422244
CLDN18	0,49274283	0,120063384
ALS2CR11	0,492762014	0,96593755
LOC646875	0,492792779	0,396343049

TPM4	0,493111353	0,01245015
LOC729713	0,493423874	0,026698275
ADRA1B	0,493577277	0,844708027
PLEKHM2	0,49358301	0,162830974
FNDC4	0,49381931	0,235897273
ITGB6	0,493879108	0,146071581
HFM1	0,494115278	0,085288344
EPHB2	0,494315291	0,165172371
PCDHA2	0,494734492	0,48082083
MYO15B	0,494831472	0,237201832
USP53	0,494989603	0,051449759
BCCIP	0,495258104	0,119965019
C20orf71	0,495272588	0,335344799
LOC729520	0,495353494	0,212882848
A4GNT	0,495905309	0,092842329
LOC390856	0,495979143	0,185737498
HTR5A	0,496039606	0,446347457
TNNI3	0,496104105	0,141648409
HMGB1L1	0,496437984	0,146485342
PROSC	0,496460978	0,077428971
WARS	0,496760767	0,918243519
TSPAN14	0,496877987	0,125208193
GLT8D4	0,497053554	0,123150077
C20orf160	0,497053652	0,422343003
OSR1	0,49731729	0,80679696
RAP1A	0,497579849	0,296281854
NPAS2	0,497904745	0,327497943
EDG5	0,497944521	0,066558461
KIAA1644	0,498119035	0,509186975
DKFZp686L1818	0,498216414	0,278409705
DEFB121	0,498264152	0,138720722
MTIF2	0,498306787	0,355383474
SLC9A3R2	0,498342014	0,936419366
OCM	0,498608052	0,881586568
GRTP1	0,498749576	0,067253797
NICN1	0,498980486	0,372603635
LOC728594	0,499388997	0,012214016
P4HA2	0,499468056	0,23506241
C18orf54	0,499623588	0,178969751
LOC643332	0,499746984	0,29577495
MGC4399	0,499791402	0,075621197
CDH19	0,500194208	0,024116653
LOC81569	0,500241354	0,641603252
LOC729979	0,500434027	0,283276548
PARVA	0,500437629	0,141784191

P2RX5	0,50054282	0,602606907
PDE2A	0,500650011	0,766442709
MYC	0,500741581	0,189687485
ASB11	0,500862739	0,83572788
TIEG2	0,501110844	0,245437412
LOC730241	0,501271551	0,030992459
LY64	0,501468293	0,25658763
CLTB	0,501834015	0,227088548
PAFAH1B3	0,502116694	0,078844689
IL17RB	0,502155293	0,133586049
ENPP4	0,502177333	0,080825743
SIRT4	0,502191356	0,089758038
C19orf10	0,502246814	0,019916813
ZNF278	0,502308113	0,768283229
LOC647315	0,502493218	0,947618956
B3GNT4	0,50275034	0,301785348
EVC	0,502799804	0,277356966
GALNT11	0,503081989	0,690834553
SPEF1	0,503190742	0,101842702
PPP3CC	0,5033138	0,148109463
TH1L	0,503421045	0,061589024
TM2D1	0,503444025	0,06028721
BRE	0,503597252	0,122481899
LOC388915	0,503910718	0,344245852
RAP1GDS1	0,504106177	0,060461277
AFAP1L2	0,504284034	0,135501754
SYBL1	0,504293515	0,791213502
HIST1H3A	0,504389274	0,133803393
MCP	0,50449042	0,190821583
SHREW1	0,504813351	0,209416303
GIMAP4	0,504918022	0,416282383
KIAA1377	0,505119494	0,072357592
CCDC12	0,505401482	0,12889721
SYS1	0,505401693	0,412719255
ZNF498	0,505431061	0,203977027
RASSF1	0,505457601	0,369128591
STCH	0,50587992	0,55207084
LOC729015	0,505908641	0,23472705
TMEM102	0,505954812	0,247240137
LOC729643	0,506219621	0,318187741
NCAM2	0,506350178	0,207801545
FBXO41	0,50647166	0,095006764
TMEM203	0,506525146	0,346174065
CHST7	0,506592494	0,328252583
KRTHA3B	0,506854971	0,948769215

PPAP2B	0,506878883	0,366574071
LAS1L	0,506928429	0,056537346
JMJD2B	0,50701861	0,082034936
PLAGL1	0,507238567	0,643982913
SULT1B1	0,507490257	0,148234648
TMEM106C	0,507808127	0,142761941
C6orf191	0,507851605	0,805637789
CCDC106	0,50813907	0,609969441
PATE	0,508232997	0,154997679
LOC389842	0,508272921	0,108188096
SF3A3	0,508359948	0,62168652
ODZ3	0,50842127	0,926214641
EXOSC3	0,508813457	0,1177514
LOC27320	0,509043106	0,156503272
LOC730243	0,509204334	0,123014434
TNIP1	0,509413272	0,125816849
LOC338756	0,509672288	0,314545084
IL21R	0,509919394	0,239366534
ZNF197	0,510229337	0,205497962
MAGIX	0,510359114	0,036381233
LOC729710	0,510395069	0,264283582
KIAA0352	0,510556054	0,698327537
ZNFN1A4	0,510621655	0,554348563
OR8B4	0,510752958	0,199311425
SLCO6A1	0,51075385	0,28823196
LOXHD1	0,511362503	0,050316671
LOC645655	0,511716266	0,075607523
CHURC1	0,51190868	0,542924455
MRE11A	0,512200686	0,70075261
BIRC7	0,512223646	0,922877547
FOXD1	0,512265444	0,538655486
CNTNAP3	0,512469776	0,940711376
SIAT7C	0,512729322	0,407998161
CXCL11	0,512740136	0,183256417
FUK	0,512840455	0,202330458
DEFB123	0,512944241	0,129729227
LAX1	0,513050472	0,107466321
LONRF3	0,513264124	0,160556062
MYO1B	0,513348867	0,468247912
CHRNA2	0,513364808	0,199343918
FEN1	0,513633182	0,191350663
SUOX	0,513859954	0,12332161
EDIL3	0,514033422	0,023894175
ZNT8	0,514233308	0,332845586
hCG_1795065	0,514242163	0,279413745

P4HA3	0,514546205	0,334784971
BNIP3L	0,514678408	0,087070592
C1orf94	0,514942303	0,828954359
PHPT1	0,51525318	0,422721098
PRKCQ	0,515300368	0,210015311
HSP90BB	0,515316028	0,36912676
CSAD	0,515599505	0,468916505
GTF2H1	0,515813615	0,296201334
MYL9	0,515959229	0,036695472
CLEC4M	0,516172829	0,512807012
LOC727929	0,516242539	0,450305496
ENSA	0,516649494	0,134037299
LOC729059	0,516679289	0,215703549
LOC646799	0,516774992	0,83912158
GPR8	0,516934805	0,634509118
TRIM37	0,516980601	0,365842848
PIBF1	0,517155165	0,077810328
KIAA0409	0,517260898	0,018183532
WDR21B	0,517817513	0,549084577
MGAM	0,517830771	0,07146528
PLA2G2A	0,518034393	0,634963795
NCOA6	0,518149801	0,073873662
ZFP276	0,518261967	0,197208068
FLJ39080	0,518402735	0,106635753
SLC1A7	0,518452131	0,109919741
DDX52	0,518558148	0,208862803
SEMA6D	0,518679404	0,220209151
CDKL4	0,518879222	0,460061384
ZNF815	0,518908115	0,239214702
SLC10A5	0,518965668	0,1693885
TRPV2	0,519339391	0,090874203
RPL34	0,519607579	0,599116248
hCG_1783907	0,519826171	0,531335936
OR8G5	0,51991372	0,012860341
ENPP2	0,520030836	0,116115338
ADCY5	0,520141863	0,876445437
DNAJC5G	0,52023916	0,166626424
ZNF248	0,520326451	0,542941988
USP20	0,52033771	0,19079022
GIMAP8	0,520379332	0,162052229
ERCC1	0,520489445	0,105101774
KIAA0980	0,520556166	0,120627782
EFHC2	0,520884464	0,844722927
hCG_1780278	0,521202156	0,047525102
COX4I2	0,521234808	0,136191044

C1orf149	0,521498481	0,087757724
BACE2	0,521595708	0,156878527
SERPINA2	0,521655057	0,342759395
CITED2	0,521725966	0,140703468
CERCAM	0,521770177	0,369270531
TMEM98	0,522022659	0,867709372
STAB2	0,522301262	0,050832519
SLC2A9	0,522474224	0,08991882
FDFT1	0,522965657	0,684315689
MAPK1	0,522996252	0,242822015
C11orf9	0,523014985	0,178728729
LOC729395	0,523061792	0,293675641
RASSF2	0,523171083	0,232767354
CAMK2A	0,523190389	0,199636758
FAM152B	0,52322467	0,043949882
INHBA	0,523411376	0,190341583
DCUN1D3	0,523586986	0,13398683
PDXDC2	0,523926741	0,613532811
MTRR	0,52426584	0,348338888
FAAH2	0,524417214	0,130730482
HIST1H1T	0,52460377	0,172273292
IQCF2	0,524607916	0,37598649
GREB1	0,524804114	0,242274
GPR81	0,524938932	0,291762101
NTN4	0,524947127	0,253756108
LOC729275	0,524983253	0,101662201
PTPRE	0,525078502	0,236306432
LOC731102	0,525120734	0,039384709
POLR2G	0,525310392	0,443837931
PIP5KL1	0,525676373	0,797183
GLB1L2	0,526078742	0,036874023
FREM1	0,526108287	0,090106612
ZNF830	0,526109415	0,095865785
PLAC2	0,526314561	0,643979029
GYPC	0,526485661	0,228544296
OR51B5	0,526644656	0,061398633
CCNH	0,526999031	0,521180003
OR4K17	0,527095764	0,150323551
LOC645027	0,52712275	0,207913685
CHST10	0,527131041	0,130699372
MGC3162	0,527163106	0,035744577
MRPS14	0,527600792	0,087243155
GPR112	0,52768819	0,486052302
MGC15631	0,52778286	0,999218922
ZFYVE19	0,528089658	0,453715263

SEMA4G	0,528140812	0,862045907
CNOT4	0,528337735	0,276975476
USP28	0,528417272	0,22007069
CD164L2	0,528654368	0,275528125
LOC730141	0,528760133	0,113198683
EP300	0,528994677	0,077487851
SPANXB2	0,52905957	0,121485745
RP13-347D8,3	0,529214597	0,218194183
LOC729746	0,529379098	0,070350389
ANKAR	0,529437138	0,969042547
LOC728442	0,5295564	0,041767695
OR1B1	0,529704765	0,211241795
LOC283867	0,529842036	0,073749815
RPS21	0,530106341	0,727377054
LOC440490	0,530280521	0,190388349
GATA4	0,530344513	0,250125656
ZNF335	0,530427398	0,76418305
FOXD4b	0,530832388	0,129334268
KCNC2	0,530904562	0,186590383
LOC730057	0,531043643	0,027109614
C11orf74	0,531135616	0,026212912
SLC22A11	0,531252197	0,22929149
SYT12	0,531285244	0,20080894
HNRPCL1	0,531550164	0,166018208
PITPNA	0,531854779	0,442400847
P2RY11	0,532012975	0,211318329
RGS8	0,532313741	0,346729331
OR6B1	0,532377135	0,107948416
DGCR14	0,532383125	0,120289621
DKFZP434B0335	0,532440399	0,681100051
GFRA1	0,5324752	0,437533967
ADNP	0,532540624	0,03314454
ZNF266	0,532625602	0,209994476
TMEM167B	0,532780798	0,344287454
LOC643330	0,532918152	0,382548326
ANO8	0,532976138	0,079013861
LOC646012	0,532978287	0,083555025
LOC442175	0,533019222	0,021711804
TMEM109	0,533094936	0,178622909
FEZ2	0,533155348	0,251504621
GALP	0,533159856	0,192856329
HBS1L	0,533177028	0,247939035
CLIC5	0,53383788	0,291354737
LRRC49	0,533865619	0,629021145
USP40	0,533912375	0,504179894

LOC643751	0,534021854	0,08915505
SLC18A2	0,534043223	0,319720792
PAPOLA	0,53430171	0,817705945
GJB1	0,534317206	0,058086369
LOC648830	0,534353421	0,058349532
LOC728074	0,53445272	0,56137346
LCE4A	0,53458956	0,514323325
CEACAM8	0,534866392	0,190384526
IGDCC3	0,535234752	0,313519426
ESCO2	0,53535478	0,25082919
SIAH1	0,535804937	0,558026625
LOC731425	0,536086453	0,098795135
C1QC	0,536195348	0,242044768
CUZD1	0,536239537	0,020839089
OR1L3	0,536317868	0,245903791
FLJ40298	0,536332553	0,678179538
RAB11FIP5	0,536424033	0,108261099
C10orf11	0,536681261	0,037485644
MAT2B	0,53684422	0,111308179
RNF122	0,5370576	0,906467409
FLJ38973	0,537269595	0,094394172
EFNB2	0,537325347	0,071613772
C1orf104	0,53770753	0,241634963
LOC731816	0,538197406	0,197690055
CCDC58	0,538543604	0,167995428
LOC642014	0,53863328	0,483178474
IMP-1	0,538671204	0,117433665
GSK3A	0,538731178	0,285284509
HLA-DOA	0,538739969	0,13084347
ZNF140	0,53879452	0,966916504
AIP1	0,538900157	0,122935127
LOC51057	0,539117899	0,087765401
LOC647988	0,539210823	0,175458807
SGTA	0,539211471	0,812665934
DLG1	0,539279159	0,214424009
CA10	0,539420708	0,1049497
RNF17	0,539533132	0,057665242
LOC642160	0,539920933	0,222405363
LOC643997	0,540028645	0,11800601
KIAA1967	0,540042118	0,136171033
DKFZP564O0823	0,540310703	0,091445384
SURF2	0,540350832	0,149853414
LOC731587	0,540381806	0,350223546
SPANXN3	0,540417862	0,975629269
C15orf33	0,540576145	0,003458758

SPSB4	0,540841307	0,111690119
POLR2C	0,54100992	0,375169777
DLGAP1	0,541018048	0,256071464
KRT72	0,541259833	0,152842439
RBM23	0,541369875	0,187515979
DHX16	0,541389633	0,360857271
SLA	0,541595902	0,14508231
LOC728357	0,541644231	0,097757653
RBM19	0,541925486	0,105045613
PDXP	0,542251125	0,053446324
ARHGEF5	0,542259827	0,389795524
FBXO28	0,54227644	0,498429251
HHLA3	0,542513801	0,5258398
KIS	0,542907651	0,010893029
CCNT1	0,542957448	0,039286346
GPR35	0,542984432	0,078162544
PVALB	0,543005479	0,503919383
FBXO7	0,543125568	0,221242316
GRIN3B	0,543142085	0,588145295
RGS11	0,543907315	0,078084998
MYADM	0,54403591	0,019529369
AMIGO2	0,544338731	0,052399581
RECQL5	0,544391336	0,032671828
LOC647102	0,544497715	0,434999885
NTNG1	0,544805431	0,222330902
LOC649198	0,544840132	0,120649629
EPHA3	0,54491923	0,11895839
LOC727886	0,545211735	0,107639808
STMN2	0,545363998	0,009821774
LOC390007	0,545472598	0,191598976
C14orf174	0,545500601	0,21427604
GAD1	0,545706121	0,469902748
FLJ35848	0,545789967	0,090552851
RIMS3	0,545970029	0,052735327
LOC728573	0,54622303	0,119155382
PHYH	0,546377574	0,486805867
MMP20	0,54647369	0,867342633
LOC730245	0,546479184	0,113203843
SNAI2	0,546605674	0,136670287
SLC25A13	0,546781816	0,496043137
BFSP2	0,546951171	0,077213341
CCR8	0,547050479	0,569656588
LOC647946	0,547063322	0,478852319
FAM47A	0,547124308	0,152140395
DGAT2L3	0,547539274	0,066244708

C8orf46	0,54754301	0,058191836
ATXN2	0,548177619	0,18280085
BNIPL	0,548574969	0,010722389
LOC730495	0,548584071	0,274180388
ZNF821	0,548807665	0,129148008
LOC646871	0,548874297	0,036780747
AAAS	0,549399431	0,076830197
CTNNA1	0,549476015	0,047611991
ARL2	0,549526408	0,172902894
PDPR	0,549698058	0,770807179
LOC647204	0,549760151	0,006740687
F3	0,549849993	0,742756731
SYCN	0,549911734	0,578490565
BRWD2	0,549971149	0,107305744
LOC729902	0,550000307	0,05531525
C8orf47	0,55019675	0,354198872
LOC728686	0,550255756	0,206453801
CCDC67	0,550272346	0,75605331
EED	0,550530562	0,021084652
LOC646993	0,550735642	0,138251526
WIT1	0,550761803	0,097606072
PTPN9	0,5508755	0,037343904
GEMIN5	0,55169954	0,897612238
ADAMTS18	0,551764863	0,013831403
FLJ20464	0,551788692	0,556686851
SRD5A2L2	0,551823355	0,618820064
C10orf48	0,551824764	0,157565256
DMC1	0,553236242	0,466231656
GRK6	0,553462846	0,30367483
LOC652783	0,553520815	0,038285815
LOC641808	0,553695533	0,115079817
LOC642892	0,553798162	0,9409075
C14orf169	0,5538401	0,214116271
INSM1	0,553942823	0,202309556
C6orf184	0,554285262	0,186047778
IQCF1	0,554288593	0,090460324
CTAGE1	0,554288661	0,126653561
KIAA1510	0,554297159	0,406332476
RPS27A	0,554403336	0,146709227
ARID5A	0,554431801	0,174615262
LOC199897	0,5545808	0,32350007
KCTD12	0,554870653	0,193563384
MYH6	0,554878186	0,113480266
PRKWNK2	0,554996766	0,182145503
LDHA	0,555053204	0,183506274

TRPC5	0,555110135	0,628591211
ACTN3	0,555151738	0,845465857
LOC644368	0,555181467	0,051674611
BMP10	0,555323494	0,202898956
LOC645930	0,55550833	0,060983755
SLC22A4	0,555529518	0,100553173
AGRN	0,555969984	0,015400085
PELI1	0,55645205	0,29240853
SLC30A6	0,556534937	0,219770973
ALDH3B1	0,556629887	0,725284919
CD44	0,556663302	0,078810586
PSMC6	0,556666397	0,184498001
KCNT1	0,556698157	0,251175946
PRR4	0,556773802	0,121947085
GAS2L2	0,557011783	0,206395846
COPS7A	0,557319376	0,059806827
TEX13B	0,557418843	0,039658887
RGS21	0,557566944	0,355896709
ACYP2	0,557638227	0,212891353
RPS4Y1	0,557834735	0,026448205
LOC644023	0,558188517	0,254009268
TEB4	0,558389729	0,147211132
RIN3	0,558451725	0,095980205
CLRN2	0,558455716	0,087881382
RBBP4	0,558473647	0,153178569
C9orf79	0,558585915	0,801549016
UNC84A	0,558590633	0,309368574
LTF	0,55890443	0,20075243
SENP7	0,559484119	0,086645458
LOC732286	0,559582772	0,374643396
GTF2A2	0,559671549	0,786734613
FLJ30403	0,559783933	0,195808515
RNASE12	0,559794057	0,896596267
DEFB116	0,55997917	0,082862889
KLP1	0,559998721	0,183790101
ARHGEF2	0,560309349	0,679571325
GBE1	0,560450337	0,175367619
HAS3	0,560566319	0,043818234
M160	0,560748724	0,17694682
LOC653808	0,560787672	0,036418257
TMEM20	0,560952243	0,891857308
CXADR	0,561552405	0,070101365
FAM71E1	0,561605027	0,073009049
FAM22E	0,561639043	0,065845207
LMX1B	0,561989981	0,193525915

LOC646324	0,562144852	0,228526262
C19orf62	0,562162172	0,441303945
TMED3	0,562383652	0,057594021
C1QL4	0,562632902	0,011417317
DDEF2	0,563177852	0,676489454
SPANXA1	0,563189697	0,342361441
OFCC1	0,563233223	0,164808234
ZNF8	0,563287645	0,358123609
COMP	0,563316137	0,049649096
LYG1	0,563776846	0,032340788
TMEM196	0,563992155	0,226069807
LOC732114	0,563993564	0,118849443
DCLRE1A	0,56408508	0,407224864
SYT11	0,564101863	0,02196774
SLC3A2	0,564456142	0,248230722
ICK	0,564488779	0,220907971
MAS1	0,56457568	0,041906759
ZNF71	0,564593443	0,150434282
MAPBPIP	0,564656689	0,267945637
CD276	0,564745051	0,104788699
OR5H6	0,564760552	0,258493001
SLC22A23	0,56481256	0,058893811
MRPL51	0,565045416	0,26010026
CTAG2	0,565075583	0,219874873
CD3Z	0,565202843	0,070388345
C4orf14	0,565231397	0,04772438
LOC728836	0,565239433	0,191402765
HOXD11	0,565526585	0,157304838
HFE2	0,565599529	0,141589349
CLCA1	0,565615952	0,155031279
TEX101	0,566147889	0,139044668
SF3B4	0,566232117	0,52351282
SLC25A14	0,566277158	0,275804767
LOC731517	0,566308602	0,036187704
SLC22A1LS	0,566417976	0,345119415
RAI14	0,566762249	0,212609642
MGC33414	0,566865657	0,193063936
ZFC3H1	0,566899605	0,084415231
CENPO	0,566935168	0,633140201
RAG2	0,567377694	0,405989194
C17orf76	0,567696816	0,253795879
RNF138	0,567701905	0,242999221
SCRT2	0,56783176	0,205639773
SERPINC1	0,56802375	0,060003439
SKIV2L	0,568669238	0,047419095

TRIM66	0,568774293	0,331113148
QSER1	0,568870149	0,043563454
RBED1	0,569080065	0,23855907
LGALS3BP	0,569258415	0,239062015
LOC729993	0,569330739	0,272205878
DKFZP547L112	0,569331585	0,107939047
C21orf7	0,56936497	0,253105424
FLJ45803	0,569987525	0,175654993
MRPL20	0,56999578	0,162937954
OR7A10	0,570159504	0,16772553
LOC732475	0,57037089	0,418332562
ART5	0,570727595	0,682920024
ZNF451	0,570777348	0,782837524
PARP16	0,570878916	0,131764014
LOC645417	0,570911998	0,569458237
LOC391742	0,570914717	0,056439968
ANXA9	0,570969995	0,441886079
BEX2	0,571003303	0,111067231
M6PRBP1	0,571229965	0,170856979
CENTB2	0,57141728	0,063862167
TP53I11	0,571490596	0,139305118
USP18	0,571544938	0,261112659
SRD5A2	0,571723077	0,999354174
PTE1	0,571775362	0,005741574
LOC647169	0,572041124	0,109752534
ASNA1	0,57232804	0,164575874
RGS14	0,572451481	0,154662083
ASMT	0,573014624	0,135215615
LOC645018	0,573405201	0,831553461
KIAA1841	0,57368849	0,05451113
TAS2R10	0,573928324	0,296045504
ZNF331	0,573984632	0,195236205
ZNF616	0,574344566	0,672657037
OR5AC2	0,574534139	0,142173261
KCNK10	0,574657857	0,460487166
KCNJ6	0,574681329	0,757273436
LOC651667	0,574715972	0,127766811
LOC647309	0,57476811	0,393298194
SUMF2	0,574777406	0,170784804
NUDT1	0,574829858	0,180721526
C9orf93	0,574973027	0,103577073
TEAD1	0,57513916	0,26257512
C14orf127	0,576010371	0,121593215
LOC729528	0,576078764	0,097058978
ZNF608	0,576114784	0,218888136

ISCA2	0,576310012	0,078091551
LOC729860	0,576388675	0,598921288
RHPN1	0,576846005	0,368717327
MGC50722	0,576961188	0,634165429
C17orf28	0,577102724	0,13778337
MLLT2	0,577241058	0,12767073
FOXM1	0,577382569	0,133022608
HDGFRP3	0,57738366	0,245517211
KCNJ8	0,577600018	0,500206996
PCDH9	0,57772914	0,131824441
CHRNA4	0,577808594	0,251102104
CCDC22	0,577883348	0,336368458
TMED6	0,577974101	0,257853498
RNPC3	0,578012326	0,392504925
ATP9A	0,578030941	0,181058182
DAB1	0,57806924	0,255428394
C19orf61	0,578105715	0,05048391
UMPS	0,578209261	0,185647071
AKR7A2	0,578310225	0,162848193
NFE2L2	0,578455206	0,795256797
C4orf37	0,578802035	0,096008092
DKFZp564K142	0,578930679	0,108171328
GPR84	0,579044782	0,044942595
LOC728172	0,579234931	0,078164921
NCF1	0,579320667	0,241265477
hCG_1657112	0,579369419	0,093545887
LOC285501	0,579510951	0,047258833
LOC642936	0,579516936	0,101137455
CACNA1F	0,579521862	0,104157496
TOE1	0,580014789	0,35126468
SLC4A11	0,580423824	0,749711092
OR6M1	0,580659579	0,145217214
LOC645874	0,580664216	0,009311065
MXRA7	0,58102279	0,795670132
KLK13	0,581700223	0,215409728
IL17RC	0,581939746	0,004448552
LOC440748	0,582109772	0,036126816
JAM3	0,582145973	0,147645932
CRYZ	0,582170001	0,098600261
TMEM16B	0,582336284	0,043246829
VSX1	0,582469122	0,680491762
CASP10	0,582513133	0,24225978
RALGDS	0,582893918	0,889285063
LOC645489	0,583104256	0,233343271
ZNF26	0,583686747	0,260607717

LOC376475	0,583734773	0,302883247
SRGAP2	0,584223049	0,132973844
TTC30A	0,584323999	0,654980613
LOC728453	0,584386241	0,741093314
TUBB2	0,584998845	0,097723767
ZC3H12C	0,585104201	0,761608891
ASXL2	0,58512269	0,05124566
SIGLEC8	0,585815471	0,393552202
LGALS14	0,586057285	0,49606346
HIF1A	0,586240887	0,004275726
LOC442221	0,586388687	0,356455311
LOC731901	0,586395064	0,08447988
OR9K2	0,586465628	0,085983334
ZNF137	0,58681601	0,164265365
LOC646561	0,58686704	0,07242565
ATP8B3	0,58710841	0,251352092
LOC644935	0,587441657	0,111030688
LOC751071	0,587639414	0,046133089
SP140L	0,587669572	0,265917475
RDH12	0,588269849	0,028107652
HLA-DPB1	0,588439726	0,357890089
FOXD2	0,588768044	0,015759965
ADAMTS7	0,588779312	0,087690112
SOS2	0,588791065	0,122847872
NDUFB1	0,589059507	0,799953699
SOX8	0,589110993	0,35429979
TPST1	0,589197616	0,229450794
LOC728995	0,58935527	0,417304191
EPC2	0,589601369	0,231093778
ITM2C	0,589648737	0,244335196
ABCA3	0,590282889	0,232121788
LOC646627	0,590315747	0,040202039
KRT24	0,59033435	0,187331467
BMP1	0,590421773	0,146996838
C2orf48	0,590449024	0,091731249
LOC402217	0,590510978	0,499349693
SAMD3	0,590712624	0,623835686
LRP1	0,590854287	0,782462838
LOC730148	0,590878602	0,316420862
LSM14B	0,591267034	0,060824323
KIAA1600	0,591284041	0,243603884
LOC730453	0,591529044	0,047842299
CGREF1	0,591539541	0,187431286
CCL26	0,591565816	0,236297921
RPS13	0,591574766	0,086041712

RCBTB1	0,59162865	0,055274722
OR5A1	0,591657354	0,215129487
KIAA0746	0,591705696	0,108342198
NDUFA11	0,591821411	0,163071126
C1orf57	0,591836573	0,039187266
LOC645166	0,59185922	0,644335459
LOC388720	0,59228568	0,226131734
RGAG4	0,592623685	0,205117965
CD99	0,59262944	0,124485582
LOC728289	0,593954395	0,132689204
PARP8	0,594126656	0,38698154
MARK4	0,594518665	0,184985382
SLC29A1	0,594655277	0,633383659
CALN1	0,594780986	0,177402291
PNKP	0,594788444	0,33902715
CUEDC1	0,595471063	0,339859652
KRTAP3-2	0,595733829	0,154108775
C13orf18	0,595760407	0,180409486
PPID	0,595949328	0,504492561
CFDP1	0,596206876	0,034220631
ANTXRL	0,596225846	0,230297617
C3orf30	0,596285211	0,284391229
ACHE	0,596428306	0,698245503
RNF190	0,596479592	0,016762153
C7orf25	0,596970351	0,515420571
DTWD1	0,59701263	0,32903803
KCTD16	0,597060591	0,011758559
OR13G1	0,597345668	0,095042035
MRAS	0,597408105	0,370663068
UBE2B	0,597427047	0,252036099
CTSZ	0,597736022	0,222609239
WFDC1	0,597870326	0,339738551
ELMO2	0,597982165	0,144160182
CCDC17	0,598379012	0,06949035
LOC650557	0,598713424	0,064019866
DACT3	0,598782117	0,02308732
ART1	0,598868451	0,821750755
OSM	0,598875115	0,128921437
NFIC	0,598882272	0,136609071
GNB5	0,599100767	0,241083239
APOBEC3B	0,59935337	0,268913593
LOC649425	0,599371598	0,151540132
SFRS15	0,599457568	0,012233785
C3orf19	0,599791293	0,155591175
C1orf67	0,599874263	0,077452435

LOC646254	0,59992762	0,002189946
GSTM1	0,600085615	0,094345935
LOC728104	0,600327223	0,06999995
NDUFA2	0,600374307	0,07922929
RETNLB	0,600396241	0,36438183
TIGD5	0,600581398	0,086723281
SH3RF1	0,600599032	0,01501882
hCG_1644608	0,60093923	0,116656901
ETL	0,601042795	0,271674774
LOC730238	0,601125188	0,06065915
FBXW7	0,601207023	0,123978252
C15orf41	0,60125598	0,06772607
KRTAP12-1	0,60166408	0,213645099
PDZGEF1	0,601836344	0,332102306
TMEM18	0,60192042	0,114758088
HLA-DOB	0,602069943	0,711404953
VPS4A	0,602203309	0,088777156
SERINC5	0,602222601	0,052778711
FLJ33655	0,602295728	0,181807911
PTPNS1L3	0,602657099	0,79541741
C17orf80	0,602893102	0,321802467
LOC730631	0,602922449	0,013057511
OR5K1	0,603190855	0,394372608
FRMD1	0,603286056	0,196319403
LOC653653	0,603334031	0,470840145
FAM55A	0,603373828	0,247533072
CRSP7	0,60363534	0,31649549
TRIM5	0,603753506	0,899494666
IDH3A	0,603813054	0,266126843
CLCN2	0,603819335	0,704494021
FAM50A	0,60390451	0,017568157
KIAA1683	0,603951399	0,509684586
JAG1	0,604197762	0,213937201
RIC8A	0,604240364	0,170438494
SNAPAP	0,6042983	0,013857532
LOC652904	0,604316636	0,020600429
LOC728052	0,60436982	0,164138324
LOC728828	0,604496112	0,21819677
LOC728108	0,604496537	0,169635728
TNNI2	0,604643603	0,577122877
SPARCL1	0,605099506	0,436094429
LOC643517	0,60522592	0,246451501
CRYBA2	0,605259228	0,288616037
ZUFSP	0,605317051	0,269315333
VGF	0,605579056	0,17665174

KIF22	0,605793825	0,174364841
USH1G	0,606281975	0,098319054
C7orf27	0,606402737	0,132384052
BCAR1	0,606444588	0,1743304
ATF7IP2	0,606612271	0,0237617
C6orf47	0,606731574	0,052369983
LOC130355	0,606837693	0,236556876
LOC643594	0,606890542	0,855217315
RASSF7	0,607022567	0,330746629
TMEM161B	0,607128126	0,05079166
LOC646804	0,607363309	0,28472274
ZNF23	0,607667236	0,643364608
GSG1	0,607679687	0,216602198
ERCC4	0,607683597	0,305623408
CSGlcA-T	0,607806952	0,268999752
TMC3	0,607848198	0,411239404
IL1A	0,607881423	0,181432101
FLJ45032	0,608132631	0,029163307
NKPD1	0,608229225	0,48588941
MGC24975	0,608464523	0,041817097
SLC9A9	0,608478074	0,836377827
MSH4	0,608574609	0,25437676
PLAGL2	0,608956491	0,096654057
11, Sep	0,609031435	0,105694848
C8A	0,609154562	0,19144282
HIST1H4H	0,609380674	0,170560742
FAM124B	0,609440533	0,043789473
KIAA1613	0,609756066	0,2418841
LOC642440	0,609784604	0,949944281
HAS2	0,609950722	0,118226558
C1orf129	0,610139917	0,030500374
GPR74	0,610226857	0,152973888
TAB3	0,610283087	0,298466967
GCH1	0,610357208	0,797299387
CTF1	0,610568167	0,637086456
BTRC	0,610901357	0,112112557
LOC90806	0,610917745	0,50895868
TNFSF11	0,611150416	0,024829341
GRIA2	0,611273206	0,135597813
STAT5A	0,6113438	0,112362069
ARAP3	0,611598594	0,013958842
LRP6	0,611682616	0,123910054
FLJ14213	0,611877644	0,230105054
PDE4B	0,611885594	0,241123077
LIMK2	0,612002758	0,379810515

KLC2	0,612157299	0,314012165
OVOS2	0,612232913	0,373925298
XYLT2	0,612593067	0,04761957
VPS24	0,612630052	0,162855824
N4BP2L1	0,613043551	0,049571799
LOC342293	0,613362983	0,187857777
NFKBIZ	0,613439238	0,008439779
CST11	0,613501807	0,071322966
GAB2	0,61375619	0,311072341
LOC729957	0,613758847	0,130522493
MKI67IP	0,613808054	0,077018588
HOXA9	0,613831729	0,17561424
KIAA1328	0,613961592	0,71945684
DNAJC21	0,61397038	0,09371309
KIAA0999	0,614145664	0,164200769
SPR	0,61444188	0,155803536
OSTalpha	0,614608706	0,725381223
P2RY8	0,614693979	0,393846276
LOC647436	0,614965712	NA
C9orf153	0,61515372	0,354590594
SLC7A6	0,615229676	0,296101231
C1orf51	0,615314617	0,190374097
LOC440157	0,615350946	0,014449267
GOT2	0,61545814	0,269167327
CPSF1	0,615683612	0,203091919
REEP4	0,615742271	0,282833833
PACSIN2	0,615945374	0,399098283
C11orf30	0,616287322	0,183237572
TRA2A	0,616371803	0,165154918
PPP1R3D	0,616392194	0,596964552
AREG	0,616512735	0,05180548
MTMR4	0,616581643	0,681026558
FBXO3	0,616905822	0,701400306
PCP4L1	0,617049888	0,088293192
NUDT4	0,617232425	0,033418783
C6orf15	0,617257951	0,218279255
AXIN2	0,617268657	0,087001411
LOC339967	0,61736715	0,057867135
MAGEB6B	0,617507847	0,078730882
PTPRU	0,617880183	0,035142574
ETV4	0,618063849	0,142005513
KCNAB3	0,618420201	0,446196679
NBL1	0,618620583	0,109570984
TRIM64	0,619286275	0,157498582
GHRL	0,619331378	0,102498814

MRGX4	0,619387873	0,197481389
CDC14A	0,619715524	0,153263403
ZNF46	0,62005705	0,511995978
C9orf6	0,620174133	0,103796895
LOC645915	0,620249132	0,033130597
CA4	0,620485743	0,197539219
TSKS	0,620599811	0,08981125
LOC729831	0,62068566	0,139661396
NBPF20	0,620745635	0,344454974
C9orf150	0,620793056	0,069506036
UPP1	0,620893177	0,1036631
LOC442049	0,620971998	0,086296518
PCSK7	0,621354008	0,090373915
DIRAS1	0,621472742	0,032057274
FAM176B	0,621478214	0,12107136
SPHK2	0,621686875	0,346722076
WFDC10B	0,621745189	0,242269789
INSL3	0,621926413	0,154300155
LCN9	0,621948123	0,376973521
PCDHGB4	0,622137053	0,14509364
CDC6	0,622260422	0,054397444
C1orf222	0,622484519	0,248156739
LOC339344	0,622540473	0,165022679
TAS2R41	0,622698263	0,565632846
TXNDC15	0,622843168	0,094140298
UBE2S	0,623594457	0,957752446
IRF2BP1	0,623660245	0,175476781
COL3A1	0,623668127	0,109194216
hCG_2045437	0,623839068	0,104647104
LOC340602	0,623875834	0,042925363
LOC150577	0,623942226	0,469205125
DDC	0,624009845	0,086304091
ARSB	0,62415799	0,255920462
ID3	0,624675027	0,3112855
ZNF326	0,624910552	0,256293459
LOC729286	0,624931331	0,157238951
NECAP1	0,625020008	0,115657913
HIST1H1A	0,625037481	0,188996824
CCL5	0,625094396	0,149067028
PHC2	0,625227781	0,143345687
PI16	0,625560241	0,148025097
LOC728067	0,625564526	0,036099502
TAS2R48	0,625624962	0,269581838
GRB10	0,626040781	0,436272756
ENTPD8	0,626054599	0,208150645

LOC646543	0,626431036	0,094967049
FAM114A1	0,626519799	0,084850834
FAM65C	0,626594324	0,305193892
FLJ16369	0,626902684	0,686413233
RPL8	0,627140728	0,304115178
CDH20	0,627509784	0,28985267
C9orf21	0,627719718	0,117182988
TMEM146	0,627875929	0,35124481
LOC84643	0,628104328	0,21202866
ITLN2	0,628430789	0,060909386
RFC4	0,628431033	0,943876173
HIST2H3D	0,628998834	0,011519544
IRF8	0,629009788	0,304600596
C11orf68	0,629011288	0,196261179
TCF1	0,62908489	0,090478326
OR51A4	0,629412202	0,217681916
LTA4H	0,629498053	0,220657786
LOC642132	0,629722265	0,059005527
CALCA	0,629899376	0,374957031
DHRS10	0,629964656	0,446516372
RHOH	0,630009462	0,417384288
PFKP	0,630014512	0,205194785
SGK2	0,630147372	0,349286363
PTPN1	0,630168527	0,374849139
WASF1	0,631041078	0,014465199
HCCA2	0,631582213	0,759657718
SH3YL1	0,631759141	0,134067647
hCG_2000329	0,632073077	0,801006597
GPR101	0,632189785	0,517699543
C1orf223	0,632375083	0,532836257
LOC116437	0,632388976	0,173152463
LOC645481	0,632513002	0,219943997
LOC51619	0,632745547	0,100611169
WDR45	0,633874255	0,883153996
TCP1	0,633900745	0,180989873
C3orf37	0,634007539	0,306007438
LOC732156	0,634195362	0,806610188
C15orf57	0,634284055	0,36602348
LOC285047	0,634886706	0,084318192
C9orf46	0,634999564	0,068545734
FAM163B	0,63503833	0,036726746
TXNRD3	0,635165862	0,227863718
ANPEP	0,635384476	0,546640707
NAP5	0,635516083	0,031892475
LOC728181	0,635516104	0,103428731

PCYT2	0,636108601	0,119195
PNMT	0,636277396	0,097179797
GLUD1	0,636459202	0,455640055
VLDLR	0,636713638	0,56264159
NQO1	0,636794407	0,287934746
AMY1A	0,636984498	0,184584756
FGF6	0,637062488	0,141546129
PSMB5	0,637304835	0,050421497
PRDM2	0,637675425	0,123127922
PCAF	0,637872425	0,024183109
ANKS6	0,638248966	0,037052557
ACK1	0,638289841	0,70638358
MGC45806	0,638440111	0,126886551
FLJ30058	0,638485934	0,216523151
TMEM159	0,638491457	0,465526414
FLJ14129	0,638581639	0,255335256
CLEC4G	0,63882769	0,223577113
LOC727916	0,639051761	0,524174427
CSF2RB	0,639341787	0,945563091
ALG3	0,639523598	0,152221759
SYT2	0,639543176	0,353531424
MICA	0,639836332	0,075556404
NCL	0,639952584	0,063941542
AIDA	0,640109647	0,083093572
JTB	0,640134459	0,46708757
CHL1	0,640461771	0,12485252
DKK2	0,640597738	0,040312913
LPL	0,640688169	0,361500245
CCL7	0,640883757	0,099870977
FAM8A1	0,641019816	0,008812853
LOC729622	0,641229078	0,539996465
WDR81	0,641311113	0,124152254
SEC24D	0,641328065	0,328580517
ITGA8	0,641427376	0,45612084
BTG4	0,641523396	0,095687391
AMN	0,642117186	0,501420529
UBE2R2	0,642145125	0,196511052
LOC728445	0,642439043	0,174345329
FGF22	0,642501219	0,107030913
LOC728479	0,642515796	0,893993281
CD200R2	0,642683676	0,126521057
HRSP12	0,642949278	0,622730922
COG8	0,643305928	0,19216275
LOC644265	0,643413388	0,564391093
SHB	0,64345714	0,630744547

MT1F	0,643679561	0,627327979
C12orf31	0,643762091	0,15385541
FLJ22649	0,643969948	0,669594875
CCT3	0,644094335	0,094486394
LOC729649	0,6441623	0,057804733
AVPR1A	0,644218899	0,227166186
MAPK11	0,64488423	0,233411643
ARIH1	0,645222776	0,068543271
KLHDC1	0,645828895	0,481415694
BTNL2	0,645848519	0,592704706
CEL	0,645897317	0,28486587
RPAP2	0,645938612	0,1399507
CKAP2	0,646021428	0,162646264
LOC729624	0,646232495	0,033888335
SESN3	0,646328316	0,207393774
SNX3	0,646343174	0,230373327
PFDN2	0,646847989	0,573819351
FAM120A	0,647622355	0,023995154
PRMT7	0,647688458	0,062825071
DLGAP2	0,647790608	0,066585788
ZNF533	0,647856839	0,210992747
HTR3E	0,648029452	0,124894777
USF2	0,64810243	0,853935296
LOC731792	0,648671294	0,051773841
IMPAD1	0,648848763	0,112506814
WNT9A	0,64889934	0,149864493
WNT7B	0,649002076	0,92477447
ALG8	0,649039102	0,122613339
FES	0,649447	0,046335142
SERPINI2	0,649472851	0,072824689
TMEM89	0,649597388	0,023072434
LOC653052	0,649747932	0,07152908
FBXL18	0,650107067	0,444844733
TRAF6	0,650132117	0,067499081
CHRDL2	0,65029019	0,458883262
RWDD4A	0,650928984	0,163701894
OR1E2	0,651307297	0,17205925
C3orf41	0,651474697	0,052638643
FRMD8	0,65151281	0,085563673
GDF2	0,651921901	0,310500548
MGC23908	0,651942061	0,249672344
CSAG3A	0,652277128	0,142963486
C19orf41	0,652369368	0,158570792
ZHX2	0,65330583	0,145774261
CMTM1	0,653488782	0,062532865

LOC142910	0,65354214	0,082849273
MRPL49	0,65364805	0,200143103
SPRED3	0,653725979	0,301393937
OSMR	0,653780282	0,144329345
MYRIP	0,653797198	0,13188062
ZNF319	0,654024667	0,243879431
SPACA3	0,654050458	0,193081096
IL1F7	0,654157969	0,232270262
HOMER1	0,654170283	0,410426009
LOC728942	0,654395389	0,952550819
FCGR3A	0,654415738	0,441550036
NNAT	0,654453035	0,1565178
LOC400960	0,654779933	0,068768579
DLL4	0,654926041	0,053920294
CUEDC2	0,655054189	0,007869696
C11orf56	0,655137624	0,035030131
LY6K	0,655615097	0,285974519
ATCAY	0,655770315	0,071846233
BCAP29	0,655825059	0,035613904
FLJ25471	0,656407862	0,272631901
CERK	0,656446647	0,072490973
FANCE	0,656887505	0,829571965
LOC646817	0,656954292	0,001986524
GLOD5	0,657067074	0,839338931
ACVR2B	0,657474443	0,08867229
GCC2	0,657573161	0,137798259
KIAA0628	0,657632375	0,096462508
RAD54B	0,657796695	0,364927661
POLD3	0,658075725	0,126007632
LOC729283	0,658160269	0,210309819
OR13D1	0,658172362	0,30962436
MST150	0,658762148	0,140570403
TRAPPC2L	0,659043564	0,600618274
SMPD4	0,659284368	0,019880817
BET1	0,659585273	0,911259632
EDG8	0,659707504	0,229022969
TEDDM1	0,659712161	0,219333597
PNUTL2	0,659866382	0,206360901
SERPINB12	0,659924436	0,269424414
USP38	0,660833466	0,227992078
hCG_2024596	0,661142464	0,136113133
SIAT4B	0,661225724	0,415497472
DENND2C	0,66142274	0,124544367
LPPR2	0,661565527	0,502859887
ITGA2	0,661720218	0,220055558

FLJ44635	0,661756208	0,177848767
LOC729805	0,66218691	0,169961686
GDF1	0,662286725	0,713506398
ARMC7	0,662513963	0,046936944
LOC729461	0,662931463	0,044141397
KNS2	0,662966816	0,073938033
KIAA0427	0,663018577	0,025174754
NKAIN3	0,663526935	0,099651558
ZBTB22	0,663634044	0,086337514
GPT	0,66375002	0,06792058
C18orf19	0,663941537	0,044223525
DNAH5	0,664036165	0,135045274
ABCC9	0,664078999	0,156610173
ACSL1	0,664239859	0,545311133
NRD1	0,664397346	0,074367196
LRRC8C	0,664477537	0,464434073
LGR6	0,664612226	0,391042344
KRTAP4-1	0,664808899	0,141457355
ALDH4A1	0,665297558	0,031096643
UREB1	0,665499833	0,096816824
PRIC285	0,665519613	0,09961976
LAMC1	0,66581019	0,081747048
ERICH1	0,666044052	0,223620804
LOC729852	0,666059928	0,18475258
PNRC2	0,666308824	0,019372543
GOLGA8A	0,666310765	0,02221203
SEC16B	0,666627755	0,023762685
EPB41L1	0,666684539	0,126330833
LIM2	0,666874125	0,030959082
EDD	0,66731083	0,109744094
LOC730216	0,66768626	0,01380382
MORN1	0,668306763	0,089353381
C3orf14	0,668411217	0,937044147
HTRA3	0,668498047	0,040883526
MKRN2	0,668530562	0,272887476
IREB2	0,66887496	0,073028719
S100A5	0,66889463	0,071103052
OR10A4	0,668959651	0,197393822
MLF1	0,66896512	0,120953653
CHST8	0,668977407	0,192581459
SYPL	0,669160513	0,044227608
SLC17A7	0,669338152	0,698466132
IL1RL2	0,669420759	0,257934813
OKL38	0,669692837	0,11465453
LOC732159	0,67017647	0,08625785

TP53TG5	0,670290204	0,117460665
FAM19A1	0,67030512	0,194261621
CDC42EP1	0,670598438	0,065821794
IMPDH2	0,670727447	0,214760539
FLJ44124	0,6710107	0,858571396
FLJ36208	0,671428252	0,647491643
HELT	0,671541025	0,274961955
CCDC91	0,671628228	0,212329956
GANC	0,671636409	0,101222325
LOC392262	0,671639196	0,345602471
PANX3	0,671685007	0,286684062
LOC728805	0,671719739	0,054863004
ITGB1BP1	0,672133352	0,269156237
UCN	0,672201924	0,143699578
ABHD12B	0,672266471	0,315924365
LOC440302	0,672621019	0,109240182
PMP22	0,672651035	0,205194343
LOC727926	0,67279146	0,746019338
SLC39A12	0,672855571	0,091815642
LOC400581	0,672916553	0,080280569
MS4A5	0,673081851	0,045805131
MGC44669	0,673178658	0,027414516
LOC647927	0,673326379	0,018032433
NFATC4	0,673455815	0,79931863
RCN3	0,673930932	0,252661141
BCMS	0,674188846	0,251933609
DNASE1L1	0,674192743	0,54080676
C20orf27	0,674488569	0,294470444
NFKBIA	0,674490759	0,064249478
LOC732347	0,674490759	0,093479889
NKX3-1	0,674783182	0,103402145
STAC3	0,675068165	0,114642993
AKAP28	0,675229113	0,148022888
LOC729415	0,675357507	0,780195395
UTP14C	0,675681023	0,186956242
SCTR	0,675978708	0,320306823
FLJ46361	0,67598193	0,34255069
ADCY8	0,676225006	0,059475532
DYDC2	0,676232753	0,0601839
ZNF342	0,676398054	0,310115954
MORN5	0,676429881	0,199180141
HRNBP3	0,67651624	0,066837292
SUV39H1	0,676652218	0,028404374
ASB8	0,676816712	0,17038579
SRCRB4D	0,676873718	0,02320487

NRGN	0,677334843	0,158641183
C1QBP	0,677505689	0,20781872
CCR6	0,677647597	0,139416339
ATP6V1C1	0,677775432	0,515096609
MRPS21	0,677793082	0,04495376
APOLD1	0,677938936	0,453353709
LOC730181	0,678011868	0,336200607
HEXDC	0,678198026	0,233043351
LOC731865	0,678367684	0,047634955
SMG5	0,678482043	0,076940031
LPHN1	0,678535789	0,115940625
ANKRD54	0,678559744	0,128992326
HIP1R	0,678593504	0,104554723
LOC144481	0,678912737	0,191285632
EIF2C2	0,679064456	0,120322818
ZNF575	0,679075936	0,047108246
POP5	0,67913033	0,016194831
EIF2B1	0,679214282	0,212590482
LOC646677	0,679254903	0,098855699
ABCE1	0,679404993	0,074678376
C6orf199	0,679560316	0,084445524
NUP133	0,679571241	0,119599331
C11orf45	0,68010102	0,369821088
LOC644567	0,680603961	0,389636212
BYSL	0,680646551	0,137370562
LOC729161	0,680836553	0,133481685
ZFPM2	0,681009628	0,120315961
PIWIL3	0,681458268	0,411327535
SEMA4B	0,681722431	0,194206476
MAB21L2	0,681788454	0,01470431
LOC729947	0,682262363	0,027889717
PITPNM3	0,682547229	0,456619463
TIMM13	0,682862492	0,745567853
CEP164	0,682956361	0,016951702
NDUFB6	0,683231224	0,032402677
PGCP	0,683245151	0,344147098
LOC645716	0,683432323	0,171326865
C17orf68	0,683811623	0,183812628
DF	0,683889192	0,138913661
C8G	0,684045827	0,700506577
RAB3GAP1	0,684083773	0,187238344
GPR171	0,684150386	0,133038616
TRIM75	0,684411811	0,016442764
NF1	0,684706041	0,041720992
PGRMC1	0,684970289	0,017526209

SLC18A3	0,685266977	0,216045808
LOC727933	0,68582847	0,08006682
CLN8	0,686045992	0,113136237
OTUD6B	0,686172282	0,141398299
ELOVL2	0,686306401	0,072352303
GAA	0,686311982	0,457780416
CUBN	0,686823687	0,570307388
IFNE1	0,687198787	0,053590514
LOC651373	0,687269455	0,041186058
POFUT1	0,687301039	0,003997588
C9orf164	0,688736877	0,065840567
ODF2L	0,688742921	0,050777604
C1orf66	0,688879089	0,230486383
LOC728527	0,688980689	0,110238753
C17orf71	0,689179277	0,056400446
TEX15	0,689269932	0,679639573
TAX1BP1	0,689870053	0,111029641
SYTL2	0,689880694	0,823839405
PYDC2	0,690101863	0,178611293
HHIPL1	0,690110356	0,705773332
FLJ36144	0,690461238	0,356984809
FLJ36848	0,690609326	0,116511399
TYMS	0,691381983	0,06049912
SPINK5L3	0,691423261	0,405313234
ERN1	0,691470874	0,081471584
LOC727931	0,691971096	0,554729822
MRPL1	0,692337305	0,054358482
C6orf52	0,692536475	0,109295048
FOXH1	0,693115344	0,094311425
PABPC4	0,693498509	0,294693656
SLC40A1	0,693842023	0,026320994
OR2G3	0,694126588	0,117833776
TCF25	0,694335847	0,242386989
XAB2	0,694525768	0,34435048
MDS028	0,694532929	0,228923758
LOC728164	0,695209507	0,157739923
C12orf34	0,695700975	0,250719991
GPRIN1	0,696599641	0,054114049
SHC4	0,696868494	0,916415544
WDR68	0,697210934	0,525526532
NEDD5	0,697517377	0,301578882
LOC57168	0,697554264	0,092315501
CHMP4C	0,69765373	0,073564307
DDX28	0,697970351	0,062680502
TCEA3	0,697983855	0,199189304

RASD2	0,698171969	0,614005911
GPR4	0,698392398	0,080652721
LOC728439	0,698403058	0,083302567
AMSH-LP	0,698501397	0,35034765
MAD2L1	0,698668105	0,108705275
S100A1	0,6987037	0,296395927
ADIPOR1	0,698726929	0,223892311
ADPRHL1	0,698805729	0,222199177
MPHOSPH1	0,699027698	0,087115616
CYP3A7	0,699118184	0,121814265
OMP	0,699394271	0,333862228
ASB2	0,699686303	0,560190216
MAP4K4	0,699802177	0,753110542
FAM43B	0,700898087	0,219178755
PHF19	0,701042935	0,087950608
LOC651868	0,70117174	0,028213409
KIAA0430	0,701640451	0,092740153
LASP1	0,701711097	0,975060201
LOC727847	0,702034819	0,090811491
CAMK2D	0,702090722	0,5552584
LIN52	0,702163511	0,102120691
PNPLA4	0,702352644	0,276760652
SLC25A24	0,702429259	0,122508196
C9orf84	0,702632801	0,949643918
FLJ20084	0,702674218	0,00878608
ZFP57	0,703077535	0,171469395
DNTTIP2	0,703112183	0,346802455
SND1	0,703187415	0,040046656
HCK	0,703187822	0,046463629
SCAP1	0,703214667	0,072880466
FLJ11046	0,703287127	0,652054205
RTCD1	0,703780594	0,316765591
FRMD3	0,704022008	0,289006129
FLJ10490	0,704186802	0,119905014
NAPRT1	0,704188428	0,110370013
DNCL2B	0,704477304	0,705540892
PMM2	0,704610117	0,099486307
LOC402175	0,704897645	0,193591244
LYRM7	0,705052281	0,167554188
ALDH8A1	0,705385827	0,146313435
MLL4	0,705920757	0,480699007
INDO	0,70601417	0,984199206
RNPC1	0,70612448	0,123588276
LOC729122	0,706328982	0,254886552
C8ORFK36	0,706543702	0,356580222

PDGFRA	0,70680215	0,555472659
EMILIN2	0,706862296	0,313198068
DNAH3	0,706895685	0,22983979
SPIN4	0,70719258	0,432125223
MAPK6	0,707765129	0,405411175
C18orf25	0,708326888	0,745322362
LOC647049	0,708773832	0,038757988
ATAD4	0,708882539	0,610359015
LOC642009	0,709525296	0,113170342
GRXCR2	0,709602333	0,113839308
LOC731791	0,709612808	0,042303612
eIF2AK2	0,709757156	3,58E-72
CDH3	0,709958209	0,087082971
LOC727973	0,709974576	0,050644372
TBX4	0,710044428	0,644546073
ZNF10	0,710267135	0,192672004
LOC728654	0,710398528	0,644942026
LOC646851	0,710634476	0,107344844
C20orf12	0,710661082	0,308729603
LOC54103	0,710948731	0,023850279
SLC38A8	0,71106884	0,145470345
CHCHD7	0,711418345	0,424740216
PALM2-AKAP2	0,711505613	0,382888245
SNX8	0,711589222	0,168660912
HGFAC	0,711700695	0,288716364
LOC644497	0,712071795	0,186976983
C20orf7	0,712096514	0,019537101
SPTBN5	0,712789575	0,22881511
ARPP-21	0,712893634	0,047158467
AADACL1	0,713307365	0,112582758
LOC731210	0,713418437	0,052125967
CRB2	0,71358965	0,052284104
LOC651112	0,713886925	0,071413542
LOC729670	0,71401437	0,080332289
PIP5K1C	0,714100404	0,051917343
AOAH	0,714414497	0,122297742
OTX2	0,714433804	0,18445057
HMGCS2	0,714576996	0,162795841
TMEM164	0,714644432	0,135108167
LOC730139	0,714883621	0,070848273
C16orf61	0,714940244	0,028096625
OR1A2	0,715423069	0,265736649
KIAA1958	0,715505477	0,025048508
FLJ37078	0,715847891	0,064792131
ZNF532	0,715850983	0,230576127

LOC647380	0,71596551	0,010240529
MEF2A	0,71632012	0,110744876
ABHD9	0,716439502	0,971780692
LRRK2	0,716651478	0,294178392
PDC	0,717082424	0,129216873
ATF2	0,717315349	0,215787082
EXOSC4	0,717470622	0,219012763
LOC732187	0,71759534	0,093295698
DEFB32	0,717749238	0,026000039
CENTG1	0,717851808	0,05199029
COL7A1	0,718164942	0,160308189
CACNA1H	0,718261875	0,10223631
SKP2	0,718456502	0,01403787
PLCL3	0,718623456	0,656353048
HOXD3	0,718768686	0,110716261
OR4D11	0,718806652	0,316538459
MPZ	0,719297643	0,144533371
AGPAT3	0,71948346	0,020957551
TES	0,719581855	0,11391903
MTAC2D1	0,719650555	0,023358628
LOC388210	0,720164977	0,184674826
CCDC125	0,720257077	0,112536081
FGF2	0,720354313	0,04887169
LOC729177	0,720394359	0,290329312
KCNK6	0,720626977	0,076595587
LOC643878	0,720864854	0,900121869
LOC730017	0,721099543	0,678817826
TFG	0,721216704	0,026126814
LOC647216	0,721276387	0,14307123
SCGB1D1	0,721568461	0,115832104
PCCB	0,721580052	0,159354621
RNF18	0,721692016	0,166489354
PIP5K2B	0,72198653	0,284842602
CRYGB	0,722047499	0,183203773
MYSM1	0,722157427	0,181381013
TTC21B	0,722301491	0,051761334
AICDA	0,722575723	0,110934648
CL640	0,723218231	0,225993992
MOV10L1	0,723449164	0,172445005
NRG3	0,723524517	0,024751477
LOC647678	0,723620226	0,124222068
LOC729013	0,724202762	0,483330792
RAGE	0,724213362	0,253508248
AP2E	0,724386958	0,084227041
IGSF5	0,724480967	0,149578779

PRM1	0,724589817	0,547172729
AURKB	0,724902328	0,040324678
MTBP	0,725046994	0,024709918
PDGFC	0,725181265	0,235272011
SP3	0,725358483	0,185816383
USP9X	0,725653565	0,374204398
SAMD14	0,725705459	0,141313577
CDK2AP1	0,726181115	0,105126789
COL17A1	0,726234453	0,404127061
CYB5	0,72672442	0,018406139
LOC729567	0,726816627	0,212816826
RUSC2	0,726978598	0,030995255
NTRK3	0,726988812	0,163436526
SNCAIP	0,727013486	0,178472153
DPM2	0,72707239	0,18390103
DSCAML1	0,727115733	0,276659009
SKIP	0,727558005	0,050086063
C10orf72	0,727896109	0,817594484
ZNF138	0,727989899	0,500277153
TMEM169	0,728693866	0,115036146
C20orf166	0,72893877	0,035385364
STK22D	0,729170464	0,190442113
HYPK	0,729729907	0,213904042
MUC13	0,729783449	0,077666196
LRRN4CL	0,729889634	0,016669996
MTERFD2	0,729906289	0,000882864
GALNT14	0,72993929	0,061868686
PDIR	0,730384164	0,243223563
DPEP2	0,730543314	0,080576291
NT5M	0,730770364	0,588407526
C17orf97	0,731858175	0,064157698
SPOCD1	0,732036815	0,354472387
CD19	0,732811303	0,294847716
SNX19	0,732953694	0,55628188
ENTPD4	0,733129321	0,618277173
DPYSL3	0,733130944	0,60696849
ITGB8	0,733210803	0,362908269
PIB5PA	0,733477218	0,177427015
TMEM45A	0,733687094	0,137950569
ITGB3	0,733862574	0,219071185
TMEM205	0,733907957	0,478950143
NARG2	0,734657598	0,267103754
LOC729477	0,734662329	0,090512556
KIF24	0,734944437	0,573185476
FZD4	0,73496922	0,30709355

PAX7	0,735020715	0,782380739
LOC728537	0,73510698	0,047845104
DNAJA4	0,735150696	0,040472636
B4GALT6	0,73523292	0,232034816
LOC728200	0,7356152	0,098907259
PGM2	0,735627795	0,187723242
CXorf27	0,735754871	0,781122968
LOC644151	0,735888751	0,012453951
LOC729776	0,736527162	0,546911075
LOC729856	0,73670821	0,55109393
LEP	0,736744144	0,120599222
YDJC	0,736929067	0,150743475
LOC399829	0,737471266	0,345479042
RBM11	0,737681159	0,427872057
LOC388572	0,737818019	0,183912718
LOC731947	0,737980637	0,037192283
VTCN1	0,738115081	0,12705243
SH3KBP1	0,738520784	0,988120343
SERPINA6	0,738669914	0,458340794
CYBASC3	0,738673386	0,075459232
GCS1	0,738777921	0,072807118
FAM119B	0,738860355	0,022444524
TBCC	0,739385775	0,022517379
HMGA2	0,739401897	0,954286697
OR51B4	0,739403329	0,086091171
C1orf185	0,739577492	0,01924602
OR10X1	0,739696805	0,158691661
LOC441448	0,740209901	0,102412203
MRVI1	0,740246697	0,100185508
TCP11	0,740634453	0,015269926
NMNAT3	0,740839499	0,096561706
GPR23	0,740858233	0,062102218
LOC389230	0,741091054	0,179279743
C22orf9	0,741107386	0,250771475
LYRM5	0,741796234	0,096307967
TBC1D3	0,742001274	0,080205082
SERPINB7	0,742141097	0,014589804
FAM129A	0,742271133	0,428878748
LOC645602	0,742275805	0,050543891
C11orf35	0,742352664	0,30848233
hCG_1811337	0,742374636	0,08900602
MYOCD	0,74261511	0,177586407
LOC730908	0,743020653	0,77556272
S100A7	0,743050142	0,648087043
DDX17	0,743123077	0,112821953

CHRM3	0,743127652	0,457282443
ELA3A	0,743513925	0,089921886
LOC732335	0,743638821	0,024526192
C1orf123	0,744054087	0,454564948
DQX1	0,744437992	0,263073867
ARRDC1	0,744469553	0,171850961
XM_290793	0,744923821	0,553840529
GKAP1	0,745082649	0,279090979
C1orf225	0,745118739	0,069264875
WDR60	0,745346765	0,068757185
CNNM2	0,745541736	0,02965871
C10orf120	0,74555895	0,089697517
CCL11	0,745942649	0,08636575
C14orf73	0,745996732	0,059242062
PDE6A	0,74701367	0,409680663
SFRS3	0,747114559	0,245900564
CCT8	0,747121971	0,283229347
CASK	0,747178639	0,088989953
MRI1	0,747230097	0,014056926
REEP5	0,747404641	0,054639132
UCC1	0,747409913	0,004136516
TBX6	0,747600127	0,128320354
IL15	0,74775724	0,084104267
TMEM204	0,747845201	0,041849485
C11orf31	0,747972243	0,372875932
FER1L3	0,748155222	0,014365172
PRODH2	0,748304532	0,101491749
GMIP	0,748392087	0,041256414
LOC729066	0,748891096	0,425410346
TMEM80	0,74901666	0,308155672
LOC652517	0,750012138	0,734084979
CCDC127	0,750023605	0,304027156
CA9	0,750049268	0,005690056
RPGRIP1	0,750403245	0,031135462
POLDIP3	0,750419673	0,03136857
EMB	0,750889274	0,057943329
ZNF6	0,750982063	0,131344407
C3orf32	0,751239156	0,100833716
MXI1	0,751610438	0,133027098
IAH1	0,751660695	0,692180444
LOC732396	0,751793467	0,762271579
MS4A14	0,751807751	0,462137444
KIAA0205	0,752283962	0,966908078
LOC731227	0,752617637	0,084431214
C6orf170	0,753061048	0,129106282

LOC645895	0,753142796	0,039084158
LOC652755	0,753339301	0,008564642
FPGT	0,753476888	0,491530888
TCTN1	0,753492022	0,009327346
POU6F1	0,753583011	0,326368653
GGCT	0,753678881	0,800435891
LOC645864	0,753768243	0,052844794
PPP1R2	0,753867721	0,656609756
SDCCAG8	0,754371731	0,138054667
UQCRFS1	0,754476974	0,196596647
NRSN1	0,754501558	0,282171697
LOC645231	0,754989494	0,228264159
CNGA1	0,75508834	0,217231364
C4BPB	0,75509083	0,772079113
RP11-114H20,1	0,755859986	0,08057953
MGC45428	0,756046384	0,333961968
LOC728948	0,756128914	0,924697216
ADH5	0,756241704	0,201068246
DIP2A	0,756282733	0,002654296
MGC16169	0,756542069	0,578641146
CARD12	0,756551619	0,162396905
C1orf83	0,756793803	0,102579947
WARP	0,757005635	0,502458796
TXNL6	0,757317745	0,043474644
CHST1	0,757451561	0,247620062
C3orf45	0,757720527	0,083060401
AKT3	0,757936403	0,024607937
ADAT3	0,758254943	0,00234368
LOC730095	0,75841188	0,001119194
FLJ32685	0,758472086	0,063239577
CSNK1G2	0,758816966	0,134629316
LGALS8	0,758896768	0,00892965
WDR54	0,758924475	0,275388088
KIAA1522	0,759162092	0,21727025
PMS2L5	0,759447379	0,405702114
LOC729003	0,759676831	0,007579163
RARS	0,759882859	0,209235136
CACNB3	0,760006463	0,142662981
MOCS1	0,760171368	0,416364325
RNF180	0,760557291	0,418505279
TXNL4A	0,760890909	0,677804096
CBARA1	0,761284152	0,288439007
BAZ1A	0,761679281	0,219115934
KRTAP2-1	0,762090708	0,244844664
SLC6A6	0,762690379	0,074155337

PPFIBP2	0,762707997	0,047310345
LOC647983	0,762737475	0,160243522
ARHGEF19	0,762787339	0,349462122
LOC729734	0,763187503	0,032204321
VAV3	0,763319223	0,158929282
CLECSF7	0,763327134	0,236861801
BRUNOL5	0,764005848	0,081828845
INVS	0,764071651	0,797091773
MGC19531	0,764634847	0,151097902
CHRDL1	0,764832594	0,022591544
UNQ2963	0,765344165	0,466074574
PLEKHA6	0,765819035	0,10256046
LOC441795	0,766042503	0,670874296
LOC728907	0,766472815	0,789986242
C5R1	0,76654188	0,176189183
BAP1	0,766543258	0,417921513
ARF1	0,76664877	0,051532781
POU4F2	0,766774189	0,108641921
CD48	0,767283539	0,357485436
LOC642005	0,767658454	0,150973592
ZWINT	0,767717525	0,159998835
PSMC4	0,76784666	0,078295229
PRG-3	0,767942033	0,004559161
PRPF39	0,767960075	0,07687598
AP3B2	0,768206114	0,076672864
FLJ11286	0,768324019	0,253056086
EML2	0,768593415	0,014846272
PHF1	0,768606505	0,394748105
MAST2	0,768633207	0,009611775
ZNF285	0,768800243	0,251795012
HMGB3	0,769266638	0,627009371
CXCL16	0,769302051	0,458808959
TAS2R40	0,769963483	0,002146252
CCDC88A	0,770075593	0,058592894
FAM153C	0,770231611	0,076103193
OR8I2	0,770280001	0,365751492
EIF4E1B	0,770651818	0,414976284
TNFSF13B	0,770891145	0,216555105
IGHMBP2	0,770914264	0,324724819
FLJ44838	0,771109243	0,972801525
HEBP1	0,771840887	0,208768637
CD300C	0,77252729	0,138297829
PSMA4	0,772586777	0,050225222
FAM103A1	0,772681403	0,247994377
GABPA	0,772725218	0,341287136

ENC1	0,772765434	0,400724331
ASGR1	0,772917542	0,03341302
FADS3	0,773183669	0,013044898
VPRBP	0,773243621	0,176066746
HOMER3	0,773696777	0,15696226
WFDC10A	0,773888073	0,280740954
SH3BP1	0,774054261	0,501879003
LOC728129	0,774411359	0,509146981
LOC646139	0,774775675	0,020645387
CSRNP3	0,774909195	0,071879018
TP73L	0,775081014	0,360270867
PLEKHA2	0,775293707	0,673369579
C12orf55	0,775800316	0,544252611
TRIM46	0,775930609	0,47161427
SSTK	0,776125185	0,871729604
LY9	0,77614176	0,145154703
GRK4	0,776531177	0,126415288
FLT1	0,776537286	0,14345113
MBOAT4	0,776940404	0,048886354
ZNF615	0,777002761	0,150797425
TCEAL2	0,777055655	0,162791191
IREM2	0,777924979	0,327434255
SLC25A28	0,778006816	0,328923503
UBE2NL	0,778448084	0,070081392
LOC347119	0,778540127	0,034906581
DDIT4L	0,778616054	0,152671068
FNBP1	0,778958845	0,073095544
NCR3	0,779083832	0,110035855
POLA2	0,77910713	0,484924174
TDRKH	0,779230256	0,122497199
LOC732262	0,779366752	0,079204696
LOC284701	0,779519404	0,272907686
LOC729627	0,779577703	0,33380306
CD38	0,780008769	0,043651371
FAM3D	0,780076734	0,368529286
FLJ21156	0,780207284	0,242647139
DRAM	0,780300041	0,062612621
FAM82A2	0,780443282	0,46100196
C20orf151	0,780730856	0,141178593
AKAP12	0,781231593	0,323570378
LOC648822	0,781254861	0,003011672
C19orf31	0,781601202	0,455603665
LRRC45	0,781909145	0,215879562
LOC728504	0,782179768	0,060640697
MAGEH1	0,782180685	0,081359169

CR1L	0,782686528	0,912005924
TFAP2C	0,782793516	0,261306722
RAC1	0,783157402	0,626669142
PRRT2	0,783253198	0,059227104
TPK1	0,78361668	0,588991093
PRB4	0,783962579	0,107878686
ADCY4	0,783967209	0,042796901
CPNE5	0,78447679	0,357007226
LOC440498	0,784495368	0,33502424
OTUD5	0,784816146	0,028298923
CCDC44	0,784847436	0,069936251
TGFA	0,784864643	0,8132566
ARHGAP10	0,785007555	0,085250402
C11orf17	0,785551018	0,026422476
C3orf39	0,785807921	0,164944407
LOC647500	0,786205316	0,543187153
TMEM176B	0,786589051	0,234656037
LECT2	0,786692168	0,222614563
C10orf116	0,786747366	0,129465166
SLC36A2	0,787333059	0,057792066
PDRG1	0,787407761	0,205348707
PSMB2	0,787522421	0,202992516
HEPN1	0,787667433	0,514155995
XRCC5	0,787939869	0,431828817
SYT10	0,787995553	0,168308084
ITPRIPL2	0,788298543	0,128690192
TIMM8B	0,788470056	0,128254294
AMPD2	0,788699621	0,013230762
CPA4	0,78887941	0,288440955
NOMO2	0,78899718	0,035363177
SLC6A20	0,789130565	0,347600284
C5orf40	0,789420691	0,00245799
C5orf41	0,790094196	0,161420661
FRY	0,790543389	0,366171296
KRTAP4-6	0,79117079	0,2268396
COX7A2	0,791175159	0,631881162
FLJ45187	0,791652983	0,118987494
EPHA7	0,792058597	0,200209887
TTLL2	0,792081309	0,103546899
PIGT	0,792218219	0,104050832
LOC650262	0,792380561	0,032727304
MYPN	0,792659818	0,162531212
ZNF306	0,792670522	0,958200564
LOC649486	0,792690613	0,002781891
PLAC8L1	0,792733323	0,283544878

ZGPAT	0,792953468	0,047480842
CGI-72	0,793087081	0,03641282
ELK4	0,793101336	0,646694266
LOC653879	0,79313143	0,277352213
LOC146429	0,793219388	0,942775825
UCP2	0,793362727	0,563698099
PTPDC1	0,79384507	0,185801072
CDC42EP4	0,794319512	0,01027181
NPHP1	0,794337687	0,345137406
LOC441476	0,794398056	0,038438507
LOC728692	0,794624359	0,479077118
GLT8D3	0,794905904	0,323661128
LIMK1	0,795529489	0,021504674
ZSWIM3	0,796103875	0,0066869
ARMCX5	0,796163663	0,019619193
DGKA	0,796492167	0,048094634
RAP2C	0,796595817	0,071311105
SCAMP5	0,796604048	0,068646784
CRIPT	0,79664362	0,167755515
LOC134285	0,797088695	0,24367423
SLC35E1	0,797183561	0,085655369
SLC38A7	0,797207936	0,074662062
STON2	0,797772118	0,790846991
SPRR1B	0,797987733	0,120820713
LOC731940	0,798618936	0,010312127
COMMD1	0,798659365	0,110878945
CPT1B	0,799168391	0,736169909
ANKRD29	0,799180416	0,108380074
C12orf71	0,799423157	0,200381568
SNTG2	0,799434077	0,269685535
ASB13	0,799874013	0,179494871
LOC729537	0,79988156	0,804042057
LOC440742	0,800037431	0,020508786
LDLR	0,800225786	0,139413256
VMD2L1	0,800350571	0,116633095
HLA-DRA	0,800857472	0,056341211
BF	0,801078738	0,587430106
THUMPD1	0,801756161	0,183425022
C3orf26	0,801989002	0,016071471
PCDHA13	0,802217601	0,225155056
HEATR4	0,80298851	0,121872438
TCL1B	0,803139994	0,006526483
ABT1	0,803392424	0,146141272
TP53I3	0,803408392	0,41325637
LOC730078	0,804182887	0,340683705

LOC650883	0,804295559	0,173521852
LOC646482	0,804325484	0,117393374
GRAMD1A	0,804448397	0,144924464
TMPO	0,804538115	0,152839727
COL18A1	0,805133595	0,048458476
LOC646570	0,805149501	0,289999402
DDOST	0,805434633	0,227025691
C19orf34	0,805470156	0,052002645
SLC7A6OS	0,805521815	0,108106588
PNRC1	0,805558353	0,09278471
FBXO38	0,805643463	0,275389708
LOC729504	0,805751191	0,133807496
KIAA0586	0,805876467	0,142770679
C11orf83	0,80617868	0,05248476
MRPL3	0,806603983	0,455073739
RASGRF2	0,80666176	0,28988654
GMFG	0,807131307	0,096980073
LOC643624	0,807309493	0,389729912
CD274	0,807394818	0,270561345
LOC146712	0,807431492	0,125822587
CDC42EP5	0,807433151	0,010644357
APBB2	0,807646368	0,121672239
OR5R1	0,808249451	0,775051821
CER1	0,808808245	0,073890939
ENTPD5	0,808898411	0,094821473
ZNFN1A1	0,809224849	0,532319546
CDH10	0,809290182	0,012516879
BLNK	0,809777325	0,429453295
CD28	0,810103348	0,07556876
FLJ23751	0,810589518	0,042725706
OSTC	0,810602772	0,086230376
BMP8A	0,810694162	0,126846794
FBXO34	0,811837431	0,140555889
SH2B2	0,811974571	0,01005487
NKX2-6	0,812095588	0,067709588
LOC400958	0,812414879	0,674543473
DYDC1	0,812815548	0,065273103
DDX23	0,81284616	0,312982224
CRABP1	0,81300363	0,448660407
LOC388630	0,81302011	0,072414987
MLYCD	0,813333364	0,132184126
GGTLA1	0,814554984	0,211370643
LOC728991	0,815931016	0,044453683
OR13C4	0,816246161	0,170660902
CECR2	0,816264824	0,034317939

SMAD4	0,816291735	0,122491433
RNF34	0,816477268	0,018968362
HERPUD1	0,817747028	0,067264515
ZNF366	0,818362625	0,182663278
MGC9712	0,818705484	0,025659139
HSFY1	0,818976811	0,602392224
SYT14	0,819193683	0,036244969
FAM47C	0,819319094	0,011988883
ETFB	0,819356631	0,074928266
LOC645687	0,819421074	0,015523677
AZI2	0,819661394	0,212489283
TCF4	0,819683527	0,013334634
DER1	0,819691478	0,133861147
LOC730911	0,819922072	0,121460017
LOC642662	0,820042188	0,035736781
MAML2	0,820330124	0,032525803
ATXN7	0,820490447	0,115754346
C16orf35	0,820931842	0,378878661
OR1N1	0,820951206	0,230367416
SCN5A	0,821014449	0,176471517
CIRBP	0,821624019	0,004188214
NHLH1	0,821747362	0,193925214
CLPTM1L	0,82189351	0,004732566
CD37	0,82277864	0,264661988
HVCN1	0,822914241	0,41489664
MGC2752	0,823030891	0,759696691
LOC728135	0,823132386	0,007400552
NSDHL	0,823167828	0,173822436
E2-230K	0,823256217	0,048703204
FLJ22662	0,823509726	0,409035349
USP39	0,823690827	0,511882751
CCDC129	0,824175829	0,017790729
HUS1	0,825199169	0,157891775
MAG	0,825330644	0,140510243
LOC730131	0,825556428	0,150329917
AP1S1	0,825700804	0,054340323
STARD6	0,82570807	0,041160264
LOC257039	0,82598268	0,053191477
CLUL1	0,82614599	0,09728051
TATDN2	0,826231448	0,910252637
GMPS	0,826440212	0,788642594
TPT1	0,826670183	0,060385911
IFNG	0,826723562	0,008045162
ERBB2	0,82674341	0,315694616
TACC3	0,827361884	0,068649157

BBS5	0,827976439	0,068776916
CASP3	0,828095581	0,072835015
SPRYD5	0,828255841	0,358761091
ATF7	0,828363635	0,076405544
TMOD2	0,828484042	0,085720772
ZNF9	0,828766735	0,256504873
CNTN6	0,828908611	0,028602566
WDR32	0,829066778	0,015203643
CCDC76	0,829806811	0,108623808
NACAD	0,829850924	0,120793855
SIVA	0,8302259	0,312477835
YIF1B	0,830652275	0,081481161
LOC728045	0,830681357	0,347766112
PIGS	0,831429077	0,033961769
PPP6C	0,83149924	0,176991646
LOC283104	0,832182547	0,111307136
GLTSCR1	0,832185718	0,114732369
PSTK	0,832331545	0,165365511
ASPSCR1	0,832476577	0,062486457
TUBGCP4	0,832696309	0,107710502
LOC441282	0,833322632	0,015137294
SEMA6A	0,833807379	0,159152212
STX18	0,833865432	0,339209826
hCG_1813818	0,833992632	0,447022715
LOC642721	0,834048326	0,115499113
DFNB59	0,834121158	0,148351801
TULP3	0,834226651	0,569077391
LOC727998	0,8352231	0,011198706
C14orf21	0,835916257	0,046555255
ARMC4	0,836238234	0,088789831
C2orf51	0,836718233	0,06233276
MLLT1	0,836852649	0,061406274
LOC728685	0,836961441	0,366946757
LOC729385	0,837099675	0,129901656
LMAN2L	0,837679081	0,093153645
NUCKS1	0,83778749	0,037374361
ARHGEF3	0,838025247	0,784113233
PCDH12	0,838045588	0,020470135
FAM180A	0,83842313	0,320754802
LIMD1	0,838628312	0,142832747
QSCN6L1	0,838800652	0,192337014
UTS2D	0,839456297	0,902700283
LOC728210	0,839662985	0,392681667
CTSO	0,84001035	0,226609343
LOC729229	0,840016175	0,114429019

BTN2A2	0,840083456	0,073643773
FERMT1	0,84072979	0,193894659
LOC729029	0,840733846	0,187026694
RBP3	0,840835779	0,218116806
LOC388199	0,840943126	0,071099588
SYNE1	0,841197217	0,034567692
UGT1A3	0,841491916	0,163736046
C6orf81	0,84151025	0,13178622
P4HA1	0,841828016	0,372874445
CPA3	0,84197413	0,069141768
TNS1	0,842776091	0,123942539
CDCA8	0,842787377	0,221306389
BRD9	0,842886655	0,028374861
LOC729369	0,843122	0,089258131
MRM1	0,843125566	0,521244353
PHKA2	0,84333628	0,23276316
LOC652834	0,843448667	0,192213133
C15orf19	0,843790536	0,218606638
KREMEN1	0,843805875	0,449543611
UBQLN1	0,844397759	0,198697012
PAI-RBP1	0,844522443	0,43954918
EME1	0,844788661	0,156845626
BANK1	0,846077133	0,220481104
DNMT2	0,846254353	0,170020521
LOC729740	0,846518034	0,089117733
PTPN3	0,846638774	0,130035533
ORC1L	0,846685042	0,507412346
MARVELD3	0,846931431	0,365831397
CCDC134	0,847656737	0,164143233
PIWIL2	0,847728812	0,870395363
C9orf169	0,847849049	0,062145961
TNFRSF10C	0,848036025	0,067740383
LOC729000	0,848173814	0,17219385
ELAVL2	0,848186272	0,448388307
C10orf118	0,849019909	0,055976893
ECAT8	0,849070082	0,208202107
LOC729910	0,849149493	0,012308984
FOSL1	0,849241237	0,650618362
KRT2A	0,849382932	0,133135993
RUNDC2A	0,849540269	0,453605824
CLCN6	0,849608837	0,372679418
AQP8	0,850447274	0,222933205
C16orf33	0,850751294	0,024263549
GARNL4	0,850991176	0,37161841
FBXO42	0,851111223	0,056210657

GIT2	0,851375962	0,130912518
SDCBP2	0,851598589	0,000844659
MST4	0,851670605	0,369785191
VPS13A	0,851697551	0,060385624
NOSIP	0,852052306	0,149921258
DDX26B	0,852110401	0,154940061
FKBP5	0,852117587	0,431313418
ACSS3	0,852342083	0,382080153
DCLRE1B	0,852349976	0,051913126
LOC732454	0,852528594	0,021504797
KIAA0692	0,852539319	0,116624799
SSBP1	0,853143625	0,076326513
LOC652821	0,853231381	0,173674527
LACTB	0,853447079	0,230302714
LOC728730	0,853460279	0,183079467
RPS3A	0,853515047	0,266869799
PRDM11	0,854217827	0,341185387
ERN2	0,854245106	0,105519552
CXorf61	0,854492345	0,509212297
SET	0,854498017	0,10219533
IF	0,854579446	0,42825492
S100A9	0,854660338	0,131764676
C2CD2	0,85506112	0,241528527
FBLN1	0,855264307	0,082451984
SUPT3H	0,855299408	0,003339671
LOC284288	0,855530725	0,072606666
LOC652661	0,855758896	0,079392929
FLJ43950	0,855970773	0,078635283
LOC345630	0,85607337	0,123824415
GRM1	0,856312551	0,140533592
MRPS18A	0,856746955	0,107259547
NCDN	0,856751401	0,089753942
NDUFAF2	0,85748016	0,230693741
ZC3H12D	0,857537693	0,979696146
ZNRD1	0,857821755	0,118450761
LOC646040	0,857906525	0,68576083
GKN2	0,858152823	0,472354332
DIAPH3	0,858275068	0,100939175
GPR19	0,859038084	0,171029563
TCP11L1	0,859185641	0,102308612
LOC387700	0,85949839	0,326778804
FNTB	0,859712449	0,08401499
PROK2	0,860120601	0,357619114
N-PAC	0,860541994	0,433475146
LOC644974	0,860566836	0,025351537

FAM78A	0,860641471	0,094268163
PLA2G12B	0,86090302	0,024494521
ARL6IP5	0,861230184	0,351758301
LOC732004	0,861332405	0,128839312
DKFZp566O084	0,861336352	0,347668707
FBXO48	0,861667445	0,053070888
CGI-04	0,862034168	0,302926727
NDUFAB1	0,862183948	0,181797809
C2orf44	0,862213405	0,030662318
LOC149478	0,862940313	0,257190162
C14orf104	0,863146856	0,369777248
OSGEP	0,863182094	0,089693428
SAGE1	0,863191702	0,122735064
C8orf38	0,863334572	0,06824671
DEFB126	0,863697772	0,387197534
FLJ40217	0,863924258	0,17306749
MGC21394	0,86479491	0,047185789
GPR10	0,865013908	0,044221632
MOG	0,866104815	0,081879669
ZNF480	0,866143792	0,37447174
KNSL7	0,86623209	0,167103535
KRT6L	0,86673896	0,037589831
FLJ13224	0,866923917	0,08397854
SIX4	0,866974348	0,151886044
OR14A16	0,867276737	0,00439982
MGC30208	0,867340016	0,022295808
FANCB	0,868085773	0,01699215
C6orf26	0,868095364	0,043336078
RNF152	0,868441292	0,26781237
ZNF175	0,868532049	0,130516279
MTUS1	0,868775637	0,187375842
ZNF253	0,868939178	0,07764504
CARD14	0,869496389	0,119478671
DNAH17	0,869508245	0,226852267
ZNF827	0,869533483	0,068762715
RBPMS	0,869658759	0,092307626
FSTL1	0,869704098	0,082390823
COX7A1	0,86983184	0,299328481
TRIB2	0,870343424	0,211417029
C10orf93	0,870508911	0,179305239
DFFB	0,870853853	0,116795849
DNAL4	0,871417392	0,132965489
LOC729897	0,871640077	0,101237221
LOC729532	0,871818966	0,292742542
CHRAC1	0,871839479	0,147415042

DEFB110	0,873057734	0,268374613
VSIG8	0,873154636	0,569575513
LOC646675	0,873473538	0,189548194
ULBP3	0,873588706	0,156918926
SAR1P3	0,873612237	0,02287825
ST13	0,874169677	0,030506382
ABCA8	0,874229853	0,113634066
MEOX1	0,874655749	0,110500451
RAB39	0,874703349	0,277447441
FLJ41481	0,874754417	0,218115528
C2orf43	0,874957858	0,082851252
CD9	0,875837782	0,216376428
MMP23B	0,876045812	0,084289174
TRPV1	0,876362926	0,520041906
C19orf51	0,876412333	0,01143659
LOC730644	0,876538489	0,106120668
VMD2L2	0,87665055	0,210691312
LOC730208	0,876892271	0,095011289
NLRP2	0,877129809	0,050648514
HSF1	0,877246283	0,134413702
FAM78B	0,877831472	0,047236962
KRT26	0,87834996	0,024698209
TFPI	0,878939791	0,13388169
SH3GL2	0,879062937	0,062736767
TRIM43	0,879113868	0,078922303
KRTAP4-2	0,879122849	0,191397955
LRP10	0,879158356	0,325722526
SSR4	0,879369315	0,92647921
GGA2	0,879383863	0,503760999
CCL28	0,879577377	0,975822755
FAM3B	0,879604465	0,057174724
ZNF559	0,880721784	0,100467158
LOC642340	0,881009517	0,01991251
MGC45564	0,881382322	0,979094906
FAM122B	0,881506616	0,098451186
CCDC90A	0,881955966	0,11198023
FAM151A	0,881993467	0,462645632
C6orf166	0,882437973	0,239547828
OXER1	0,882440563	0,065636708
SCDR10	0,882712963	0,97580934
PGS1	0,88297722	0,241569095
TEKT4	0,883041229	0,096666367
KRCC1	0,883948965	0,022896815
UGT2B11	0,884150563	0,278795477
TM9SF4	0,884866639	0,661652593

TGM3	0,885656197	0,239187415
LOC727814	0,886192147	0,024354262
LRRC59	0,886387318	0,226113833
ARL6	0,886441777	0,086361654
FBXL16	0,886943648	0,291906111
LNPEP	0,887211022	0,09047302
LOC727943	0,887355797	0,091397722
HOXC6	0,887442537	0,233552191
LOC120376	0,887500541	0,101101451
RAB5A	0,888161771	0,027942713
TRPV6	0,889216132	0,501967364
ACY1	0,889263446	0,141482773
C19orf57	0,889539039	0,026872613
LOC645448	0,891383419	0,850265438
GYPA	0,892394253	0,016419269
CCL18	0,892660909	0,209732117
PRPS1	0,892826911	0,322030605
HSPB3	0,893353229	0,059857778
LOC345537	0,893730831	0,374225568
PLXDC2	0,89376905	0,235840658
POLR1A	0,894557868	0,179460582
MICAL3	0,89482682	0,013357897
LTBP2	0,894929415	0,042984043
DLL1	0,895037048	0,577549476
FGFR1OP	0,895522583	0,230912422
GART	0,895535307	0,184101282
LOC642934	0,895549871	0,109665978
PARVB	0,895680314	0,211960053
LOC442501	0,895810506	0,733710078
TRMT61A	0,895963326	0,004263769
LOC641912	0,896204957	0,048677042
TSC2	0,896450433	0,18653555
C10orf88	0,897143414	0,054609671
LOC645971	0,897225804	0,125962306
C16orf7	0,897342346	0,07531027
LOC390877	0,897838549	0,149268384
NDUFS8	0,897993585	0,026930443
SYMPK	0,898030231	0,216866718
USP4	0,898167356	0,046349917
FLJ41170	0,898430603	0,016651364
LOC113230	0,898747554	0,267836445
AMPD3	0,899151721	0,055844026
TMUB1	0,899178587	0,163026295
LOC642677	0,899287847	0,082157072
GTF2H4	0,899810713	0,06223784

IL3	0,899873204	0,014449408
KCTD5	0,900285538	0,047812887
LOC646034	0,900650049	0,029933358
TMEM208	0,900919609	0,062622673
PTCHD1	0,901010333	0,263765127
S100A8	0,902181663	0,051125472
NPY5R	0,902271009	0,003204634
HNRPR	0,902919615	0,333911571
TLR2	0,903111913	0,212726662
FLJ40296	0,903231692	0,913953712
LOC127099	0,903357656	0,335033549
MATN3	0,904120503	0,130724602
RANBP10	0,904165041	0,711229184
C13orf11	0,904201278	0,106795651
LOC652692	0,904574796	0,944268598
PCDHB3	0,904717782	0,078188473
MRPS24	0,90487928	0,071070133
POP7	0,906012632	0,026953423
NOS2A	0,906037437	0,087484012
FAM83F	0,906158941	0,029732098
RPGR	0,90657213	0,001787782
PSMG2	0,90670582	0,164722565
BAIAP2L2	0,906805517	0,090166782
RGS12	0,907397112	0,066780299
CTNNAL1	0,90761239	0,834995012
LOC728148	0,907887752	0,032144289
LOC389365	0,908067761	0,102769854
UNC5C	0,909622216	0,01475899
FAM69B	0,909724282	0,095883928
GPR26	0,909887664	0,86340135
LOC389676	0,910371823	0,058136423
ECE1	0,910694343	0,114072266
LOC149420	0,910834273	0,319727325
LOC645652	0,91099532	0,221785588
LOC729468	0,911362202	0,082218382
CCNE1	0,911487925	0,04276298
FAM76B	0,911631666	0,142269964
HCFC1R1	0,911953414	0,037705607
INPP5F	0,912238098	0,041487623
RXRB	0,912695698	0,534608174
LOC646484	0,912930851	0,022748975
YTHDF3	0,913452301	0,241184171
LOC730009	0,913489322	0,238040737
BCL9L	0,913577753	0,164968462
hCG_1790474	0,91426619	0,029151386

B3GNT6	0,914335336	0,061764126
DEFB112	0,914391286	0,065444472
PKHD1L1	0,914574626	0,22313448
PERLD1	0,914589492	0,049431655
LOC730109	0,914649661	0,045803265
LMO2	0,914934817	0,036640431
SCOC	0,914961604	0,135192109
KIAA0319	0,915381324	0,218061832
PDHA2	0,915433707	0,079582501
IFNB1	0,915462459	0,162339871
BEX4	0,91546357	0,185541677
MYOC	0,916190726	0,179850973
NT5DC2	0,916330315	0,096282282
TRPM3	0,916875779	0,390297937
SLC35B1	0,916895049	0,06254954
CAGE1	0,91697666	0,065503379
FLJ23152	0,917179416	0,243903891
PRKX	0,917392538	0,753908944
KANK2	0,917786057	0,258106811
MYOM2	0,918701568	0,086104994
YIPF3	0,91947841	0,727152483
PEMT	0,919522101	0,120193692
PPP1CA	0,919627076	0,052726476
LOC728709	0,920263078	0,456760879
OSGEPL1	0,920497419	0,056518086
IRX1	0,921087667	0,765963945
TNFAIP8L3	0,921337944	0,132211248
LOC645218	0,921812183	0,585949111
CCDC41	0,921818961	0,001128717
ADAMTS4	0,92201037	0,101858164
ATXN1	0,922041716	0,086319897
MOSC2	0,922844985	0,030670885
MGC1136	0,922884243	0,009241854
LOC649618	0,922890577	0,313627993
TMEM119	0,9230495	0,769776447
ZNF433	0,923808222	0,032724415
SAP30L	0,924031873	0,051798367
ANKHD1-EIF4EBP3	0,924660705	0,746709885
NFATC2	0,924887016	0,259768287
CDKL2	0,925061507	0,173258232
C10orf62	0,925206485	0,692721392
LOC652565	0,925358325	0,070923285
KIAA1128	0,925686416	0,135059769
LOC390595	0,925815876	0,330166983
MYLIP	0,926176843	0,141774562

TFPI2	0,926229928	0,04897266
LOC730034	0,926379488	0,27421193
C4orf44	0,926595962	0,391204903
IL1F9	0,926694867	0,016065683
CCT7	0,926764343	0,057563072
SUMO3	0,926818944	0,001936323
GALNT6	0,927097576	0,184905196
PKLR	0,927173735	0,135141953
KLF15	0,927404088	0,09280204
LOC645100	0,927665519	0,058529854
RABGGTA	0,927765784	0,048880071
LOC644937	0,927776148	0,059647747
MGAT3	0,928201389	0,209488133
MEGF11	0,928299101	0,42570973
C11orf58	0,928520206	0,031795964
FAM73B	0,929353982	0,220301885
LOC652417	0,929354087	0,093128396
C4orf45	0,929912594	0,382334001
LOC730250	0,930008609	0,187308331
AACS	0,93023757	0,11705243
WDFY2	0,930313212	0,011186825
LOC732448	0,93085897	0,161329309
C1orf201	0,931293443	0,001444124
C2orf55	0,931900615	0,052165664
AP1GBP1	0,931970945	0,577973294
DKFZP686C2281	0,932142668	0,02993869
MRPL23	0,932973037	0,285934855
FLYWCH1	0,932983643	0,024124064
EFNA3	0,932992004	0,366412165
CXXC4	0,933209305	0,16146855
RHOT1	0,933732232	0,056430342
LOC442572	0,933960457	0,05567045
ZNF233	0,934713431	0,070730419
FLJ46266	0,935057807	0,083693967
DDN	0,935871621	0,005893231
TMEM16C	0,936191605	0,197741204
DRD1	0,936316346	0,068045313
C1orf91	0,936350553	0,23868647
SH3BP2	0,936484258	0,155141175
LOC283440	0,936620381	0,093359684
HIST1H2AM	0,937171104	0,03879763
ARL3	0,937249829	0,035542441
LRP12	0,937833295	0,065270969
RIN1	0,937917519	0,019391503
C14orf50	0,93839259	0,196183316

WNT9B	0,938709379	0,442956509
LYVE1	0,938818391	0,603918447
SBDS	0,939027327	0,687170396
GPR65	0,939485975	0,184367832
NPM2	0,93950515	0,117348857
GFI1	0,93987063	0,280416468
SVIL	0,940075533	0,775327319
RABAC1	0,940263633	0,115920237
SAA2	0,940461697	0,218383395
OR8D1	0,940607963	0,237080123
GPR82	0,940971249	0,133653646
SRGAP1	0,941277924	0,411271536
PRKAR1B	0,941828095	0,141311601
CUTL1	0,942040362	0,187928668
WDR65	0,942900169	0,193364666
LOC729833	0,942942758	0,049972619
DEF8	0,943112652	0,358246442
ME3	0,943275073	0,189703805
RLN3	0,943795996	0,125015641
ACSM4	0,943826183	0,132727441
ZNF81	0,943962856	0,121197802
IGLL1	0,943996052	0,003986207
CCRK	0,944274044	0,343621286
ALS2CR12	0,944291638	0,11038263
ISCA1	0,944357292	0,051101509
STOX1	0,944522538	0,48364017
NMS	0,944676245	0,128086176
FAM86B1	0,944726401	0,032747129
SEC14L3	0,944766428	0,029011694
PCBP4	0,944774591	0,016108506
PMFBP1	0,945866729	0,203015513
CYBRD1	0,946188256	0,104395029
GOLGA2	0,946746774	0,063421485
FOLH1	0,94725331	0,112568482
TMLHE	0,947315019	0,122555835
TMEM54	0,94735814	0,229330224
LOC645521	0,947631434	0,964633565
FAM167B	0,948140303	0,03226101
ATP2A2	0,948260805	0,242631559
IFI16	0,948988249	0,268852436
TNFRSF13C	0,949038217	0,125601972
PC4	0,94909042	0,03699447
MAGOHB	0,950138039	0,544092916
LOC728268	0,951171786	0,638098674
TNFAIP1	0,951366934	0,109461454

LOC730037	0,951544424	0,104692154
LAP3	0,951717838	0,251622581
LOC729589	0,952455552	0,15018322
RPL13A	0,952762999	0,381623844
CYP4B1	0,953233473	0,047624049
LOC731945	0,953235766	0,087927516
RANBP2L1	0,953286796	0,168097567
LOC729501	0,953773968	0,054205657
BTBD4	0,953817922	0,294604926
CXorf56	0,954012972	0,12047839
ITGA9	0,954423486	0,095220227
MSC	0,954507023	0,101726172
FLJ41423	0,954680397	0,409418938
TFRC	0,955647929	0,039865593
NR2F2	0,955691462	0,085572311
GJA3	0,956082299	0,072331329
LOC652627	0,956656395	0,204289826
LOC731909	0,957016419	0,515923977
EPB41	0,957043385	0,064111021
PFAS	0,957479663	0,906938071
DMBT1	0,957712806	0,079731595
RPS24	0,957861678	0,103082167
SIX6	0,957946624	0,051869931
HM13	0,957980578	0,172976656
MTCH2	0,95802935	0,209494636
FBXO30	0,958083627	0,011126454
KLK11	0,958274729	0,024599351
RNASE2	0,958604223	0,641443323
MORG1	0,95935416	0,052393002
FLJ45974	0,959530309	0,192675154
GPR174	0,959532609	0,049678199
MGC35440	0,959898918	0,346910548
OR4F29	0,959939691	0,060986199
ARHGEF16	0,960749454	0,316032633
LOC729485	0,960981124	0,062687524
IRF4	0,961257495	0,066906546
UGT1A8	0,961465452	0,055748051
CCDC5	0,961697319	NA
CCDC151	0,9618101	0,106827074
PITX3	0,962214903	0,480305895
TNFSF15	0,962386839	0,030331609
OXA1L	0,962520358	0,033870781
SEPHS1	0,962637154	0,097904366
WDR82	0,963030139	0,03009002
LY6G5C	0,963312309	0,454729963

TMEM50B	0,963686714	0,109936578
AKAP11	0,963702632	0,213943959
PB1	0,964151894	0,036888117
PLK1	0,964339883	0,014036652
MFGE8	0,964767178	0,104734023
EXOD1	0,965403255	0,12743825
OR10A6	0,96558366	0,134225641
LOC729297	0,965847256	0,229397371
AKNA	0,965899618	0,391512101
CTF8	0,966171839	0,076866634
PTK2B	0,966935516	0,038175788
FLJ20277	0,967267427	0,167222122
NTN2L	0,967391775	0,115978816
PNR	0,968150315	0,19466836
LOC641796	0,968172662	0,010038952
CSF3	0,968302158	0,305459591
NRN1	0,968524316	0,127995664
UNC5A	0,968749438	0,51353766
ROCK2	0,969028025	0,115961599
WEE1	0,969199408	0,07891227
HEBP2	0,969270649	0,140519984
HAPLN1	0,969772813	0,224710023
RBM9	0,969840549	0,318669694
MRPL44	0,970462051	0,053393352
TFF3	0,97065462	0,125920194
PRKACA	0,970732182	0,279183184
PSCA	0,971195124	0,052521086
NBPF1	0,971465528	0,054259596
SIDT1	0,971618855	0,115712346
WDR88	0,971633708	0,44223937
ARL7	0,972678005	0,105884704
CTDP1	0,973390833	0,006611446
FLJ23233	0,973589103	0,063646511
PSCD1	0,974302514	0,182640071
LOC147646	0,97462505	0,003610934
OPRM1	0,974658903	0,097731222
LHX2	0,97544407	0,111385145
BHMT2	0,975559052	0,111542448
FAM116B	0,975602532	0,08219431
MID1IP1	0,975923443	0,135088176
LOC728063	0,97604959	0,095944787
GLB1	0,976649518	0,016635609
CREG1	0,976832358	0,077556967
GCN5L2	0,977023247	0,148170447
RHAG	0,977465751	0,172263049

TEX2	0,977706229	0,564300662
TAF7	0,977983381	0,284212813
OR2H2	0,978479365	0,026874722
PRRC1	0,978562997	0,191279998
OR5J2	0,978685626	0,074248592
ITGA10	0,978700297	0,022517935
TTC1	0,979413995	0,148922173
OSBPL3	0,979566912	0,000160611
DCXR	0,979790612	0,162151655
PBX4	0,979855792	0,740931736
EGF	0,980052709	0,821109254
SERPINF1	0,980513332	0,315765805
BSCL2	0,981312169	0,206799769
ZNF518B	0,981589811	0,023219838
IFRD2	0,981669033	0,208253142
PRKAB1	0,981826475	0,065370836
LRRC42	0,982398951	0,102843197
hCG_1776018	0,982987405	0,015758866
SFRS7	0,98299538	0,225277469
SIAT7B	0,984187924	0,039845624
C8orf33	0,984199807	0,039286438
PNPLA2	0,984209405	0,106889832
C21orf67	0,984386577	0,262374282
LOC649371	0,984882069	0,083086989
TRIM40	0,984925006	0,171394969
TCF12	0,98542187	0,048127427
WDR4	0,985616605	0,005571047
C10orf64	0,985712023	0,051966938
PRKCN	0,986003951	0,087384729
C17orf66	0,986564647	0,869586443
hCG_2045614	0,986765312	0,050149761
LOC729312	0,987146487	0,182991525
SLC2A6	0,987523111	0,731680699
LDHD	0,987537857	0,22785951
FAM46C	0,988649065	0,843102462
ARHGEF10	0,989235352	0,009134004
CCDC105	0,989347411	0,075668827
LOC730875	0,98939703	0,869672212
VKORC1L1	0,989762319	0,377097745
BARHL2	0,990857916	0,044295522
SLC22A5	0,991428442	0,072898581
C16orf63	0,99155916	0,163787766
WDR44	0,991578483	0,020161463
GDF15	0,991676531	0,043309265
LOC731626	0,992678806	0,087551951

PKNOX2	0,993029847	0,089971477
AHCY	0,99367263	0,003554735
MAPKAPK2	0,993747068	0,208313122
STARD3	0,99413469	0,009978372
LGMN	0,994288623	0,373306408
LOC642707	0,994692542	0,194573365
LOC648494	0,994803542	0,300145196
UCHL3	0,99482852	0,488817122
LOC389895	0,996641826	0,358787858
LOC284912	0,997050213	0,016368465
RFC1	0,99749831	0,011542592
GFPT1	0,997659889	0,125726693
FLJ30473	0,997922992	0,653935259
RAB14	0,998057713	0,126402773
NSMCE2	0,998900317	0,044735591
NEIL2	0,998929982	0,263759914
MVD	0,998935459	0,103895333
LOC644541	0,999397309	0,517486108
SDHD	0,999577899	0,121102807
PCMT1	0,999595523	0,146237369
SARS2	0,999684301	0,265513461
TMEM39A	0,999781425	0,000254979
LOC338758	1,000011248	0,13710392
TWISTNB	1,00039635	0,020678624
PLCXD3	1,000504548	0,210311622
MPN2	1,000937031	0,165866712
LOC729036	1,000995964	0,05916968
CEACAM7	1,001143505	0,013079059
SNRK	1,001473931	0,245572631
TMEM59L	1,001769705	0,72301562
LOC728679	1,002298011	0,03472056
PELI3	1,002305961	0,210110028
PBX3	1,002535717	0,110639742
CHMP2A	1,003419989	0,023773729
TSPY1	1,003547059	0,114254239
LILRA2	1,003724531	0,29205462
MRGX3	1,004497807	0,226104683
ACADVL	1,004713033	0,002090179
CCDC11	1,004976321	0,209248378
ZNF835	1,005624093	0,08364586
MC4R	1,006139876	0,253782164
LOC651337	1,006276445	0,18484964
CCKAR	1,006670447	0,184641184
SLC2A12	1,006879225	0,085240649
GPR1	1,0070607	0,182609552

ISCA1L	1,007663423	0,21558073
TIGIT	1,007677719	0,022282277
LOC728756	1,007879135	0,117057729
CDA	1,00825198	0,18118849
PGPEP1	1,008302449	0,108619197
C7orf26	1,008395856	0,078094171
KIAA0802	1,008403567	0,123659626
IGFBP7	1,008506159	0,002103027
AP1M1	1,008599744	0,04332612
ALKBH6	1,008719408	0,096424887
UQCRC2	1,008914296	0,065736942
AANAT	1,009044214	0,458217968
LOC651845	1,0095561	0,051459723
REG3G	1,009561064	0,521018087
KCNE1	1,009685766	0,195377314
KGFLP1	1,010136463	0,007532496
OR2T34	1,010767174	0,180779766
RGS2	1,010853795	0,174647666
TMEM74	1,010906458	0,073586569
SULF1	1,010981043	0,320845886
C4orf36	1,01099042	0,010666203
LOC729488	1,011016189	0,001766757
GREM1	1,011032943	0,071365081
DACH2	1,011434531	0,104663909
hCG_1642354	1,011459464	0,061687366
MAN1C1	1,011505248	0,10836042
C1orf86	1,012032871	0,054223486
TUSC2	1,012763069	0,043558662
APG4A	1,012780653	0,024403436
LTB4R	1,012975538	0,032776496
DKFZP564C196	1,014371628	0,01461117
LOC730998	1,014635309	0,068411612
LOC730202	1,014823194	0,499283104
OGG1	1,014913604	0,154477082
KLC2L	1,015750082	0,111951051
ZC3H8	1,015764734	0,030302513
LOC652591	1,016003557	0,034030352
C20orf106	1,018321128	0,195085753
CAD	1,018387221	0,130391018
CSDA	1,018465314	0,012535475
C10orf96	1,018595022	0,085153139
ESPNP	1,018854927	0,698798514
ANAPC5	1,018875952	0,252853438
C2	1,018889931	0,055753303
MYH9	1,019680675	0,523386237

ZNF300	1,019773251	0,325361795
CLN2	1,020008293	0,08358142
NEXN	1,020704148	0,049650377
OR10AG1	1,021209018	0,082142752
DPF1	1,021284704	0,077218761
C9orf47	1,021911656	0,012895089
USP51	1,021996186	0,124812229
SYNC1	1,023186368	0,152190597
UBAP2L	1,023574215	0,073600159
DUB3	1,023671791	0,010443201
MAP7	1,023890651	0,358417728
BCAM	1,025852431	0,143438275
WDR23	1,028189863	0,37203415
C4orf8	1,028353922	0,217124454
LOC728308	1,028535597	0,50740876
PPAP2A	1,029011853	0,119283675
ZNF313	1,029434964	0,076931821
HIST1H2BO	1,030088737	0,509755273
APLF	1,030137055	0,036780014
C5orf47	1,030791925	0,115579899
ZNF167	1,030919124	0,012920905
HDAC7	1,031054205	0,120860278
AZGP1	1,031156804	0,156442412
TRAF3IP1	1,031245907	0,103517797
LOC727759	1,031403119	0,255138457
C6orf174	1,031584165	0,026862023
OR8K3	1,03185423	0,41515117
DDX12	1,033145149	0,040591768
NR3C2	1,033539289	0,446020838
CMKOR1	1,033723189	0,179157995
LOC732447	1,03385243	0,235345902
C2CD3	1,035020395	0,452039822
MYL7	1,03516757	0,066742483
FLJ40039	1,035303159	0,070317365
SLC22A16	1,035616999	0,094630622
TMEM178	1,035984624	0,099777053
CTTNBP2	1,03648035	0,111296314
C5orf4	1,036610632	0,089596001
LOC731667	1,036711843	0,008848941
MLLT3	1,036837235	0,971796164
SCRN1	1,037323742	0,142609488
LOC388923	1,037502595	0,040510628
SCRIB	1,038292123	0,216678733
CYLC2	1,038296742	0,204333174
ZNF274	1,039114363	0,504987194

LOC732091	1,039466616	0,050603168
POLK	1,039485404	0,032800607
NSD1	1,039827391	0,039402452
VAMP2	1,040032762	0,123486824
CDS1	1,04156844	0,110626698
COX6A2	1,042244234	0,05602349
LOC401074	1,042558214	0,22440584
LOC645638	1,043145489	0,279992097
OR5B12	1,043710575	0,034939556
BLOC1S2	1,04400705	0,377129808
UBE2Q	1,044641048	0,035353989
HDAC8	1,045268675	0,605814324
CSK	1,045587184	0,080247236
ABCC12	1,047099241	0,03065873
PTPRCAP	1,047387148	0,024982893
MFAP2	1,047771055	0,178679664
MGC27121	1,04786463	NA
XRCC3	1,047910889	0,294793102
LOC731400	1,047962492	0,108574383
KAAG1	1,048207265	0,001850543
C2orf25	1,048838054	0,085135012
LOC283551	1,049502646	0,072196405
LOC729618	1,049578356	0,189423131
SEC62	1,050469429	0,121414844
LOC730160	1,051018505	0,198076456
LOC728682	1,051164128	0,028027159
LOC728365	1,051445984	0,058981495
LOC651672	1,051586803	0,071635088
MGC14793	1,051648954	0,232103128
XKR7	1,052859881	0,061269327
CHMP7	1,054033263	0,104150877
JMJD8	1,054207428	0,015434676
LOC730719	1,054711715	0,118058558
LOC161823	1,054712787	0,013954534
LOC728012	1,055338484	0,161793666
C11orf71	1,055643832	0,103980933
NEDD9	1,056140679	0,073670133
SPATS1	1,056431802	0,025397706
AMD1	1,057058134	0,01140633
STX10	1,057525126	0,047379382
MCM6	1,057804105	0,0737969
GPI	1,058206221	0,107917149
LOC730792	1,058360075	0,01930413
FAM57B	1,058457322	0,213543191
LOC643936	1,058713307	0,056823089

LOC645420	1,059200051	0,065661894
AFAP1L1	1,059230945	0,094608871
LOC729204	1,059278006	0,027101595
LOC650749	1,059321469	0,072955882
LOC643086	1,059339685	0,029339138
B3GNT9	1,05940682	0,022766167
PIGH	1,059552857	0,038373725
GBP1	1,059615107	0,226501849
SPRR4	1,059738306	0,02774661
KIAA0590	1,059805283	0,044469964
AARSL	1,0603369	0,042332052
MPEG1	1,061190731	0,259406723
DKFZP586B1621	1,061538203	0,186019761
POU5F1	1,061816927	0,290530567
ZNF121	1,062718735	0,376567633
DMRT2	1,063202696	0,081002975
LOC728351	1,06362878	0,01586473
AMELX	1,064284852	0,188314179
TOB2	1,06468954	0,328598567
CHI3L2	1,065893598	0,04222115
PSARL	1,065894017	0,024814821
MSRB	1,066576721	0,105555407
FLJ42986	1,06732738	0,04096571
APOBEC4	1,067890896	0,306226934
LGTN	1,068388497	0,157652097
RRM2	1,068862817	0,061237924
PCDHB14	1,069620794	0,56519545
GPT2	1,069648456	0,080474563
CHRND	1,069727588	0,148028712
MMGT1	1,069826504	0,000597286
DMRTC2	1,069979567	0,021463701
HOXB6	1,070268794	0,091162577
SLC28A3	1,070806968	0,126860293
CCDC75	1,071200125	0,423829
C1orf122	1,071668508	0,154442397
LOC729424	1,072221195	0,021753639
CCR9	1,072287247	0,183323625
KSP37	1,072456203	0,249556199
ALPP	1,072807403	0,029109812
LOC728102	1,073396898	0,123606914
STH	1,073691587	0,108826075
MMRN1	1,0741979	0,044372072
TPD52L1	1,075171413	0,85229458
CHAF1B	1,07522993	0,200421182
LYAR	1,076247443	0,099487267

SPINK1	1,076904177	0,066330883
LOC729121	1,076934035	0,570590616
LYPD4	1,078785966	0,73505484
FASLG	1,079303456	0,115161476
SSR3	1,079426079	0,107112879
OR52W1	1,079480284	0,220154725
LOC283999	1,079526074	0,107874649
RRAS	1,079638756	0,016305796
LOC285735	1,079892245	0,097775513
DNASE1L2	1,079949041	0,389555166
SLC23A1	1,080117922	0,002494839
CT47,13	1,080143032	0,046826787
GPR77	1,080599133	0,072048026
TIMM44	1,080655252	0,123842996
NEU2	1,080838955	0,392277699
LOC729292	1,080868421	0,00952496
STAC	1,081429564	0,215679721
DUSP8	1,081447645	0,004995834
LOC730094	1,081712353	0,231532202
DNAJB12	1,08252202	0,763788236
PIR	1,084131976	0,169320571
STC1	1,08491122	0,148482881
LOC644756	1,085014051	0,381883177
PCDH7	1,085048458	0,935796167
KCNAB2	1,085914369	0,006265357
STK31	1,086655763	0,073605677
ACCN5	1,086786043	0,079648162
EMX1	1,08693424	0,123181284
LOC728510	1,088030524	0,017947033
PCBD	1,088226355	0,316360395
RPL10	1,088904041	0,797385926
FAM3A	1,089266176	0,039420215
C12orf41	1,090464329	0,012481122
MGC33894	1,090817498	0,333125756
IL17F	1,091148285	0,011652652
EFG2	1,091489278	0,025786056
LOC644844	1,092207584	0,13771814
OR1G1	1,092463672	0,191487724
DCP1B	1,092469386	0,250496661
NDE1	1,093536391	0,168767377
TAS2R49	1,094034736	0,195204192
UBE3A	1,094110247	0,052321052
ATP4B	1,094223967	0,160301116
OLIG3	1,094251495	0,137012373
LOC730677	1,094311221	0,201998529

IGFBP6	1,094509084	0,053940673
P2RX2	1,094860075	0,135862232
FKTN	1,094982828	0,022956472
LOC728470	1,095324396	0,038059875
SNPH	1,095414112	0,020334055
C14orf138	1,095483255	0,09309284
LOC442204	1,095834035	0,118417012
C11orf61	1,097018411	0,366099419
RTN4RL1	1,097119167	0,340477646
C3orf48	1,097831141	0,088082833
SULT1A2	1,098154628	0,091641142
LOC440084	1,098357951	0,081732267
CAMK4	1,098466297	0,004823852
KBTBD4	1,098647159	0,087130436
EML3	1,098653786	0,00074053
PCTP	1,099274795	0,132822271
TNKS2	1,100028005	0,292494069
LOC732078	1,100227697	0,038328837
LOC646177	1,100531692	0,187054974
TSTA3	1,101447837	0,133986762
HIGD2A	1,101453165	0,55566522
LOC642366	1,101660923	0,23361746
SOX11	1,102100055	0,12392041
DECR1	1,103425805	0,082341472
DOK4	1,103662316	0,45678696
LOC642620	1,103694505	0,069712825
GFER	1,103909335	0,357271516
ADAM28	1,104873515	0,045847777
BHC80	1,104996634	0,144180708
LOC731023	1,105090359	0,135372469
FLJ39061	1,105340254	0,141627755
EYA1	1,105373554	0,121919343
PLAC9	1,105895398	0,167634863
BANF2	1,106126012	0,159223193
PROCR	1,107260445	0,096751732
FLJ36031	1,107509097	0,086179127
LOC643008	1,107585019	0,095821897
C3orf34	1,107977794	0,287995984
KY	1,108437519	0,042598684
LOC729621	1,108628784	0,069417875
VSTM2A	1,108694599	0,029542871
FLJ44815	1,109109398	0,194265407
EMCN	1,109519502	0,067340065
SAPS2	1,110140744	0,013415662
KCNQ5	1,110413782	0,007912263

WDR21A	1,110477268	0,008680993
FLJ10948	1,111133195	0,02599839
LOC652444	1,112085024	0,29767137
SHH	1,112106673	0,226015491
CSPG2	1,112879643	0,074665981
DDR2	1,113402481	0,117762456
ZNF503	1,113831944	0,713326401
MAN1A2	1,114223802	0,190364437
TMEM39B	1,114560238	0,364858158
COP1	1,115479642	0,038525607
ERAL1	1,115784778	0,049511988
DST	1,115901313	0,029715769
LOC730088	1,116632848	0,003160098
SGCD	1,116642571	0,163433956
CFHR3	1,11697651	0,041769738
ADAM32	1,117725636	0,21428738
HIAT1	1,117839132	0,027927472
LOC653885	1,118921987	0,051141035
BCKDHA	1,119242229	0,028661111
C1orf31	1,119504167	0,078367269
hCG_2040376	1,119944902	0,103803641
SNAI1	1,120218525	0,183190863
CEP152	1,121159471	0,074992238
TOMM40	1,121252273	0,845078788
RNF24	1,121753328	0,035966299
SEC31A	1,121981948	0,044788972
C1orf2	1,123241528	0,070376856
GNRHR	1,123831869	0,064565058
TRIP6	1,124716818	0,004111664
LOC729047	1,124730745	0,129393617
EGFL10	1,124742683	0,082825835
FAM26B	1,124977237	0,365965947
MN1	1,125068053	0,87056401
C7orf54	1,125986523	0,516315898
FAM127B	1,126210356	0,066653961
MAGEA10	1,126645693	0,021472364
KIR3DX1	1,127083557	0,165073824
LOC727793	1,127749634	0,102189068
LOC729766	1,127919331	0,418133145
CRYBA1	1,1281275	0,354696996
RTKN2	1,128175484	0,045660709
FAM98C	1,128187842	0,233486884
LHX8	1,128199481	0,00824007
PRPH	1,128754766	0,001768974
OSBPL7	1,128925107	0,100038034

LOC729743	1,129315766	0,103264105
C20orf26	1,129326723	0,029186892
CUL2	1,129568772	0,085851362
RAB38	1,129847873	0,101396457
C12orf43	1,130418596	0,06971012
PPFIBP1	1,130487229	0,459146293
C7orf36	1,131132286	0,419516905
UNK	1,132669133	0,176512942
C10orf137	1,132741228	0,5
C19orf43	1,132822851	0,100770016
PRRX1	1,133417514	0,046625624
LOC728414	1,133620343	0,676799812
OR13C3	1,134069593	0,15791456
MBTPS2	1,134473138	0,022879167
KIAA0774	1,1348624	0,140976375
ITPR3	1,13504016	0,268095329
LOC389024	1,135915333	0,383845185
CALCB	1,135916516	0,068391348
LOC727869	1,137295869	0,482295612
CATSPER2	1,137406693	0,09912709
MARCH7	1,137654903	0,13079114
USP6	1,137705857	0,194101859
MGC57341	1,138647109	0,360219857
RTP3	1,138694114	0,256284704
LOC643951	1,138754336	0,096532361
LOC284184	1,139311005	0,469125225
LDLRAP1	1,139920017	0,043572959
ACAA1	1,140056123	0,009452372
LOC440587	1,140406476	0,030799067
LOC651741	1,140476134	0,193812179
TTF1	1,140649561	0,707200777
C9orf100	1,140843826	0,036933712
CLLU1OS	1,143202826	0,014129123
HDLBP	1,143268371	0,148504318
PTPN18	1,143276896	0,133194918
C1orf170	1,143316527	0,217696003
MORC	1,143558317	0,636308451
MRPL46	1,14375457	0,139959736
CCT4	1,144227213	0,106411187
LOC440350	1,144378851	0,583517276
ZNF491	1,14441304	0,096876687
IL26	1,144634633	0,000100676
NPAS4	1,144960349	0,106588935
EPB41L4B	1,146457584	0,082897087
HNMT	1,146601745	0,023207858

C14orf166B	1,147025873	0,146966088
NUP35	1,147146537	0,274755022
CSF2RA	1,147247745	0,280722156
BRWD3	1,147839059	0,237176957
PCDHB11	1,147908643	0,354789571
GSDMD	1,147991067	0,392758173
LOC284395	1,148349186	0,024486197
CCDC142	1,149187549	0,100925341
POGK	1,14952547	0,09017674
RFX3	1,149611231	0,036483906
p66alpha	1,150515888	0,074373879
EP400	1,151712282	0,041383497
RABL5	1,152395115	0,00936427
LOC644684	1,152575168	0,287944844
MAP2K4	1,1537641	0,094443492
LSMD1	1,154419865	0,658665964
LTV1	1,154552436	0,276304557
LOC729800	1,154821083	0,05176842
LOC729921	1,155337741	0,000403863
CRHBP	1,156293201	0,589314378
LOC145783	1,156680532	0,223690142
LOC728674	1,157567815	0,04385043
HSRTSBETA	1,157692974	0,156026961
HHLA1	1,157925905	0,25753754
NRM	1,157953386	0,013275466
TAGAP	1,158182629	0,010395792
ABI3BP	1,158609858	0,014055936
FAM21D	1,158612088	0,198797889
ASPM	1,158667449	0,534367324
LOC55908	1,158957234	0,014554371
TFCP2	1,160028577	0,031008406
RBBP7	1,160608971	0,186046821
KIAA0317	1,160797699	0,027537962
FUSIP1	1,162389648	0,092776887
TMEM139	1,16267735	0,138442915
ZNF222	1,163159122	0,17134072
MMEL2	1,163410523	0,142043232
SNX9	1,163771733	0,089642288
ATF6	1,164095165	0,300778058
WDR6	1,164590513	0,121953083
LOC728834	1,164607349	0,220103394
NUP98	1,164859992	0,063548997
TAS2R7	1,165054782	0,015015065
DNMT3B	1,166997654	0,119682959
C11orf79	1,167713536	0,098719447

CDH7	1,168341262	0,000164446
LOC731957	1,168880303	0,032150086
EPS8L1	1,169228477	0,058785854
FLJ34515	1,169411418	0,225526085
IL20	1,169513779	0,446890892
LOC728721	1,170382537	0,768452163
PSMG3	1,17038469	0,016384102
IDH3B	1,170768108	0,047757184
C10orf51	1,171002638	0,004703378
SPG4	1,17176653	0,115151642
ARHGEF1	1,171885879	0,098342333
CRIM2	1,172144098	0,004308638
ARHGEF11	1,17232687	0,067119041
IFT80	1,172352948	0,130444149
MGC35130	1,173100683	0,033450424
LOC51257	1,173150896	0,13986955
C6orf70	1,173156176	0,084837035
UGT2A3	1,173916305	0,099549764
SEMA4C	1,174036093	0,097414583
PCDH10	1,174196747	0,092223795
ZMYM3	1,175070338	0,059223364
VN1R2	1,175319481	0,040196028
BTNL9	1,175321877	0,413068151
C9orf140	1,175681592	0,08552305
SCN3B	1,176073523	0,396963398
HCRTR2	1,176132326	0,020762106
MUSK	1,176891886	0,185479591
SENP3	1,177143275	0,076930677
KIAA1194	1,177364823	0,017962334
SMCHD1	1,177640784	0,045924857
TFDP1	1,177905301	0,027425866
ITSN1	1,177927728	0,03320284
SYNJ1	1,178167313	0,062132919
CSNK2B	1,179217425	0,026078737
NAGPA	1,179520393	0,0485636
CELSR1	1,179692675	0,151998088
SULT1C1	1,179767324	0,01645851
ZNF364	1,180099064	0,525248655
RP11-520H14,1	1,180120529	0,156324637
VN1R1	1,180234703	0,065726446
SIGLEC9	1,181542772	0,039315272
LOC652726	1,181660662	0,041851213
TAS2R50	1,182013556	0,125038232
SPRR2F	1,182997195	0,05883788
FABP1	1,184287548	0,025501649

TXNDC16	1,184488007	0,168275004
ECOP	1,184609217	0,501946679
RTN1	1,184803614	0,039806107
MAFK	1,184935989	0,165516262
HISPPD1	1,185568289	0,053574506
SIAH3	1,186268366	0,028721755
MT1CP	1,186426992	0,098794423
ZBTB10	1,187393242	0,157295668
LYSMD3	1,18954958	0,282480122
OR5P3	1,189912452	0,063186678
KIAA1826	1,189967661	0,06015187
ASTE1	1,191128825	0,120981405
SFMBT1	1,191393088	0,335653515
PKMYT1	1,1924058	0,25636667
TRIM45	1,193243973	0,058887488
APCS	1,193415252	0,20122021
LOC642461	1,193830453	0,319216715
DLAD	1,194458568	0,051261753
C17orf35	1,195242905	0,000675656
FTHFD	1,195441038	0,097644169
DHX34	1,195566111	0,12697432
LOC652799	1,196941851	0,109120637
COLEC11	1,197320736	0,129132194
PEPP-2	1,197400727	0,048202823
BLCAP	1,197805073	0,144193756
NIPA	1,197858649	0,683615497
MON2	1,198497742	0,181002264
TGIF2	1,199428083	0,129034965
PCDHGA1	1,199719792	0,118305816
BIRC5	1,20147073	0,033200873
NFYC	1,201564552	0,04181984
TNFRSF1B	1,201678833	0,208815216
TMEM17	1,202436915	0,062662633
DSCR6	1,202960709	0,02791031
LPP	1,203055286	0,09275037
PER1	1,203699299	0,077466937
FOXK2	1,204467536	0,009048237
CABLES1	1,205090791	0,02764368
VPS29	1,205123411	0,616459772
MAP2K2	1,205132811	0,04608747
PDLIM2	1,205159501	0,279882567
PCDP1	1,205570194	0,006563724
FSCN2	1,205788701	0,073283448
FAM22F	1,205893707	0,01097803
MITF	1,206455916	0,069687843

ISL2	1,20781936	0,028058314
MTHFD1	1,20796442	0,178260491
EFCAB4A	1,208060904	0,112560058
DLG4	1,208168347	0,031002585
OCM	1,208261722	0,02053808
LOC729172	1,208815131	0,156771163
LOC729658	1,208831871	0,08718431
PSPH	1,208983731	0,149488552
KCND3	1,209977881	0,006298831
PRRG3	1,210793493	0,007373949
DNA2L	1,211005408	0,019150343
IL11	1,212204182	0,250004358
KIAA0830	1,212618745	0,110382219
CPD	1,213056811	0,634740312
LOC284952	1,213388218	0,516210313
FTSJ2	1,213414658	0,045412722
UGCGL2	1,213425957	0,024373116
C6orf102	1,213693445	0,098815565
HCRTR1	1,214124853	0,000966422
KIAA0962	1,215318877	0,168854919
FAM159A	1,215432439	0,057751278
DGAT1	1,216031656	0,151908677
PIP3AP	1,216447875	0,088074833
SPACA1	1,216879494	0,164414437
MBIP	1,217202809	0,043213161
HOXB3	1,217407853	0,061367385
SPOCK	1,217456703	0,03544729
LOC283985	1,217569823	0,049504722
BAK1	1,217735731	0,169937641
STT3B	1,218074449	0,056096726
PEX11A	1,218122751	0,128782045
LIMS2	1,218568456	0,325098065
TMEM189	1,218877991	0,089107276
SILV	1,219630285	0,214282487
PTCH2	1,219952742	0,853708349
S100PBP	1,220014296	0,201267607
PAX2	1,221395864	0,069159437
NR2F1	1,221606914	0,202295892
C7orf29	1,222739216	0,353589545
HK2	1,223519971	0,541958941
LOC340096	1,224047965	0,671825037
ETV5	1,224120497	0,026366386
LOC732226	1,224361852	0,030833034
WDR41	1,224462597	0,050802333
CRELD1	1,224837099	0,907526274

NCE2	1,225216461	0,080094959
MGC29643	1,225386655	0,072054256
ACTG1	1,225770383	0,310531678
CAMK1D	1,227055636	0,083111267
BIRC3	1,227528946	0,467599596
TRIM2	1,228331031	0,175170404
PPIG	1,228452276	0,009399235
KIAA1632	1,228728275	0,036210877
CDC45L	1,230238961	0,076774539
ARF5	1,230353616	0,163486152
CLDN17	1,23040778	0,260269418
PACSIN3	1,231469345	0,288429818
TSPAN15	1,231578556	0,066223831
LOC401180	1,232499282	0,273045998
hCG_2032978	1,232516941	0,147661586
AMIGO1	1,23272933	0,349005032
LOC731332	1,232890721	0,051108374
ANKRD31	1,234431194	0,224666323
CLDN16	1,236699453	0,14755174
C10orf99	1,236932986	0,079793797
LOC641798	1,236970109	0,016265033
LOC728120	1,237745528	0,226716041
GRK5	1,238381843	0,015175777
CCDC90B	1,238645125	0,021322899
CMPK2	1,238759635	0,128930166
LOC642669	1,239636606	0,038840914
FUT2	1,241125025	0,050872188
MEOX2	1,241437134	0,004921139
UBE2M	1,24146936	0,000523108
CILP	1,241749448	0,499580609
C1QTNF9	1,241874723	0,006104211
SEC22A	1,242202143	0,047080805
FGFR4	1,243989703	0,087749416
EMR3	1,244843878	0,011397563
FBXO32	1,246792448	0,107797458
PTPRM	1,247197035	0,06681703
LOC648517	1,248195549	0,084794626
PLA2G7	1,248457894	0,005921633
TBC1D22B	1,248553332	0,000693647
DIO3	1,250007955	0,069901779
LOC440313	1,250272207	0,115763551
DKK4	1,250593341	0,070894884
CLYBL	1,251462073	0,364878596
OR6A2	1,251645072	0,021141118
RNASEH1	1,251773423	0,060607108

STAT2	1,251835594	0,026095746
DNAH17	1,251908236	0,044008159
LOC731523	1,252106077	0,124344599
PAICS	1,252512538	0,281667552
ILF3	1,253088762	0,036806893
LOC729596	1,25331728	0,062533945
SOX7	1,253582702	0,104724288
HRK	1,253662962	0,007358306
TNK1	1,254549949	0,04925958
MOGAT1	1,254606984	0,094033973
DIA1	1,254839946	0,05531054
UBL4B	1,255879013	0,45676663
XKR5	1,256907117	0,089464828
PMP2	1,25713545	0,101118989
CCDC108	1,257309312	0,287039878
RBBP9	1,257986328	0,001706742
ZFP161	1,258805761	0,153761433
MFRP	1,259353141	0,140937034
MGC16121	1,25937317	0,22433242
CCL25	1,259724695	0,00145942
GPR114	1,260063949	0,089775621
LRRC34	1,260877923	0,281264934
RGS20	1,26101119	0,052341619
PEX13	1,262734187	0,035985437
HRASLS2	1,262792168	0,013661398
LCE3C	1,263775125	0,096085312
SDF2L1	1,264590541	0,069402461
TOMM22	1,265155438	0,134149469
TRIM11	1,265199415	0,075193786
LMCD1	1,2659711	0,024335919
RRAS2	1,266024056	0,384232806
PRKG1	1,267086295	0,148297027
FAM135B	1,267335208	0,027832848
FLJ40125	1,267407122	0,127998295
LOC143678	1,267947384	0,157779138
CALD1	1,268198247	0,417158364
H6PD	1,268313469	0,213441805
GABRB3	1,268902478	0,095404437
UCN2	1,269432846	0,050100031
LOC643037	1,270186453	0,012292273
C14orf1	1,271228173	0,015260532
LOC729959	1,271956145	0,280821957
ADCY7	1,272069142	0,04525049
LY6G6D	1,273108033	0,260800969
NUTF2	1,274616991	0,001393829

DPY19L4	1,274681064	0,195677949
SLC26A1	1,276033051	0,067317582
PGM2L1	1,276775913	0,298154241
LOC197350	1,27769209	0,029962625
CSRNP2	1,277956306	0,276971011
FRS2	1,278081679	0,112604452
LOC650901	1,278836527	0,175080903
YIF1A	1,278854354	0,186310328
PSMB3	1,279292093	0,054931316
LOC130678	1,279540626	0,079117328
C12orf56	1,279751265	0,037677153
SNX10	1,279773934	0,044872064
GCKR	1,279792707	0,195803221
BMS1P5	1,280604067	0,07713423
UPK1B	1,281000688	0,449536535
LOC730864	1,28147326	0,098433752
OR1L6	1,282010479	0,205674873
NOVA2	1,282303432	0,054434497
LOC730124	1,282871967	0,534353468
ENDOG	1,284916886	0,144019038
ARHGEF18	1,28528644	0,110701188
LOC652886	1,28530034	0,009390554
CCT6A	1,287796517	0,016044117
ZNF519	1,288122724	0,101873485
ARAF1	1,288682142	0,17441563
TCEB1P3	1,289341489	0,281947477
LOC730754	1,289495821	0,060773501
SPATA18	1,289679823	0,105856076
LOC642843	1,289742486	NA
LOC644982	1,290342425	0,512269597
TPM3	1,291399825	0,22753832
MYO1E	1,291463488	0,057354353
OAZ2	1,292433798	0,18170319
CCDC146	1,29287059	0,022729507
OR52J3	1,293101415	0,013251692
GABRG1	1,29325479	0,156295303
TTK	1,293437686	0,058673058
KRTAP20-1	1,293746698	0,01742494
PTPRN	1,294873631	0,029944488
ANKRD28	1,294939011	0,184877208
LRDD	1,294971539	0,062804635
LIN37	1,295635615	0,59023769
HIPK3	1,29668701	0,208934483
LOC642656	1,297187614	0,018933049
VWA1	1,297328991	0,307320763

LOC729620	1,298030323	0,194040526
PTPN7	1,298076591	0,139799594
ZNF213	1,298833554	0,035784844
MT1X	1,299819582	0,04627585
ING3	1,300383725	0,01367615
LOC647278	1,300855216	0,049879398
POU3F3	1,301640631	0,064527354
VNN3	1,303008098	0,060531033
L3MBTL2	1,303038123	0,01907063
SLC19A2	1,303187839	0,062624604
KIAA0052	1,303422922	0,005795455
ARMCX6	1,303615606	0,245582117
DISC1	1,303995793	0,095385052
LOC727942	1,305012555	0,002352887
C2orf78	1,305445843	0,120519796
RNF43	1,308343607	0,210997653
CDC20	1,30911048	0,157572961
LOC439985	1,309486031	0,098208879
SPINK4	1,309701404	0,070316617
SLC35A1	1,310301187	0,066503333
KLHL21	1,310848135	0,046900037
TRAPPC9	1,311023481	0,077171265
LOC132707	1,31132019	0,046963909
HHEX	1,313569545	0,121216564
TIMM17B	1,313671527	0,091654865
FAM83G	1,313969216	0,220109904
ZNF558	1,314644294	0,070244538
TEX261	1,315015853	0,083028885
ANKS1B	1,316376864	0,03096283
TRIM32	1,317409598	0,635508756
TIF1	1,318284794	0,304742456
ILT7	1,318490542	0,085865036
C11orf75	1,318788074	0,064085563
DPY19L1	1,320961254	0,552630673
RASAL1	1,320982898	0,129451573
SEC14L1	1,322682826	0,132863422
SUCLA2	1,323540715	0,08847919
ITK	1,32412625	0,172334621
SCARF2	1,324459933	0,113724632
RHOC	1,326329586	0,13073219
TMEM170A	1,32663889	0,010952049
FPGS	1,326800463	0,017194956
NKIRAS2	1,326972653	0,362812862
SLC34A1	1,32710017	0,549441465
DNMT1	1,328306807	0,061057284

C11orf66	1,328347044	0,060731054
MRPL42	1,328527537	0,782236726
LOC729111	1,328594748	0,052269692
BICD1	1,329474032	0,024692116
CD3D	1,330048006	0,087613052
C12orf10	1,330092644	0,014852629
LYNX1	1,331884622	0,118040804
FAM117B	1,332883417	0,178296148
OR7A17	1,33359988	0,02303004
tcag7,893	1,333799575	0,255566236
BMP2K	1,334369418	0,119285001
FABP7	1,335873759	0,020533423
GIMAP1	1,335875984	0,048089228
C18orf10	1,336030355	0,110660589
CLEC1A	1,336053268	0,305054243
C12orf26	1,336812982	0,036274439
UNC93B1	1,337791966	0,406606093
IQCB1	1,337982362	0,126568597
LOC728086	1,338930598	0,080144951
TRAPPC2	1,339152646	0,24294509
LOC648934	1,339497435	0,404575312
FLJ16423	1,339717992	0,0618131
SLC27A5	1,340098157	0,185946675
CWF19L1	1,34052606	0,081605558
VEGFC	1,340671674	0,401761924
PNPLA1	1,341510929	0,085515603
MSRA	1,341647365	0,137410439
CCR7	1,342818257	0,020605855
ZNF339	1,343538327	0,144747783
RAB37	1,343639542	0,125115827
LOC652259	1,344807562	0,643198362
LOC440145	1,344895134	0,002437268
PPP1R11	1,345013148	0,013051659
MYO18B	1,345110104	0,157447777
TMEM66	1,346358415	0,142418167
LOC731779	1,346851011	0,020242847
C1orf77	1,348715128	0,264455186
RHD	1,350227705	0,78076989
FLJ11171	1,350801322	0,038866498
LOC729022	1,351343233	0,077626105
C11orf73	1,351565314	0,156672595
SLC25A18	1,351924283	0,213386726
CCDC35	1,352502934	0,045544596
ARHGEF9	1,353201327	0,233536513
LOC727872	1,353500311	0,093397649

LOC643479	1,354008417	0,02402362
ZNF92	1,35445771	0,221286197
LOC728190	1,355067595	0,018764766
ACP6	1,355121909	0,479333757
TFCP2L1	1,356202619	0,080653968
FSCB	1,356836088	0,104058981
LOC730599	1,357491526	0,083307834
TAS2R60	1,357682252	0,289879699
LMNB2	1,357780321	0,595432496
PH-4	1,358477558	0,173334187
LOC729950	1,358763605	0,196001653
KLHDC2	1,358784308	0,032726958
C7orf20	1,361516389	0,268566029
CDC26	1,362366691	0,059868243
UBR1	1,36297143	0,321543181
hCG_1795091	1,363413217	0,16864445
OR6K3	1,364705552	0,045929777
OTOA	1,364971569	0,090186511
CENPA	1,365489592	0,177134802
MMP17	1,366482988	0,027031577
LOC646448	1,369032413	0,063705136
PCDHB9	1,369648926	0,091539119
EHBP1L1	1,36996283	0,037018251
C1orf150	1,370756901	0,21513285
C1orf111	1,371208871	0,059685973
LOC400831	1,372435664	0,21249866
PTDSS1	1,372600884	0,143553176
PCDH1	1,372789375	0,342201209
CHD3	1,373740679	0,195731068
MCFD2L	1,374198281	0,808082983
PSG3	1,375036329	0,255973321
RBM43	1,37631885	0,051513099
C8orf59	1,376596467	0,007077182
TEPP	1,377146706	0,0087547
VISA	1,377221093	0,133631826
LOC730909	1,377762405	0,469580711
MT1E	1,37790545	0,157365421
OLIG2	1,378340377	0,271562999
PHLDA1	1,37857616	0,068348103
LAF4	1,378692153	0,058095989
C18orf21	1,378724507	0,09196648
LOC652774	1,379602175	0,153121201
CCIN	1,379936193	0,091954612
TLK2	1,381451062	0,118357708
DPAGT1	1,383113683	0,121088784

LOC646960	1,385987674	0,189686718
FRMD6	1,386706029	0,026800754
FNDC3A	1,386882605	0,006186622
SYNPO	1,387082224	0,041961445
SLC16A1	1,387386042	0,054335967
FAM136A	1,387433829	0,687226856
DHRS4L1	1,387445073	0,08233863
C8orf49	1,388330128	0,089058056
IFI27L2	1,389698889	0,313152401
C10orf30	1,390196118	0,036383945
ZNF234	1,390503244	0,009581477
LOC117584	1,390722265	0,071097924
LOC648984	1,39180818	0,039061531
CXorf31	1,393429254	0,000435424
SNX20	1,393852071	0,311406658
ITGB5	1,393894976	0,021368598
MICALL1	1,394046888	0,160403808
PMS1	1,394724057	0,052996986
LOC731008	1,395193759	0,197737189
PPP1R1C	1,399174613	0,364720965
LOC643446	1,402115295	0,542824015
MYEOV2	1,402132414	0,256722065
PPHLN1	1,402174912	0,011686161
C8orf45	1,403130276	0,150669686
KIF5B	1,403139314	0,093113298
RPL23	1,404076948	0,038286432
ATF4	1,40773711	0,054520697
COL14A1	1,407867681	0,755248525
CCDC47	1,408305202	0,082254088
ARHE	1,408311842	0,090066232
DCUN1D4	1,409256849	0,131607335
HIST3H3	1,409415372	0,25900725
GPR172B	1,409572096	0,067830942
C20orf30	1,410441476	0,065143342
FLJ20244	1,411646824	0,144642666
MLL3	1,41169532	0,012507272
STK32B	1,41223783	0,032740504
GOSR2	1,412359632	0,410785635
LOC643339	1,413514564	0,003602979
LOC727972	1,414979707	0,103595328
TAS2R4	1,415575609	0,081056862
C5orf36	1,418596681	0,171781439
DKFZP434K046	1,419049252	0,195999749
C19orf56	1,421595206	0,089050315
EML4	1,422686237	0,034039847

SSH1	1,423055483	0,069599766
BSND	1,423737181	0,048743699
LOC653884	1,42586978	0,03362965
UNC5B	1,426074309	0,051087572
PHF6	1,426401226	0,124984144
EN2	1,427685409	0,009427949
RTN4R	1,428203053	0,493980639
ASK	1,428591629	0,170309239
LOC402679	1,429695548	0,121230518
PCDHB2	1,429716209	0,012169467
CDC25A	1,429752526	0,095918893
PKNOX1	1,430038861	0,013187213
DYRK4	1,430580885	0,054083882
OR4A16	1,430888065	0,038358034
EPRS	1,43218808	0,022881123
HPCAL4	1,43316079	0,155983388
PSMB7	1,435433244	0,254503146
ZNF25	1,435640901	0,146739375
WDR77	1,437718028	0,372912791
ARSI	1,437747364	0,381755127
NDUFAF1	1,439248207	0,009622726
LAD1	1,440407047	0,000466184
MS4A15	1,440653138	0,07728772
TAL2	1,440686052	0,076089717
LOC728137	1,442046817	0,491357796
OSBP	1,442536552	0,180270196
SRM	1,442907818	0,024742643
hCG_1794003	1,443062172	0,109190398
LOC377064	1,443250829	0,037749463
PTP4A2	1,444045263	0,281335717
EIF5B	1,444502544	0,100272345
KCND2	1,444809334	0,036064605
CASP1	1,445832297	0,040172159
SLC36A1	1,448096371	0,227401626
CCNF	1,448197687	0,241808036
FLJ26850	1,448376094	0,04923021
POLR2J	1,448486383	0,369539391
LOC652622	1,449209126	0,048259644
SCUBE2	1,449225998	0,338634713
MMP21	1,449431818	0,099574469
OTOP2	1,449958467	0,020070427
AMDHD2	1,450163337	0,085393285
LOC730008	1,450883349	0,089397275
PLEKHA5	1,452474473	0,033946077
LOC728020	1,453353095	0,108024464

LOC729492	1,453355474	0,005879852
COLEC10	1,453547655	0,170702629
CHST11	1,453813371	0,097142783
PRKAB2	1,454267739	0,058660557
DAGLB	1,455361466	0,145173562
ALG10B	1,456273712	0,010406358
UBE2H	1,456553324	0,163762004
IL1RAPL2	1,456600515	0,189318794
COCH	1,457009991	0,019485985
NCSTN	1,458668593	0,003827409
SAMD10	1,45897122	0,077971231
KHDC1	1,459635323	0,173778284
ATP6V1H	1,460114759	0,026661965
SSR1	1,462132488	0,034467287
SELB	1,462152034	0,072352725
LOC646070	1,462398505	0,107065544
ZC4H2	1,462545495	0,052398289
LOC728187	1,462658992	0,080881129
BAG1	1,463562845	0,169674923
KIAA0924	1,464827607	0,068249246
PUS1	1,465166769	0,043310495
CMTM4	1,466487987	0,05049856
STK38L	1,466687096	0,116976437
B3GALT3	1,467275466	0,376843962
TRIM7	1,467523328	0,141246902
PPP2CA	1,468429855	0,063178067
LOC729619	1,470574877	0,103462994
TNFAIP8L1	1,470587338	0,070876911
EZH2	1,4714582	0,019144503
LOC643475	1,471561104	0,162192213
STAG3L1	1,473073846	0,098974452
COX7A2L	1,473376881	0,081812092
LOC389369	1,473650911	0,240443541
OR10H3	1,473667398	0,011588685
BRCC3	1,473702348	0,060449357
SLC7A4	1,474839045	0,168699897
LOC441347	1,476355025	0,000633939
KCNH2	1,477219097	0,078135621
LOC728872	1,478029001	0,011051462
MPPE1	1,478483068	0,358535426
NHSL2	1,478853086	0,096918577
LOC729637	1,479372005	0,107039186
APAF1	1,480116041	0,104407254
RAB27B	1,480777753	0,23275471
CARD15	1,480816888	0,081361296

GPR7	1,482927534	0,029786886
BCL6B	1,486121194	0,007461479
YWHAQ	1,486621265	0,069812581
LOC732048	1,486752131	0,056032032
CLIC3	1,487975888	0,086786694
LOC731508	1,488281879	0,020350558
MARK3	1,488474169	0,056768272
STAT5B	1,489647792	0,12909948
SEMA3F	1,489764377	0,290948693
RHOBTB1	1,490056319	0,132521954
SLC13A4	1,490065239	0,154610433
CRAMP1L	1,490249343	0,010325963
LOC648217	1,490825385	0,038631491
C21orf126	1,491671321	0,010805942
ALDH9A1	1,492793506	0,026832265
PCDHA4	1,493683412	0,167782398
CABYR	1,494000555	0,061909768
LOC728111	1,494234616	0,019426957
OR6V1	1,495410393	0,071379781
EPLIN	1,495448653	0,065960886
TRHR	1,495769197	0,077336288
LMTK2	1,495801602	0,510932373
NIN	1,49585511	0,104423215
ARFGAP1	1,49689903	0,038079507
LOC642098	1,497356436	0,145792824
CST1	1,498236584	0,075491734
CRLF2	1,498615396	0,145909062
WARS2	1,501143799	0,11800207
FLI1	1,502449654	0,104586263
FAM157A	1,502528162	0,125282176
LOC401068	1,502555475	0,02710807
MYF6	1,502790447	0,030624198
WDR69	1,504005832	0,124837285
KRT71	1,504097989	0,074611417
HABP4	1,506082853	0,061590273
HPS6	1,507339065	0,022050974
PCTK1	1,508270943	0,185606041
TREX1	1,508847474	0,30342085
SLFN11	1,509086006	0,100677377
LOC642521	1,509222874	0,225343695
ELAC2	1,510131798	0,014443393
LOC728947	1,511055214	0,066698445
C19orf35	1,51264237	0,059866656
C2orf58	1,512686128	0,031865422
NDST2	1,513213682	0,058922221

LOC644524	1,515514357	0,000891604
SERPINA4	1,51645697	0,035285492
SH3TC1	1,516680637	0,263775336
SAPS3	1,516843798	0,003482393
RERG	1,516990331	0,012206864
LOC728046	1,517104067	0,078520214
SFRP5	1,517884391	0,245190971
CACNA1C	1,517990957	0,752899978
LOC728203	1,519613165	0,070159996
LOC728124	1,520509464	0,098289495
TMEM79	1,52148076	0,34469152
PABPC1L	1,522086326	0,029239463
LOC653303	1,523318023	0,005116943
GLTP	1,523656062	0,095894958
COL8A1	1,525096057	0,047064426
ARG1	1,526308355	0,186759069
TLL1	1,526803551	0,44328633
LOC730079	1,529506539	0,11685652
ITGB2	1,530127697	0,034603188
SLC16A9	1,530230471	0,303366983
RP11-321G1,2	1,531294049	0,165057154
Tenr	1,53256087	0,135907733
DUSP19	1,53274903	0,193290705
VRK3	1,534433453	0,011818076
SH2B1	1,536945983	0,097849048
LOC731015	1,537339935	0,047325572
CLDN23	1,538412986	0,078717951
OR7A5	1,539610798	0,132612858
LOC728366	1,540011656	0,029492041
MPRA	1,540052584	0,158023031
NCB5OR	1,540423012	0,011669429
FAM81B	1,540625437	0,085649302
SLC39A2	1,540719703	0,035819538
KIAA1324	1,541165677	0,001243501
MID2	1,541547884	0,023055632
MAST1	1,54231847	0,223137881
DNAH8	1,542553425	0,000367235
TSPAN18	1,542586414	0,03646963
NWD1	1,544565503	0,236718042
LOC729027	1,546735041	0,029334655
OR5B21	1,548457498	0,048296377
DPF3	1,550818352	0,205464195
BTBD7	1,551085664	0,012066658
NAIF1	1,55164162	0,059538191
FANCA	1,551676843	0,050050552

CTPS	1,551989915	0,065788655
CNGB1	1,554111295	0,054943384
SYT13	1,555174404	0,002892221
LOC730058	1,556044047	0,038480183
ZNF620	1,556124145	0,147972683
DACH1	1,558131436	0,17148202
RFT1	1,558358862	0,136830392
LOC727820	1,558384214	0,026835588
RASL12	1,558512178	0,364738769
DKFZP564B1023	1,558907121	0,040587578
PLXNA4	1,559036701	0,026431752
ERK8	1,55926034	0,303418693
LOC388965	1,559833331	0,009507666
FAM128B	1,560052485	0,106996559
LOC653256	1,560080957	0,011390945
CRIP2	1,560462609	0,264466072
METT11D1	1,561429069	0,132609947
MYH14	1,562464285	0,184885266
ZFX	1,562809648	0,039119427
DDX19-DDX19L	1,562932534	0,400648369
MGAT2	1,562985461	0,111347831
SSB1	1,563112785	0,00687655
FLJ41856	1,563140233	0,057860612
OR3A3	1,563805917	0,595553531
UFM1	1,563938285	0,03481792
LRP4	1,56547361	0,03309863
KIAA0625	1,565811096	0,072689434
C7orf62	1,566926408	0,016069207
GPR111	1,567288004	0,183837131
LOC729570	1,567668201	0,085912625
PYGO1	1,569314625	0,012570517
C3orf65	1,57080959	0,001094315
DUSP3	1,571065383	0,077931512
REN	1,571486495	0,423312913
KIAA1143	1,57158927	0,075934468
POLM	1,574772708	0,115972381
TCIRG1	1,575042492	0,209198667
PSRC1	1,576697125	0,047056359
ACMSD	1,576706937	0,117576161
LINS1	1,578069935	0,065866934
LOC392221	1,579336923	0,69150201
FGF11	1,579675712	0,14817716
OR2A2	1,582897192	0,036905035
LOC729380	1,583304461	0,003686865
LOC643711	1,583877924	0,022316425

C1QTNF8	1,584096872	0,048039874
CREBL1	1,586495374	0,135419291
RUSC1	1,586959345	0,231004888
NBPF9	1,588084628	0,082582608
TPCN2	1,58851855	0,088755208
IL16	1,591274723	0,006280075
LOC730589	1,592237009	0,038111629
MPP1	1,59337082	0,200619134
CYP2C19	1,595157383	0,069290934
EPHX2	1,595520856	0,017291018
LOC647591	1,59625214	0,017331951
OR4C15	1,596336039	0,097208173
NUDT15	1,59680539	0,016831716
KDELC1	1,597315297	0,076322182
ZNF136	1,597906484	0,002162668
SNAP47	1,599093071	0,060774101
KRTHA4	1,599426922	0,077584874
TMEM64	1,600472821	0,000300308
F9	1,602894175	0,106153957
SPG3A	1,603002366	0,11982324
LOC727861	1,603063311	0,139659575
MMP9	1,603276669	0,464748802
NOP10	1,60347275	0,074436392
EPX	1,60356343	0,006359904
TRIM54	1,603782662	0,310623537
SLC4A9	1,605169619	0,061718078
LOC645397	1,605794885	0,004931041
LOC729014	1,60624406	0,326366426
FLOT1	1,606771883	0,293754083
LOC643275	1,606969282	0,069702743
TRIAD3	1,607015253	0,143707751
BTBD11	1,60825383	0,043439388
LPHN2	1,609047194	0,097759565
PQLC1	1,60981354	0,1449943
CBFA2T1	1,609858164	0,026925725
TDG	1,610638202	0,07156408
FAM26F	1,61109121	0,01120548
HDAC5	1,611759594	0,054314736
RNF150	1,612510382	0,210795574
OR51F2	1,612534359	0,12952748
C16orf3	1,613000027	0,124912828
LOC728011	1,6147338	0,047698617
ATP6V0D1	1,615059185	0,168890119
LILRB3	1,616208016	0,081089192
MRPS36	1,616627927	0,135427183

LOC731592	1,617685552	0,116767226
HS2ST1	1,617855527	0,109632307
CYP7A1	1,618484517	0,293341247
NR4A3	1,618835612	0,141609428
WDR34	1,620221037	0,028109811
FLJ10618	1,622050876	0,105841695
TDGF1	1,625487625	0,022054924
MGC26605	1,626550678	0,224483839
C21orf123	1,628170051	0,000111311
TUBB4Q	1,629023982	0,001808253
TSGA13	1,629777539	0,21148835
TENS1	1,630520862	0,332663739
OR10G3	1,630607416	0,008505054
LOC644165	1,631459113	0,138945226
TMEM45B	1,632600218	0,042679001
C4orf49	1,633406589	0,142155629
THNSL1	1,634334311	0,076491451
IQCF3	1,634802873	0,081883946
ABCE1	1,636236607	3,76E-50
KIAA1853	1,636440394	0,064418467
CNTFR	1,637656545	0,099065991
KHDRBS3	1,637838042	0,003752545
SLAIN2	1,641789794	0,141092152
CHRNA6	1,642865329	0,297497817
GRM8	1,642874284	0,081376164
HSPC182	1,647359611	0,073157352
PCSK2	1,648034495	0,000320619
ABCA1	1,64953483	0,092970838
UXS1	1,651632086	0,065706412
LOC728579	1,652584048	0,129770896
C19orf19	1,652649357	0,088408073
NEUROD4	1,653227042	0,352647797
REPS1	1,65376618	0,170662236
LOC728528	1,653953389	0,01955214
LOC730133	1,654973541	0,020520296
TCL6	1,655951123	0,174390807
PBOV1	1,660750781	0,074787879
RNASE10	1,662405558	0,102294956
LOC729285	1,662972785	0,572737264
ARID5B	1,663615904	0,811545043
RUNDC3A	1,666108005	0,183457592
LOC731797	1,666428341	0,176741412
C5orf25	1,667398251	0,067777379
RHOBTB2	1,668098461	0,076497456
LOC196993	1,668536952	0,222058813

SAMD4B	1,669763785	0,000426526
ROMO1	1,673620046	0,003595985
LOC727781	1,674611417	0,03955511
JSRP1	1,67551613	0,067705124
H2AFX	1,677156687	0,019427978
SPATA7	1,677400933	0,151581811
CLTA	1,677974604	0,101056928
PLD2	1,679956577	0,328860731
PLB	1,68134868	0,214136129
C10orf67	1,684104148	0,031382125
KRTAP10-10	1,684476605	0,200098919
FAM177B	1,685068432	0,213358612
TBX5	1,686295975	0,071930121
ZNF133	1,686737564	0,104291965
EXOC3L2	1,687664583	0,189722269
TYW3	1,689242416	0,017090496
LOC646730	1,692682153	0,153014773
C1orf165	1,694454285	0,035872308
TGM1	1,696001396	0,236882763
LOC730185	1,697268941	0,068123194
C12orf60	1,697577143	0,017402446
LOC730142	1,697708618	0,015710658
LOC136242	1,697761033	0,036004007
LOC729585	1,699432297	0,004987162
KCNH5	1,700130724	0,21896496
LOC647855	1,700213095	0,019416263
SLC5A11	1,701527675	0,062555242
C9orf80	1,702047711	0,101998953
TTC24	1,702244168	0,016669895
PKD2	1,703747495	0,043714774
COL4A5	1,704342846	0,032380993
CCDC66	1,704498037	0,053377924
OR4M2	1,706052927	0,083816393
FLJ44606	1,706294794	0,549272969
PHF8	1,7068963	0,031427905
C17orf78	1,707458905	0,010602951
PIK3C3	1,707574557	0,192052406
PP8961	1,708811781	0,192071377
ABCA5	1,710812439	0,027979139
FOXA2	1,712283749	0,078448493
NDUFS3	1,712965784	0,270745326
TACC2	1,71312672	0,213718838
MAPKAP1	1,714103093	0,015799048
LGR7	1,715581194	0,115972898
TSLP	1,716677788	0,291768299

PLEKHH2	1,71690205	0,077266997
LOC642345	1,717958334	0,020989949
RADIL	1,718061384	0,000244958
AMHR2	1,718352879	0,033071402
GCNT6	1,719456232	0,023618502
GPD1L	1,720116113	0,02203978
VHLL	1,72036507	0,922418843
LOC727899	1,72122552	0,187604764
LHFPL4	1,721812733	0,011772176
CTTN	1,721962261	0,132895947
MAN1B1	1,722238575	0,18483178
C7orf60	1,722573968	0,103970154
TRRAP	1,728265476	0,056871694
ANKRD34B	1,728523038	0,076993623
PLEKHG5	1,729721378	0,006215822
RPP40	1,730478637	0,055663004
MNAT1	1,731138285	0,13772321
VMO1	1,731517251	0,153863121
RSRC1	1,735364368	0,053566672
LOC730022	1,73807473	0,105013158
LOC387804	1,7394601	0,007054444
HEATR5B	1,740164607	0,019641674
hCG_2040572	1,740879826	0,113045307
ITPA	1,740970674	0,19710886
GPX3	1,741309918	0,051463436
TTC34	1,741425028	0,361646196
LOC729575	1,741712896	0,005011009
KRT42P	1,746197839	0,034059232
HIST1H4L	1,746738833	0,138401499
LOC730923	1,748273679	0,285342458
LOC389203	1,748614379	0,024300121
C16orf53	1,748908057	0,153954296
LOC390364	1,749503281	0,013588918
LOC732144	1,75040839	0,027109028
TMEM116	1,751411498	0,082073892
FLJ20259	1,751625474	0,000130503
LOC646831	1,753749769	0,201044898
C15orf53	1,753847119	0,245388192
LOC730674	1,754336014	0,015548886
GNL1	1,759467177	0,367077041
NDUFB4	1,760840719	0,158876032
AIP	1,762139361	0,228253516
GSTT1	1,762770901	0,206901169
RGS9BP	1,762946356	0,041156105
FGF14	1,763198513	0,0414348

LOC643134	1,763777174	0,178316209
TCEA1	1,765404409	0,001823409
LTB	1,765550172	0,068411451
LOC441546	1,765880047	0,056926821
CLEC3B	1,766428169	0,214696497
MLPH	1,767742276	0,032807915
RELN	1,768083707	0,068945331
LOC388946	1,768397363	0,073351809
NAPA	1,769258075	0,023357474
C1orf177	1,774119526	0,151012205
ZNF135	1,77503061	0,09521962
FAM26D	1,778146169	0,275771431
ANAPC2	1,779347681	0,166681211
LOC149773	1,7835	0,101399265
HN1	1,784054727	0,118524595
LOC728561	1,7845135	0,1009896
HLA-DQB2	1,787253466	0,123018218
RUNDC2B	1,788246983	0,016754252
VAT1L	1,788955905	0,099326634
EBI2	1,790326403	0,117373362
ACTL6A	1,790810428	0,025329909
SHC1	1,792702113	0,089206009
ECH1	1,79271776	0,054815193
AGMAT	1,793665979	0,038835387
LOC729281	1,79406125	0,219672448
STRN	1,794278874	0,165014892
CX3CL1	1,797060915	0,138159644
TMEM52	1,797406609	0,016001679
EFEMP2	1,799319101	0,284992246
OR51B2	1,803141134	0,077587712
ZNF493	1,803284583	0,00472275
HIST2H2BE	1,803507136	0,000810838
GATC	1,803820428	0,004149585
SH3PXD2B	1,807157487	0,040944641
ORC2L	1,808044277	0,120043181
ATP2A1	1,813042942	0,032127339
ZNF551	1,813210881	0,074970311
FY	1,814462672	0,137633382
C9orf139	1,819563816	0,222351042
C16orf79	1,820962542	0,004618373
ATE1	1,821120561	0,001871217
ACRC	1,823410634	0,235176259
LOC644926	1,824701382	0,256192482
SPATA6	1,825314932	0,002703114
FLJ34389	1,82626035	0,06906749

SLC6A3	1,828080607	0,122547095
TMEM70	1,828415689	0,01360349
LOC731220	1,828617115	0,061857767
LOC731040	1,82892344	0,091268143
ST14	1,830267973	0,226579612
LOC90120	1,833062177	0,138082434
C15orf32	1,834168853	0,014879755
LOH3CR2A	1,834388758	0,095013227
MORC2	1,837146777	0,011100089
DAB2IP	1,842298605	0,005246966
LOC647174	1,848326447	0,18579553
AK7	1,849477218	0,187451463
ASXL1	1,84961273	0,030544915
CST6	1,849730933	0,059518477
CD8B1	1,84995854	0,206094528
FGF8	1,852186727	0,10889054
SDC1	1,852219954	0,291923108
PTS	1,852759306	0,092028879
PTPRR	1,852880269	0,141343216
MRPL48	1,854904295	0,256652693
PHYHD1	1,855113783	0,149817363
CLPTM1	1,856110175	0,006901289
LOC651008	1,856752145	0,022908093
HOXD9	1,857932608	0,172503862
HIST1H3D	1,860164136	0,086229966
SAMD11	1,86066443	0,035338306
LOC653163	1,861387916	0,142217912
ATP6V0B	1,862498154	0,085569548
C2orf59	1,864718982	0,478817725
GPHB5	1,867863779	0,092662952
MEFV	1,868084151	0,144509143
TRIM41	1,870254513	0,029653848
LOC728804	1,872346915	0,014807326
ELA1	1,87267021	0,000861033
E2F1	1,872694835	0,02392266
LOC643100	1,873667677	0,070008809
LOC652599	1,874104579	0,14533202
LOC92017	1,874990198	0,078273133
ZNF611	1,876662631	0,039242709
ARTS-1	1,876721229	0,327076825
DNAI1	1,877635118	0,229683505
F7	1,878524686	0,005841377
HIST1H1E	1,879546606	0,018860349
ATP6AP1	1,883922416	0,256008453
GAS2L1	1,883959919	0,055436495

CLEC9A	1,888378985	0,213761181
ADAMTSL5	1,888604119	0,106502992
BRPF1	1,889277196	0,103839896
RNF7	1,889334413	0,088369332
PAX4	1,890715464	0,16473332
GALNT7	1,89095947	0,008820103
GPR109A	1,891567912	0,154476177
C15orf16	1,89244021	0,090469335
LOC729871	1,892507819	0,094091302
MCAM	1,892994874	0,187293046
CCDC85A	1,894053421	0,053378432
Klkbl4	1,896118446	0,020321066
SLC13A1	1,900288791	0,326968339
AMOT	1,903270967	0,069315038
LOC391609	1,903704109	0,060836809
TMEM117	1,906558369	0,062860953
LOC730217	1,907638367	0,30437445
NRIP1	1,908478808	0,131194962
KIAA1949	1,912161042	0,013802981
IDI2	1,913096672	0,118338737
SPP1	1,914025451	0,045536004
NRXN2	1,91530316	0,033525106
GRAMD1C	1,915437775	0,071306807
CLDN9	1,915989223	0,084162465
LOC728966	1,918004298	0,088379969
FMO3	1,921669459	0,01440666
LOC728613	1,925784435	0,006958517
TBC1D26	1,926264502	0,06935255
FLJ37300	1,927755092	0,193922081
DRD5	1,929114555	0,20009602
LOC728630	1,930937925	0,219608217
KIAA0296	1,931007697	0,024807162
LOC728117	1,932440479	0,371497807
IL1B	1,934475395	0,560049565
COPEB	1,934834413	0,05689961
GPR110	1,935874142	0,066425662
VRK1	1,937311396	0,033142823
KIAA1920	1,937755728	0,051858142
BIN1	1,938570888	0,142506186
TNP2	1,939595681	0,037434975
FLJ45224	1,941561223	0,024398239
LOC644402	1,941768198	0,005127567
TMEM50A	1,942046823	0,800173375
FAM118A	1,943046373	0,099654627
AHDC1	1,944276182	0,003920754

BBC3	1,946203932	0,154926313
FRMPD1	1,947577541	0,007190268
PTPRK	1,949818709	0,153990497
IL3RA	1,95437149	0,064552672
NIPA1	1,95457652	0,004933622
LOC400856	1,954640703	0,009737432
ZAR1	1,954960659	0,0015027
QARS	1,955178023	0,085922432
GSS	1,958469665	0,062312338
HCFC1	1,95956795	0,030922645
OR5AR1	1,962411669	0,030022726
NSMCE4A	1,964856968	0,200008542
LOC730018	1,965434731	0,016492024
CREBBP	1,968822596	0,011147836
GZMH	1,969522719	0,220626572
TBL3	1,973541637	0,066996309
S100A10	1,975565406	0,070570239
SLC27A3	1,977209426	0,288734566
OR11G2	1,97732168	0,655448321
HIVEP3	1,978414542	0,112404288
RAI2	1,979379588	0,103874583
OR10K2	1,980797707	0,155861388
CITED1	1,986521559	0,007972678
LOC728854	1,988244795	0,032511176
OR1L1	1,994060057	0,109009536
CGRRF1	1,996308413	0,052591134
LOC284723	1,996336284	0,201531843
MPHOSPH8	1,997334772	0,036590059
OR7G2	1,997578495	0,063853186
LYZL4	1,999229335	0,059766851
SASH1	2,000086656	0,033362908
IRGM	2,000510788	0,092634816
LOC645277	2,000726367	0,088349211
PRKXP1	2,000846535	0,193791426
HECA	2,001217453	0,083226573
MXD4	2,001471836	0,021530098
USP50	2,001571412	0,237868435
C6orf208	2,005072318	0,065671906
MMP26	2,007851054	0,025299337
TEX10	2,011267833	0,04047836
SCN2B	2,011293396	0,213246686
SFTPB	2,015679999	0,009148925
LOC732248	2,016057072	0,015879537
MAGEB6	2,016467201	0,168397529
C3orf24	2,016771055	0,020307058

LOC389541	2,017745613	0,157958754
NUDCD3	2,019785136	0,217684851
EIF5A2	2,021772403	0,042665672
AKR1B10	2,023074702	0,048693991
PIGR	2,028187508	0,080888969
TMEM149	2,029305049	0,093351302
ZNF488	2,029733568	0,000283287
PCDHA12	2,030042469	0,261223683
SDSL	2,032940043	0,06544675
SIX1	2,033016446	0,012142258
LOC441119	2,033124168	0,005397643
BMX	2,036806058	0,103590125
PCTK3	2,037636587	0,041919295
LOC647718	2,040992078	0,06922146
RNF8	2,041028312	0,160141361
TP53RK	2,041311582	0,295708019
PKDREJ	2,043117455	0,0038373
ZPLD1	2,043378772	0,114105749
XPO1	2,043771873	0,044767623
NFATC2IP	2,046274905	0,04589258
DUSP27	2,048217555	0,230892504
LOC645586	2,049964778	0,017562562
PDPK1	2,050046105	0,318018919
SPG7	2,050840212	0,026514733
CAPN2	2,053791052	0,294251249
MLH3	2,054162057	0,023828182
HIST1H3G	2,054462499	0,053737342
CATSPER3	2,054619292	0,087972782
HEMK1	2,055588872	0,062948747
ADCK2	2,055892197	0,072902159
HIST1H3E	2,05711042	0,170415062
LOC729094	2,057919546	0,388545407
TAS1R2	2,059383679	0,032303242
RMND5B	2,061553754	0,058154146
FLJ20249	2,06379982	0,006089933
RRM1	2,064679657	0,106942953
OPALIN	2,064950826	0,041496667
ABCC2	2,066292861	0,040981439
ASB5	2,069301617	0,109220381
HIST2H3A	2,070370691	0,003174621
LOC652708	2,070483876	0,282548587
ARC	2,071176746	0,000834692
LOC131691	2,07418276	0,079956553
LOC728752	2,075059696	0,07202693
JMJD1A	2,075091651	0,145445473

DENND4B	2,076071533	0,003650686
FDX1	2,077220009	0,124372129
LOC645946	2,079306554	0,081729178
TBRG4	2,079684594	8,77E-05
OR10P1	2,07980646	0,032102579
TNNI3K	2,07988933	0,100979079
GRIN3A	2,083064869	0,007038346
C20orf41	2,083067312	0,120756435
SLMAP	2,08678921	0,078664485
RHOT2	2,090426875	0,20307591
LOC647470	2,090890889	0,065058338
PARG1	2,097068111	0,115394518
BLMH	2,099033624	0,002712611
ILKAP	2,099239786	0,168076983
TENC1	2,099763355	0,002823842
SLC38A10	2,101906164	0,105643174
GGT2	2,103982188	0,000340719
TAF9	2,104174128	0,025513656
GTF3C3	2,112806321	0,07035735
CDK7	2,114396789	0,034694525
MAP2	2,116299189	0,692091795
C15orf49	2,116752765	0,008556008
KCNJ15	2,120154005	0,040140704
MLL2	2,122762209	0,064802742
ARPM2	2,126083328	0,079176183
GTF2H3	2,126663738	0,063298144
MYT1L	2,127377286	0,016913088
LOC730267	2,129395688	0,140425093
YWHAH	2,134652122	0,078199115
TALDO1	2,135245641	0,047170669
CORT	2,140038462	0,006273746
DKFZp761H039	2,141613107	0,223955343
CNTNAP2	2,14346068	0,027059262
TMEM16A	2,143730306	0,693063887
LOC646821	2,145238301	0,2460957
LOC340351	2,146046284	0,069798467
FAM164C	2,147881861	0,071640298
PPM1G	2,148597107	0,204804761
EDAR	2,150498901	0,017515722
GP1BA	2,151811378	0,111927952
RRP22	2,153086503	0,436984386
SMAD5	2,154761139	0,04513879
RNASEH2B	2,157060372	0,125442441
GAR1	2,159432453	0,063972126
C1R	2,159895317	0,033598383

LOC650968	2,161434159	0,00121907
C5orf32	2,163054301	0,027531513
TMEM165	2,163342694	0,029274407
IGSF11	2,165659051	0,19096407
MTCH1	2,165772202	0,068645018
LOC729635	2,166401072	0,020690921
DDEF1	2,171885231	0,088151822
TRIM35	2,173022811	0,018028658
LOC149224	2,175330851	0,071657088
SLC16A2	2,17951569	0,034058828
IFITM5	2,181378903	0,071449778
RNF186	2,182649215	0,018656577
POLE2	2,183649274	0,157734831
LOC390735	2,183883812	0,006257543
TSG101	2,188810084	1,56E-60
KLHL34	2,190226575	0,429823665
CASR	2,192290572	0,015657917
C1orf135	2,197066774	0,11727784
ADSL	2,197768597	0,023036361
LOC644717	2,199428186	0,029958158
TNRC4	2,201102218	0,208688901
ZNF570	2,207525924	0,083915409
PRKACG	2,20823233	0,147915054
DPPA4	2,208911453	0,001233686
PRELID1	2,208964019	0,085486978
STK3	2,210566306	0,043854661
TCF23	2,211413041	0,61575442
RABGAP1L	2,213146721	0,064254143
CSMD2	2,213340629	0,199236319
HSPCA	2,213857373	0,024247943
TLR9	2,214557562	0,412754595
JMJD1C	2,217775079	0,037049963
FLJ12331	2,219099027	0,02885302
OPN5	2,219223967	0,053721113
LOC727756	2,222596244	0,049120101
PKD2L1	2,223896429	0,02611884
LOC729722	2,230232978	0,075645834
NRG2	2,230433906	0,04601843
SFTPC	2,23272918	0,056762138
SEC23A	2,235243736	0,107941944
OR56A5	2,240098616	0,000722617
BEND3	2,242305188	0,188476065
LOC728475	2,242526471	0,020500519
VMD2	2,250674683	0,10395452
UCHL1	2,252560637	0,121762872

LOC120824	2,254708704	0,046257947
FAM24A	2,255474863	0,002862433
C9orf7	2,261452721	0,005292733
SCGB1D2	2,263874881	0,001401469
MGC33556	2,265109139	0,462820237
KRTHB6	2,26607827	0,095983663
JAZF1	2,267812617	0,108490321
LOC729534	2,267869571	0,456467939
PAX8	2,268343523	0,102500635
OR6C4	2,272078175	0,043826763
VPS35	2,273980549	0,017982579
SERPINB1	2,276522472	0,050608245
ZCCHC9	2,276926327	0,004464305
GPR116	2,281038165	0,017421787
LOC728659	2,285411224	0,037981443
HAGH	2,287189814	0,112456227
TNF	2,290839242	0,138720028
PTPN23	2,291679669	0,291090477
OR2A14	2,297912888	0,078856174
VWA3B	2,301627266	0,197805548
GEMIN7	2,304724096	0,023303436
VBP1	2,309550952	0,038805198
OR10Q1	2,309575535	0,042118389
IL33	2,310786332	0,012445994
LOC728751	2,311148437	0,0382095
CLIP2	2,314526926	0,065724943
C1orf93	2,32168123	0,144712925
SENP1	2,324478812	0,001311846
CCNB3	2,326310082	0,182155647
ASCL5	2,328884829	0,015542876
PTPRG	2,332418876	0,051278008
LYPD2	2,334047862	0,188204384
LOC729489	2,338508909	0,000317288
EEF2K	2,340437944	0,160801128
LOC645914	2,341306483	0,013717995
RASGEF1C	2,343270099	0,023964837
SRR	2,346525862	0,068356913
NOX3	2,348235514	0,042773211
DEM1	2,354017659	0,045057676
TPTE2	2,354161241	0,412184969
FUT9	2,354170153	0,032438195
ROD1	2,362888376	0,021649938
CENPV	2,366043062	0,08215517
BRD4	2,370680324	0,066902904
MRPS18C	2,377942512	0,008536002

SPATA12	2,380834023	0,065672374
HMBS	2,38531566	0,106201429
ATP1A1	2,38611759	8,02E-44
LOC390335	2,386881036	0,035867593
LOC645379	2,388417023	0,040186779
hCG_1793136	2,396151329	0,067566416
HRMT1L6	2,396459064	0,021138127
POLD2	2,397563779	0,09480556
C11orf44	2,400915336	0,171584333
METRN	2,408138504	0,076734447
hCG_2045751	2,409388963	0,010114318
ASAH1	2,411150727	0,021597838
CLPP	2,419918296	0,087225478
TESSP5	2,420199489	0,000922277
TM2D3	2,421128256	0,059654586
HNRPL	2,421425857	0,000693895
TMEM111	2,424008927	0,031982708
SRY	2,424871009	0,156861626
PIAS1	2,42783949	0,241368574
ANKRD5	2,431071541	0,033081808
LOC91120	2,432146072	0,001967423
FKBP7	2,433638811	0,61084446
hCG_1657980	2,437958462	0,016930056
RNASE1	2,438023626	0,116564494
RGP1	2,439494387	0,255184971
GPR157	2,446175304	0,132194726
LCN7	2,446364228	0,133770063
MYL3	2,450449345	0,024617536
DEFB136	2,450668591	0,065255308
MEIS1	2,450726005	0,023962615
KLHL6	2,452850569	0,084526659
LOC347549	2,453406291	0,022150753
WBSCR14	2,454318002	0,201934507
DDX55	2,45815122	0,132072632
HTATIP	2,459296602	0,076038379
LOC646056	2,474224768	0,028808568
ARSA	2,474642914	0,047355746
IMMP2L	2,481915949	0,113835005
LOC642995	2,48270731	0,03062391
LOC730364	2,483550899	0,001812918
ODF1	2,492289387	0,00690212
LOC90313	2,502847633	0,152033154
HTR3A	2,503790231	0,390766639
OR8U1	2,507030885	0,014199526
RPLP0	2,508033901	0,089911414

ESRRB	2,511261979	0,050763585
GPR142	2,511726805	0,089038991
E2F4	2,51236115	0,025928732
KIAA1811	2,514195969	0,656364946
HOXD12	2,520137592	0,05087198
RPLP1	2,520228501	0,053515934
LOC730996	2,525658232	0,007826208
HSPG2	2,537764173	0,014806331
SRGAP2P1	2,541891852	0,18363127
LOC729943	2,555086392	0,127392813
SLC10A2	2,562367467	0,280959179
CLLU1	2,562700183	0,051512645
LOC727958	2,563348993	0,220664749
LOC730608	2,563672066	0,058823488
CDC42BPB	2,564506354	0,26397819
TNFAIP3	2,574363819	0,160324928
LOC651593	2,574794646	0,033806821
SEC24B	2,580960358	0,015319481
KRTAP5-5	2,582997947	0,037476977
HLA-DRB1	2,585543025	0,005002928
PDCD1LG2	2,587256156	0,038441564
CYP11B2	2,591012647	0,145589163
EAF1	2,593175474	0,052292831
LOC442227	2,595967965	0,171356718
TOR1B	2,597862497	0,000768153
GRAP2	2,601422672	0,032960244
LOC643524	2,602160679	0,051838082
MRPL27	2,60338477	0,03902267
LCP1	2,604628873	0,086485649
LOC728286	2,606565927	0,093073216
SLC37A3	2,60847008	0,011913587
C16orf81	2,610652169	0,041809764
C21orf59	2,610719743	0,030404558
SCIN	2,616079592	0,262335637
PNMA6A	2,616606129	0,003839608
HDAC9	2,620734049	6,67E-05
KLHL12	2,62105689	0,049328228
SLC8A1	2,623698196	0,045183053
ITPRIP	2,624795977	0,014121674
RPH3AL	2,628305008	0,012683783
CUL4B	2,636785378	0,111369402
DEFB129	2,644074137	0,042050949
GSTM2	2,650632528	0,009412287
ZC3H4	2,651392179	0,023833294
IFNAR1	2,663090507	0,002563242

GTF2B	2,663656543	0,119735827
LOC728570	2,670409511	0,111241041
FLJ44653	2,678616227	0,09008211
MGC48628	2,684633229	0,063794949
ADAMTS16	2,688430579	0,092047758
C6orf48	2,704157854	0,078853404
HMGB4	2,709949294	0,058485339
LOC729702	2,713570174	0,081680093
C3orf63	2,715607112	0,019677348
PPARGC1B	2,717716925	0,189369625
RHBDL1	2,724334008	0,001143655
NDUFA10	2,726564347	0,017873897
PIK4CA	2,728264783	0,07217115
SDF2	2,73455033	0,039288484
LIPE	2,74014097	0,001268051
VIM	2,741160078	0,152759541
PIGV	2,741874767	0,012365682
FAM102A	2,743352231	0,111090985
RELA	2,744260611	0,067079926
DOCK5	2,745047164	0,039827321
GALK1	2,746049233	0,012762511
KRTAP26-1	2,750227921	0,006210554
CTAGE6	2,757607669	0,121299533
AD-017	2,758223197	0,183237338
LOC730668	2,767840987	0,086838079
PCYT1A	2,76998104	0,193665092
USP24	2,789854891	0,158429106
MASP1	2,793184362	0,030980622
FOXI2	2,804802372	0,000626905
TPD52L2	2,820134752	0,169254103
DNAJB14	2,820320786	0,017772016
LOC729227	2,839798888	0,095083919
LOC730135	2,83985084	0,002516884
SULT1A4	2,851769342	0,032427499
ARVCF	2,856442662	0,130808204
UGCGL1	2,857131155	0,013948895
LPIN3	2,858749277	0,126302134
LOC729866	2,859772802	0,183045463
LETMD1	2,86010717	0,054926705
RLF	2,865724208	0,083038115
DLD	2,86853896	0,133319743
PPP2R1A	2,869113029	0,082687434
COL4A3BP	2,8741518	0,066728419
LRP2	2,874210814	0,088432868
SLBP	2,874996705	0,018862321

HES1	2,878709507	0,033626818
GIPR	2,880202017	0,040042595
LOC730233	2,881132567	0,141922604
GGT6	2,882535628	0,154617086
LOC730218	2,883102752	0,370258381
LOC389722	2,89957878	0,000466923
SEMA5B	2,905286142	0,002819864
C22orf31	2,910602731	0,268158934
TDRD3	2,915636879	0,01155013
MDS032	2,918969432	0,089963388
LOC731150	2,920350146	0,000810015
MAP3K12	2,932068943	0,140778608
FLJ31485	2,935432467	4,68E-05
SUPT7L	2,93869515	0,026004062
KRTHB4	2,94117467	0,022799462
FLJ22419	2,945309375	0,069789086
GOLGA7	2,94793802	0,050365454
OOEP	2,963546046	0,076130686
LOC728976	2,963979665	0,001064464
EAF2	2,987545653	0,131821279
hCG_1642947	2,99775925	0,252080318
TAF1	3,000547117	0,01990618
CROCCL2	3,025187948	0,089360744
C9orf127	3,032539768	0,00985235
TBC1D3G	3,033654534	0,038616432
KLK1	3,049364051	0,053970223
OR1D4	3,061490153	0,120872079
KIR2DL1	3,073436929	0,000273214
LOC730003	3,079836253	0,019003535
GCDH	3,092809589	0,05594621
TMEM176A	3,093891977	0,141068782
ZNF473	3,096994022	0,046997074
FAM86A	3,100123869	0,184309763
TMEM55B	3,100272217	0,032084662
LOC645321	3,128191428	0,092996832
SPATA17	3,12935395	0,216487943
LECT1	3,130091809	0,046754859
LOC731223	3,13274005	0,055532175
SFTPA1	3,135018493	0,007393542
OPA3	3,136369373	0,071703667
BTG1	3,142464558	0,001746822
PAQR6	3,153807816	0,02853127
CTA-216E10,6	3,169903516	0,215535134
VIL1	3,187157129	0,154078782
TDP1	3,192739045	0,063123328

LGALS9B	3,197484623	0,020537193
OR14K1	3,197806553	0,056824963
ST8SIA6	3,199367713	0,225620476
RPLP2	3,208389391	0,000979671
NELF	3,217102834	0,621139671
NFIX	3,218437271	0,014020424
NUP62	3,226702462	0,038136848
LOC729663	3,227827387	0,022122708
AK3L1	3,228236078	0,07935106
CHD4	3,233560496	0,036209638
LHX5	3,237623761	0,180500602
DEFA1	3,26472128	0,035349925
TRPC7	3,271449912	0,304395473
KIFC3	3,272110324	0,005648047
LNX	3,277312542	0,127083878
ATAD5	3,277382112	0,018646434
TRIM38	3,278652237	0,154487889
SLC26A10	3,29478037	0,052975123
HSPB6	3,297259851	0,091829704
TOM1L2	3,320058162	0,058752476
CRELD2	3,337023658	0,007059352
HERPUD2	3,337424347	0,100828682
CTNNA2	3,342939656	0,049738852
C16orf71	3,367338798	0,068381016
TAF12	3,374312106	0,038887779
ATRX	3,378930393	0,001504488
LOC646450	3,393195372	0,003048262
LOC652455	3,40642128	0,108165591
JPH4	3,425822899	0,209187426
PLCG2	3,428789506	0,189282835
C21orf69	3,438755062	0,042894675
FOXP3	3,443818407	0,07338936
KIAA1950	3,450087902	0,171481616
LOC441872	3,457287989	0,053763326
SPRY4	3,46014863	0,083454699
KCNS2	3,470157884	0,062294119
NDUFA8	3,471569461	0,043422783
TAF3	3,488056485	0,059904827
ZNF206	3,493461675	0,034871978
NUDT19	3,49963897	0,439480831
RAD18	3,499933769	0,205732796
LOC643043	3,502734919	0,021842958
LOC648089	3,533387066	0,061640729
SPINK2	3,54528638	0,138187685
LOC730203	3,548547823	0,031016457

ZNF831	3,5569829	0,214032966
PTCD1	3,561566309	0,000377768
ARK5	3,574322559	0,082010516
TCEAL5	3,582111575	0,202382073
SYP	3,603469642	0,023169209
FLJ32549	3,606646295	0,056213177
COL4A4	3,60687463	0,083665919
SFPQ	3,611051565	0,001603181
PARK7	3,618286206	0,149285445
GLA	3,631023518	0,053374974
THAP5	3,6428714	0,289554517
SUMO4	3,675018198	0,155772942
FAM71D	3,692959227	0,045817421
LOC732231	3,696117615	0,004807352
ACTG2	3,719184579	0,121373763
C9orf61	3,72545194	0,059952353
YTHDC1	3,733664414	0,051192355
C19orf15	3,735762264	0,26533294
DDX11	3,751650493	0,03713776
NCBP1	3,752413796	0,027045458
EDA2R	3,770607126	0,011317609
TAF4	3,807141495	0,000458188
TRPM1	3,810698284	0,069173924
IL8RB	3,835696016	0,015148855
LOC644828	3,850735569	0,000233207
MMP27	3,88709308	0,243459812
TEX12	3,901907439	0,034856061
OR5V1	3,918622558	0,03599021
CETN1	3,958967509	0,024082644
BACH	3,971006793	0,230788034
ACCN3	4,005350658	0,019730873
SLC13A5	4,034527411	0,078552975
LOC387867	4,098752575	0,14706132
LOC400299	4,101808882	0,038205645
WDR93	4,122600015	0,01605849
CD70	4,126789089	0,159950912
ZCWCC3	4,237807673	2,08E-05
SOAT2	4,243664721	0,097225856
LRRC57	4,244741837	0,095345336
VDAC3	4,247728	2,93E-05
TDRD10	4,283233327	0,099318363
URM1	4,316990245	0,15094038
ATP6V1B1	4,328385508	0,215286936
LOC729895	4,335023598	0,000349607
SFRS1	4,340096597	0,001610987

SOSTDC1	4,353124351	0,022578296
LOC652458	4,398928488	0,003377627
C15orf39	4,509626115	0,01024764
GRPR	4,516629044	0,028902248
IQSEC1	4,544331049	0,033307148
LOC440295	4,546332594	0,062076211
LOC651123	4,600305347	0,017969753
ING1	4,653119807	0,075534936
POLD1	4,67913281	0,139719408
ICAM2	4,683369305	0,004947526
CD24	4,784022512	0,05905429
FAM9B	5,001604058	0,020922703
PTPRH	5,040732174	0,172179069
PEX14	5,179092593	0,064597744
VCY	5,2390308	0,104149999
HR	5,324793953	0,239613668
LOC647971	5,372035937	0,017737559
DAXX	5,389007549	0,011338755
DDX48	5,514380987	NA
RPS9	5,521242213	NA
WDR18	5,58099256	0,043437462
OTOG	5,665297509	0,007462557
SLC22A17	5,679009115	0,214655502
KRTAP5-6	5,759077821	0,107773401
F8	5,88260624	0,128502938
IDH1	5,893184975	0,070266968
NUDT17	5,927921384	0,236646035
NLF1	5,931169614	0,122311853
ERGIC3	6,456151705	0,089662772
NTRK1	6,48532038	0,069380222
C12orf35	6,642873194	0,000220576
PABPC1	6,655353895	0,174022097
LEFTB	6,744232068	0,113756601
SLCO1A2	6,894816354	0,191066945
SUMO2	7,264131383	0,000275662
CHAF1A	7,622095325	0,006236657
HNRPC	7,6766137	0,007807733
TRIM33	7,715805449	0,002447176
NPAT	8,954291034	0,003287117
CASP8AP2	9,325322047	0,001687821
ATF7IP	13,61672513	0,002753892
UBE2I	16,59377312	0,010777605
UBA2	17,99220961	0,016870682
SAE1	21,50683055	0,000654819
