# Supplementary figures and images for: The SUMOylation Pathway Restricts Gene Transduction by Adeno-Associated Viruses
Source: PLoS Pathog. 2015 Dec 1;11(12):e1005281. doi: 10.1371/journal.ppat.1005281 (PMC4666624; doi:10.1371/journal.ppat.1005281)

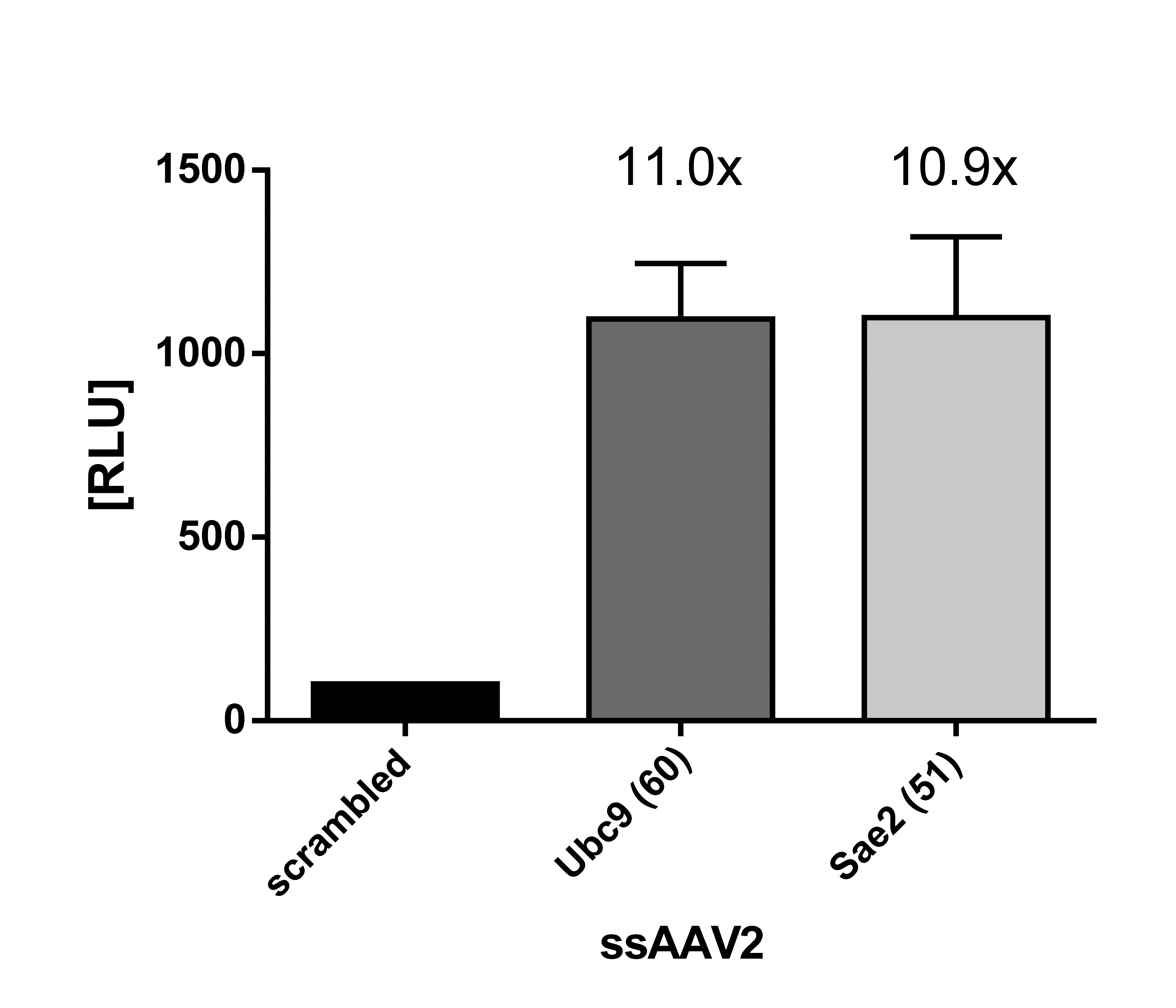

Supplement: S1 Fig — Cells were treated with siRNA targeting Sae2 or Ubc9 for 48 h followed by transduction with ssAAV2 encoding renilla luciferase. Shown are the mean values and standard deviations of the RLU of three independent experiments and normalized for treatment with scrambled siRNA. Data was normalized to scrambled siRNA, which was set to 100. (TIF) [file ppat.1005281.s002.tif]

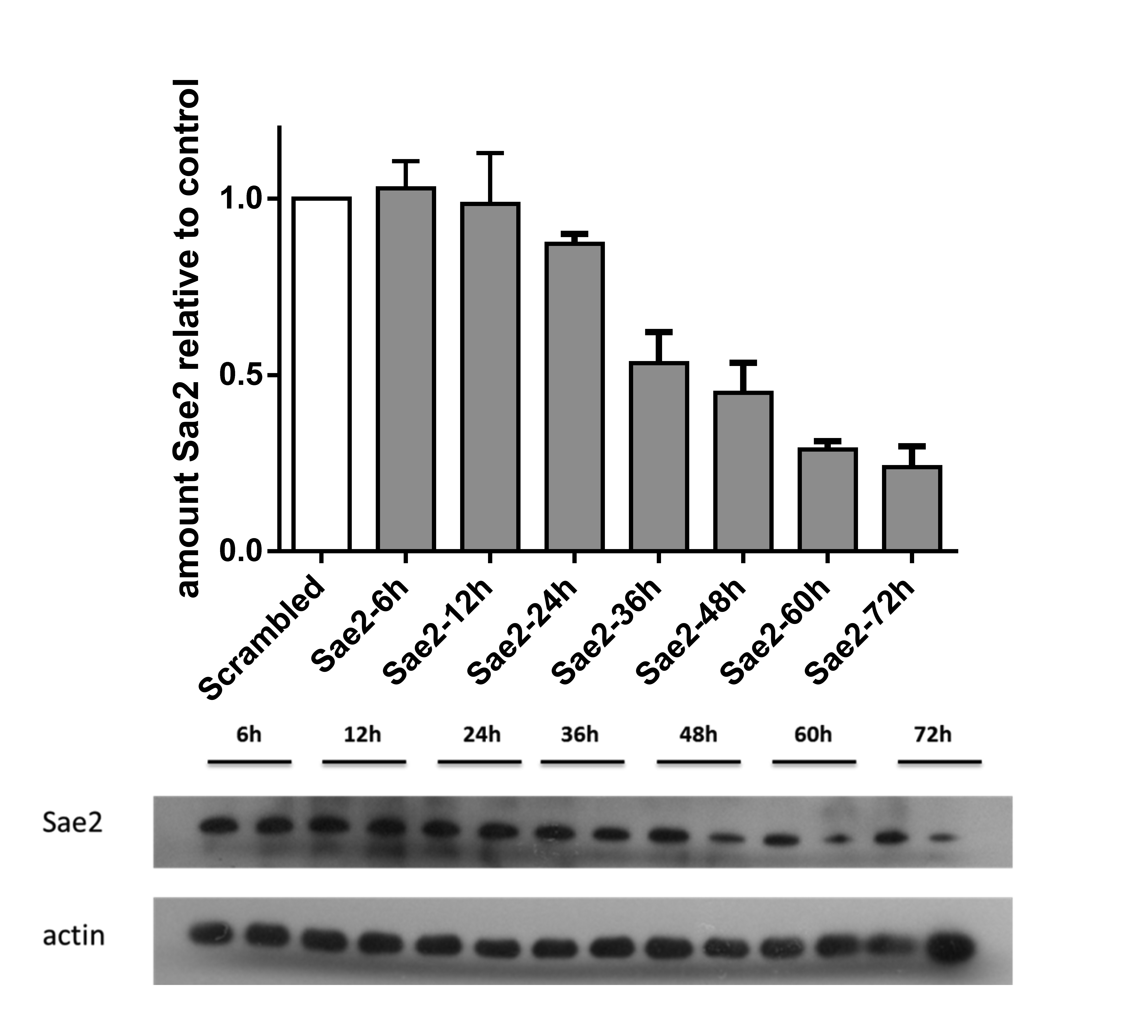

Supplement: S4 Fig — Hela cells were transfected with siRNAs targeting Sae2 or with scrambled siRNA and harvested at time points indicated. Sae2 expression was determined by western blot and quantified by ImageJ. The graph shows relativee2 expression levels normalized for each time point to actin and to Sae2 signals of cells transfected with scr siRNA (set to 1). (TIF) [file ppat.1005281.s005.tif]
